# Health Equity Rounds: An Interdisciplinary Case Conference to Address Implicit Bias and Structural Racism for Faculty and Trainees

**DOI:** 10.15766/mep_2374-8265.10858

**Published:** 2019-11-22

**Authors:** Joanna Perdomo, Destiny Tolliver, Heather Hsu, Yuan He, Katherine A. Nash, Stephanie Donatelli, Camila Mateo, Cynthia Akagbosu, Faraz Alizadeh, Alexandra Power-Hays, Tyler Rainer, Daniel J. Zheng, Caroline J. Kistin, Robert J. Vinci, Catherine D. Michelson

**Affiliations:** 1 General Academic Pediatrics Fellow, Boston Children's Hospital; 2 Resident, Department of Pediatrics, Boston Children's Hospital; 3 Resident, Department of Pediatrics, Boston Medical Center; 4 Assistant Professor, Department of Pediatrics, Boston Medical Center; 5 Assistant Professor, Department of Pediatrics, Boston University School of Medicine; 6 Instructor, Department of Pediatrics, Boston Medical Center; 7 Instructor, Department of Pediatrics, Boston University School of Medicine; 8 Resident, Department of Neurology, Boston Children's Hospital; 9 Health Services Research Fellow, Boston Children's Hospital; 10 Professor, Department of Pediatrics, Boston Medical Center; 11 Professor, Department of Pediatrics, Boston University School of Medicine

**Keywords:** Implicit Bias, Structural Racism, Faculty Development, Interdisciplinary, Diversity, Inclusion, Health Equity, Interprofessional Education, Case-Based Learning, Editor's Choice

## Abstract

**Introduction:**

The medical community recognizes the importance of confronting structural racism and implicit bias to address health inequities. Several curricula aimed at teaching trainees about these issues are described in the literature. However, few curricula exist that engage faculty members as learners rather than teachers of these topics or target interdisciplinary audiences.

**Methods:**

We developed a longitudinal case conference curriculum called Health Equity Rounds (HER) to discuss and address the impact of structural racism and implicit bias on patient care. The curriculum engaged participants across training levels and disciplines on these topics utilizing case-based discussion, evidence-based exercises, and two relevant conceptual frameworks. It was delivered quarterly as part of a departmental case conference series. We evaluated HER's feasibility and acceptability by tracking conference attendance and administering postconference surveys. We analyzed quantitative survey data using descriptive statistics and qualitatively reviewed free-text comments.

**Results:**

We delivered seven 1-hour HER conferences at our institution from June 2016 to June 2018. A mean of 66 participants attended each HER. Most survey respondents (88% or more) indicated that HER promoted personal reflection on implicit bias, and 75% or more indicated that HER would impact their clinical practice.

**Discussion:**

HER provided a unique forum for practitioners across training levels to address structural racism and implicit bias. Our aim in dissemination is to provide meaningful tools for others to adapt at their own institutions, recognizing that HER should serve as a component of larger, multifaceted efforts to decrease structural racism and implicit bias in health care.

## Educational Objectives

By the end of this activity, learners will be able to:

1. Identify and analyze the effects of implicit bias and structural racism in clinical scenarios.
2. Describe the historical context and present-day role of structural racism and its impact on the health care system.
3. Employ evidence-based tools to recognize and mitigate the effects of personally held implicit biases.
4. Use newly learned strategies to combat structural racism at the institutional level and reduce the impact of implicit bias on patient care and interprofessional relationships.

## Introduction

There is increased attention in the medical community to addressing health inequities^1^ by directly confronting both (1) structural racism, defined as the cumulative effects of policies, institutional practices, cultural representations, and other norms that work together to perpetuate racial inequity,^2^ and (2) implicit bias, defined as learned stereotypes and prejudices that are automatically and unconsciously exercised. Structural racism and implicit bias both lead to poorer-quality health care and outcomes,^3–6^ yet as an academic medical community, we have few interventions to mitigate their impact on care.^7^

In the past decade, several training programs have developed curricula on structural racism and implicit bias for medical students and residents.^8–15^ However, few curricula have focused on faculty members as learners rather than teachers of these topics or engaged faculty and trainees together.^16–18^ This paucity of either team- or faculty-focused curricula is problematic. First, faculty not only participate in direct patient care but also function as team leaders and models of professional behavior for trainees. Second, as trainees increasingly seek to learn how to develop strategies to address racism and implicit bias, faculty should possess the comfort and skills needed to facilitate these conversations.^7,17,19,20^ Finally, institutions often unfairly rely on a limited number of volunteer faculty—frequently, people of color who are designated or feel personally responsible—to teach on these topics.^21^ Engaging a broader faculty audience in learning about structural racism and implicit bias can help institutions develop more faculty leaders in these areas.

To address these gaps, our team within the Department of Pediatrics at Boston Medical Center and the Boston Combined Residency Program developed Health Equity Rounds (HER): a longitudinal case-based curriculum designed to engage faculty and practitioners across training levels and disciplines together in discussions of how racism and implicit bias directly impact patient care. In each HER conference, we taught participants to apply evidence-based tools to identify how individual biases impact patient care, provided didactic instruction on the historical context or structural forces that shape these biases, and brainstormed ways to mitigate the effects of bias and structural racism in the particular case and also in our medical systems at large.

Structural racism and implicit bias are challenging and provocative topics to teach at all training levels, and if not thoughtfully presented, they can evoke negative reactions and create a defensive and counterproductive environment.^22^ Thus, we carefully selected conceptual frameworks to guide our curriculum development. We anchored our approach in a conceptual framework by Burgess, van Ryn, Dovidio, and Saha^23^ from the social psychology literature that provided tools to teach health care providers about racial bias and mitigate its impact. This framework highlighted the need to provide learners with motivation, information, emotional recognition and regulation, and space for skills building to reduce the impact of racial bias. We also incorporated a framework by Sukhera and Watling^24^ for integrating implicit bias training into health professions education. This framework emphasized the need for curricula to foster a supportive environment, provide foundational knowledge in the cognitive basis of bias, teach about the historical and present-day contexts of structural racism, deliver evidence on the effects of racism and bias on patient outcomes, and create opportunities for self-reflection, self-awareness, and empathy building.

HER is unique compared to other curricula in *MedEdPORTAL*^8–14^ in that we designed it for an interdisciplinary audience spanning training levels. Additionally, we utilized real cases from our own institutions as a foundation for discussion that involved providers, patients, and families sharing their experiences. Lastly, our curriculum is distinct in its focus on sharing and actively proposing solutions on both personal and institutional levels to confront issues raised in the cases presented.

## Methods

### Team

A small group of residents within our team first developed HER in response to a departmental call for new curricula that would meaningfully bring trainees and faculty together to engage in and discuss topics related to implicit bias and racism. Our trainee leadership was a key aspect of the curriculum's success, contributing not only to the dynamic and innovative environment fostered by the HER team but also to more rapid and widespread buy-in from audience members and the department as a whole. Our HER team was composed of residents from the PGY 1, PGY 2, and PGY 3 years, with at least one senior resident (PGY 2 or PGY 3) and one intern assigned as the leaders for each individual case conference. The senior resident leader focused on organization and mentorship, while the intern leader focused on content creation and delivery, with interns moving into senior leadership roles as they progressed in residency. As our resident leaders graduated, they moved into advisory roles during chief residency, fellowship, and junior faculty positions. Our advisory team also included faculty members, the pediatric residency program director, and the chair of the Department of Pediatrics at Boston University School of Medicine. These advisers provided feedback on case selection and presentation, assisted with evaluation, and connected our team to resources within and outside the institution. Finally, we had a supportive network of institutional leadership helping us advance and expand the conference to achieve broader culture change, including leaders from the Office of Graduate Medical Education, the hospital's vice president of mission, and the leaders of Schwartz Center Rounds at Boston Medical Center.

### Setting

We presented HER quarterly during a time reserved for a weekly departmental case conference to ensure a consistent pediatric faculty presence and reach learners at multiple stages of training. We opted for this large-group setting to create a shared fund of knowledge and build skill sets that could spur culture change within our own department. Finally, with each conference, we also extended an invitation to members of other disciplines (e.g., social work, nursing, psychology) and departments to allow for multidisciplinary input in our discussions.

### Conference Preparation and Presentation

In the following section, we describe the process of creating a HER case conference, which includes case selection, case presentation, presentation of relevant data, discussion of historical context, sharing of advocacy tools, and discussion moderation. In the appendices, we have included seven complete HER presentations, each with its own set of case-specific objectives (Appendices A-G), as well as selected handouts (Appendix H) for others to use at their own institutions. We have also included a Case Conference Creation Guide (Appendix I) that provides step-by-step instructions on how to create new HER presentations. Table 1 describes the case details, specific forms of bias discussed, historical context, reflection exercises, and resources provided.

**Table 1.** Summary of Cases

Case	Case Topic	Specific Biases Discussed	Historical/Present-Day Contexts Reviewed	Reflection Exercises	Resources Provided
1	Abdominal pain	Introduction to implicit bias	Heuristics, system 1 and system 2 thinking, the Implicit Association Test	Guided imagery, generating shared conference values using Poll Everywhere, perspective taking	Worksheet for reflection exercises
2	Sickle cell disease and fever	Stigma and bias associated with sickle cell disease	History of the false construction of race as a biological entity, the role of mistrust in sickle cell disease	Guided imagery, generating shared conference values using Poll Everywhere, reflection writing, perspective taking	Worksheet for reflection exercises
3	Discipline	Bias in approach to family discipline practices	AAP guidelines on discipline, disparities in school discipline practices, outcomes associated with physical discipline, media portrayals of discipline practices	Guided imagery, perspective taking, individual reflection	Community resources for parenting and discipline support
4	Contraceptive counseling	Bias in contraceptive counseling	AAP and SAHM guidelines on contraceptive counseling, history of forced sterilization and coercion in reproductive health counseling	Guided imagery, perspective taking, individual reflection	Contraceptive counseling algorithm, worksheet for reflection exercises
5	Williams syndrome	Bias in medical textbooks and training	Disparities in physical diagnosis education, disparities in genetics databases, medical education's role in falsely teaching race as a biological construct	Storytelling, perspective taking	Handout with action items for addressing bias in medical education
6	Trauma exposure	Colorism	Historical trauma faced by indigenous populations in El Salvador	Guided imagery, perspective taking	Handout on Diversity-Informed Mental Health Tenets
7	Behavioral counseling	Bias related to Department of Children and Families involvement	History of child protection, concept of the New Jane Crow	Guided imagery, perspective taking, incorporation of quality improvement tools including creation of a fishbone diagram	Handout on Talking Race Toolkit, fishbone diagram for quality improvement exercise

Abbreviations: AAP, American Academy of Pediatrics; SAHM, Society for Adolescent Health and Medicine.

#### Case selection and preparation

First, we solicited cases from residents and faculty within the Department of Pediatrics via an email asking for cases (1) where implicit bias and/or racism played a role in patient outcomes or experiences and (2) that were appropriate for further exploration using implicit bias and structural racism as a lens. By utilizing real patient vignettes, we demonstrated the importance of implicit bias and structural racism as they affected our own community, leveraging adult learning principles of making the content relevant and drawing on previous experiences. We preferentially selected cases with less medical complexity or intentionally removed nuanced medical details to prioritize discussion of the nonclinical aspects and promote consideration of implicit bias and racism. For many of the cases, we contacted experts in the topic areas to help inform our literature review, prioritize discussion points, and develop advocacy materials. We tried to capitalize on expertise from nonphysician disciplines and backgrounds to maximize the interdisciplinary framework of the conference. For example, a family planning counselor shared her expertise with the audience in case 4 (Appendix D), a nurse practitioner presented a case in which she was involved in case 5 (Appendix E), and a social worker presented a case and relevant tools in case 6 (Appendix F). When possible, we invited patients and families to share their experiences and participate as guest speakers.

#### HER presentation

We used PowerPoint as the platform for our conference. We began each HER by presenting ground rules to ensure that the space for discussion remained respectful and productive. We also gave each participant a glossary of keywords and concepts to establish a common vocabulary (Appendix J). We then introduced the overarching goals and objectives of the HER series, as well as the specific educational objectives for the current conference.

We devoted the next portion of the conference to presenting the medical case. During the case presentation, we explicitly asked audience members to suspend disbelief and accept that the medical details given were sufficient for understanding the objectives of the conference. Throughout the presentation of the medical case, we interjected opportunities for the audience to practice evidence-based skills to mitigate the effects of implicit bias. For example, we used guided imagery exercises to practice individuation—focusing on unique qualities rather than learned group stereotypes of individuals—and perspective taking—considering a situation from the perspective of the patient and family rather than the provider.^23^ We used different modalities to operationalize these exercises, including written reflection, share out loud, and think-pair-share.

After presenting the case, we transitioned to sharing evidence from the literature regarding the impact of implicit bias and racism on patient outcomes as a way to enhance the audience's internal motivation to combat bias.^23,24^ We then presented the relevant historical context that led to and informed the implicit bias and structural racism present in the case. For example, in HER 4 (Appendix D), which focused on reproductive justice, we provided a time line of coercive policies in sterilization and contraception targeting populations of color that took place from 1907 to 2010, demonstrating that the issue had both important historical background and contemporary impact.

Finally, in the last portion of each presentation, we provided advocacy tools to enhance providers’ confidence in their abilities to decrease the impact of implicit bias and structural racism at individual, community, and institutional levels. These included clinical decision tools, strategies for communicating effectively with colleagues, and institutional resources and initiatives related to mitigating implicit bias and structural racism. We also provided the audience with time for reflection and discussion of ideas and solutions. We carefully selected moderators, who initially were faculty but in later iterations were residents, to lead discussions. Our moderators were able to redirect conversations that deviated toward clinical aspects of the case back to the focus on implicit bias and race, felt comfortable with the subject matter, and were capable of fostering fruitful discussions with sensitivity to time limitations.

### Evaluation

We developed our evaluation in an iterative fashion, progressing from assessing the feasibility and acceptability of the curriculum to exploring different aspects of participants’ experiences with HER and how the presented material impacted participants’ lives and practices outside of HER. The Institutional Review Board for the Boston University School of Medicine reviewed and granted exemption for our evaluation work.

We tracked HER conference attendance by in-room head count or by counts from the faculty sign-in sheet plus the number of residents and medical students scheduled for conference attendance. We additionally obtained counts of continuing medical education (CME) credit requests by faculty for both HER and non-HER case conferences. We compared the mean number of CME credit requests submitted by faculty for HER to non-HER case conferences using a two-tailed *t* test with unequal variances. We considered *p* < .05 to be statistically significant. We distributed anonymous paper surveys as participants entered the conference. Alternatively, participants could complete an electronic version of the survey distributed after the conference. Surveys asked participants to identify their role (attending physician, resident, medical student, etc.) and departmental affiliation; they also included questions asking participants to state whether they thought that implicit bias impacted their own clinical practice, rate the educational value of the conference, and assess whether the conference met stated objectives or contained any inappropriate or offensive content. The survey additionally included free-text fields asking for comments, constructive criticism, or elaboration on particular questions. We include an example survey in Appendix K. We analyzed quantitative survey data using descriptive statistics. We qualitatively reviewed responses to free-text survey questions and grouped similar comments into categories.

## Results

We successfully implemented seven case-based HER conferences from June 2016 to June 2018. Table 2 describes attendance, survey response rates, and selected survey responses from the conferences. Of note, because we modified the conference surveys over time, not all survey questions were included for each conference. Specifically, in our first year of implementation, we asked focused questions about whether we had met our educational objectives that we did not ask in the subsequent year.

**Table 2.** Attendance Estimates and Postconference Survey Responses

	Conference						
Survey Measure	1: No. (%)	2: No. (%)	3: No. (%)	4: No. (%)	5: No. (%)	6: No. (%)	7: No. (%)
Estimated attendance^a^	77 (100)	71 (100)	51 (100)	51 (100)	73 (100)	66 (100)	74 (100)
Survey response	48 (62)	25 (35)	28 (55)	35 (69)	48 (66)	49 (74)	53 (72)
Attendees:							
Attending physician	15/47 (32)	4/25 (16)	15/28 (54)	18/32 (56)	14/48 (29)	20/46 (43)	10/53 (19)
Fellow	3/47 (6)	1/25 (4)	0/28 (0)	1/32 (3)	4/48 (8)	1/46 (2)	1/53 (2)
Resident	18/47 (38)	6/25 (24)	9/28 (32)	8/32 (25)	10/48 (21)	17/46 (37)	21/53 (40)
Medical student^b^	10/47 (21)	13/25 (52)	3/28 (11)	1/32 (3)	8/48 (17)	4/46 (9)	12/53 (23)
Other	1/47 (2)	1/25 (4)	1/28 (4)	4/32 (13)	12/48 (25)	4/46 (9)	9/53 (17)
Attendee department:							
Pediatrics	31/47 (66)	12/25 (48)	22/27 (81)	28/31 (90)	38/48 (79)	36/45 (80)	43/51 (84)
Family medicine	6/47 (13)	0/25 (0)	2/27 (7)	0/31 (0)	2/48 (4)	5/45 (11)	2/51 (4)
Other	10/47 (21)	13/25 (52)	3/27 (11)	3/31 (10)	8/48 (17)	4/45 (9)	8/51 (16)
Respondents answering “yes” to whether the conference met stated objectives:							
Introduce HER	46/47 (98)	24/25 (96)	26/27 (96)	29/31 (94)	NA	NA	NA
Introduce implicit bias and its application to medicine	46/47 (98)	24/25 (96)	26/27 (96)	27/31 (87)	NA	NA	NA
Promote reflection on personal implicit bias^c^	43/47 (91)	21/24 (88)	25/26 (96)	NA	NA	NA	NA
Identify resources, opportunities, or tools to address implicit bias^d^	30/46 (65)	17/25 (68)	27/27 (100)	NA	NA	NA	NA
Respondents rating conference educational value as “good” or “excellent”	47/47 (100)	24/25 (96)	21/26 (81)	31/34 (91)	47/48 (98)	NA	NA
Respondents indicating:							
Interest in attending another HER	45/46 (98)	22/23 (96)	23/26 (88)	32/33 (97)	NA	NA	NA
Elements of the content were inappropriate or offensive	2/46 (4)	2/22 (9)	1/27 (4)	1/34 (3)	0/47 (0)	2/48 (4)	0/48 (0)
Attending HER will impact their clinical practice	33/44 (75)	20/22 (91)	20/26 (77)	23/29 (79)	47/47 (100)	NA	NA

Abbreviation: HER, Health Equity Rounds; NA, not asked.

a Attendance for HER 1-4 was estimated based on faculty continuing medical education credit sign-in sheet and survey responses plus residents and medical students scheduled for mandatory attendance. Attendance for HER 5-7 was based on in-room head count.

b In HER 2, medical students included applicants to the pediatric residency program.

c The topical focus of HER 3 was spanking. Attendees were asked whether the conference met the objective to “identify personal biases associated with spanking.”

d In HER 3, attendees were asked whether the conference met the objective to “provide educational resources and opportunities for advocacy surrounding discipline.”

We had a mean attendance of 66 participants per session. The survey response rate ranged from 35% to 74%. Physician survey respondents spanned all training levels from medical student to attending physician. The percentage of nonphysician survey respondents varied by conference, ranging from 2% to 25%, and included social workers, nurses, administrators, researchers, and other clinical staff (e.g., dieticians, case managers). We achieved a consistent faculty presence at HER similar to the presence at more traditional case conferences. The mean number of requests submitted by pediatric faculty for CME credit per session at the seven HER conferences from June 2016 through June 2018 was 33.0 (*SD* = 10.6), compared to 28.1 for the 90 non-HER conferences (*SD* = 5.0, *p* = .28).

More than 80% of survey respondents rated the educational value of each session as good or excellent (range: 81%-98%) and indicated that HER promoted personal reflection on implicit bias (range: 88%-96%). Seventy-five percent or more indicated that HER would impact their future clinical practice (range: 75%-100%). During the first two sessions, 65% and 68% of survey respondents agreed that HER identified resources, tools, or opportunities to address implicit bias. However, after the third session, 100% of respondents agreed that the conference identified these resources, likely reflecting an intentional shift in conference content in response to prior feedback requesting more time for solutions-oriented discussion. A minority of survey respondents (range: 0%-9%) indicated that aspects of the conference were inappropriate or offensive, although accompanying free-text comments often indicated that the respondent was highlighting the inappropriate or offensive nature of racism rather than conference presentation.

Following are representative quotes from free-text responses on postconference surveys:

• Reactions to the nonclinical focus of a case-based conference:
  ○ “It was very uncomfortable hearing about the role of the medical profession in racism, but it was very important to highlight.”
  ○ “Appreciated active redirection toward uncomfortable topics of race.”
  ○ “I appreciated the focus on race versus too much focus on the clinical case which is often the easier aspects of the case to focus on.”
  ○ The participant noted that he/she was feeling “enthusiastic” during group discussion due to “hearing perspectives from diverse attendees especially those from different cultural backgrounds.”
  ○ “Loved it! . . . complex and emotional topic.”
• Reflections on the impact of HER on personal practice:
  ○ “[The conference] will continue to trigger me to think about how I engage with patients who trigger my own biases and to engage in discussing with residency regarding how to improve.”
  ○ “[I] plan to stop and think of my ‘illness script’ before walking into a room, asking myself what biases are present within that will hopefully help me address them.”
  ○ “[I will] increase levels of consciousness with my interactions—though hopefully not too temporary.”
  ○ “[I will practice] more individual awareness and reflection; inspired to work on making this [implicit bias] something I think about consistently during patient interactions.”
• Reflections on implicit bias at the departmental or institutional level:
  ○ “We also need to do this with intern selection.”
  ○ “[We need] more conversations about to approach this from institutional/residency level.”
  ○ “[We need to] foster more discussion [about implicit bias] with depth.”
• Negative reactions:
  ○ “I am not racist and I refused to become one with activities like this . . . To be completely honest I did not learn anything new.”
  ○ When asked “Were you offended at any point during Health Equity Rounds?” a participant answered: “No . . . but at times I felt I was being led to feel a certain way.”
  ○ “The cases in the last two Health Equity Rounds in discipline and contraception required a leap to link to the discussion of [spanking at home and] corporal punishment in schools and [contraceptive counseling for teenage girls and the history of] forced sterilization in my opinion.”
• Suggestions for improvement:
  ○ “More time spent on brainstorming solutions as people clearly had more ideas.”
  ○ “This would have been a great case to use role play to model actual term/approaches to having these discussion[s] [with patients and families].”
  ○ “Need more time for the end discussion!!!”
  ○ “More discussion on structural racism.”
  ○ “More time to focus on solutions.”
  ○ “Try and include someone who was directly involved in the case so they can share personal challenges/reflections.”
  ○ “More active guidance during self-reflection.”

Most participants conveyed enthusiasm for HER; however, several participants also reflected on the need to extend discussions of implicit bias to other areas, such as intern selection. In general, constructive feedback focused on increasing time for discussion and brainstorming solutions. Several survey respondents voiced negative reactions, including impressions that discussing implicit bias and structural racism could cause racism or that connecting structural racism and current challenges in clinical care required too great a conceptual leap.

## Discussion

HER provided a unique forum for addressing structural racism and implicit bias across the educational continuum. From June 2016 to June 2018, we successfully delivered seven HER conferences to an interdisciplinary audience at Boston Medical Center. Our results show that we engaged faculty in this curriculum and provided a unique space in which clinicians spanning multiple training levels, disciplines, and departments discussed the issues of implicit bias and racism as colleagues. Survey respondents stated that HER provided them with the awareness, motivation, and tools necessary to reflect on their implicit biases and alter their clinical practices.

We iteratively responded to feedback collected in postconference surveys, making changes with each subsequent HER. For instance, in response to audience feedback requesting more discussion about solutions to address racism and implicit bias, we created a robust solutions-focused section as the final portion of each HER and provided participants with an accompanying handout of relevant resources. We also became more intentional about explicitly naming the evidence-based exercises we employed to mitigate implicit bias during the conference so that participants could more adeptly recognize and then use them in their practices. Additionally, for some HER conferences, we offered opportunities for small-group debriefs following the larger conference to provide a forum for more intimate reflection. As HER evolved, we were mindful to target our efforts at addressing implicit racial bias, as opposed to other forms of implicit bias, such as gender, religion, weight, or sexuality, to maintain a focus on the impacts of structural racism. These are charged and difficult subjects to address; however, we have noticed increased participation in discussions surrounding racism as our audience has become more experienced with the HER curriculum.

The evaluation of HER remains our greatest area of ongoing evolution, development, and challenge. Our goal with HER is for providers to be able to recognize and mitigate the effects of implicit bias and racism in their interactions with patients and colleagues, and for this to subsequently effect broader culture change. However, measuring a single curriculum's impact on changing implicit bias, attitudes on race, and culture change is difficult, given that many factors contribute to such changes. These factors include exposure to formal and informal curricula on implicit bias and racism, workplace diversity, and lived experiences.^25^ Therefore, we are attempting to take a more comprehensive approach to understanding broad exposure to these topics by experimenting with survey questions regarding prior exposure to racial justice and implicit bias awareness trainings and comfort with discussing racism and implicit bias in different settings. In addition to conference-specific surveys, we are also in the process of conducting focus groups to further explore participants’ reactions to HER—positive, negative, and ambivalent.

A deliberate, coordinated effort to address these issues by multiple means at our institution and beyond is necessary to effect the greatest change. To that end, we have disseminated our curriculum at Boston Children's Hospital, Children's National Medical Center, Duke Children's Hospital, and Massachusetts General Hospital for Children. We have also collaborated with other groups within our institution on complementary efforts, including a racial justice seminar for pediatric interns in the Boston Combined Residency Program, a program for residents to engage in storytelling around experiences with racism and implicit bias, and a workshop on microaggressions for residency and fellowship program directors through the Office of Graduate Medical Education. The interdisciplinary experts and participants involved in HER thus far have been a critical component of the curriculum's success, yet we recognize that expanding our approach to include more interdisciplinary collaboration at the leadership level is an important next step. One way we are doing this is by collaborating with our institution's branch of Schwartz Center Rounds—a program that fosters interdisciplinary dialogue in health care organizations.

We appreciate that there are limitations to the generalizability of HER. We implemented HER at Boston Medical Center, the largest safety-net hospital in New England, with an explicit mission to provide equitable care to all and a self-selected group of faculty, staff, and students who may be particularly primed and interested in engaging in discussions on racism and implicit bias. As such, we had early institutional support and buy-in from leadership. At institutions where there may be less widespread acceptability of this curriculum, we recommend initially implementing HER in a smaller pilot forum before expanding it to a wider audience.

We anticipate that HER will continue to develop as an intergenerational and interdisciplinary means of teaching about the role of implicit bias and structural racism in medicine. We look forward to engaging with institutions interested in adapting this curriculum and partnering with them to create new content, brainstorm new methods to measure program effectiveness, and evaluate higher-level outcomes. Ultimately, we hope that HER fosters institutional culture change in such a way that combating implicit bias and structural racism is seen as a necessary component of advancing health equity.

## Appendices

A. HER 1.pptx
B. HER 2.pptx
C. HER 3.pptx
D. HER 4.pptx
E. HER 5.pptx
F. HER 6.pptx
G. HER 7.pptx
H. Selected HER Handouts.docx
I. Case Conference Creation Guide.docx
J. Glossary.docx
K. Evaluation.docx All appendices are peer reviewed as integral parts of the Original Publication.
