# Supplementary material for: Health Equity Rounds: An Interdisciplinary Case Conference to Address Implicit Bias and Structural Racism for Faculty and Trainees
Source: MedEdPORTAL. 2019 Nov 22;15:10858. doi: 10.15766/mep_2374-8265.10858 (PMC7050660; doi:10.15766/mep_2374-8265.10858)
Supplement: Supplementary file 1 — A. HER 1.pptx B. HER 2.pptx C. HER 3.pptx D. HER 4.pptx E. HER 5.pptx F. HER 6.pptx G. HER 7.pptx H. Selected HER Handouts.docx I. Case Conference Creation Guide.docx J. Glossary.docx K. Evaluation.docx [file mep-15-10858-s001.zip › A. HER 1.pptx]

## Slide 1
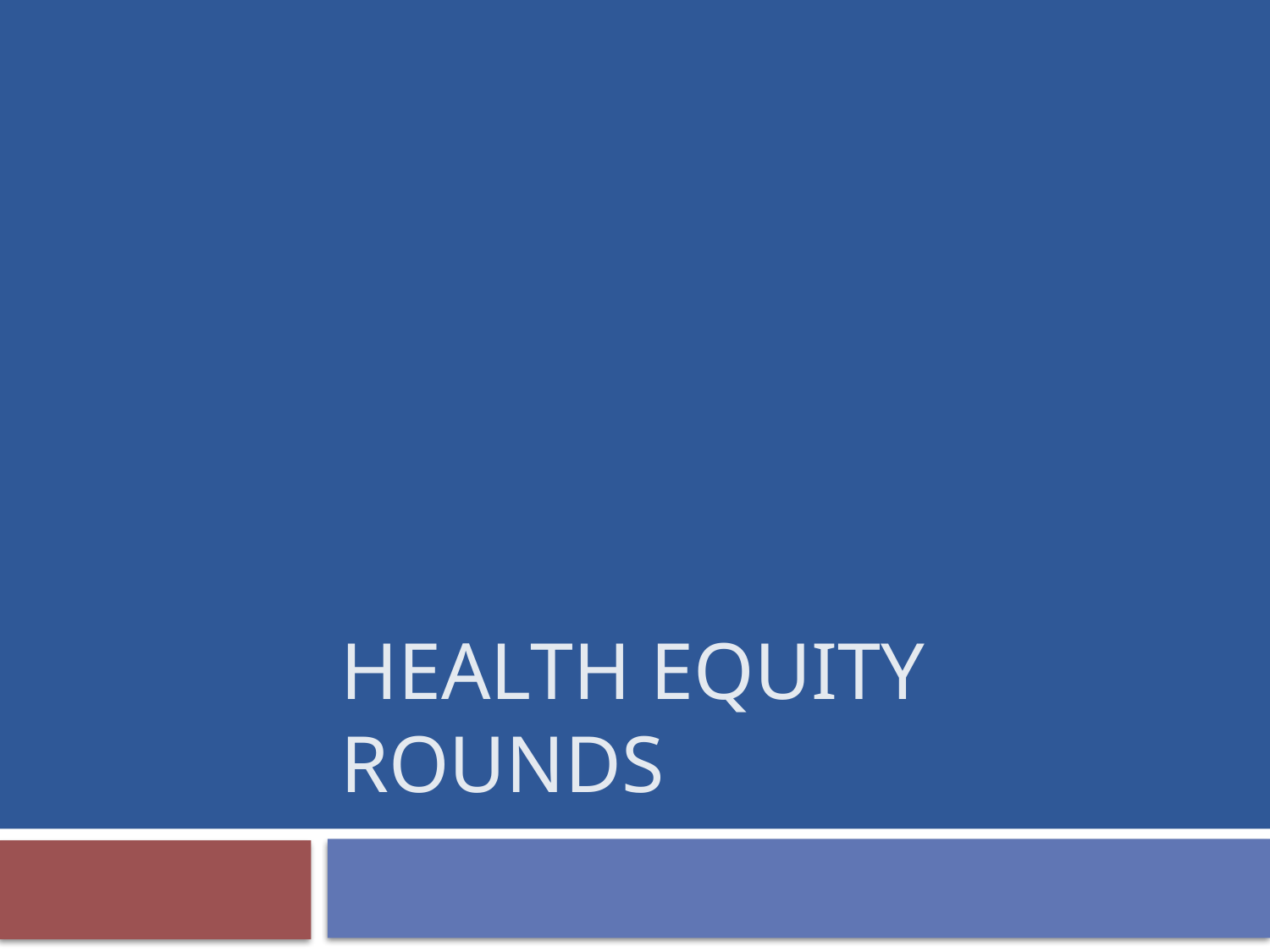

# Health Equity Rounds

## Slide 2
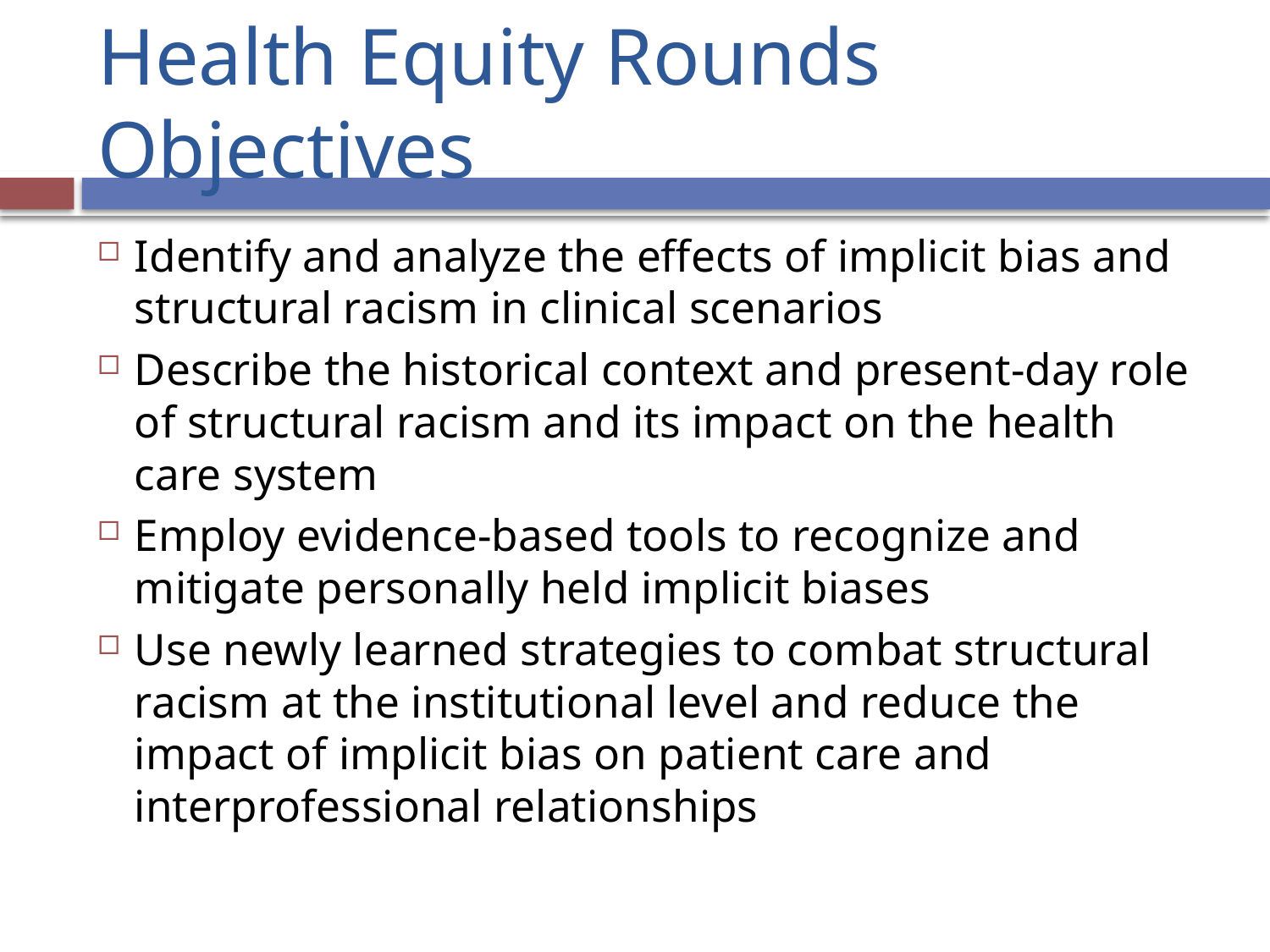

# Health Equity Rounds Objectives
Identify and analyze the effects of implicit bias and structural racism in clinical scenarios
Describe the historical context and present-day role of structural racism and its impact on the health care system
Employ evidence-based tools to recognize and mitigate personally held implicit biases
Use newly learned strategies to combat structural racism at the institutional level and reduce the impact of implicit bias on patient care and interprofessional relationships

## Slide 3
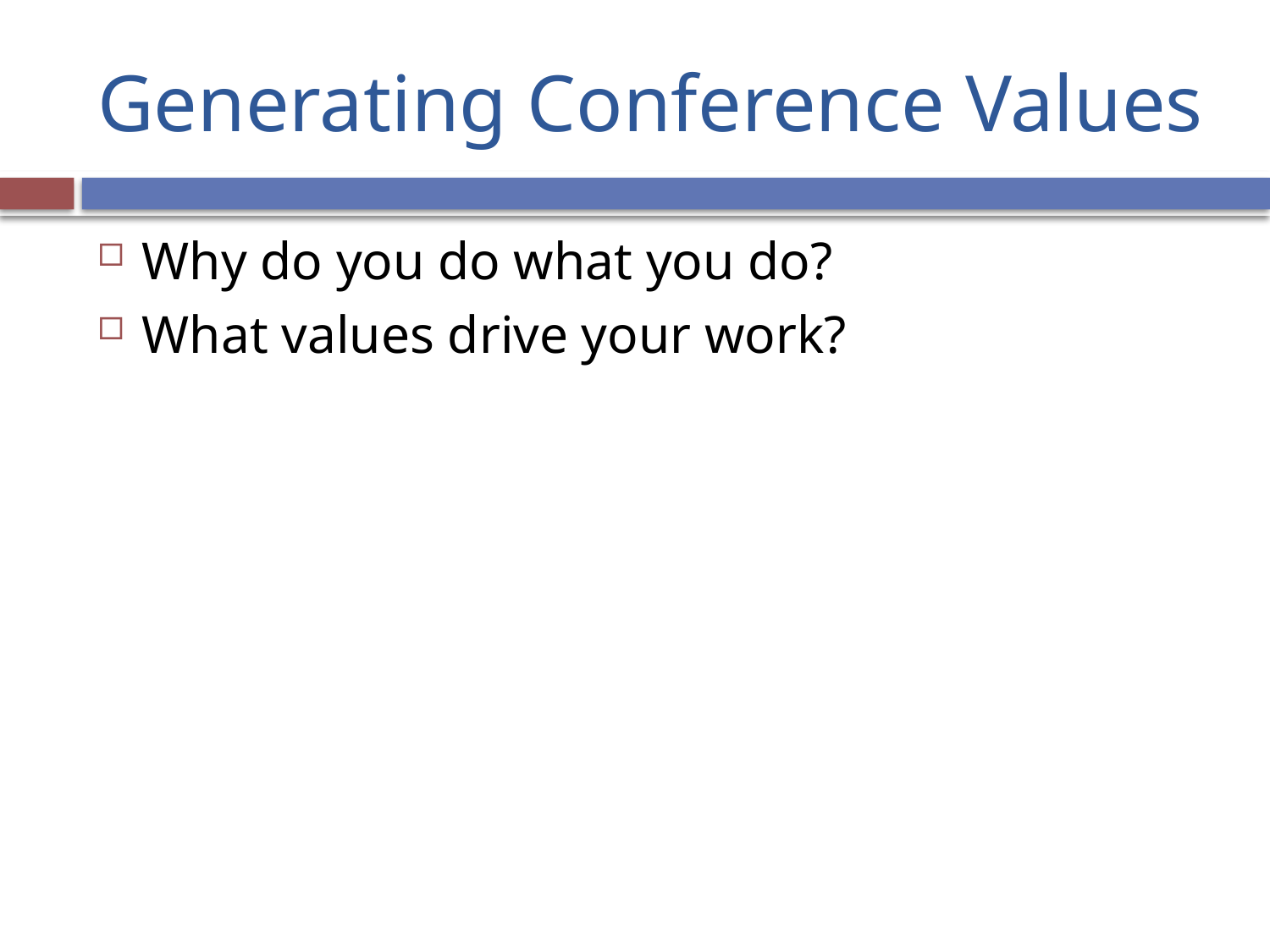

# Generating Conference Values
Why do you do what you do?
What values drive your work?

## Slide 4
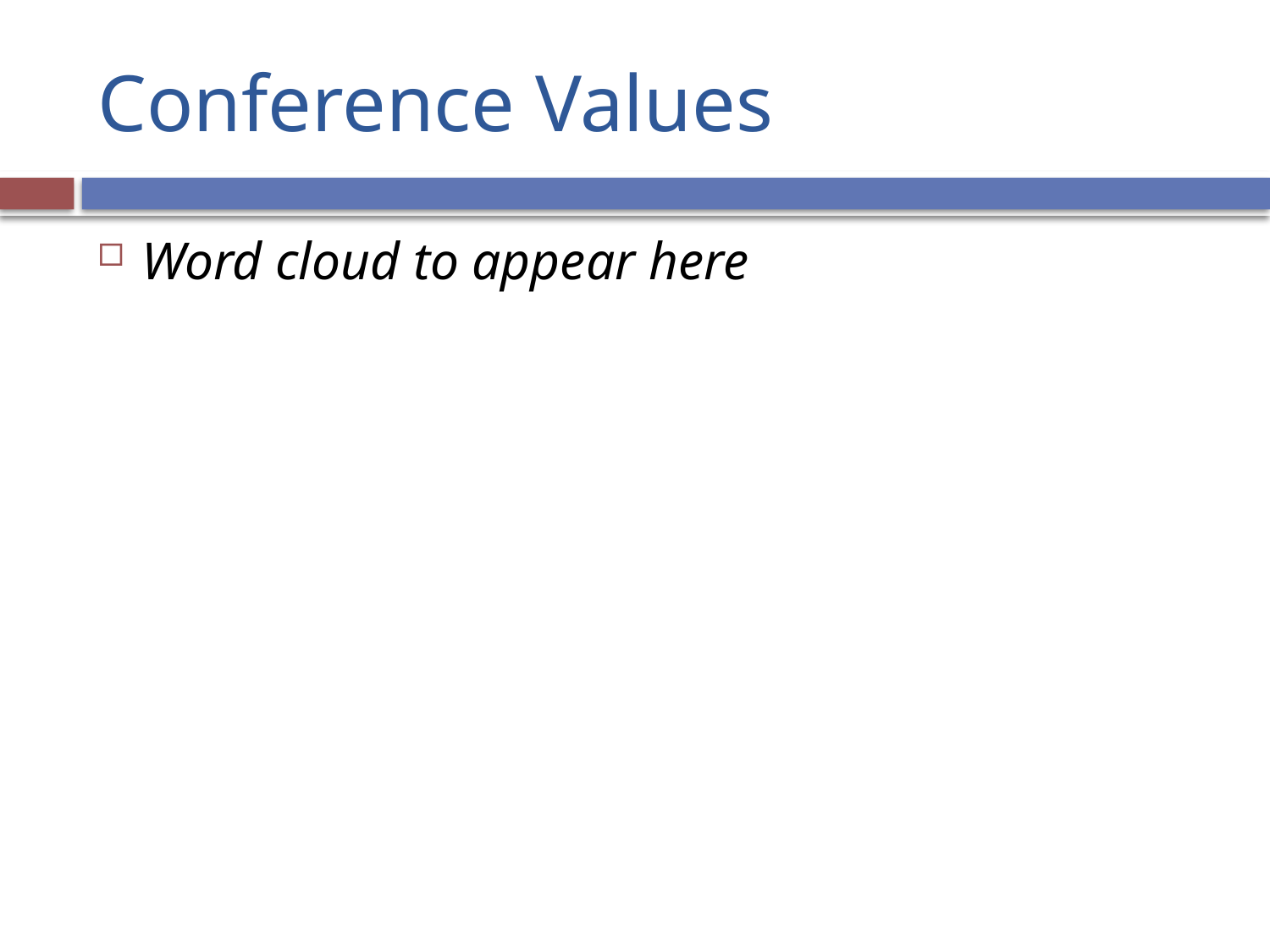

# Conference Values
Word cloud to appear here

## Slide 5
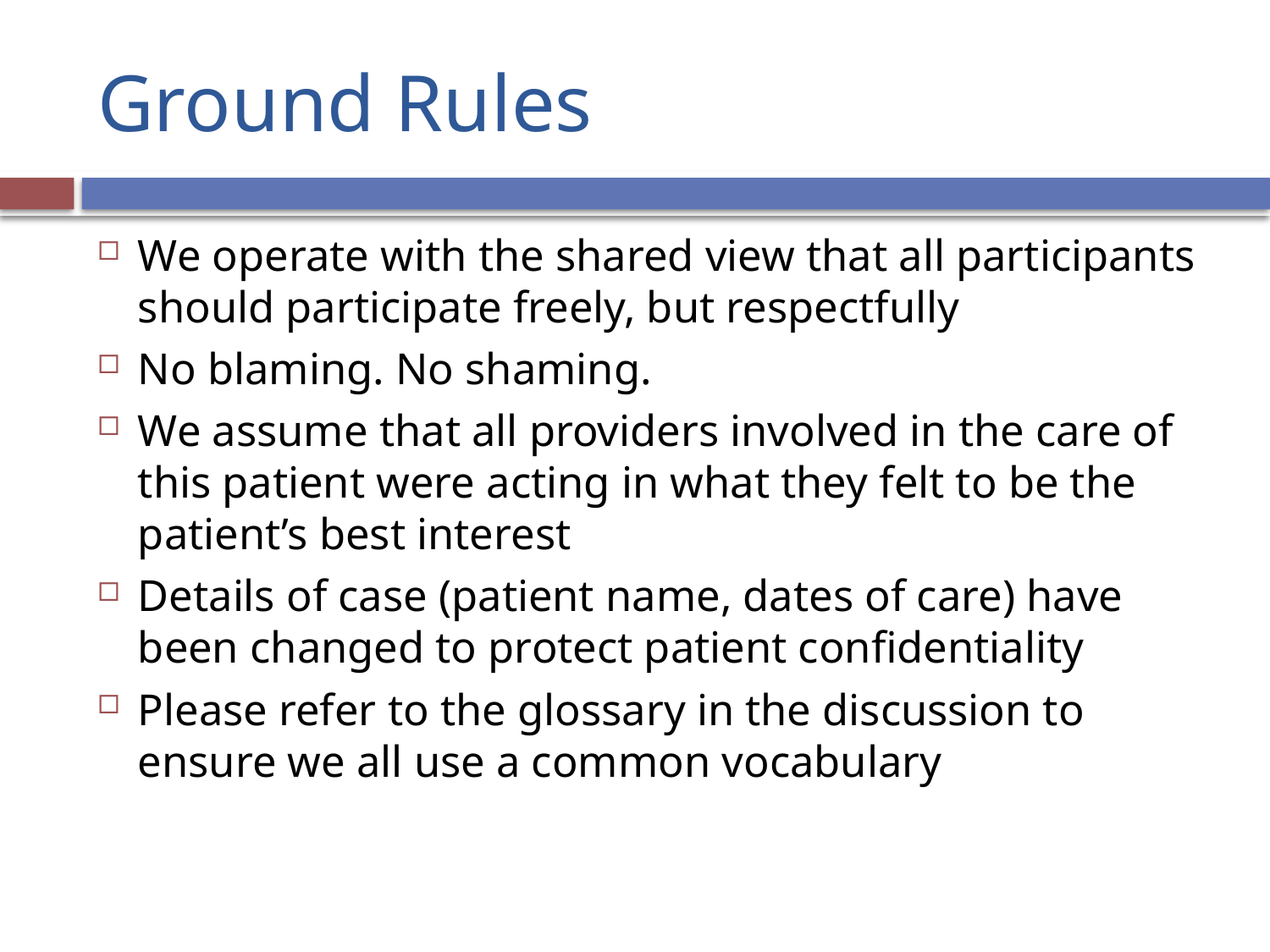

# Ground Rules
We operate with the shared view that all participants should participate freely, but respectfully
No blaming. No shaming.
We assume that all providers involved in the care of this patient were acting in what they felt to be the patient’s best interest
Details of case (patient name, dates of care) have been changed to protect patient confidentiality
Please refer to the glossary in the discussion to ensure we all use a common vocabulary

## Slide 6
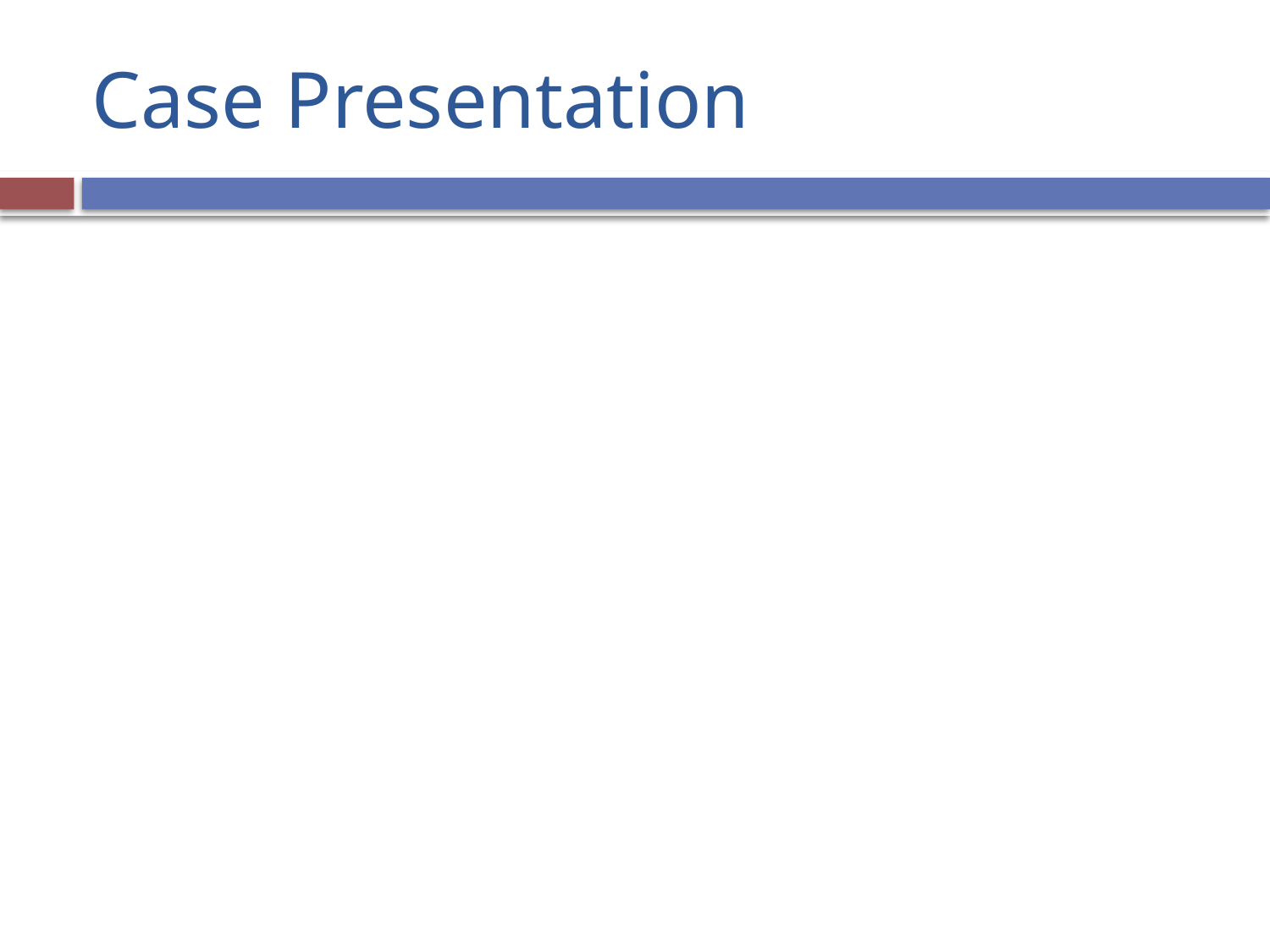

# Case Presentation

## Slide 7
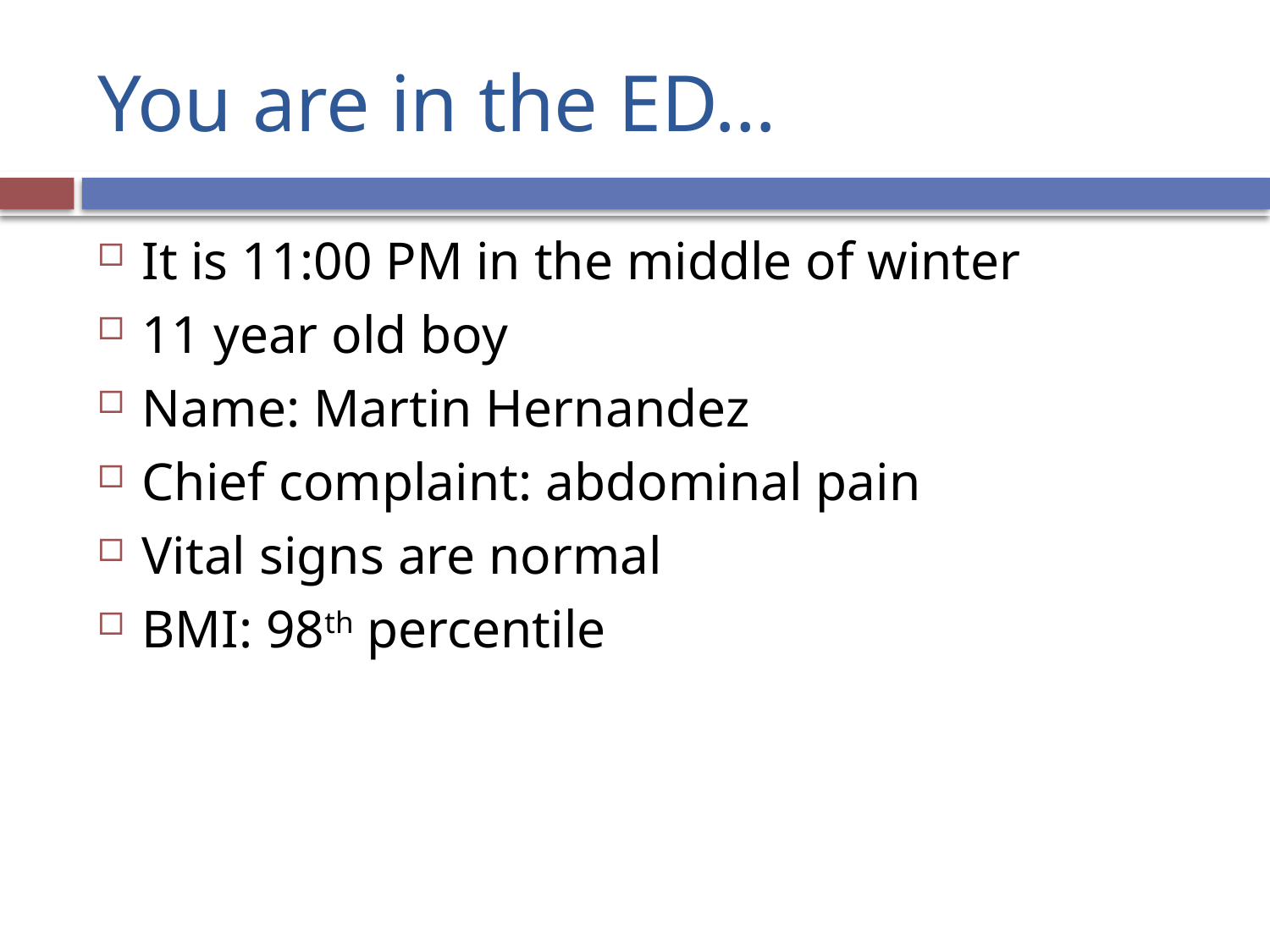

# You are in the ED…
It is 11:00 PM in the middle of winter
11 year old boy
Name: Martin Hernandez
Chief complaint: abdominal pain
Vital signs are normal
BMI: 98th percentile

## Slide 8
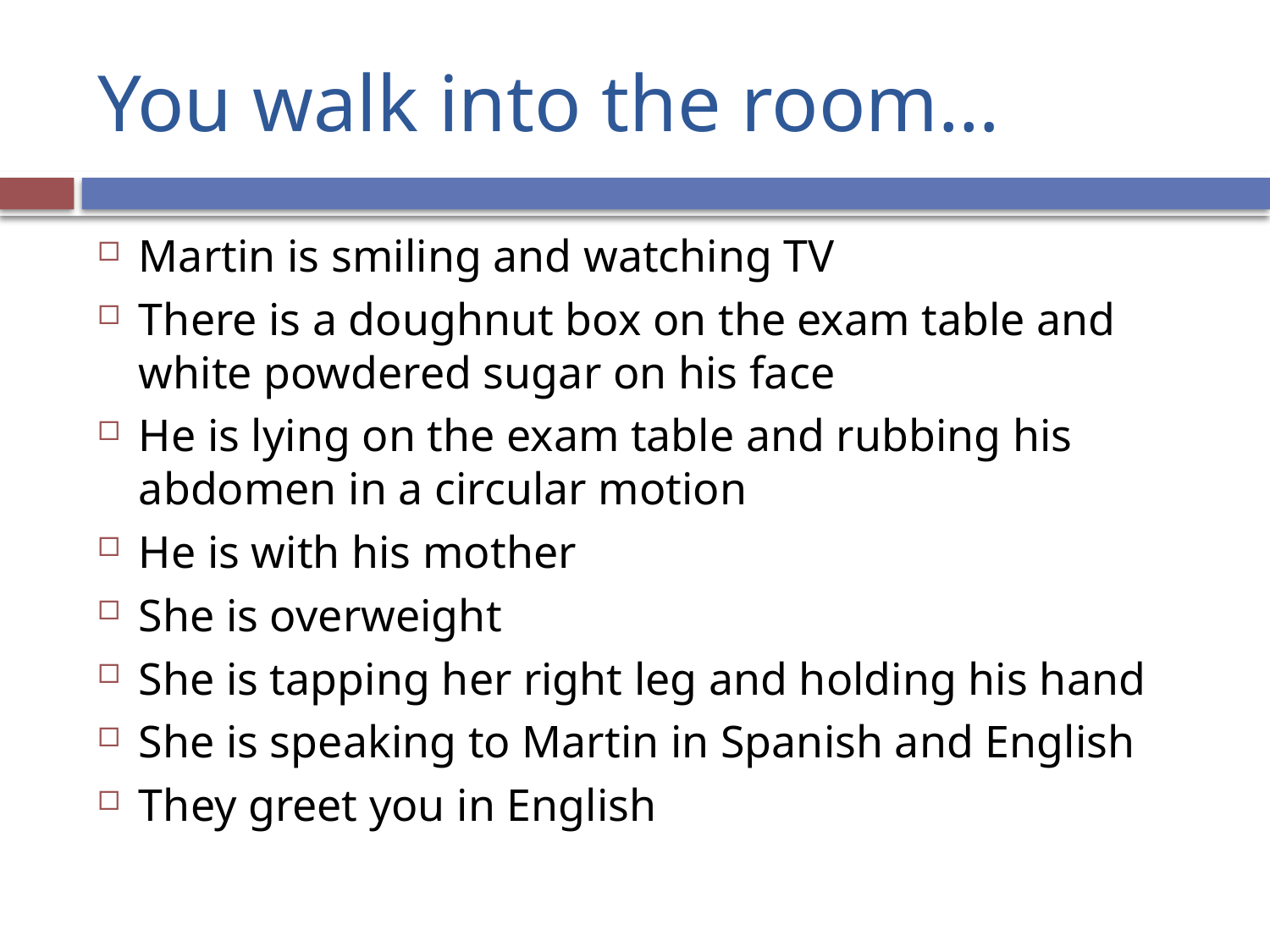

# You walk into the room…
Martin is smiling and watching TV
There is a doughnut box on the exam table and white powdered sugar on his face
He is lying on the exam table and rubbing his abdomen in a circular motion
He is with his mother
She is overweight
She is tapping her right leg and holding his hand
She is speaking to Martin in Spanish and English
They greet you in English

## Slide 9
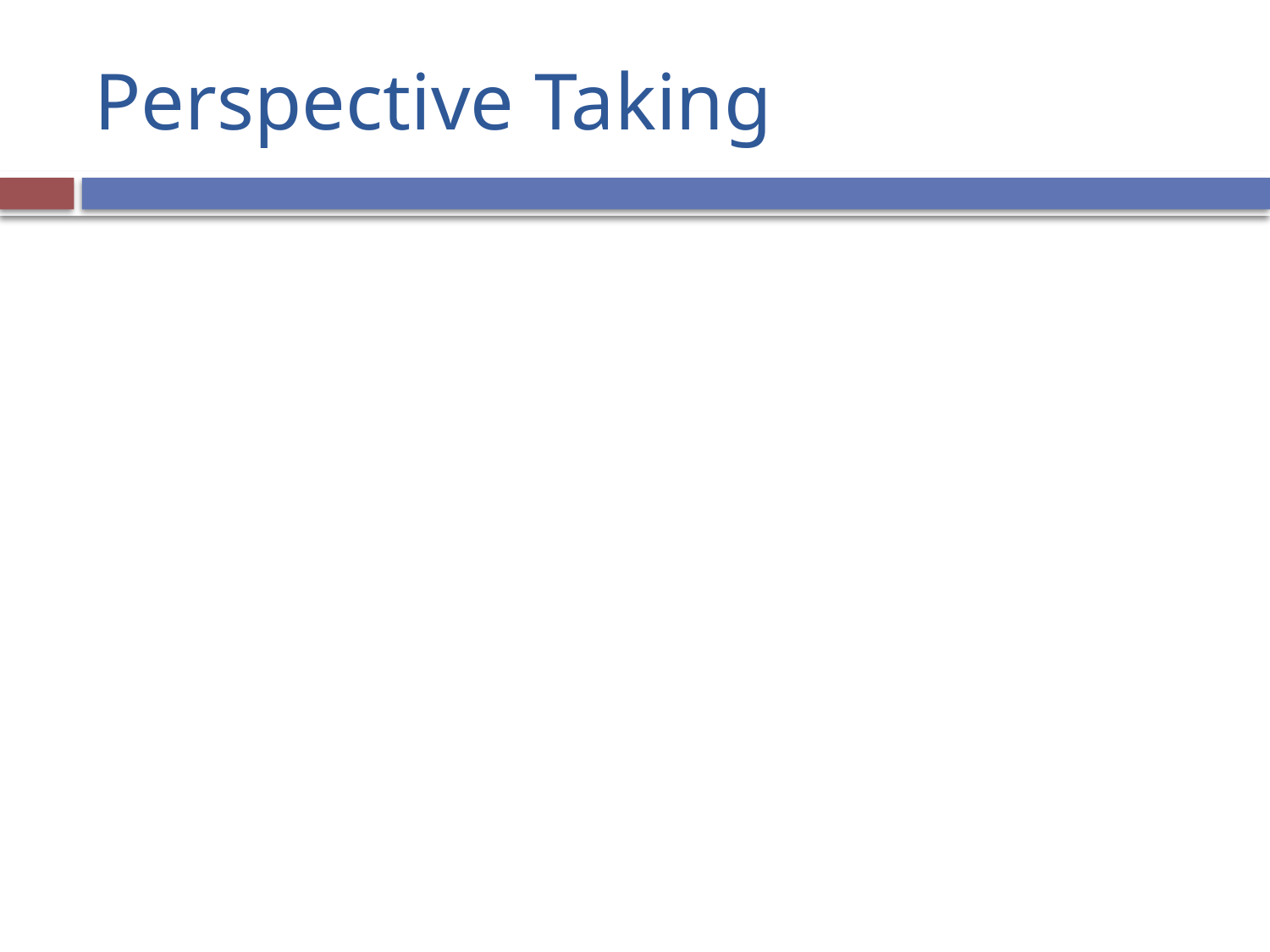

# Perspective Taking

## Slide 10
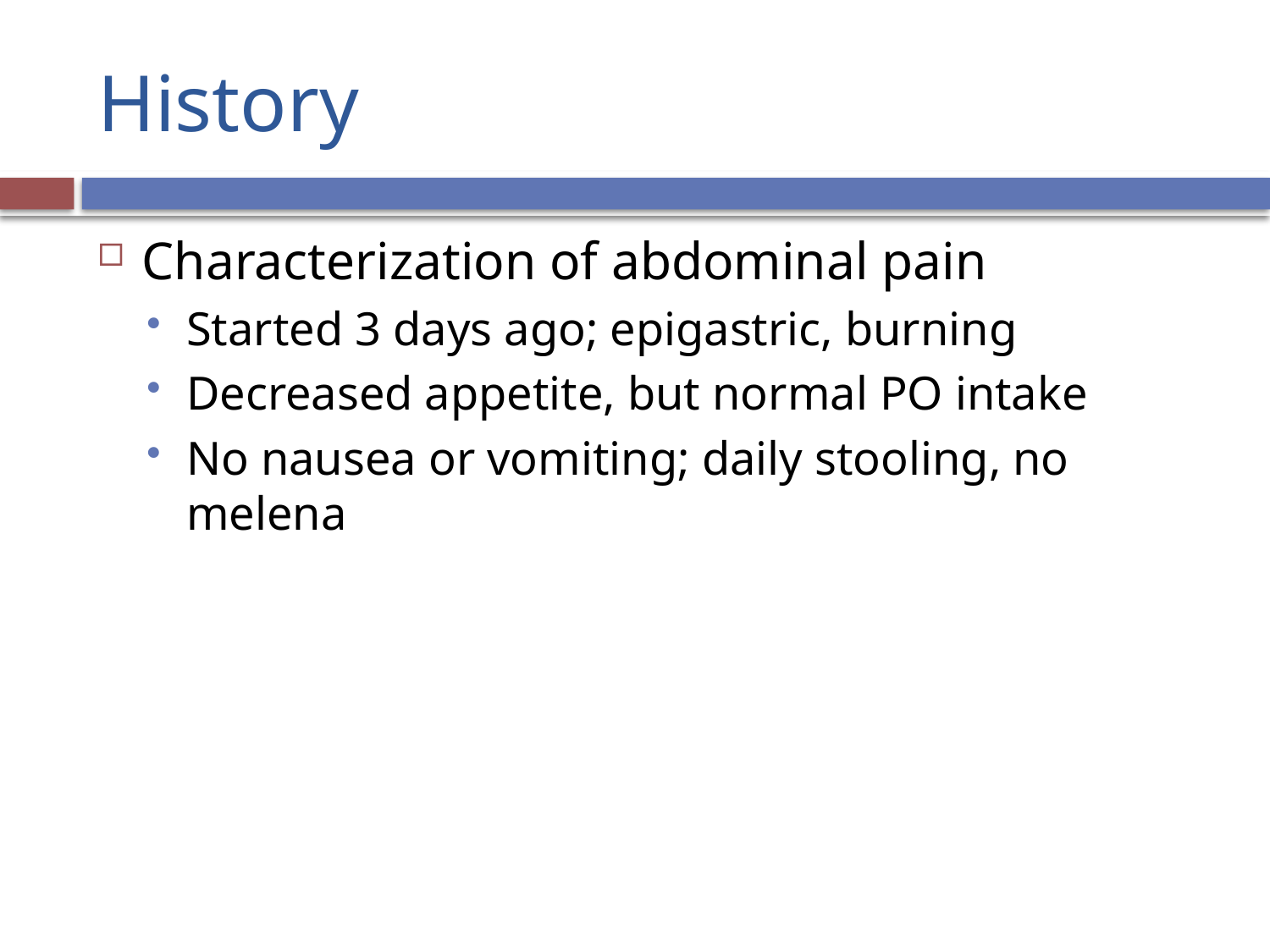

# History
Characterization of abdominal pain
Started 3 days ago; epigastric, burning
Decreased appetite, but normal PO intake
No nausea or vomiting; daily stooling, no melena

## Slide 11
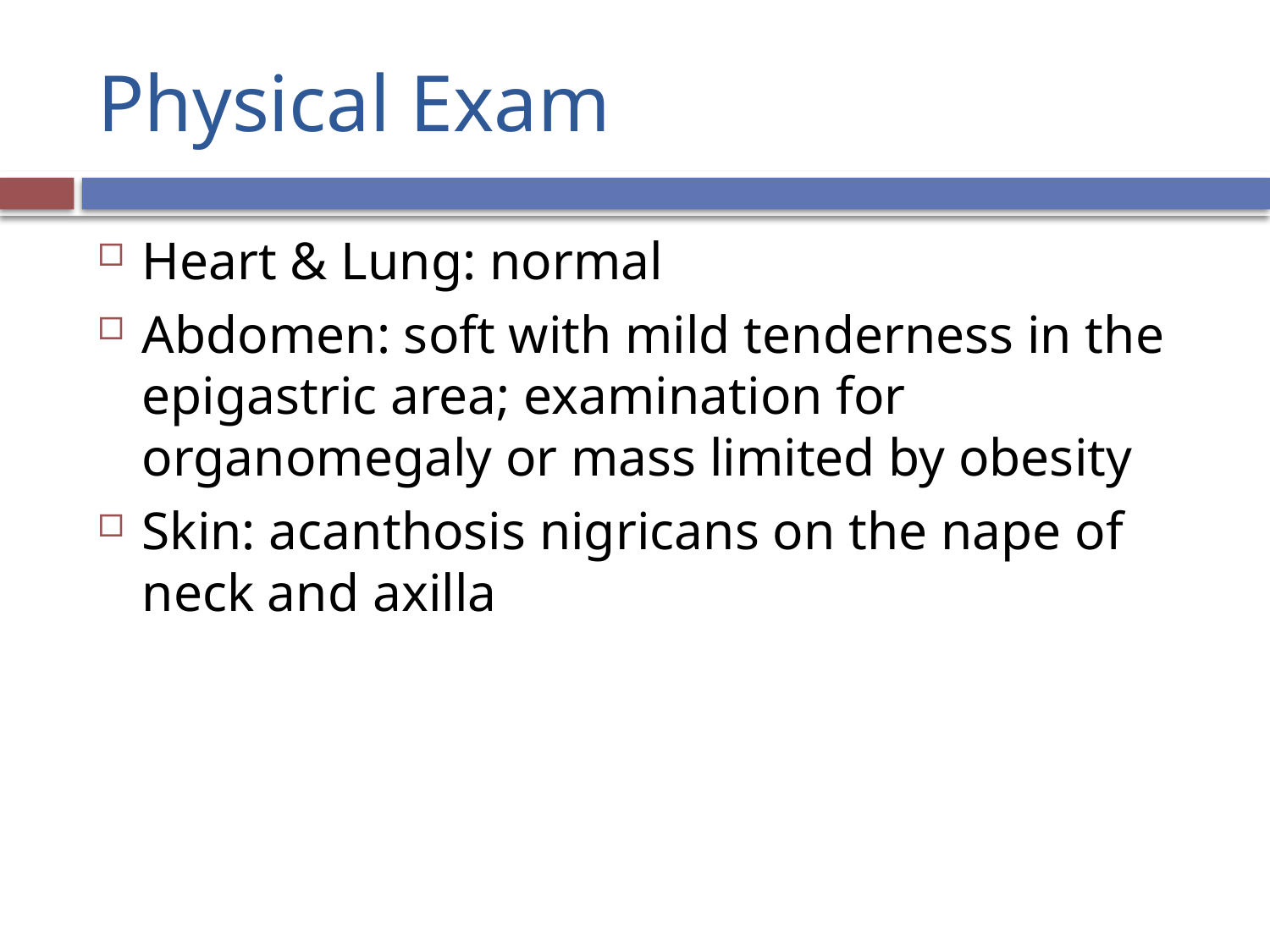

# Physical Exam
Heart & Lung: normal
Abdomen: soft with mild tenderness in the epigastric area; examination for organomegaly or mass limited by obesity
Skin: acanthosis nigricans on the nape of neck and axilla

## Slide 12
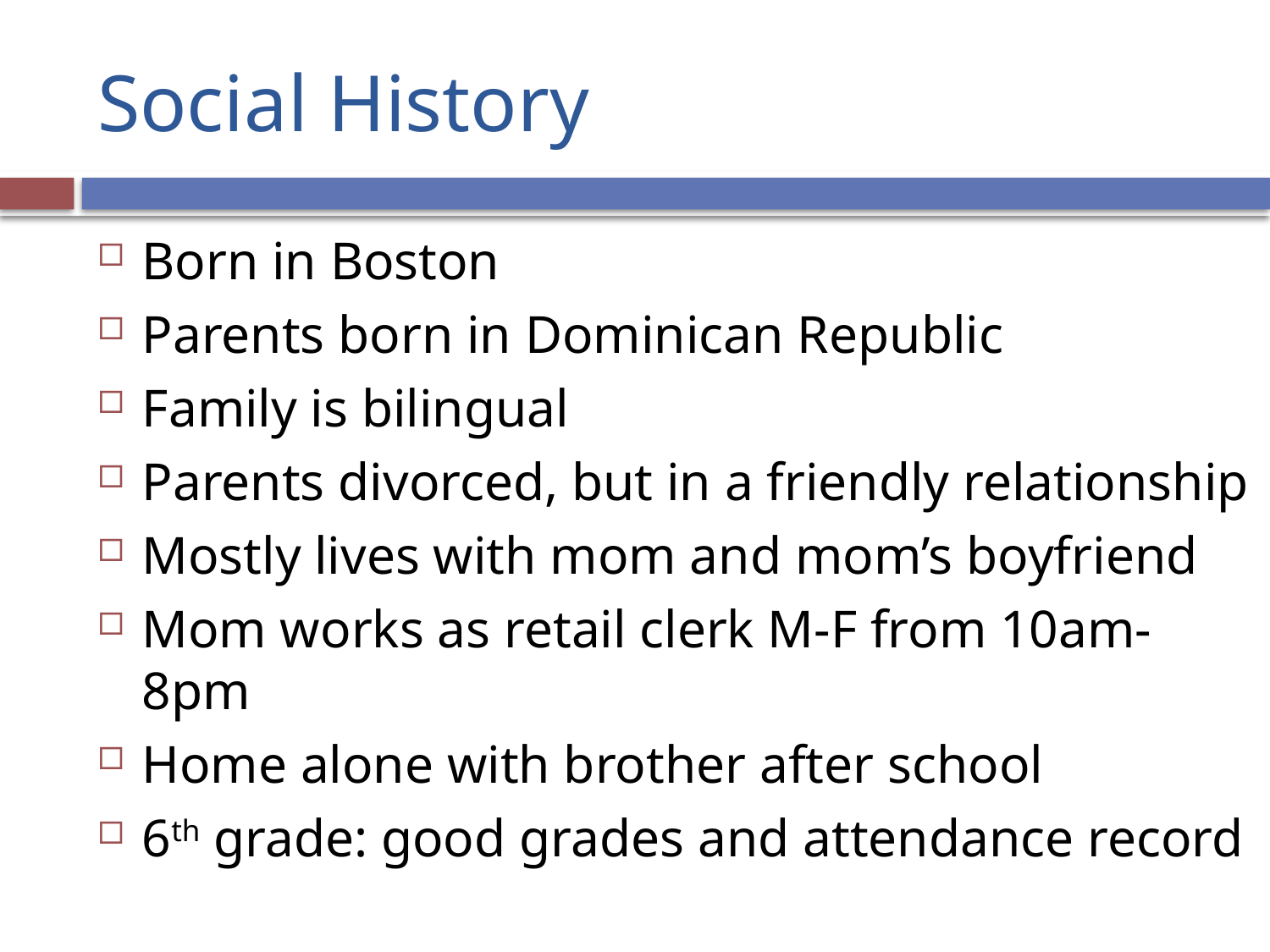

# Social History
Born in Boston
Parents born in Dominican Republic
Family is bilingual
Parents divorced, but in a friendly relationship
Mostly lives with mom and mom’s boyfriend
Mom works as retail clerk M-F from 10am-8pm
Home alone with brother after school
6th grade: good grades and attendance record

## Slide 13
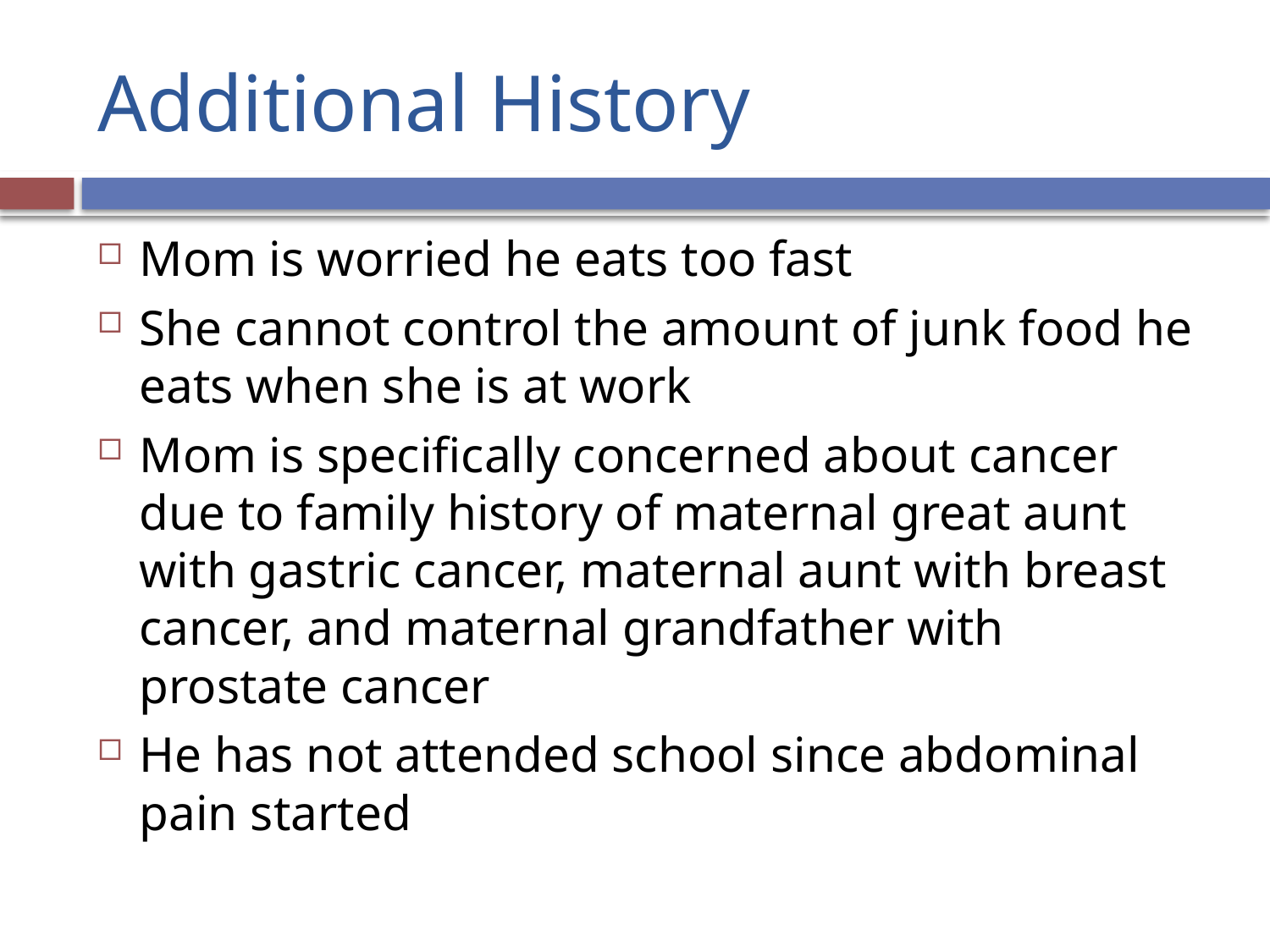

# Additional History
Mom is worried he eats too fast
She cannot control the amount of junk food he eats when she is at work
Mom is specifically concerned about cancer due to family history of maternal great aunt with gastric cancer, maternal aunt with breast cancer, and maternal grandfather with prostate cancer
He has not attended school since abdominal pain started

## Slide 14
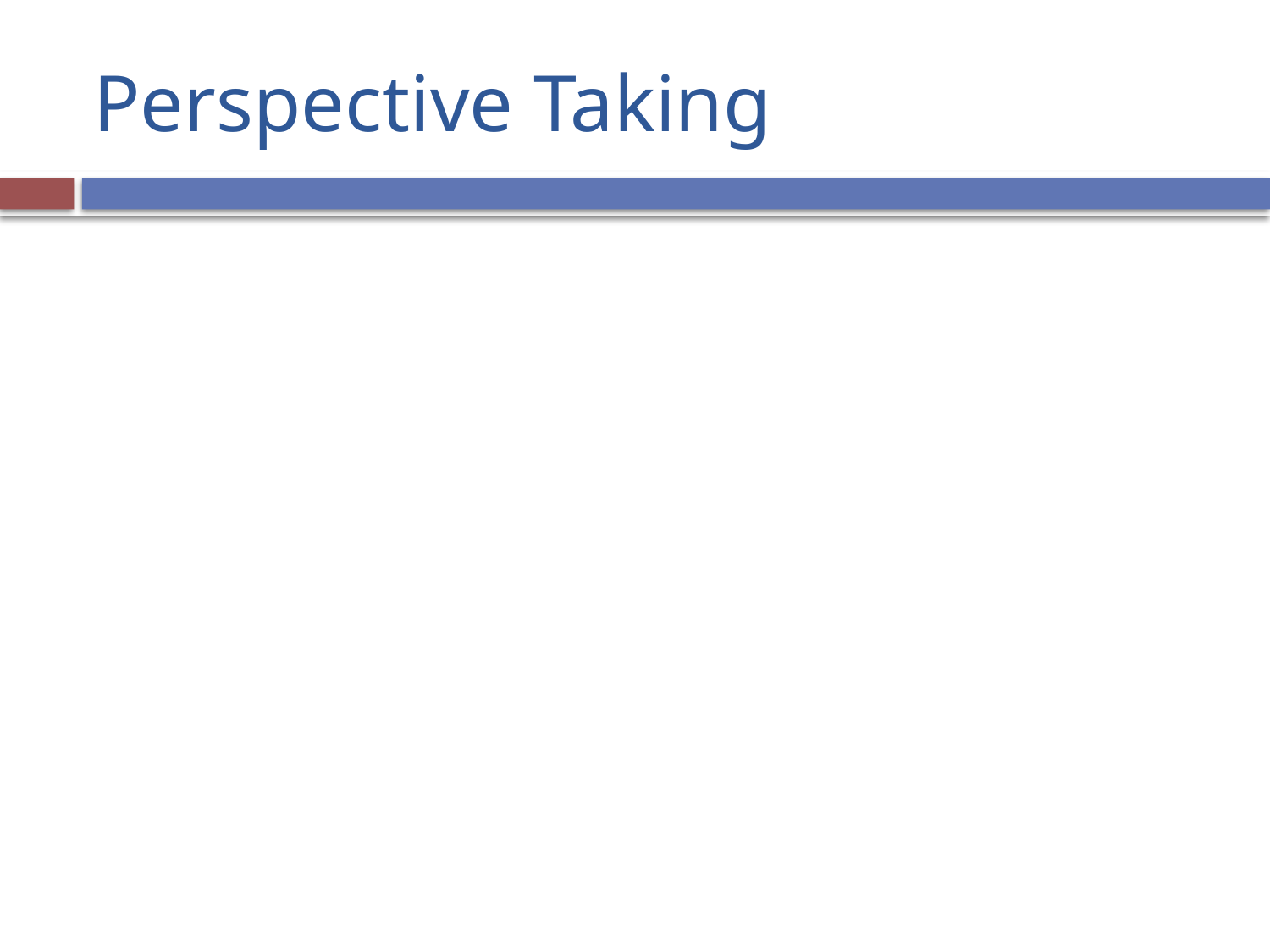

# Perspective Taking

## Slide 15
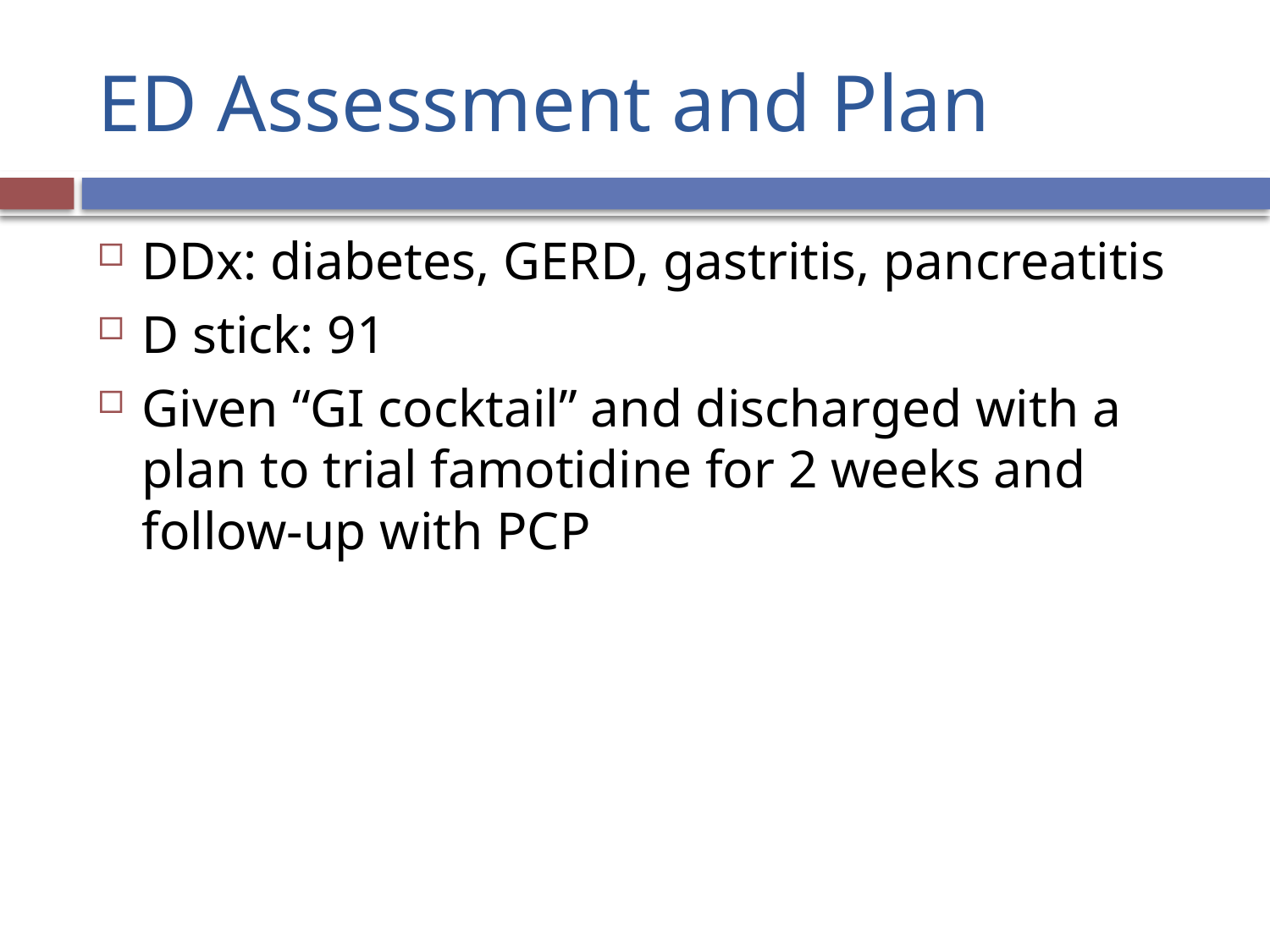

# ED Assessment and Plan
DDx: diabetes, GERD, gastritis, pancreatitis
D stick: 91
Given “GI cocktail” and discharged with a plan to trial famotidine for 2 weeks and follow-up with PCP

## Slide 16
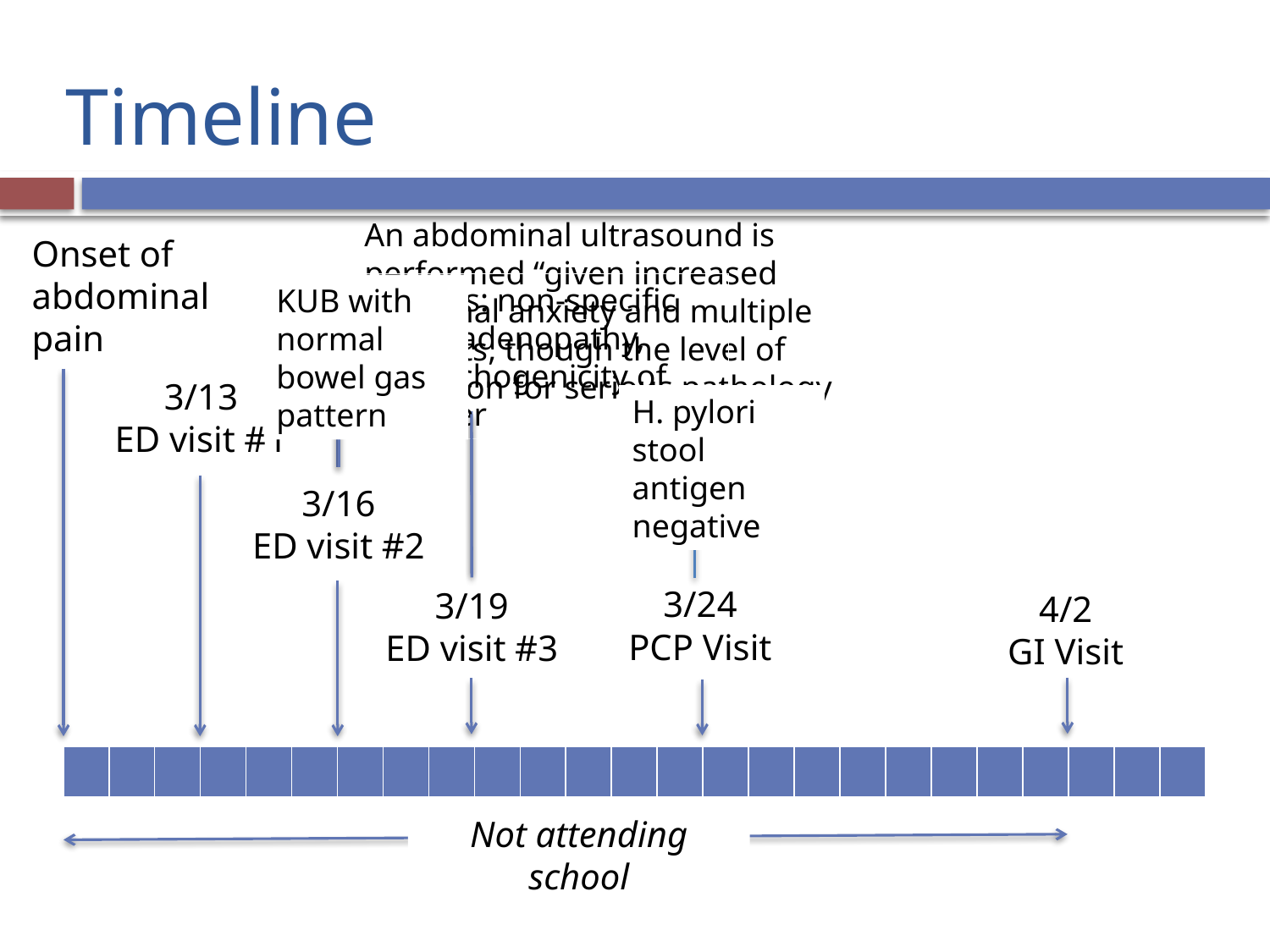

# Timeline
An abdominal ultrasound is performed “given increased maternal anxiety and multiple ED visits, though the level of suspicion for serious pathology is low.”
Onset of abdominal pain
Abd u/s: non-specific lymphadenopathy, mild echogenicity of the liver
CBC, LFTs, lipase normal
KUB with normal bowel gas pattern
3/13
ED visit #1
H. pylori stool antigen negative
3/16
ED visit #2
3/24
PCP Visit
3/19
ED visit #3
4/2
GI Visit
| | | | | | | | | | | | | | | | | | | | | | | | | |
| --- | --- | --- | --- | --- | --- | --- | --- | --- | --- | --- | --- | --- | --- | --- | --- | --- | --- | --- | --- | --- | --- | --- | --- | --- |
Not attending school

## Slide 17
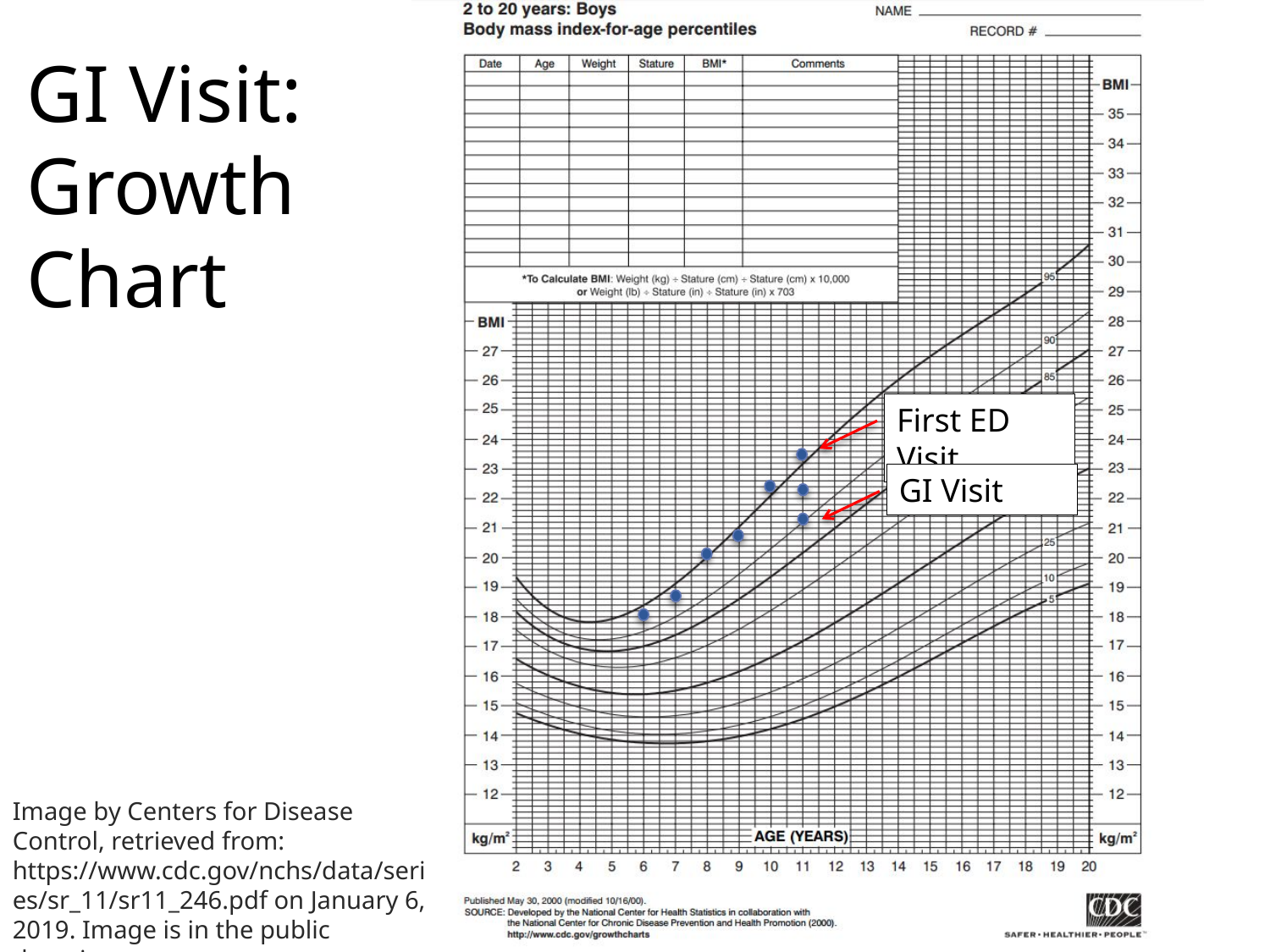

GI Visit:
Growth Chart
First ED Visit
GI Visit
Image by Centers for Disease Control, retrieved from: https://www.cdc.gov/nchs/data/series/sr_11/sr11_246.pdf on January 6, 2019. Image is in the public domain.

## Slide 18
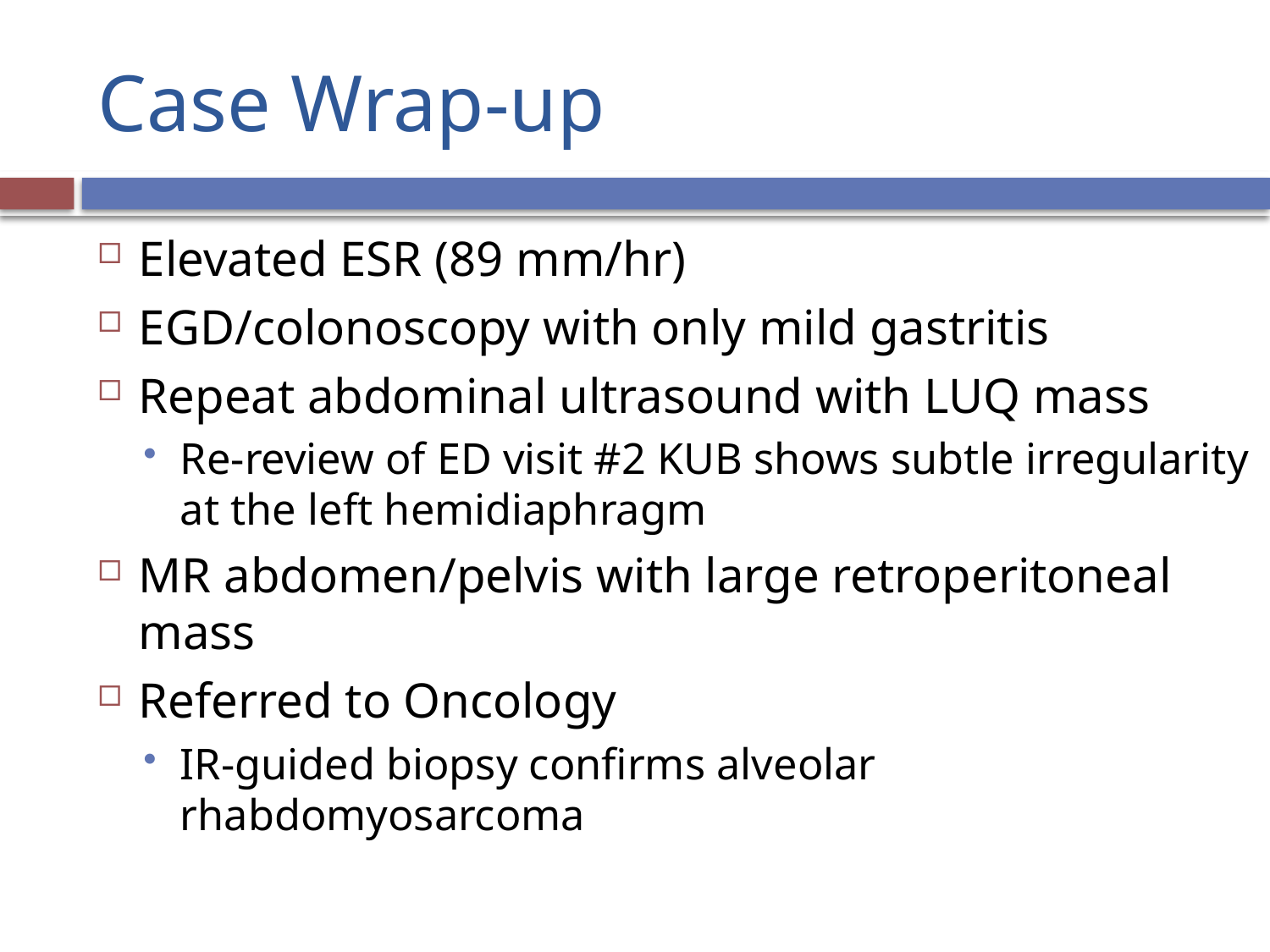

# Case Wrap-up
Elevated ESR (89 mm/hr)
EGD/colonoscopy with only mild gastritis
Repeat abdominal ultrasound with LUQ mass
Re-review of ED visit #2 KUB shows subtle irregularity at the left hemidiaphragm
MR abdomen/pelvis with large retroperitoneal mass
Referred to Oncology
IR-guided biopsy confirms alveolar rhabdomyosarcoma

## Slide 19
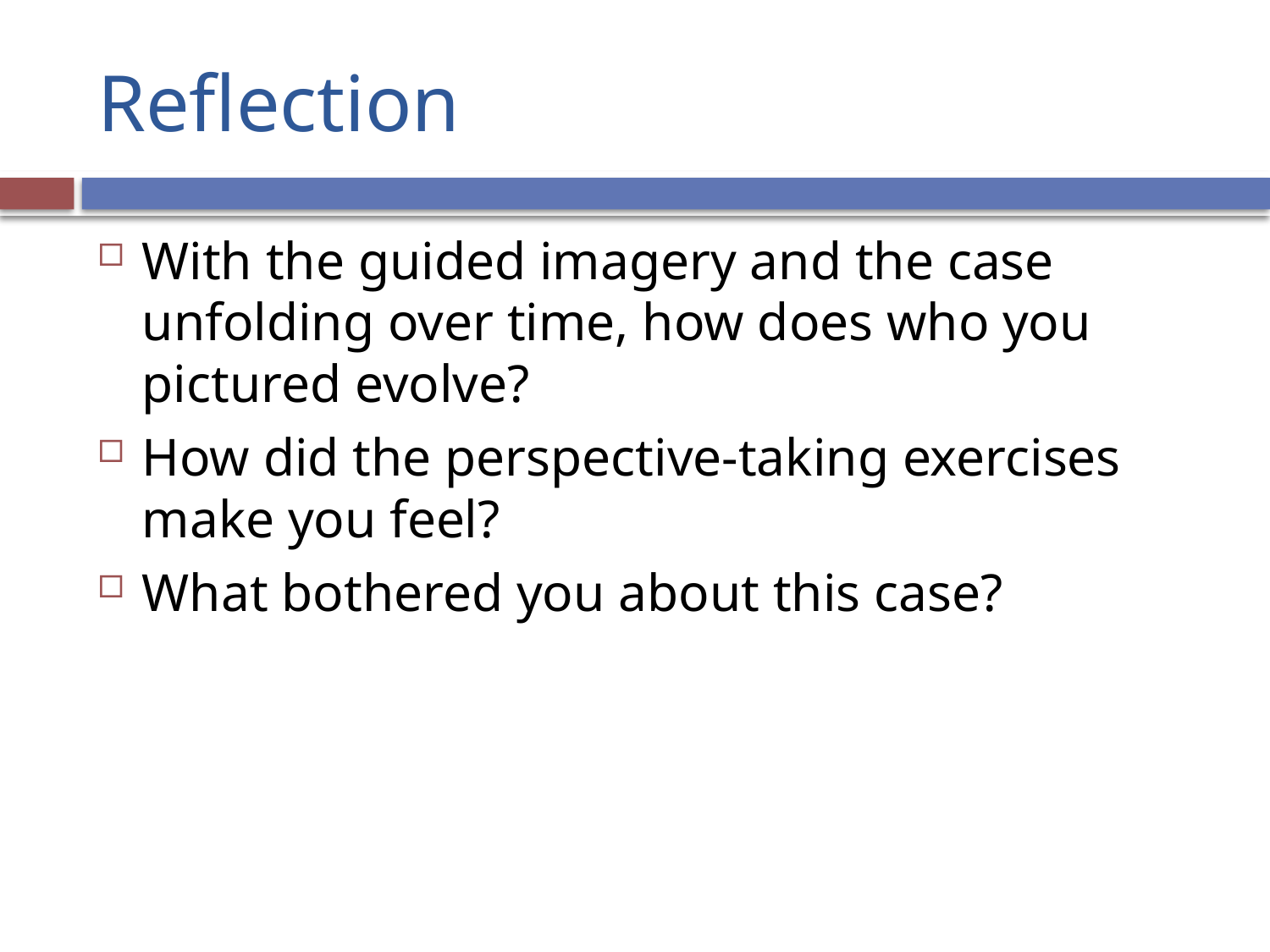

# Reflection
With the guided imagery and the case unfolding over time, how does who you pictured evolve?
How did the perspective-taking exercises make you feel?
What bothered you about this case?

## Slide 20
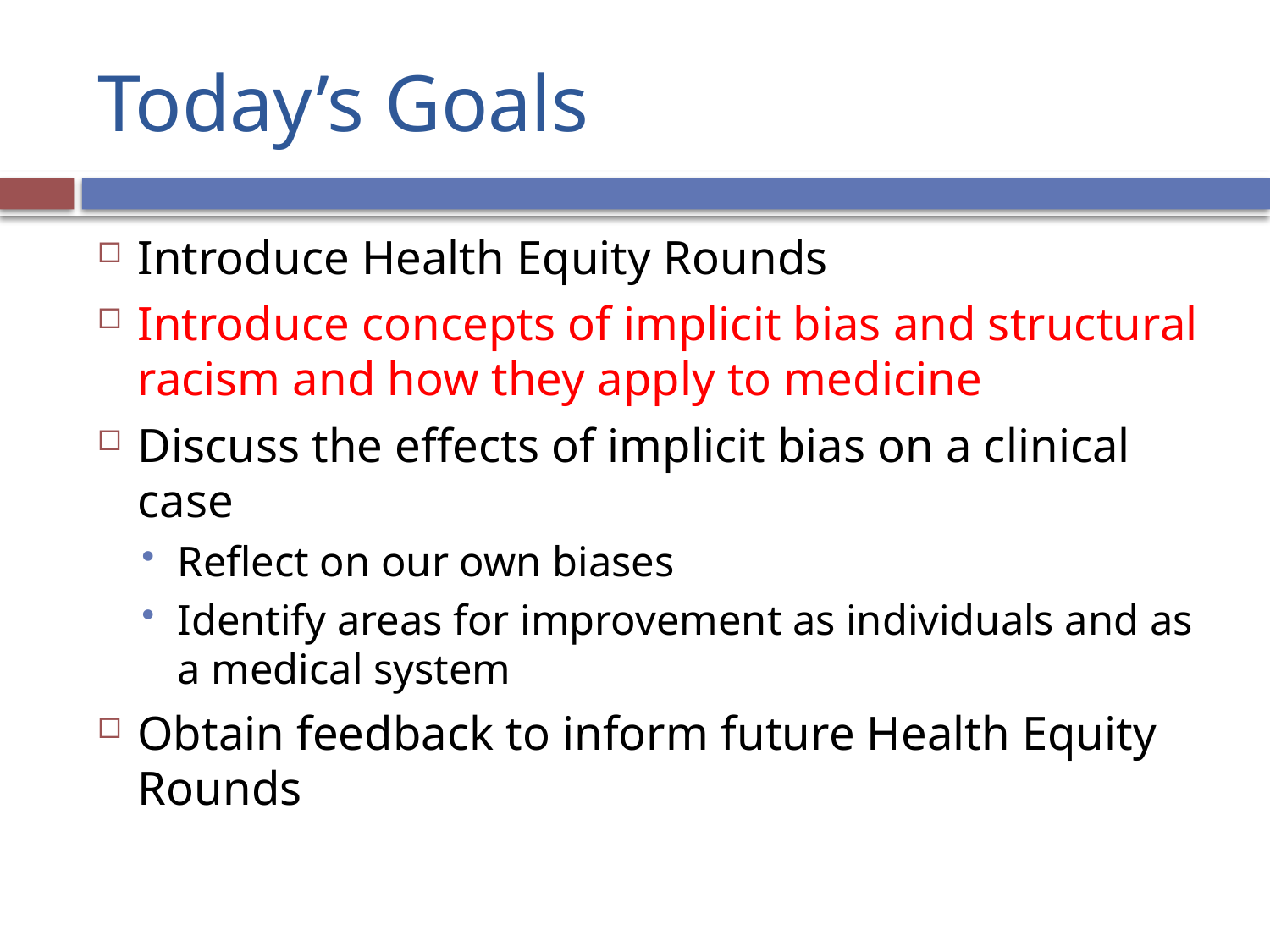

# Today’s Goals
Introduce Health Equity Rounds
Introduce concepts of implicit bias and structural racism and how they apply to medicine
Discuss the effects of implicit bias on a clinical case
Reflect on our own biases
Identify areas for improvement as individuals and as a medical system
Obtain feedback to inform future Health Equity Rounds

## Slide 21
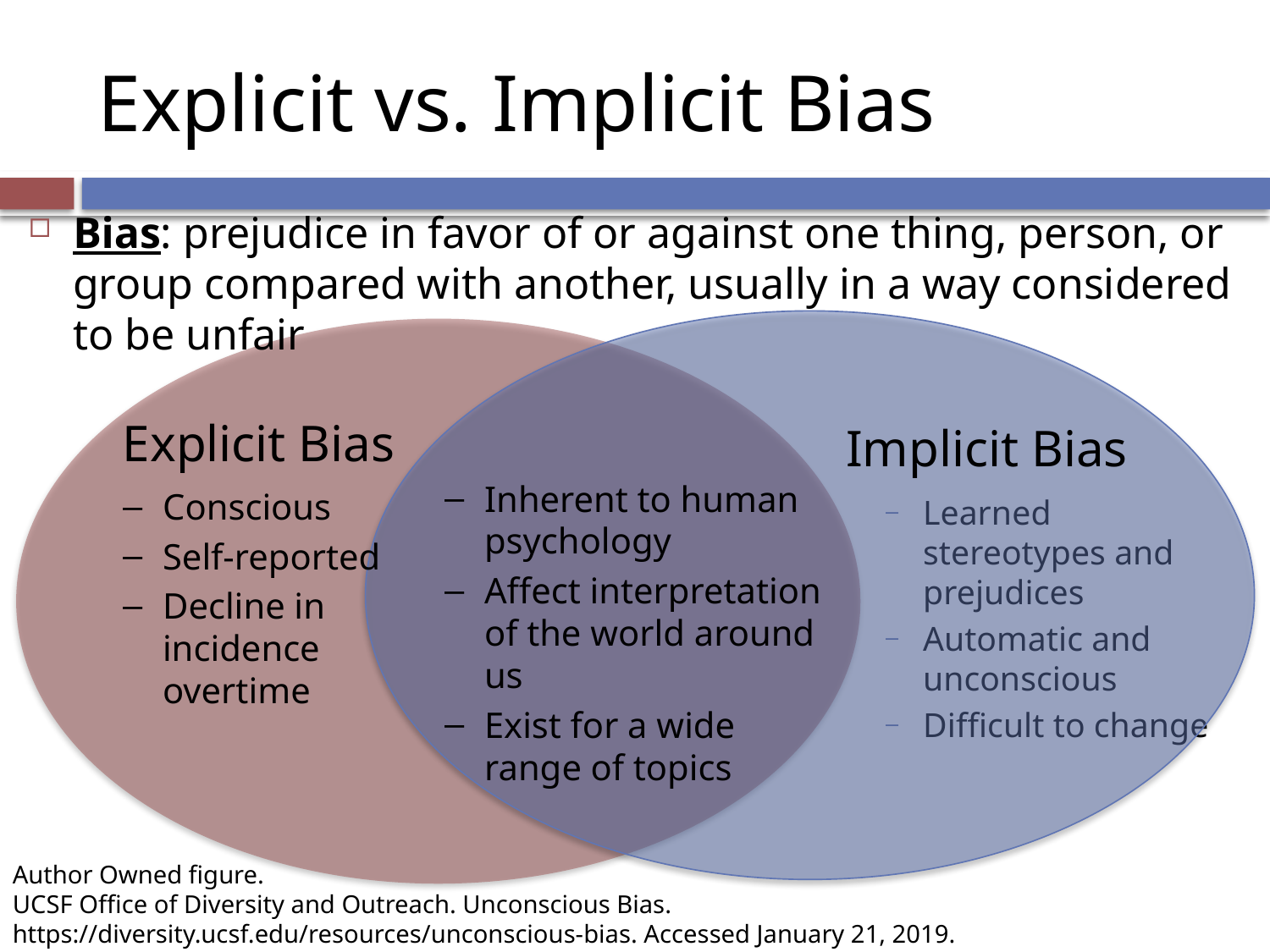

# Explicit vs. Implicit Bias
Bias: prejudice in favor of or against one thing, person, or group compared with another, usually in a way considered to be unfair
Explicit Bias
Implicit Bias
Inherent to human psychology
Affect interpretation of the world around us
Exist for a wide range of topics
Conscious
Self-reported
Decline in incidence overtime
Learned stereotypes and prejudices
Automatic and unconscious
Difficult to change
Author Owned figure.
UCSF Office of Diversity and Outreach. Unconscious Bias. https://diversity.ucsf.edu/resources/unconscious-bias. Accessed January 21, 2019.

## Slide 22
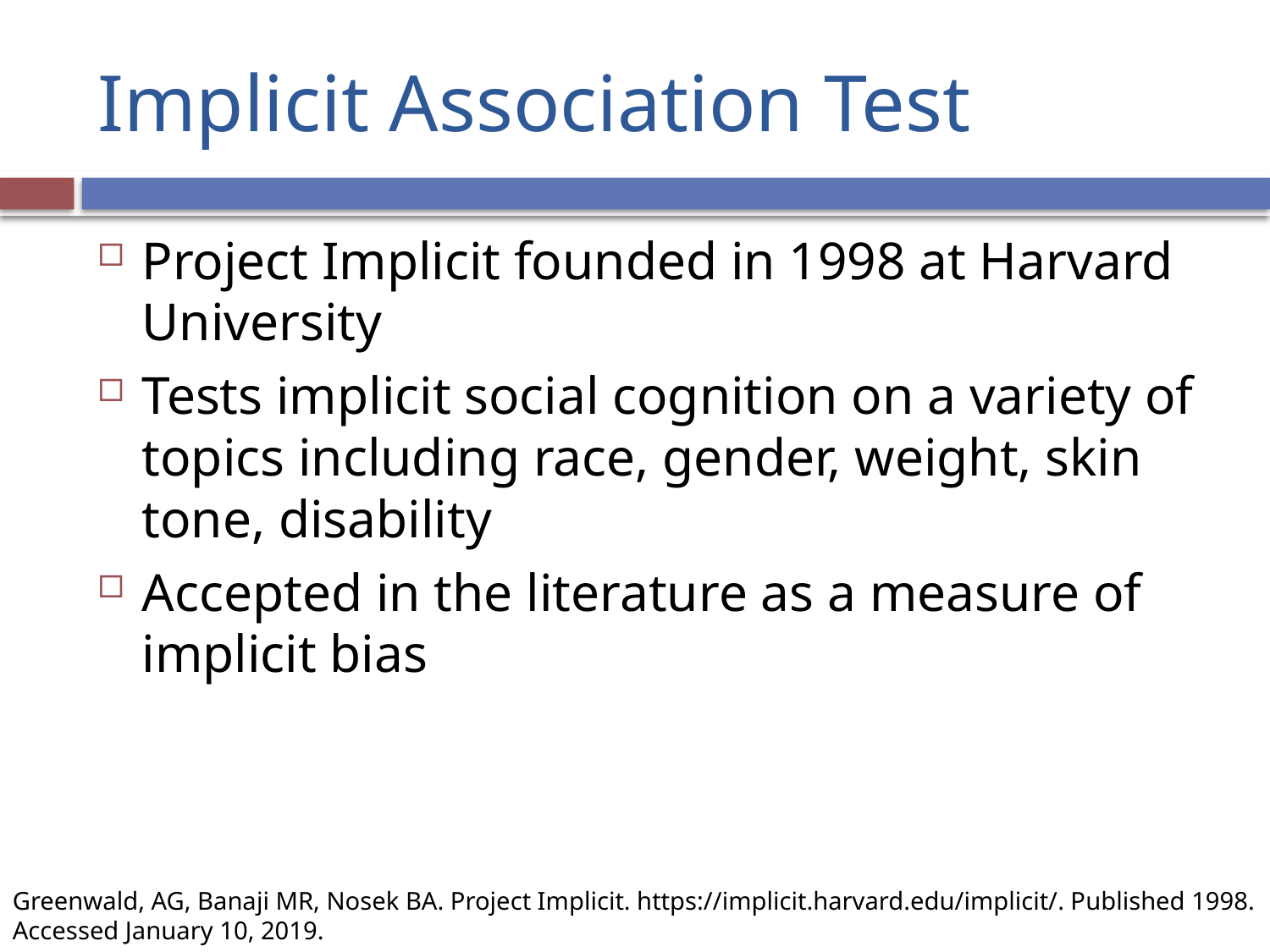

# Implicit Association Test
Project Implicit founded in 1998 at Harvard University
Tests implicit social cognition on a variety of topics including race, gender, weight, skin tone, disability
Accepted in the literature as a measure of implicit bias
Greenwald, AG, Banaji MR, Nosek BA. Project Implicit. https://implicit.harvard.edu/implicit/. Published 1998. Accessed January 10, 2019.

## Slide 23
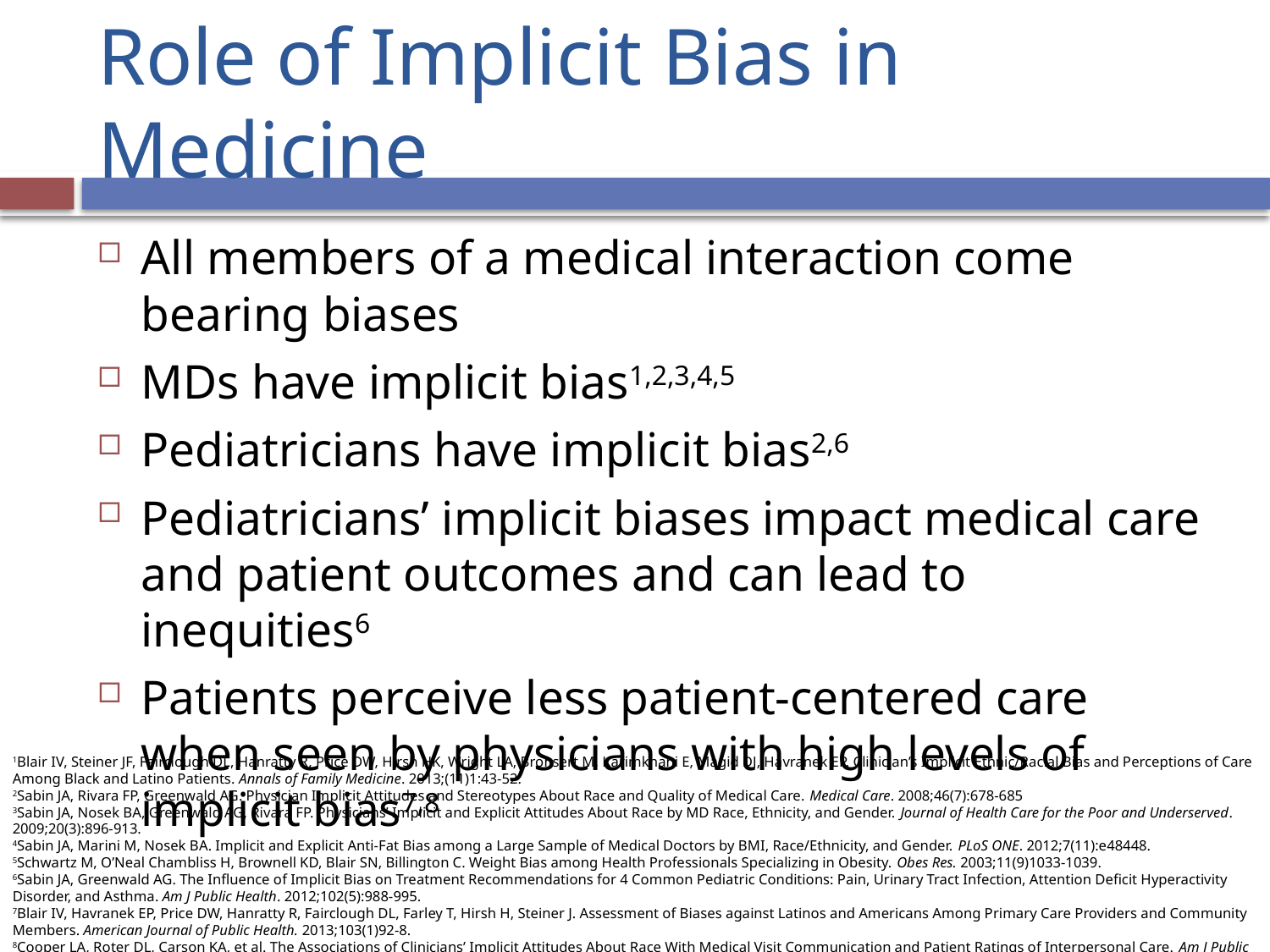

# Role of Implicit Bias in Medicine
All members of a medical interaction come bearing biases
MDs have implicit bias1,2,3,4,5
Pediatricians have implicit bias2,6
Pediatricians’ implicit biases impact medical care and patient outcomes and can lead to inequities6
Patients perceive less patient-centered care when seen by physicians with high levels of implicit bias7,8
1Blair IV, Steiner JF, Fairclough DL, Hanratty R, Price DW, Hirsh HK, Wright LA, Bronsert M, Karimkhani E, Magid DJ, Havranek EP. Clinician’s Implicit Ethnic/Racial Bias and Perceptions of Care Among Black and Latino Patients. Annals of Family Medicine. 2013;(11)1:43-52.
2Sabin JA, Rivara FP, Greenwald AG. Physician Implicit Attitudes and Stereotypes About Race and Quality of Medical Care. Medical Care. 2008;46(7):678-685
3Sabin JA, Nosek BA, Greenwald AG, Rivara FP. Physicians’ Implicit and Explicit Attitudes About Race by MD Race, Ethnicity, and Gender. Journal of Health Care for the Poor and Underserved. 2009;20(3):896-913.
4Sabin JA, Marini M, Nosek BA. Implicit and Explicit Anti-Fat Bias among a Large Sample of Medical Doctors by BMI, Race/Ethnicity, and Gender. PLoS ONE. 2012;7(11):e48448.
5Schwartz M, O’Neal Chambliss H, Brownell KD, Blair SN, Billington C. Weight Bias among Health Professionals Specializing in Obesity. Obes Res. 2003;11(9)1033-1039.
6Sabin JA, Greenwald AG. The Influence of Implicit Bias on Treatment Recommendations for 4 Common Pediatric Conditions: Pain, Urinary Tract Infection, Attention Deficit Hyperactivity Disorder, and Asthma. Am J Public Health. 2012;102(5):988-995.
7Blair IV, Havranek EP, Price DW, Hanratty R, Fairclough DL, Farley T, Hirsh H, Steiner J. Assessment of Biases against Latinos and Americans Among Primary Care Providers and Community Members. American Journal of Public Health. 2013;103(1)92-8.
8Cooper LA, Roter DL, Carson KA, et al. The Associations of Clinicians’ Implicit Attitudes About Race With Medical Visit Communication and Patient Ratings of Interpersonal Care. Am J Public Health 2012;102(5):979-987.

## Slide 24
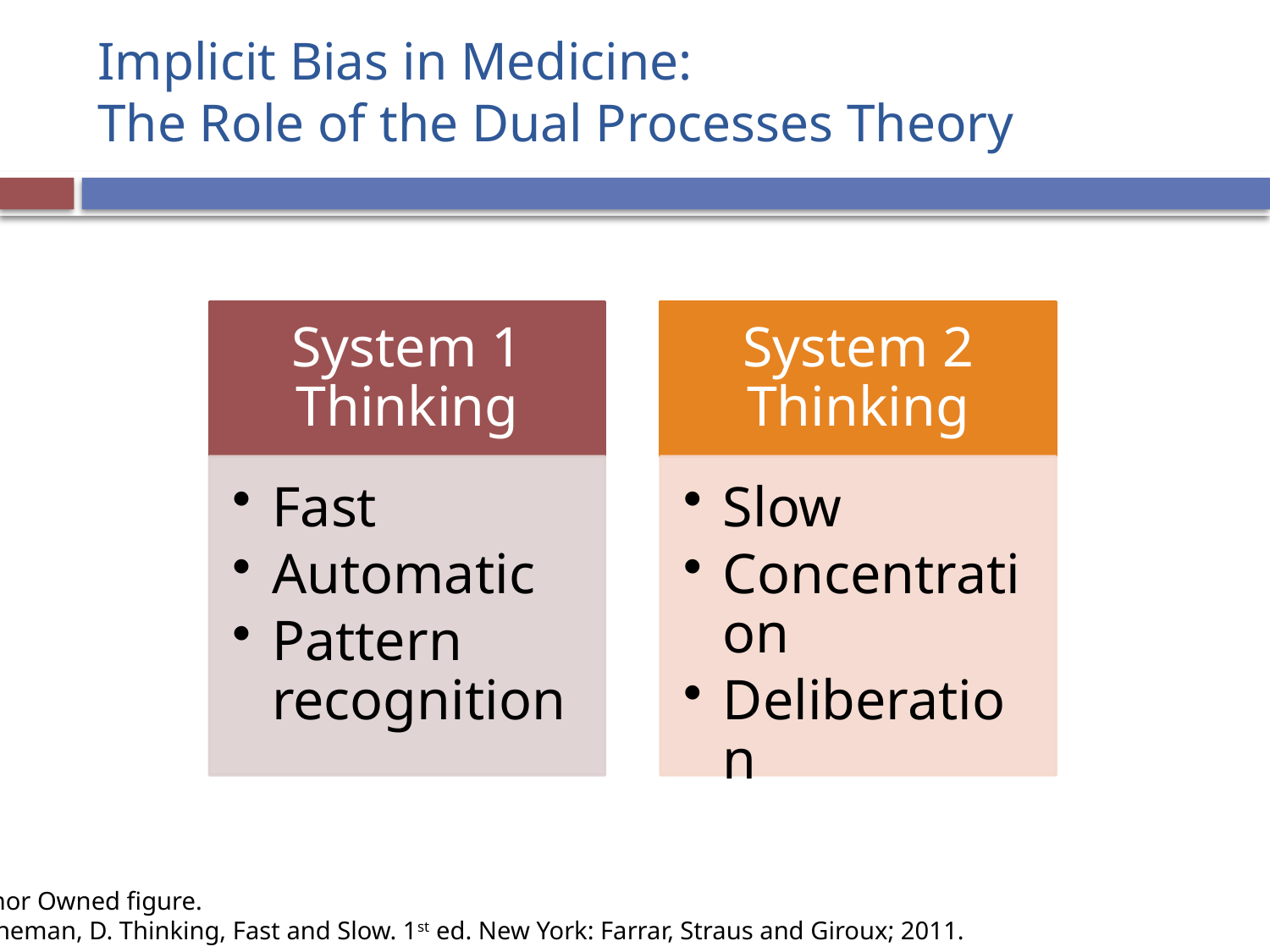

# Implicit Bias in Medicine: The Role of the Dual Processes Theory
Author Owned figure.
Kahneman, D. Thinking, Fast and Slow. 1st ed. New York: Farrar, Straus and Giroux; 2011.

## Slide 25
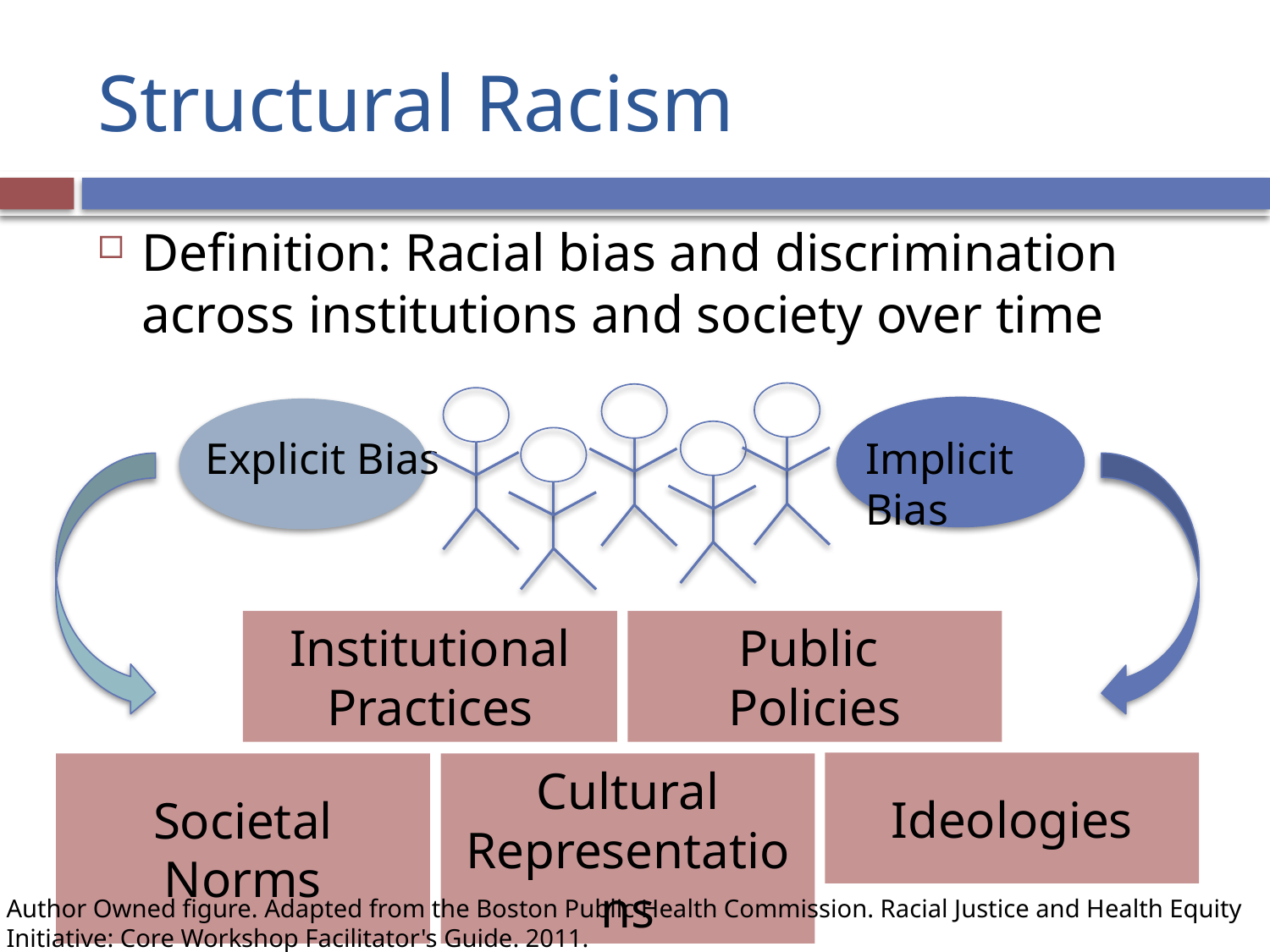

# Structural Racism
Definition: Racial bias and discrimination across institutions and society over time
Explicit Bias
Implicit Bias
Institutional Practices
Public
Policies
Ideologies
Societal Norms
Cultural
Representations
Author Owned figure. Adapted from the Boston Public Health Commission. Racial Justice and Health Equity Initiative: Core Workshop Facilitator's Guide. 2011.

## Slide 26
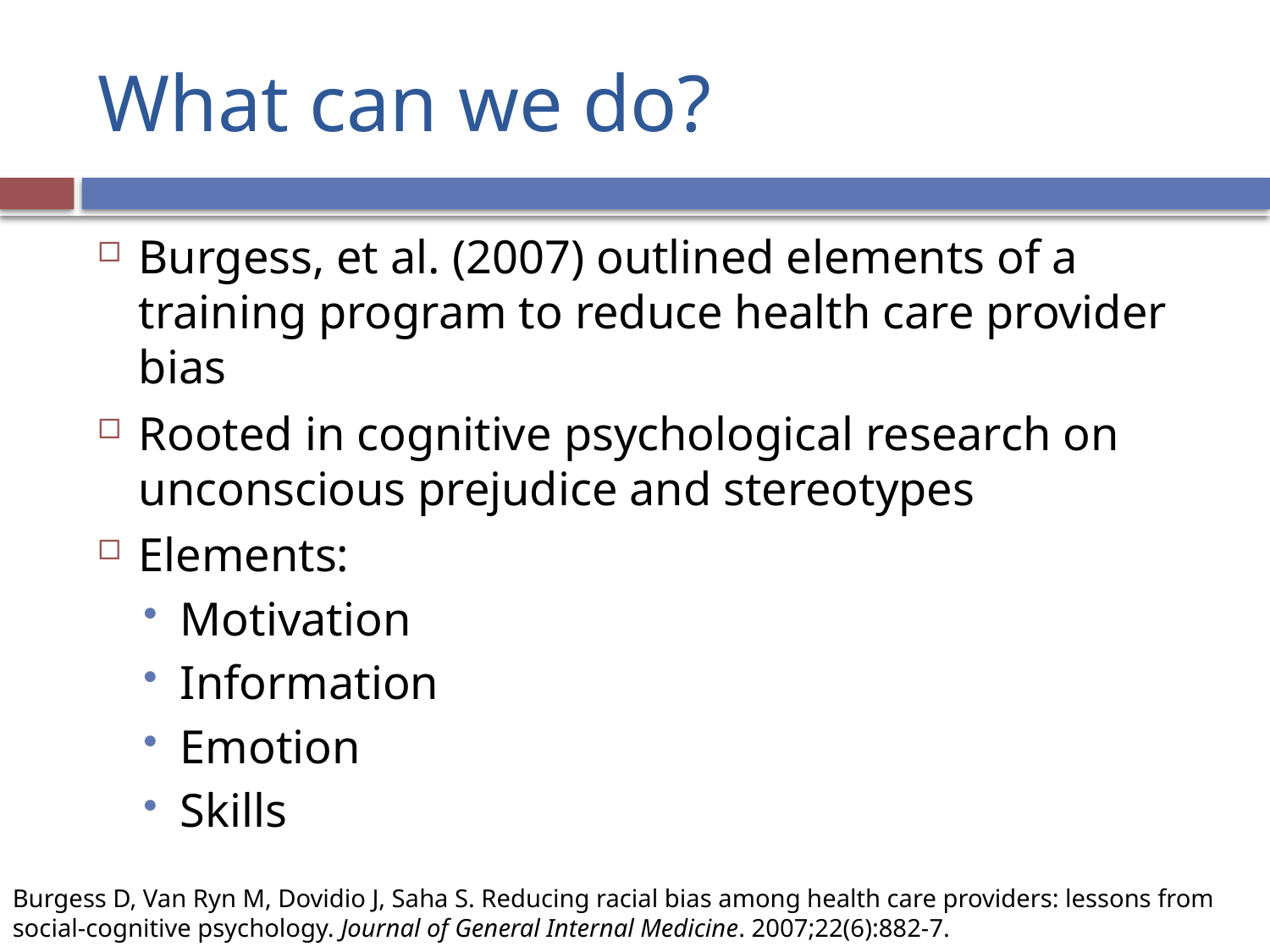

# What can we do?
Burgess, et al. (2007) outlined elements of a training program to reduce health care provider bias
Rooted in cognitive psychological research on unconscious prejudice and stereotypes
Elements:
Motivation
Information
Emotion
Skills
Burgess D, Van Ryn M, Dovidio J, Saha S. Reducing racial bias among health care providers: lessons from social-cognitive psychology. Journal of General Internal Medicine. 2007;22(6):882-7.

## Slide 27
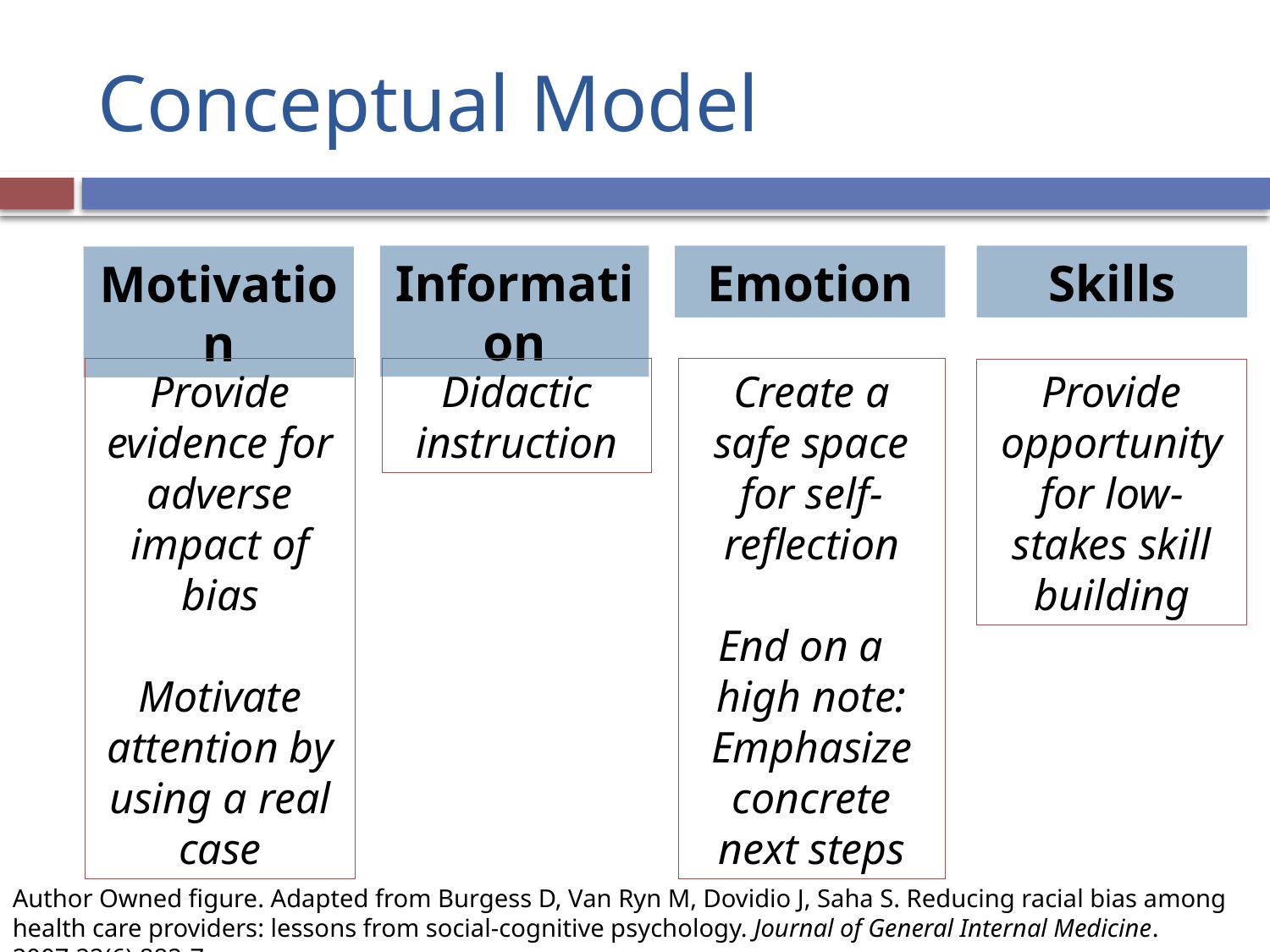

# Conceptual Model
Skills
Information
Emotion
Motivation
Create a safe space for self-reflection
d
End on a high note: Emphasize concrete next steps
Provide evidence for adverse impact of bias
Motivate attention by using a real case
Didactic instruction
Provide opportunity for low-stakes skill building
Author Owned figure. Adapted from Burgess D, Van Ryn M, Dovidio J, Saha S. Reducing racial bias among health care providers: lessons from social-cognitive psychology. Journal of General Internal Medicine. 2007;22(6):882-7.

## Slide 28
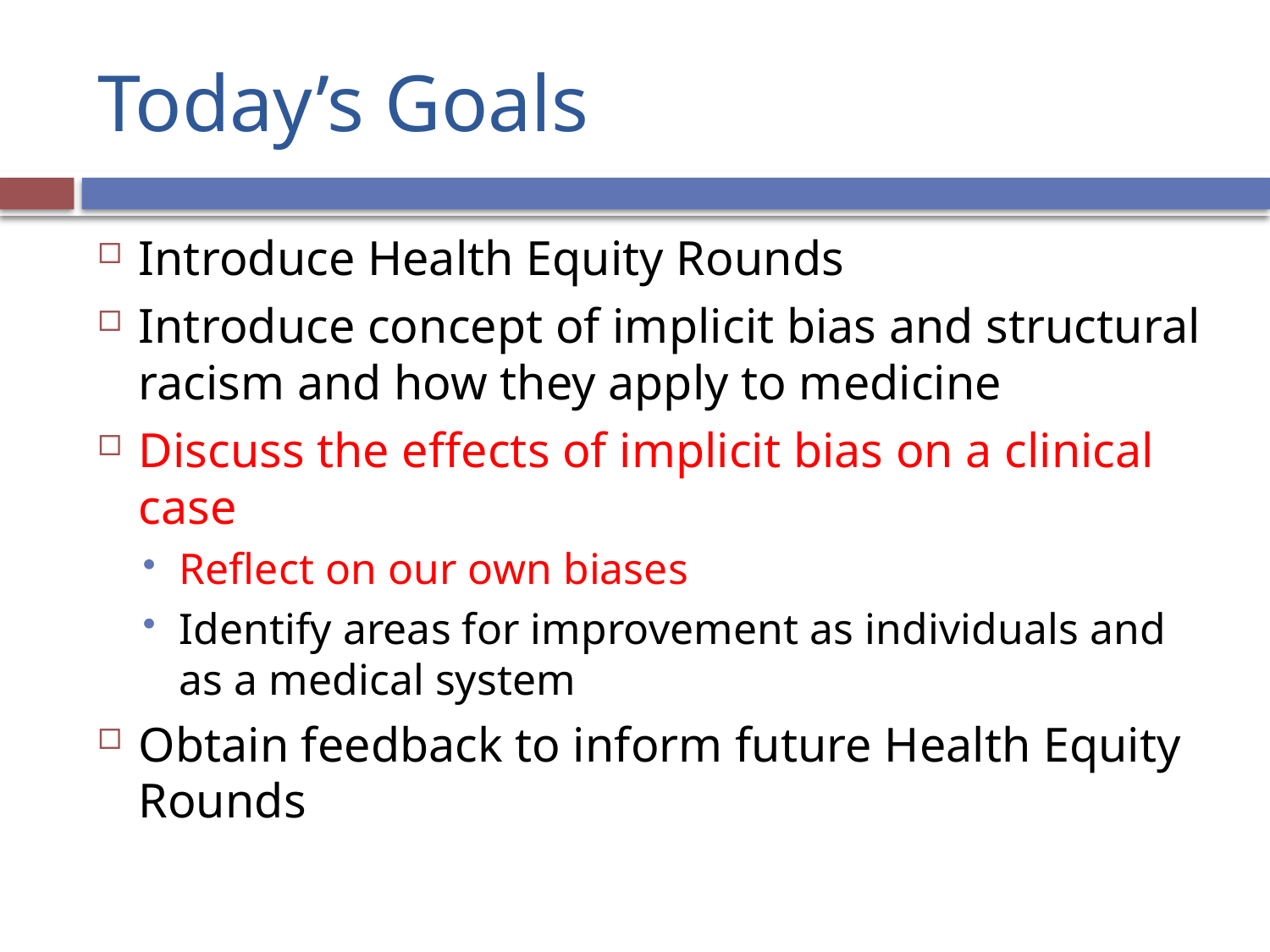

# Today’s Goals
Introduce Health Equity Rounds
Introduce concept of implicit bias and structural racism and how they apply to medicine
Discuss the effects of implicit bias on a clinical case
Reflect on our own biases
Identify areas for improvement as individuals and as a medical system
Obtain feedback to inform future Health Equity Rounds

## Slide 29
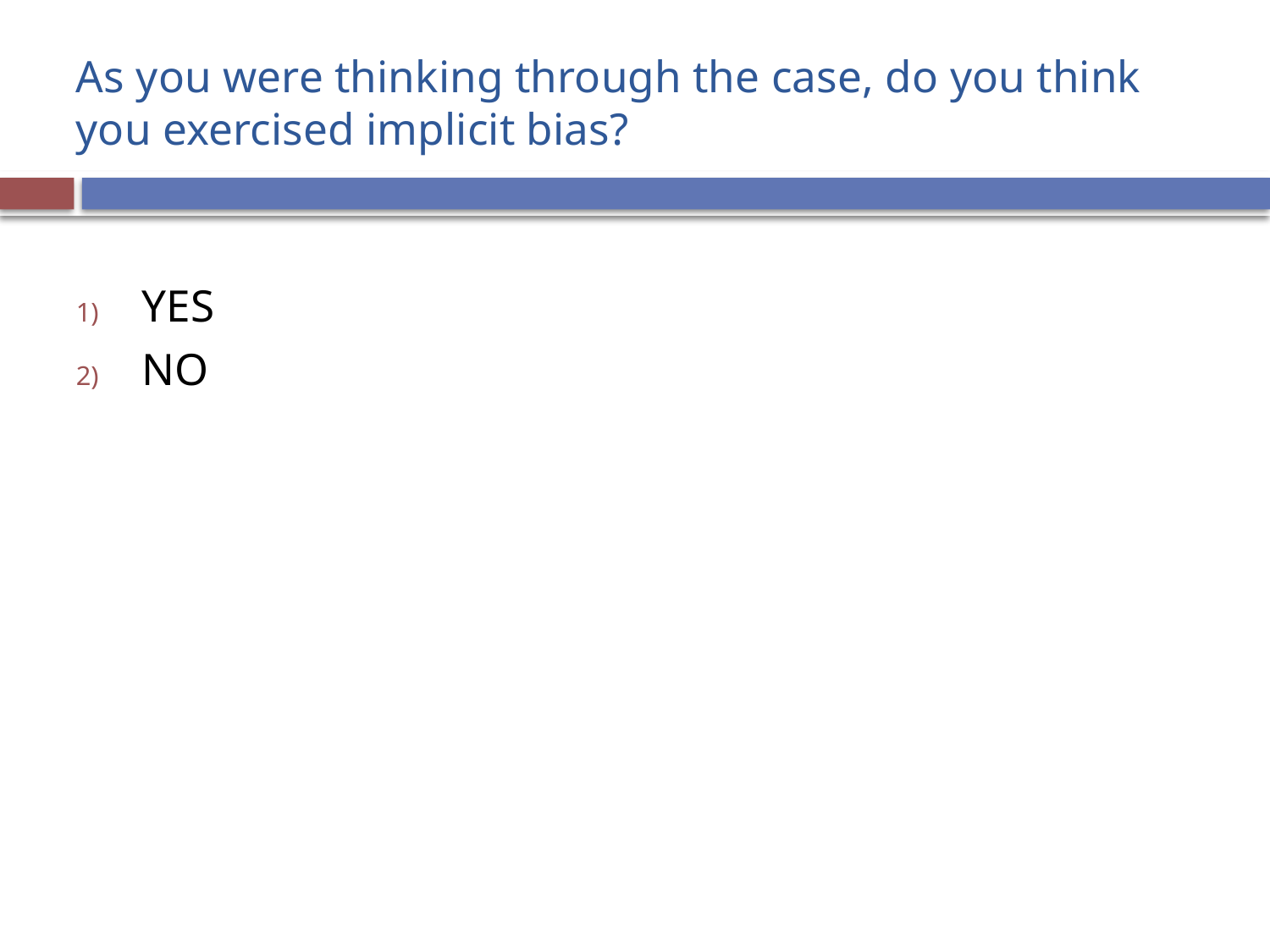

As you were thinking through the case, do you think you exercised implicit bias?
YES
NO

## Slide 30
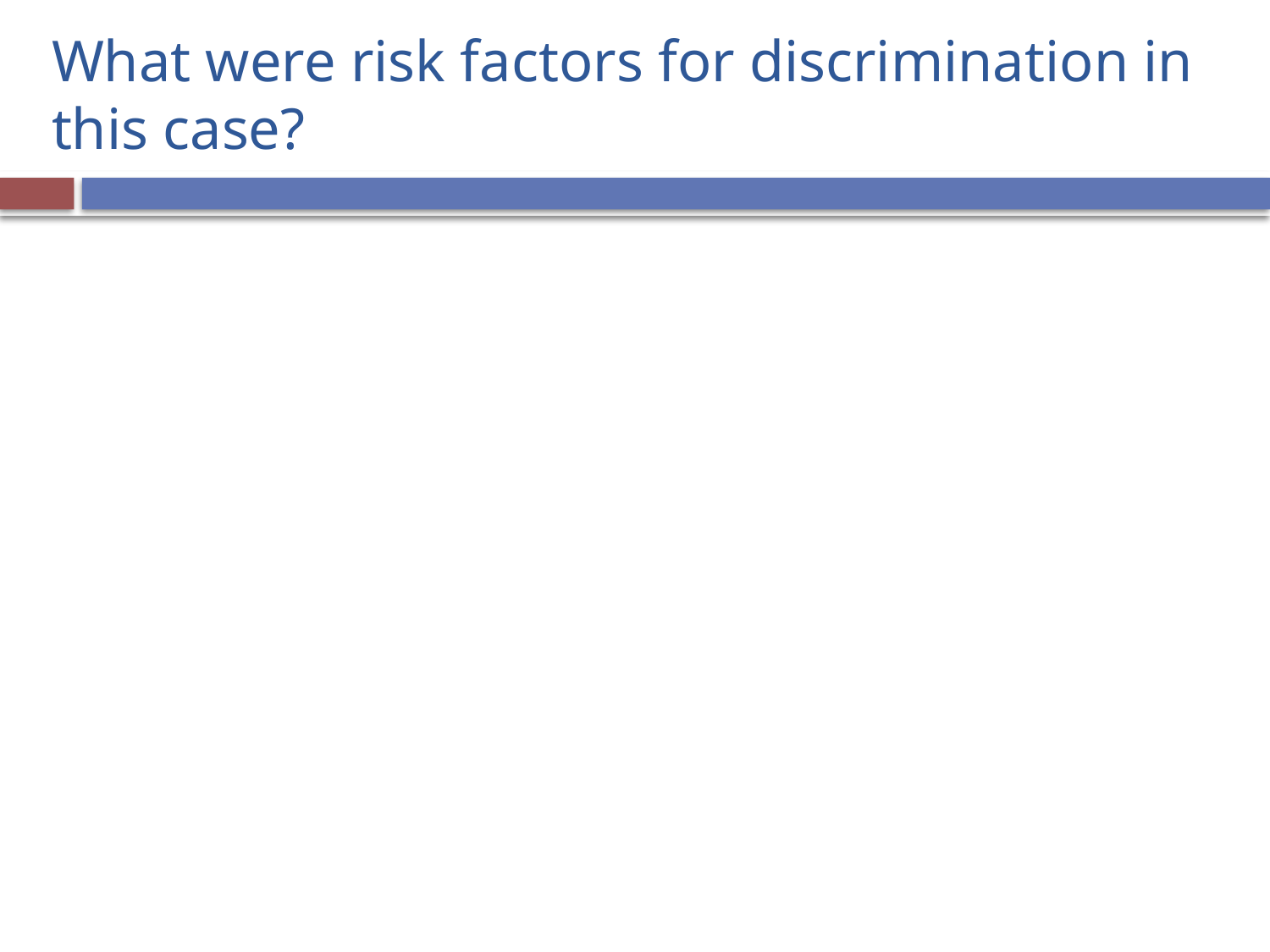

# What were risk factors for discrimination in this case?

## Slide 31
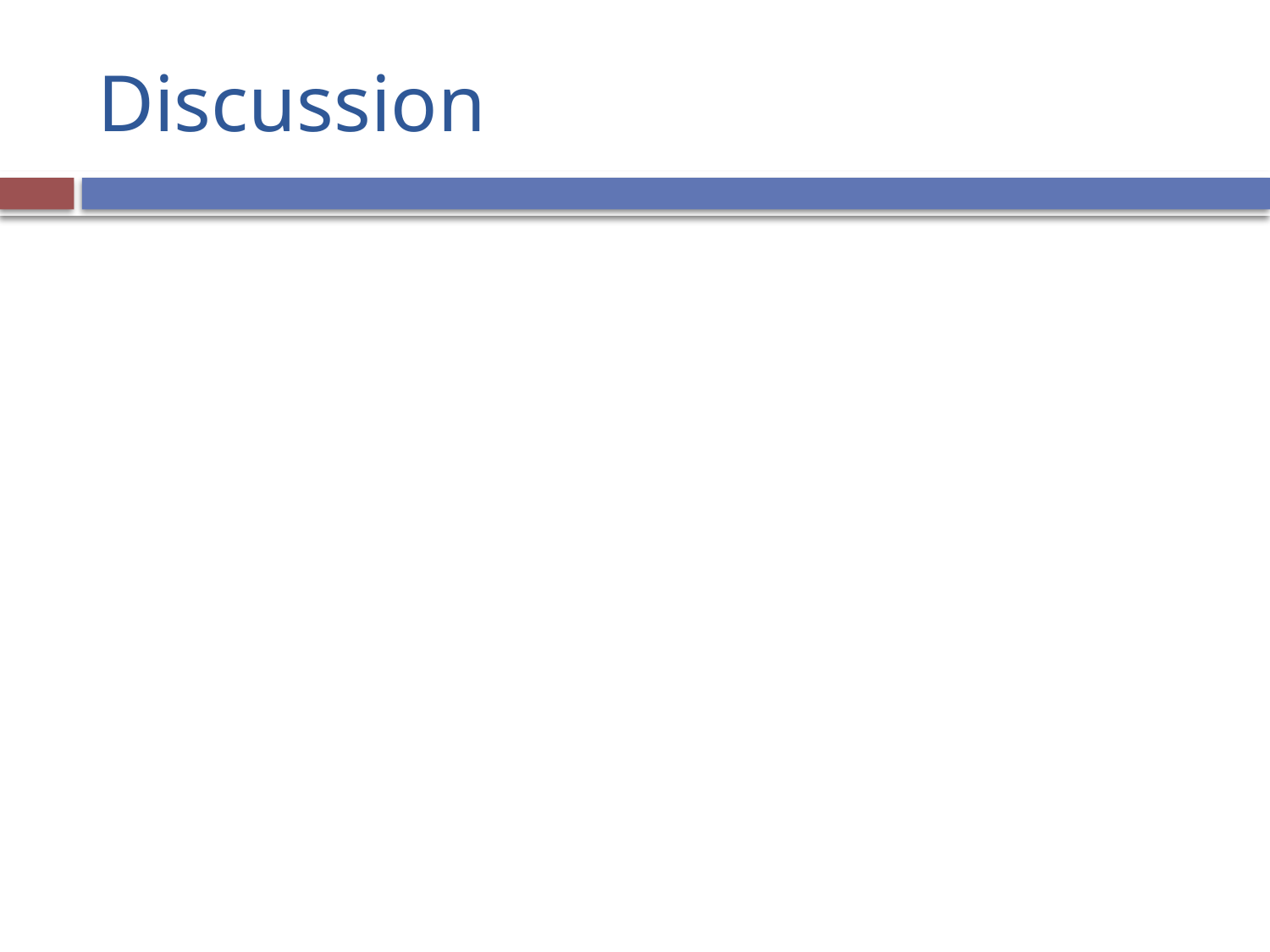

# Discussion

## Slide 32
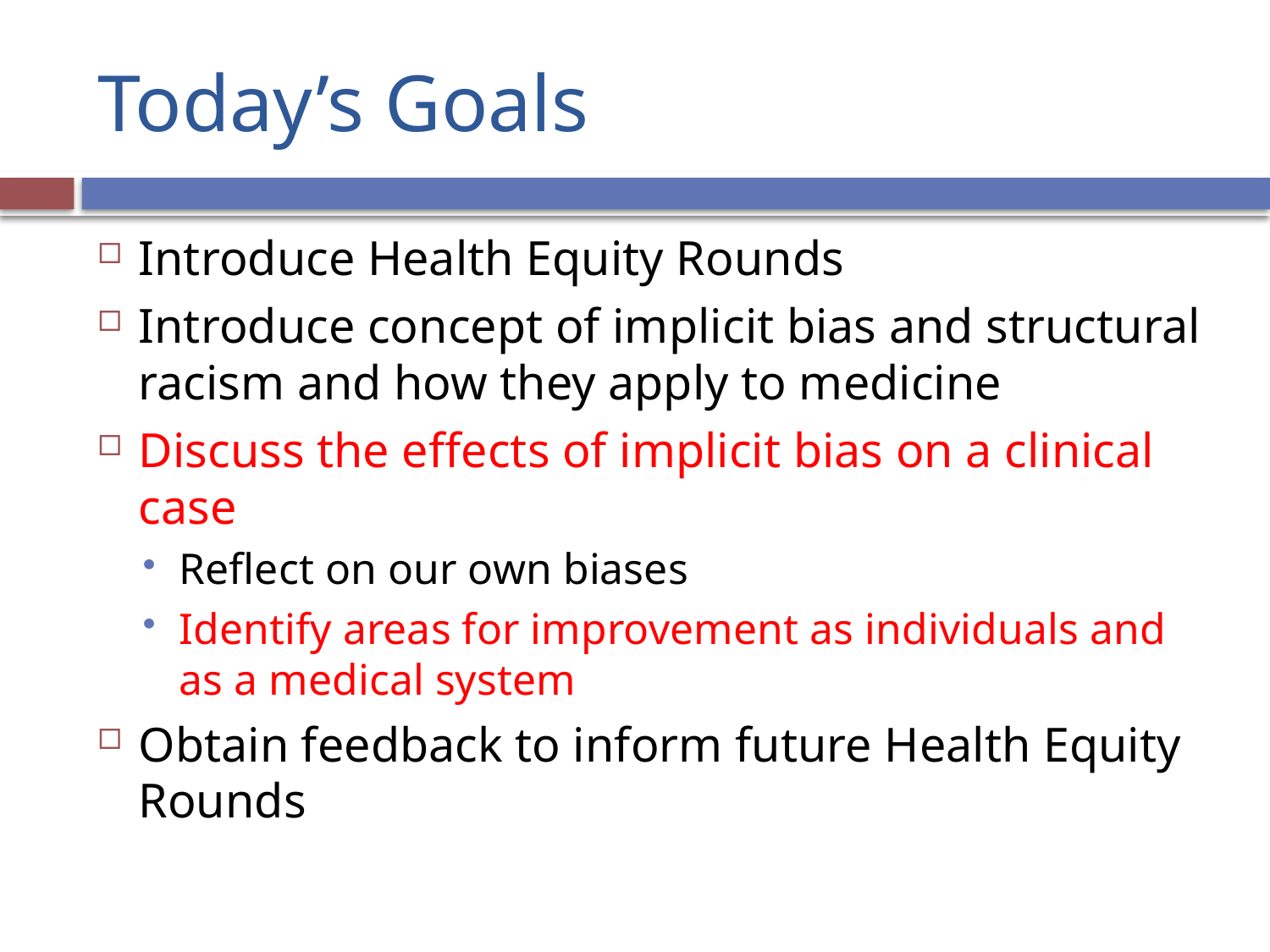

# Today’s Goals
Introduce Health Equity Rounds
Introduce concept of implicit bias and structural racism and how they apply to medicine
Discuss the effects of implicit bias on a clinical case
Reflect on our own biases
Identify areas for improvement as individuals and as a medical system
Obtain feedback to inform future Health Equity Rounds

## Slide 33
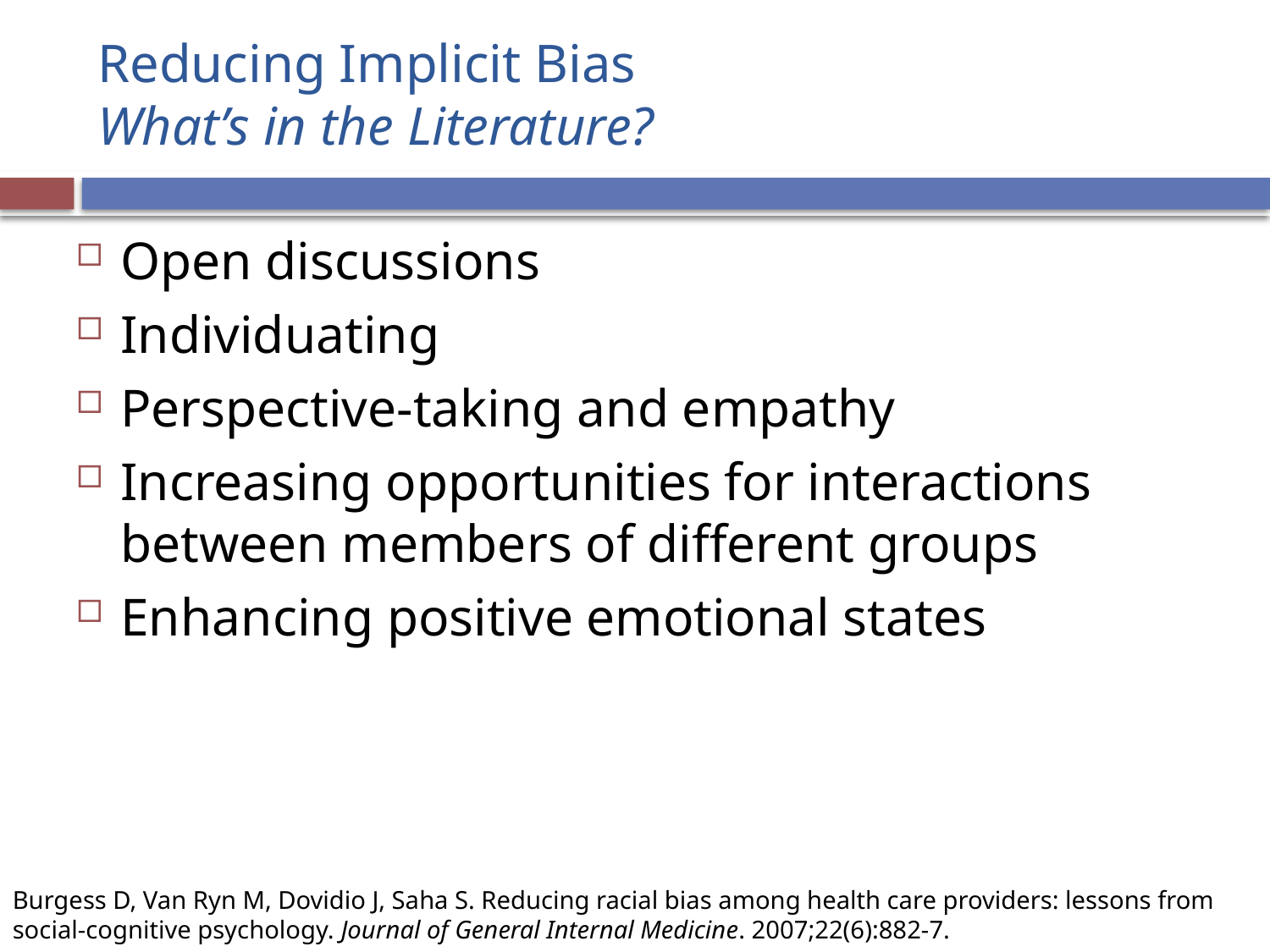

# Reducing Implicit Bias What’s in the Literature?
Open discussions
Individuating
Perspective-taking and empathy
Increasing opportunities for interactions between members of different groups
Enhancing positive emotional states
Burgess D, Van Ryn M, Dovidio J, Saha S. Reducing racial bias among health care providers: lessons from social-cognitive psychology. Journal of General Internal Medicine. 2007;22(6):882-7.

## Slide 34
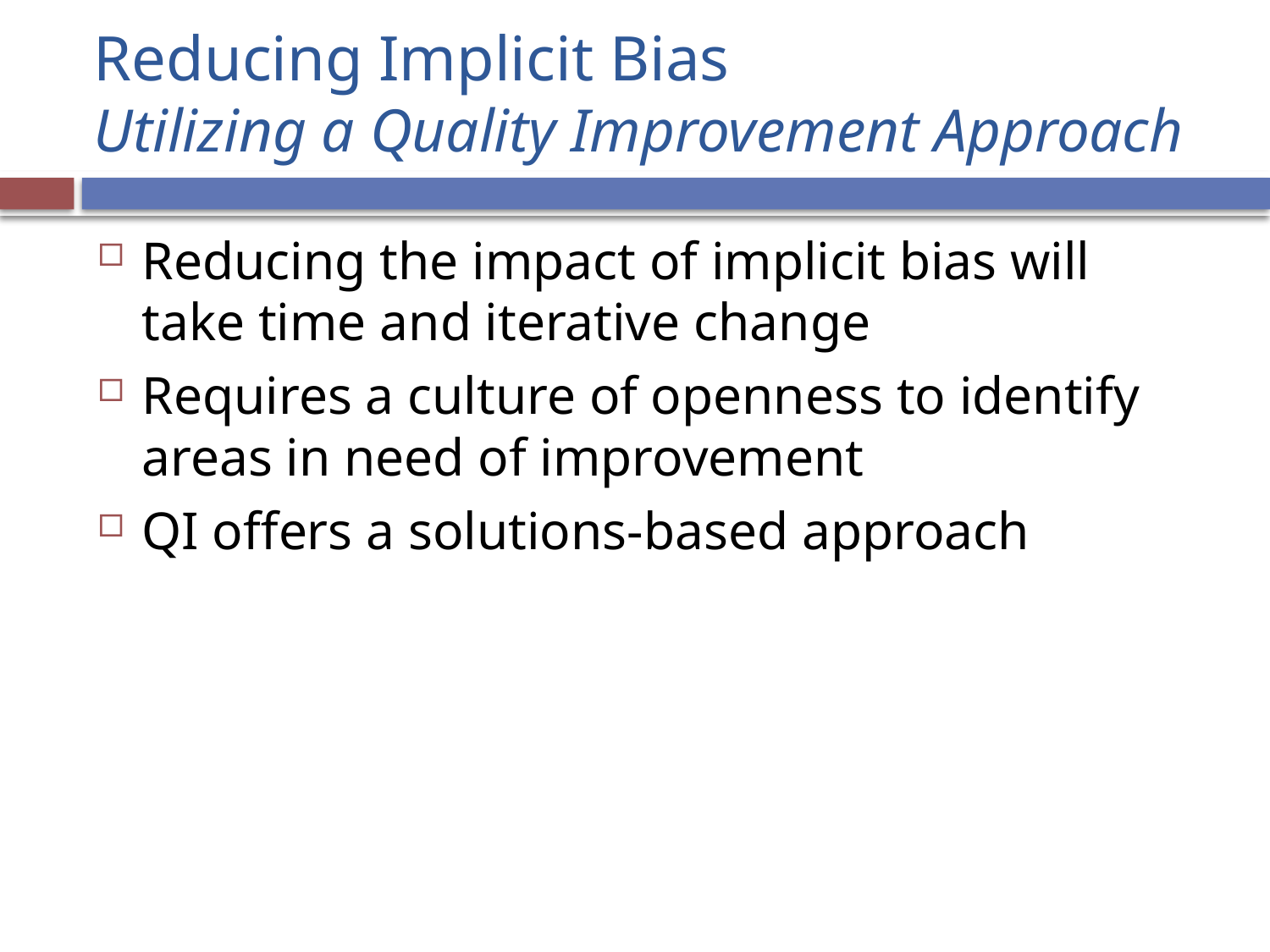

# Reducing Implicit Bias Utilizing a Quality Improvement Approach
Reducing the impact of implicit bias will take time and iterative change
Requires a culture of openness to identify areas in need of improvement
QI offers a solutions-based approach

## Slide 35
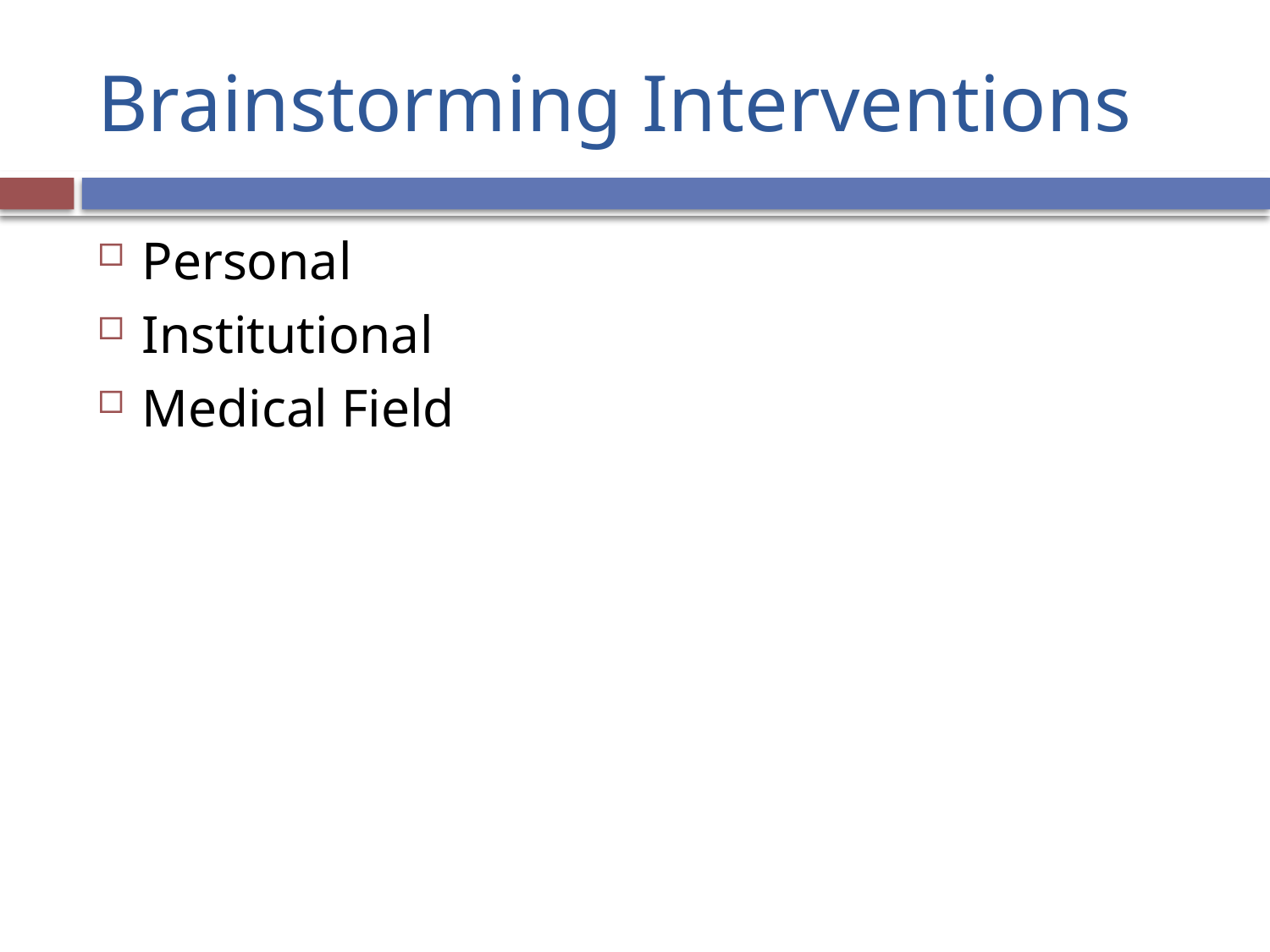

# Brainstorming Interventions
Personal
Institutional
Medical Field

## Slide 36
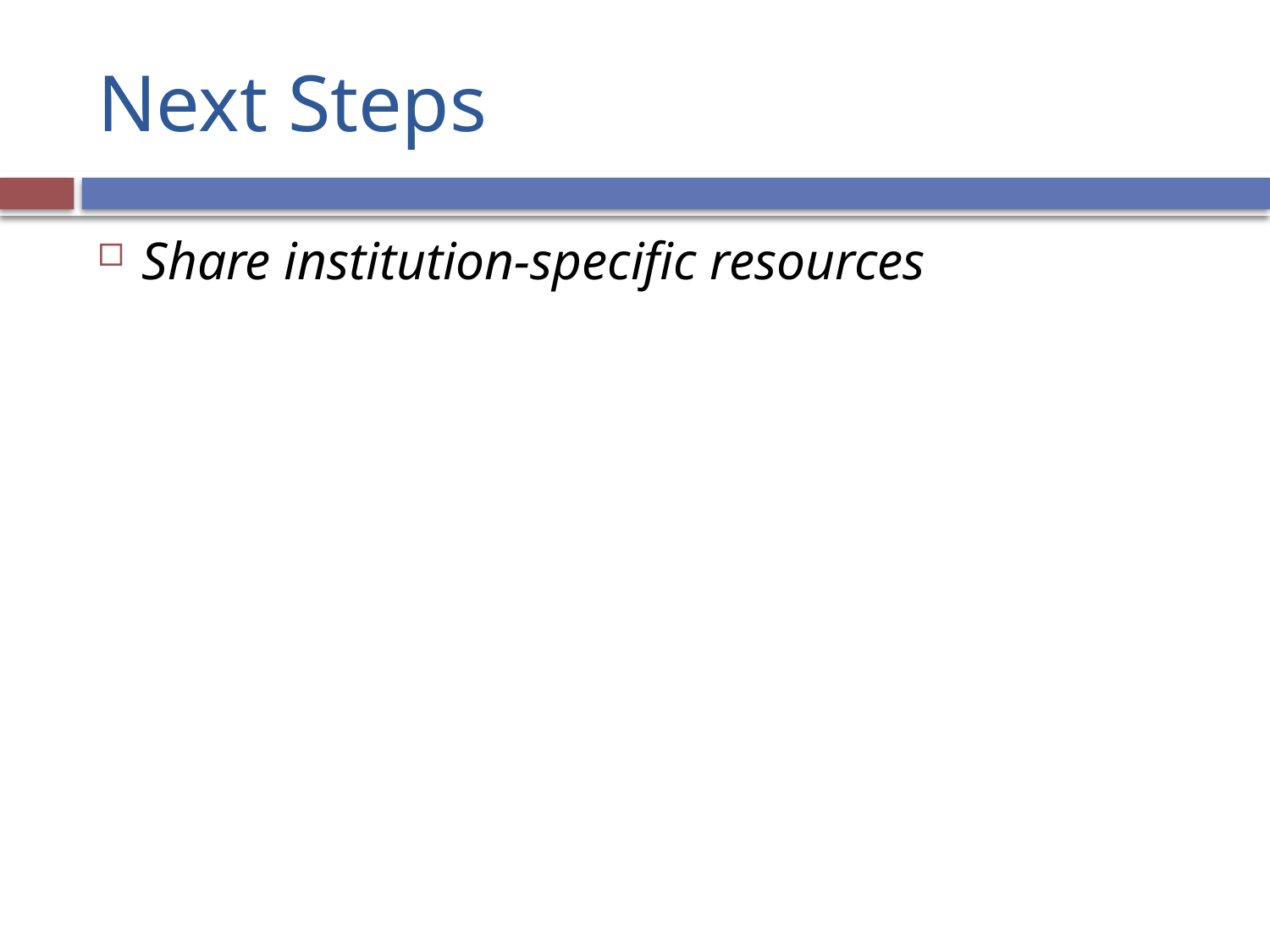

# Next Steps
Share institution-specific resources

## Slide 37
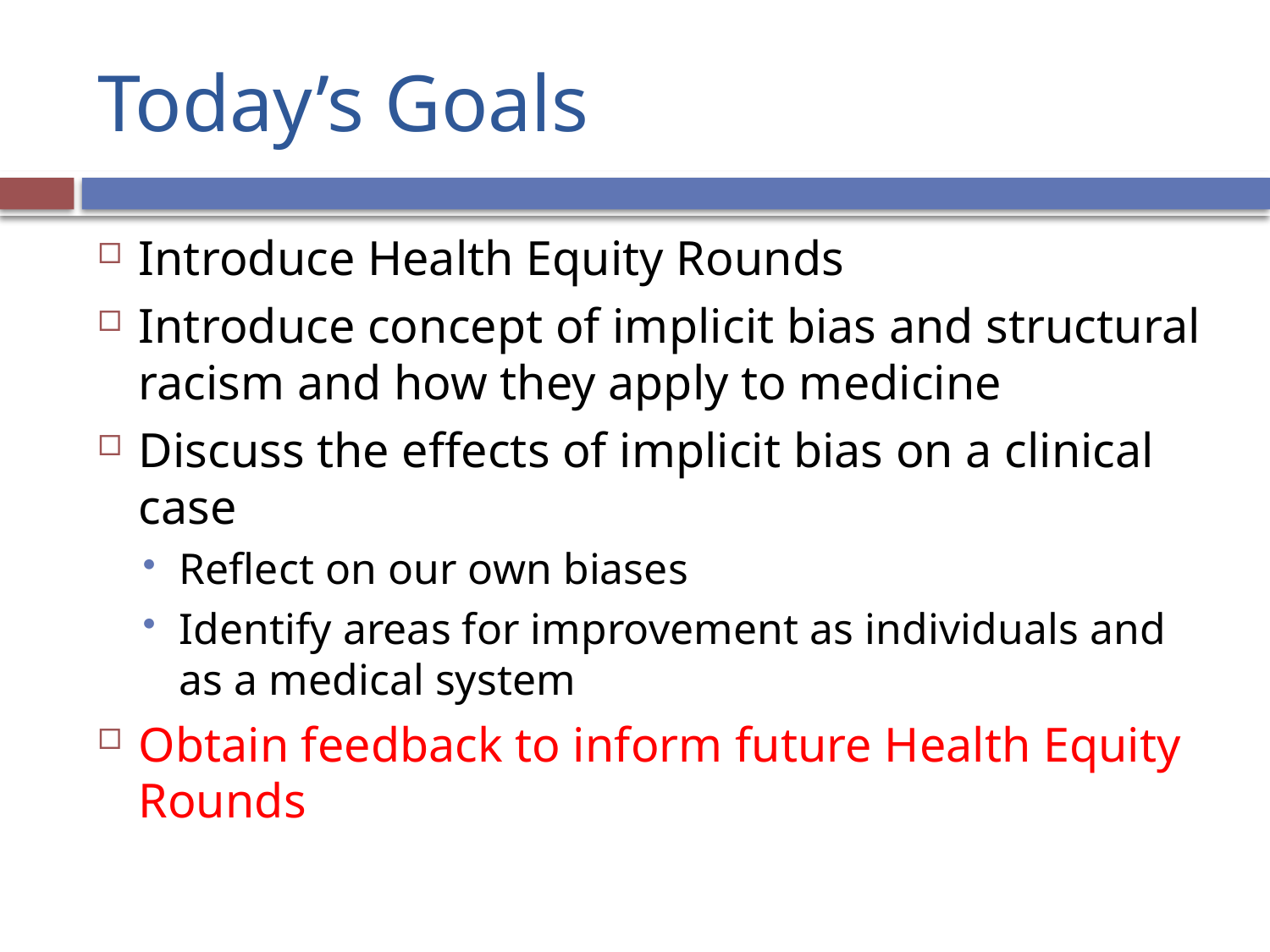

# Today’s Goals
Introduce Health Equity Rounds
Introduce concept of implicit bias and structural racism and how they apply to medicine
Discuss the effects of implicit bias on a clinical case
Reflect on our own biases
Identify areas for improvement as individuals and as a medical system
Obtain feedback to inform future Health Equity Rounds

## Slide 38
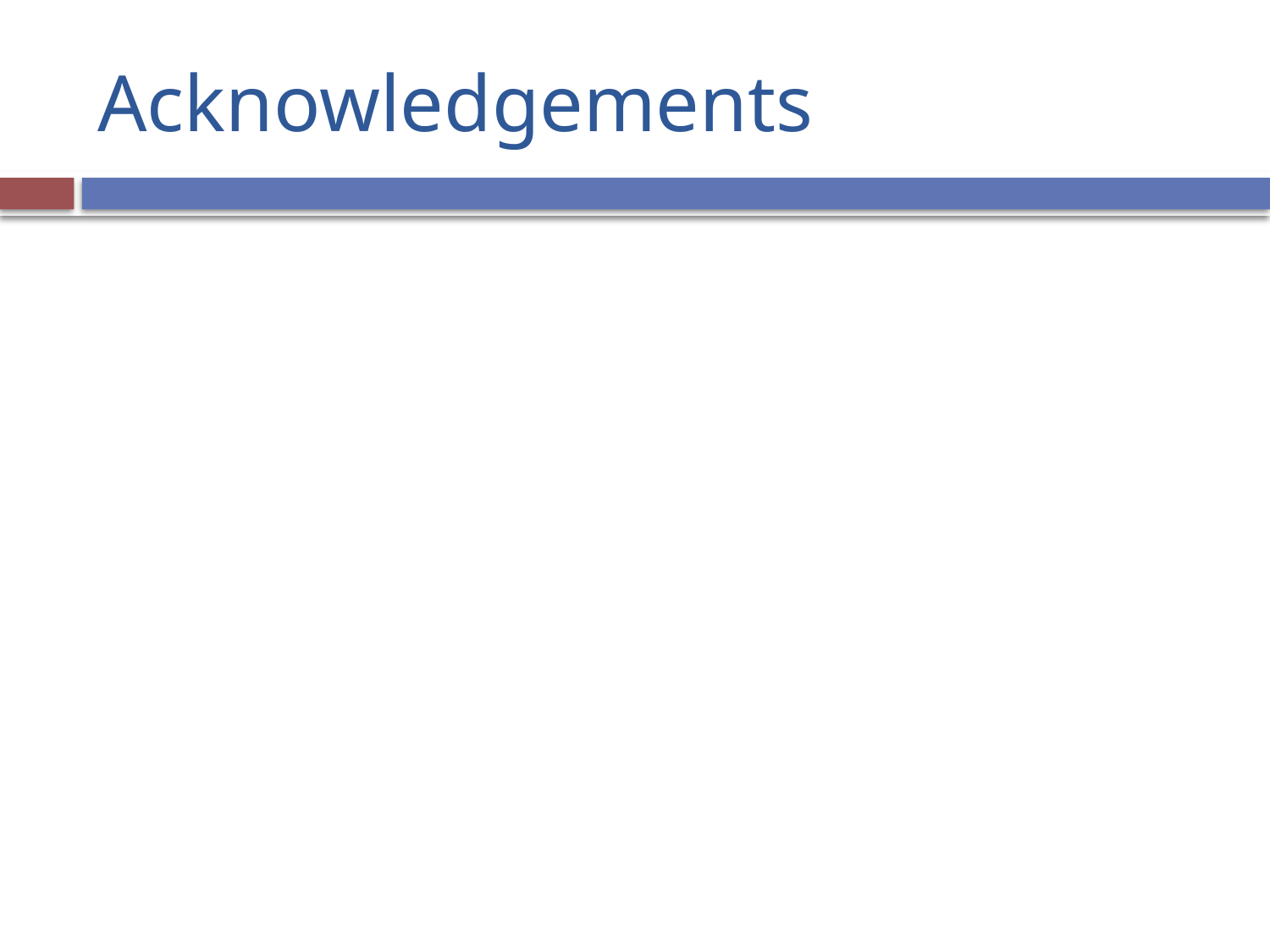

# Acknowledgements

## Slide 39
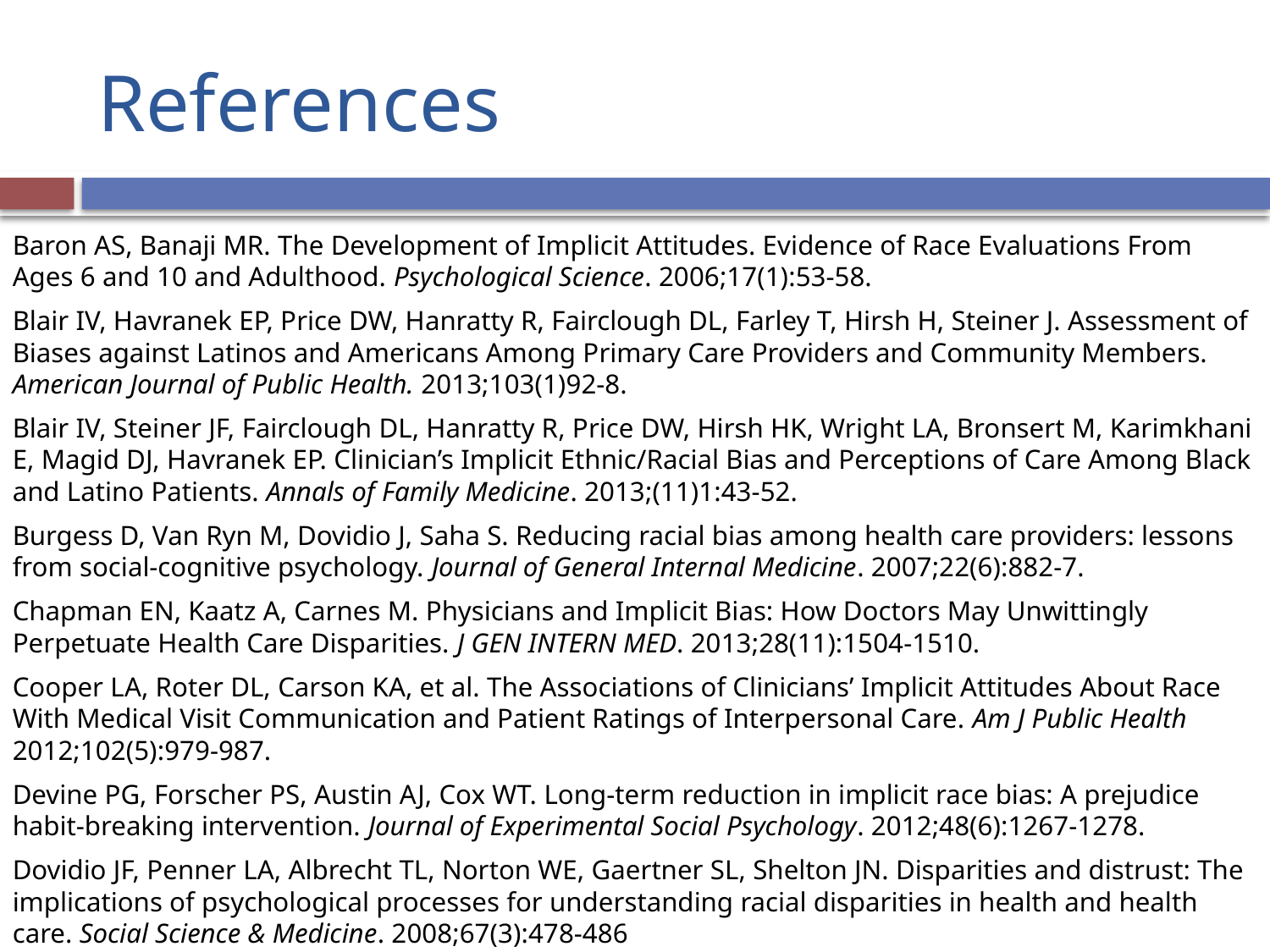

# References
Baron AS, Banaji MR. The Development of Implicit Attitudes. Evidence of Race Evaluations From Ages 6 and 10 and Adulthood. Psychological Science. 2006;17(1):53-58.
Blair IV, Havranek EP, Price DW, Hanratty R, Fairclough DL, Farley T, Hirsh H, Steiner J. Assessment of Biases against Latinos and Americans Among Primary Care Providers and Community Members. American Journal of Public Health. 2013;103(1)92-8.
Blair IV, Steiner JF, Fairclough DL, Hanratty R, Price DW, Hirsh HK, Wright LA, Bronsert M, Karimkhani E, Magid DJ, Havranek EP. Clinician’s Implicit Ethnic/Racial Bias and Perceptions of Care Among Black and Latino Patients. Annals of Family Medicine. 2013;(11)1:43-52.
Burgess D, Van Ryn M, Dovidio J, Saha S. Reducing racial bias among health care providers: lessons from social-cognitive psychology. Journal of General Internal Medicine. 2007;22(6):882-7.
Chapman EN, Kaatz A, Carnes M. Physicians and Implicit Bias: How Doctors May Unwittingly Perpetuate Health Care Disparities. J GEN INTERN MED. 2013;28(11):1504-1510.
Cooper LA, Roter DL, Carson KA, et al. The Associations of Clinicians’ Implicit Attitudes About Race With Medical Visit Communication and Patient Ratings of Interpersonal Care. Am J Public Health 2012;102(5):979-987.
Devine PG, Forscher PS, Austin AJ, Cox WT. Long-term reduction in implicit race bias: A prejudice habit-breaking intervention. Journal of Experimental Social Psychology. 2012;48(6):1267-1278.
Dovidio JF, Penner LA, Albrecht TL, Norton WE, Gaertner SL, Shelton JN. Disparities and distrust: The implications of psychological processes for understanding racial disparities in health and health care. Social Science & Medicine. 2008;67(3):478-486

## Slide 40
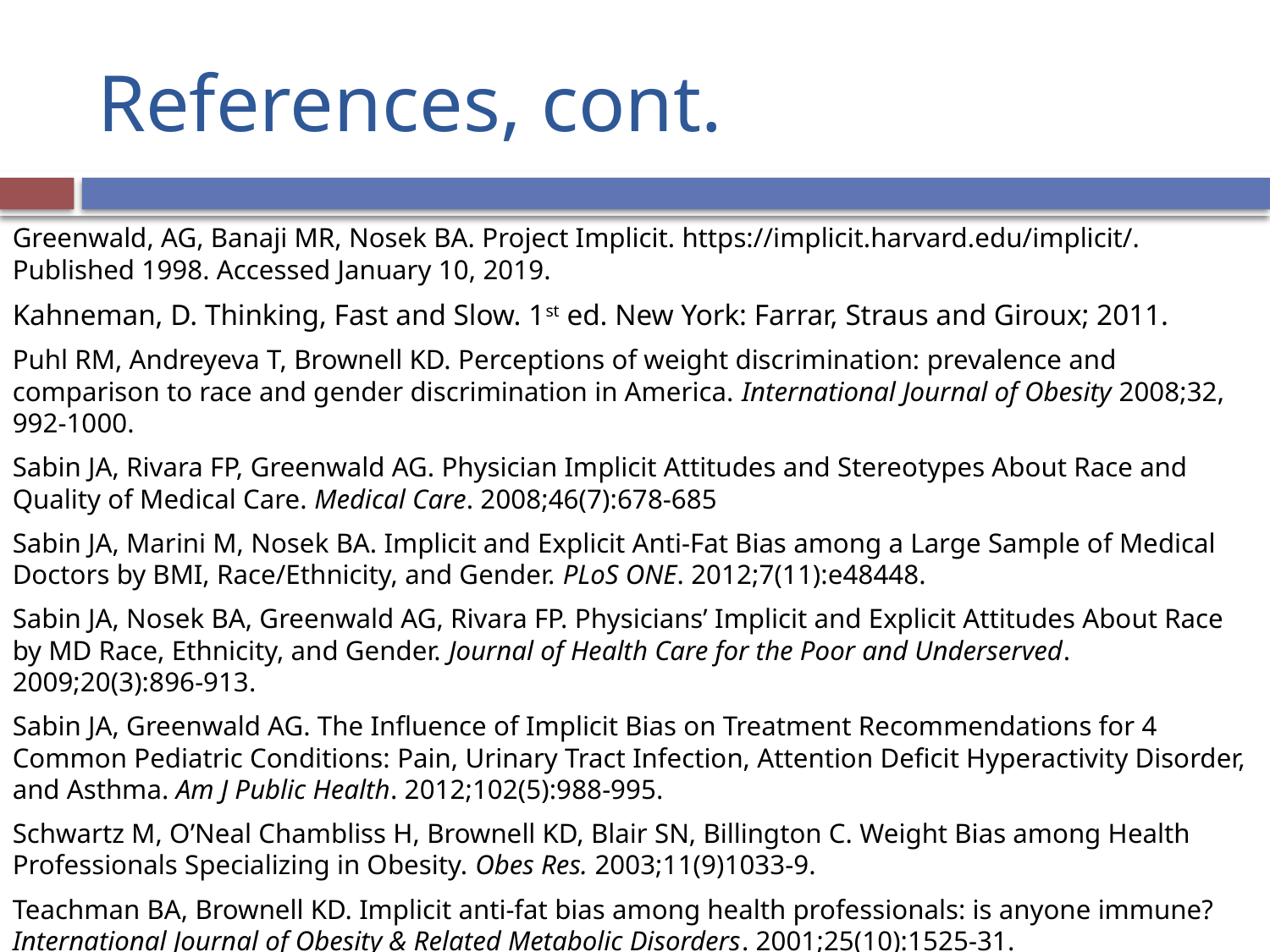

# References, cont.
Greenwald, AG, Banaji MR, Nosek BA. Project Implicit. https://implicit.harvard.edu/implicit/. Published 1998. Accessed January 10, 2019.
Kahneman, D. Thinking, Fast and Slow. 1st ed. New York: Farrar, Straus and Giroux; 2011.
Puhl RM, Andreyeva T, Brownell KD. Perceptions of weight discrimination: prevalence and comparison to race and gender discrimination in America. International Journal of Obesity 2008;32, 992-1000.
Sabin JA, Rivara FP, Greenwald AG. Physician Implicit Attitudes and Stereotypes About Race and Quality of Medical Care. Medical Care. 2008;46(7):678-685
Sabin JA, Marini M, Nosek BA. Implicit and Explicit Anti-Fat Bias among a Large Sample of Medical Doctors by BMI, Race/Ethnicity, and Gender. PLoS ONE. 2012;7(11):e48448.
Sabin JA, Nosek BA, Greenwald AG, Rivara FP. Physicians’ Implicit and Explicit Attitudes About Race by MD Race, Ethnicity, and Gender. Journal of Health Care for the Poor and Underserved. 2009;20(3):896-913.
Sabin JA, Greenwald AG. The Influence of Implicit Bias on Treatment Recommendations for 4 Common Pediatric Conditions: Pain, Urinary Tract Infection, Attention Deficit Hyperactivity Disorder, and Asthma. Am J Public Health. 2012;102(5):988-995.
Schwartz M, O’Neal Chambliss H, Brownell KD, Blair SN, Billington C. Weight Bias among Health Professionals Specializing in Obesity. Obes Res. 2003;11(9)1033-9.
Teachman BA, Brownell KD. Implicit anti-fat bias among health professionals: is anyone immune? International Journal of Obesity & Related Metabolic Disorders. 2001;25(10):1525-31.
