# Supplementary material for: Health Equity Rounds: An Interdisciplinary Case Conference to Address Implicit Bias and Structural Racism for Faculty and Trainees
Source: MedEdPORTAL. 2019 Nov 22;15:10858. doi: 10.15766/mep_2374-8265.10858 (PMC7050660; doi:10.15766/mep_2374-8265.10858)
Supplement: Supplementary file 1 — A. HER 1.pptx B. HER 2.pptx C. HER 3.pptx D. HER 4.pptx E. HER 5.pptx F. HER 6.pptx G. HER 7.pptx H. Selected HER Handouts.docx I. Case Conference Creation Guide.docx J. Glossary.docx K. Evaluation.docx [file mep-15-10858-s001.zip › B. HER 2.pptx]

## Slide 1
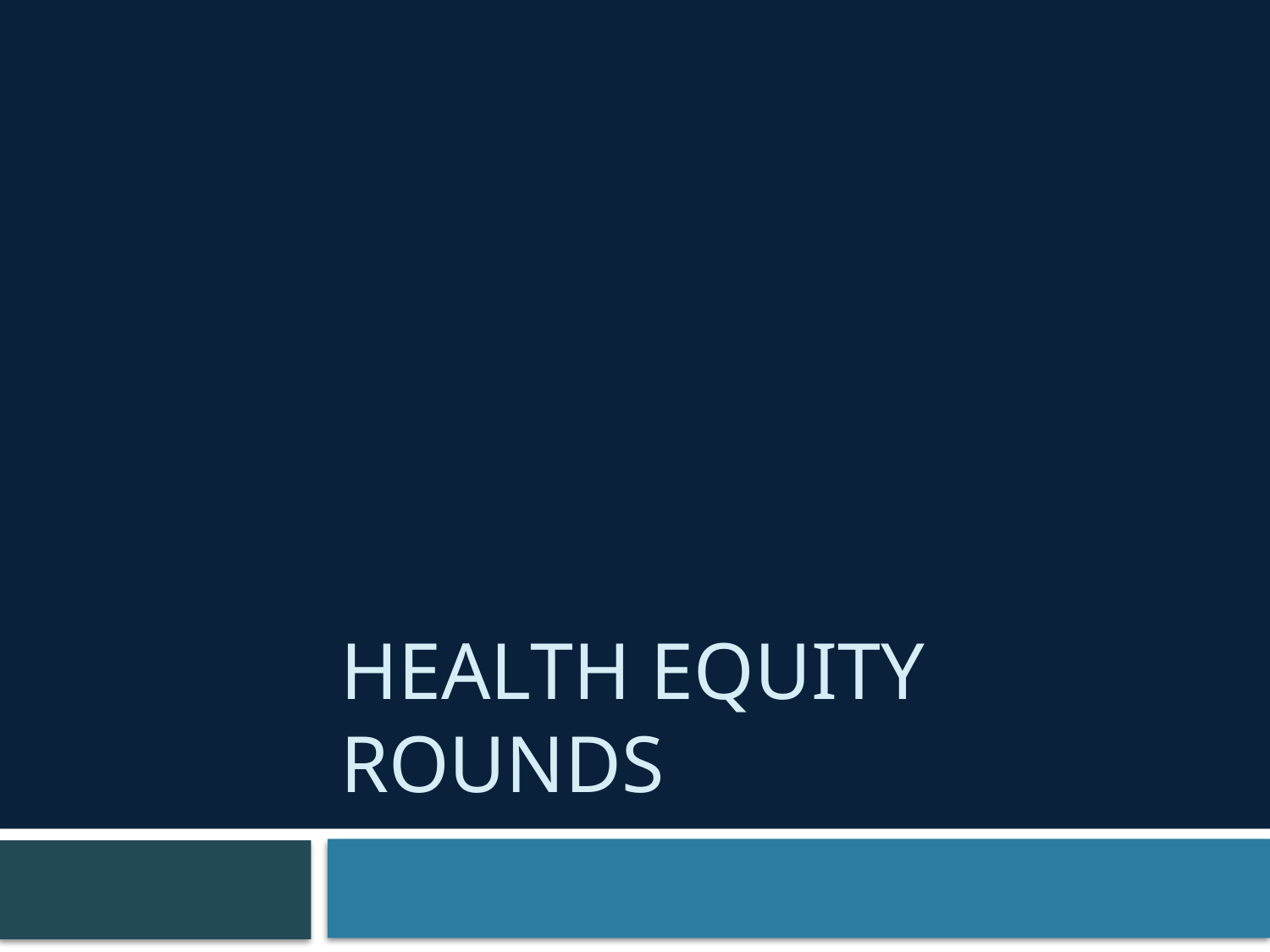

# Health equity rounds

## Slide 2
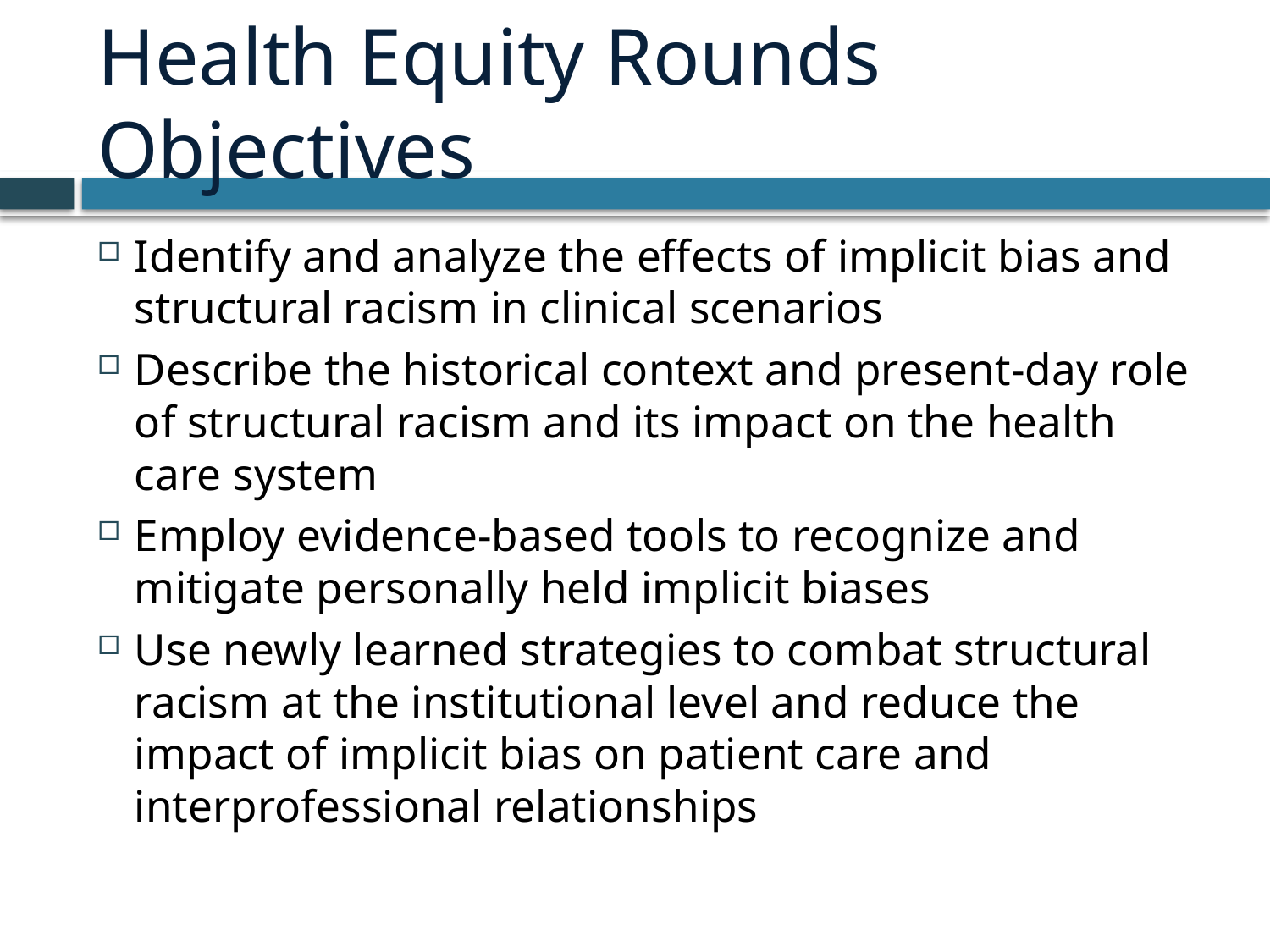

# Health Equity Rounds Objectives
Identify and analyze the effects of implicit bias and structural racism in clinical scenarios
Describe the historical context and present-day role of structural racism and its impact on the health care system
Employ evidence-based tools to recognize and mitigate personally held implicit biases
Use newly learned strategies to combat structural racism at the institutional level and reduce the impact of implicit bias on patient care and interprofessional relationships

## Slide 3
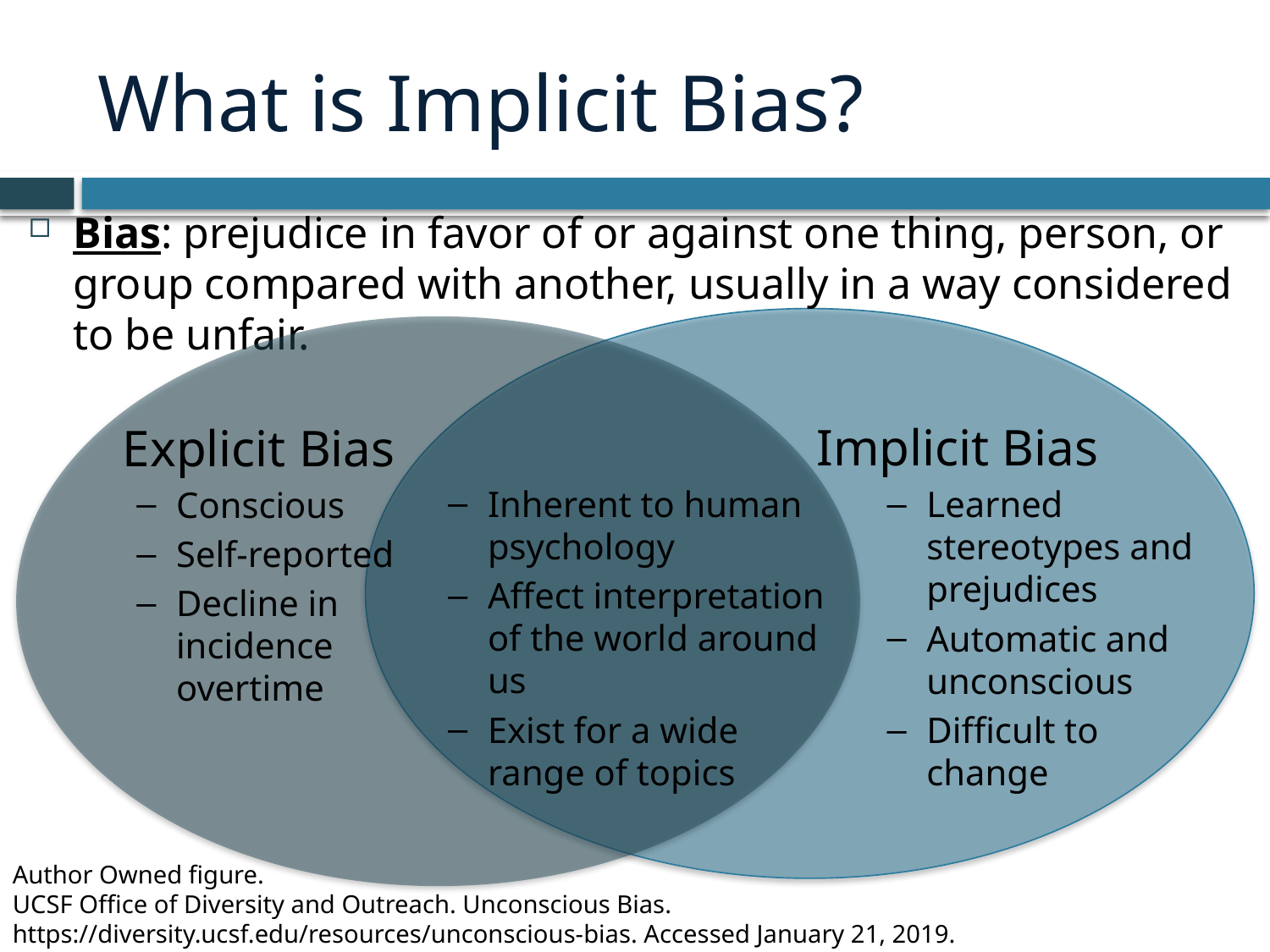

# What is Implicit Bias?
Bias: prejudice in favor of or against one thing, person, or group compared with another, usually in a way considered to be unfair.
Implicit Bias
Explicit Bias
Inherent to human psychology
Affect interpretation of the world around us
Exist for a wide range of topics
Learned stereotypes and prejudices
Automatic and unconscious
Difficult to change
Conscious
Self-reported
Decline in incidence overtime
Author Owned figure.
UCSF Office of Diversity and Outreach. Unconscious Bias. https://diversity.ucsf.edu/resources/unconscious-bias. Accessed January 21, 2019.

## Slide 4
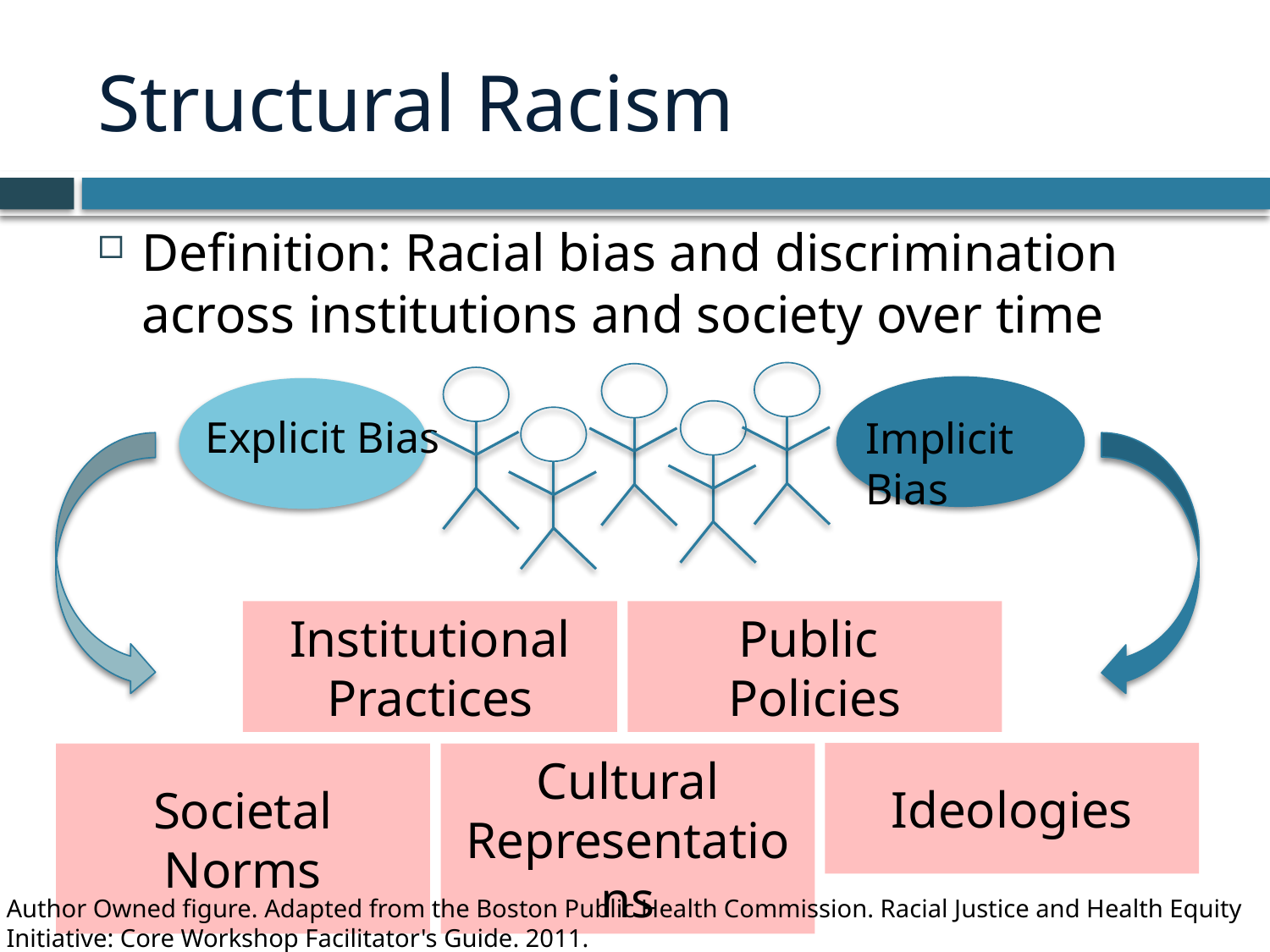

# Structural Racism
Definition: Racial bias and discrimination across institutions and society over time
Explicit Bias
Implicit Bias
Institutional Practices
Public
Policies
Ideologies
Societal Norms
Cultural
Representations
Author Owned figure. Adapted from the Boston Public Health Commission. Racial Justice and Health Equity Initiative: Core Workshop Facilitator's Guide. 2011.

## Slide 5
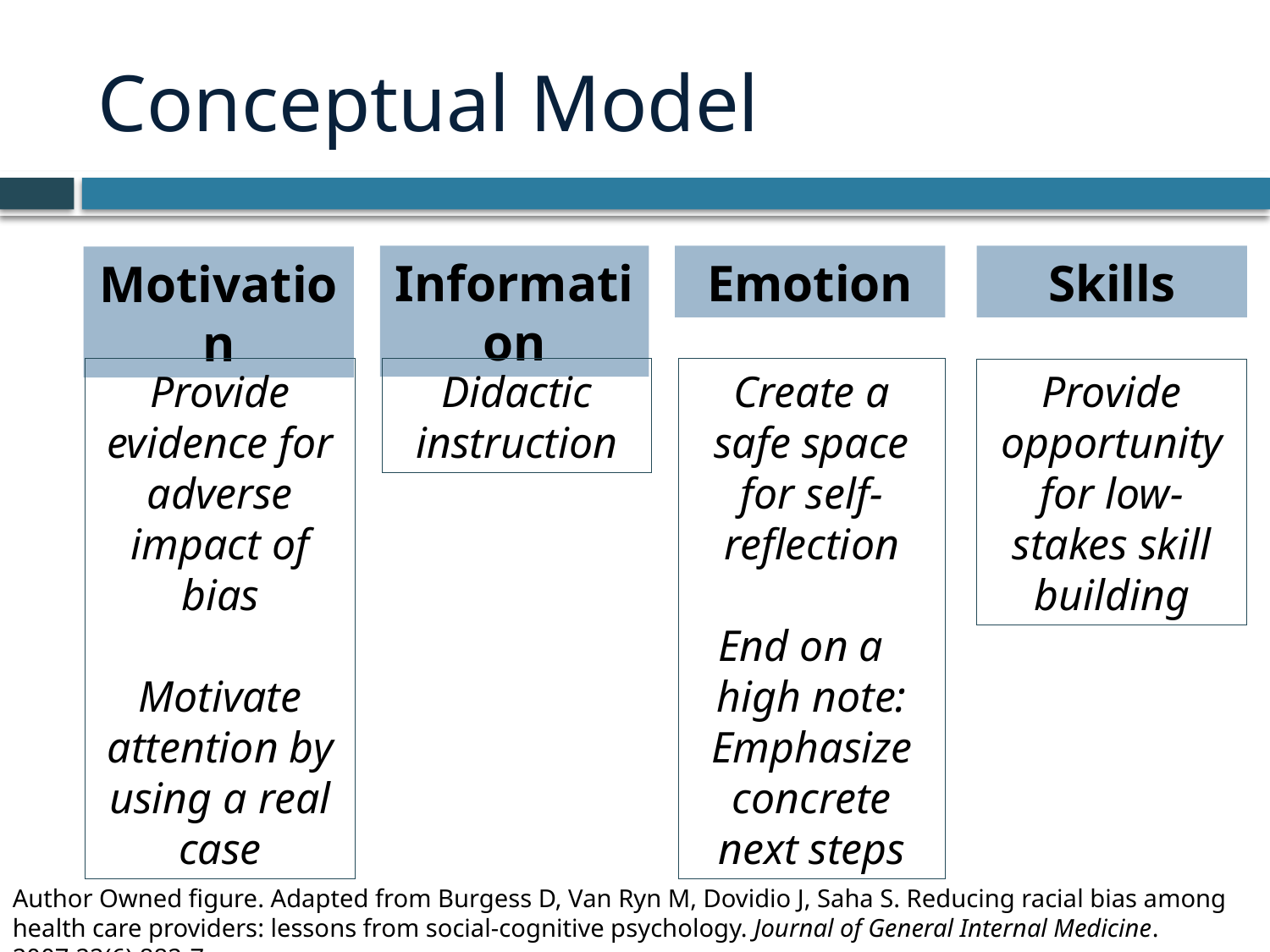

# Conceptual Model
Skills
Information
Emotion
Motivation
Create a safe space for self-reflection
d
End on a high note: Emphasize concrete next steps
Provide evidence for adverse impact of bias
Motivate attention by using a real case
Didactic instruction
Provide opportunity for low-stakes skill building
Author Owned figure. Adapted from Burgess D, Van Ryn M, Dovidio J, Saha S. Reducing racial bias among health care providers: lessons from social-cognitive psychology. Journal of General Internal Medicine. 2007;22(6):882-7.

## Slide 6
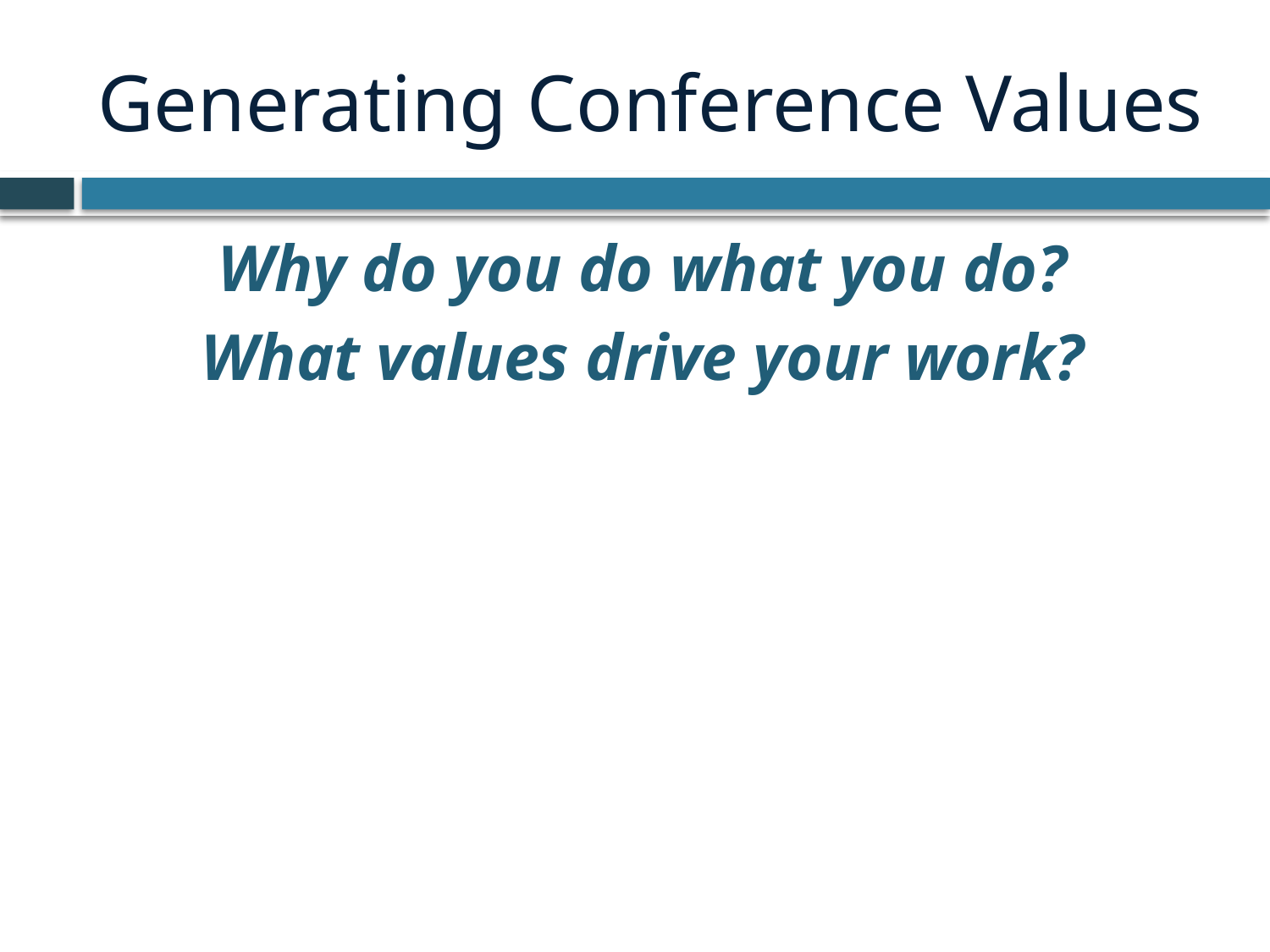

# Generating Conference Values
Why do you do what you do?
What values drive your work?

## Slide 7
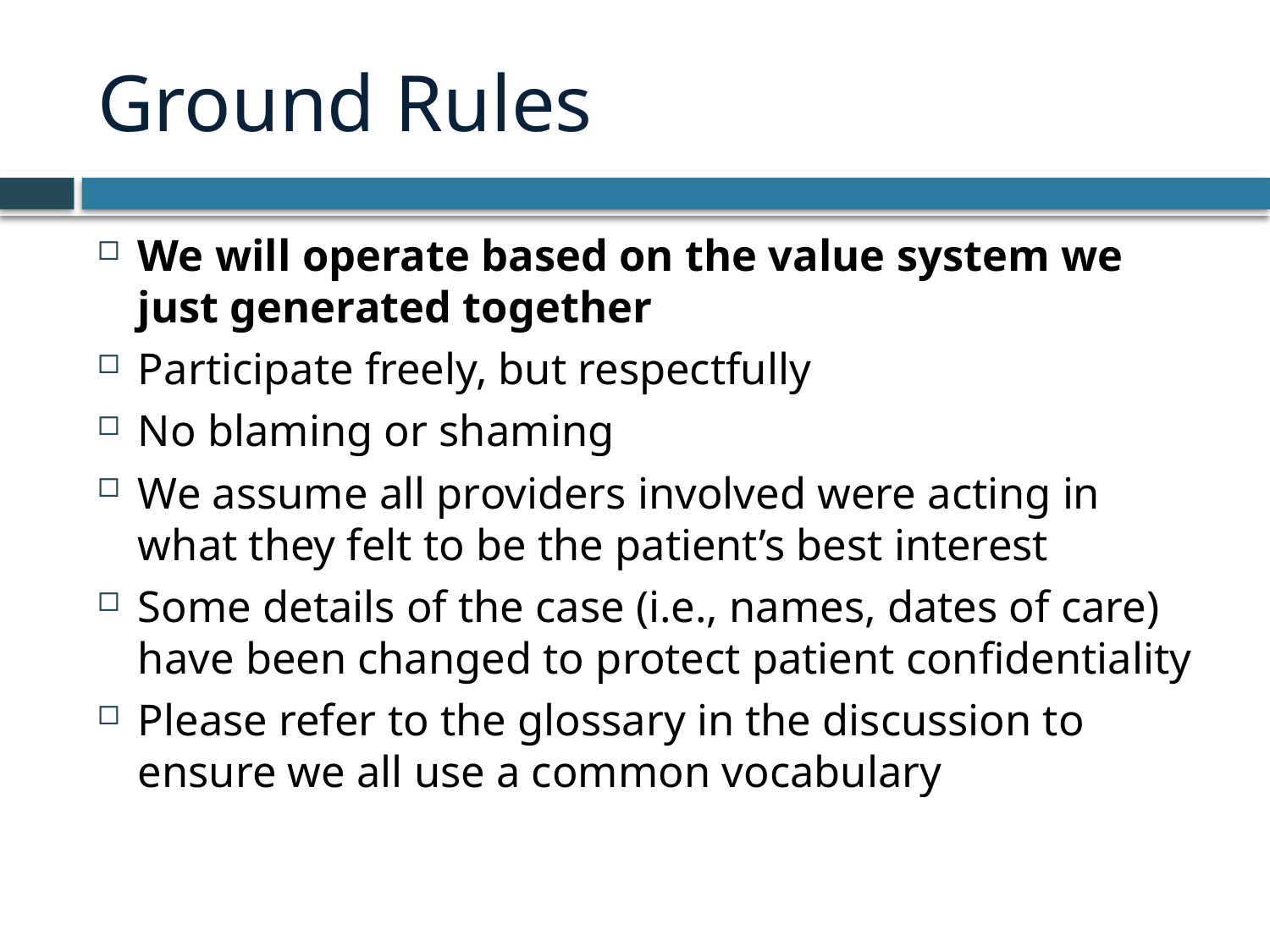

# Ground Rules
We will operate based on the value system we just generated together
Participate freely, but respectfully
No blaming or shaming
We assume all providers involved were acting in what they felt to be the patient’s best interest
Some details of the case (i.e., names, dates of care) have been changed to protect patient confidentiality
Please refer to the glossary in the discussion to ensure we all use a common vocabulary

## Slide 8
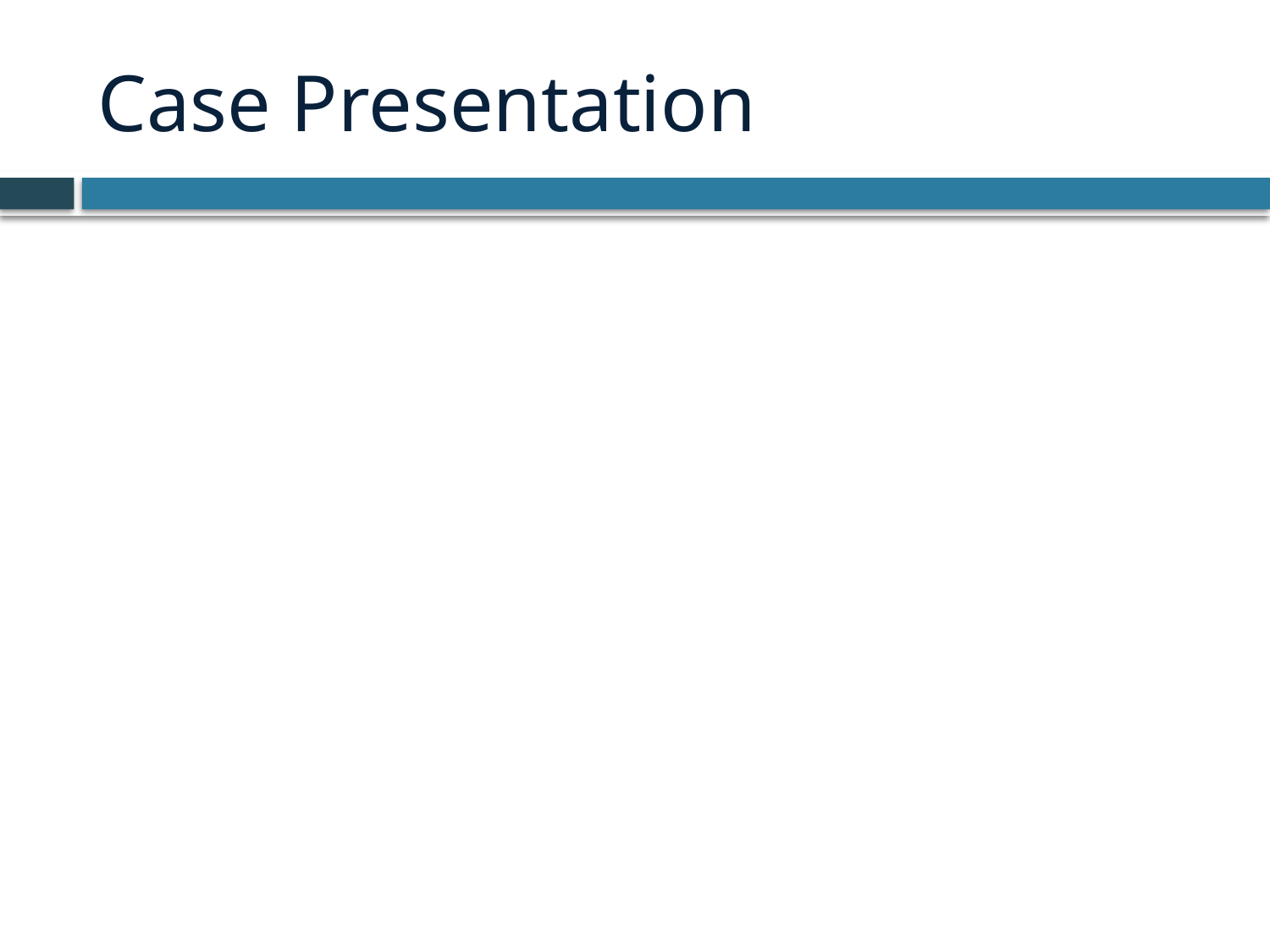

# Case Presentation

## Slide 9
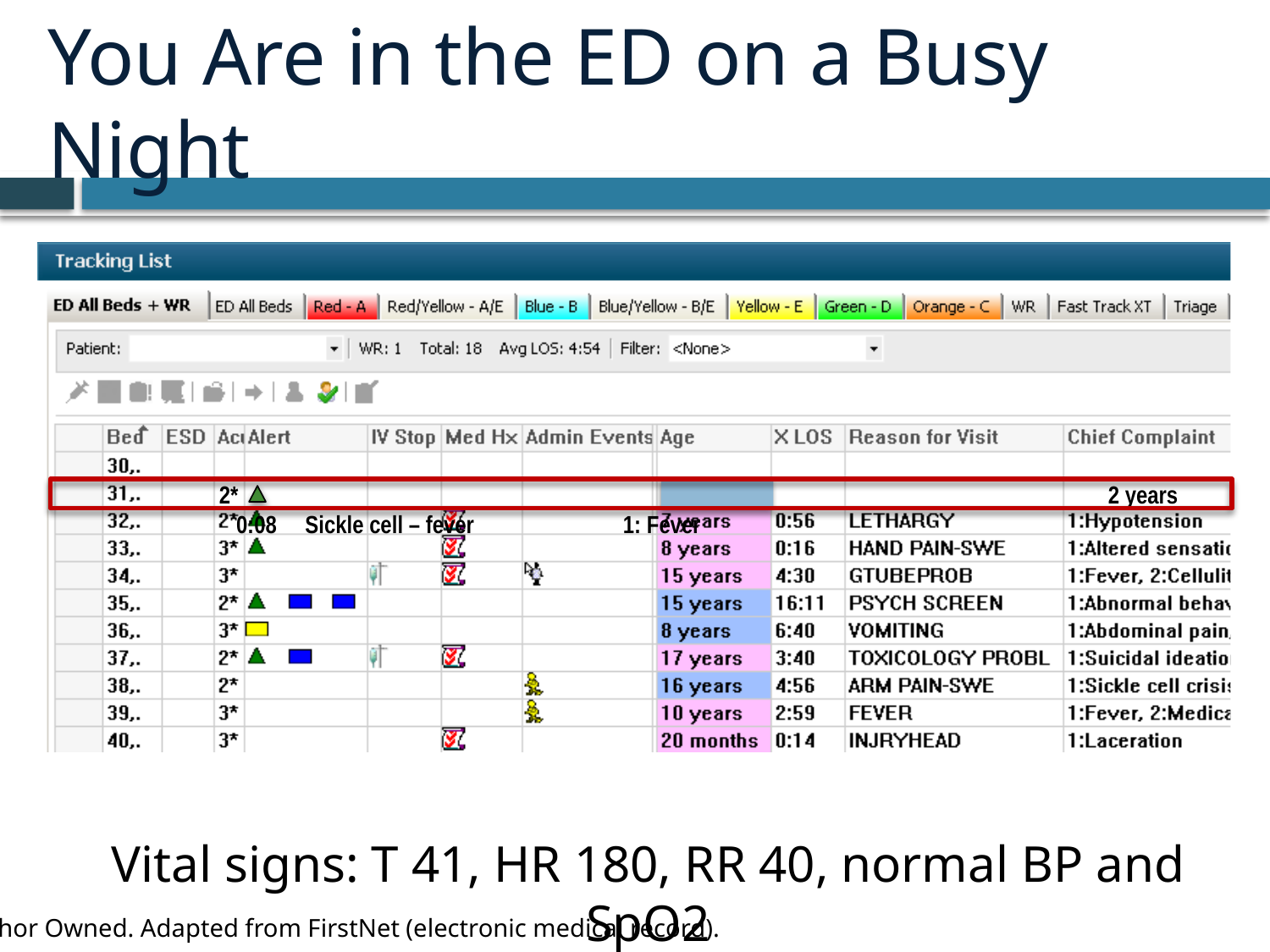

# You Are in the ED on a Busy Night
2*							2 years 0:08 Sickle cell – fever 	 1: Fever
Vital signs: T 41, HR 180, RR 40, normal BP and SpO2
Author Owned. Adapted from FirstNet (electronic medical record).

## Slide 10
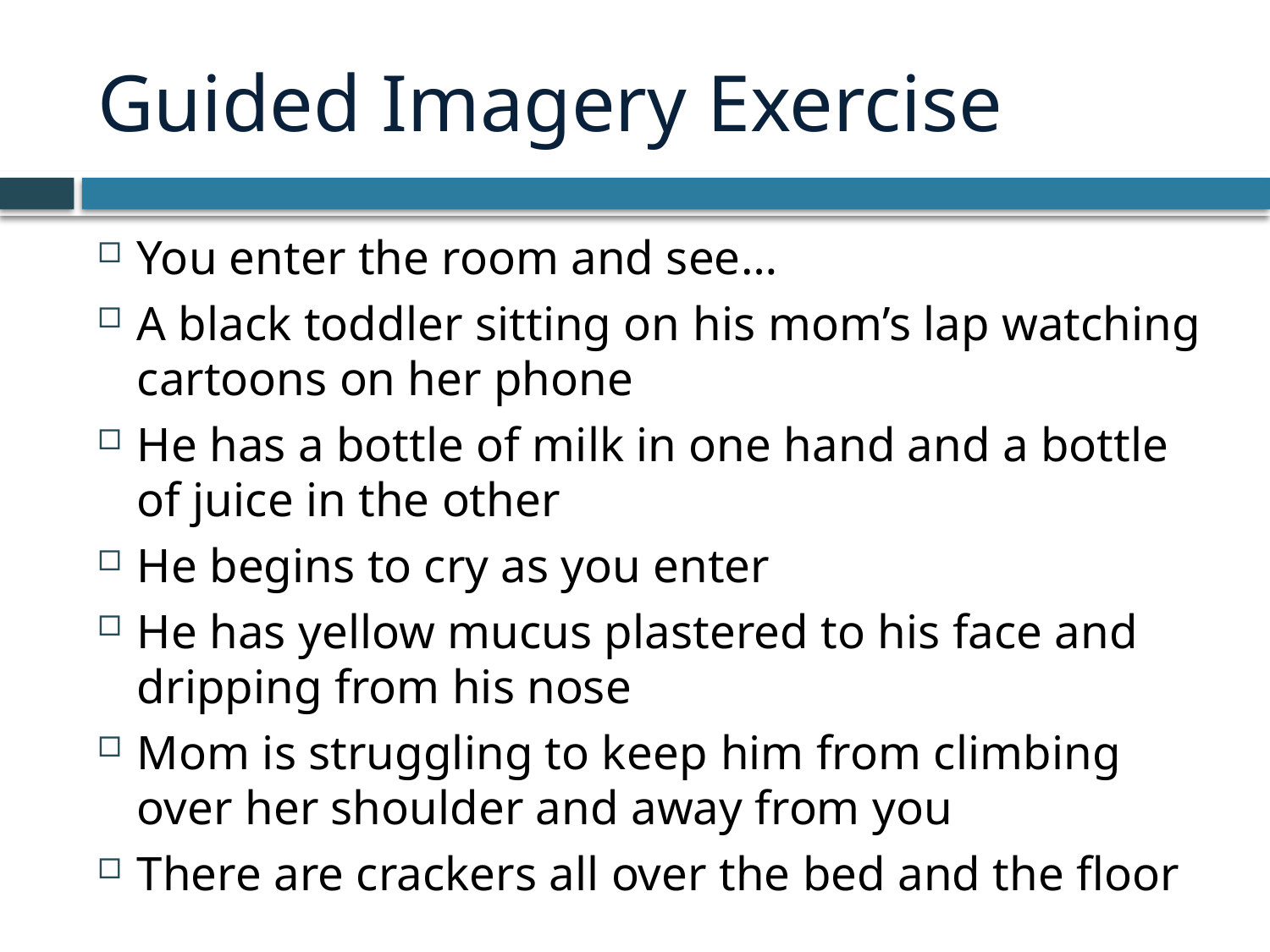

# Guided Imagery Exercise
You enter the room and see…
A black toddler sitting on his mom’s lap watching cartoons on her phone
He has a bottle of milk in one hand and a bottle of juice in the other
He begins to cry as you enter
He has yellow mucus plastered to his face and dripping from his nose
Mom is struggling to keep him from climbing over her shoulder and away from you
There are crackers all over the bed and the floor

## Slide 11
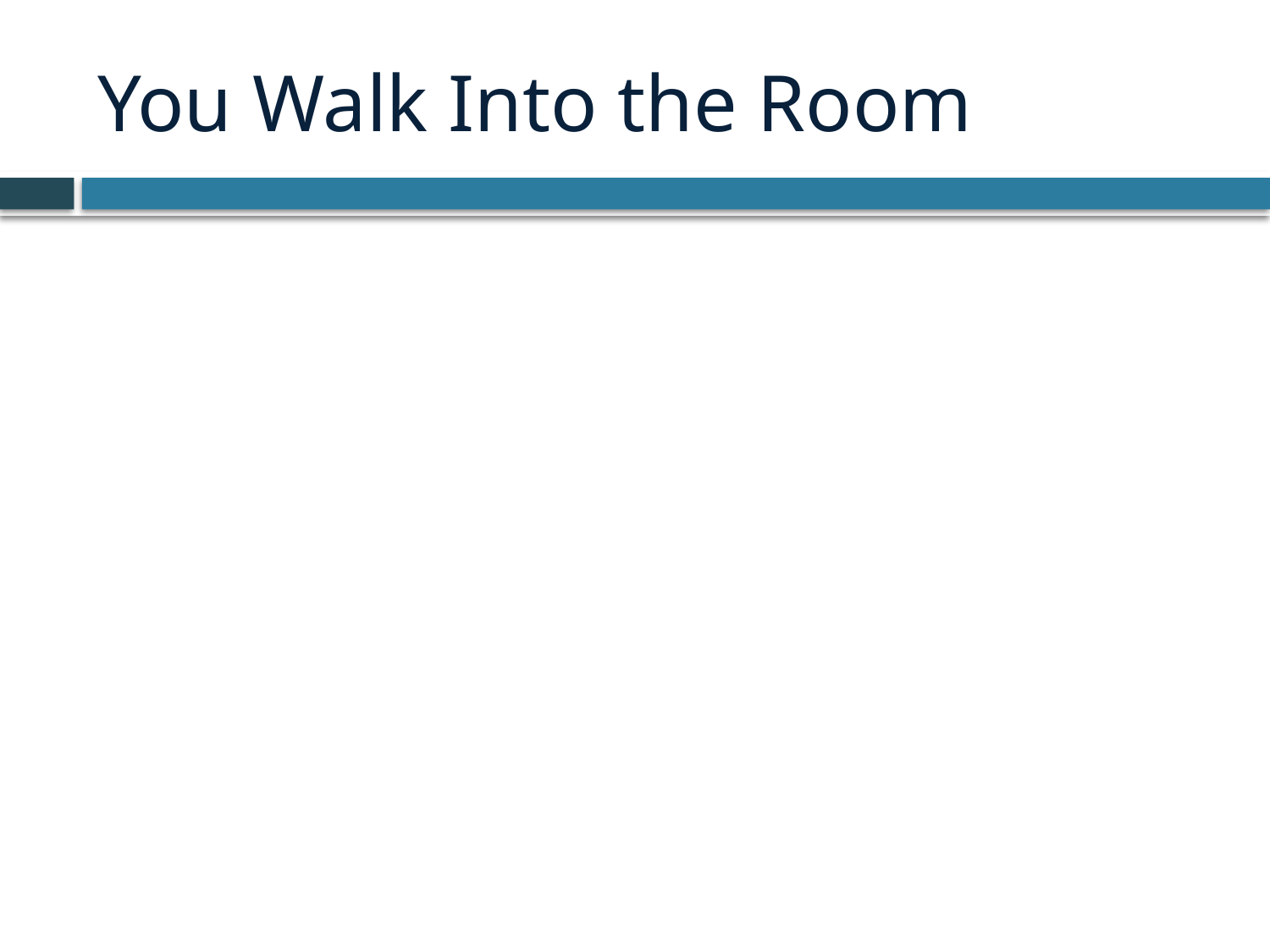

# You Walk Into the Room

## Slide 12
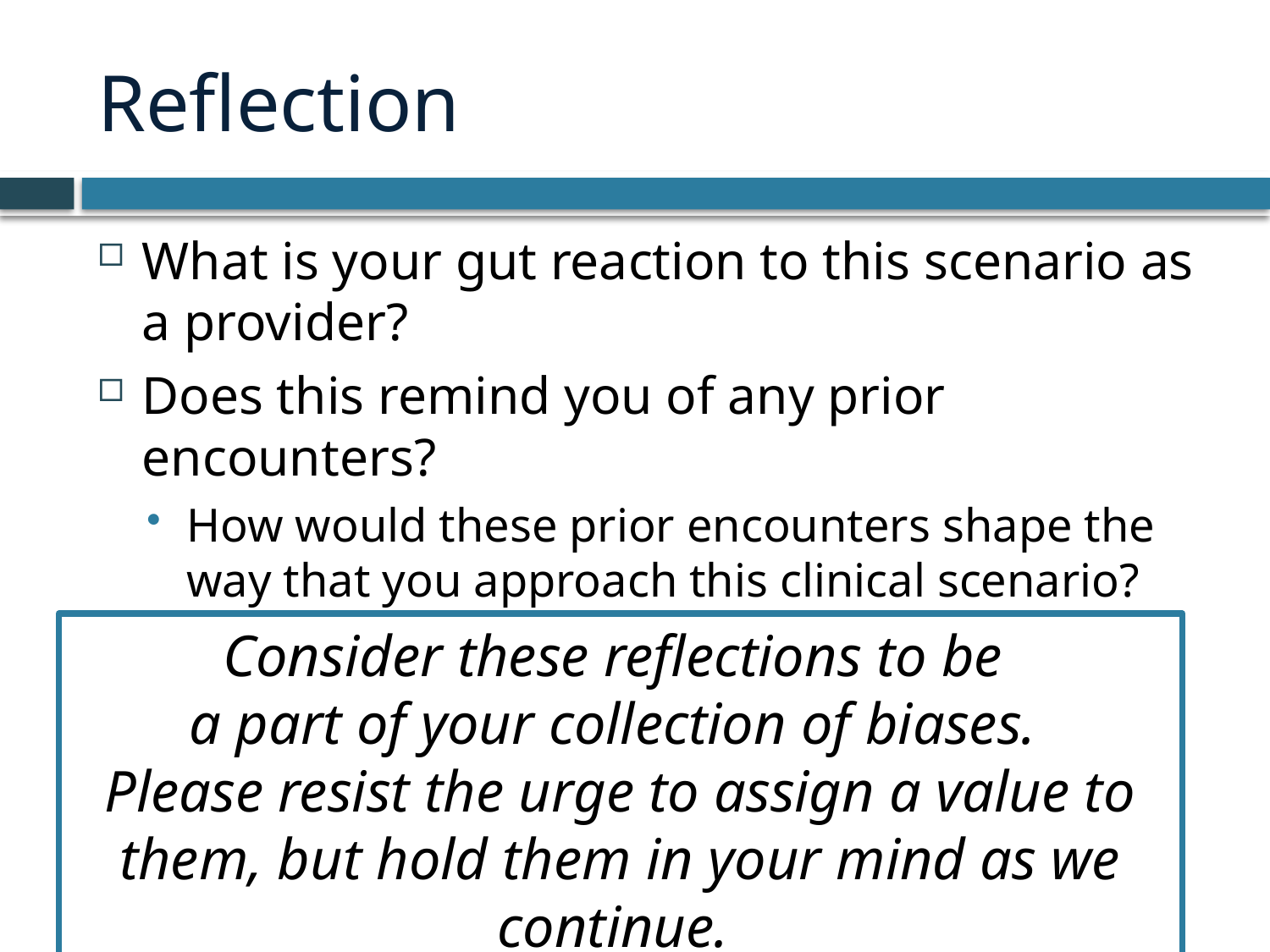

# Reflection
What is your gut reaction to this scenario as a provider?
Does this remind you of any prior encounters?
How would these prior encounters shape the way that you approach this clinical scenario?
Consider these reflections to be
a part of your collection of biases.
Please resist the urge to assign a value to them, but hold them in your mind as we continue.

## Slide 13
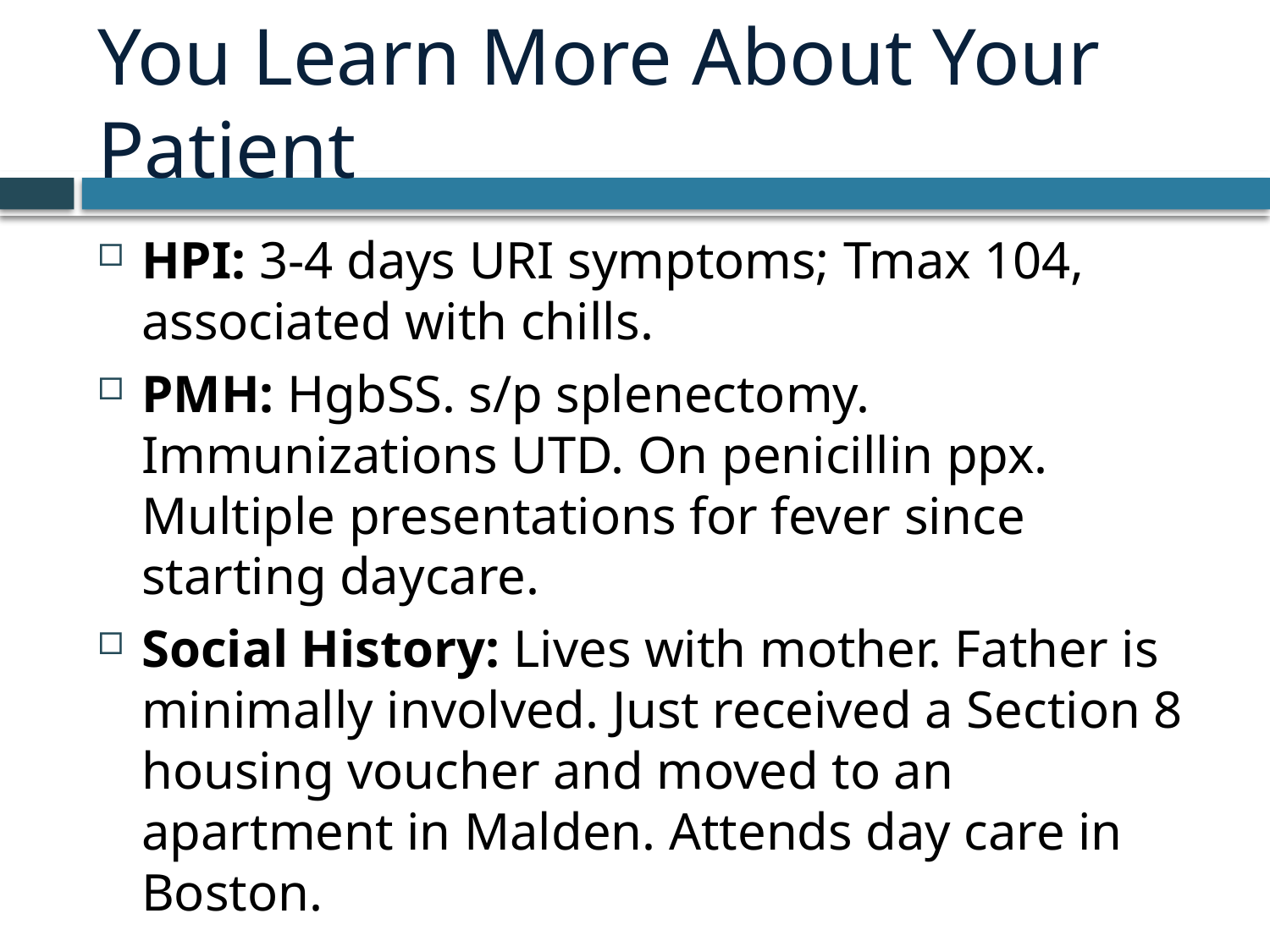

# You Learn More About Your Patient
HPI: 3-4 days URI symptoms; Tmax 104, associated with chills.
PMH: HgbSS. s/p splenectomy. Immunizations UTD. On penicillin ppx. Multiple presentations for fever since starting daycare.
Social History: Lives with mother. Father is minimally involved. Just received a Section 8 housing voucher and moved to an apartment in Malden. Attends day care in Boston.

## Slide 14
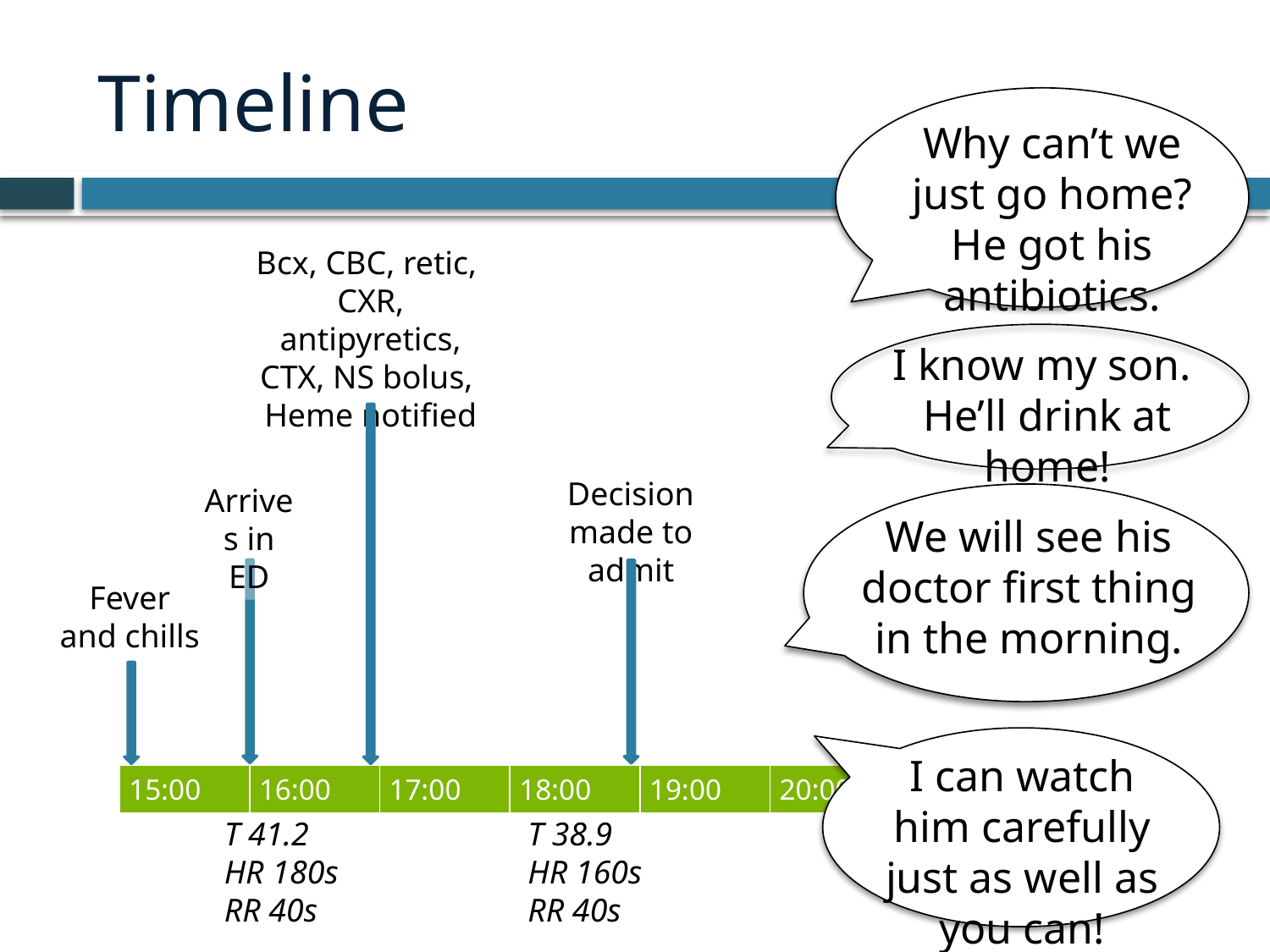

# Timeline
Why can’t we just go home? He got his antibiotics.
Bcx, CBC, retic,
CXR, antipyretics, CTX, NS bolus,
Heme notified
I know my son.
He’ll drink at home!
Decision made to admit
Arrives in ED
We will see his doctor first thing in the morning.
Fever and chills
I can watch him carefully just as well as you can!
| 15:00 | 16:00 | 17:00 | 18:00 | 19:00 | 20:00 | 21:00 | 22:00 |
| --- | --- | --- | --- | --- | --- | --- | --- |
T 41.2
HR 180s
RR 40s
T 38.9
HR 160s
RR 40s

## Slide 15
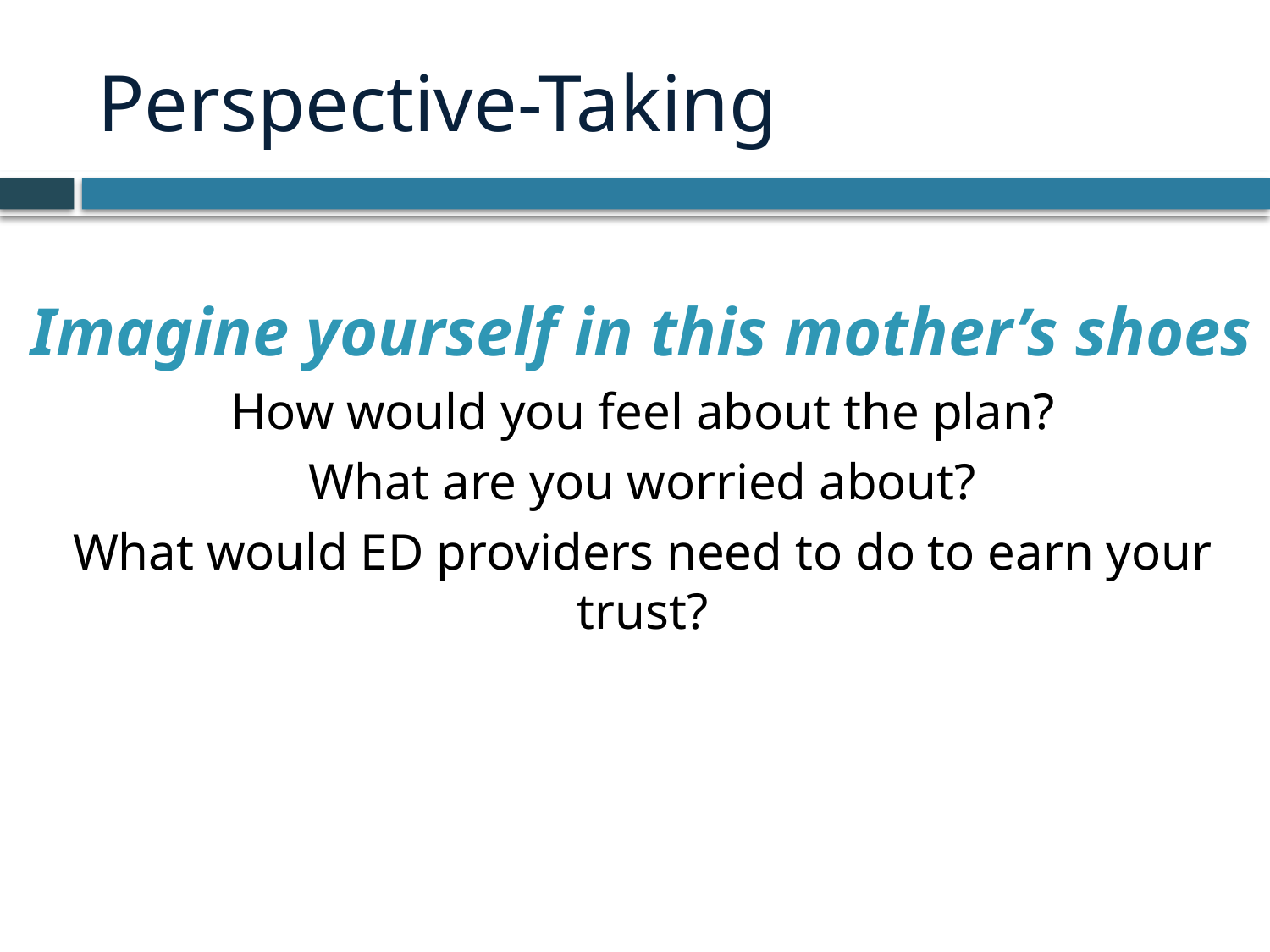

# Perspective-Taking
Imagine yourself in this mother’s shoes
How would you feel about the plan?
What are you worried about?
What would ED providers need to do to earn your trust?

## Slide 16
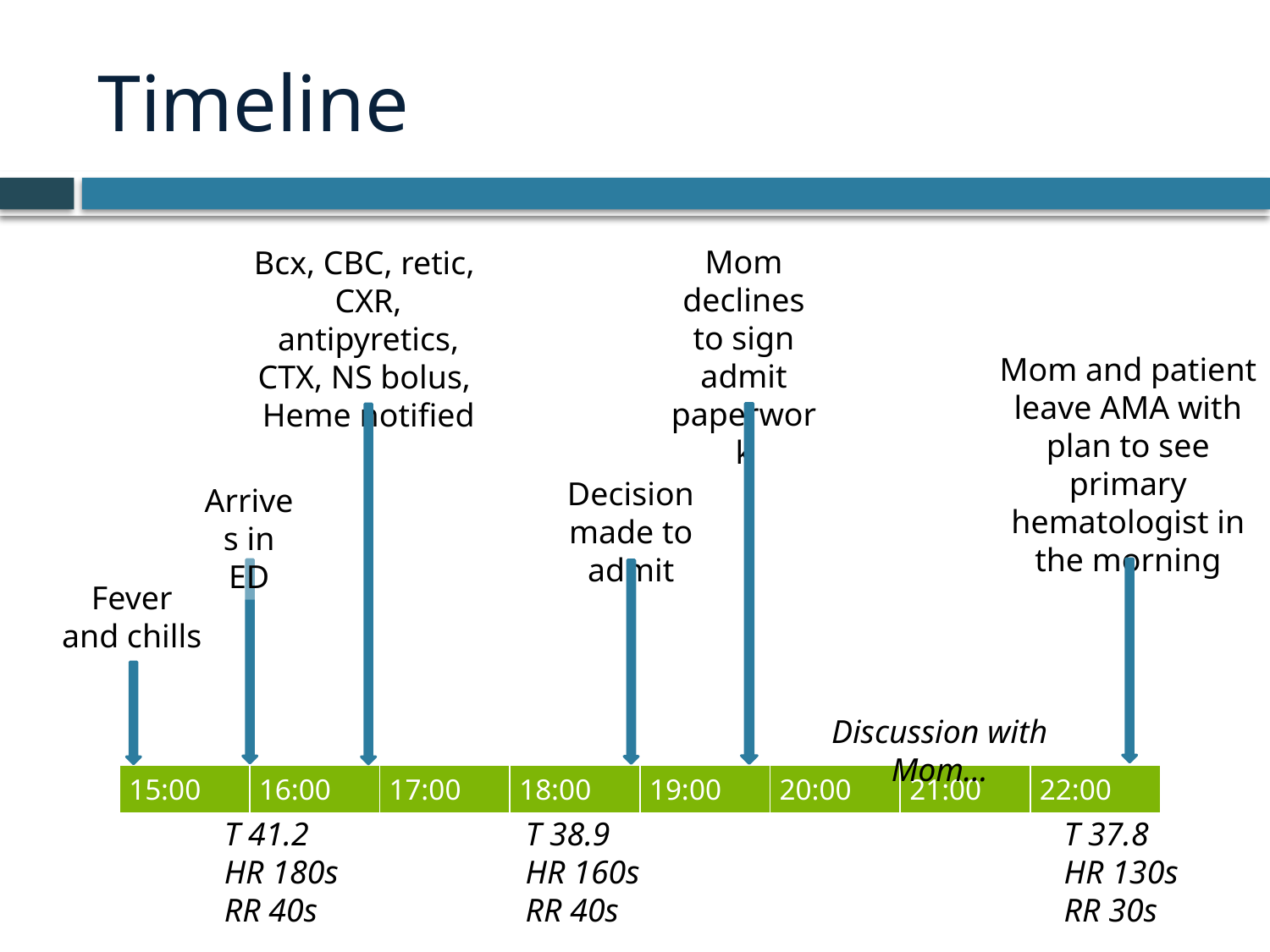

# Timeline
Mom declines to sign admit paperwork
Bcx, CBC, retic,
CXR, antipyretics, CTX, NS bolus,
Heme notified
Mom and patient leave AMA with plan to see primary hematologist in the morning
Decision made to admit
T 38.9
HR 160s
RR 40s
Arrives in ED
Fever and chills
Discussion with Mom…
| 15:00 | 16:00 | 17:00 | 18:00 | 19:00 | 20:00 | 21:00 | 22:00 |
| --- | --- | --- | --- | --- | --- | --- | --- |
T 41.2
HR 180s
RR 40s
T 37.8
HR 130s
RR 30s

## Slide 17
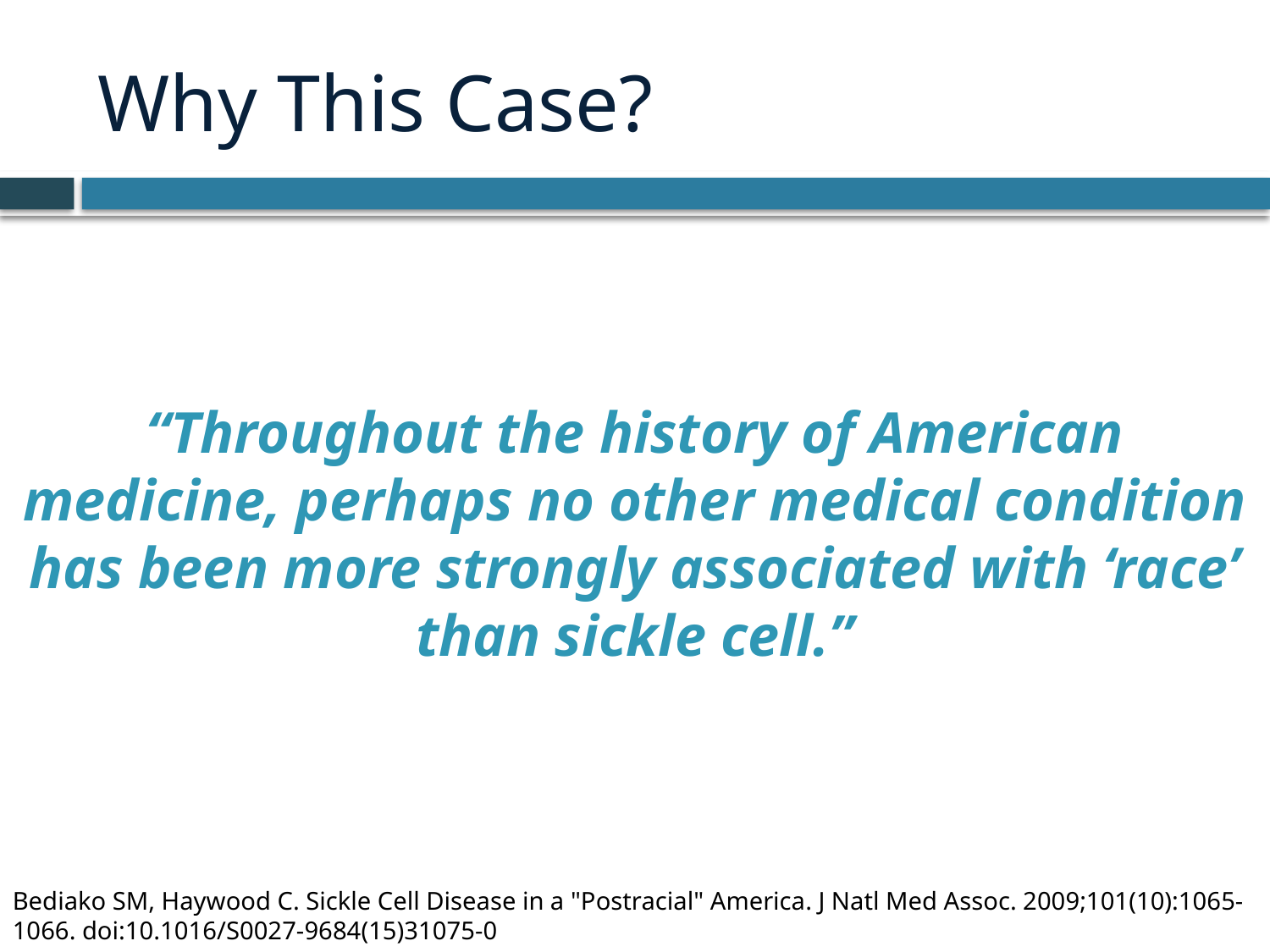

# Why This Case?
“Throughout the history of American medicine, perhaps no other medical condition has been more strongly associated with ‘race’ than sickle cell.”
Bediako SM, Haywood C. Sickle Cell Disease in a "Postracial" America. J Natl Med Assoc. 2009;101(10):1065-1066. doi:10.1016/S0027-9684(15)31075-0

## Slide 18
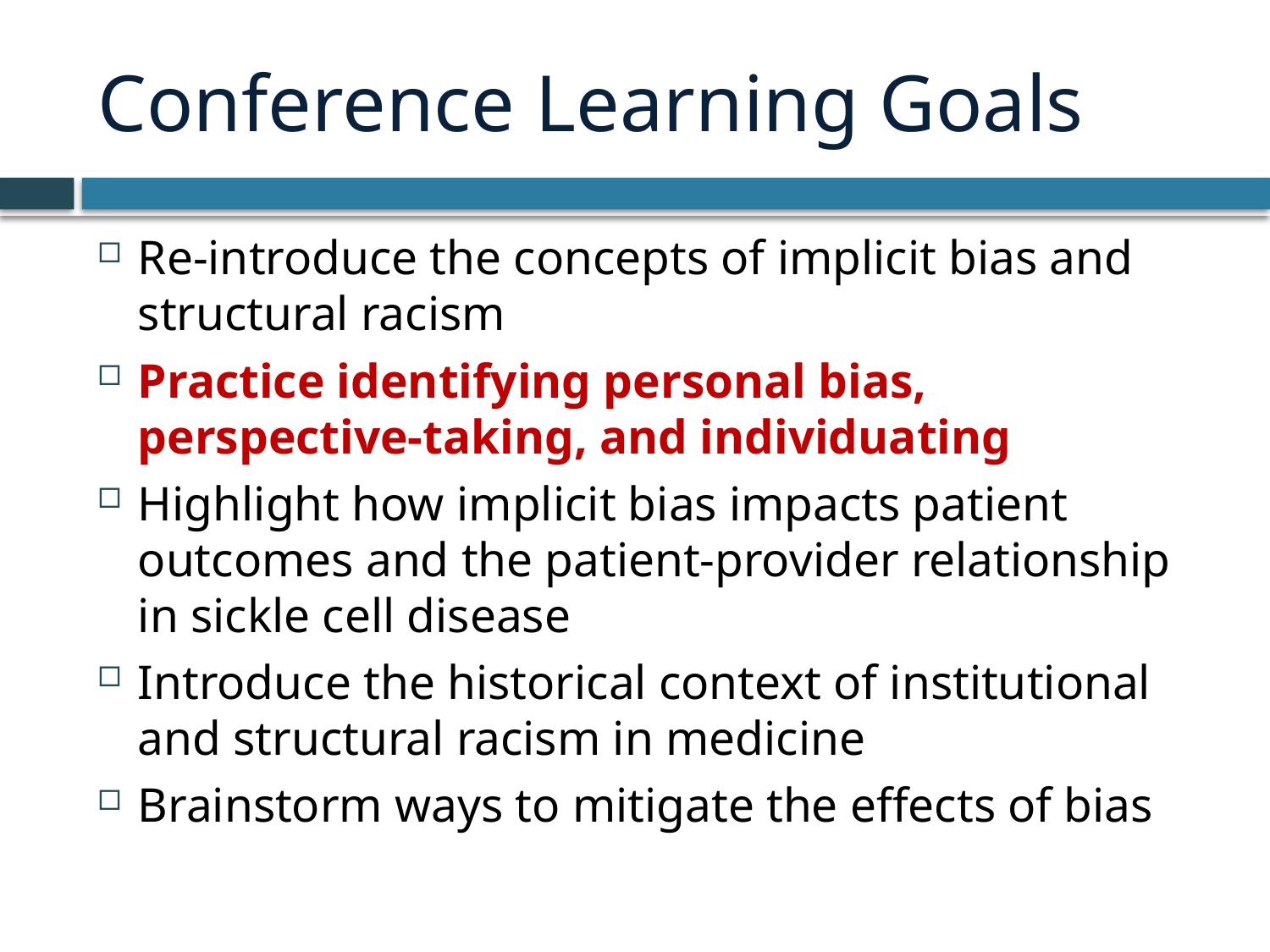

# Conference Learning Goals
Re-introduce the concepts of implicit bias and structural racism
Practice identifying personal bias, perspective-taking, and individuating
Highlight how implicit bias impacts patient outcomes and the patient-provider relationship in sickle cell disease
Introduce the historical context of institutional and structural racism in medicine
Brainstorm ways to mitigate the effects of bias

## Slide 19
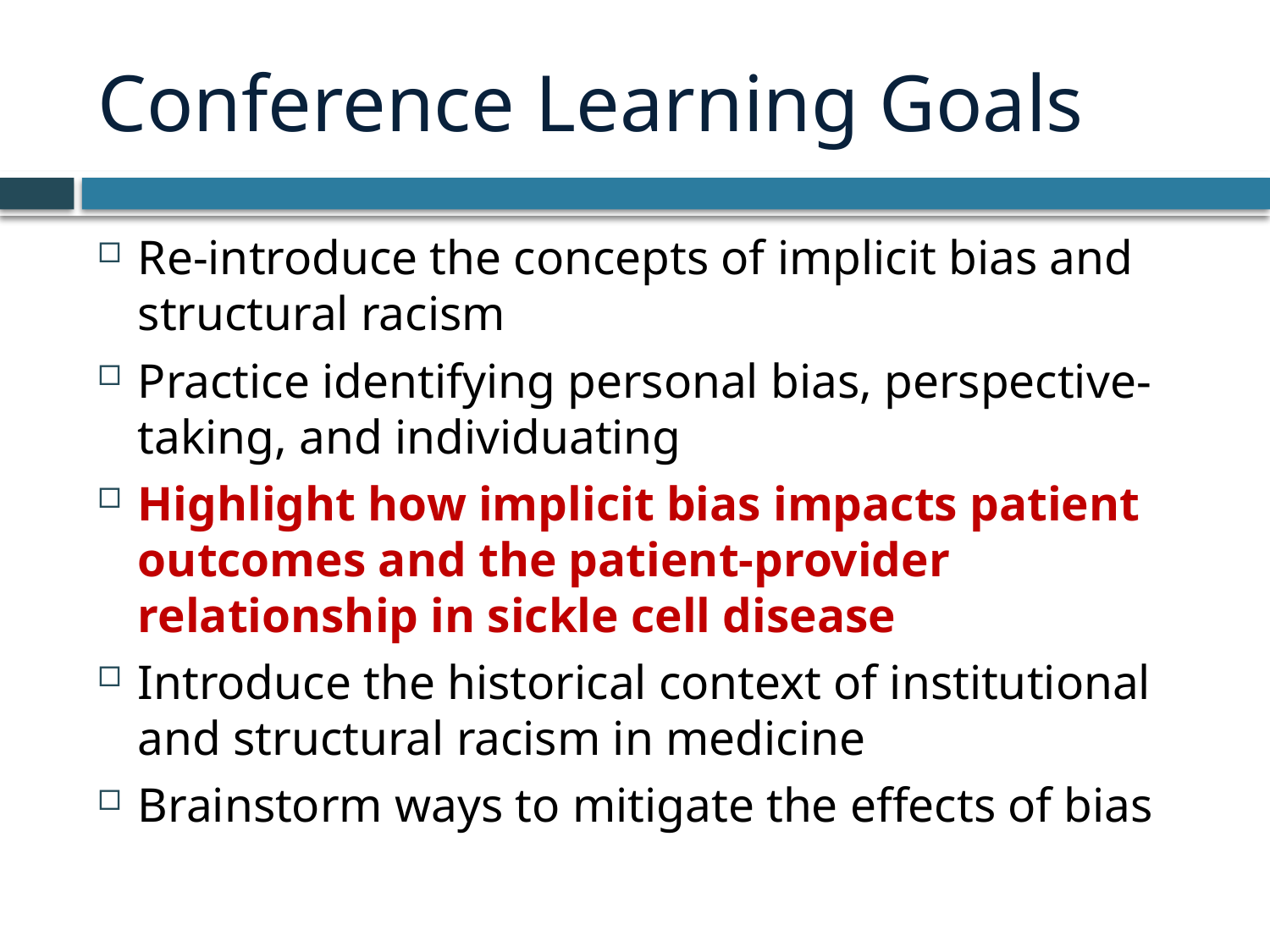

# Conference Learning Goals
Re-introduce the concepts of implicit bias and structural racism
Practice identifying personal bias, perspective- taking, and individuating
Highlight how implicit bias impacts patient outcomes and the patient-provider relationship in sickle cell disease
Introduce the historical context of institutional and structural racism in medicine
Brainstorm ways to mitigate the effects of bias

## Slide 20
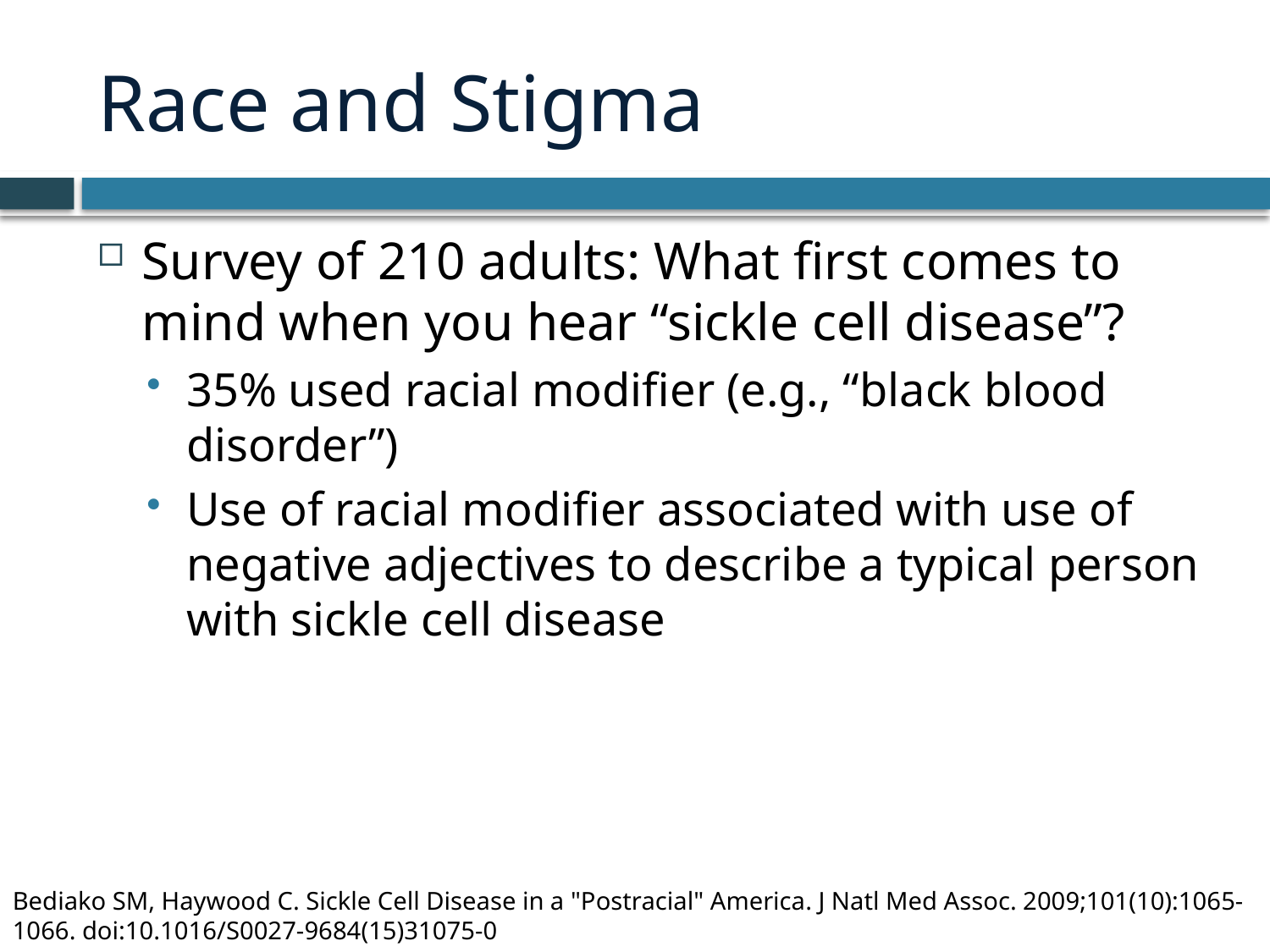

# Race and Stigma
Survey of 210 adults: What first comes to mind when you hear “sickle cell disease”?
35% used racial modifier (e.g., “black blood disorder”)
Use of racial modifier associated with use of negative adjectives to describe a typical person with sickle cell disease
Bediako SM, Haywood C. Sickle Cell Disease in a "Postracial" America. J Natl Med Assoc. 2009;101(10):1065-1066. doi:10.1016/S0027-9684(15)31075-0

## Slide 21
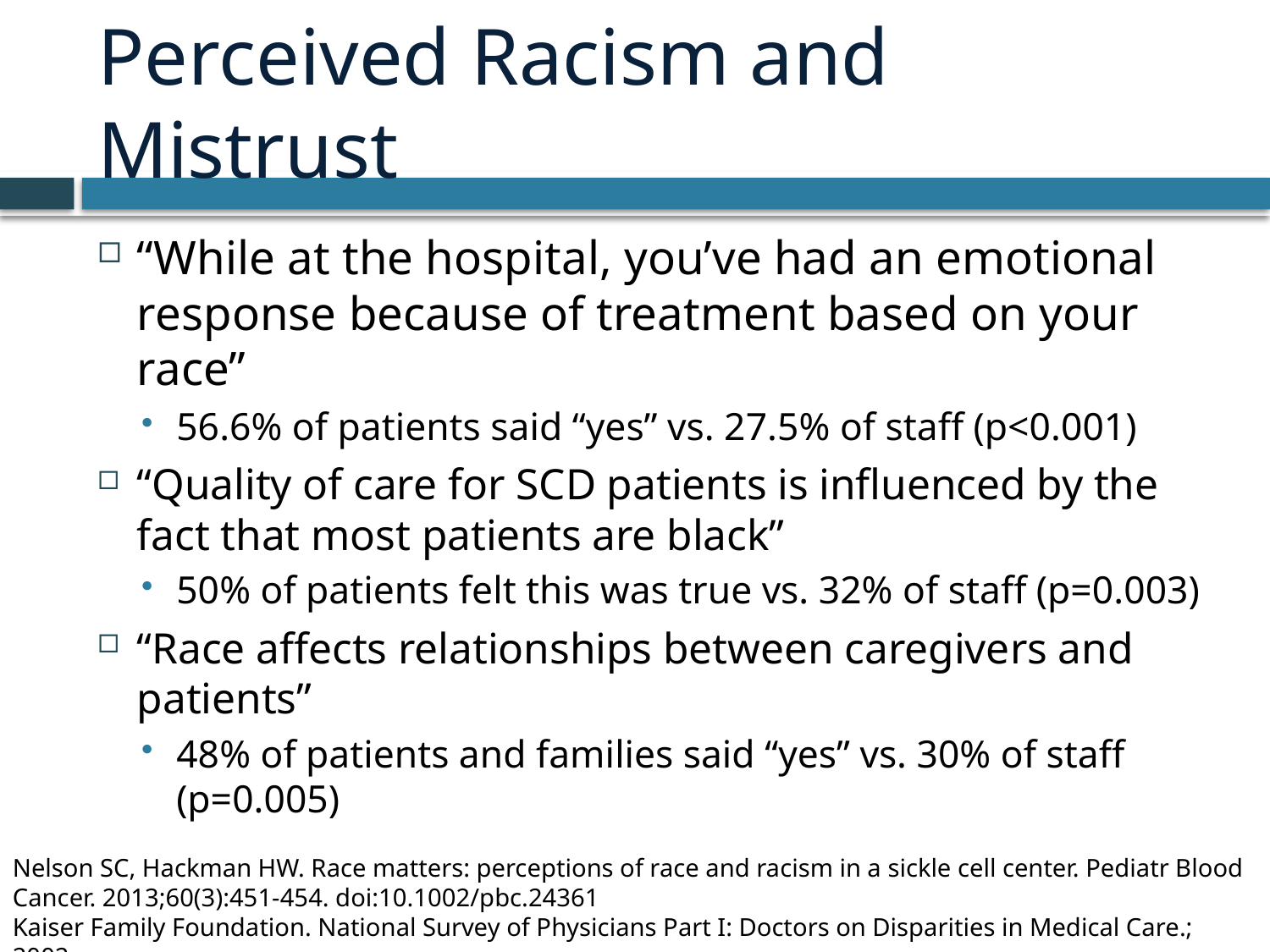

# Perceived Racism and Mistrust
“While at the hospital, you’ve had an emotional response because of treatment based on your race”
56.6% of patients said “yes” vs. 27.5% of staff (p<0.001)
“Quality of care for SCD patients is influenced by the fact that most patients are black”
50% of patients felt this was true vs. 32% of staff (p=0.003)
“Race affects relationships between caregivers and patients”
48% of patients and families said “yes” vs. 30% of staff (p=0.005)
Nelson SC, Hackman HW. Race matters: perceptions of race and racism in a sickle cell center. Pediatr Blood Cancer. 2013;60(3):451-454. doi:10.1002/pbc.24361
Kaiser Family Foundation. National Survey of Physicians Part I: Doctors on Disparities in Medical Care.; 2002.

## Slide 22
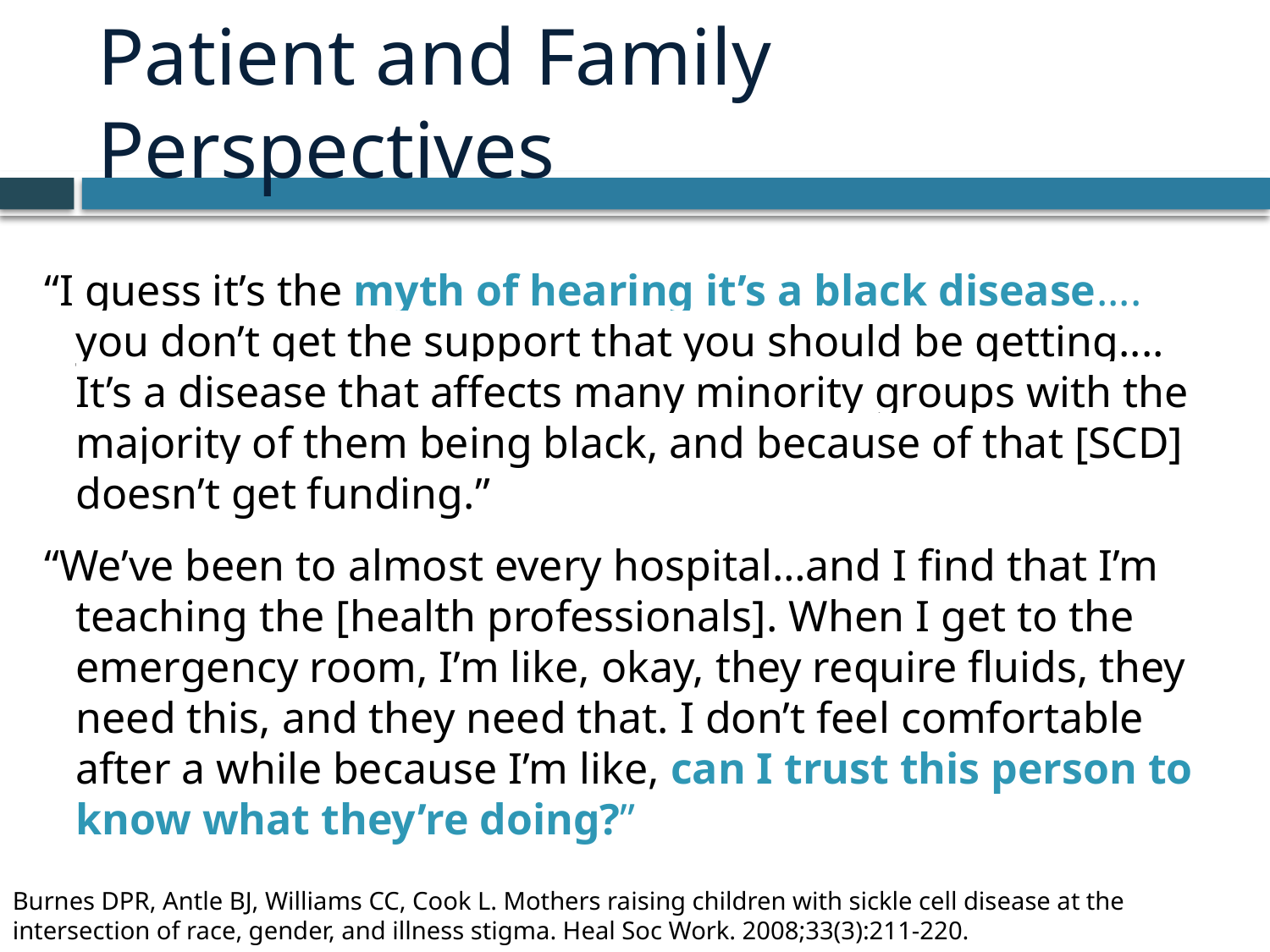

# Patient and Family Perspectives
“I guess it’s the myth of hearing it’s a black disease.... you don’t get the support that you should be getting.... It’s a disease that affects many minority groups with the majority of them being black, and because of that [SCD] doesn’t get funding.”
“We’ve been to almost every hospital…and I find that I’m teaching the [health professionals]. When I get to the emergency room, I’m like, okay, they require fluids, they need this, and they need that. I don’t feel comfortable after a while because I’m like, can I trust this person to know what they’re doing?”
Burnes DPR, Antle BJ, Williams CC, Cook L. Mothers raising children with sickle cell disease at the intersection of race, gender, and illness stigma. Heal Soc Work. 2008;33(3):211-220.

## Slide 23
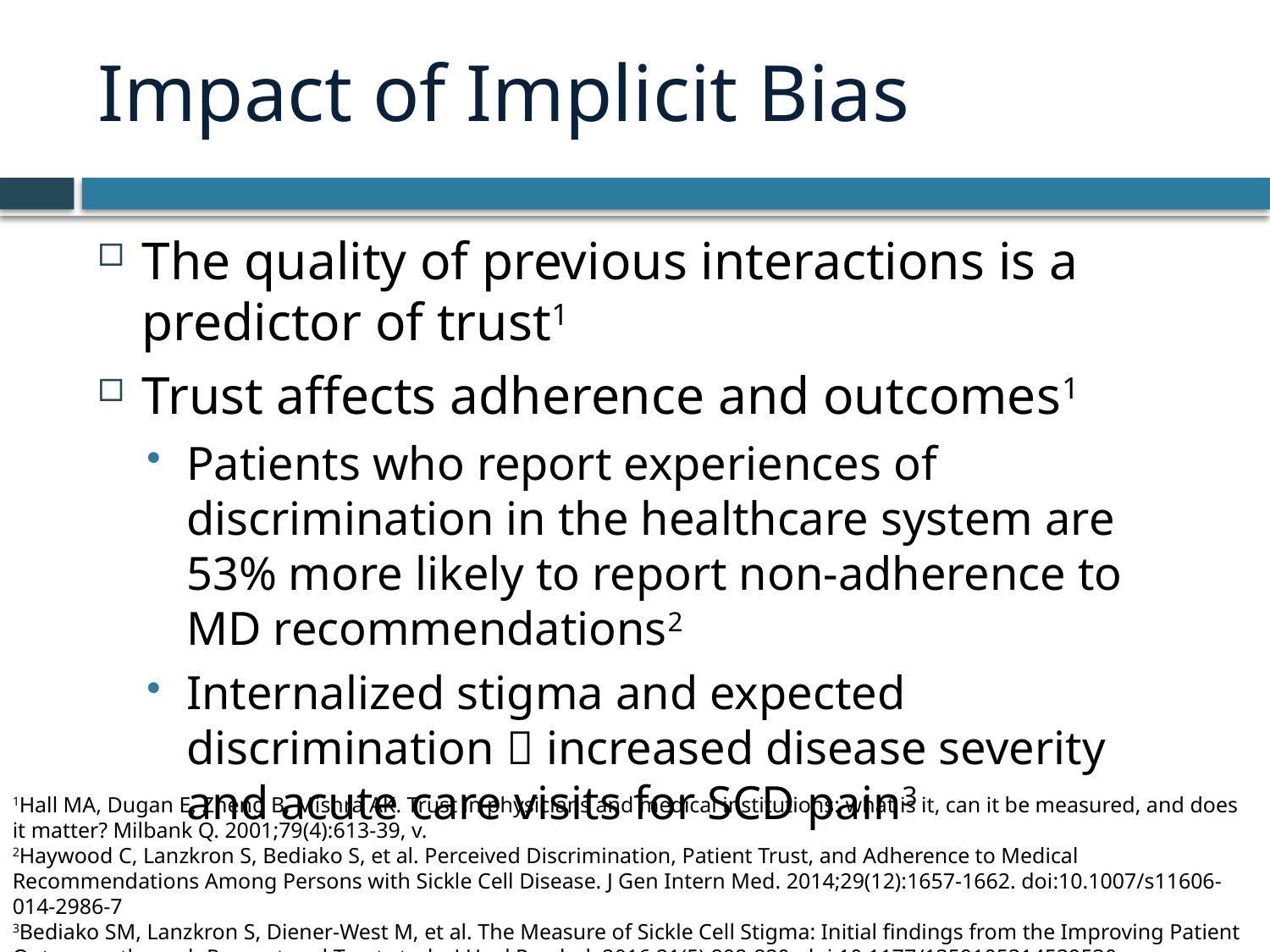

# Impact of Implicit Bias
The quality of previous interactions is a predictor of trust1
Trust affects adherence and outcomes1
Patients who report experiences of discrimination in the healthcare system are 53% more likely to report non-adherence to MD recommendations2
Internalized stigma and expected discrimination  increased disease severity and acute care visits for SCD pain3
1Hall MA, Dugan E, Zheng B, Mishra AK. Trust in physicians and medical institutions: what is it, can it be measured, and does it matter? Milbank Q. 2001;79(4):613-39, v.
2Haywood C, Lanzkron S, Bediako S, et al. Perceived Discrimination, Patient Trust, and Adherence to Medical Recommendations Among Persons with Sickle Cell Disease. J Gen Intern Med. 2014;29(12):1657-1662. doi:10.1007/s11606-014-2986-7
3Bediako SM, Lanzkron S, Diener-West M, et al. The Measure of Sickle Cell Stigma: Initial findings from the Improving Patient Outcomes through Respect and Trust study. J Heal Psychol. 2016;21(5):808-820. doi:10.1177/1359105314539530

## Slide 24
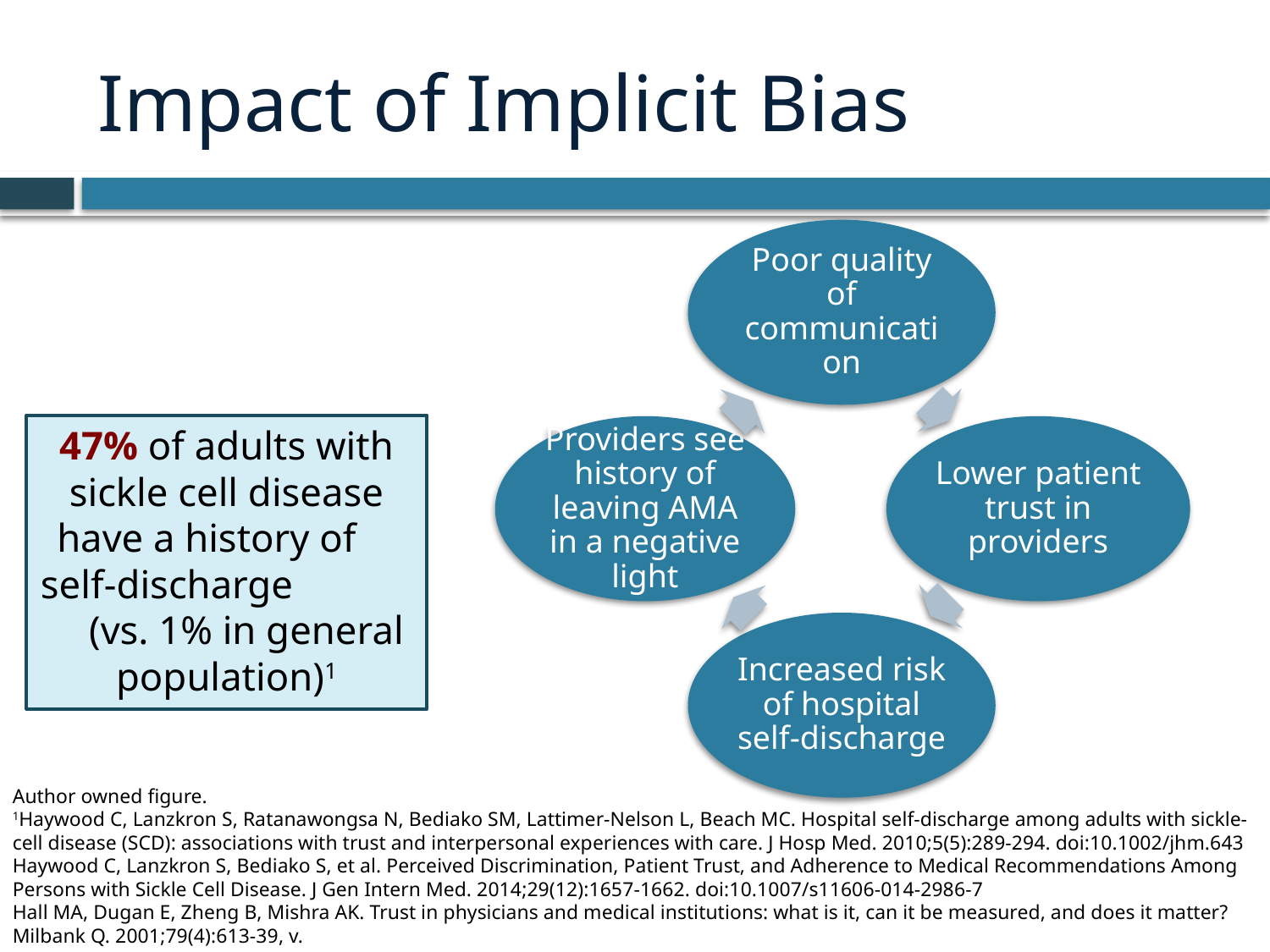

# Impact of Implicit Bias
47% of adults with sickle cell disease have a history of self-discharge (vs. 1% in general population)1
Author owned figure.
1Haywood C, Lanzkron S, Ratanawongsa N, Bediako SM, Lattimer-Nelson L, Beach MC. Hospital self-discharge among adults with sickle-cell disease (SCD): associations with trust and interpersonal experiences with care. J Hosp Med. 2010;5(5):289-294. doi:10.1002/jhm.643
Haywood C, Lanzkron S, Bediako S, et al. Perceived Discrimination, Patient Trust, and Adherence to Medical Recommendations Among Persons with Sickle Cell Disease. J Gen Intern Med. 2014;29(12):1657-1662. doi:10.1007/s11606-014-2986-7
Hall MA, Dugan E, Zheng B, Mishra AK. Trust in physicians and medical institutions: what is it, can it be measured, and does it matter? Milbank Q. 2001;79(4):613-39, v.

## Slide 25
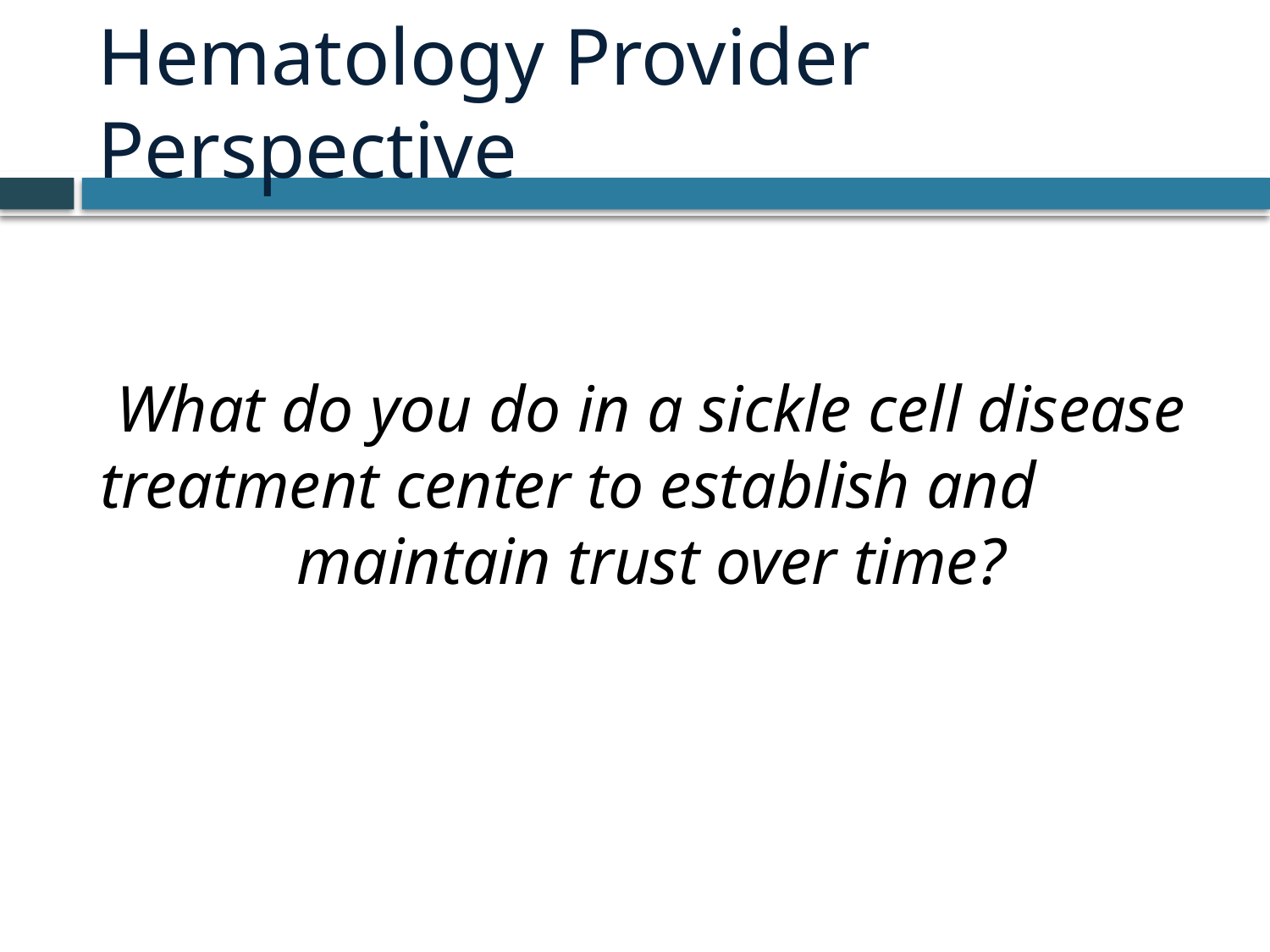

# Hematology Provider Perspective
What do you do in a sickle cell disease treatment center to establish and maintain trust over time?

## Slide 26
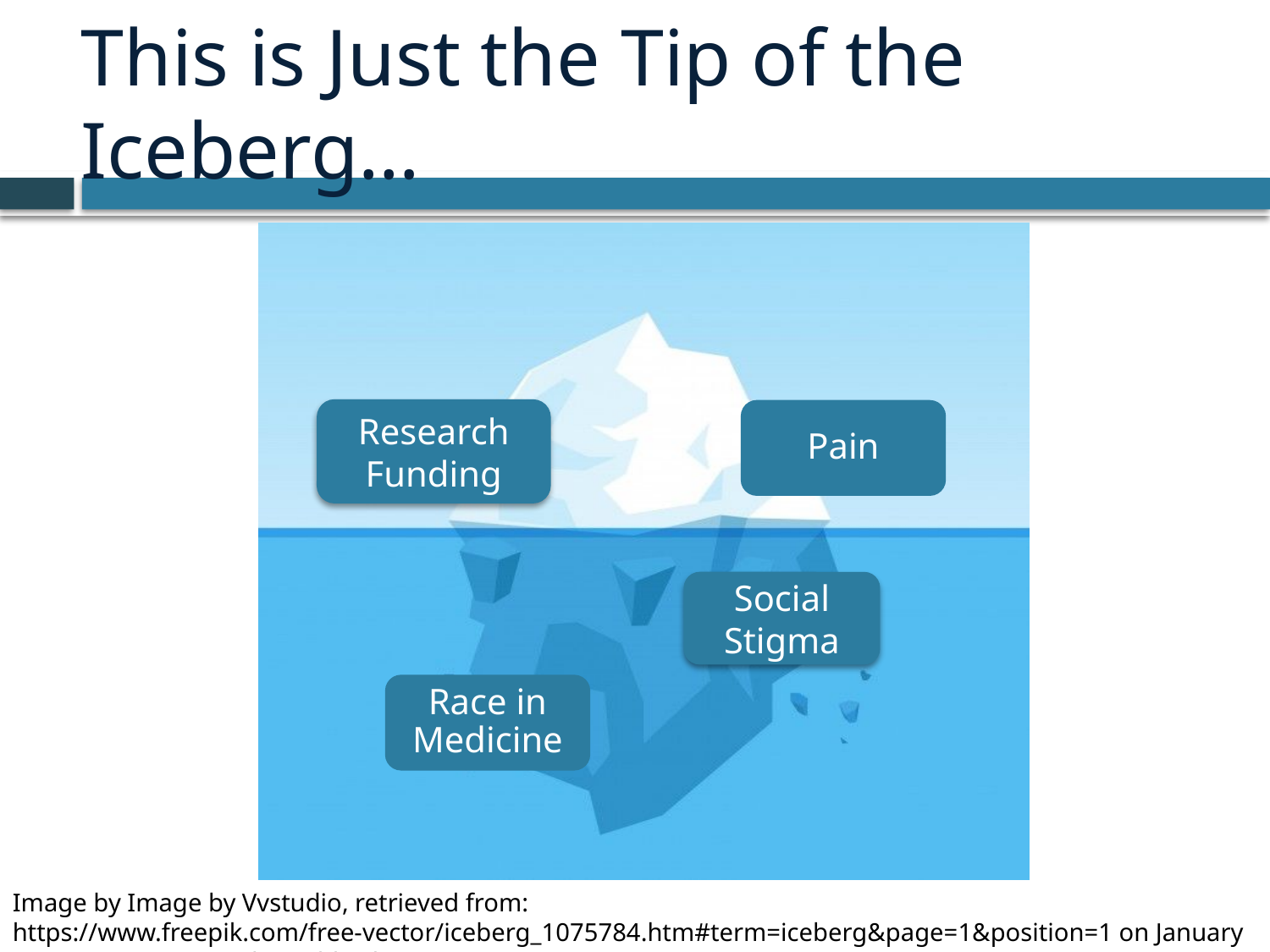

# This is Just the Tip of the Iceberg…
Research Funding
Pain
Social Stigma
Race in Medicine
Image by Image by Vvstudio, retrieved from: https://www.freepik.com/free-vector/iceberg_1075784.htm#term=iceberg&page=1&position=1 on January 6, 2019. Image is in the public domain.

## Slide 27
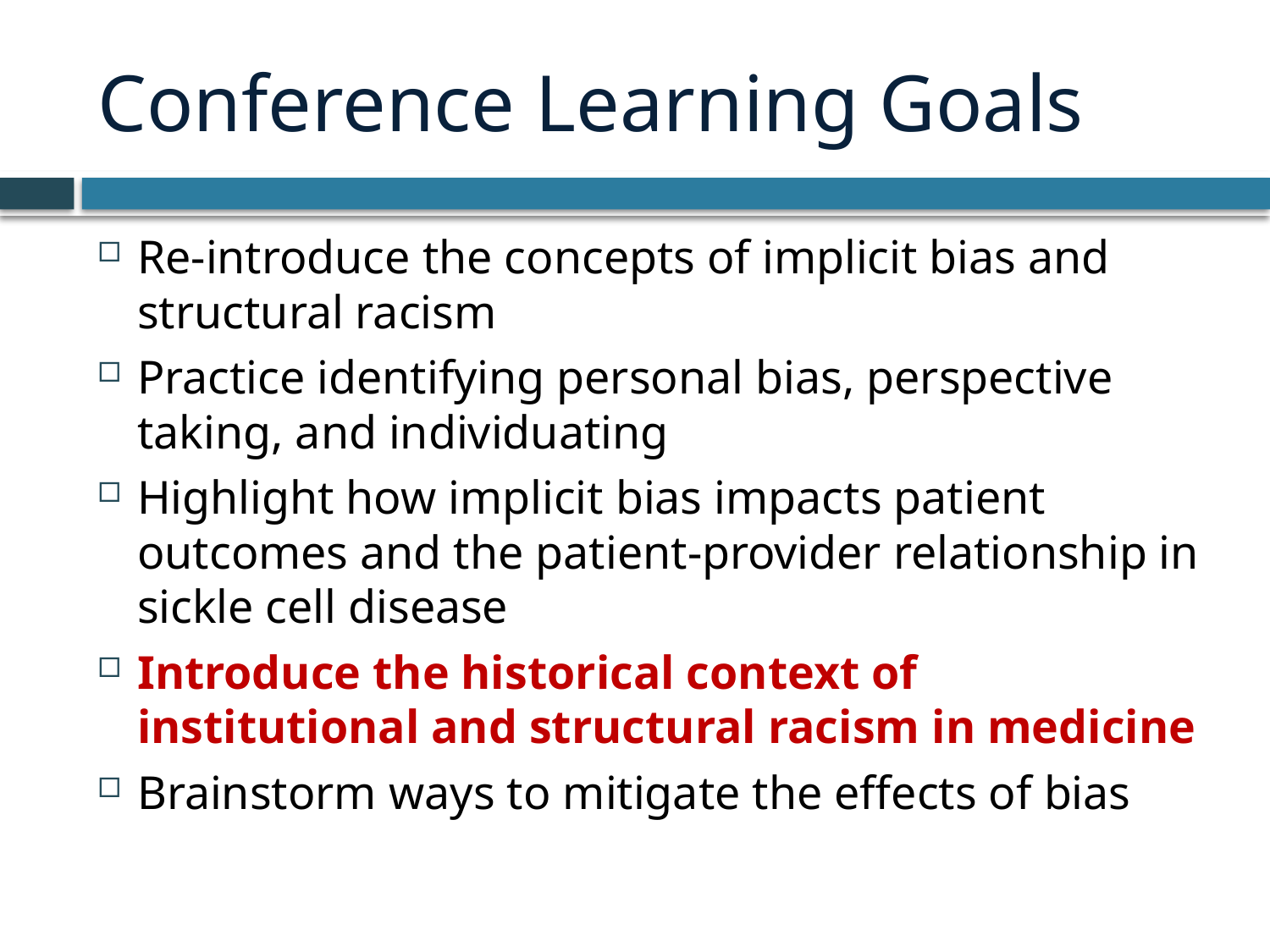

# Conference Learning Goals
Re-introduce the concepts of implicit bias and structural racism
Practice identifying personal bias, perspective taking, and individuating
Highlight how implicit bias impacts patient outcomes and the patient-provider relationship in sickle cell disease
Introduce the historical context of institutional and structural racism in medicine
Brainstorm ways to mitigate the effects of bias

## Slide 28
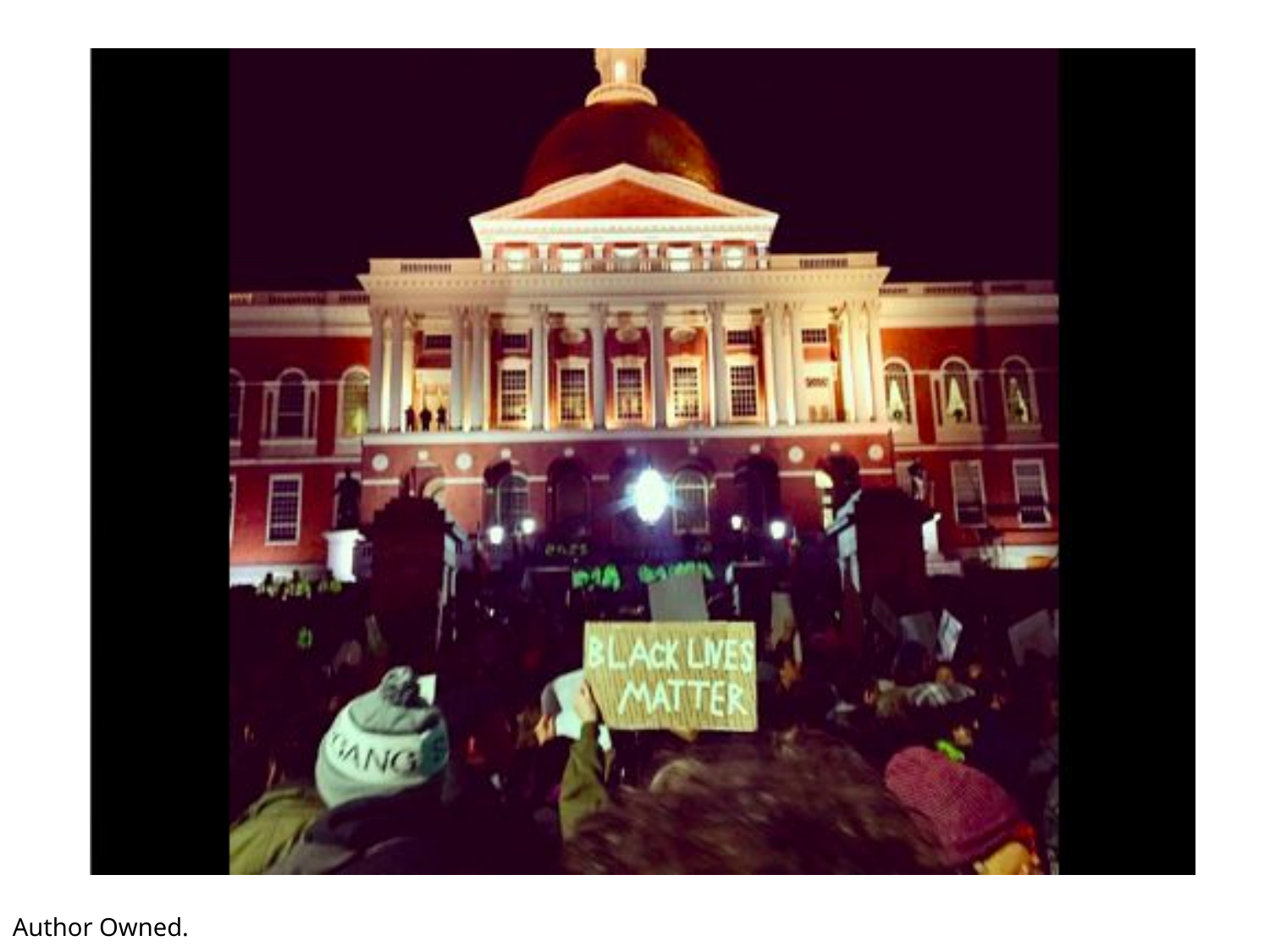

Author Owned.

## Slide 29
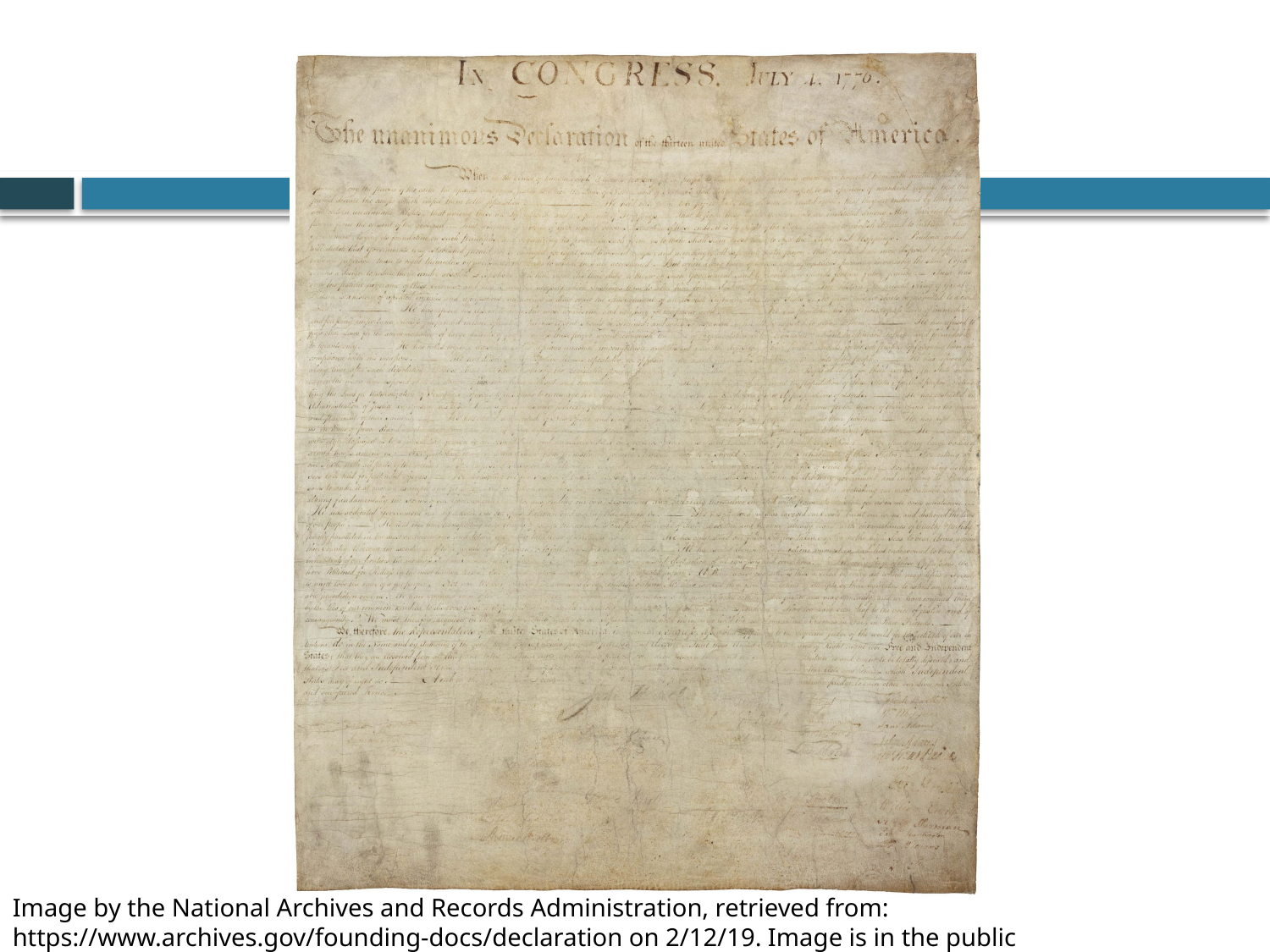

Image by the National Archives and Records Administration, retrieved from: https://www.archives.gov/founding-docs/declaration on 2/12/19. Image is in the public domain.

## Slide 30
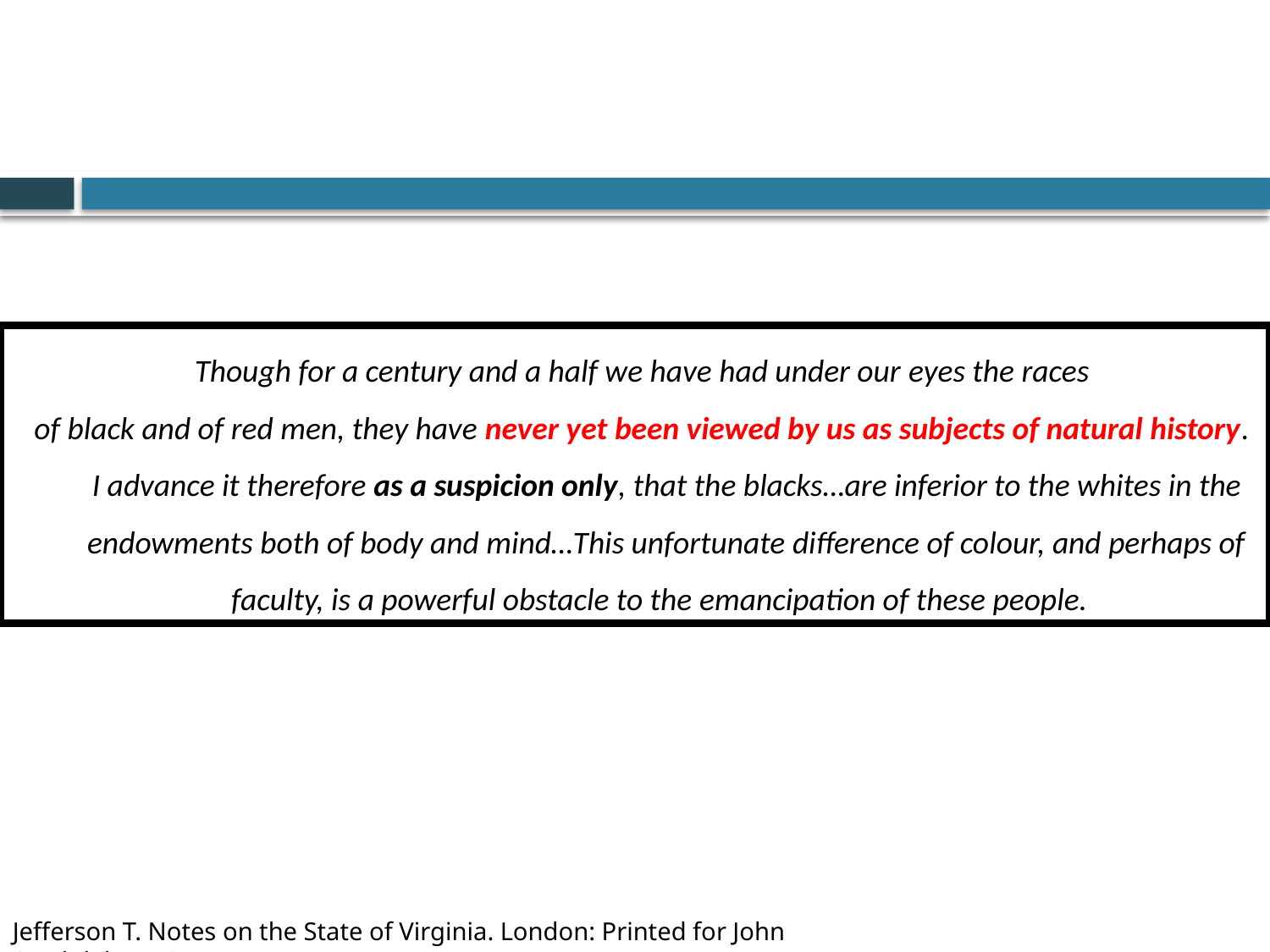

Though for a century and a half we have had under our eyes the races
of black and of red men, they have never yet been viewed by us as subjects of natural history.
I advance it therefore as a suspicion only, that the blacks…are inferior to the whites in the endowments both of body and mind…This unfortunate difference of colour, and perhaps of faculty, is a powerful obstacle to the emancipation of these people.
Though for a century and a half we have had under our eyes the races
of black and of red men, they have never yet been viewed by us as subjects of natural history. I advance it therefore as a suspicion only, that the blacks…are inferior to the whites in the endowments both of body and mind…This unfortunate difference of colour, and perhaps of faculty, is a powerful obstacle to the emancipation of these people.
Jefferson T. Notes on the State of Virginia. London: Printed for John Stockdale; 1787

## Slide 31
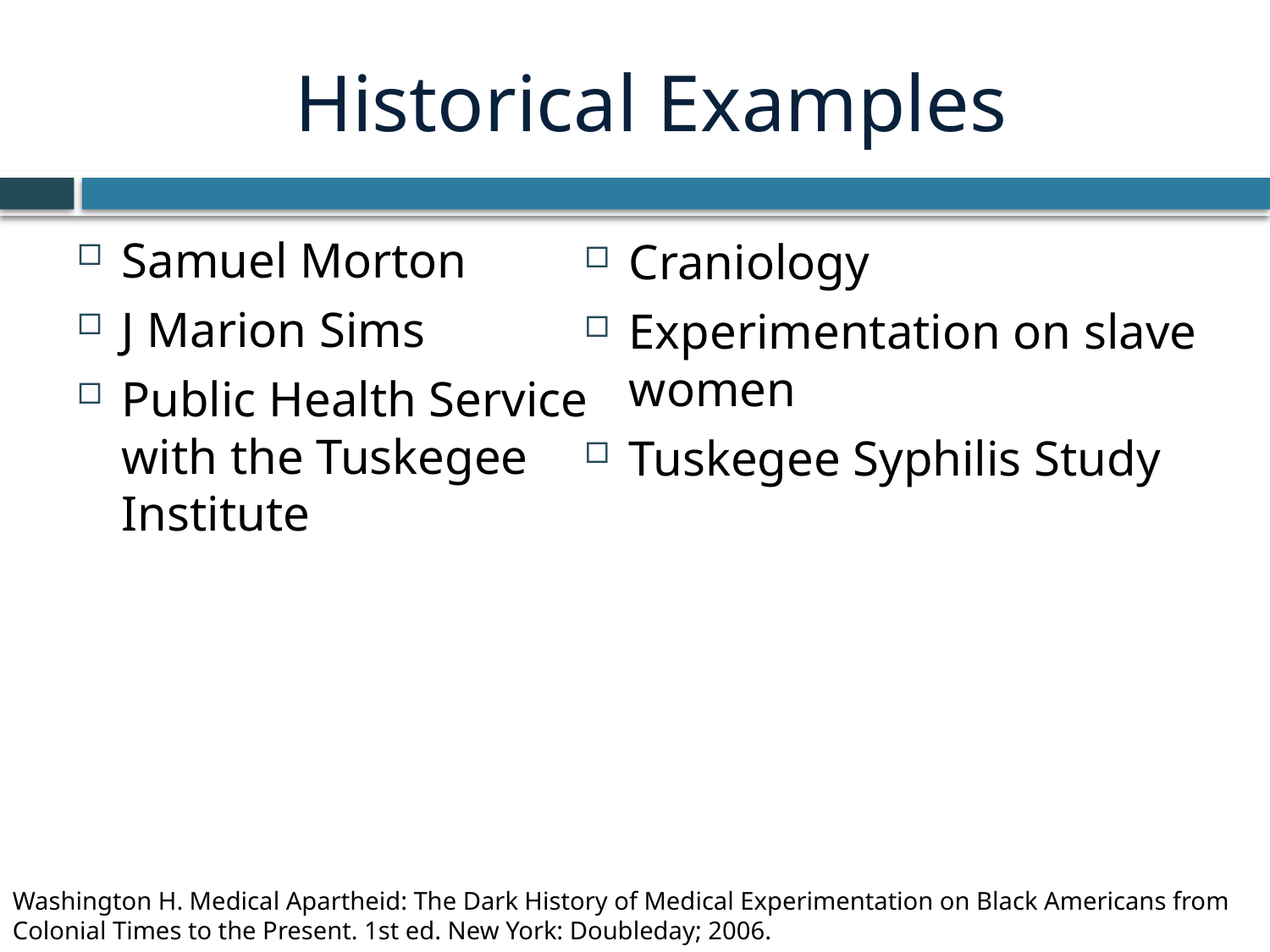

# Historical Examples
Samuel Morton
J Marion Sims
Public Health Service with the Tuskegee Institute
Craniology
Experimentation on slave women
Tuskegee Syphilis Study
Washington H. Medical Apartheid: The Dark History of Medical Experimentation on Black Americans from Colonial Times to the Present. 1st ed. New York: Doubleday; 2006.

## Slide 32
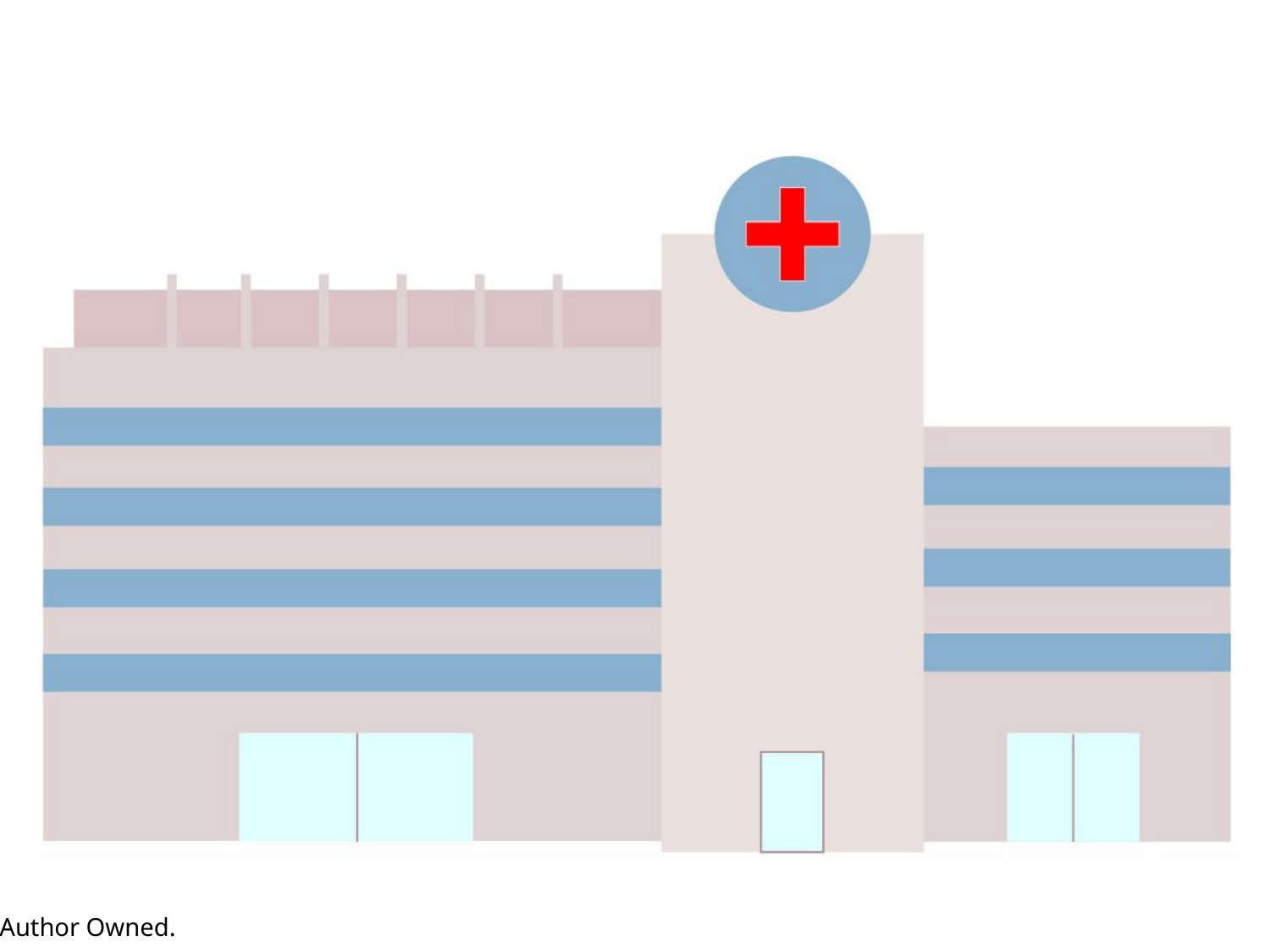

Author Owned.

## Slide 33
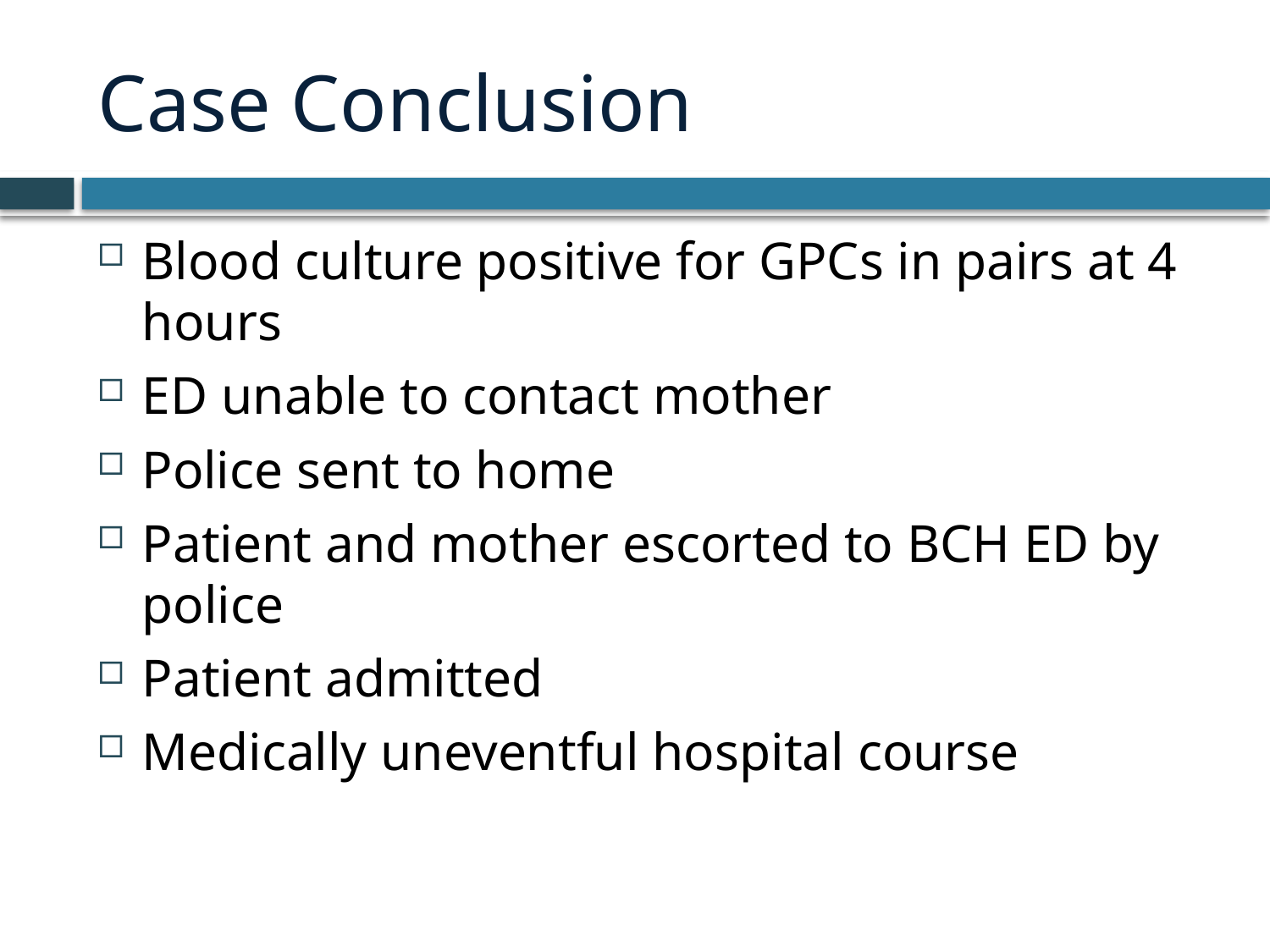

# Case Conclusion
Blood culture positive for GPCs in pairs at 4 hours
ED unable to contact mother
Police sent to home
Patient and mother escorted to BCH ED by police
Patient admitted
Medically uneventful hospital course

## Slide 34
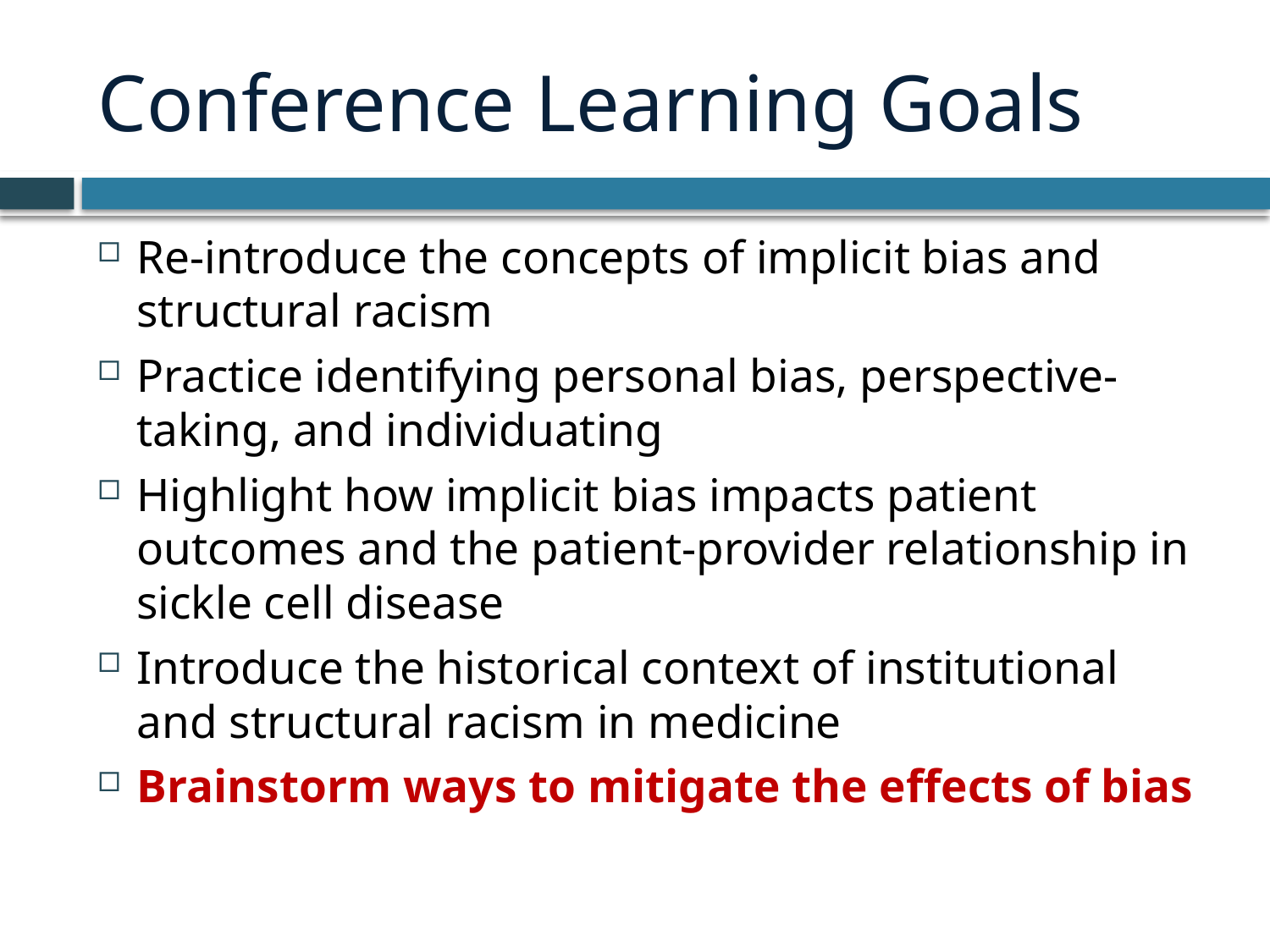

# Conference Learning Goals
Re-introduce the concepts of implicit bias and structural racism
Practice identifying personal bias, perspective- taking, and individuating
Highlight how implicit bias impacts patient outcomes and the patient-provider relationship in sickle cell disease
Introduce the historical context of institutional and structural racism in medicine
Brainstorm ways to mitigate the effects of bias

## Slide 35
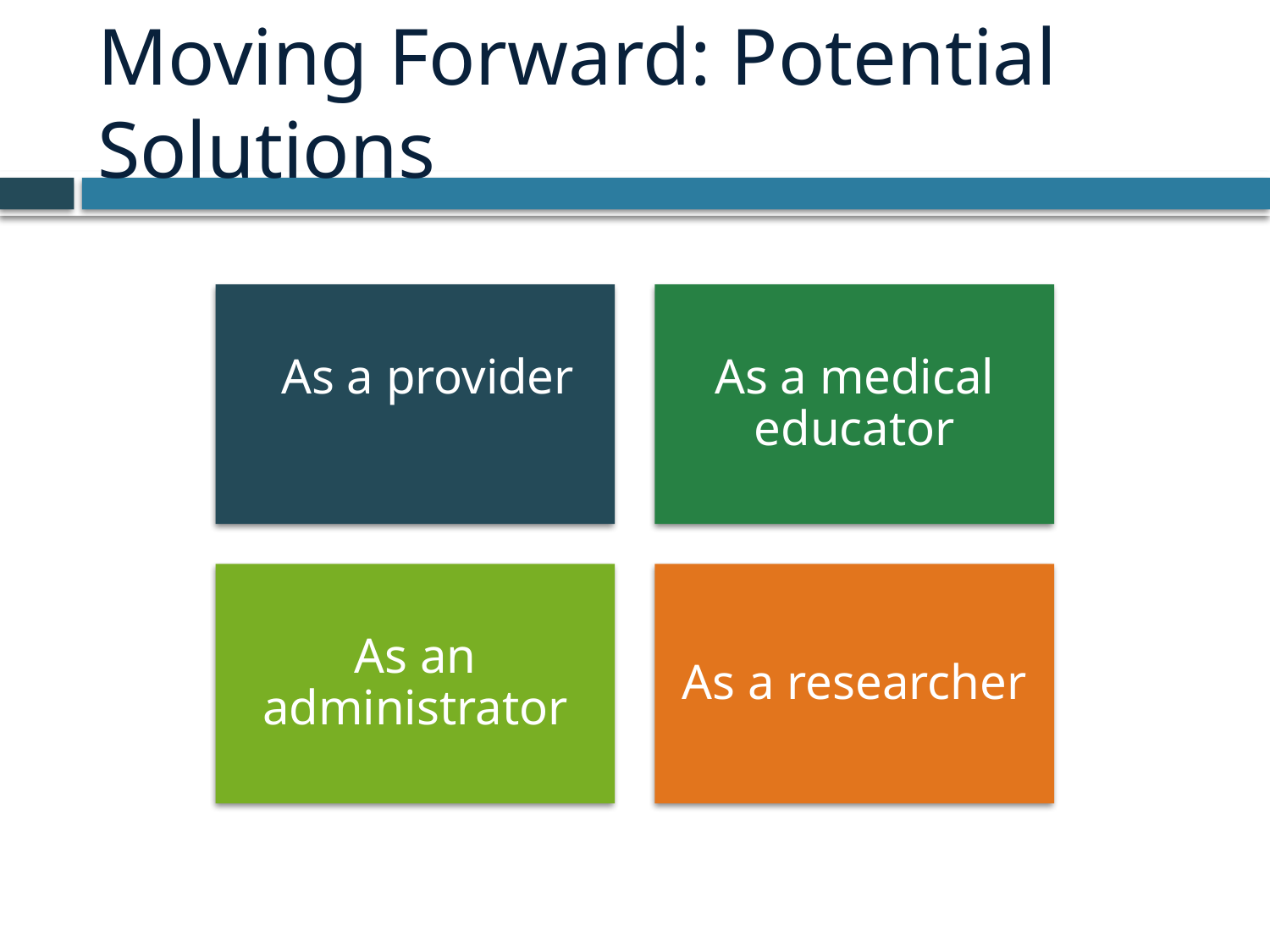

# Moving Forward: Potential Solutions

## Slide 36
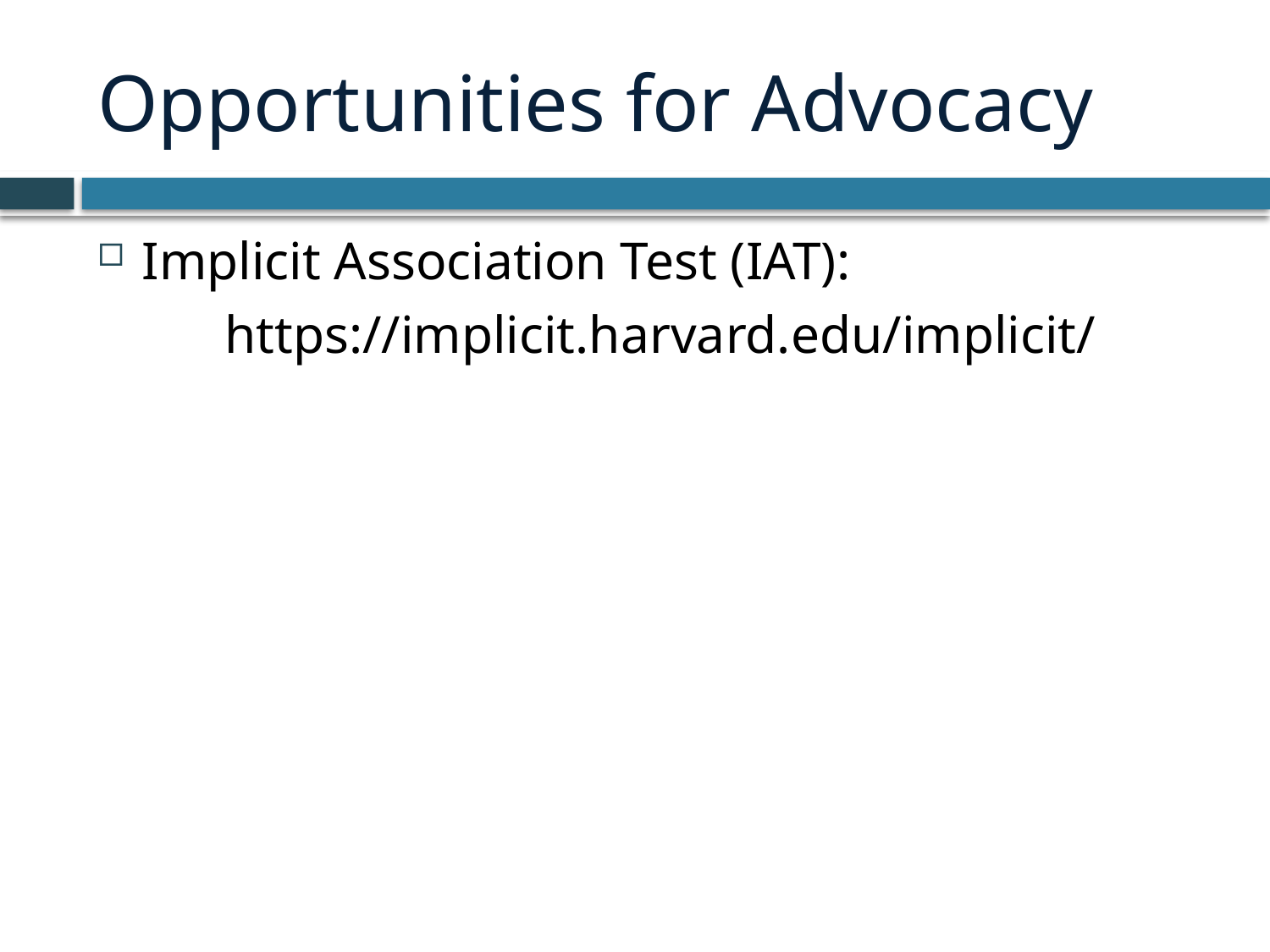

# Opportunities for Advocacy
Implicit Association Test (IAT):
	https://implicit.harvard.edu/implicit/

## Slide 37
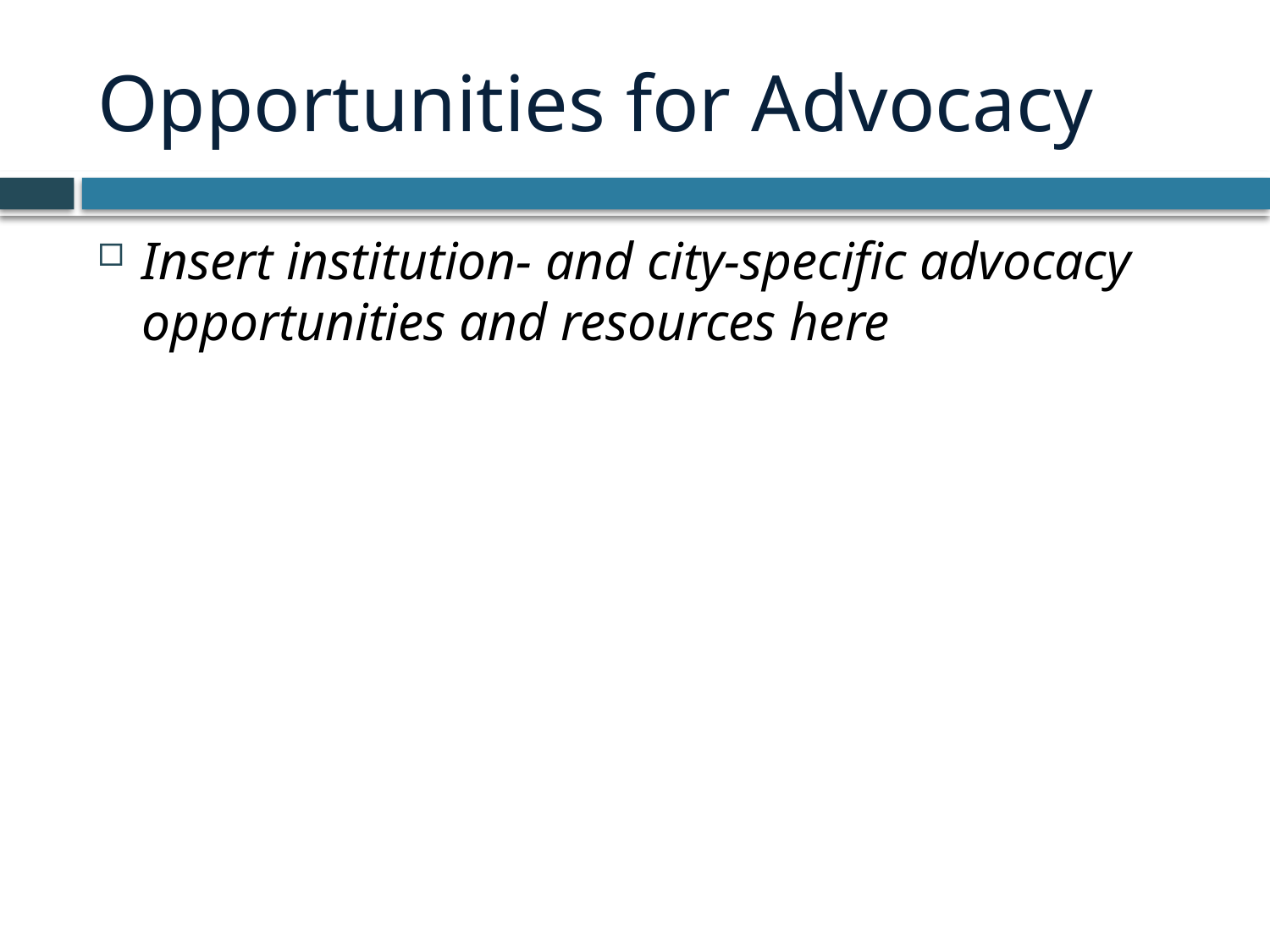

# Opportunities for Advocacy
Insert institution- and city-specific advocacy opportunities and resources here

## Slide 38
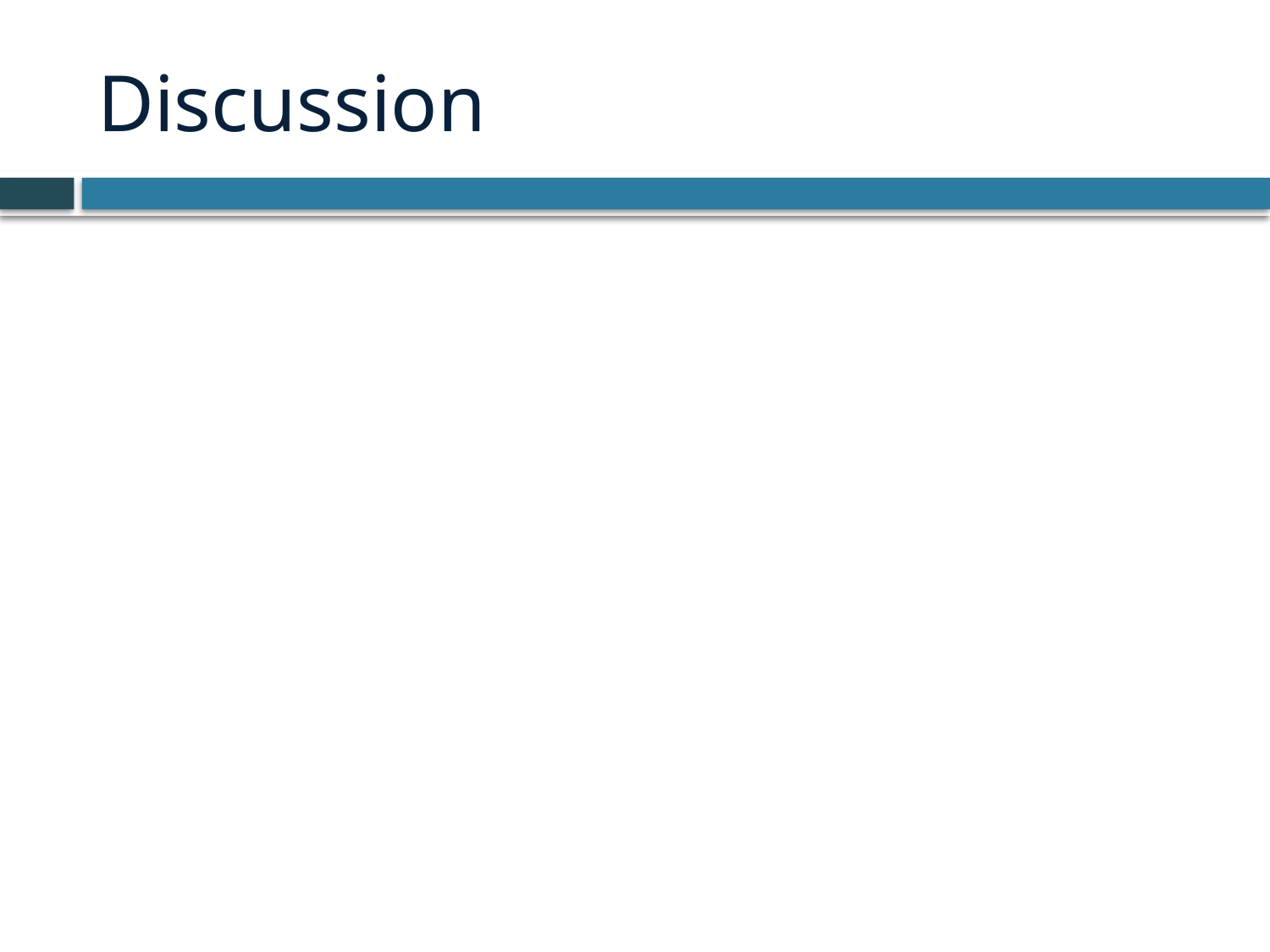

# Discussion

## Slide 39
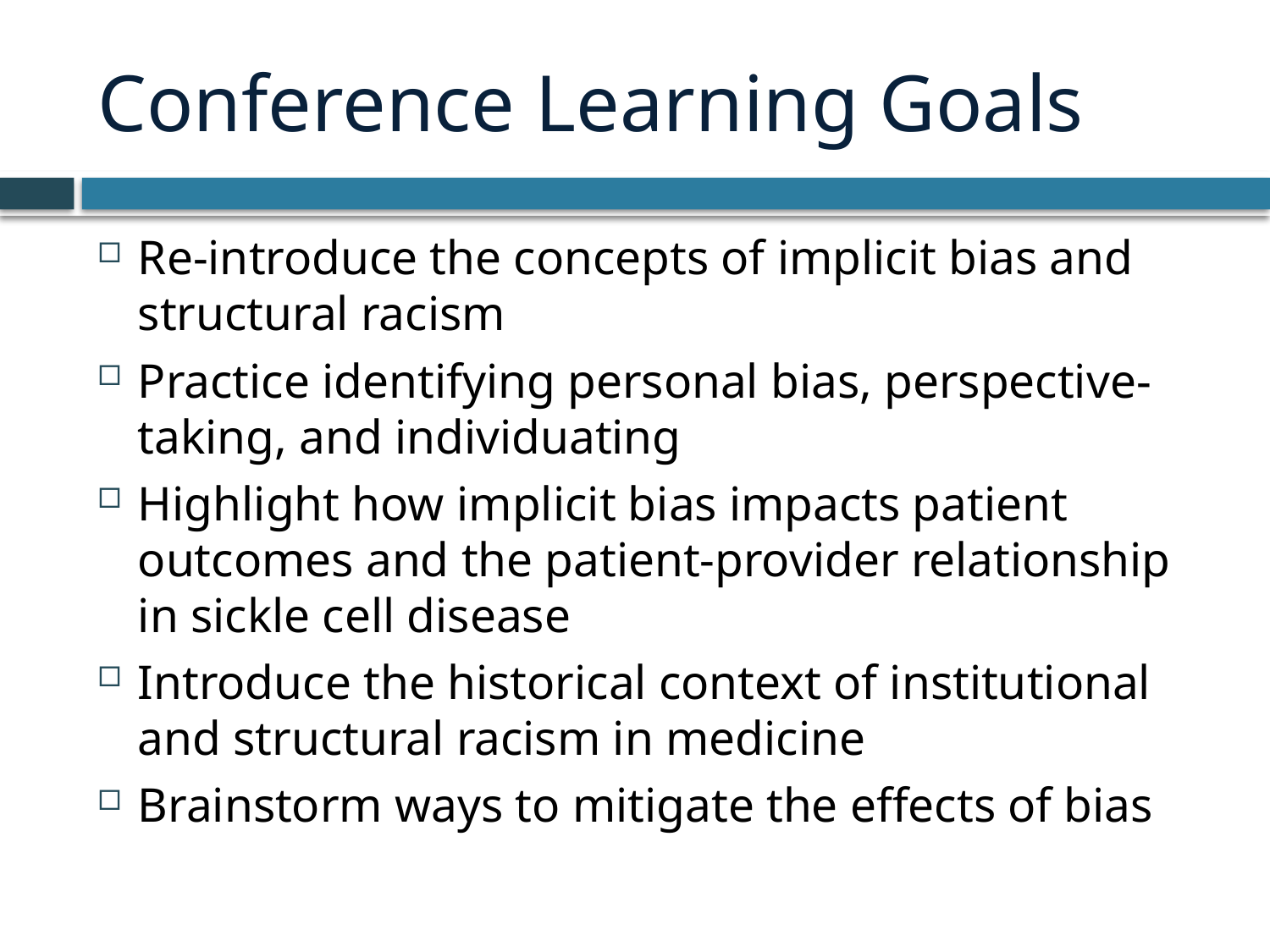

# Conference Learning Goals
Re-introduce the concepts of implicit bias and structural racism
Practice identifying personal bias, perspective- taking, and individuating
Highlight how implicit bias impacts patient outcomes and the patient-provider relationship in sickle cell disease
Introduce the historical context of institutional and structural racism in medicine
Brainstorm ways to mitigate the effects of bias

## Slide 40
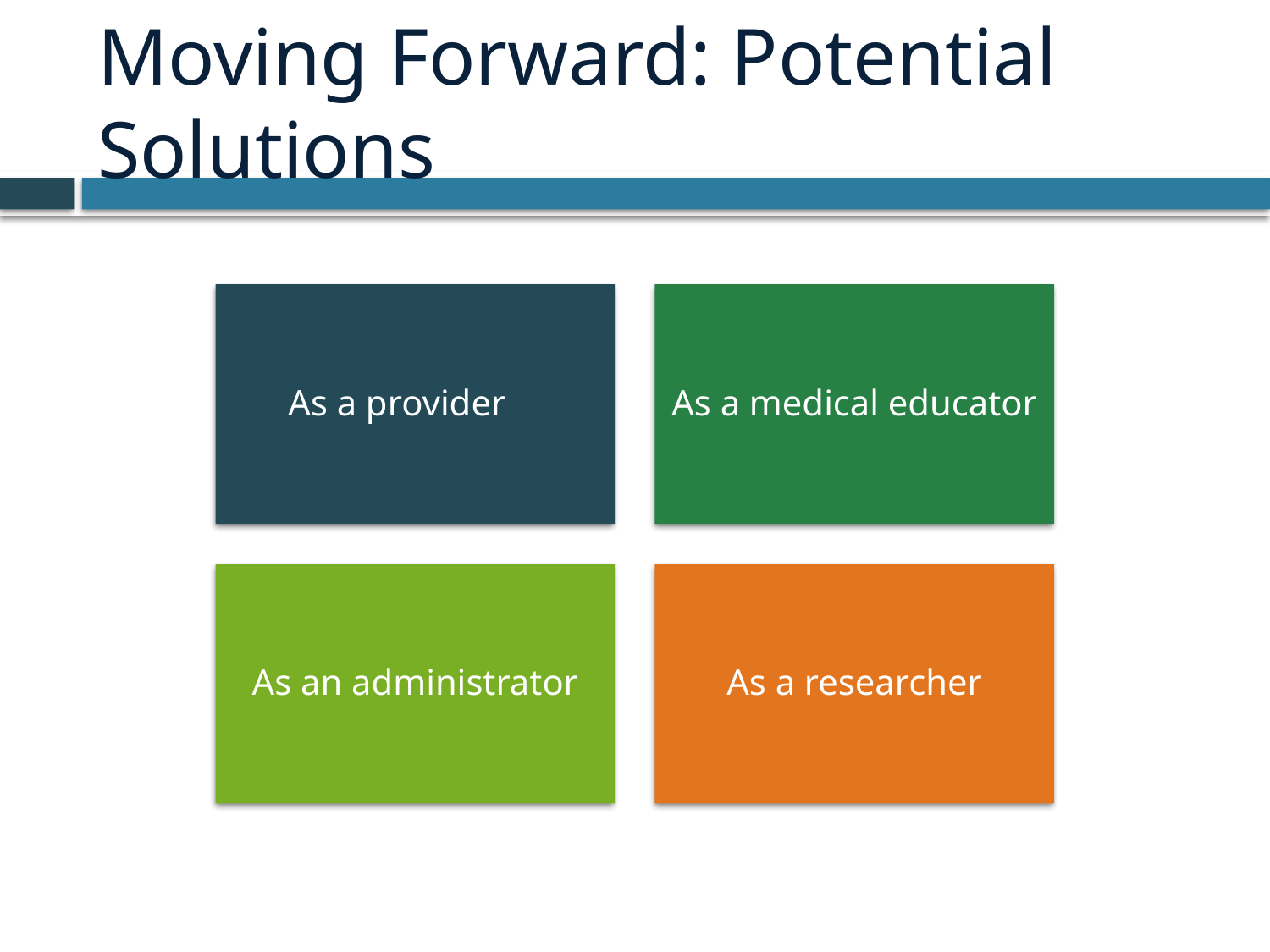

# Moving Forward: Potential Solutions

## Slide 41
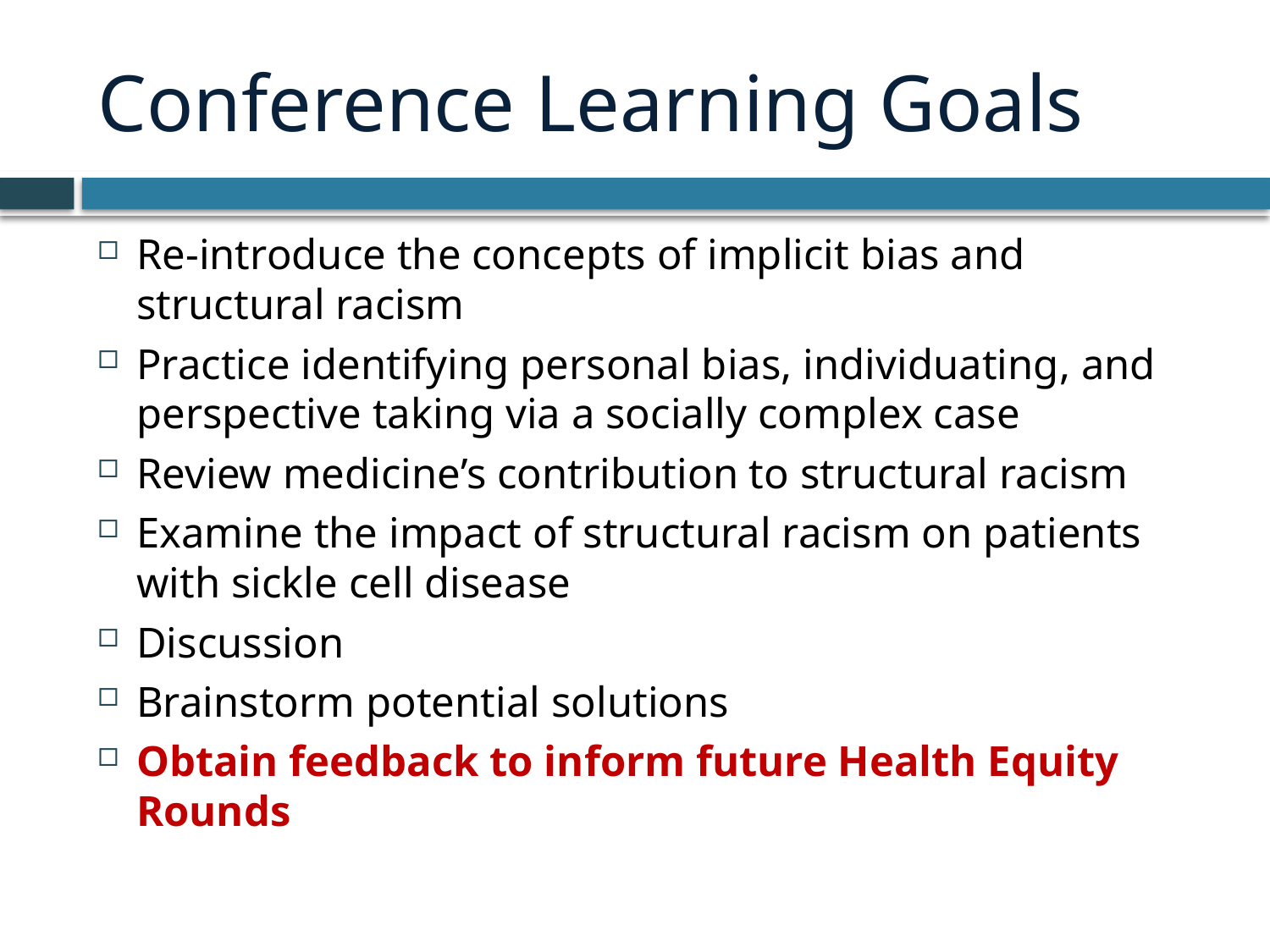

# Conference Learning Goals
Re-introduce the concepts of implicit bias and structural racism
Practice identifying personal bias, individuating, and perspective taking via a socially complex case
Review medicine’s contribution to structural racism
Examine the impact of structural racism on patients with sickle cell disease
Discussion
Brainstorm potential solutions
Obtain feedback to inform future Health Equity Rounds

## Slide 42
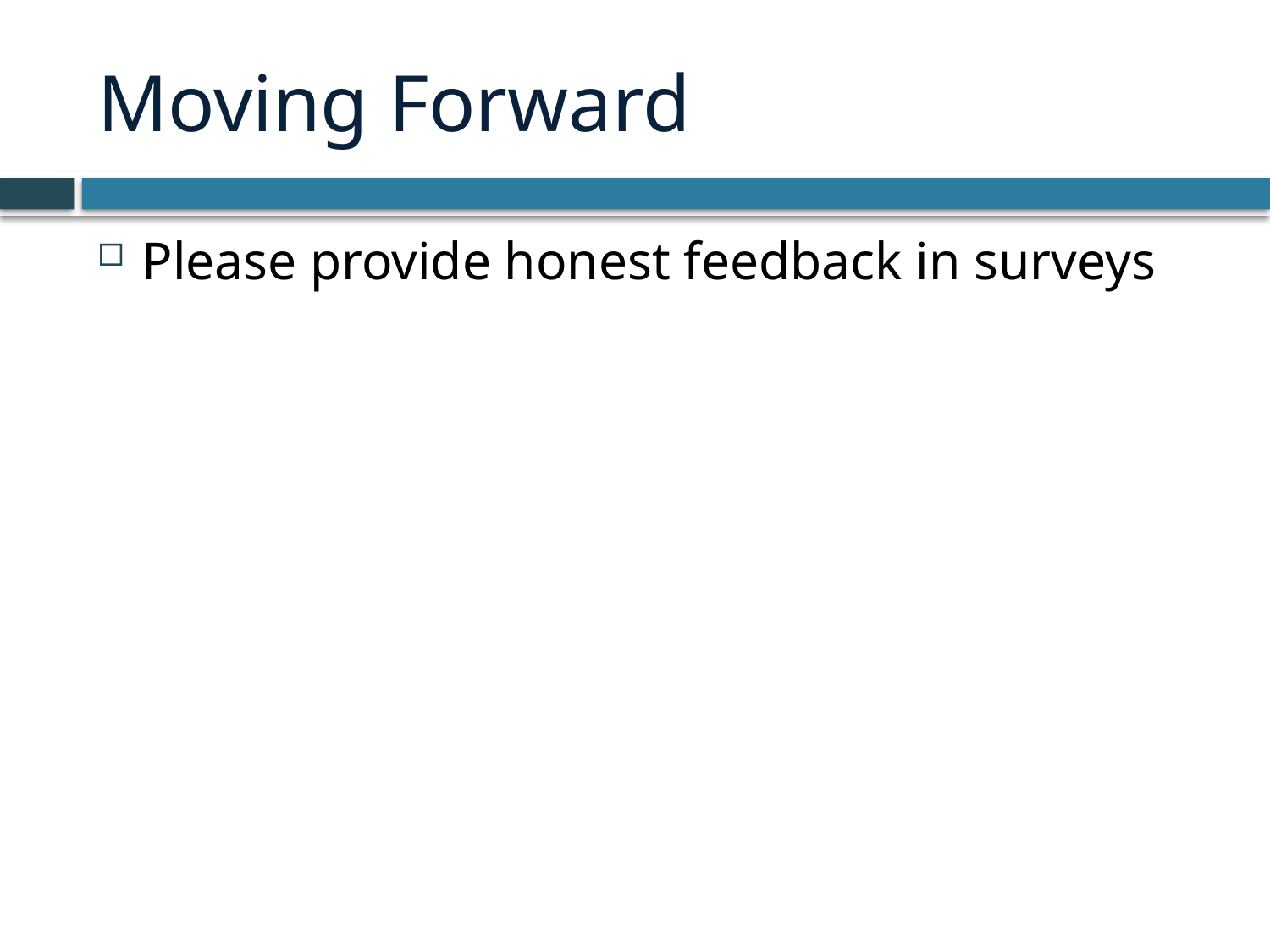

# Moving Forward
Please provide honest feedback in surveys

## Slide 43
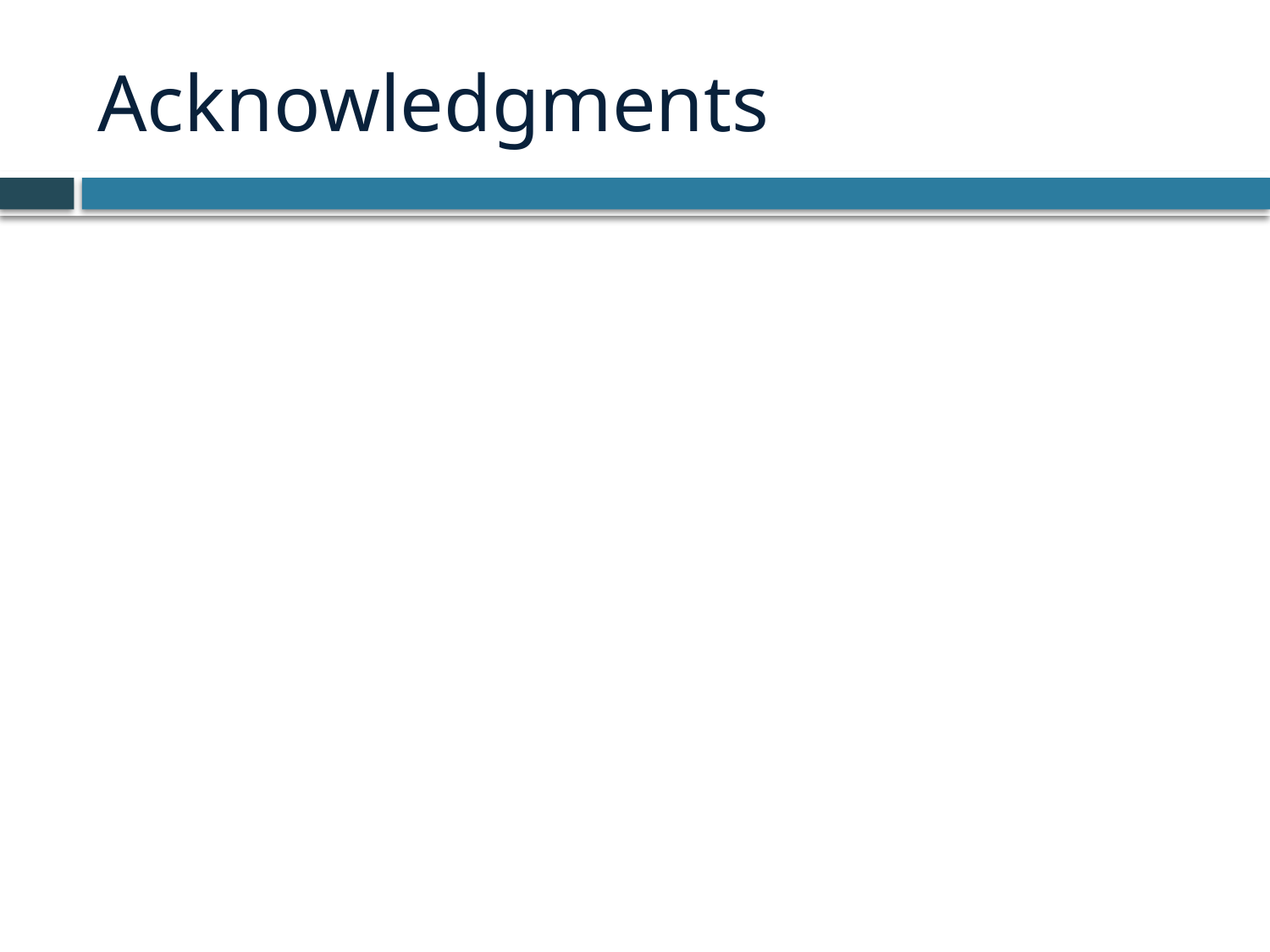

# Acknowledgments

## Slide 44
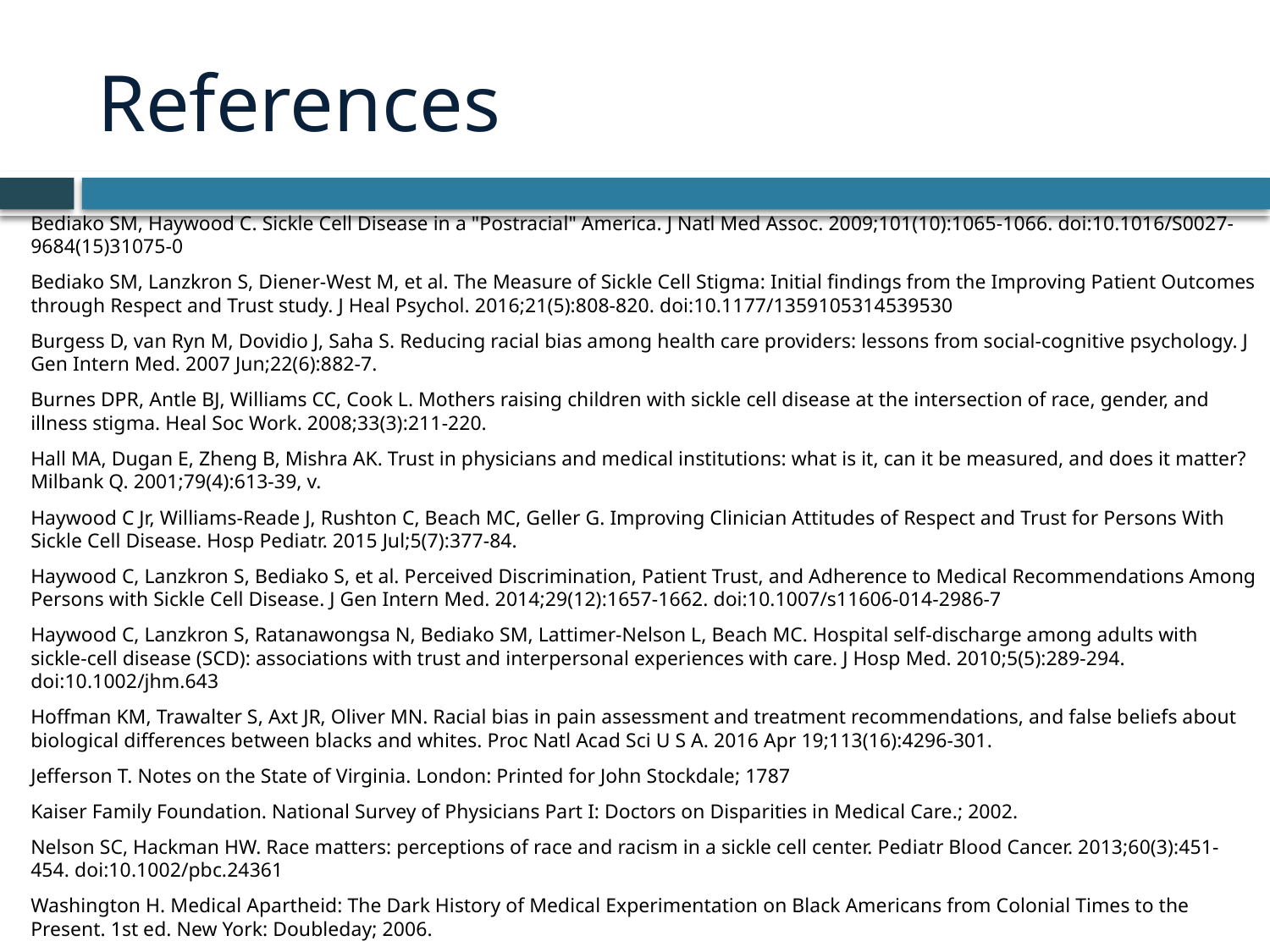

# References
Bediako SM, Haywood C. Sickle Cell Disease in a "Postracial" America. J Natl Med Assoc. 2009;101(10):1065-1066. doi:10.1016/S0027-9684(15)31075-0
Bediako SM, Lanzkron S, Diener-West M, et al. The Measure of Sickle Cell Stigma: Initial findings from the Improving Patient Outcomes through Respect and Trust study. J Heal Psychol. 2016;21(5):808-820. doi:10.1177/1359105314539530
Burgess D, van Ryn M, Dovidio J, Saha S. Reducing racial bias among health care providers: lessons from social-cognitive psychology. J Gen Intern Med. 2007 Jun;22(6):882-7.
Burnes DPR, Antle BJ, Williams CC, Cook L. Mothers raising children with sickle cell disease at the intersection of race, gender, and illness stigma. Heal Soc Work. 2008;33(3):211-220.
Hall MA, Dugan E, Zheng B, Mishra AK. Trust in physicians and medical institutions: what is it, can it be measured, and does it matter? Milbank Q. 2001;79(4):613-39, v.
Haywood C Jr, Williams-Reade J, Rushton C, Beach MC, Geller G. Improving Clinician Attitudes of Respect and Trust for Persons With Sickle Cell Disease. Hosp Pediatr. 2015 Jul;5(7):377-84.
Haywood C, Lanzkron S, Bediako S, et al. Perceived Discrimination, Patient Trust, and Adherence to Medical Recommendations Among Persons with Sickle Cell Disease. J Gen Intern Med. 2014;29(12):1657-1662. doi:10.1007/s11606-014-2986-7
Haywood C, Lanzkron S, Ratanawongsa N, Bediako SM, Lattimer-Nelson L, Beach MC. Hospital self-discharge among adults with sickle-cell disease (SCD): associations with trust and interpersonal experiences with care. J Hosp Med. 2010;5(5):289-294. doi:10.1002/jhm.643
Hoffman KM, Trawalter S, Axt JR, Oliver MN. Racial bias in pain assessment and treatment recommendations, and false beliefs about biological differences between blacks and whites. Proc Natl Acad Sci U S A. 2016 Apr 19;113(16):4296-301.
Jefferson T. Notes on the State of Virginia. London: Printed for John Stockdale; 1787
Kaiser Family Foundation. National Survey of Physicians Part I: Doctors on Disparities in Medical Care.; 2002.
Nelson SC, Hackman HW. Race matters: perceptions of race and racism in a sickle cell center. Pediatr Blood Cancer. 2013;60(3):451-454. doi:10.1002/pbc.24361
Washington H. Medical Apartheid: The Dark History of Medical Experimentation on Black Americans from Colonial Times to the Present. 1st ed. New York: Doubleday; 2006.
