# Supplementary material for: Health Equity Rounds: An Interdisciplinary Case Conference to Address Implicit Bias and Structural Racism for Faculty and Trainees
Source: MedEdPORTAL. 2019 Nov 22;15:10858. doi: 10.15766/mep_2374-8265.10858 (PMC7050660; doi:10.15766/mep_2374-8265.10858)
Supplement: Supplementary file 1 — A. HER 1.pptx B. HER 2.pptx C. HER 3.pptx D. HER 4.pptx E. HER 5.pptx F. HER 6.pptx G. HER 7.pptx H. Selected HER Handouts.docx I. Case Conference Creation Guide.docx J. Glossary.docx K. Evaluation.docx [file mep-15-10858-s001.zip › C. HER 3.pptx]

## Slide 1
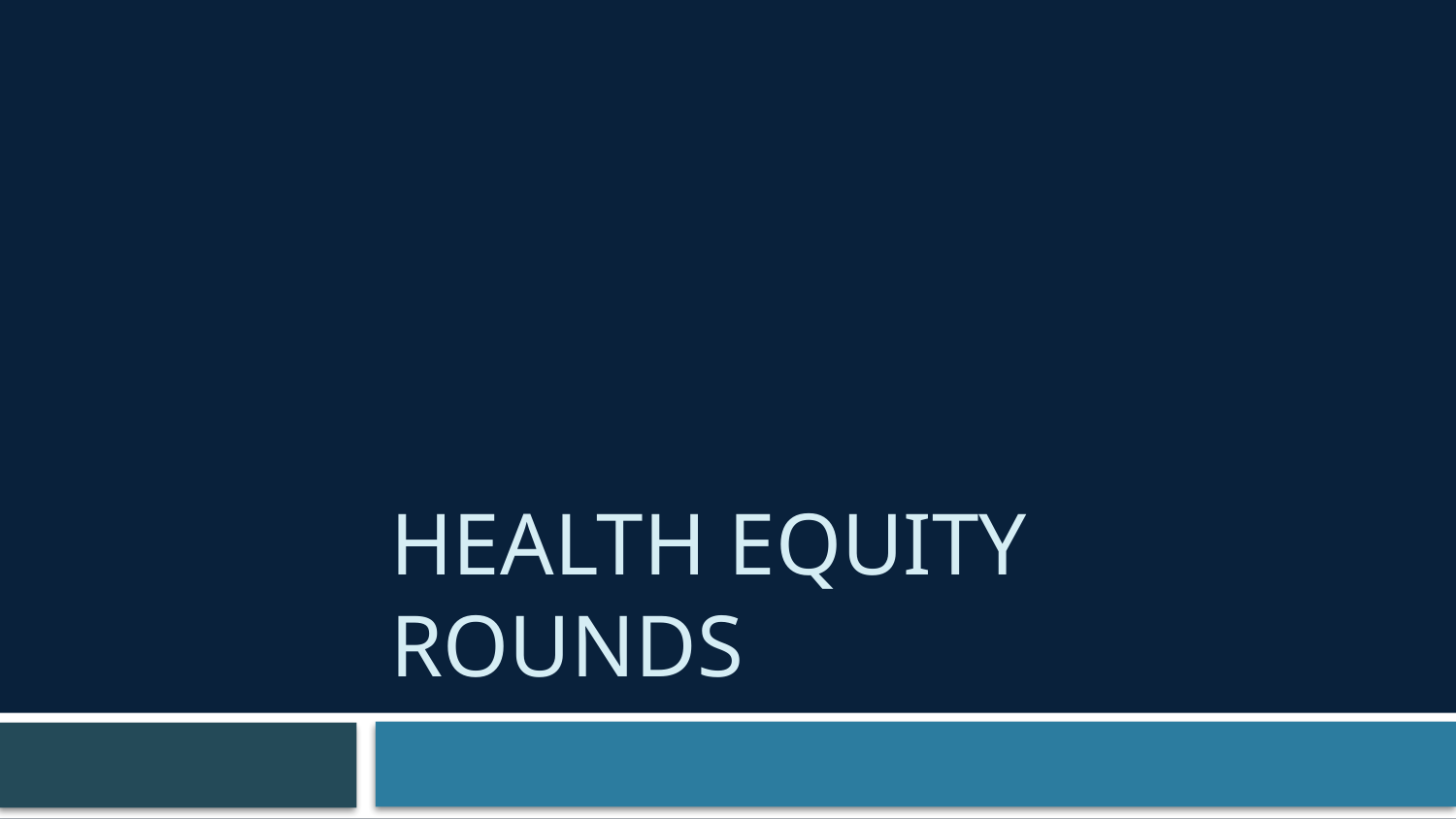

# Health equity rounds

## Slide 2
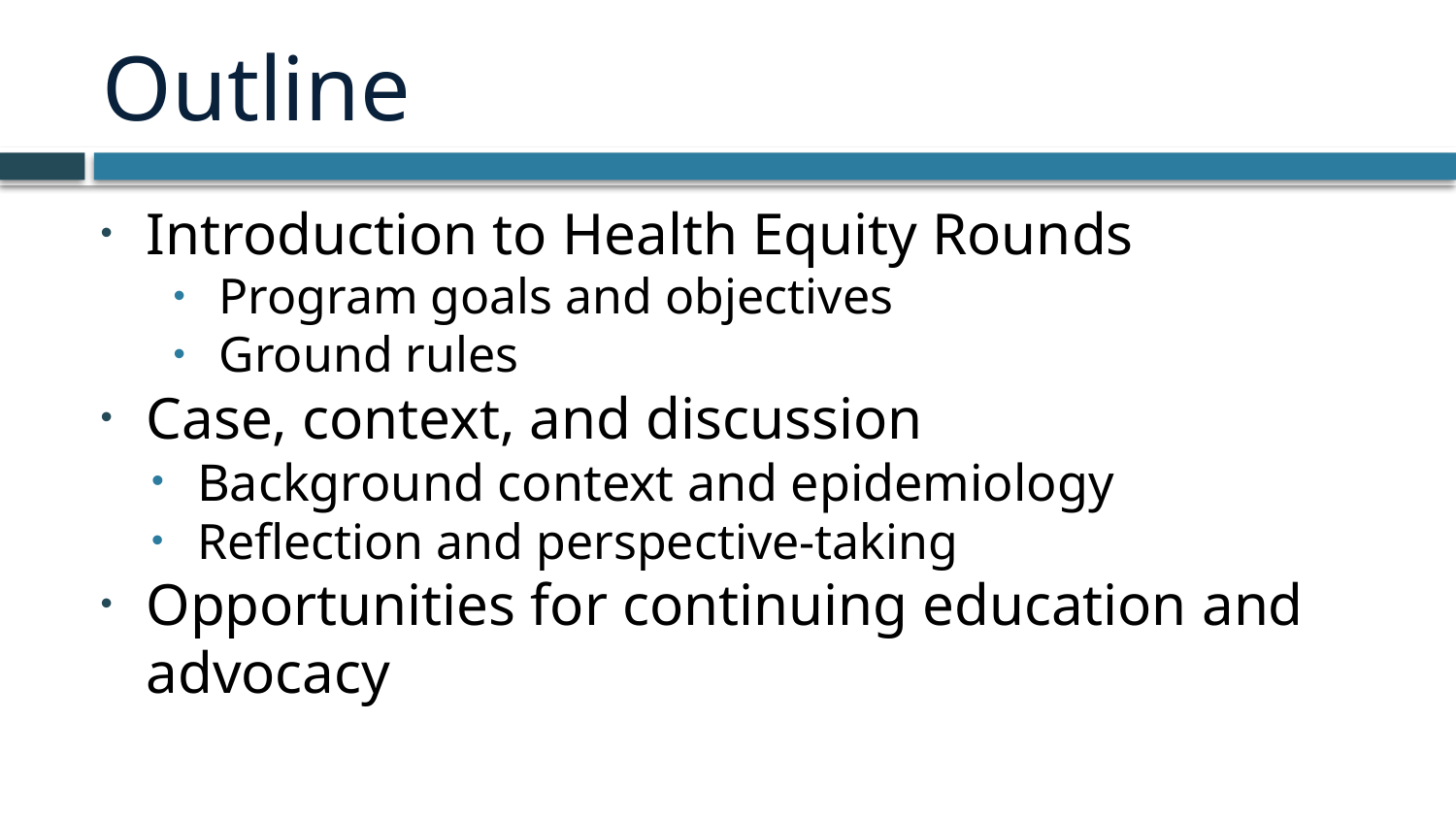

# Outline
Introduction to Health Equity Rounds
Program goals and objectives
Ground rules
Case, context, and discussion
Background context and epidemiology
Reflection and perspective-taking
Opportunities for continuing education and advocacy

## Slide 3
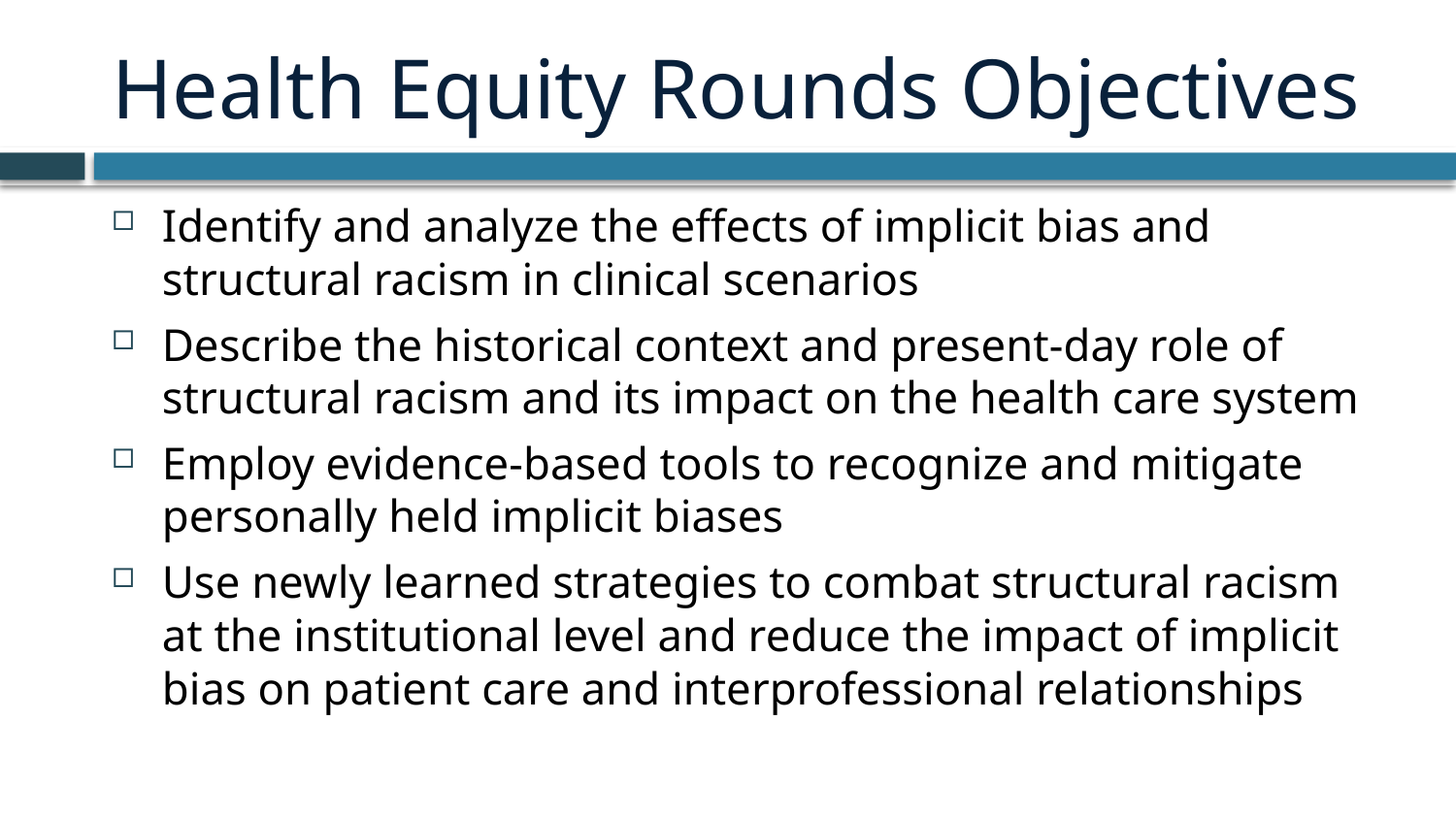

# Health Equity Rounds Objectives
Identify and analyze the effects of implicit bias and structural racism in clinical scenarios
Describe the historical context and present-day role of structural racism and its impact on the health care system
Employ evidence-based tools to recognize and mitigate personally held implicit biases
Use newly learned strategies to combat structural racism at the institutional level and reduce the impact of implicit bias on patient care and interprofessional relationships
Author Owned figure.
UCSF Office of Diversity and Outreach. Unconscious Bias. https://diversity.ucsf.edu/resources/unconscious-bias. Accessed January 21, 2019.

## Slide 4
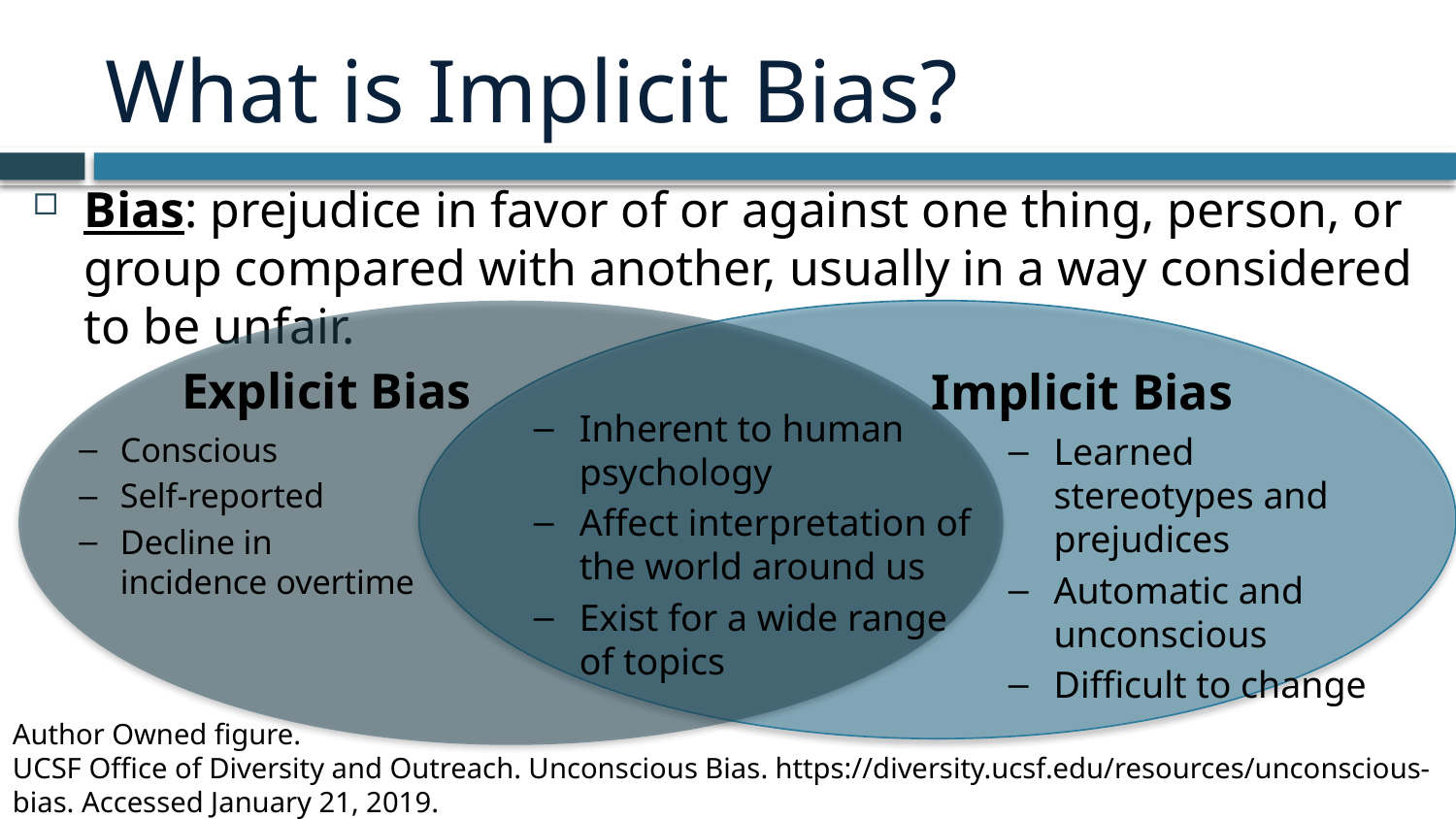

# What is Implicit Bias?
Bias: prejudice in favor of or against one thing, person, or group compared with another, usually in a way considered to be unfair.
Explicit Bias
Implicit Bias
Inherent to human psychology
Affect interpretation of the world around us
Exist for a wide range of topics
Learned stereotypes and prejudices
Automatic and unconscious
Difficult to change
Conscious
Self-reported
Decline in incidence overtime
Author Owned figure.
UCSF Office of Diversity and Outreach. Unconscious Bias. https://diversity.ucsf.edu/resources/unconscious-bias. Accessed January 21, 2019.

## Slide 5
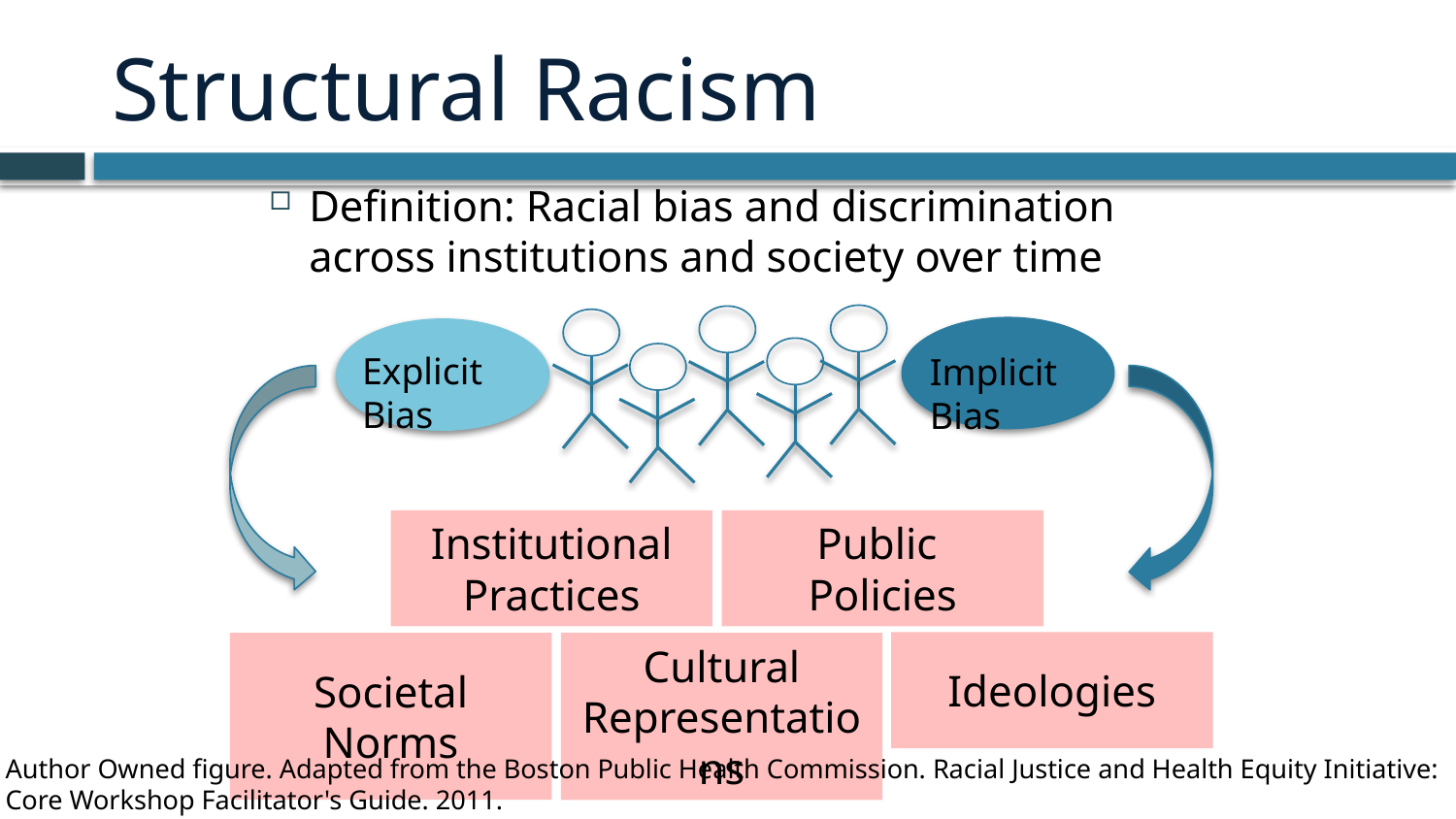

# Structural Racism
Definition: Racial bias and discrimination across institutions and society over time
Explicit Bias
Implicit Bias
Institutional Practices
Public
Policies
Ideologies
Societal Norms
Cultural
Representations
Author Owned figure. Adapted from the Boston Public Health Commission. Racial Justice and Health Equity Initiative: Core Workshop Facilitator's Guide. 2011.
Author Owned figure. Adapted from the Boston Public Health Commission. Racial Justice and Health Equity Initiative: Core Workshop Facilitator's Guide. 2011.
Author Owned figure. Adapted from the Boston Public Health Commission. Racial Justice and Health Equity Initiative: Core Workshop Facilitator's Guide. 2011.
Author Owned figure. Adapted from the Boston Public Health Commission. Racial Justice and Health Equity Initiative: Core Workshop Facilitator's Guide. 2011.
Author Owned figure. Adapted from the Boston Public Health Commission. Racial Justice and Health Equity Initiative: Core Workshop Facilitator's Guide. 2011.
Author Owned figure. Adapted from the Boston Public Health Commission. Racial Justice and Health Equity Initiative: Core Workshop Facilitator's Guide. 2011.

## Slide 6
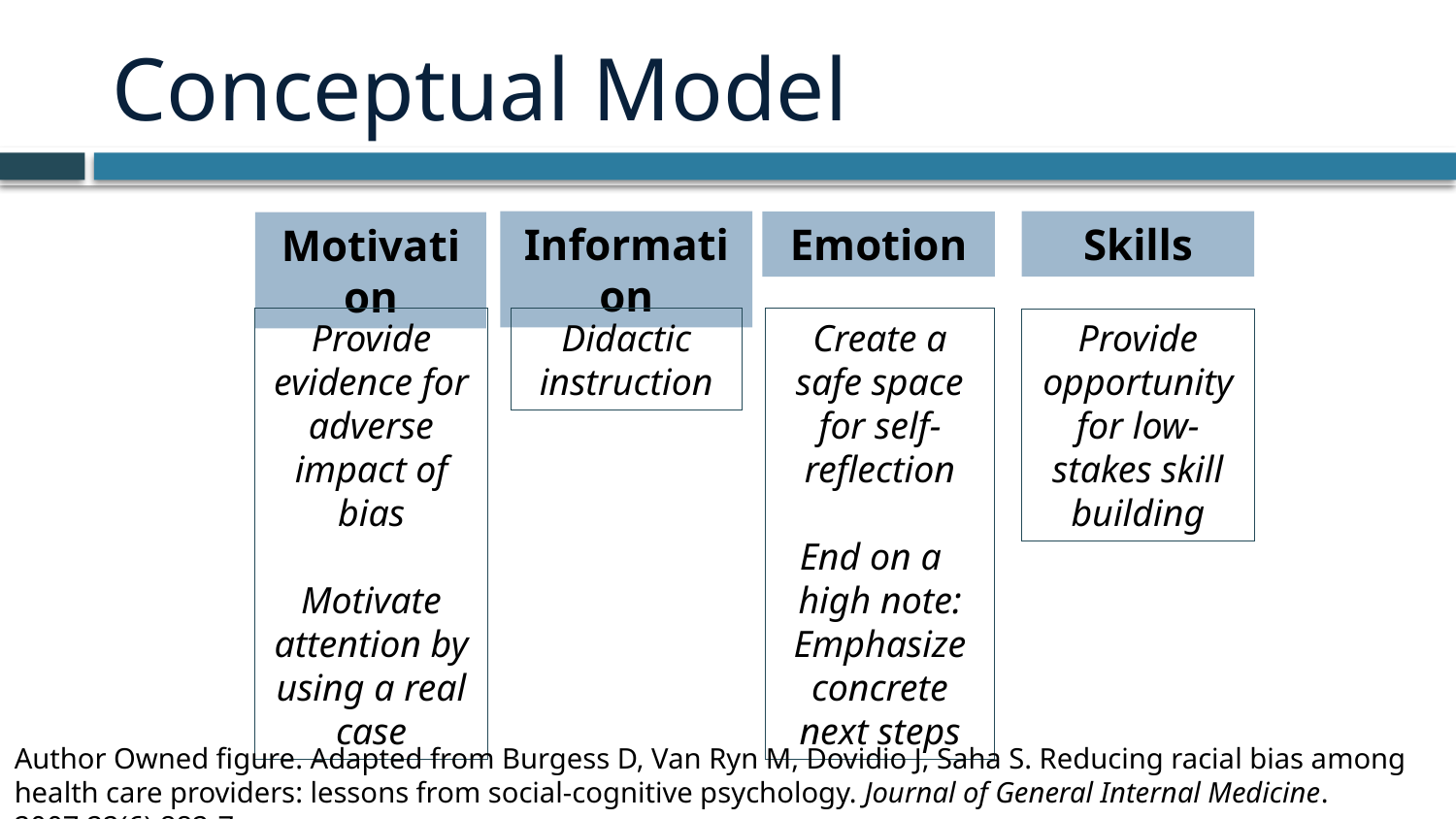

# Conceptual Model
Information
Skills
Emotion
Motivation
Create a safe space for self-reflection
d
End on a high note: Emphasize concrete next steps
Provide evidence for adverse impact of bias
Motivate attention by using a real case
Didactic instruction
Provide opportunity for low-stakes skill building
Author Owned figure. Adapted from Burgess D, Van Ryn M, Dovidio J, Saha S. Reducing racial bias among health care providers: lessons from social-cognitive psychology. Journal of General Internal Medicine. 2007;22(6):882-7.

## Slide 7
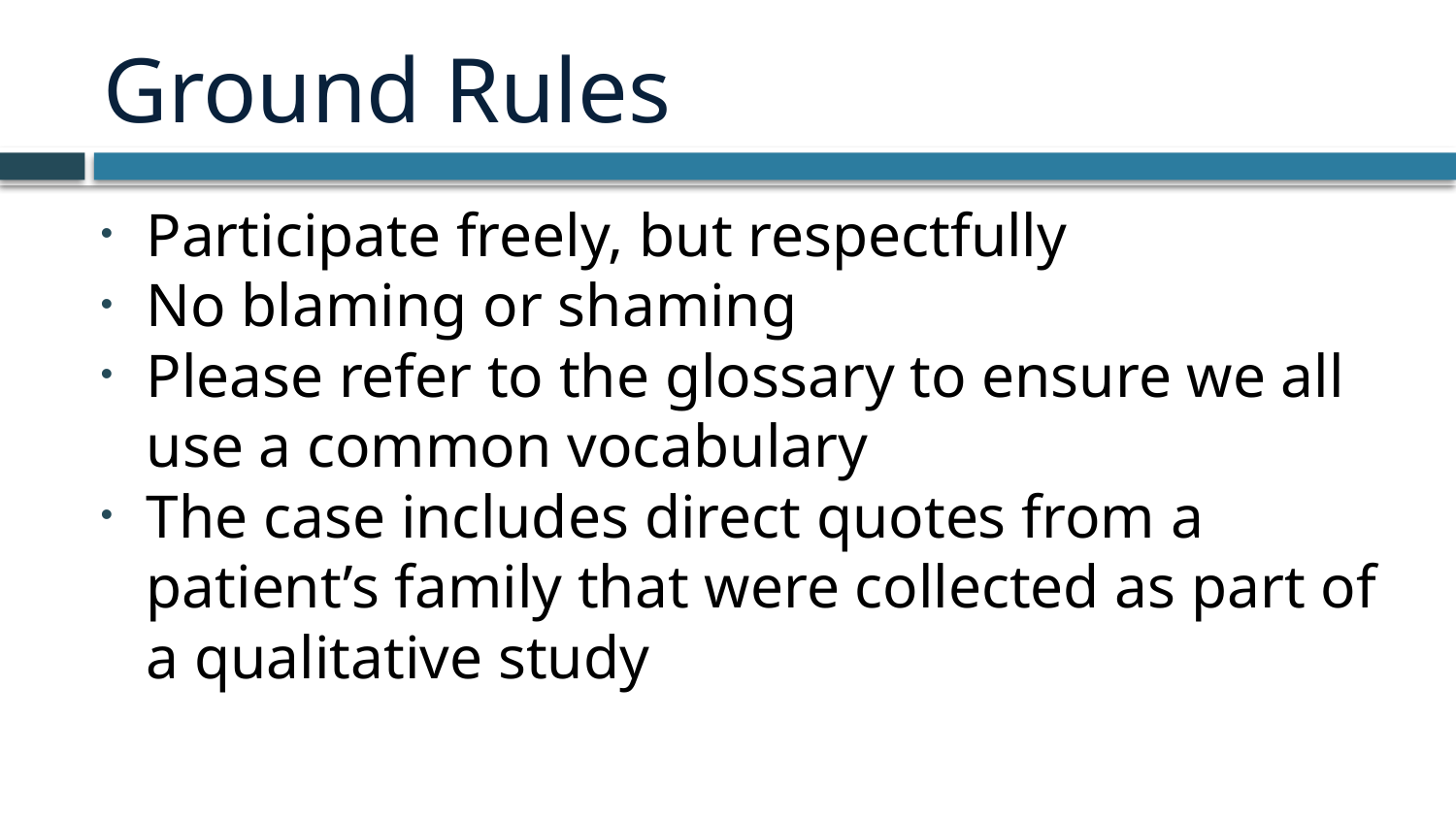

# Ground Rules
Participate freely, but respectfully
No blaming or shaming
Please refer to the glossary to ensure we all use a common vocabulary
The case includes direct quotes from a patient’s family that were collected as part of a qualitative study

## Slide 8
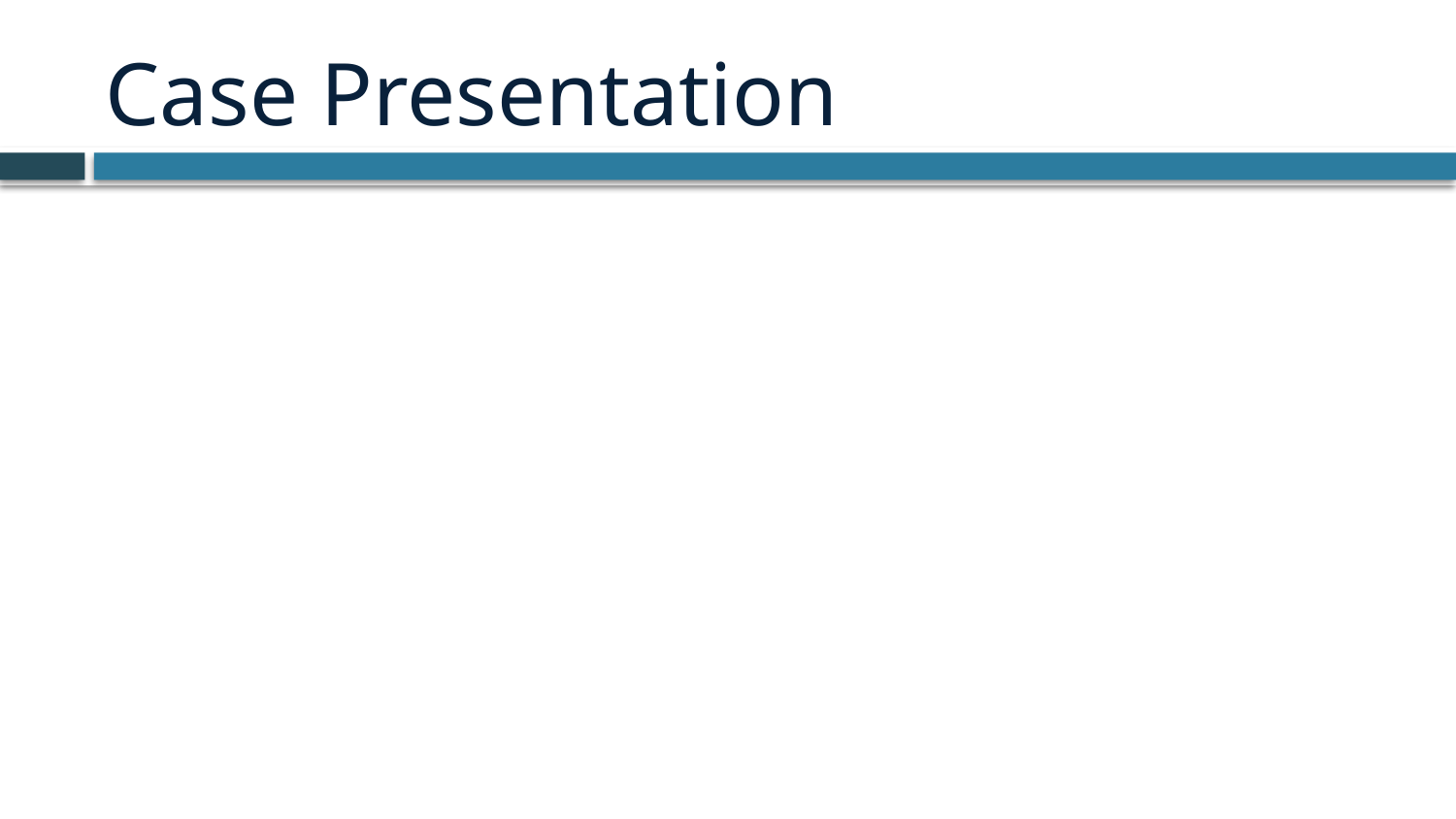

# Case Presentation

## Slide 9
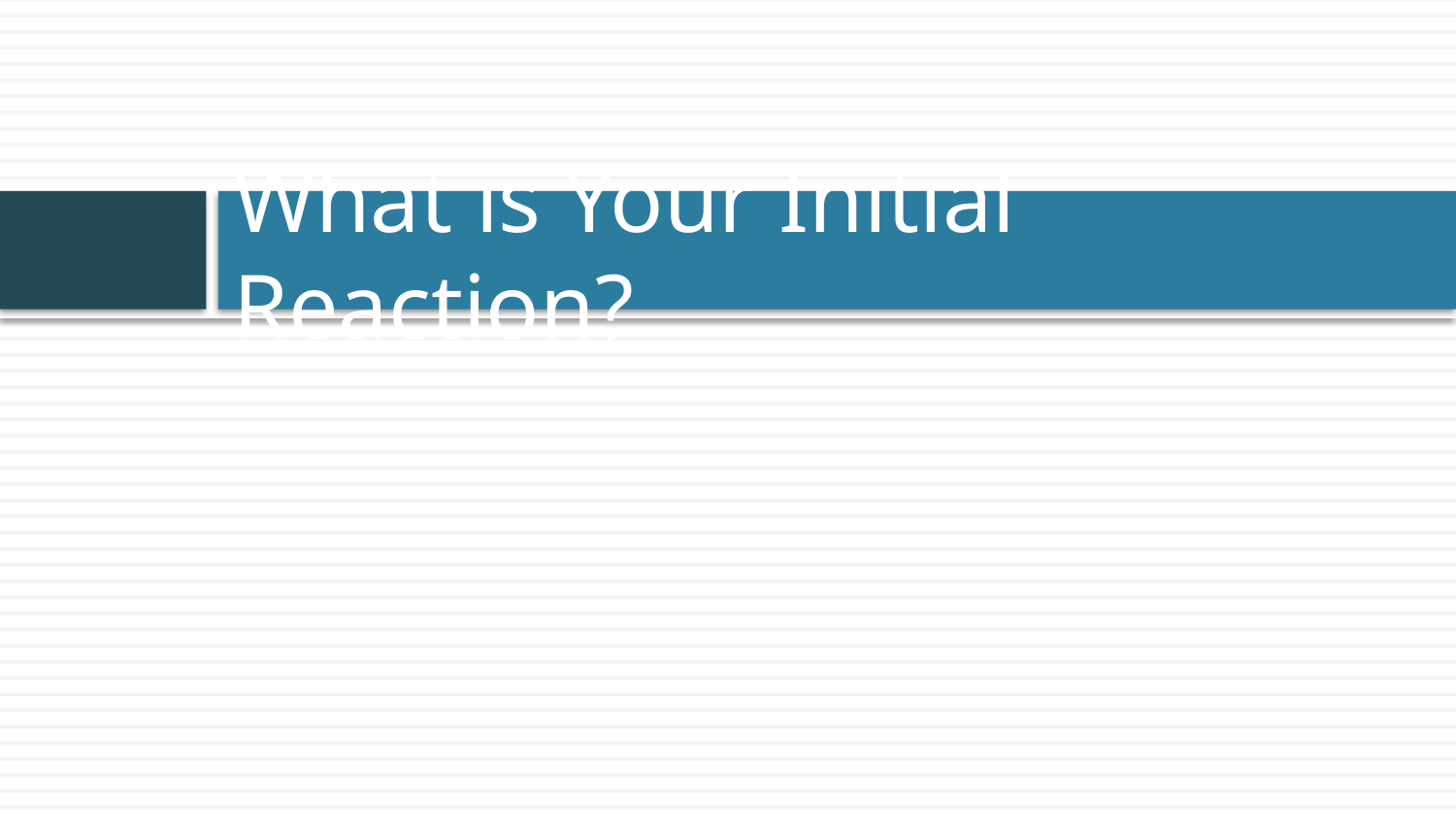

# What is Your Initial Reaction?

## Slide 10
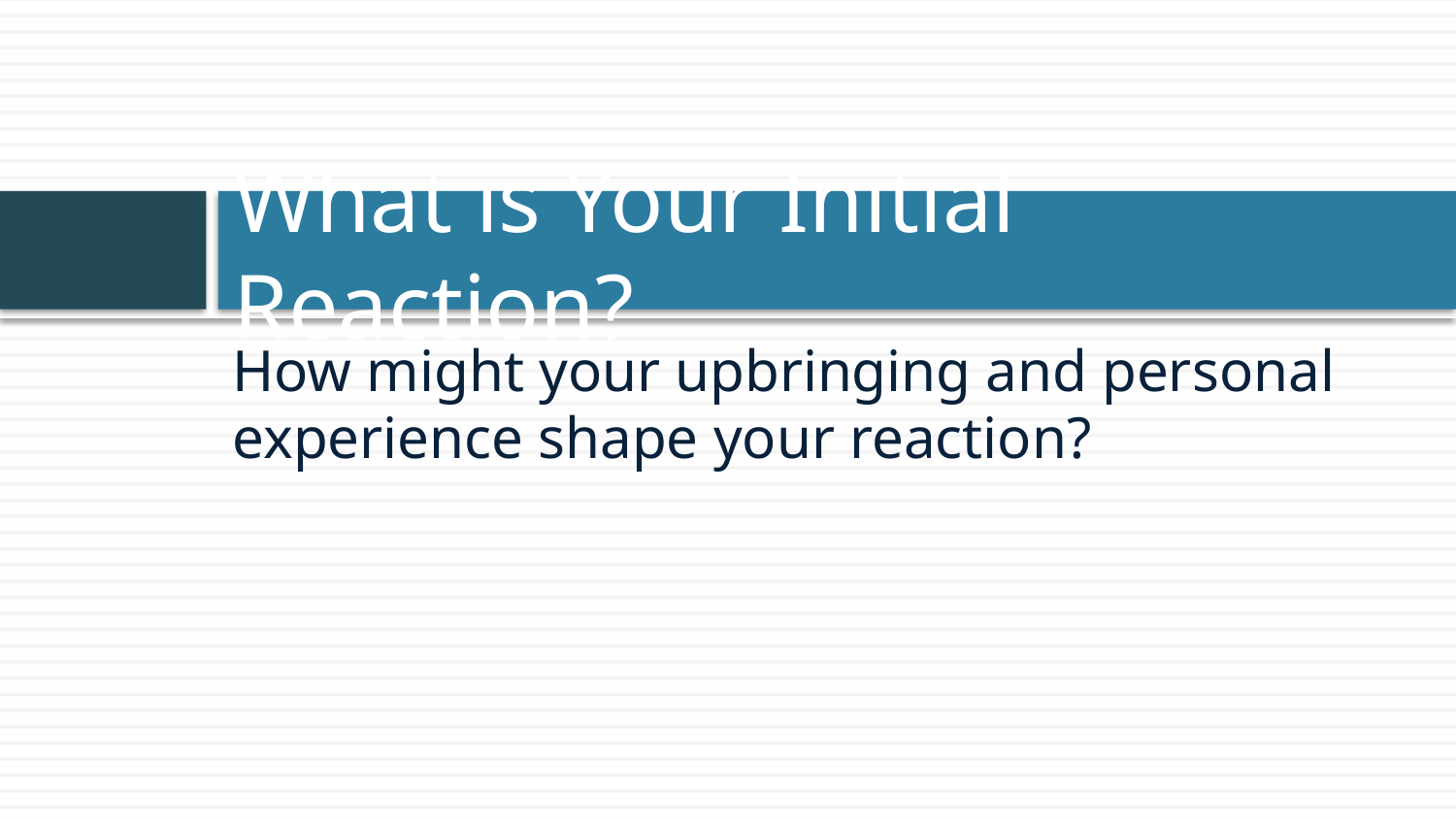

# What is Your Initial Reaction?
How might your upbringing and personal experience shape your reaction?

## Slide 11
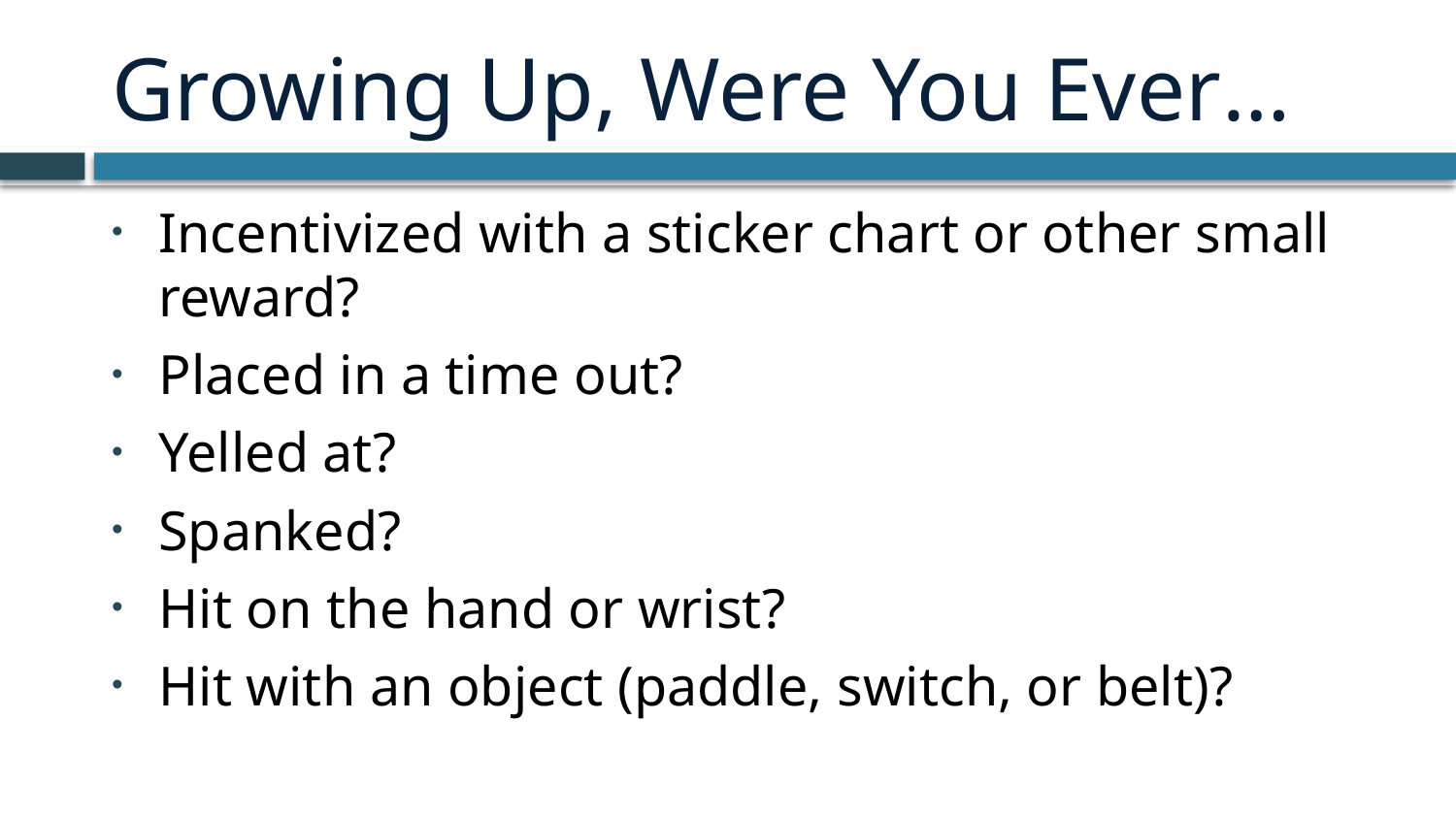

# Growing Up, Were You Ever…
Incentivized with a sticker chart or other small reward?
Placed in a time out?
Yelled at?
Spanked?
Hit on the hand or wrist?
Hit with an object (paddle, switch, or belt)?

## Slide 12
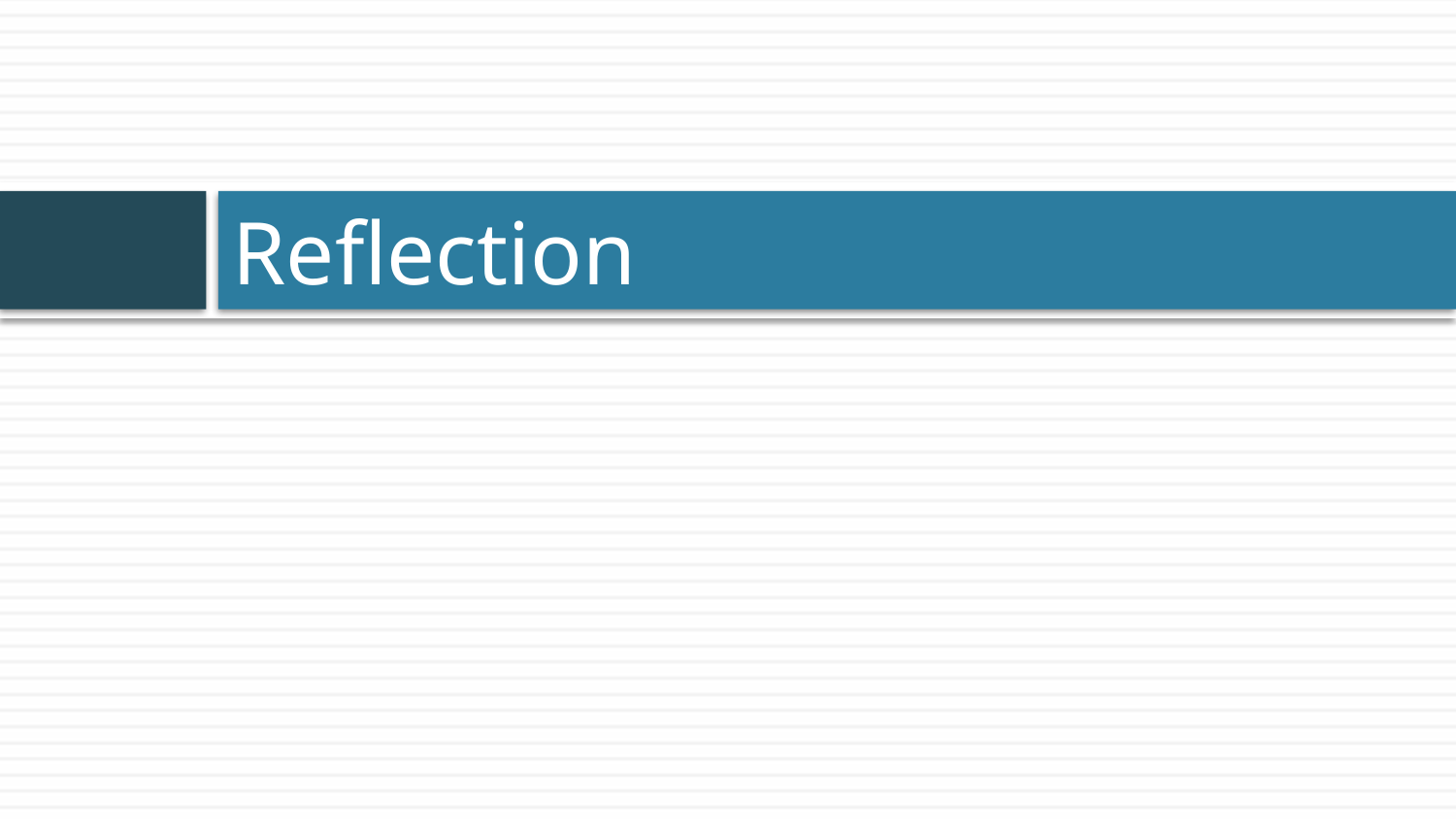

# Reflection

## Slide 13
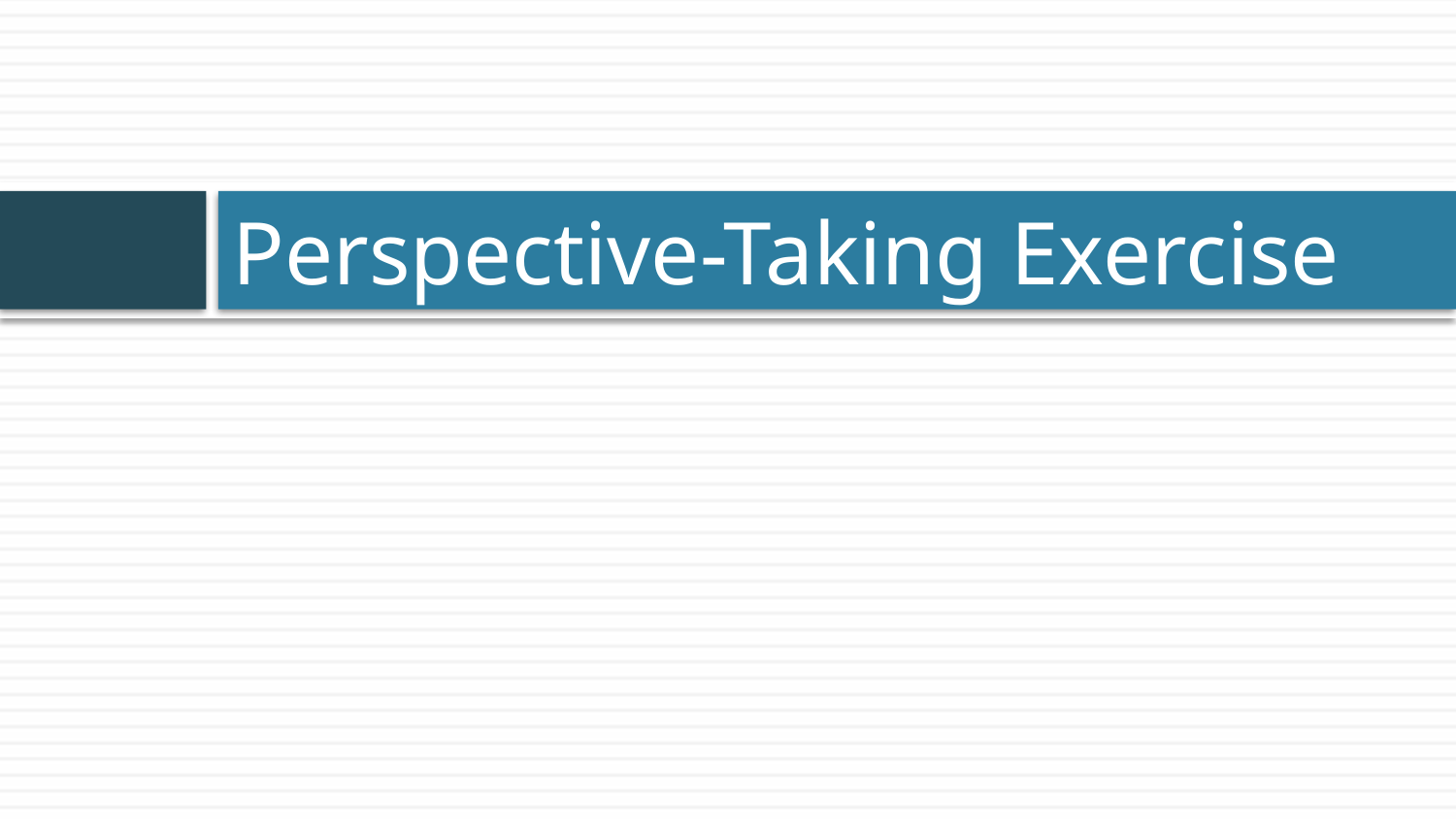

# Perspective-Taking Exercise

## Slide 14
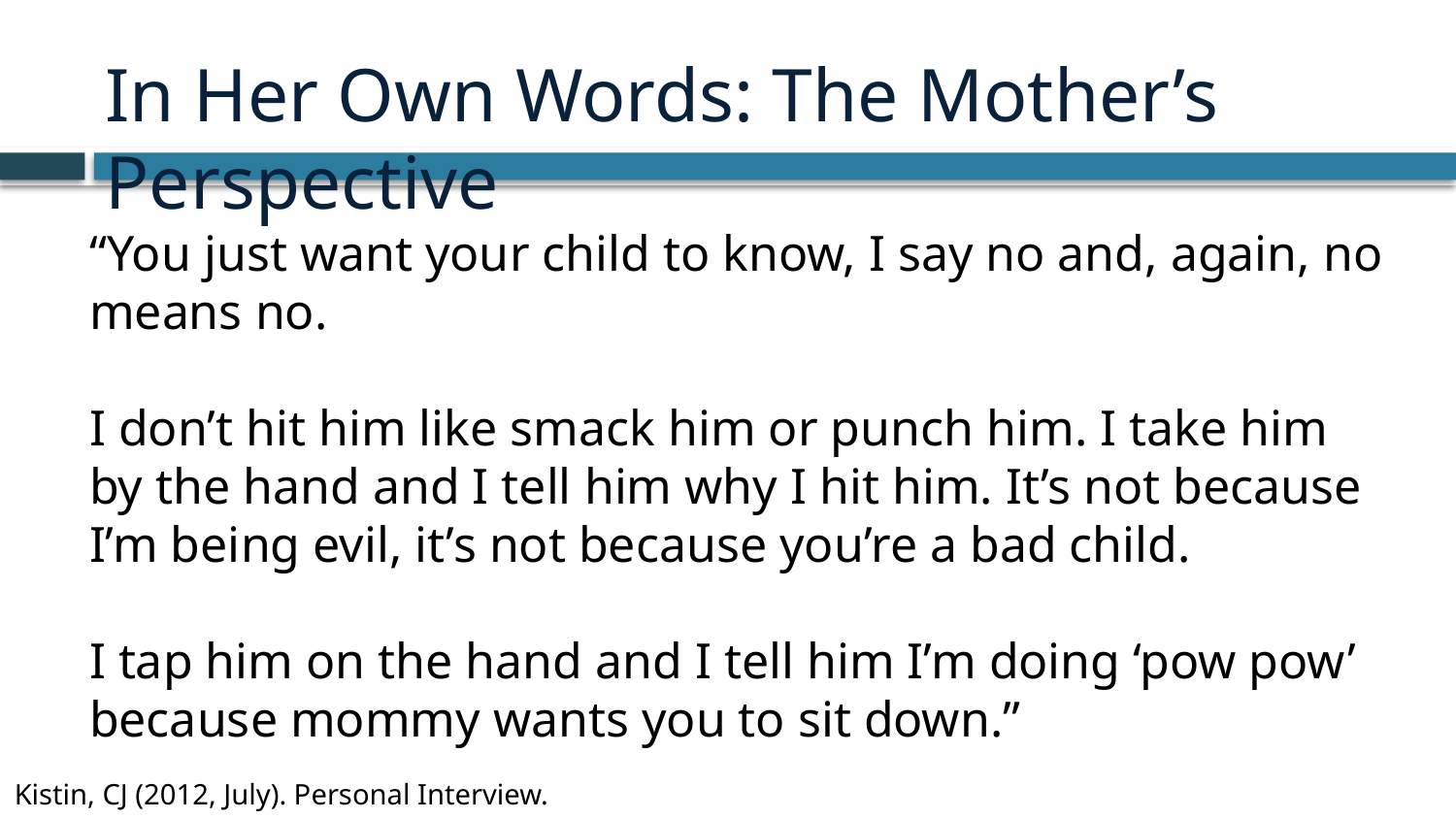

# In Her Own Words: The Mother’s Perspective
“You just want your child to know, I say no and, again, no means no.
I don’t hit him like smack him or punch him. I take him by the hand and I tell him why I hit him. It’s not because I’m being evil, it’s not because you’re a bad child.
I tap him on the hand and I tell him I’m doing ‘pow pow’ because mommy wants you to sit down.”
Kistin, CJ (2012, July). Personal Interview.

## Slide 15
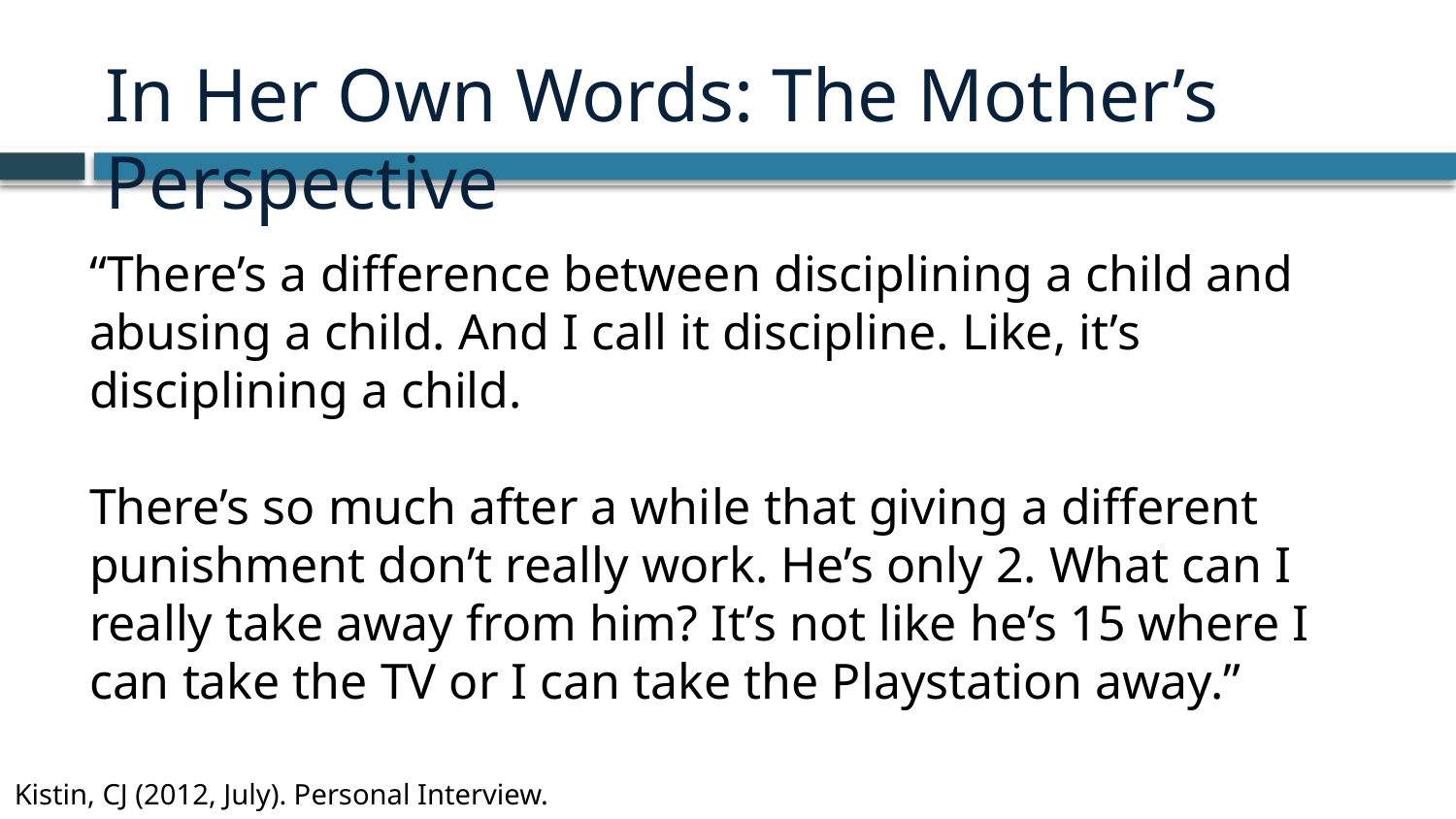

# In Her Own Words: The Mother’s Perspective
“There’s a difference between disciplining a child and abusing a child. And I call it discipline. Like, it’s disciplining a child.
There’s so much after a while that giving a different punishment don’t really work. He’s only 2. What can I really take away from him? It’s not like he’s 15 where I can take the TV or I can take the Playstation away.”
Kistin, CJ (2012, July). Personal Interview.

## Slide 16
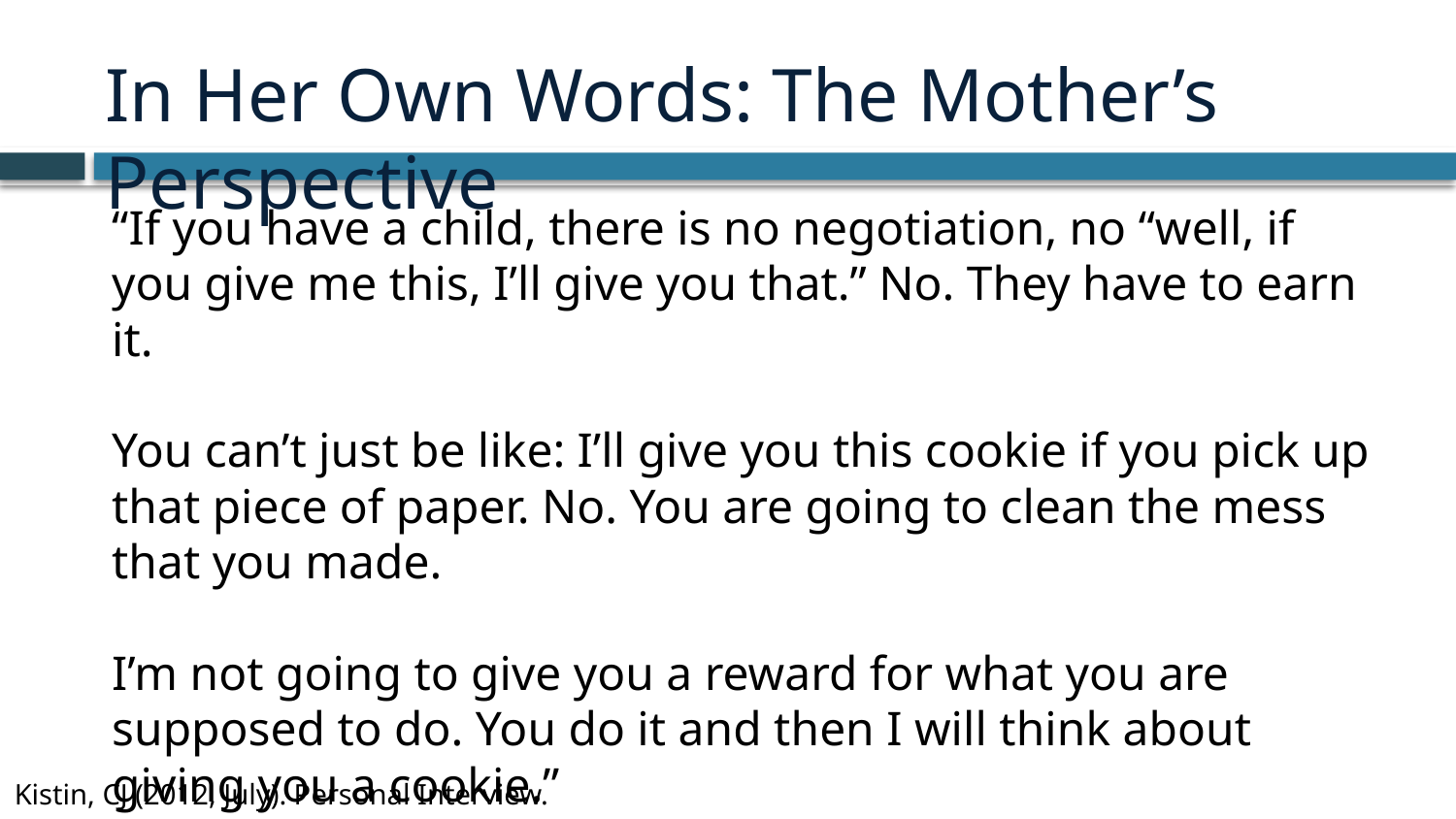

# In Her Own Words: The Mother’s Perspective
“If you have a child, there is no negotiation, no “well, if you give me this, I’ll give you that.” No. They have to earn it.
You can’t just be like: I’ll give you this cookie if you pick up that piece of paper. No. You are going to clean the mess that you made.
I’m not going to give you a reward for what you are supposed to do. You do it and then I will think about giving you a cookie.”
Kistin, CJ (2012, July). Personal Interview.

## Slide 17
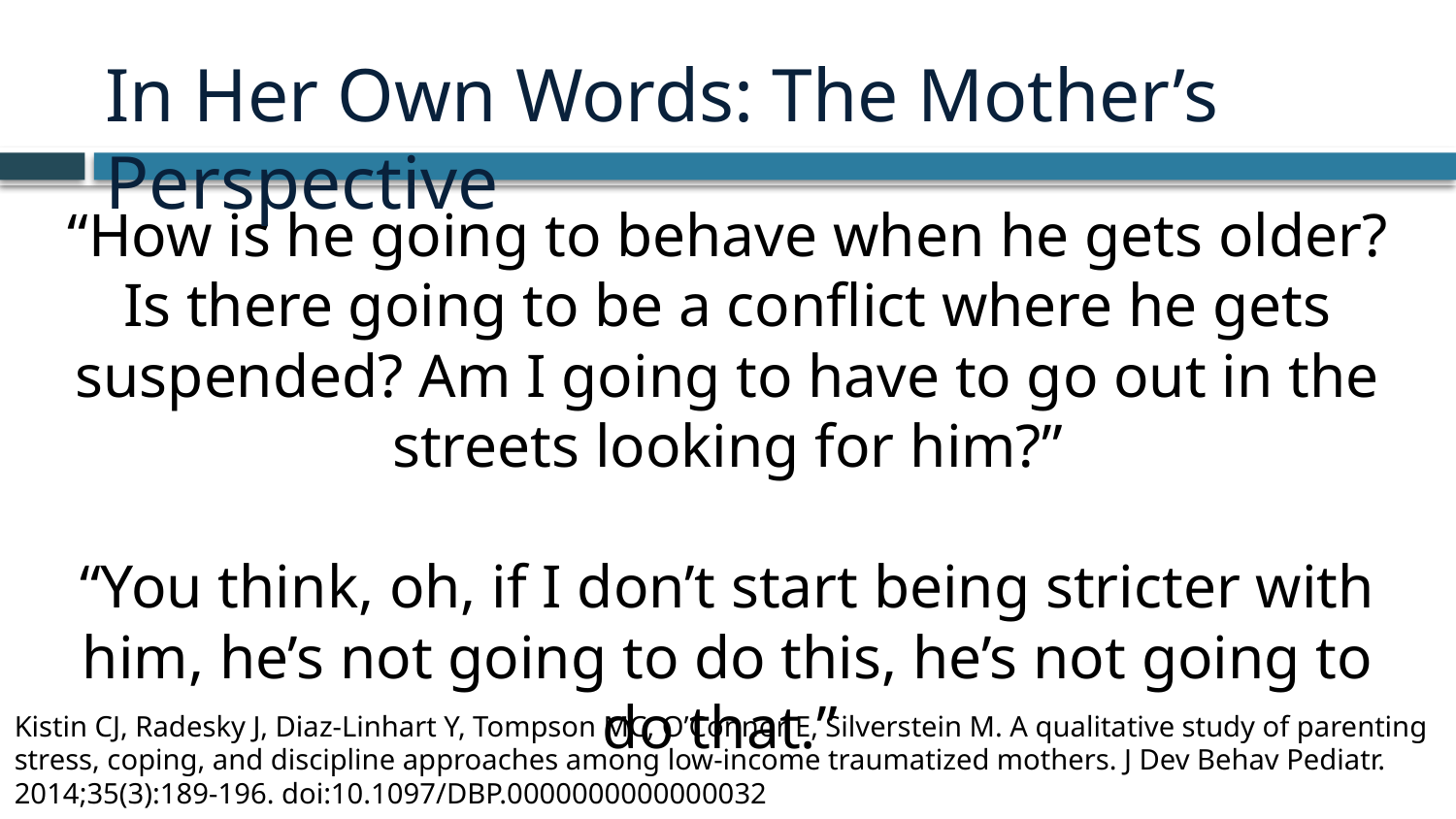

# In Her Own Words: The Mother’s Perspective
“How is he going to behave when he gets older? Is there going to be a conflict where he gets suspended? Am I going to have to go out in the streets looking for him?”
“You think, oh, if I don’t start being stricter with him, he’s not going to do this, he’s not going to do that.”
Kistin CJ, Radesky J, Diaz-Linhart Y, Tompson MC, OʼConnor E, Silverstein M. A qualitative study of parenting stress, coping, and discipline approaches among low-income traumatized mothers. J Dev Behav Pediatr. 2014;35(3):189-196. doi:10.1097/DBP.0000000000000032

## Slide 18
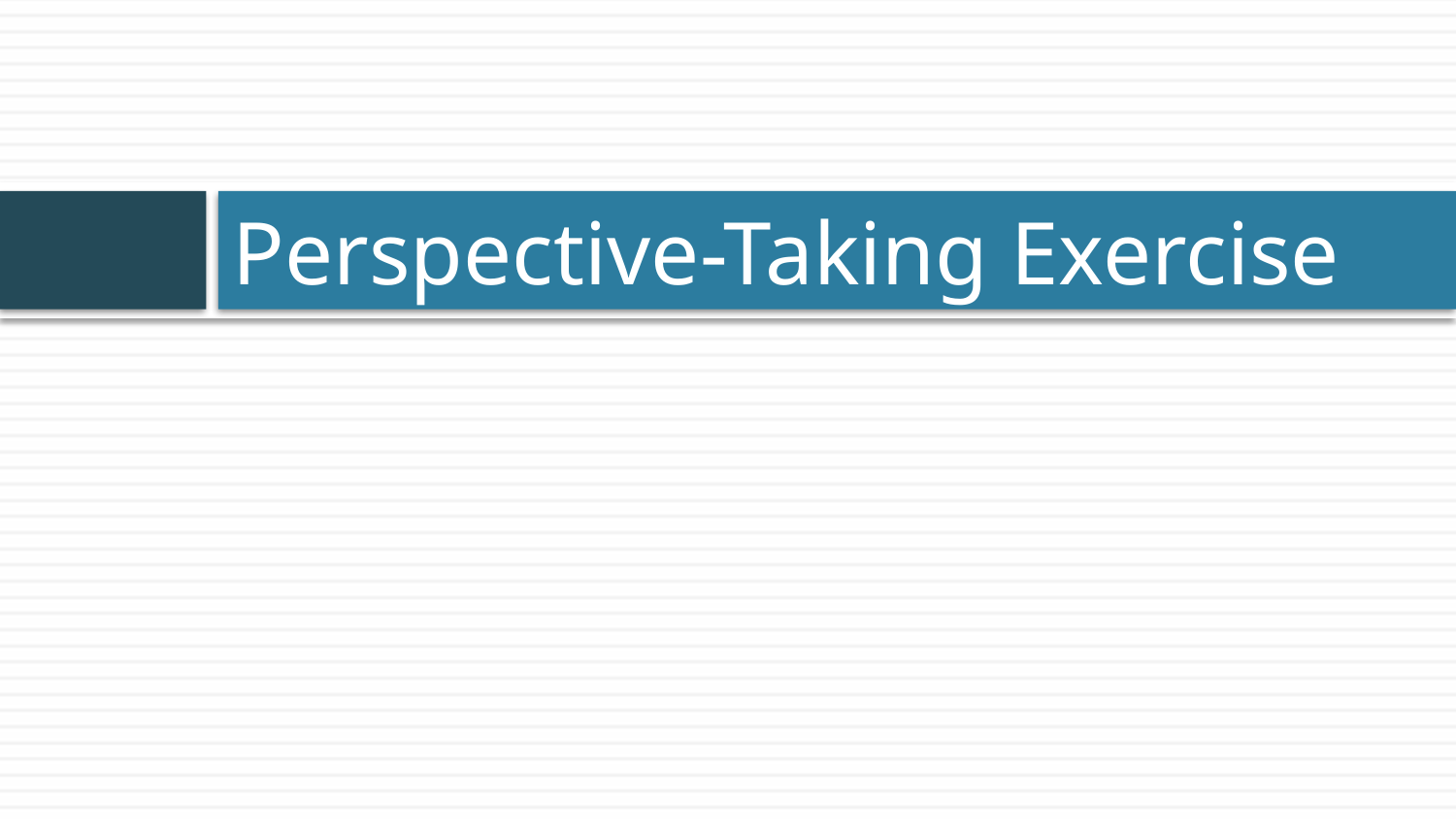

# Perspective-Taking Exercise

## Slide 19
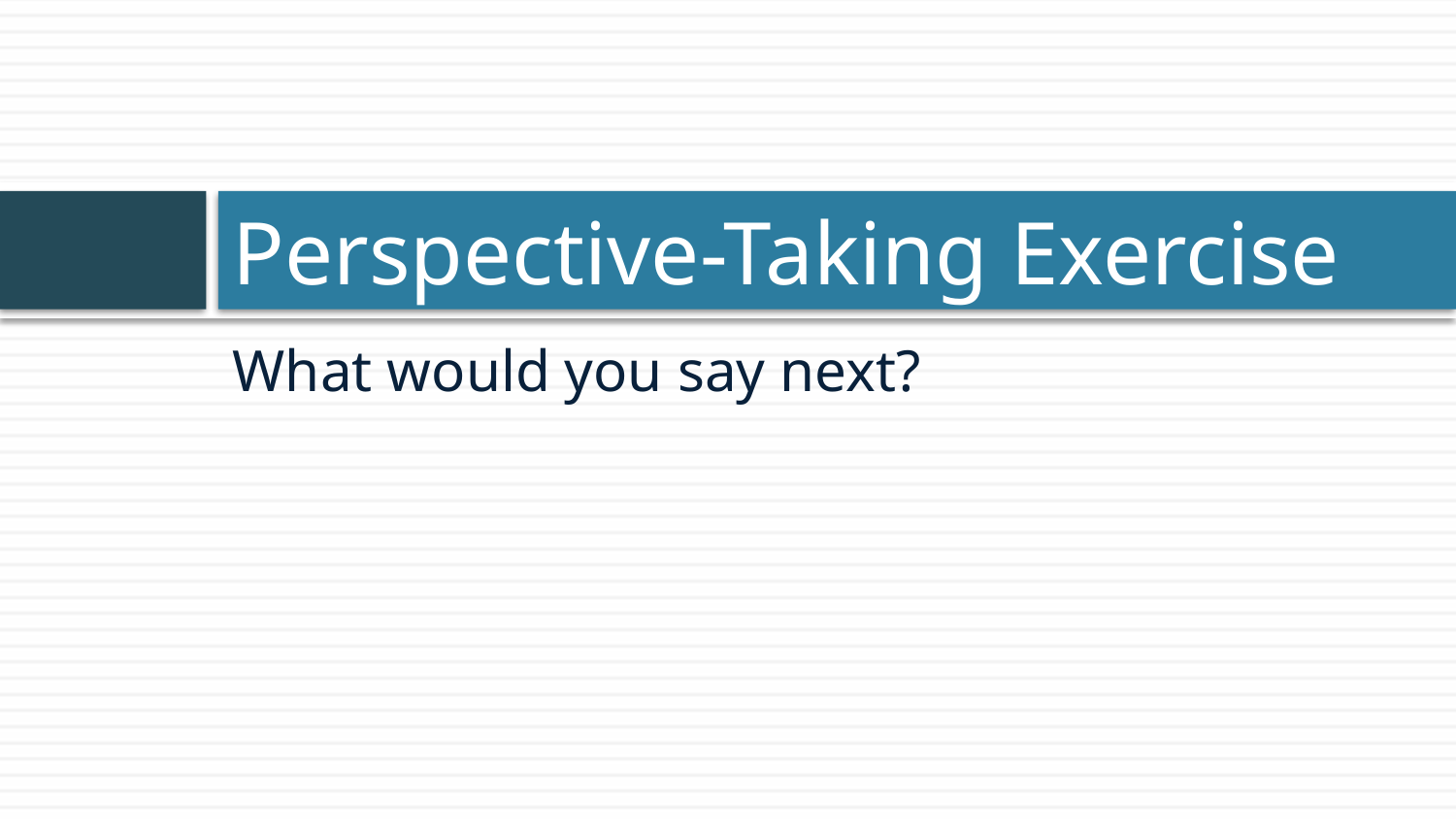

# Perspective-Taking Exercise
What would you say next?

## Slide 20
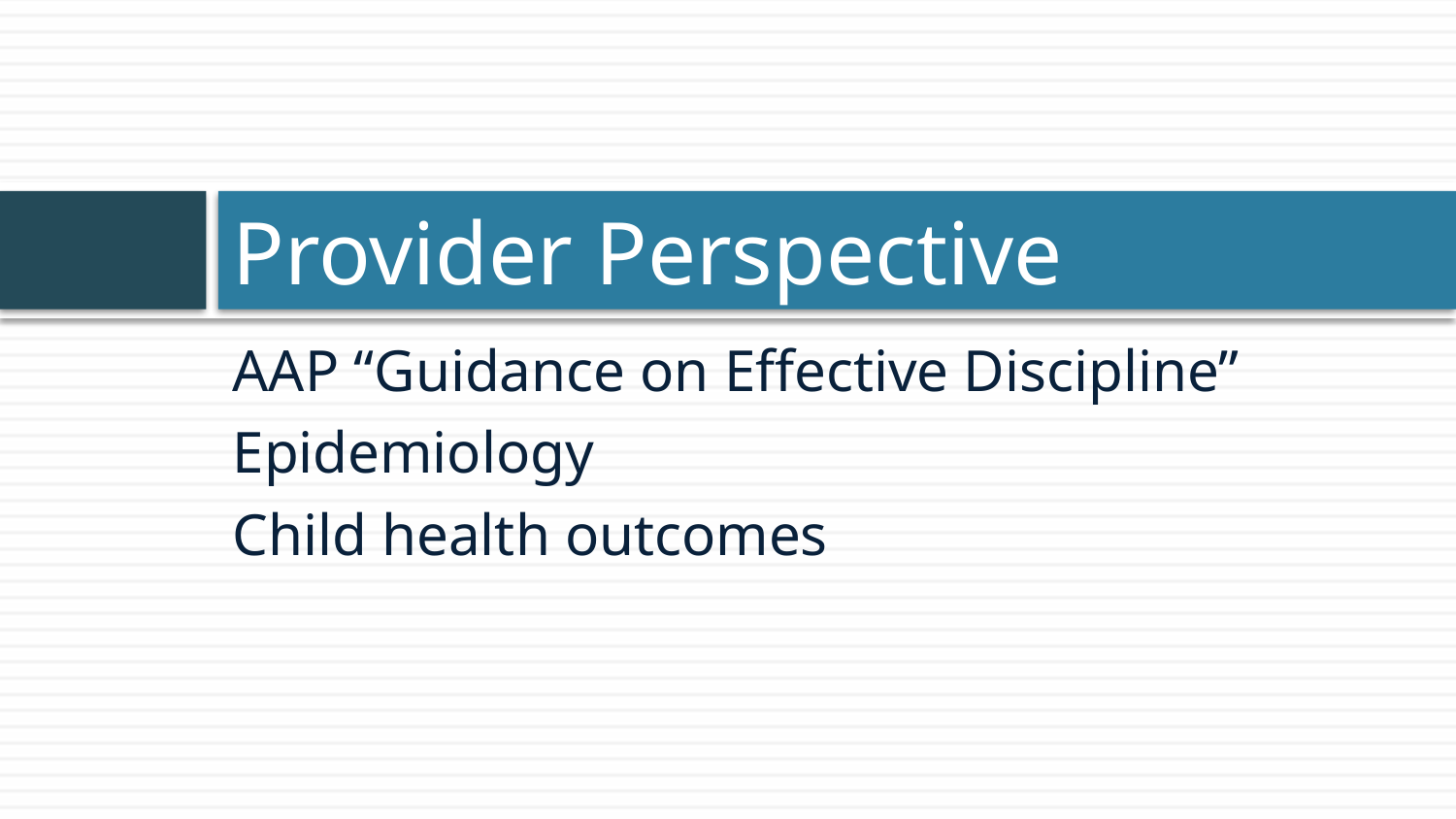

# Provider Perspective
AAP “Guidance on Effective Discipline”
Epidemiology
Child health outcomes

## Slide 21
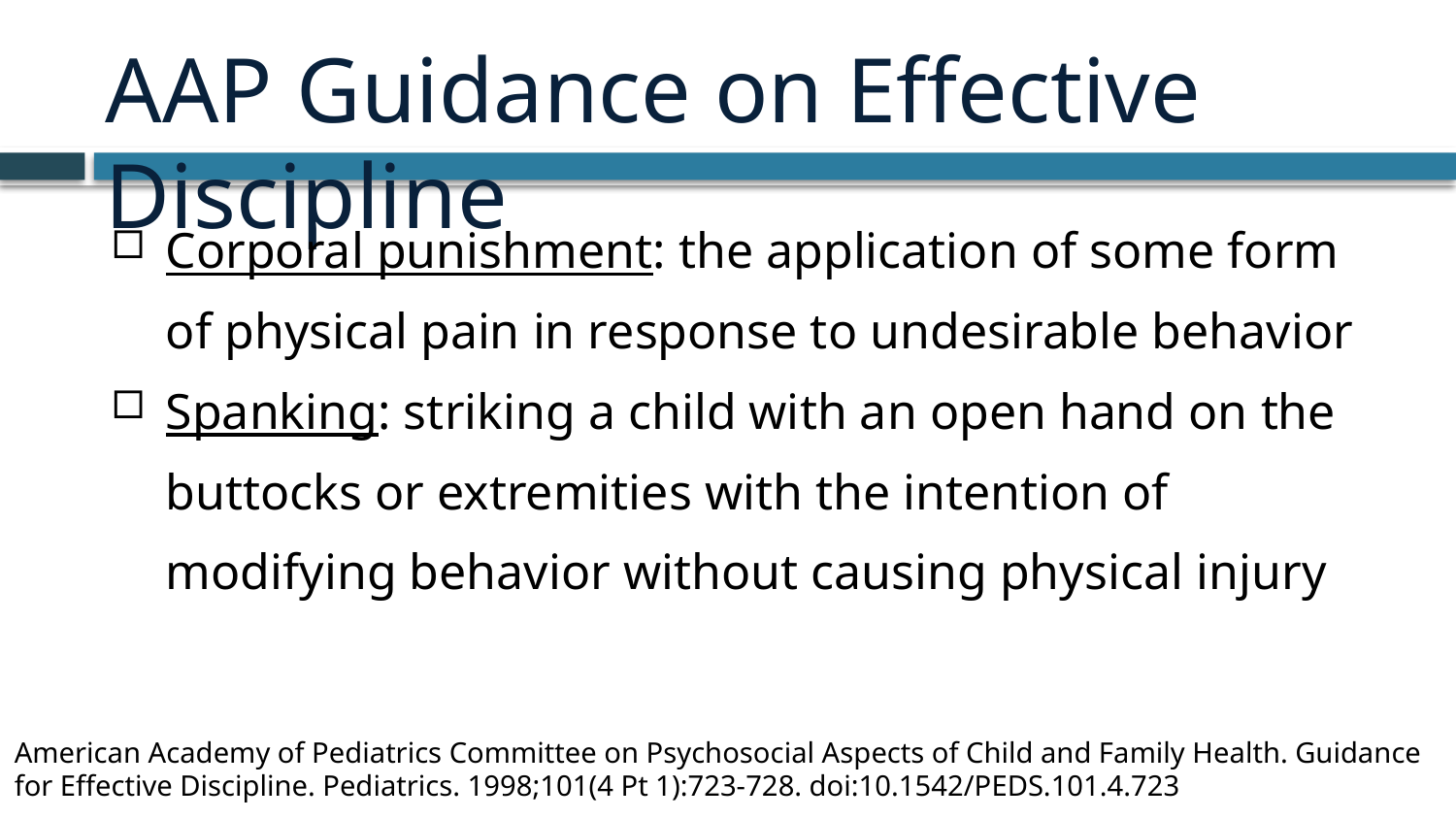

# AAP Guidance on Effective Discipline
Corporal punishment: the application of some form of physical pain in response to undesirable behavior
Spanking: striking a child with an open hand on the buttocks or extremities with the intention of modifying behavior without causing physical injury
American Academy of Pediatrics Committee on Psychosocial Aspects of Child and Family Health. Guidance for Effective Discipline. Pediatrics. 1998;101(4 Pt 1):723-728. doi:10.1542/PEDS.101.4.723

## Slide 22
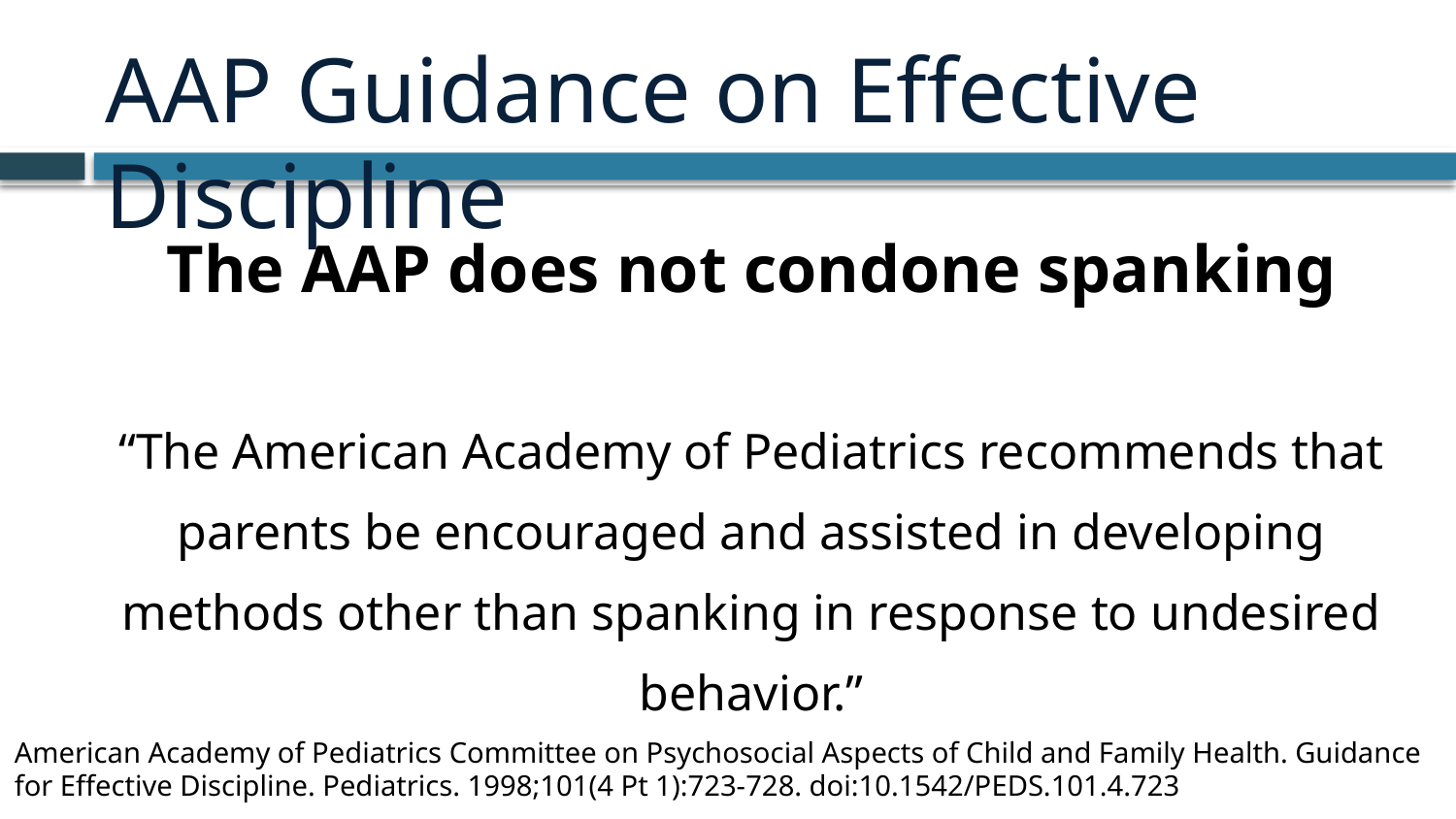

# AAP Guidance on Effective Discipline
The AAP does not condone spanking
“The American Academy of Pediatrics recommends that parents be encouraged and assisted in developing methods other than spanking in response to undesired behavior.”
American Academy of Pediatrics Committee on Psychosocial Aspects of Child and Family Health. Guidance for Effective Discipline. Pediatrics. 1998;101(4 Pt 1):723-728. doi:10.1542/PEDS.101.4.723

## Slide 23
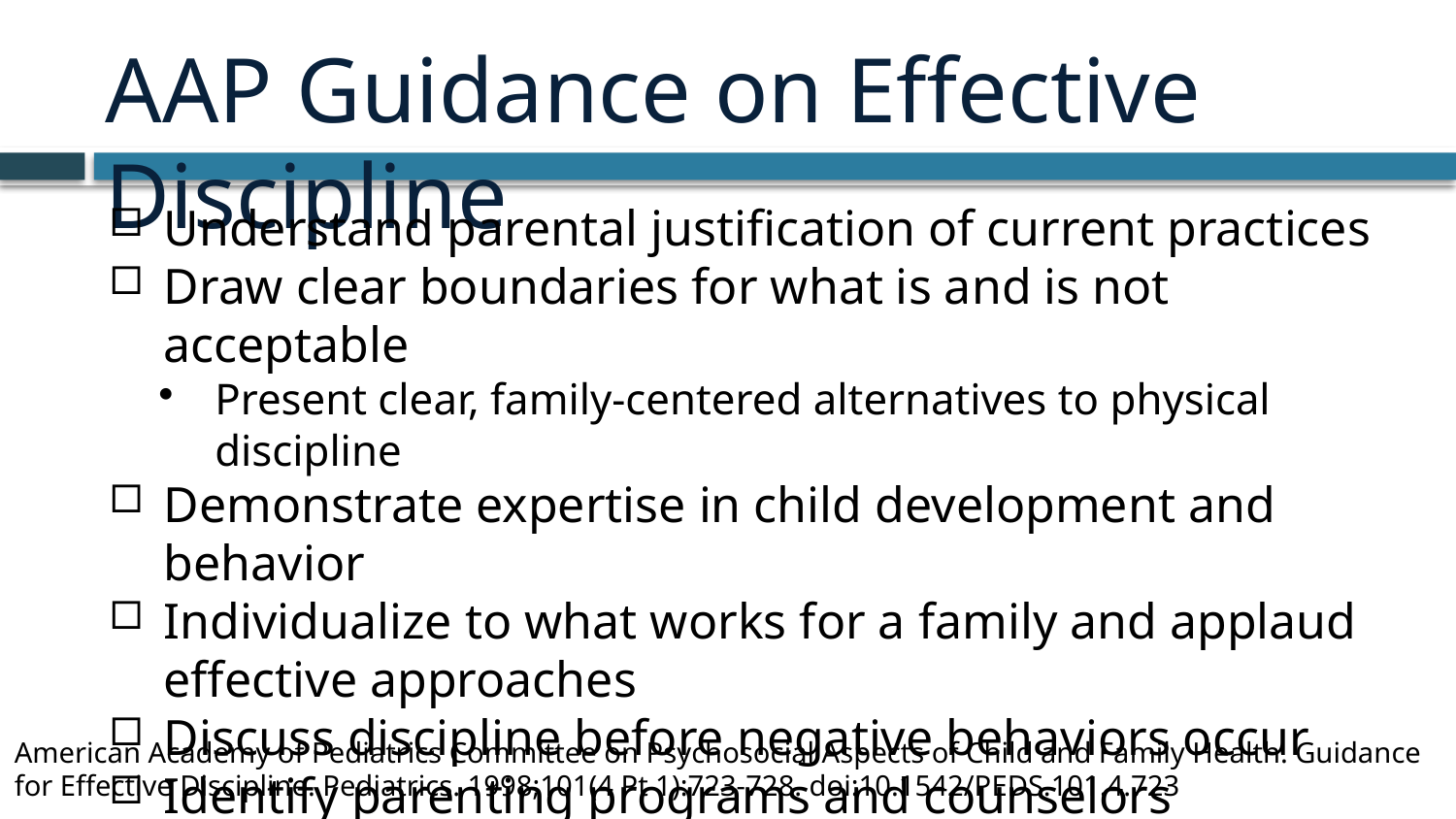

# AAP Guidance on Effective Discipline
Understand parental justification of current practices
Draw clear boundaries for what is and is not acceptable
Present clear, family-centered alternatives to physical discipline
Demonstrate expertise in child development and behavior
Individualize to what works for a family and applaud effective approaches
Discuss discipline before negative behaviors occur
Identify parenting programs and counselors
Participate in public education and advocacy
American Academy of Pediatrics Committee on Psychosocial Aspects of Child and Family Health. Guidance for Effective Discipline. Pediatrics. 1998;101(4 Pt 1):723-728. doi:10.1542/PEDS.101.4.723

## Slide 24
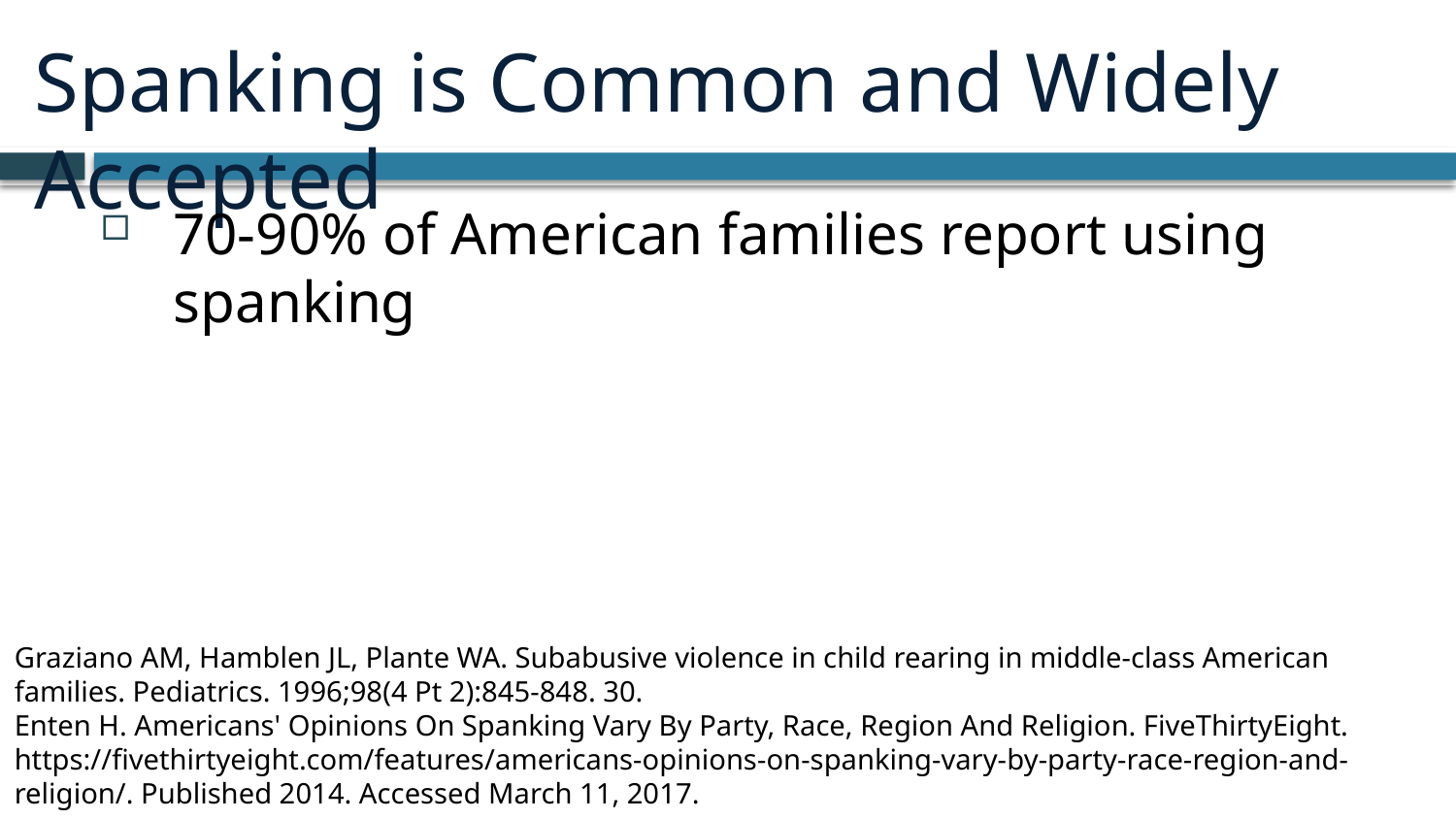

# Spanking is Common and Widely Accepted
70-90% of American families report using spanking
Graziano AM, Hamblen JL, Plante WA. Subabusive violence in child rearing in middle-class American families. Pediatrics. 1996;98(4 Pt 2):845-848. 30.
Enten H. Americans' Opinions On Spanking Vary By Party, Race, Region And Religion. FiveThirtyEight. https://fivethirtyeight.com/features/americans-opinions-on-spanking-vary-by-party-race-region-and-religion/. Published 2014. Accessed March 11, 2017.

## Slide 25
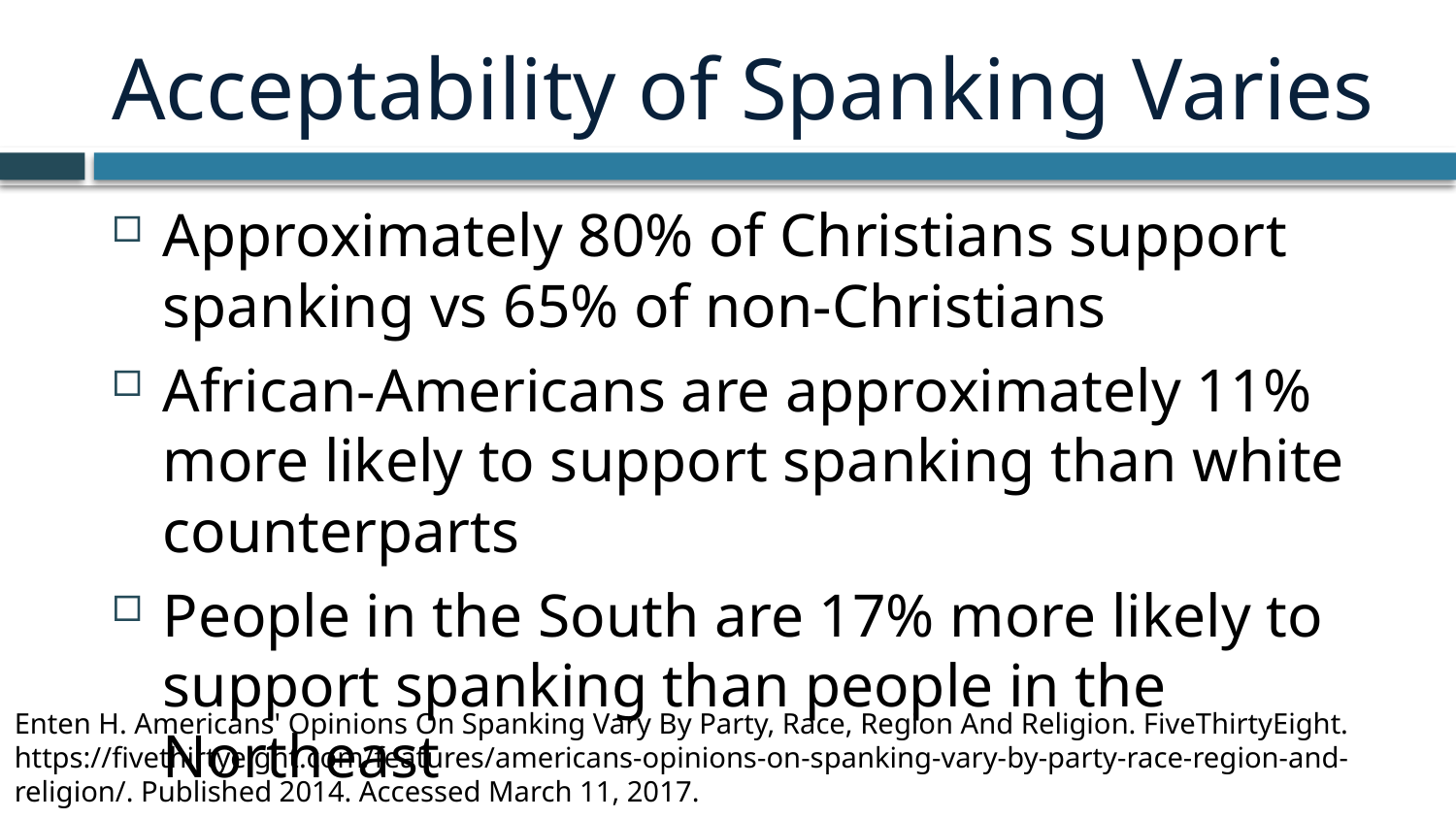

# Acceptability of Spanking Varies
Approximately 80% of Christians support spanking vs 65% of non-Christians
African-Americans are approximately 11% more likely to support spanking than white counterparts
People in the South are 17% more likely to support spanking than people in the Northeast
Enten H. Americans' Opinions On Spanking Vary By Party, Race, Region And Religion. FiveThirtyEight. https://fivethirtyeight.com/features/americans-opinions-on-spanking-vary-by-party-race-region-and-religion/. Published 2014. Accessed March 11, 2017.

## Slide 26
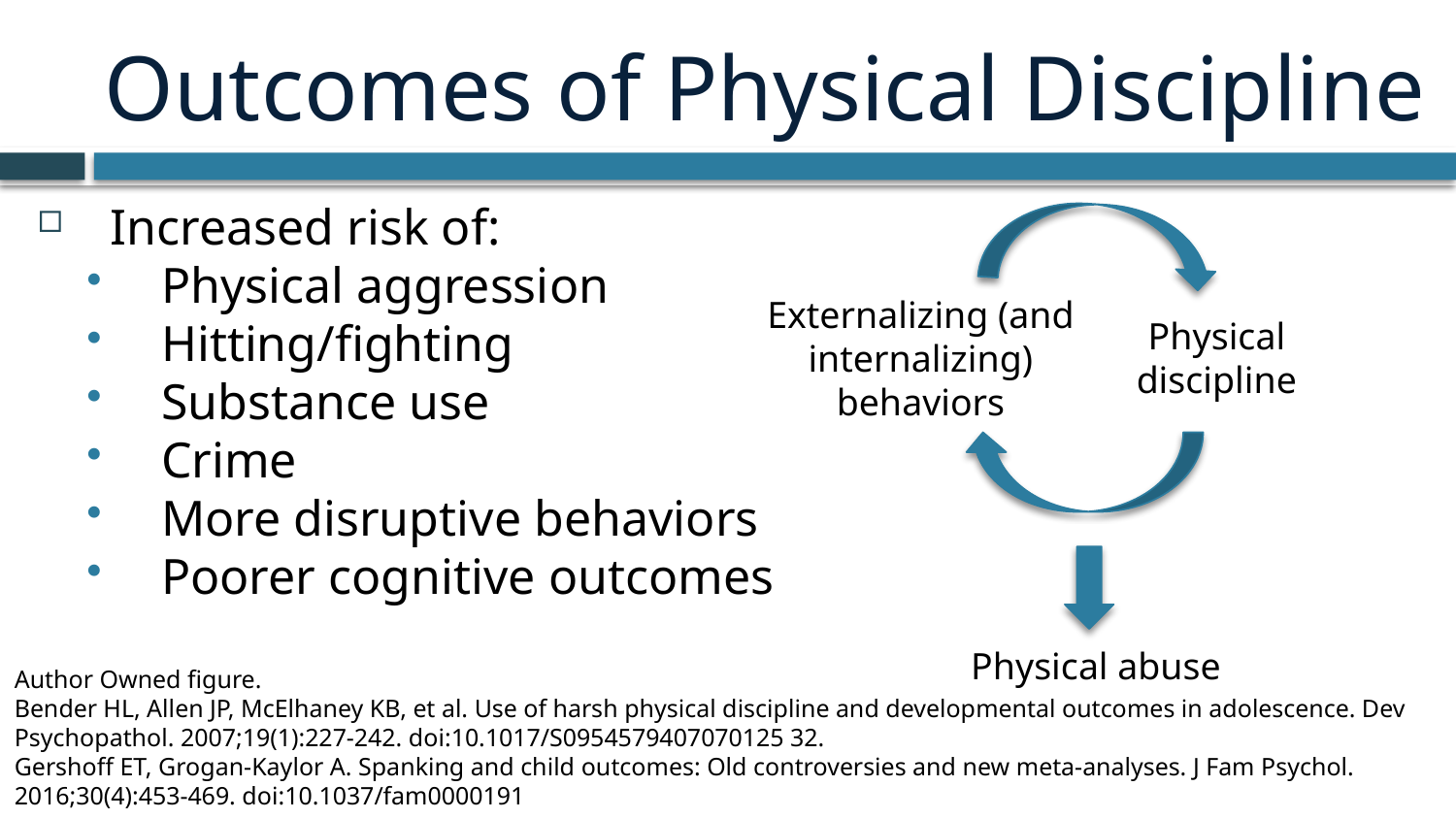

# Outcomes of Physical Discipline
Increased risk of:
Physical aggression
Hitting/fighting
Substance use
Crime
More disruptive behaviors
Poorer cognitive outcomes
Externalizing (and internalizing) behaviors
Physical discipline
Physical abuse
Author Owned figure.
Bender HL, Allen JP, McElhaney KB, et al. Use of harsh physical discipline and developmental outcomes in adolescence. Dev Psychopathol. 2007;19(1):227-242. doi:10.1017/S0954579407070125 32.
Gershoff ET, Grogan-Kaylor A. Spanking and child outcomes: Old controversies and new meta-analyses. J Fam Psychol. 2016;30(4):453-469. doi:10.1037/fam0000191

## Slide 27
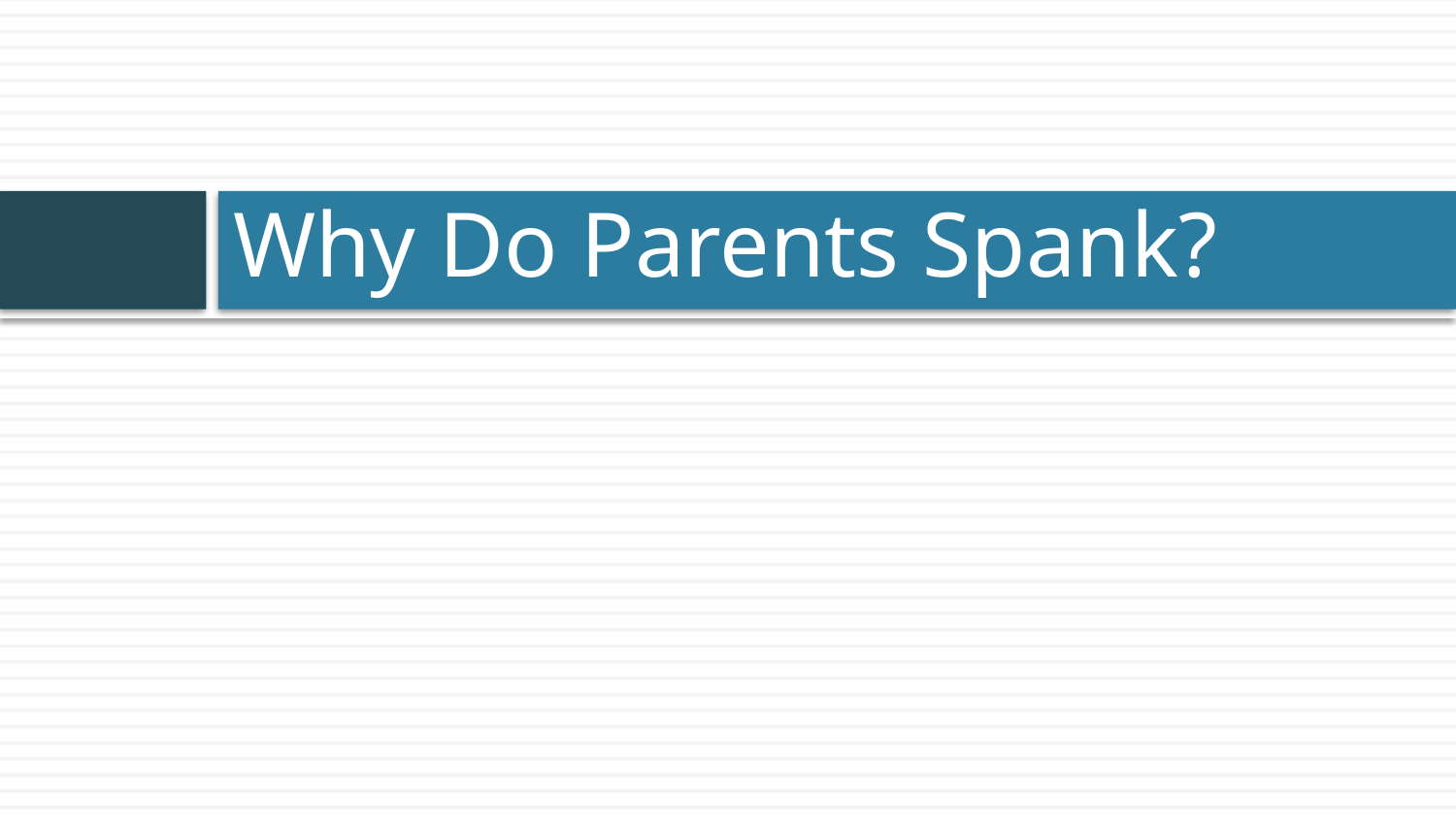

# Why Do Parents Spank?

## Slide 28
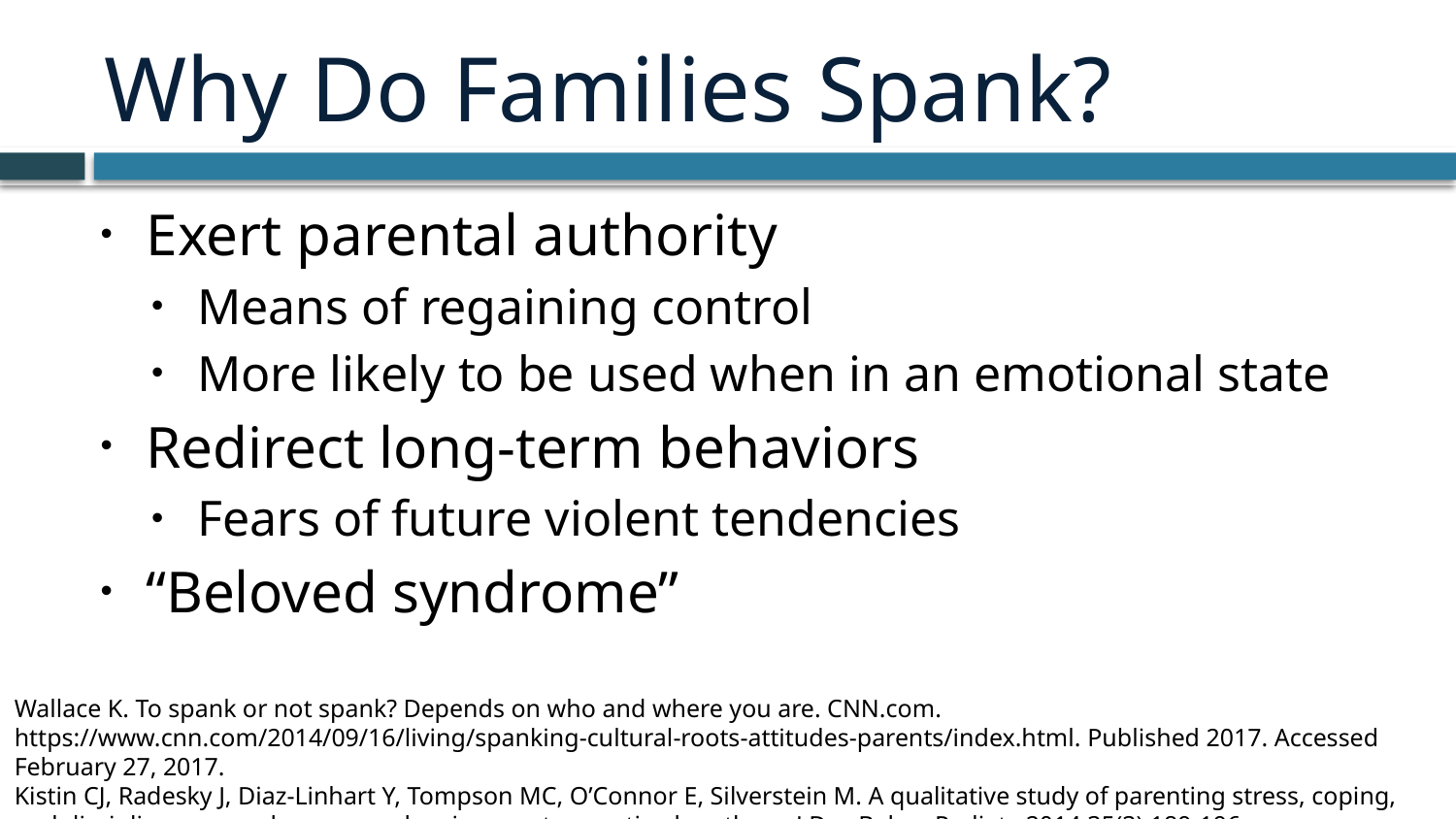

# Why Do Families Spank?
Exert parental authority
Means of regaining control
More likely to be used when in an emotional state
Redirect long-term behaviors
Fears of future violent tendencies
“Beloved syndrome”
Wallace K. To spank or not spank? Depends on who and where you are. CNN.com. https://www.cnn.com/2014/09/16/living/spanking-cultural-roots-attitudes-parents/index.html. Published 2017. Accessed February 27, 2017.
Kistin CJ, Radesky J, Diaz-Linhart Y, Tompson MC, OʼConnor E, Silverstein M. A qualitative study of parenting stress, coping, and discipline approaches among low-income traumatized mothers. J Dev Behav Pediatr. 2014;35(3):189-196. doi:10.1097/DBP.0000000000000032

## Slide 29
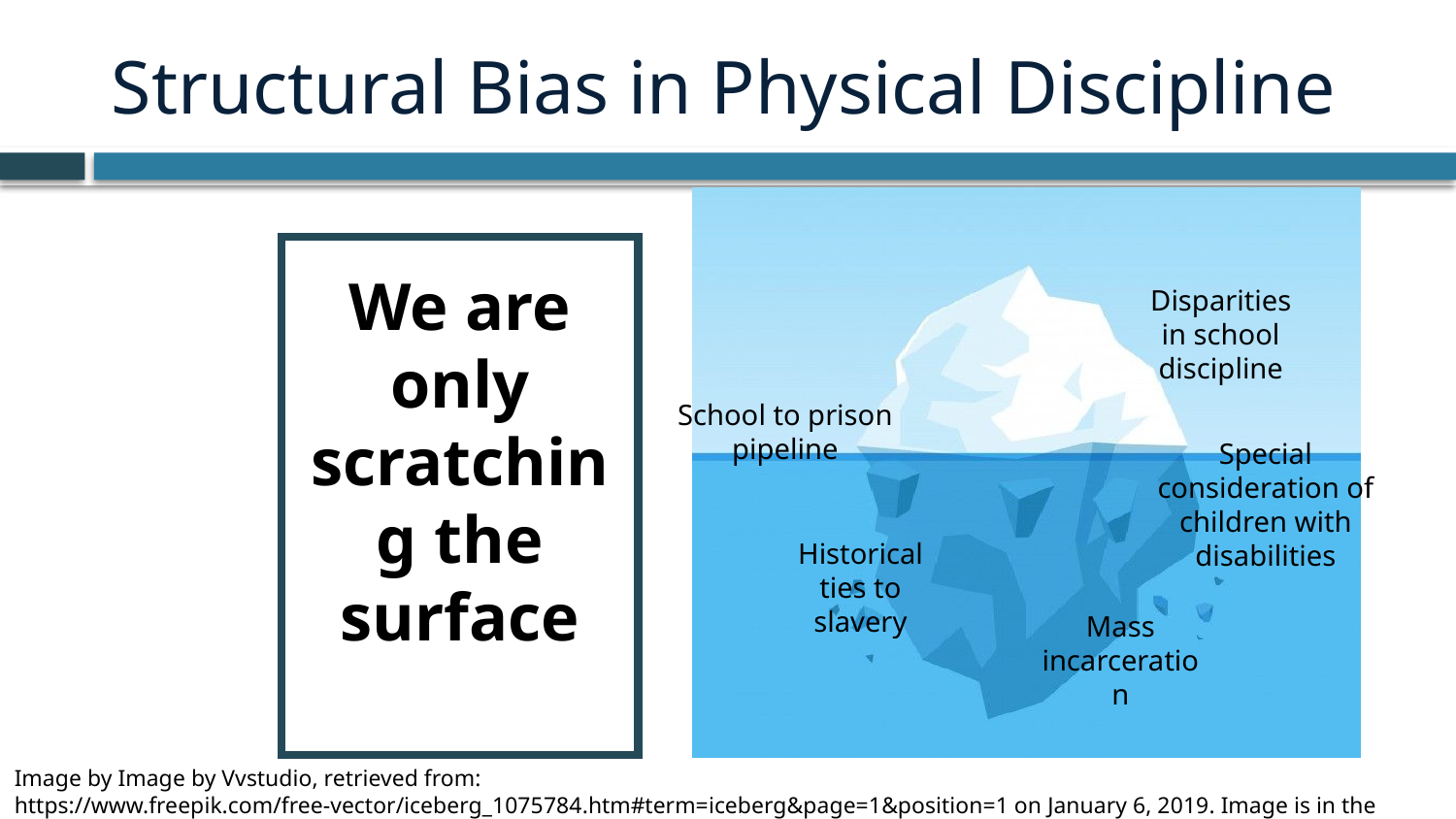

# Structural Bias in Physical Discipline
We are only scratching the surface
Disparities in school discipline
School to prison pipeline
Special consideration of children with disabilities
Historical ties to slavery
Mass incarceration
Image by Image by Vvstudio, retrieved from: https://www.freepik.com/free-vector/iceberg_1075784.htm#term=iceberg&page=1&position=1 on January 6, 2019. Image is in the public domain.

## Slide 30
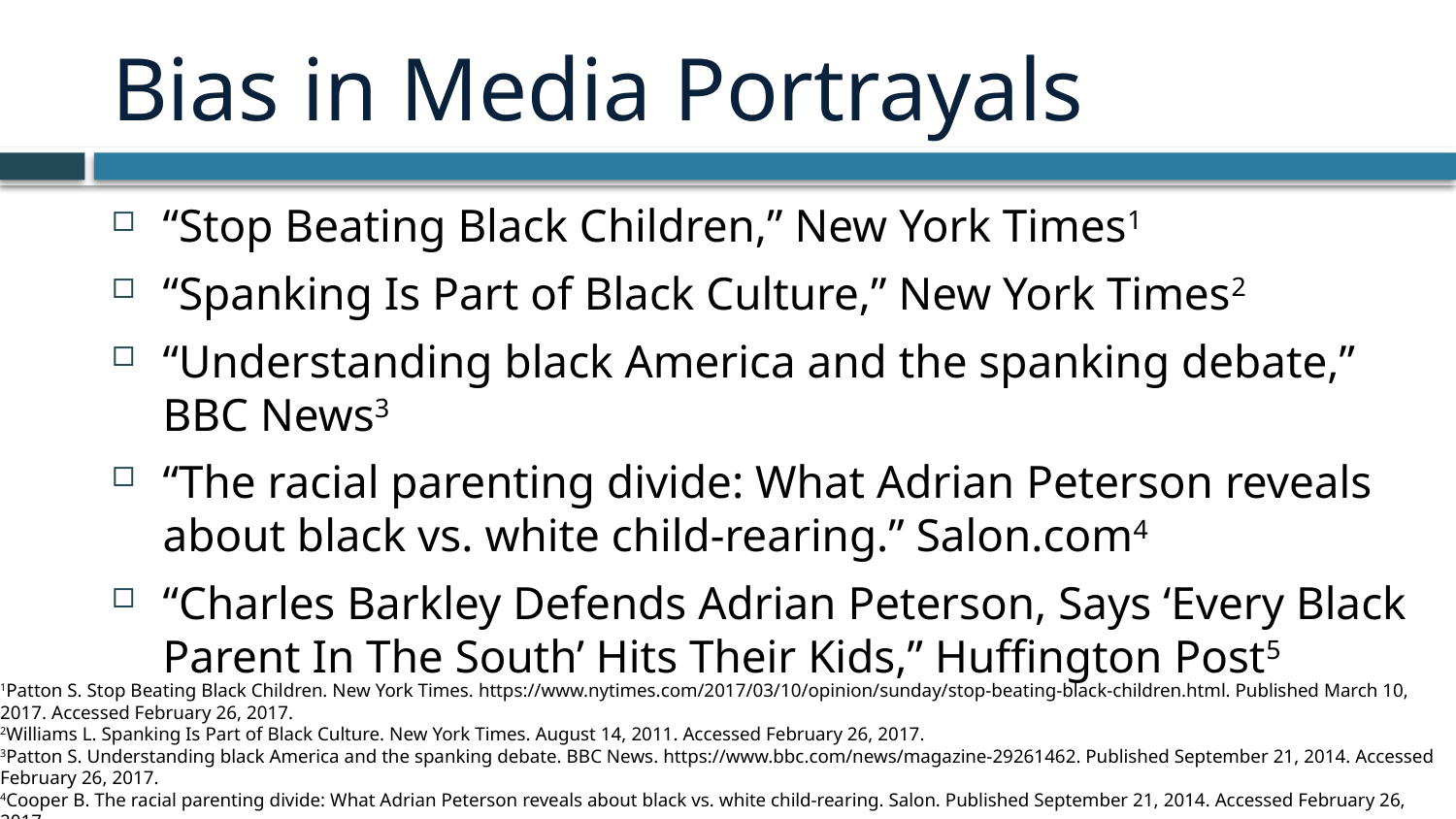

# Bias in Media Portrayals
“Stop Beating Black Children,” New York Times1
“Spanking Is Part of Black Culture,” New York Times2
“Understanding black America and the spanking debate,” BBC News3
“The racial parenting divide: What Adrian Peterson reveals about black vs. white child-rearing.” Salon.com4
“Charles Barkley Defends Adrian Peterson, Says ‘Every Black Parent In The South’ Hits Their Kids,” Huffington Post5
1Patton S. Stop Beating Black Children. New York Times. https://www.nytimes.com/2017/03/10/opinion/sunday/stop-beating-black-children.html. Published March 10, 2017. Accessed February 26, 2017.
2Williams L. Spanking Is Part of Black Culture. New York Times. August 14, 2011. Accessed February 26, 2017.
3Patton S. Understanding black America and the spanking debate. BBC News. https://www.bbc.com/news/magazine-29261462. Published September 21, 2014. Accessed February 26, 2017.
4Cooper B. The racial parenting divide: What Adrian Peterson reveals about black vs. white child-rearing. Salon. Published September 21, 2014. Accessed February 26, 2017.
5Sieczkowski C. Charles Barkley Defends Adrian Peterson, Says 'Every Black Parent In The South' Hits Their Kids. Huffington Post. September 15, 2014. Accessed February 26, 2017.

## Slide 31
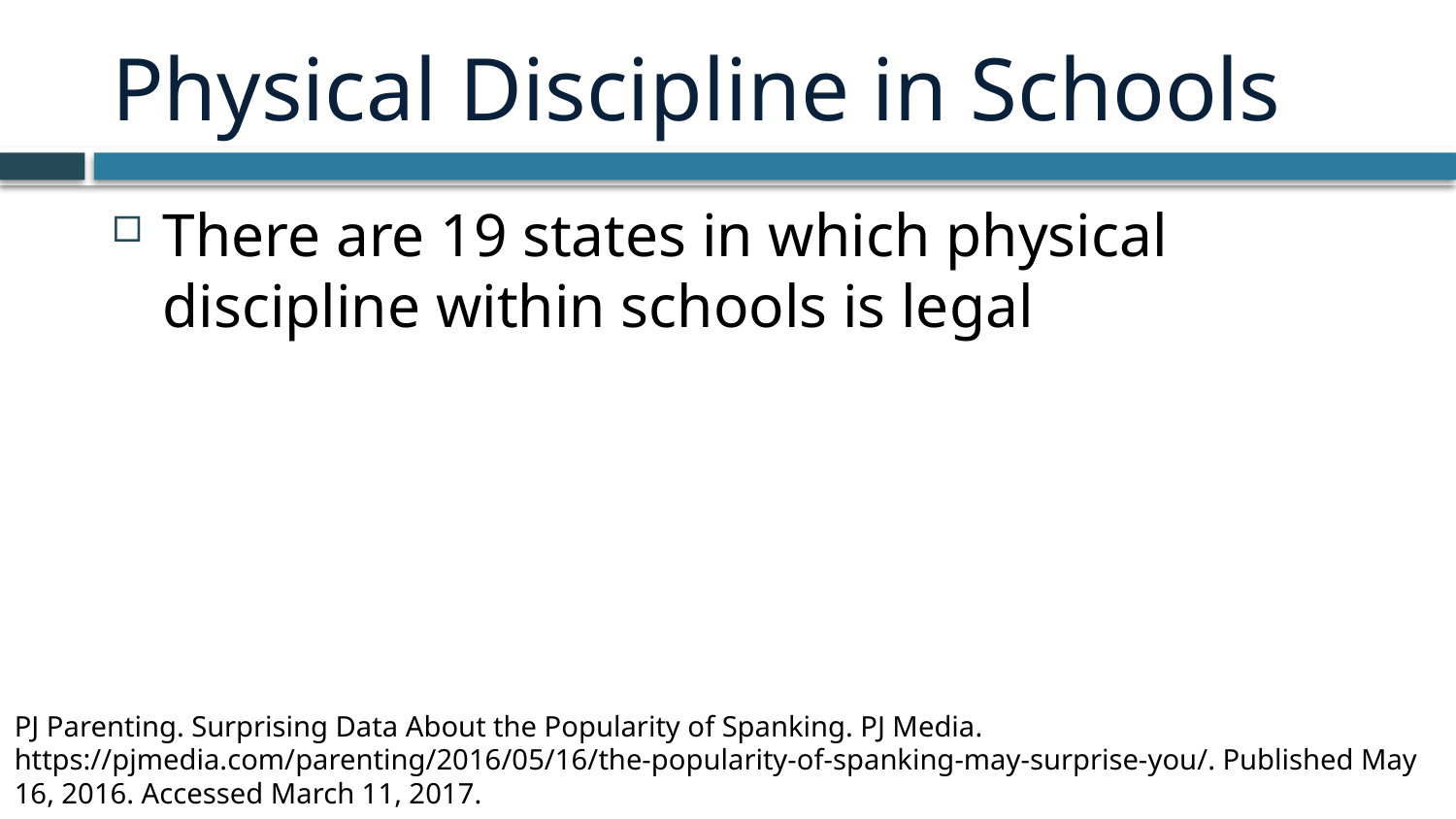

# Physical Discipline in Schools
There are 19 states in which physical discipline within schools is legal
PJ Parenting. Surprising Data About the Popularity of Spanking. PJ Media. https://pjmedia.com/parenting/2016/05/16/the-popularity-of-spanking-may-surprise-you/. Published May 16, 2016. Accessed March 11, 2017.

## Slide 32
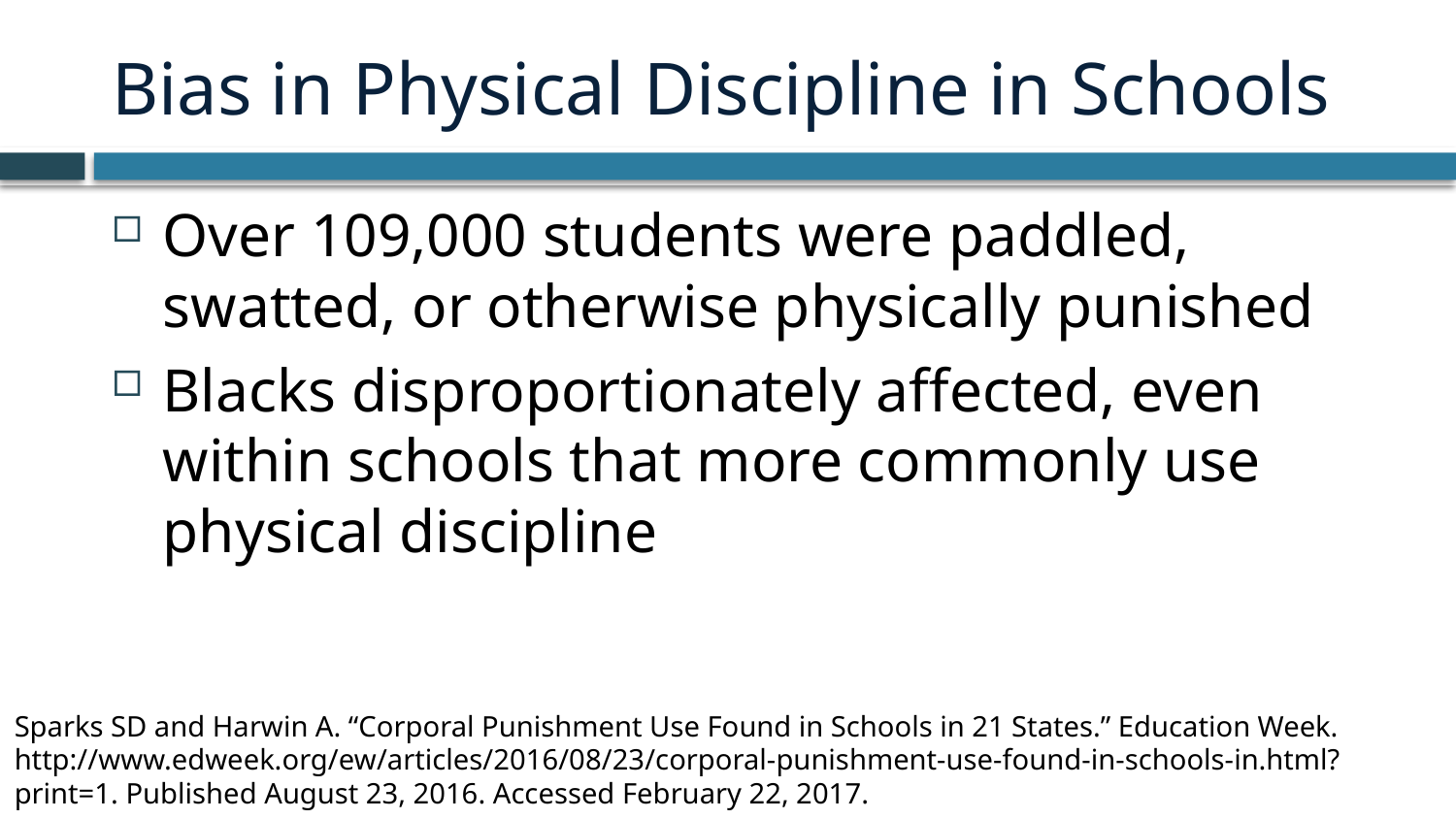

# Bias in Physical Discipline in Schools
Over 109,000 students were paddled, swatted, or otherwise physically punished
Blacks disproportionately affected, even within schools that more commonly use physical discipline
Sparks SD and Harwin A. “Corporal Punishment Use Found in Schools in 21 States.” Education Week. http://www.edweek.org/ew/articles/2016/08/23/corporal-punishment-use-found-in-schools-in.html?print=1. Published August 23, 2016. Accessed February 22, 2017.

## Slide 33
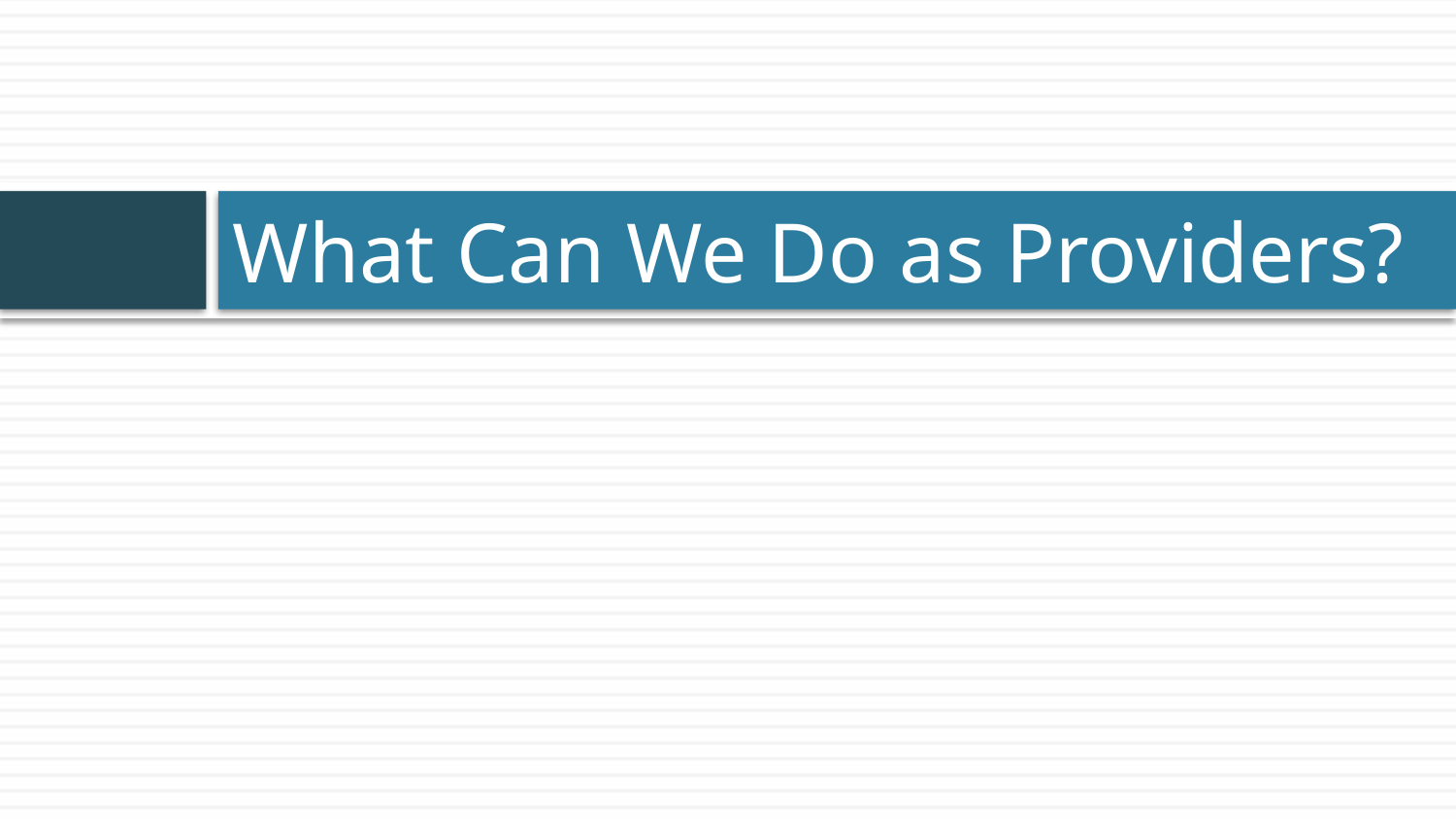

# What Can We Do as Providers?

## Slide 34
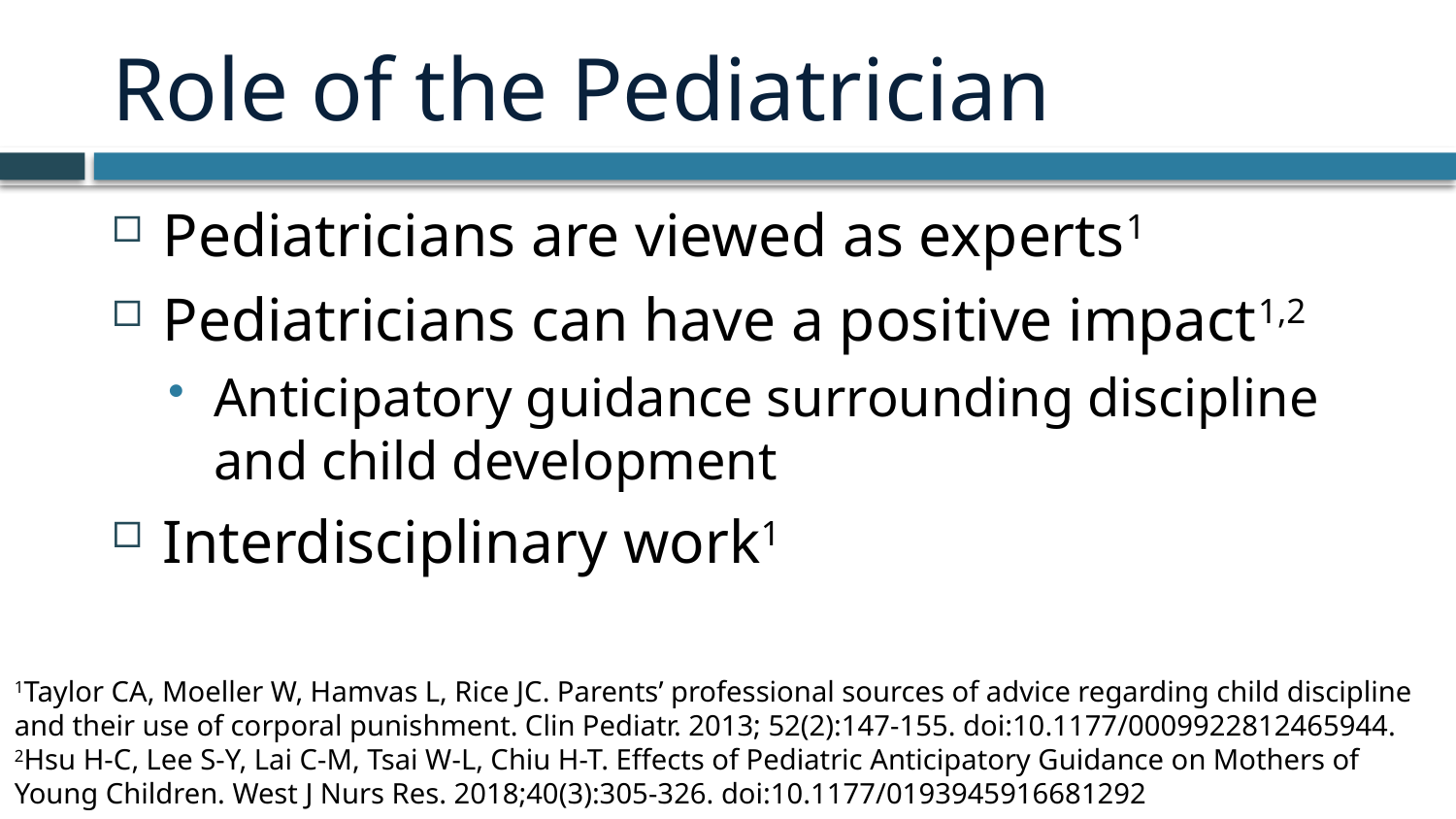

# Role of the Pediatrician
Pediatricians are viewed as experts1
Pediatricians can have a positive impact1,2
Anticipatory guidance surrounding discipline and child development
Interdisciplinary work1
1Taylor CA, Moeller W, Hamvas L, Rice JC. Parents’ professional sources of advice regarding child discipline and their use of corporal punishment. Clin Pediatr. 2013; 52(2):147-155. doi:10.1177/0009922812465944.
2Hsu H-C, Lee S-Y, Lai C-M, Tsai W-L, Chiu H-T. Effects of Pediatric Anticipatory Guidance on Mothers of Young Children. West J Nurs Res. 2018;40(3):305-326. doi:10.1177/0193945916681292

## Slide 35
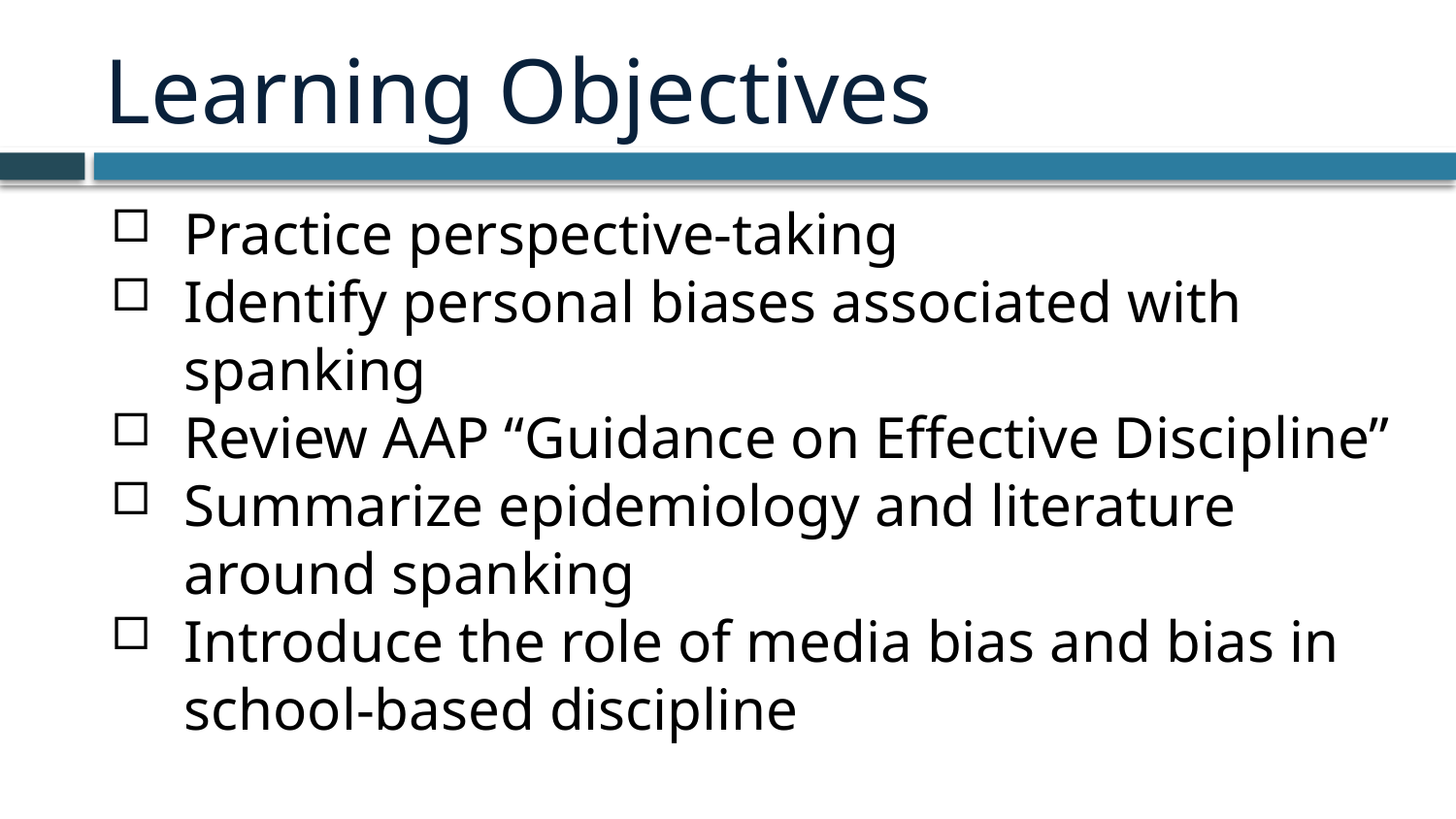

# Learning Objectives
Practice perspective-taking
Identify personal biases associated with spanking
Review AAP “Guidance on Effective Discipline”
Summarize epidemiology and literature around spanking
Introduce the role of media bias and bias in school-based discipline

## Slide 36
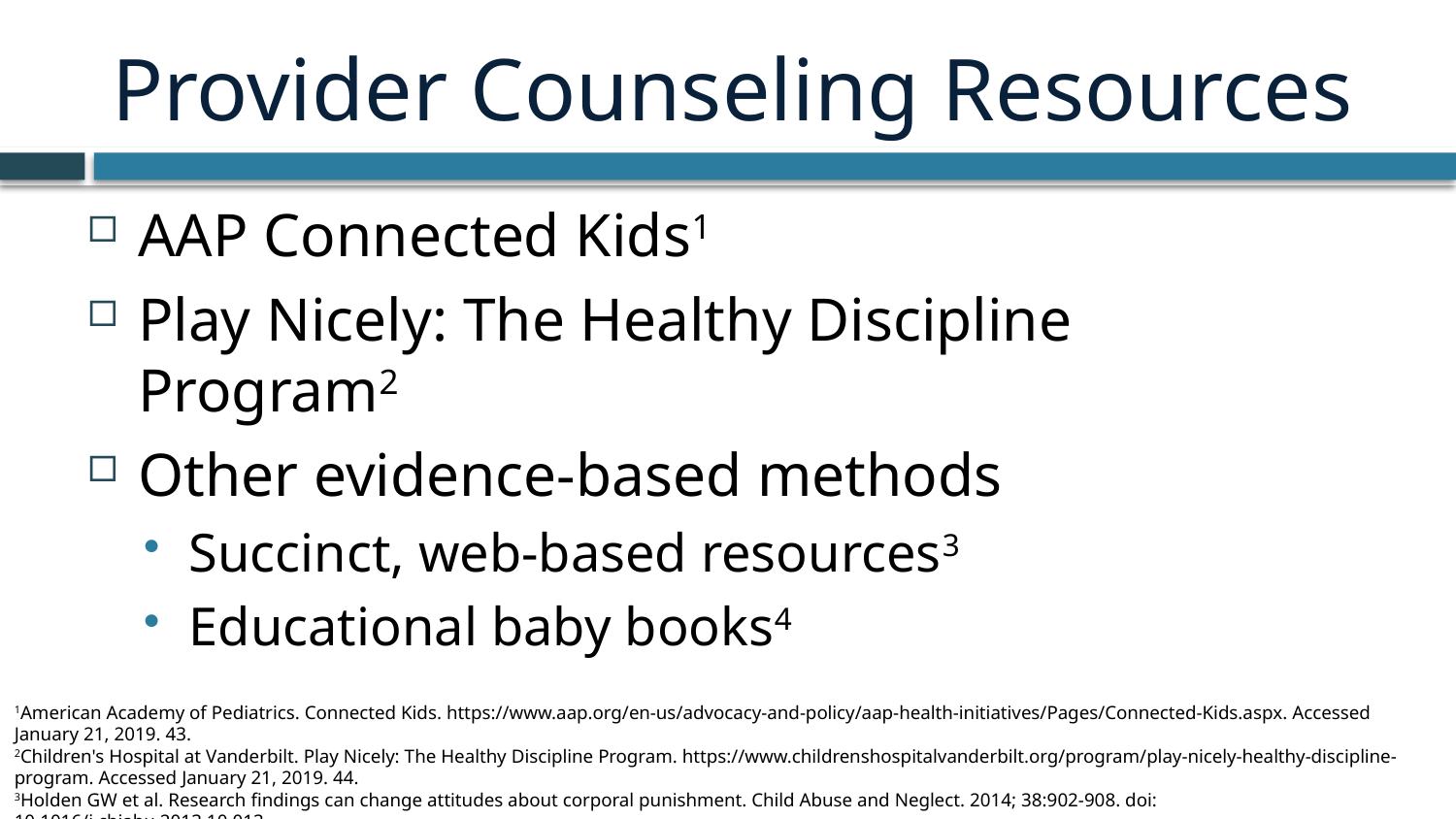

# Provider Counseling Resources
AAP Connected Kids1
Play Nicely: The Healthy Discipline Program2
Other evidence-based methods
Succinct, web-based resources3
Educational baby books4
1American Academy of Pediatrics. Connected Kids. https://www.aap.org/en-us/advocacy-and-policy/aap-health-initiatives/Pages/Connected-Kids.aspx. Accessed January 21, 2019. 43.
2Children's Hospital at Vanderbilt. Play Nicely: The Healthy Discipline Program. https://www.childrenshospitalvanderbilt.org/program/play-nicely-healthy-discipline-program. Accessed January 21, 2019. 44.
3Holden GW et al. Research findings can change attitudes about corporal punishment. Child Abuse and Neglect. 2014; 38:902-908. doi: 10.1016/j.chiabu.2013.10.013
4Reich SM, Penner EK, Duncan GJ, Auger A. Using baby books to change new mothers' attitudes about corporal punishment. Child Abuse Negl. 2012. doi:10.1016/j.chiabu.2011.09.017

## Slide 37
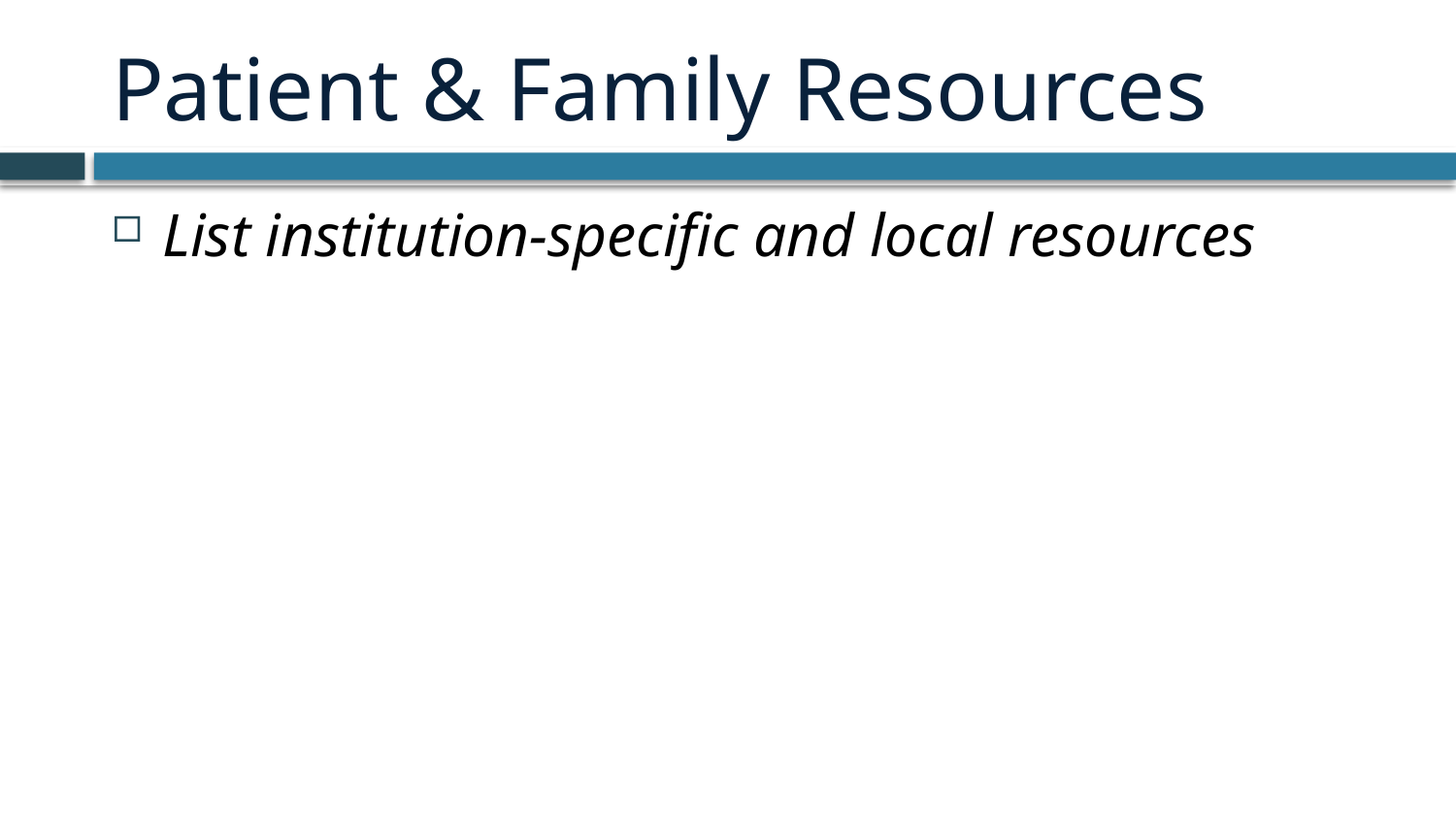

# Patient & Family Resources
List institution-specific and local resources

## Slide 38
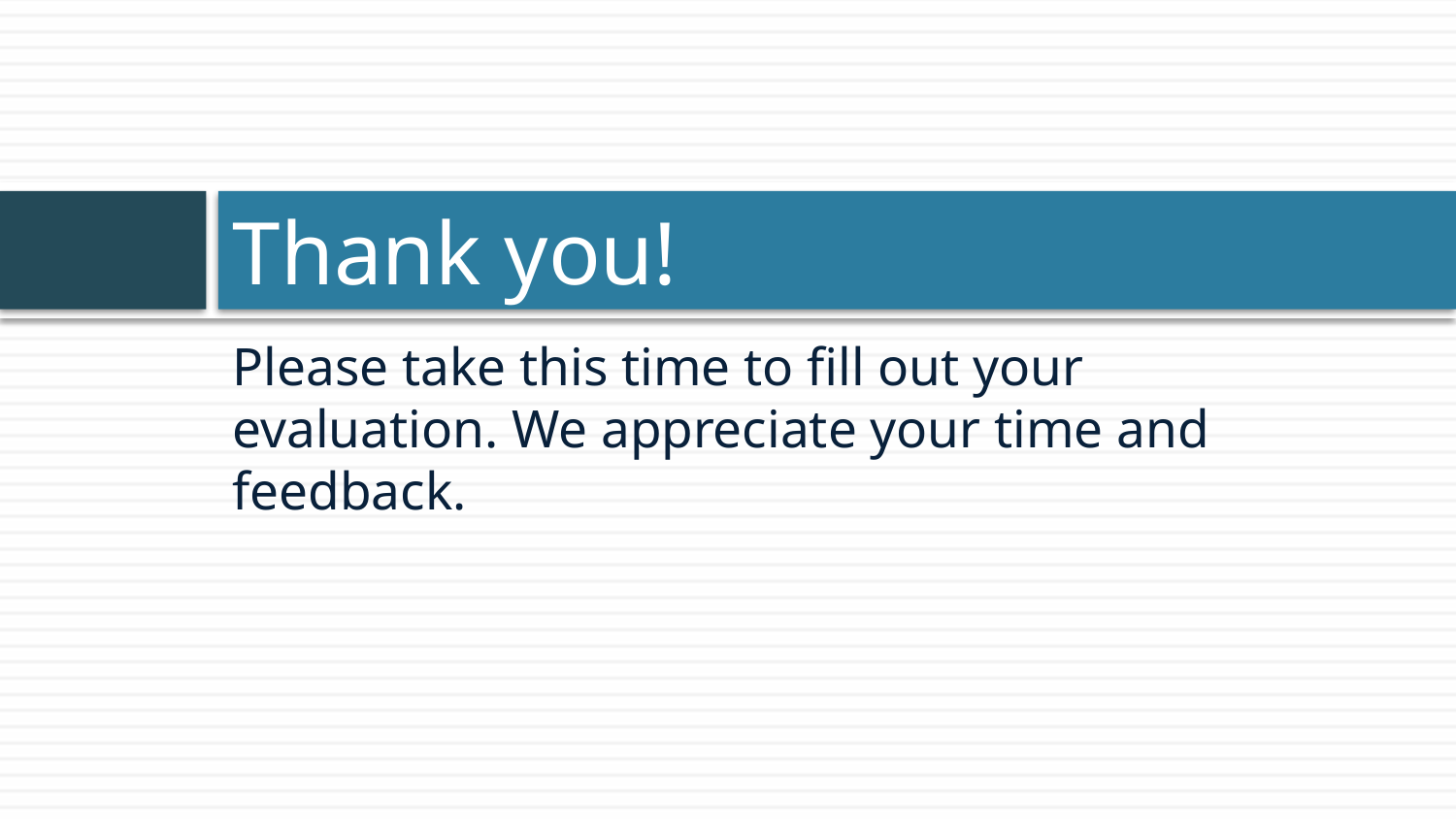

# Thank you!
Please take this time to fill out your evaluation. We appreciate your time and feedback.

## Slide 39
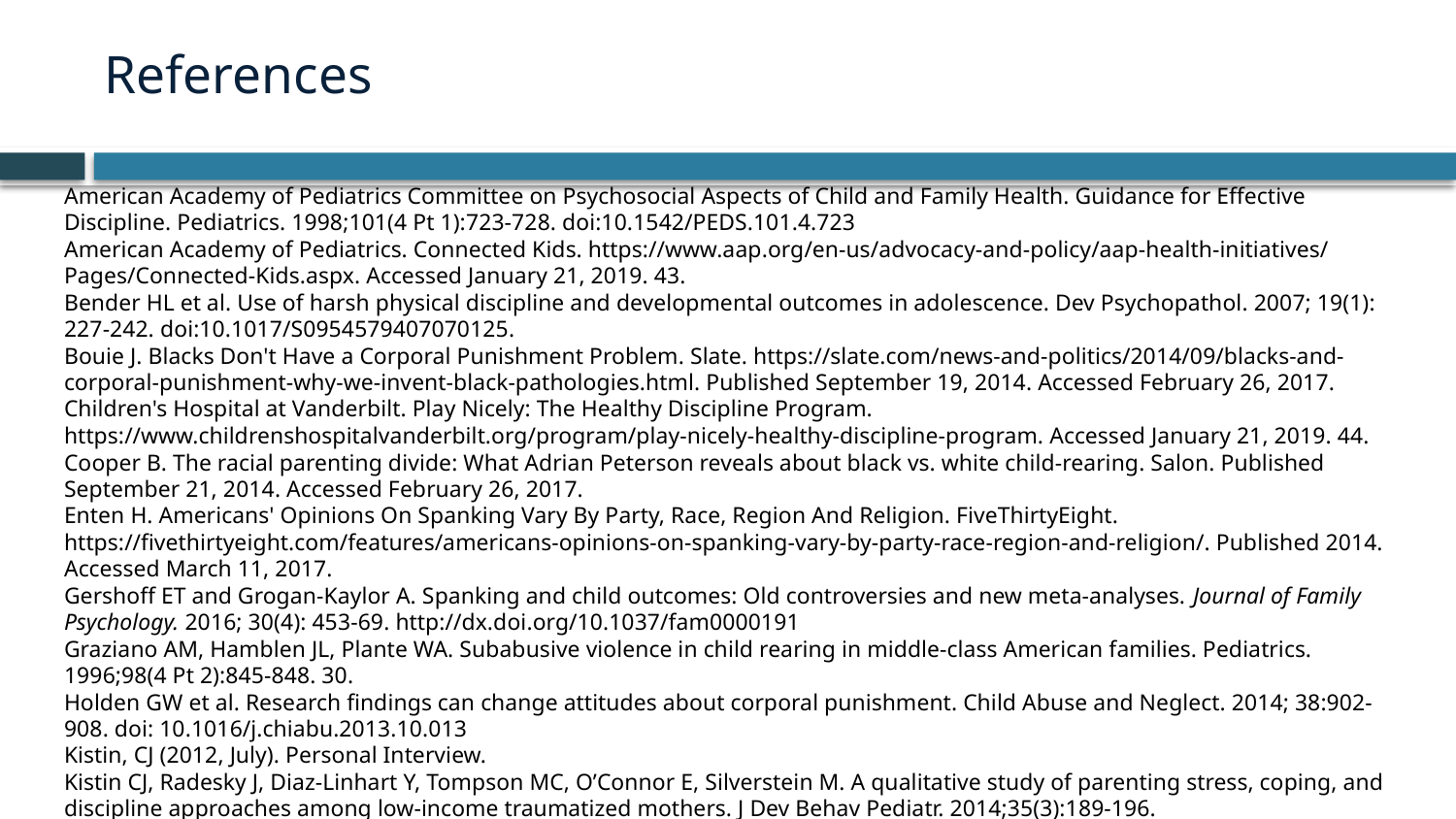

# References
American Academy of Pediatrics Committee on Psychosocial Aspects of Child and Family Health. Guidance for Effective Discipline. Pediatrics. 1998;101(4 Pt 1):723-728. doi:10.1542/PEDS.101.4.723
American Academy of Pediatrics. Connected Kids. https://www.aap.org/en-us/advocacy-and-policy/aap-health-initiatives/Pages/Connected-Kids.aspx. Accessed January 21, 2019. 43.
Bender HL et al. Use of harsh physical discipline and developmental outcomes in adolescence. Dev Psychopathol. 2007; 19(1): 227-242. doi:10.1017/S0954579407070125.
Bouie J. Blacks Don't Have a Corporal Punishment Problem. Slate. https://slate.com/news-and-politics/2014/09/blacks-and-corporal-punishment-why-we-invent-black-pathologies.html. Published September 19, 2014. Accessed February 26, 2017.
Children's Hospital at Vanderbilt. Play Nicely: The Healthy Discipline Program. https://www.childrenshospitalvanderbilt.org/program/play-nicely-healthy-discipline-program. Accessed January 21, 2019. 44.
Cooper B. The racial parenting divide: What Adrian Peterson reveals about black vs. white child-rearing. Salon. Published September 21, 2014. Accessed February 26, 2017.
Enten H. Americans' Opinions On Spanking Vary By Party, Race, Region And Religion. FiveThirtyEight. https://fivethirtyeight.com/features/americans-opinions-on-spanking-vary-by-party-race-region-and-religion/. Published 2014. Accessed March 11, 2017.
Gershoff ET and Grogan-Kaylor A. Spanking and child outcomes: Old controversies and new meta-analyses. Journal of Family Psychology. 2016; 30(4): 453-69. http://dx.doi.org/10.1037/fam0000191
Graziano AM, Hamblen JL, Plante WA. Subabusive violence in child rearing in middle-class American families. Pediatrics. 1996;98(4 Pt 2):845-848. 30.
Holden GW et al. Research findings can change attitudes about corporal punishment. Child Abuse and Neglect. 2014; 38:902-908. doi: 10.1016/j.chiabu.2013.10.013
Kistin, CJ (2012, July). Personal Interview.
Kistin CJ, Radesky J, Diaz-Linhart Y, Tompson MC, OʼConnor E, Silverstein M. A qualitative study of parenting stress, coping, and discipline approaches among low-income traumatized mothers. J Dev Behav Pediatr. 2014;35(3):189-196. doi:10.1097/DBP.0000000000000032

## Slide 40
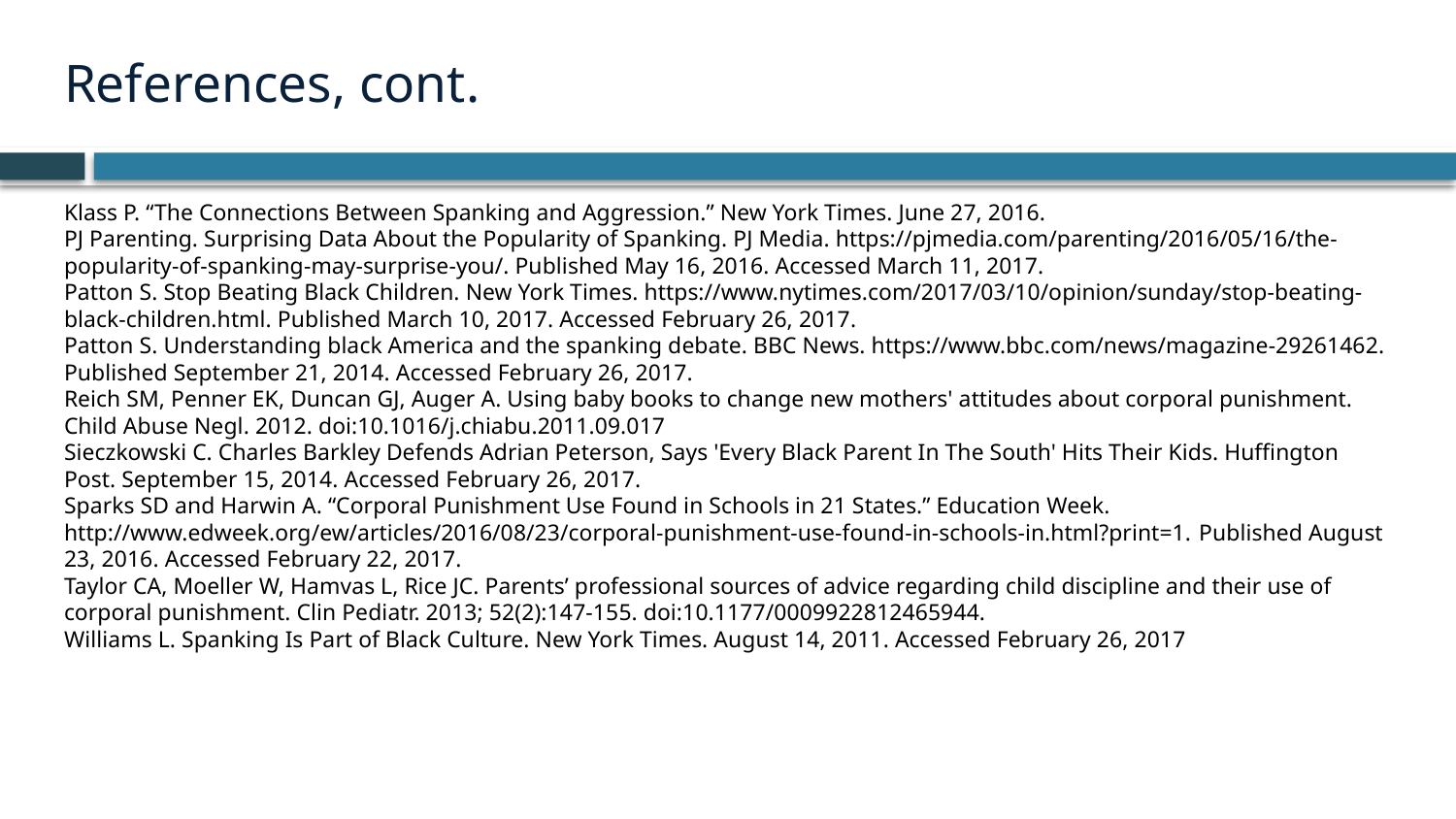

# References, cont.
Klass P. “The Connections Between Spanking and Aggression.” New York Times. June 27, 2016.
PJ Parenting. Surprising Data About the Popularity of Spanking. PJ Media. https://pjmedia.com/parenting/2016/05/16/the-popularity-of-spanking-may-surprise-you/. Published May 16, 2016. Accessed March 11, 2017.
Patton S. Stop Beating Black Children. New York Times. https://www.nytimes.com/2017/03/10/opinion/sunday/stop-beating-black-children.html. Published March 10, 2017. Accessed February 26, 2017.
Patton S. Understanding black America and the spanking debate. BBC News. https://www.bbc.com/news/magazine-29261462. Published September 21, 2014. Accessed February 26, 2017.
Reich SM, Penner EK, Duncan GJ, Auger A. Using baby books to change new mothers' attitudes about corporal punishment. Child Abuse Negl. 2012. doi:10.1016/j.chiabu.2011.09.017
Sieczkowski C. Charles Barkley Defends Adrian Peterson, Says 'Every Black Parent In The South' Hits Their Kids. Huffington Post. September 15, 2014. Accessed February 26, 2017.
Sparks SD and Harwin A. “Corporal Punishment Use Found in Schools in 21 States.” Education Week. http://www.edweek.org/ew/articles/2016/08/23/corporal-punishment-use-found-in-schools-in.html?print=1. Published August 23, 2016. Accessed February 22, 2017.
Taylor CA, Moeller W, Hamvas L, Rice JC. Parents’ professional sources of advice regarding child discipline and their use of corporal punishment. Clin Pediatr. 2013; 52(2):147-155. doi:10.1177/0009922812465944.
Williams L. Spanking Is Part of Black Culture. New York Times. August 14, 2011. Accessed February 26, 2017
