# Supplementary material for: Health Equity Rounds: An Interdisciplinary Case Conference to Address Implicit Bias and Structural Racism for Faculty and Trainees
Source: MedEdPORTAL. 2019 Nov 22;15:10858. doi: 10.15766/mep_2374-8265.10858 (PMC7050660; doi:10.15766/mep_2374-8265.10858)
Supplement: Supplementary file 1 — A. HER 1.pptx B. HER 2.pptx C. HER 3.pptx D. HER 4.pptx E. HER 5.pptx F. HER 6.pptx G. HER 7.pptx H. Selected HER Handouts.docx I. Case Conference Creation Guide.docx J. Glossary.docx K. Evaluation.docx [file mep-15-10858-s001.zip › D. HER 4.pptx]

## Slide 1
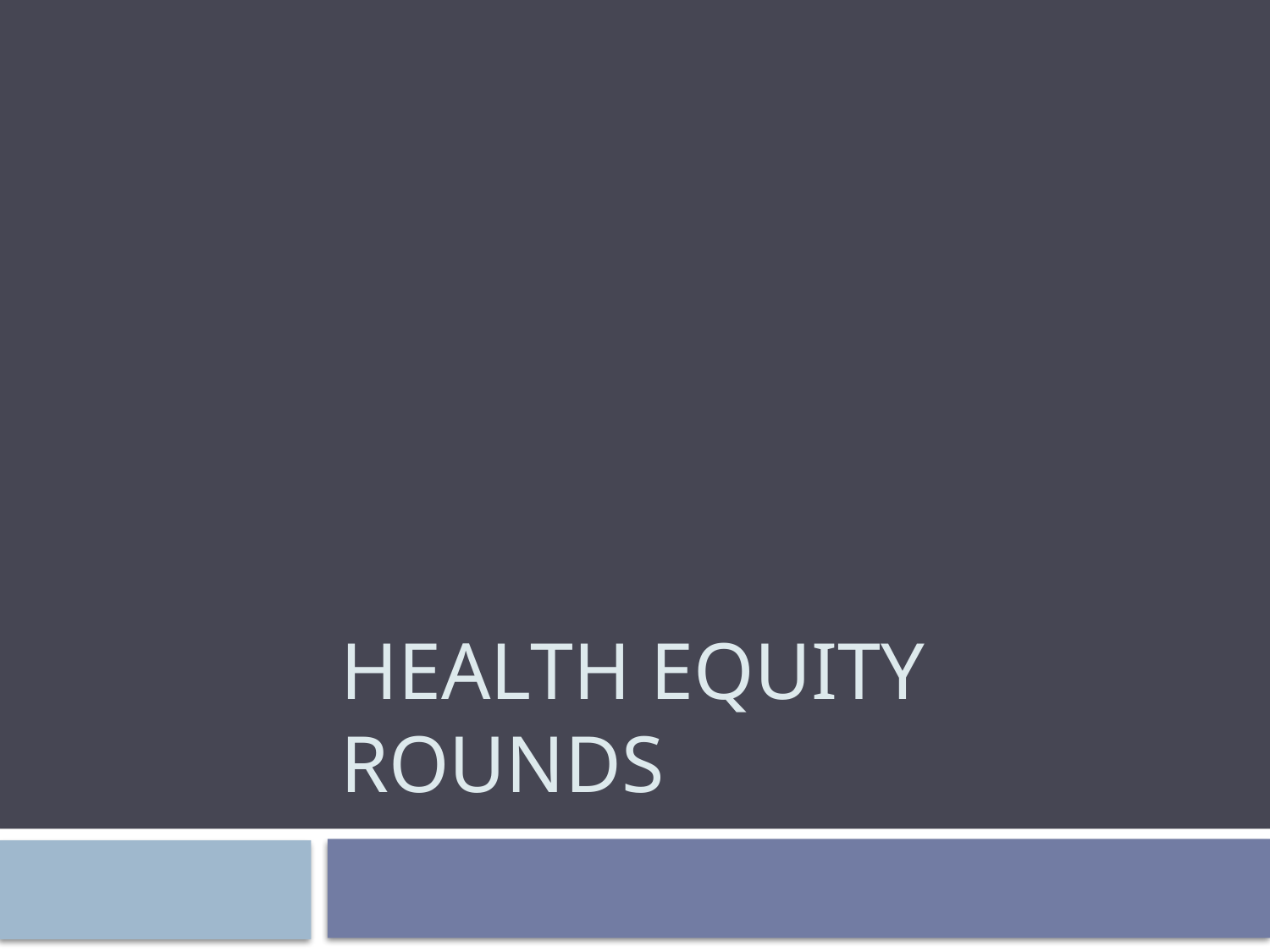

# Health Equity Rounds

## Slide 2
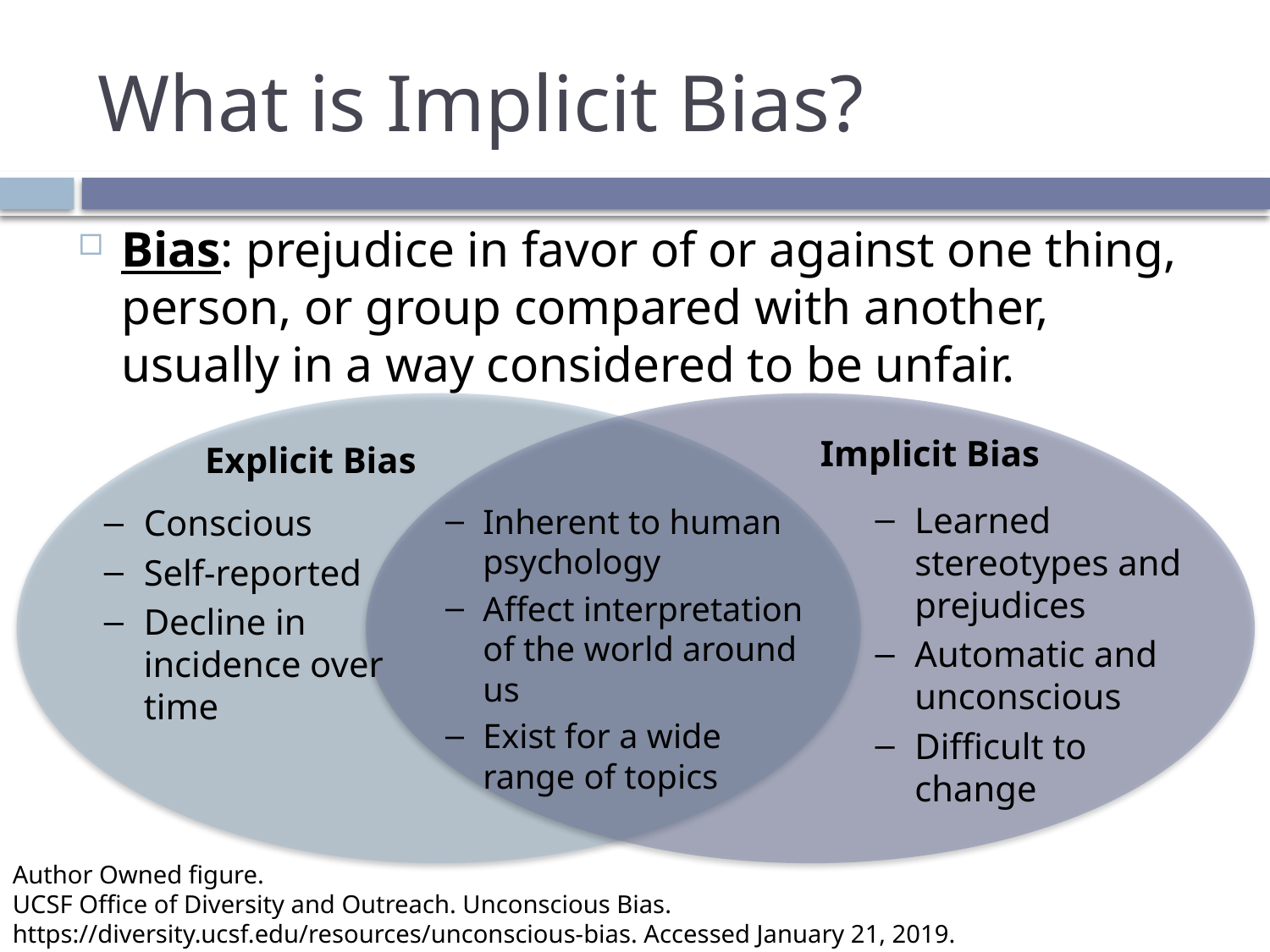

# What is Implicit Bias?
Bias: prejudice in favor of or against one thing, person, or group compared with another, usually in a way considered to be unfair.
Implicit Bias
Explicit Bias
Learned stereotypes and prejudices
Automatic and unconscious
Difficult to change
Inherent to human psychology
Affect interpretation of the world around us
Exist for a wide range of topics
Conscious
Self-reported
Decline in incidence over time
Author Owned figure.
UCSF Office of Diversity and Outreach. Unconscious Bias. https://diversity.ucsf.edu/resources/unconscious-bias. Accessed January 21, 2019.

## Slide 3
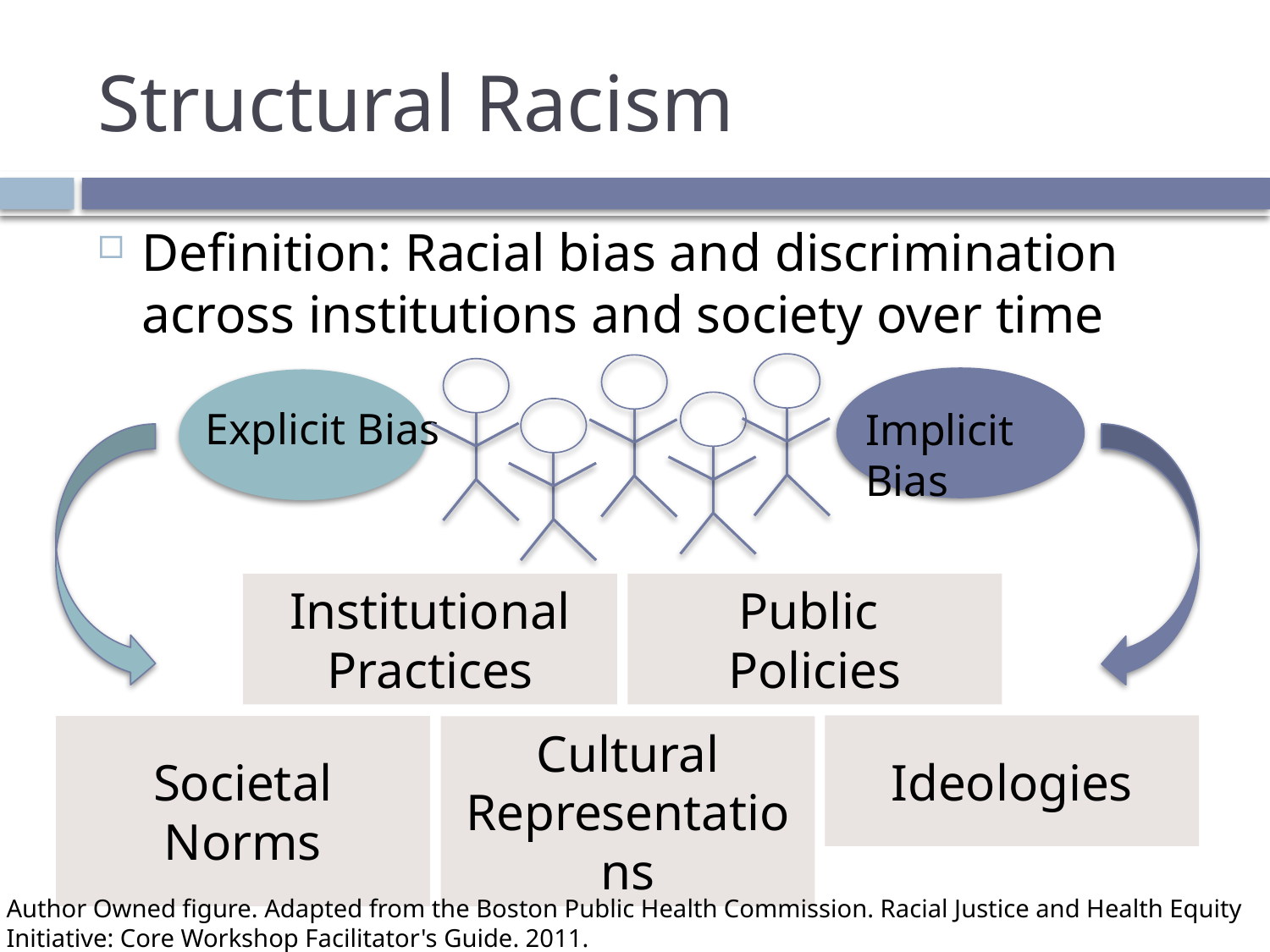

# Structural Racism
Definition: Racial bias and discrimination across institutions and society over time
Explicit Bias
Implicit Bias
Institutional Practices
Public
Policies
Ideologies
Societal Norms
Cultural
Representations
Author Owned figure. Adapted from the Boston Public Health Commission. Racial Justice and Health Equity Initiative: Core Workshop Facilitator's Guide. 2011.

## Slide 4
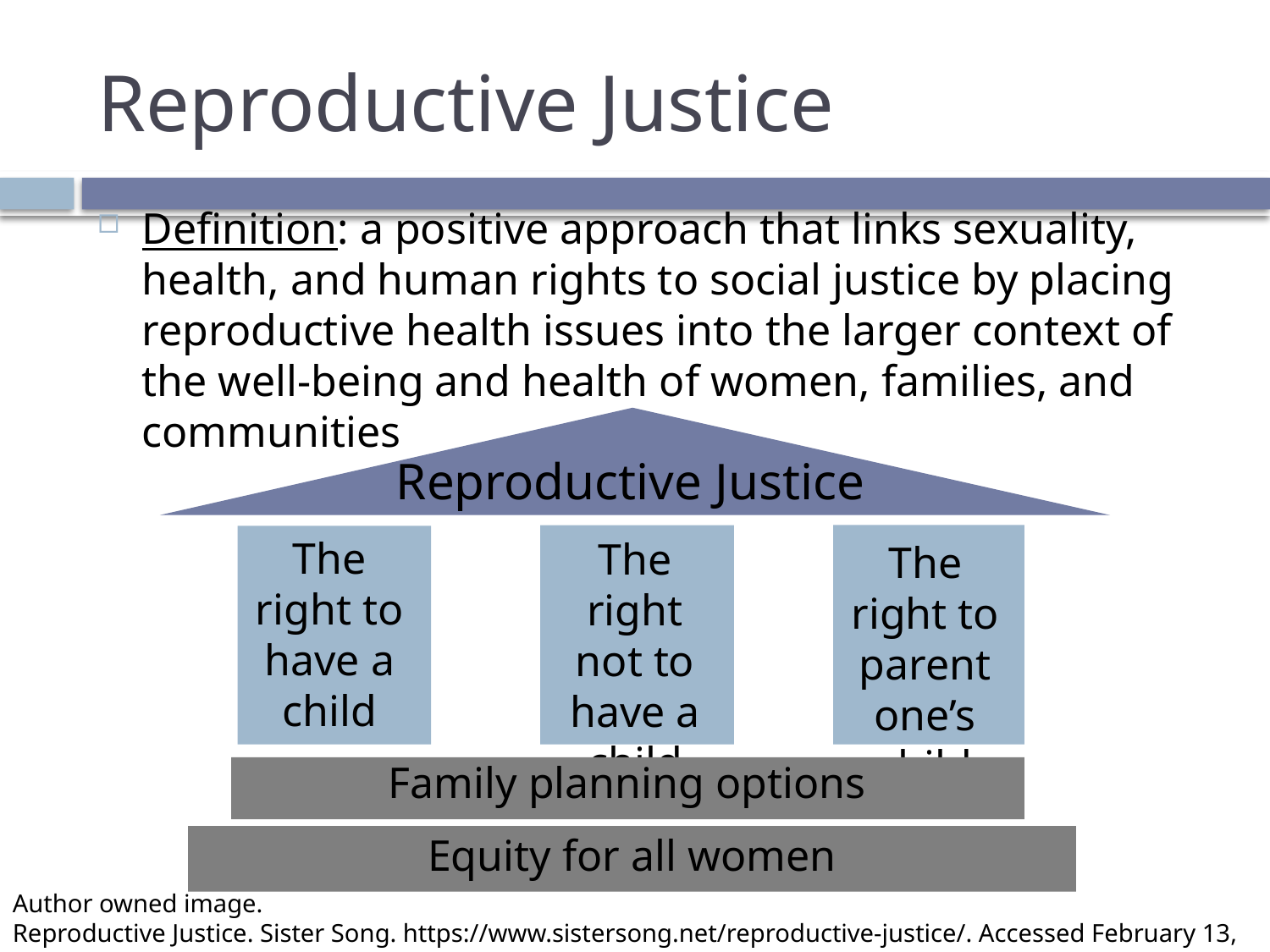

# Reproductive Justice
Definition: a positive approach that links sexuality, health, and human rights to social justice by placing reproductive health issues into the larger context of the well-being and health of women, families, and communities
Reproductive Justice
The right to have a child
The right not to have a child
The right to parent one’s child
Family planning options
Equity for all women
Author owned image.
Reproductive Justice. Sister Song. https://www.sistersong.net/reproductive-justice/. Accessed February 13, 2019.

## Slide 5
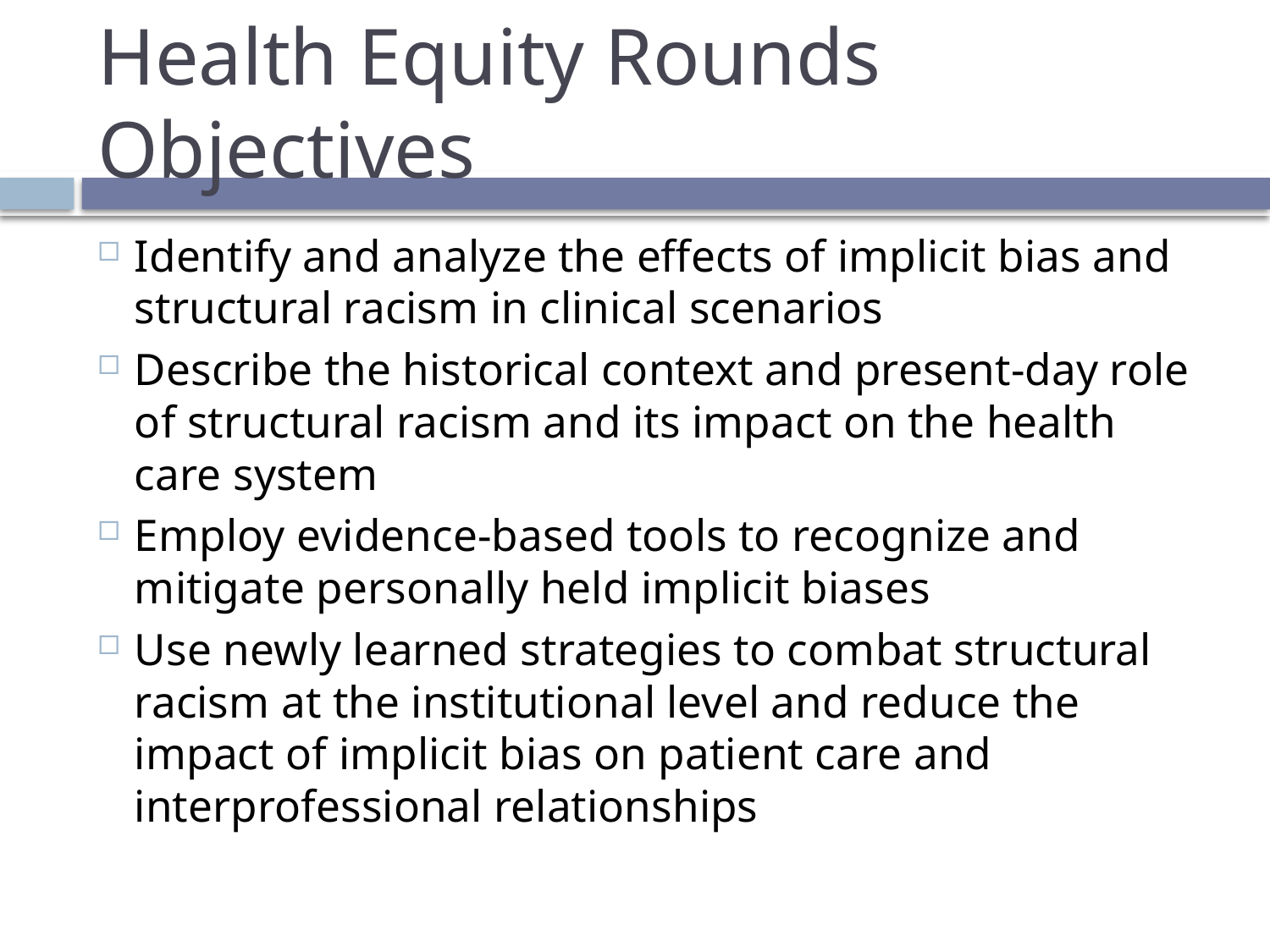

# Health Equity Rounds Objectives
Identify and analyze the effects of implicit bias and structural racism in clinical scenarios
Describe the historical context and present-day role of structural racism and its impact on the health care system
Employ evidence-based tools to recognize and mitigate personally held implicit biases
Use newly learned strategies to combat structural racism at the institutional level and reduce the impact of implicit bias on patient care and interprofessional relationships

## Slide 6
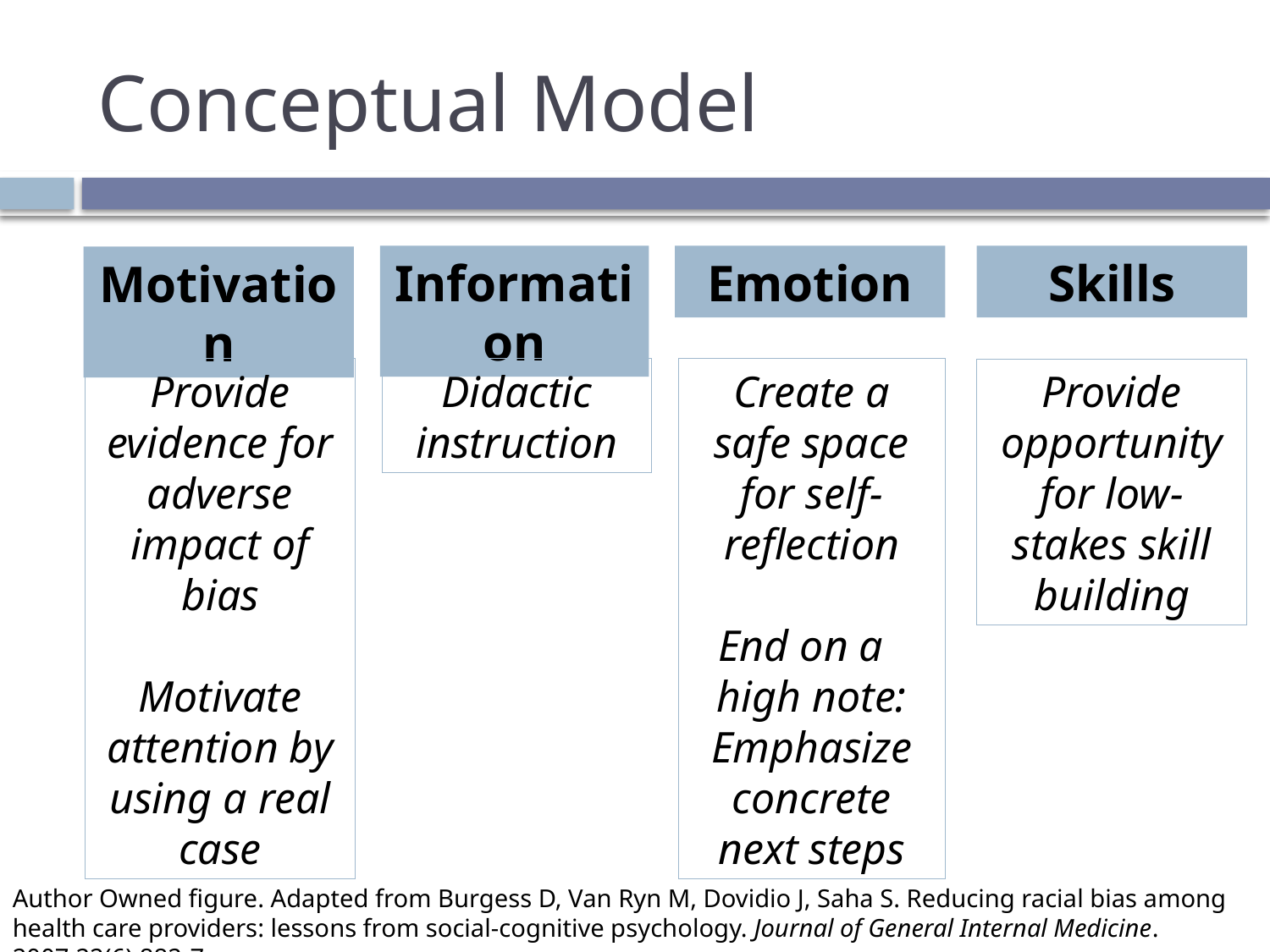

# Conceptual Model
Skills
Information
Emotion
Motivation
Create a safe space for self-reflection
d
End on a high note: Emphasize concrete next steps
Provide evidence for adverse impact of bias
Motivate attention by using a real case
Didactic instruction
Provide opportunity for low-stakes skill building
Author Owned figure. Adapted from Burgess D, Van Ryn M, Dovidio J, Saha S. Reducing racial bias among health care providers: lessons from social-cognitive psychology. Journal of General Internal Medicine. 2007;22(6):882-7.

## Slide 7
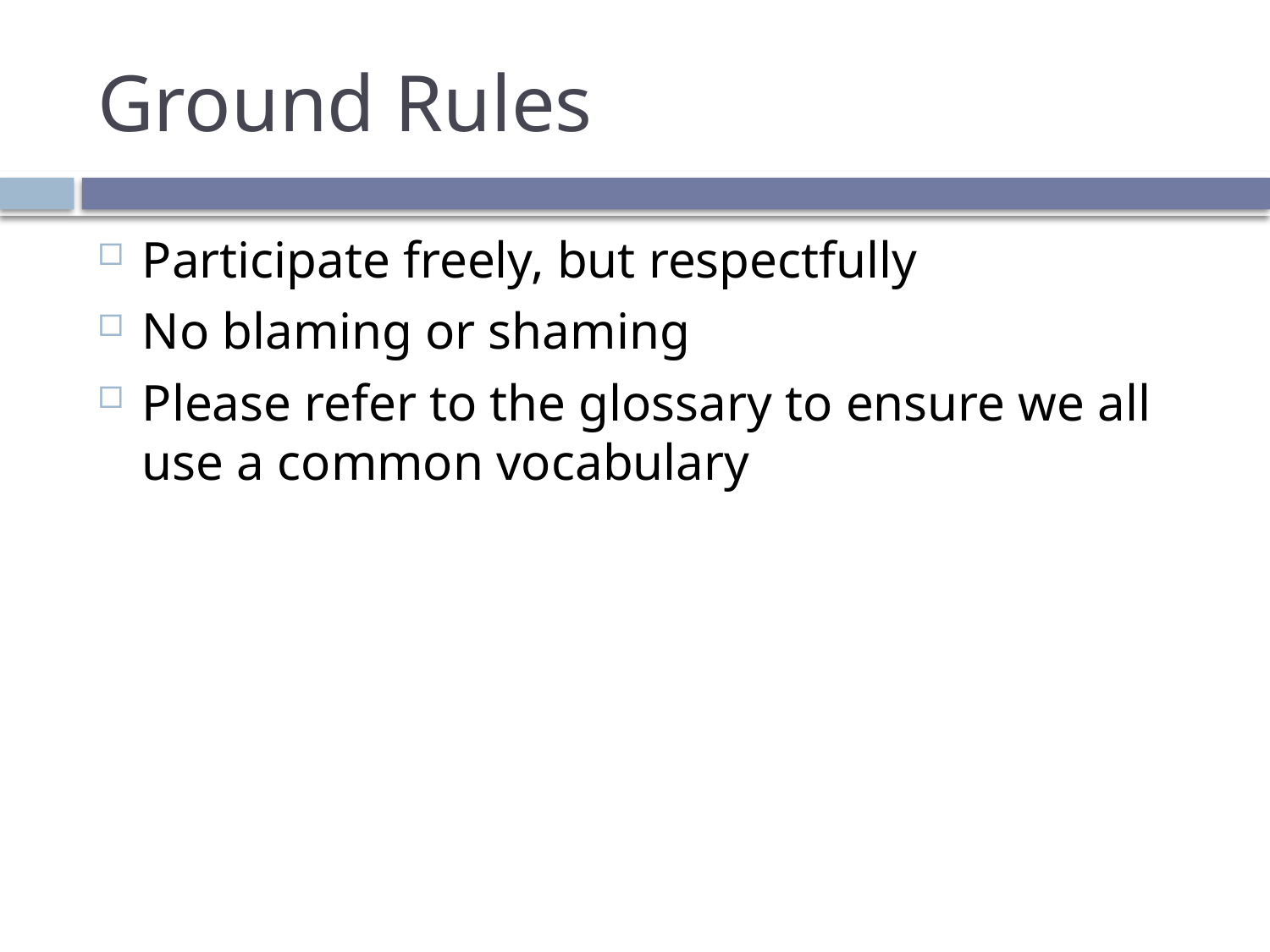

# Ground Rules
Participate freely, but respectfully
No blaming or shaming
Please refer to the glossary to ensure we all use a common vocabulary

## Slide 8
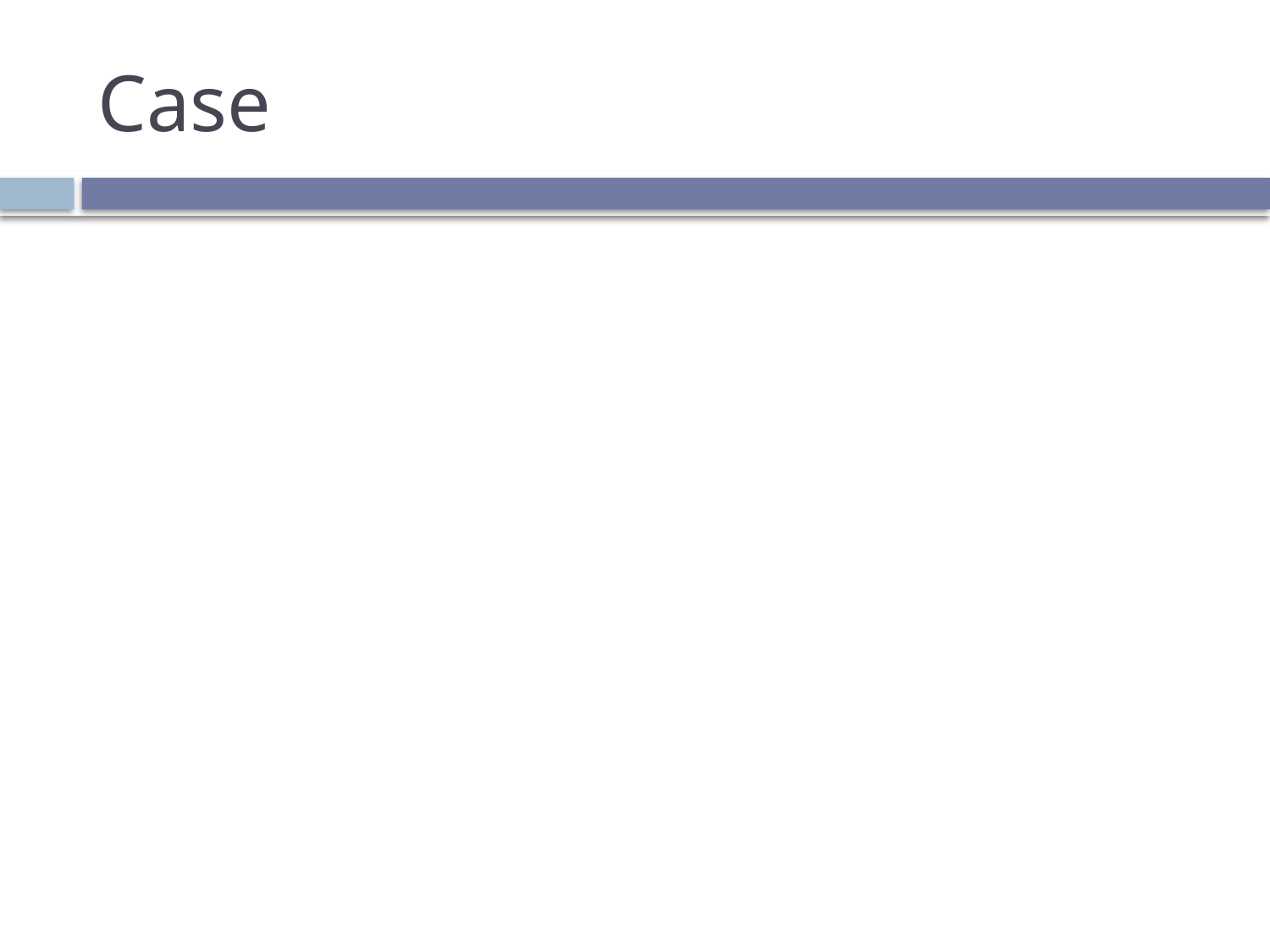

# Case

## Slide 9
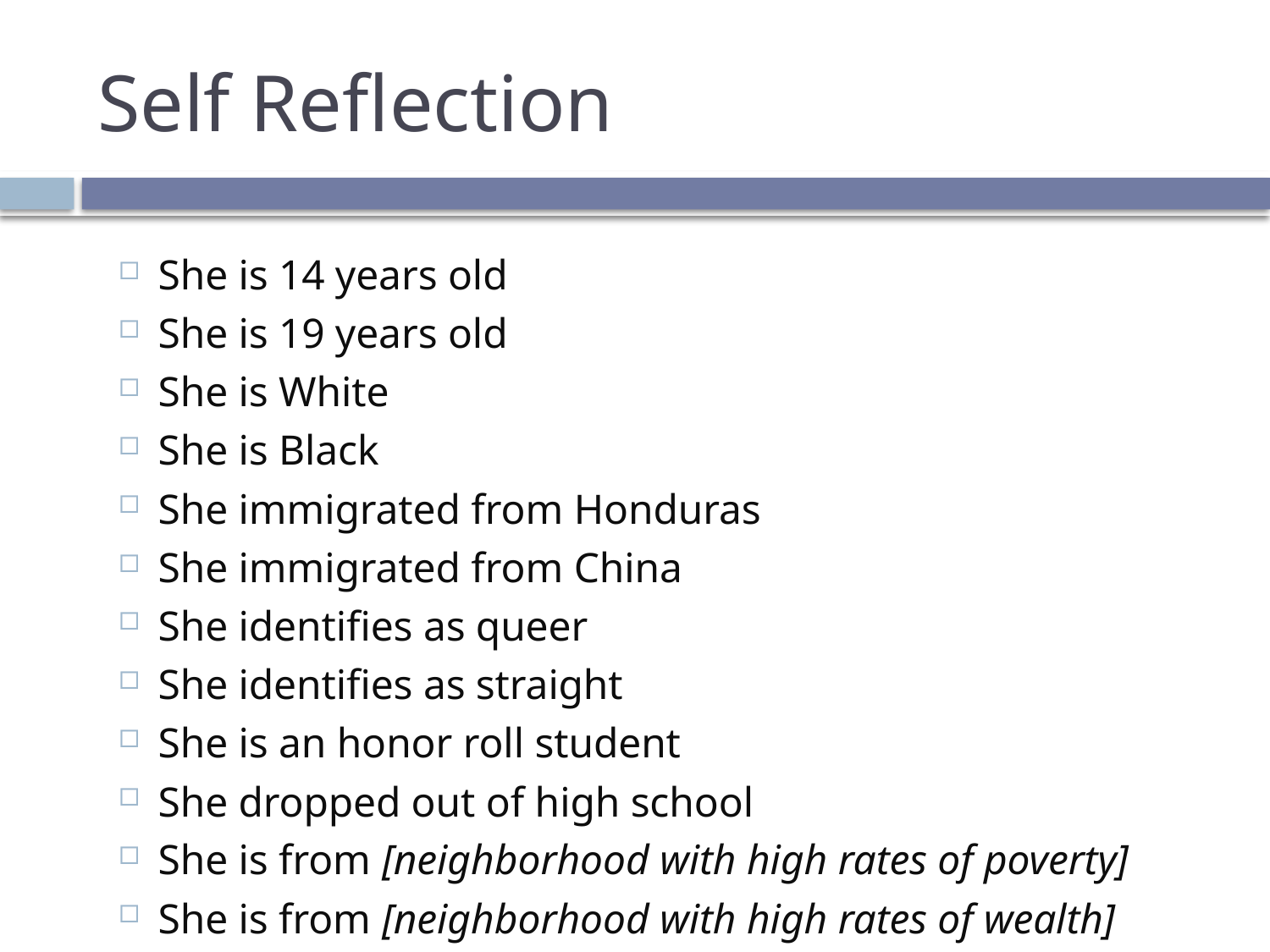

# Self Reflection
She is 14 years old
She is 19 years old
She is White
She is Black
She immigrated from Honduras
She immigrated from China
She identifies as queer
She identifies as straight
She is an honor roll student
She dropped out of high school
She is from [neighborhood with high rates of poverty]
She is from [neighborhood with high rates of wealth]

## Slide 10
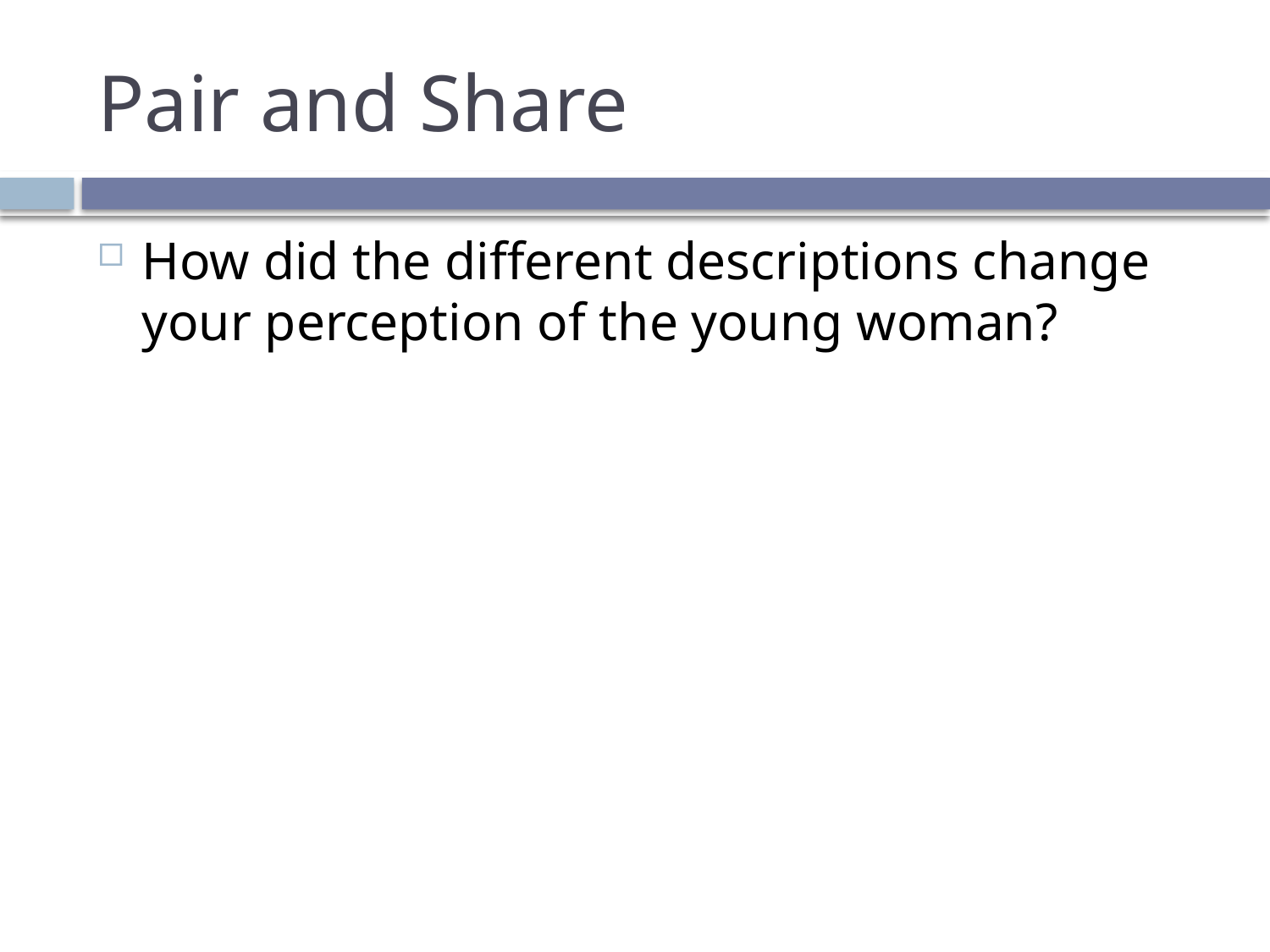

# Pair and Share
How did the different descriptions change your perception of the young woman?

## Slide 11
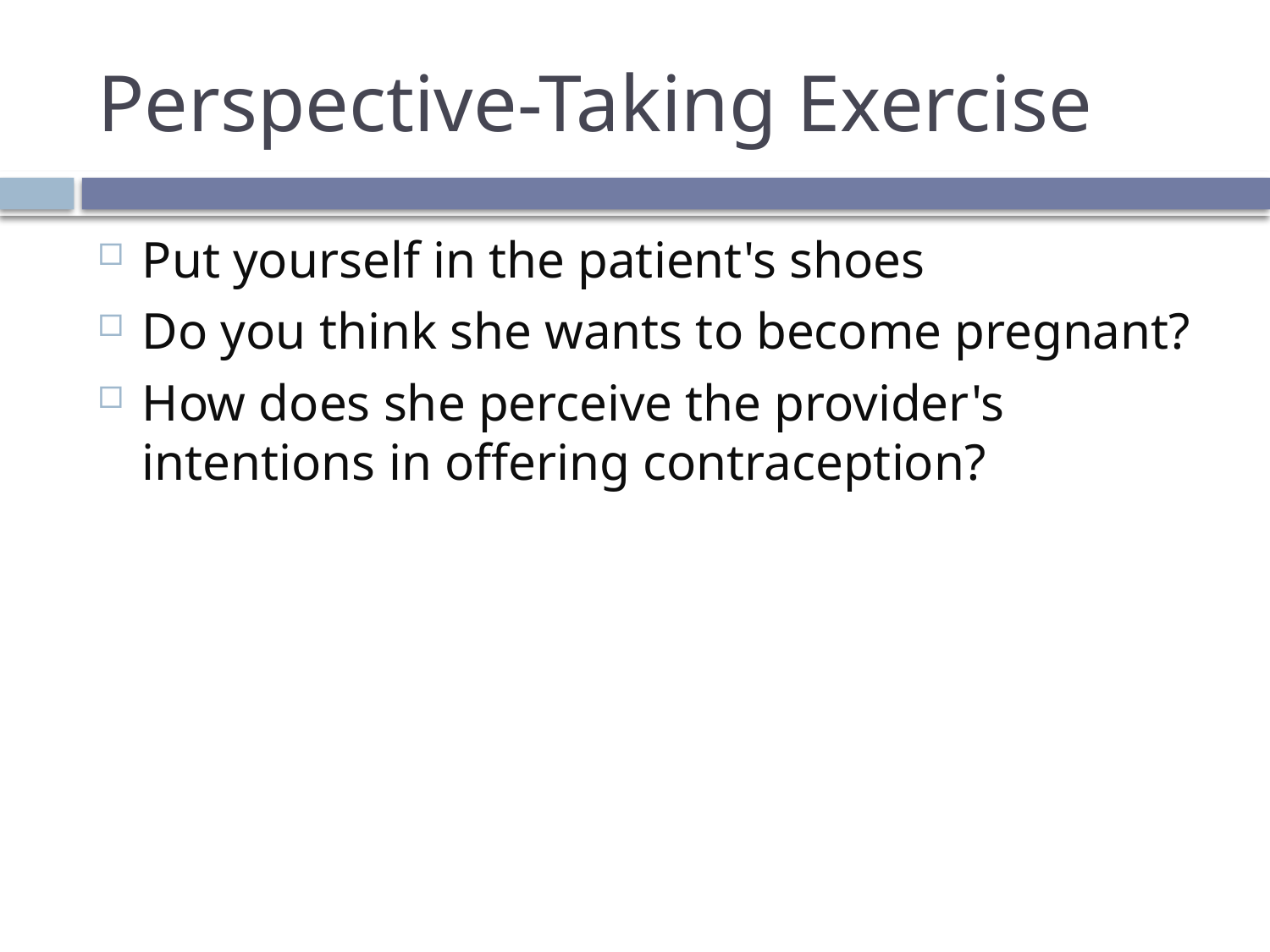

# Perspective-Taking Exercise
Put yourself in the patient's shoes
Do you think she wants to become pregnant?
How does she perceive the provider's intentions in offering contraception?

## Slide 12
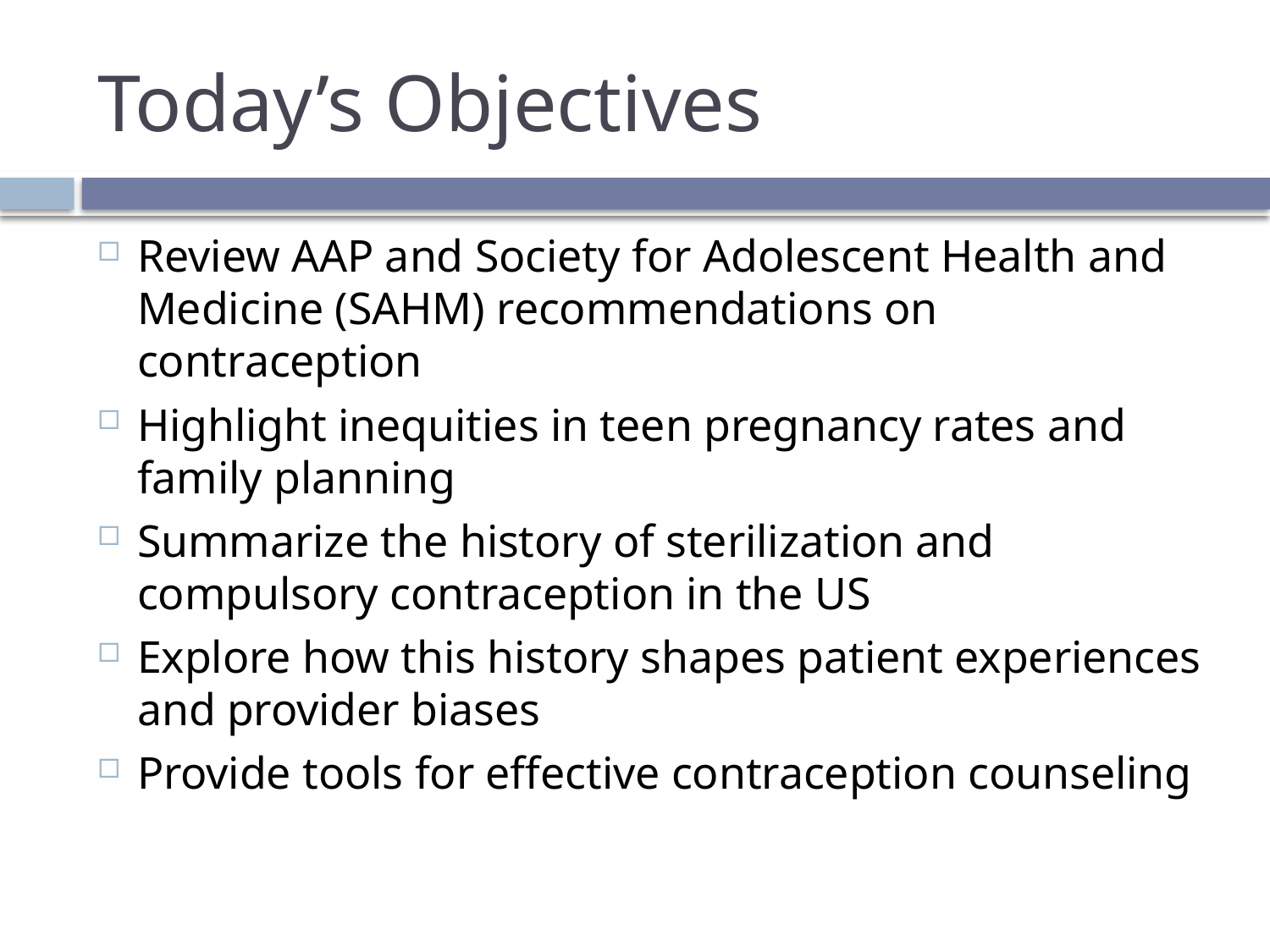

# Today’s Objectives
Review AAP and Society for Adolescent Health and Medicine (SAHM) recommendations on contraception
Highlight inequities in teen pregnancy rates and family planning
Summarize the history of sterilization and compulsory contraception in the US
Explore how this history shapes patient experiences and provider biases
Provide tools for effective contraception counseling

## Slide 13
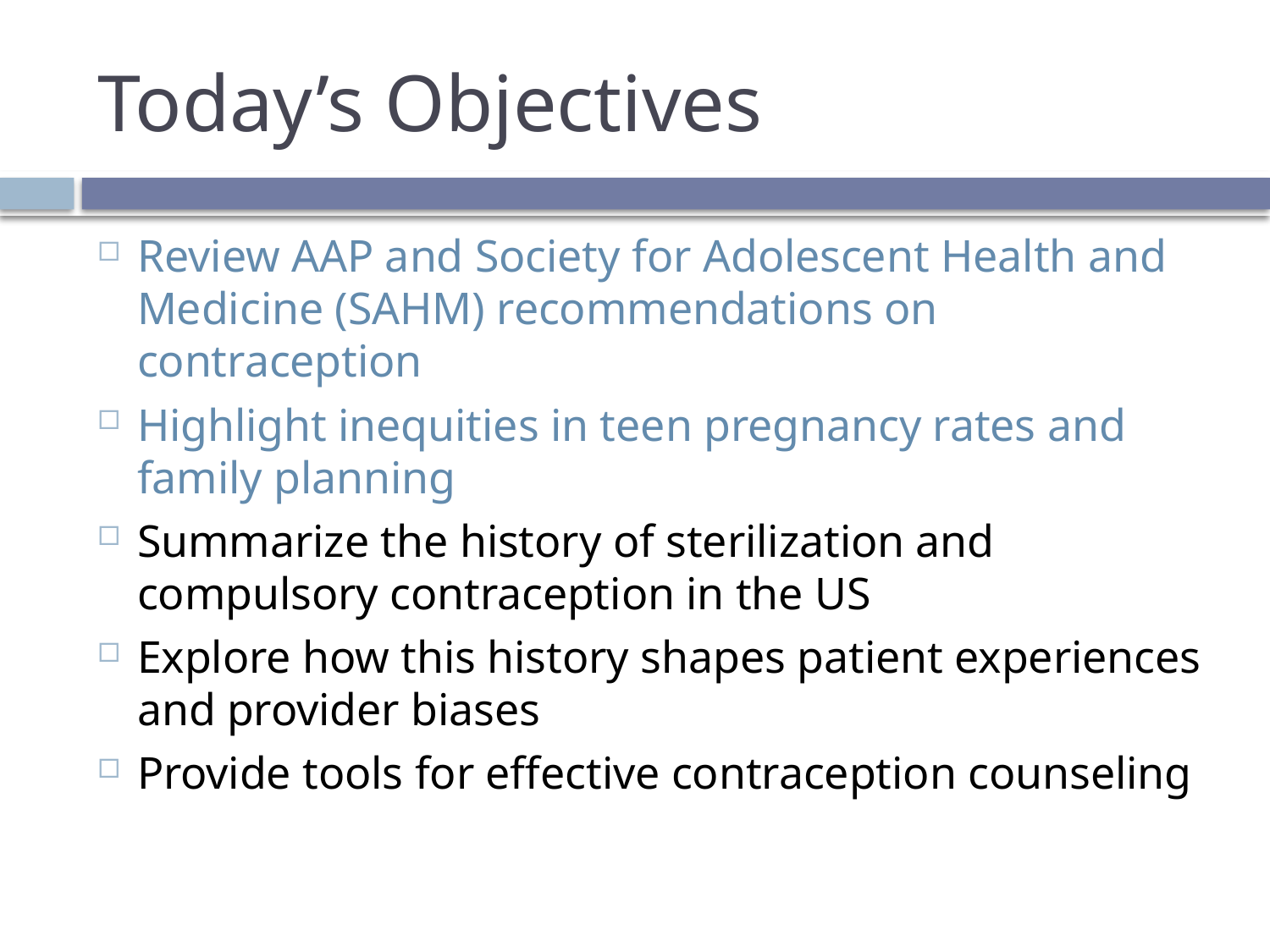

# Today’s Objectives
Review AAP and Society for Adolescent Health and Medicine (SAHM) recommendations on contraception
Highlight inequities in teen pregnancy rates and family planning
Summarize the history of sterilization and compulsory contraception in the US
Explore how this history shapes patient experiences and provider biases
Provide tools for effective contraception counseling

## Slide 14
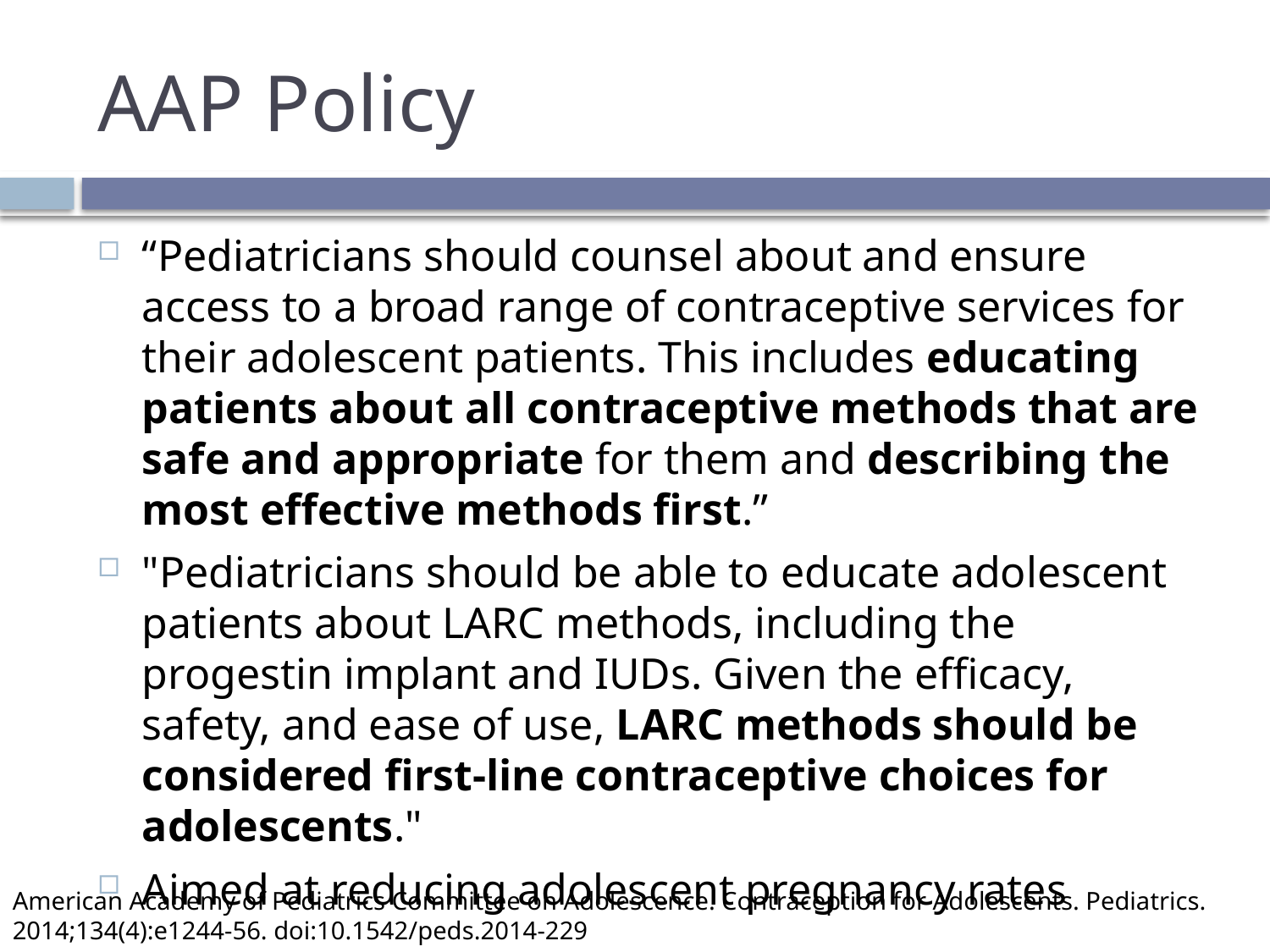

# AAP Policy
“Pediatricians should counsel about and ensure access to a broad range of contraceptive services for their adolescent patients. This includes educating patients about all contraceptive methods that are safe and appropriate for them and describing the most effective methods first.”
"Pediatricians should be able to educate adolescent patients about LARC methods, including the progestin implant and IUDs. Given the efficacy, safety, and ease of use, LARC methods should be considered first-line contraceptive choices for adolescents."
Aimed at reducing adolescent pregnancy rates
American Academy of Pediatrics Committee on Adolescence. Contraception for Adolescents. Pediatrics. 2014;134(4):e1244-56. doi:10.1542/peds.2014-229

## Slide 15
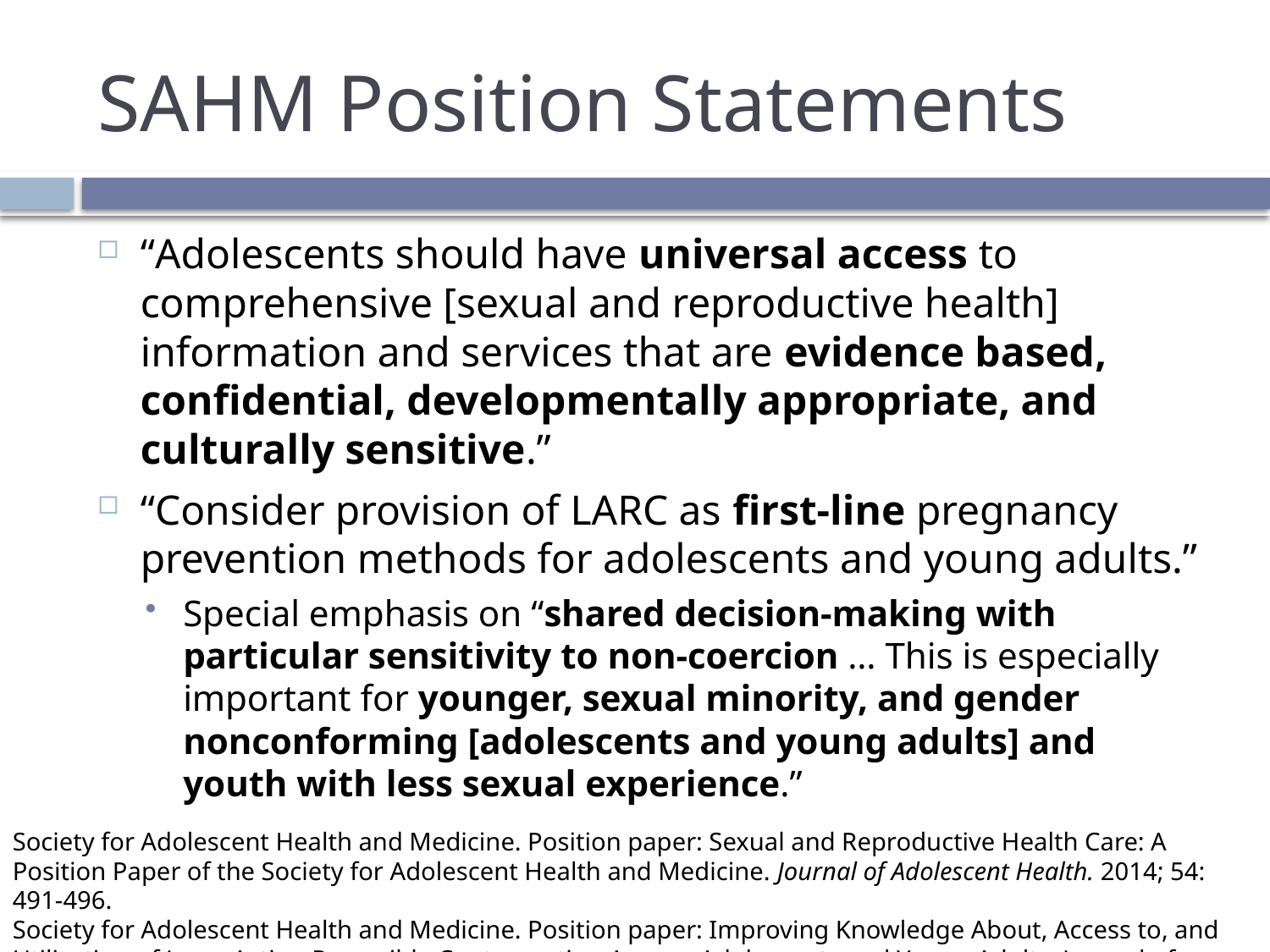

# SAHM Position Statements
“Adolescents should have universal access to comprehensive [sexual and reproductive health] information and services that are evidence based, confidential, developmentally appropriate, and culturally sensitive.”
“Consider provision of LARC as first-line pregnancy prevention methods for adolescents and young adults.”
Special emphasis on “shared decision-making with particular sensitivity to non-coercion … This is especially important for younger, sexual minority, and gender nonconforming [adolescents and young adults] and youth with less sexual experience.”
Society for Adolescent Health and Medicine. Position paper: Sexual and Reproductive Health Care: A Position Paper of the Society for Adolescent Health and Medicine. Journal of Adolescent Health. 2014; 54: 491-496.
Society for Adolescent Health and Medicine. Position paper: Improving Knowledge About, Access to, and Utilization of Long-Acting Reversible Contraception Among Adolescents and Young Adults. Journal of Adolescent Health; 2017: 60 (472-474).

## Slide 16
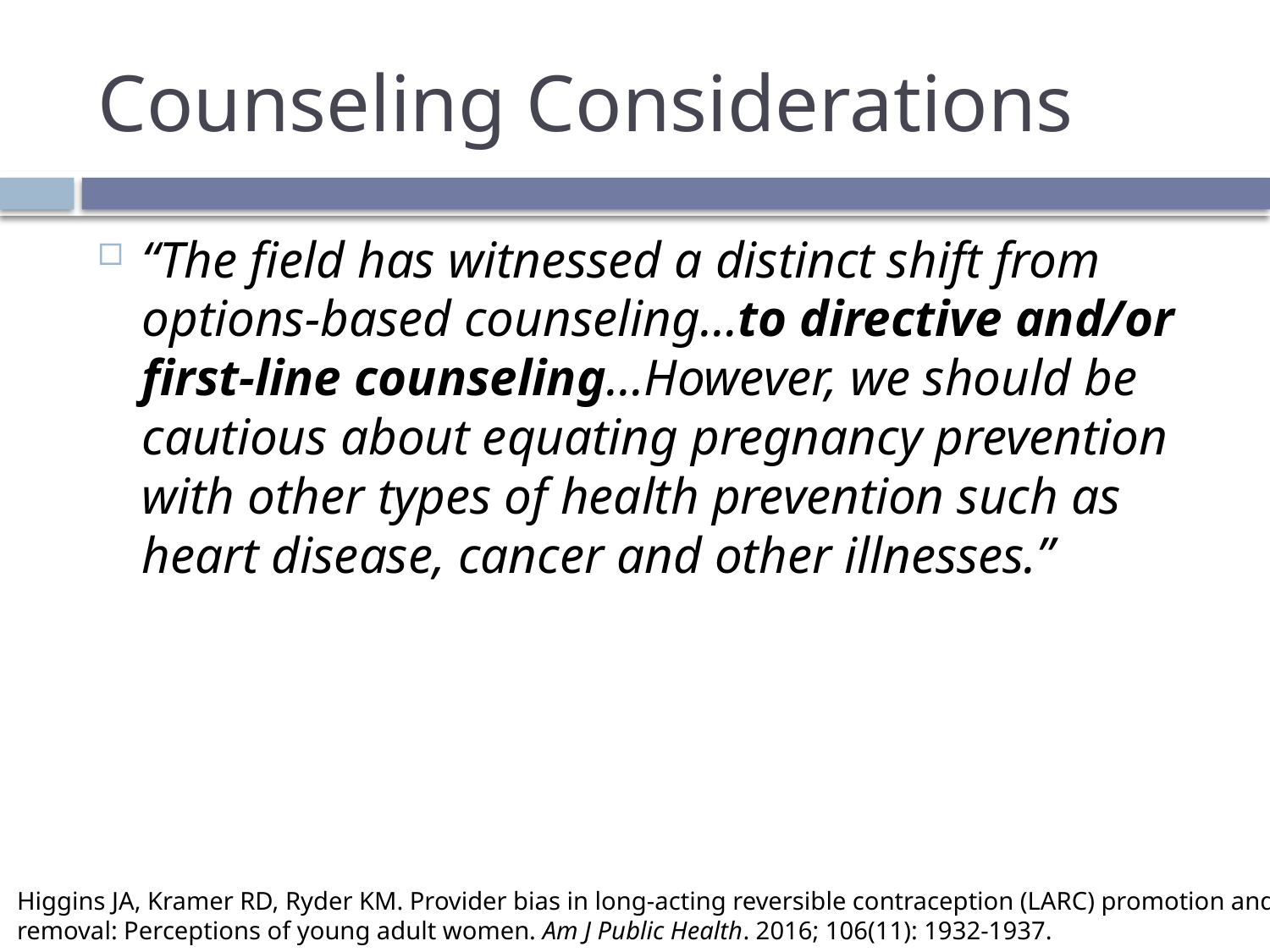

# Counseling Considerations
“The field has witnessed a distinct shift from options-based counseling…to directive and/or first-line counseling…However, we should be cautious about equating pregnancy prevention with other types of health prevention such as heart disease, cancer and other illnesses.”
Higgins JA, Kramer RD, Ryder KM. Provider bias in long-acting reversible contraception (LARC) promotion and removal: Perceptions of young adult women. Am J Public Health. 2016; 106(11): 1932-1937. ​

## Slide 17
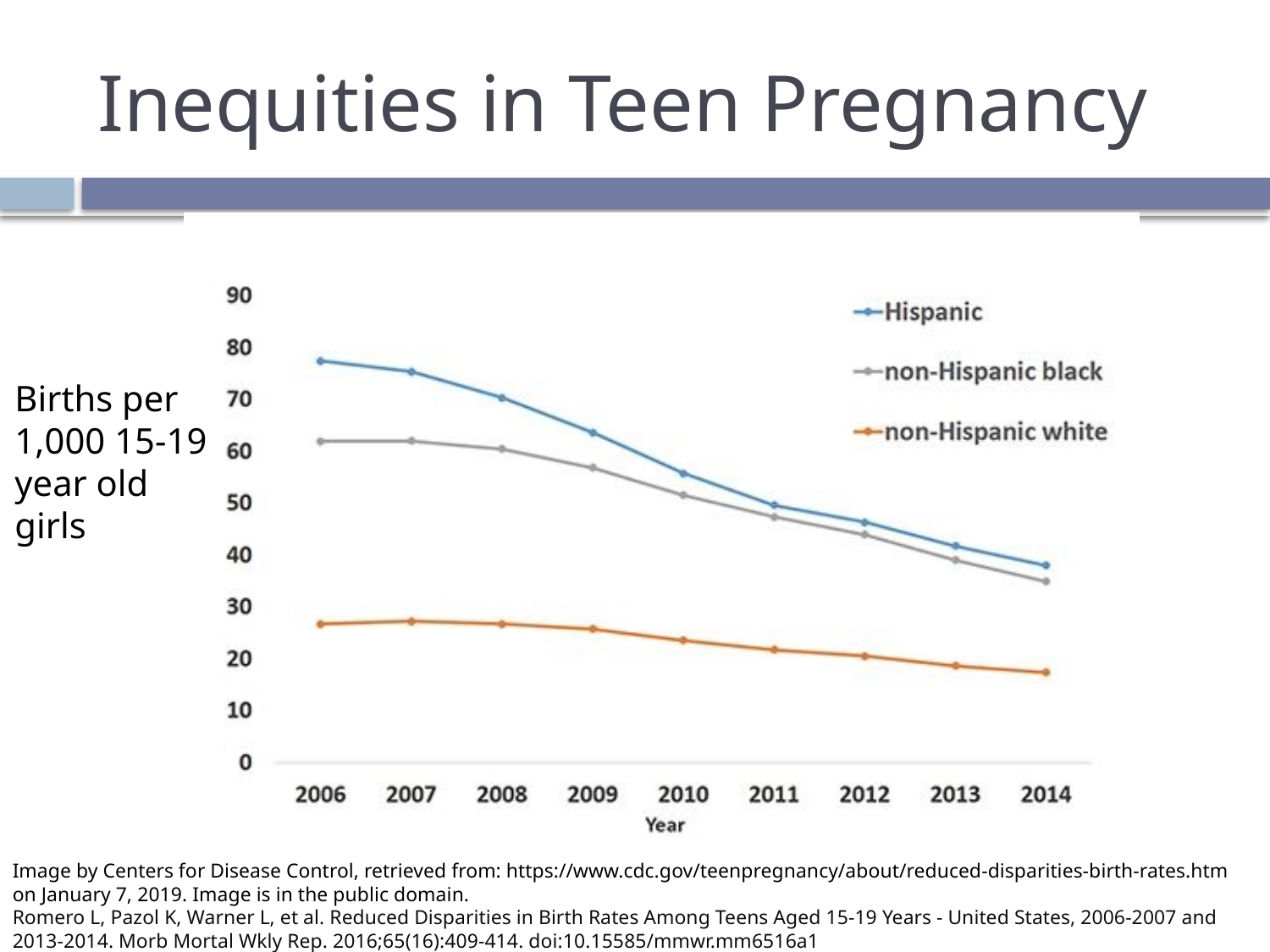

# Inequities in Teen Pregnancy
Births per 1,000 15-19 year old girls
Image by Centers for Disease Control, retrieved from: https://www.cdc.gov/teenpregnancy/about/reduced-disparities-birth-rates.htm on January 7, 2019. Image is in the public domain.
Romero L, Pazol K, Warner L, et al. Reduced Disparities in Birth Rates Among Teens Aged 15-19 Years - United States, 2006-2007 and 2013-2014. Morb Mortal Wkly Rep. 2016;65(16):409-414. doi:10.15585/mmwr.mm6516a1

## Slide 18
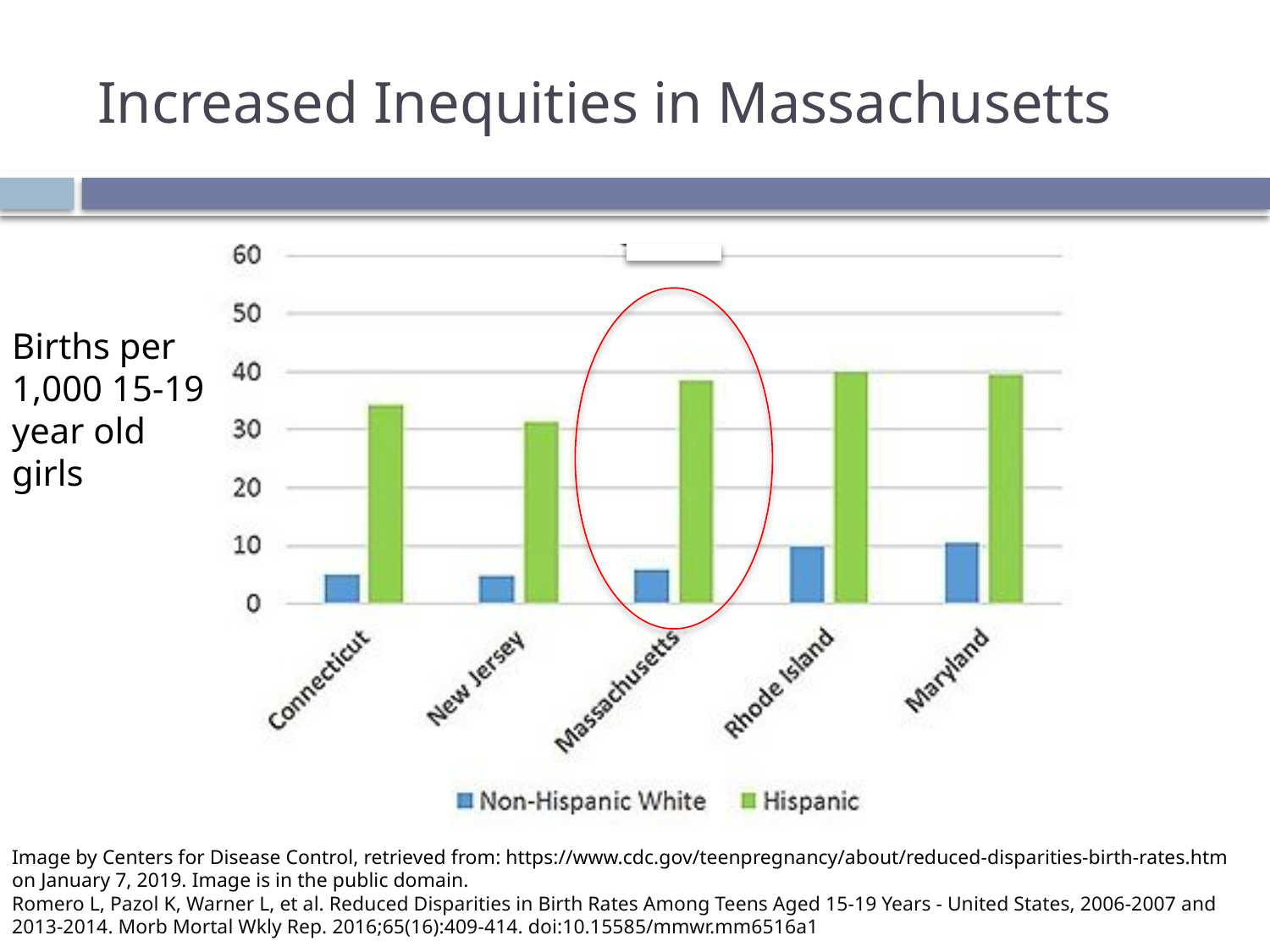

# Increased Inequities in Massachusetts
Births per 1,000 15-19 year old girls
Image by Centers for Disease Control, retrieved from: https://www.cdc.gov/teenpregnancy/about/reduced-disparities-birth-rates.htm on January 7, 2019. Image is in the public domain.
Romero L, Pazol K, Warner L, et al. Reduced Disparities in Birth Rates Among Teens Aged 15-19 Years - United States, 2006-2007 and 2013-2014. Morb Mortal Wkly Rep. 2016;65(16):409-414. doi:10.15585/mmwr.mm6516a1

## Slide 19
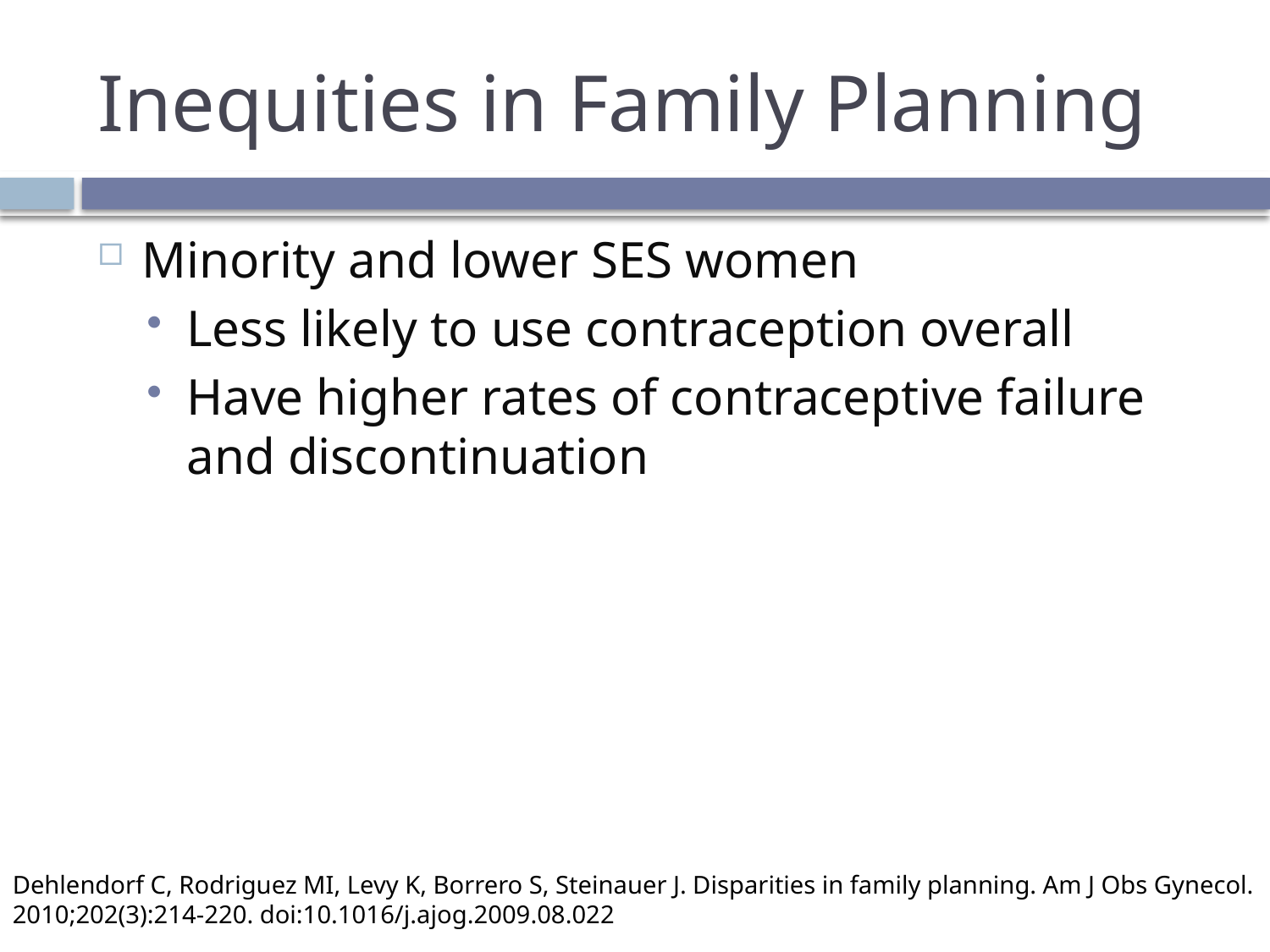

# Inequities in Family Planning
Minority and lower SES women
Less likely to use contraception overall
Have higher rates of contraceptive failure and discontinuation
Dehlendorf C, Rodriguez MI, Levy K, Borrero S, Steinauer J. Disparities in family planning. Am J Obs Gynecol. 2010;202(3):214-220. doi:10.1016/j.ajog.2009.08.022

## Slide 20
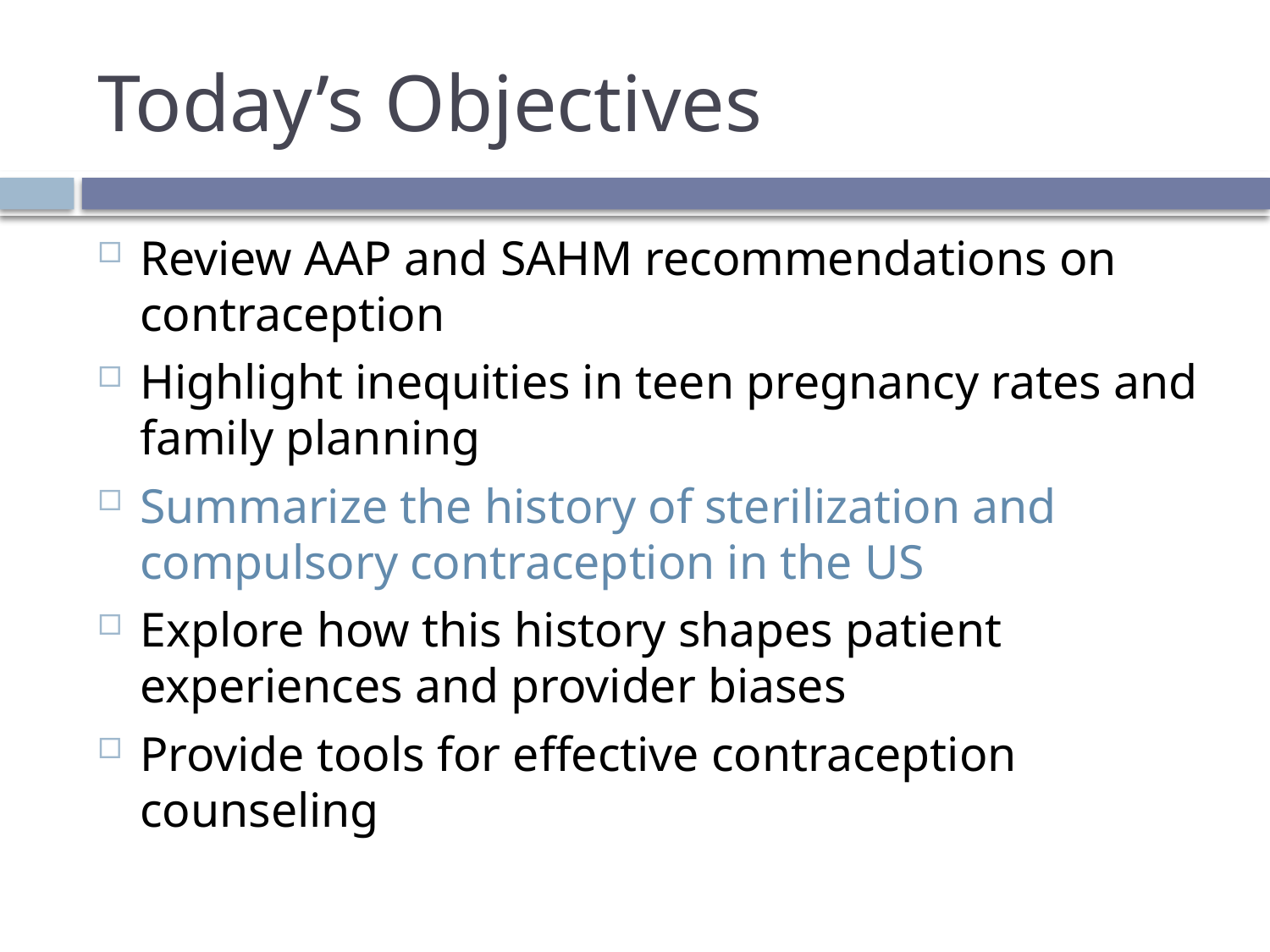

# Today’s Objectives
Review AAP and SAHM recommendations on contraception
Highlight inequities in teen pregnancy rates and family planning
Summarize the history of sterilization and compulsory contraception in the US
Explore how this history shapes patient experiences and provider biases
Provide tools for effective contraception counseling

## Slide 21
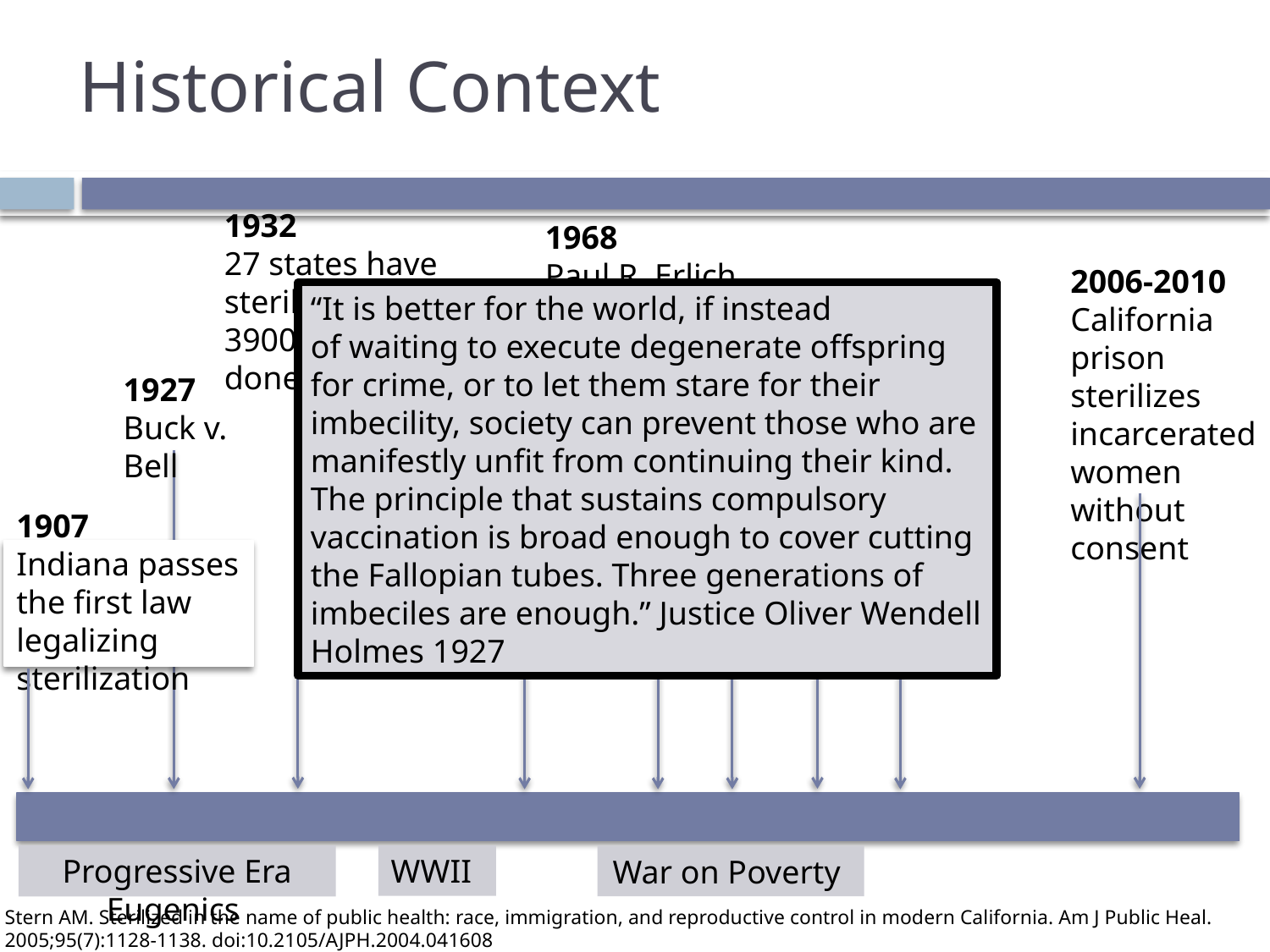

# Historical Context
1932
27 states have sterilization laws; 3900 procedures done nationwide
1968
Paul R. Erlich publishes The Population Bomb
War on Poverty
2006-2010
California prison sterilizes incarcerated women without consent
“It is better for the world, if instead of waiting to execute degenerate offspring for crime, or to let them stare for their imbecility, society can prevent those who are manifestly unfit from continuing their kind. The principle that sustains compulsory vaccination is broad enough to cover cutting the Fallopian tubes. Three generations of imbeciles are enough.” Justice Oliver Wendell Holmes 1927
1927
Buck v. Bell
1975
Madrigal v. Quilligan
1952
John D. Rockefeller establishes the Population Council
1970
Title X
1907
Indiana passes the first law legalizing sterilization
Progressive Era Eugenics
1973
Reif v. Weinberger
WWII
Stern AM. Sterilized in the name of public health: race, immigration, and reproductive control in modern California. Am J Public Heal. 2005;95(7):1128-1138. doi:10.2105/AJPH.2004.041608

## Slide 22
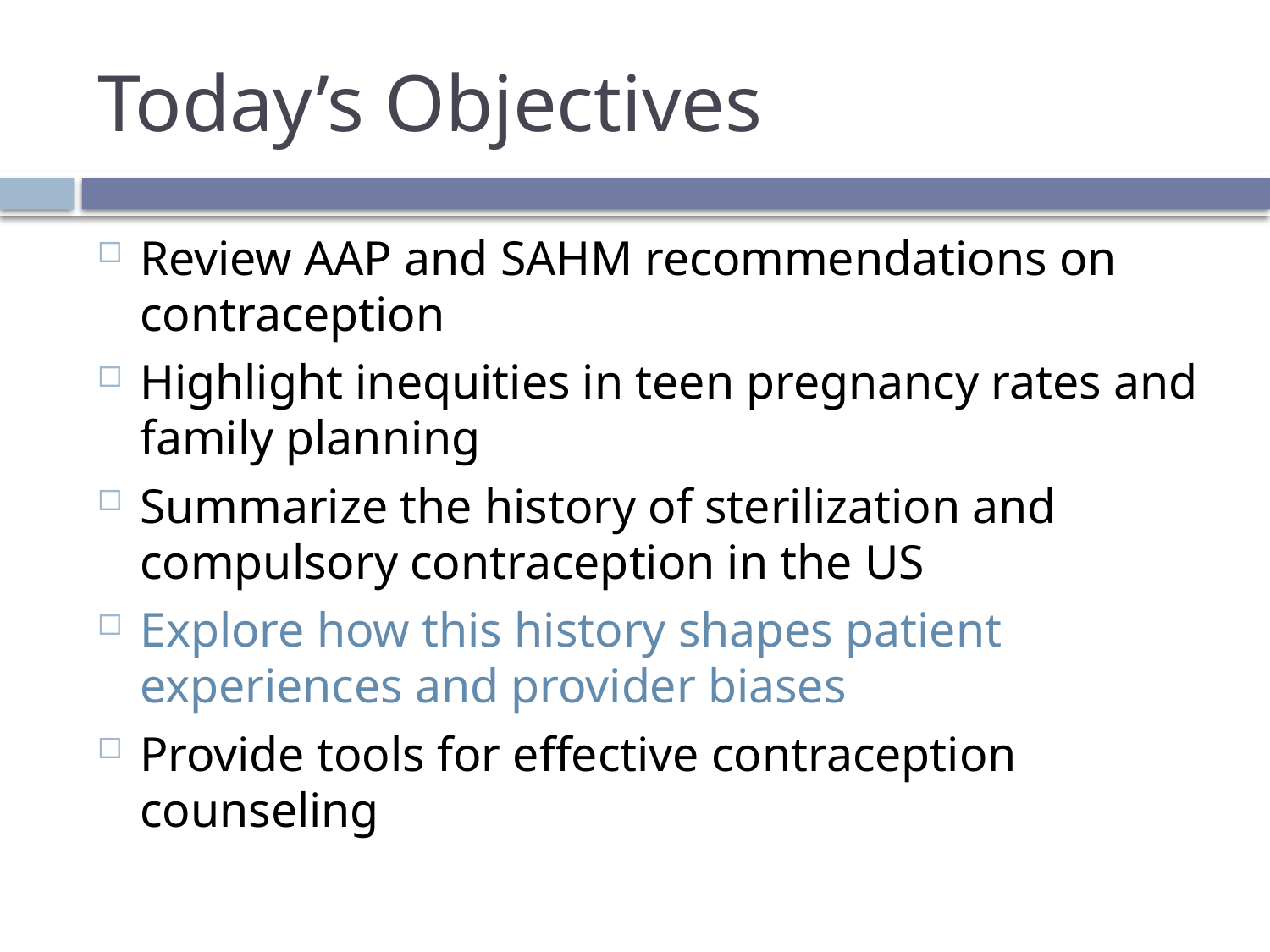

# Today’s Objectives
Review AAP and SAHM recommendations on contraception
Highlight inequities in teen pregnancy rates and family planning
Summarize the history of sterilization and compulsory contraception in the US
Explore how this history shapes patient experiences and provider biases
Provide tools for effective contraception counseling

## Slide 23
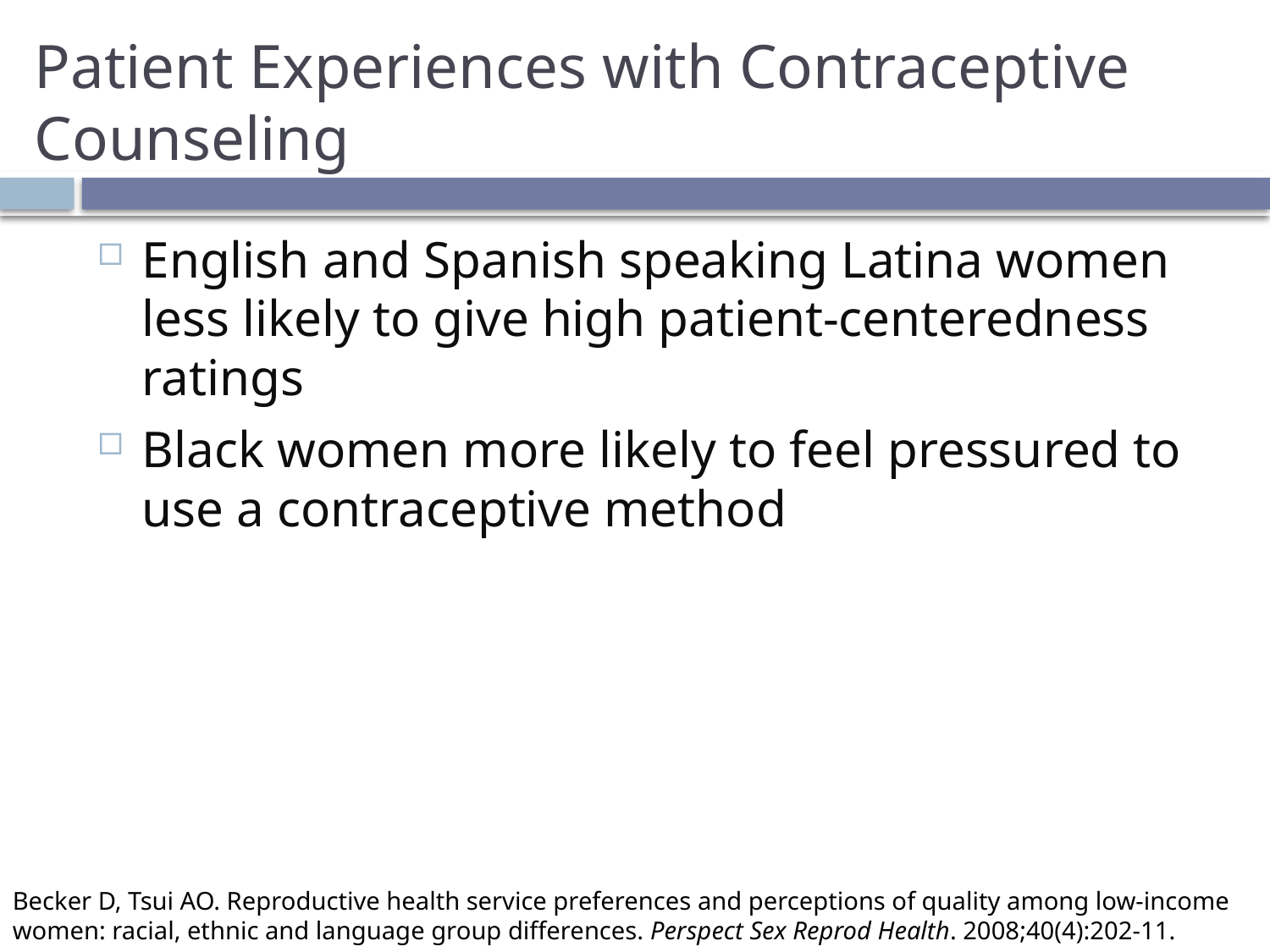

# Patient Experiences with Contraceptive Counseling
English and Spanish speaking Latina women less likely to give high patient-centeredness ratings
Black women more likely to feel pressured to use a contraceptive method
Becker D, Tsui AO. Reproductive health service preferences and perceptions of quality among low-income women: racial, ethnic and language group differences. Perspect Sex Reprod Health. 2008;40(4):202-11.​

## Slide 24
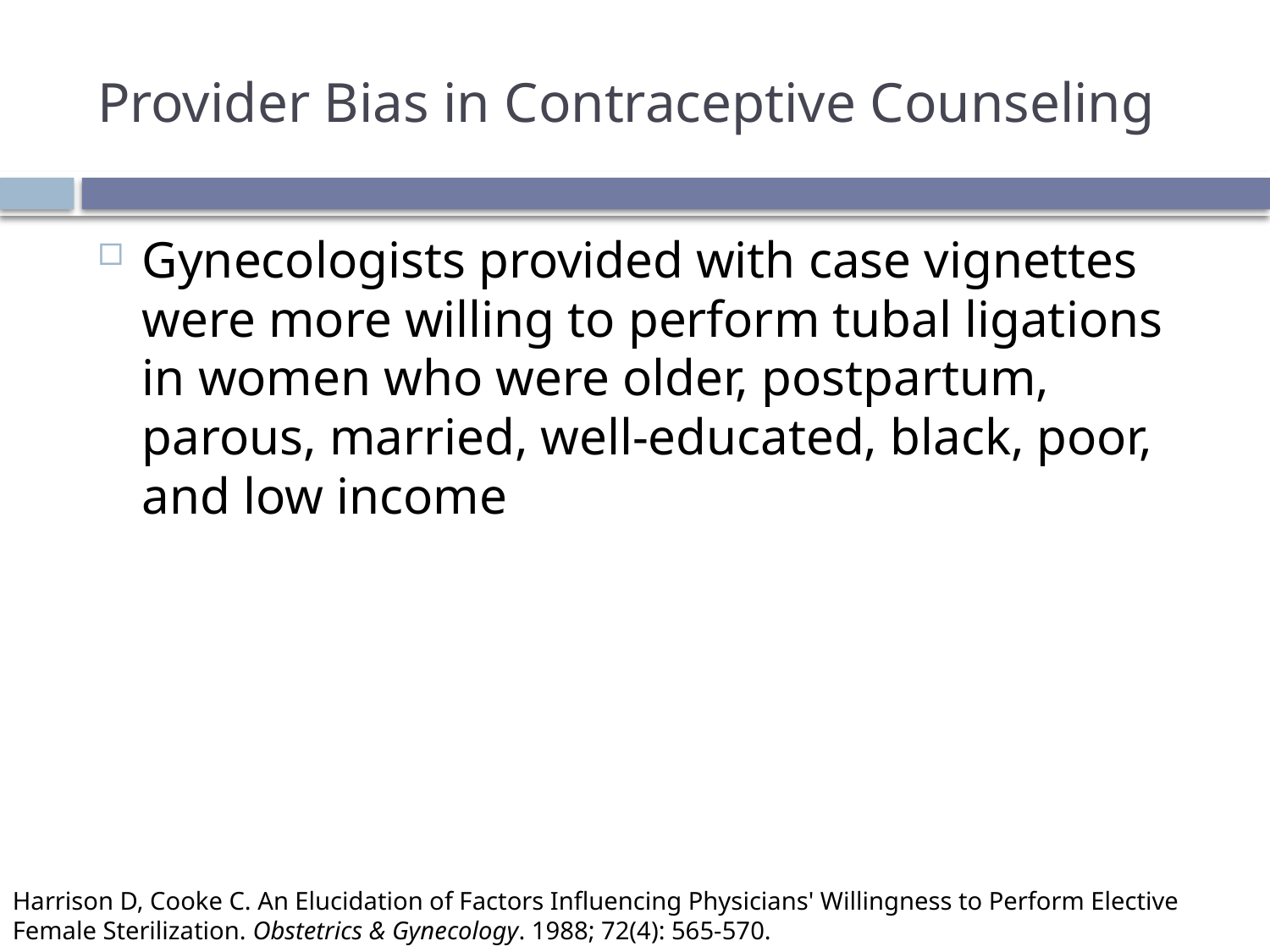

# Provider Bias in Contraceptive Counseling
Gynecologists provided with case vignettes were more willing to perform tubal ligations in women who were older, postpartum, parous, married, well-educated, black, poor, and low income
Harrison D, Cooke C. An Elucidation of Factors Influencing Physicians' Willingness to Perform Elective Female Sterilization. Obstetrics & Gynecology. 1988; 72(4): 565-570.​

## Slide 25
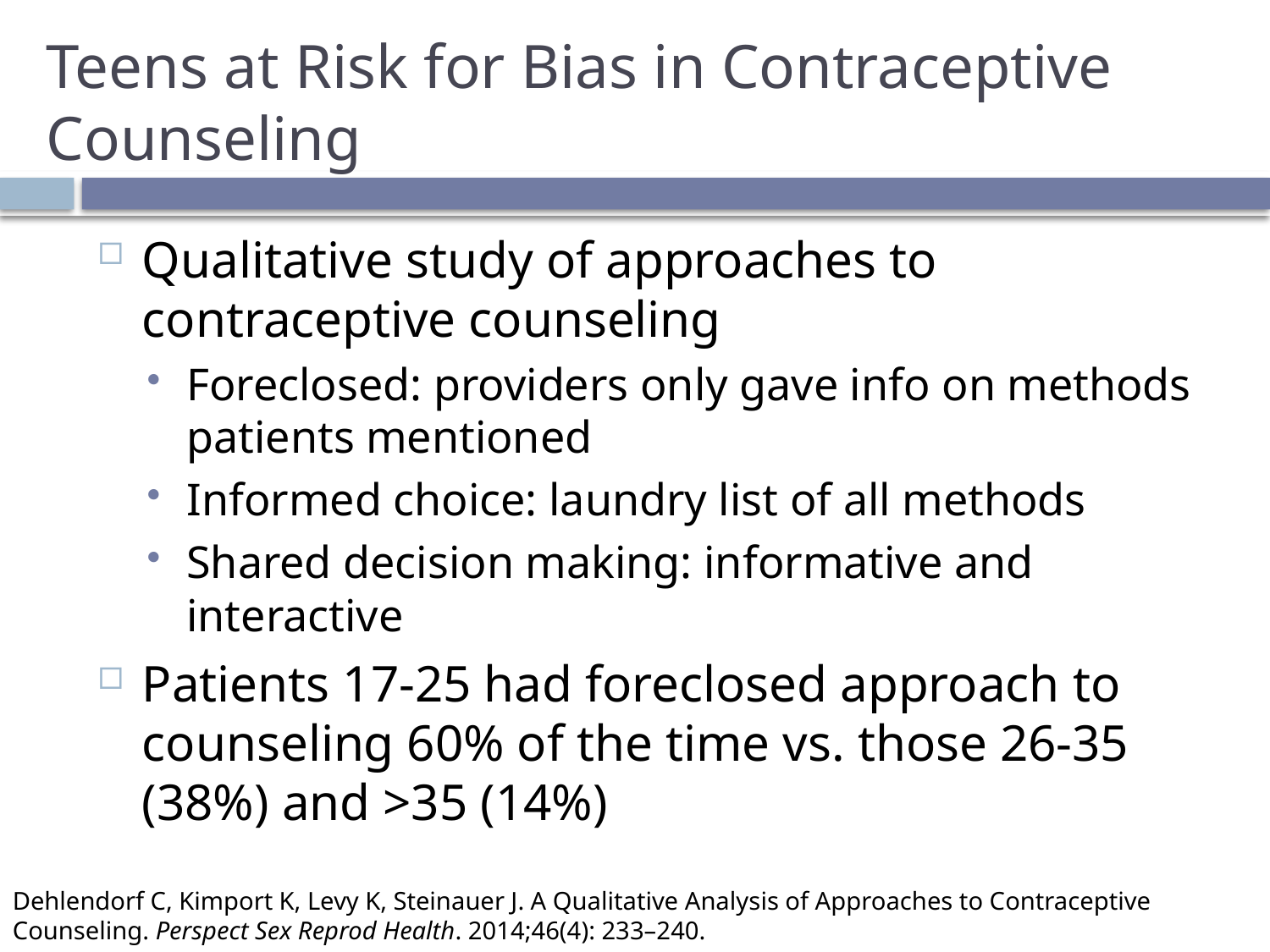

# Teens at Risk for Bias in Contraceptive Counseling
Qualitative study of approaches to contraceptive counseling
Foreclosed: providers only gave info on methods patients mentioned
Informed choice: laundry list of all methods
Shared decision making: informative and interactive
Patients 17-25 had foreclosed approach to counseling 60% of the time vs. those 26-35 (38%) and >35 (14%)
Dehlendorf C, Kimport K, Levy K, Steinauer J. A Qualitative Analysis of Approaches to Contraceptive Counseling. Perspect Sex Reprod Health. 2014;46(4): 233–240. ​

## Slide 26
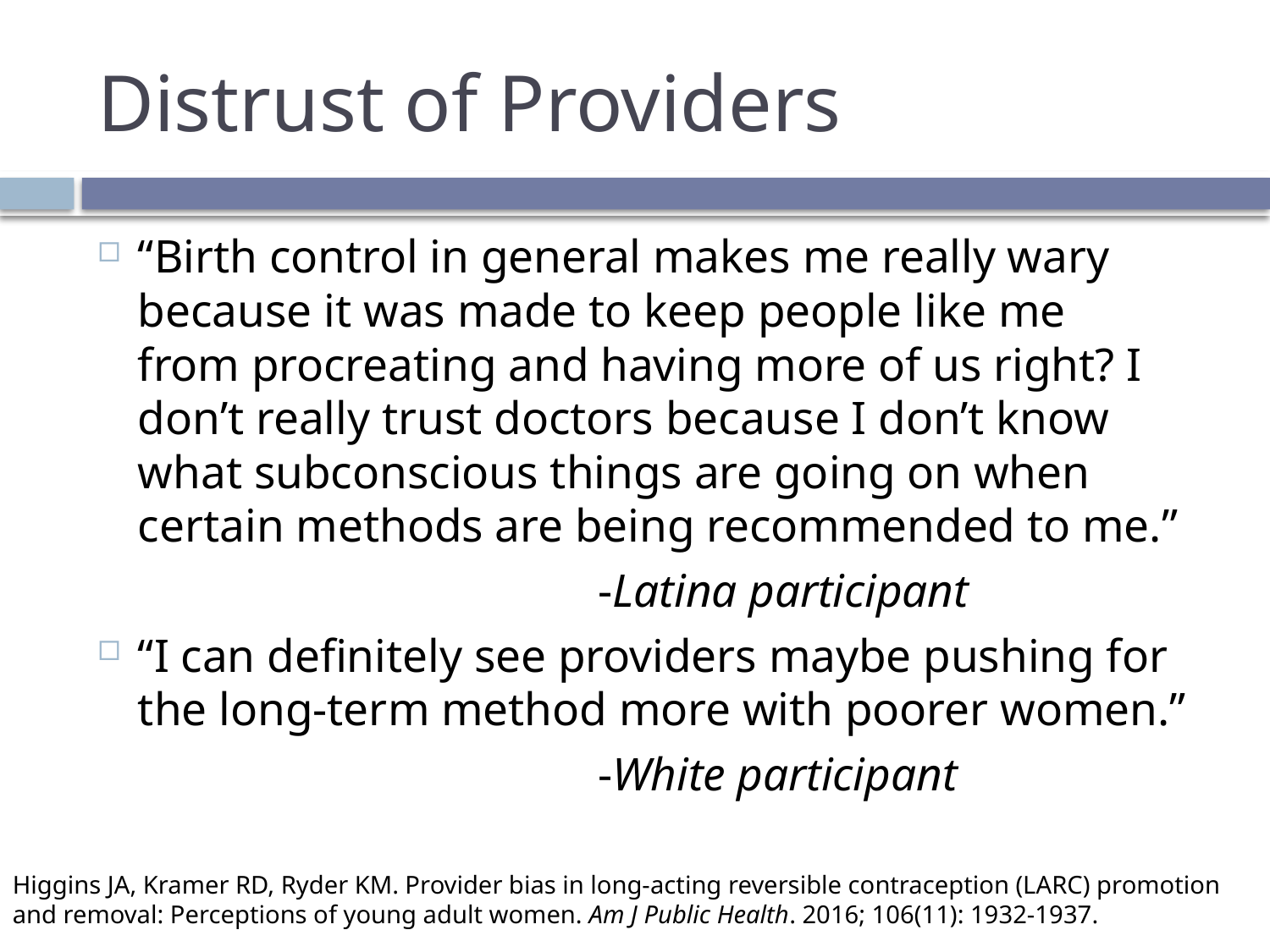

# Distrust of Providers
“Birth control in general makes me really wary because it was made to keep people like me from procreating and having more of us right? I don’t really trust doctors because I don’t know what subconscious things are going on when certain methods are being recommended to me.”
                                           -Latina participant
“I can definitely see providers maybe pushing for the long-term method more with poorer women.”
                                           -White participant
Higgins JA, Kramer RD, Ryder KM. Provider bias in long-acting reversible contraception (LARC) promotion and removal: Perceptions of young adult women. Am J Public Health. 2016; 106(11): 1932-1937. ​

## Slide 27
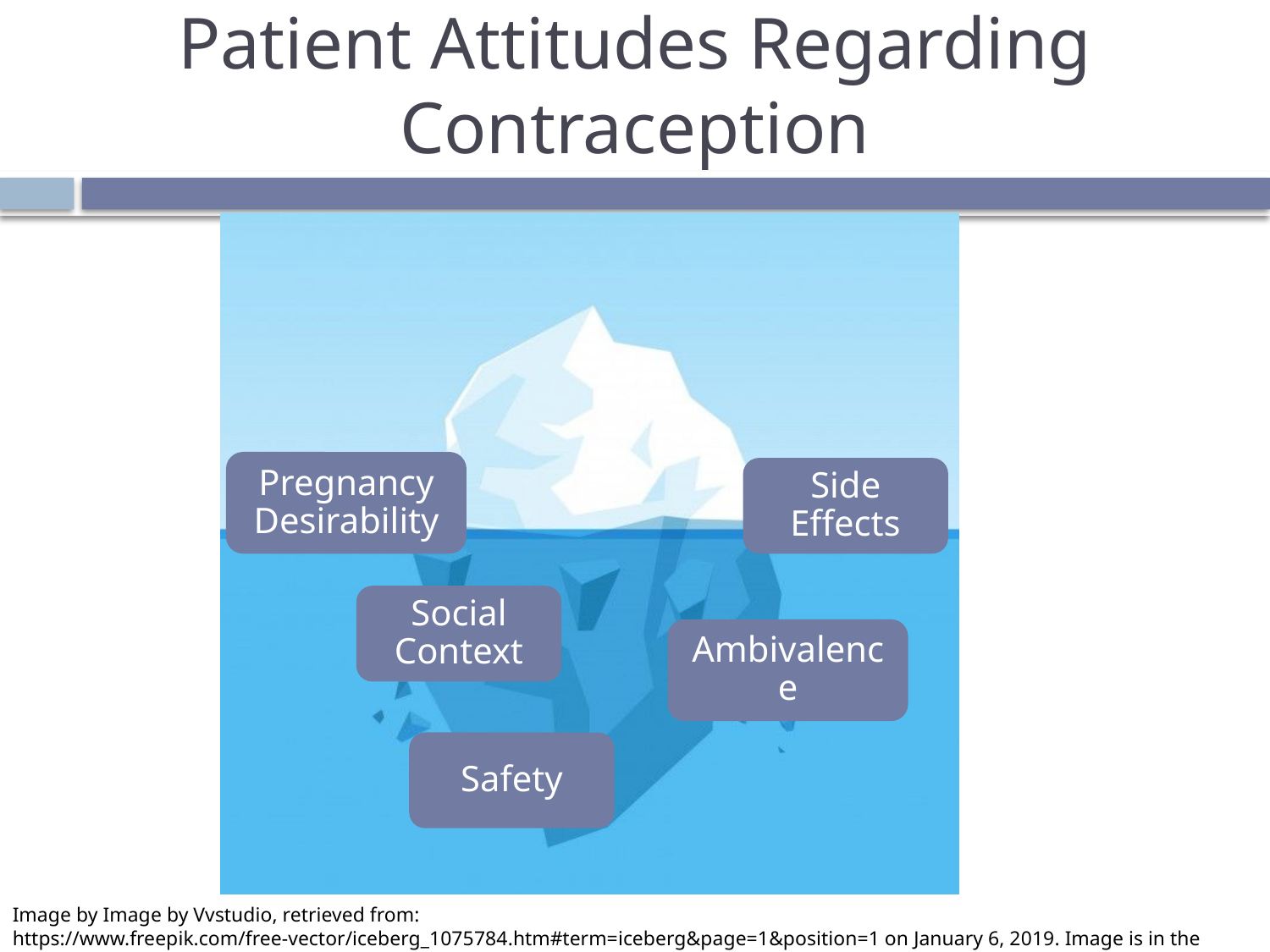

# Patient Attitudes Regarding Contraception
Pregnancy Desirability
Side Effects
Social Context
Ambivalence
Safety
Image by Image by Vvstudio, retrieved from: https://www.freepik.com/free-vector/iceberg_1075784.htm#term=iceberg&page=1&position=1 on January 6, 2019. Image is in the public domain.

## Slide 28
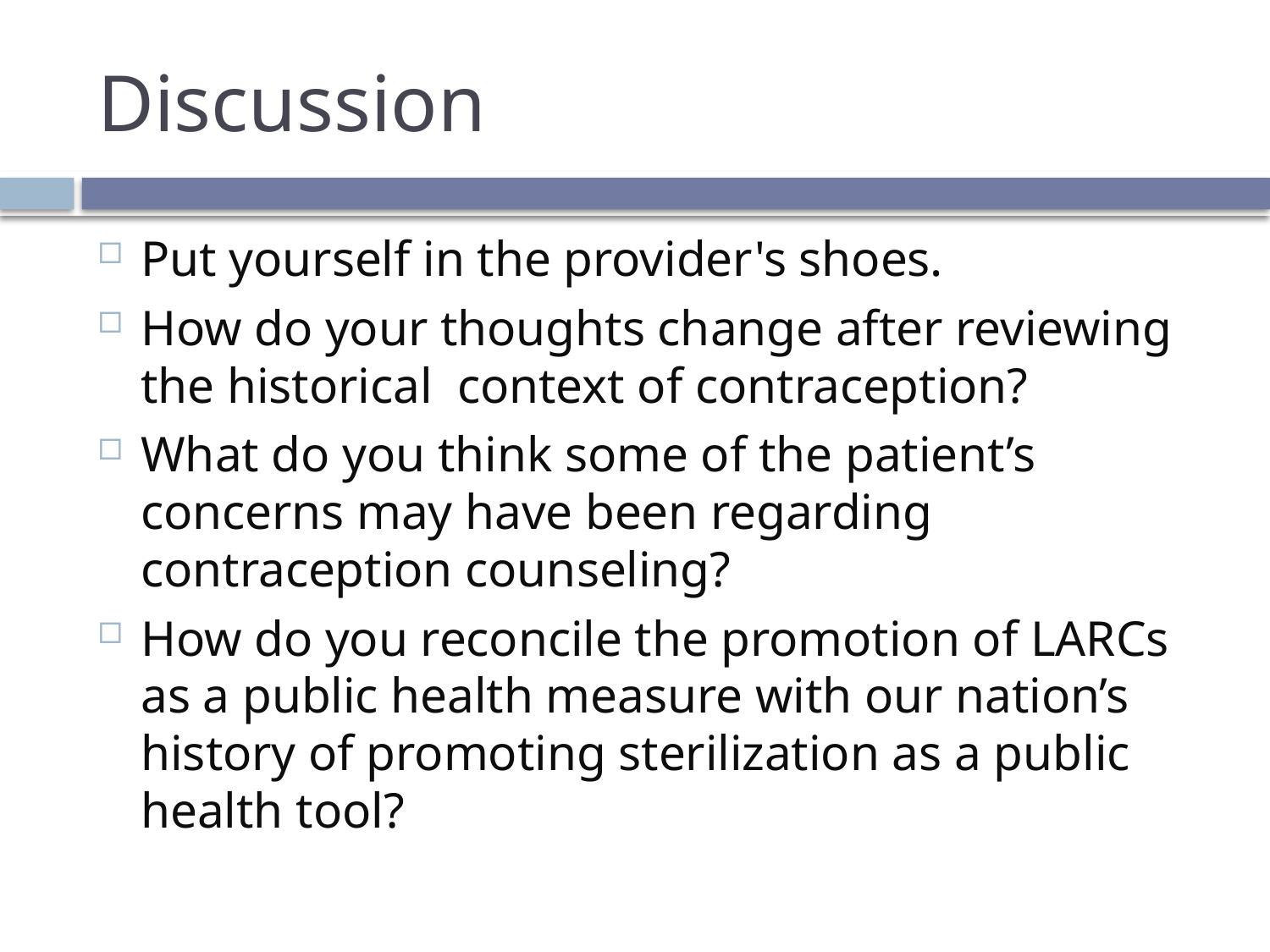

# Discussion
Put yourself in the provider's shoes.
How do your thoughts change after reviewing the historical context of contraception?
What do you think some of the patient’s concerns may have been regarding contraception counseling?
How do you reconcile the promotion of LARCs as a public health measure with our nation’s history of promoting sterilization as a public health tool?

## Slide 29
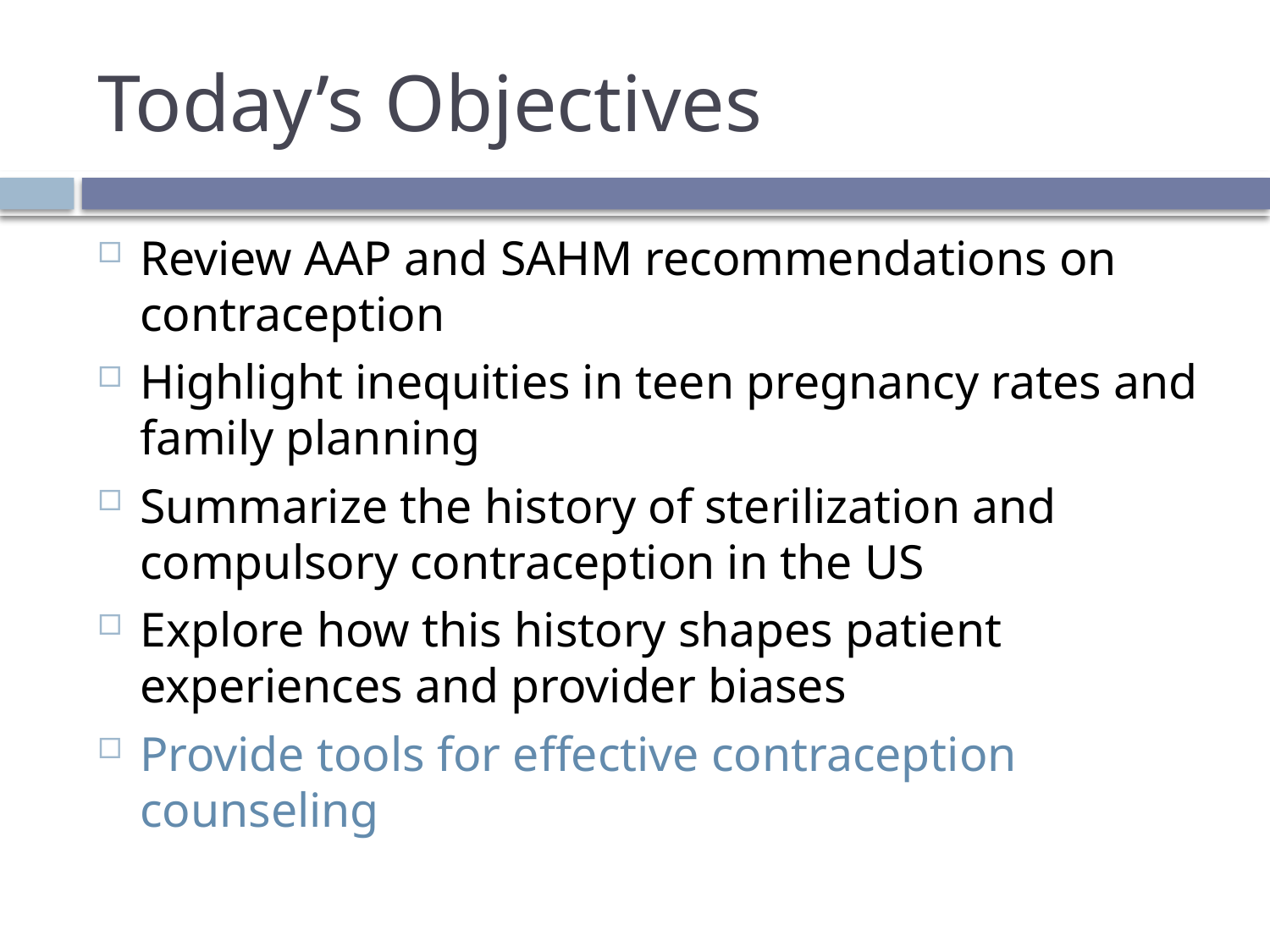

# Today’s Objectives
Review AAP and SAHM recommendations on contraception
Highlight inequities in teen pregnancy rates and family planning
Summarize the history of sterilization and compulsory contraception in the US
Explore how this history shapes patient experiences and provider biases
Provide tools for effective contraception counseling

## Slide 30
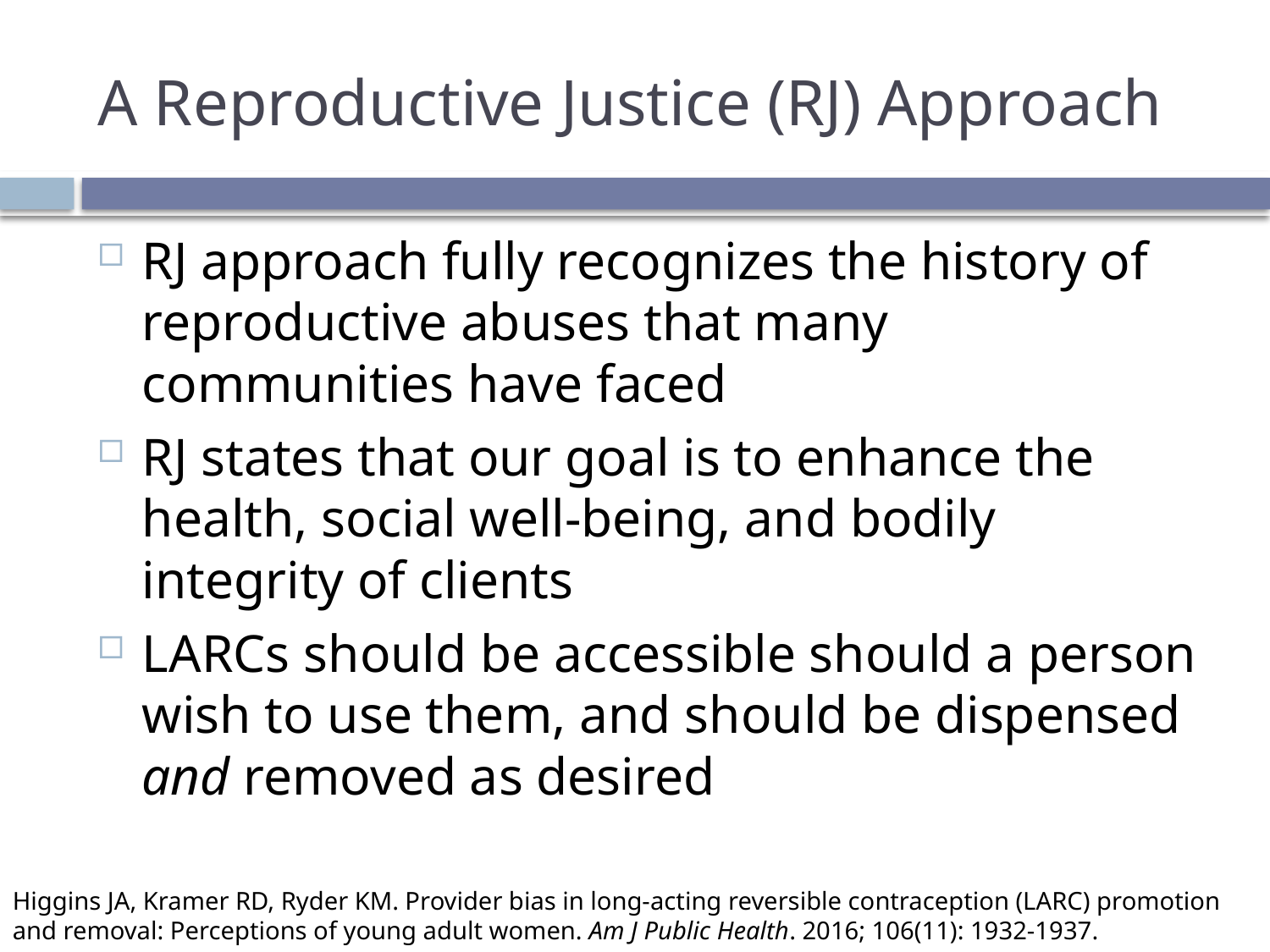

# A Reproductive Justice (RJ) Approach
RJ approach fully recognizes the history of reproductive abuses that many communities have faced
RJ states that our goal is to enhance the health, social well-being, and bodily integrity of clients
LARCs should be accessible should a person wish to use them, and should be dispensed and removed as desired
Higgins JA, Kramer RD, Ryder KM. Provider bias in long-acting reversible contraception (LARC) promotion and removal: Perceptions of young adult women. Am J Public Health. 2016; 106(11): 1932-1937. ​

## Slide 31
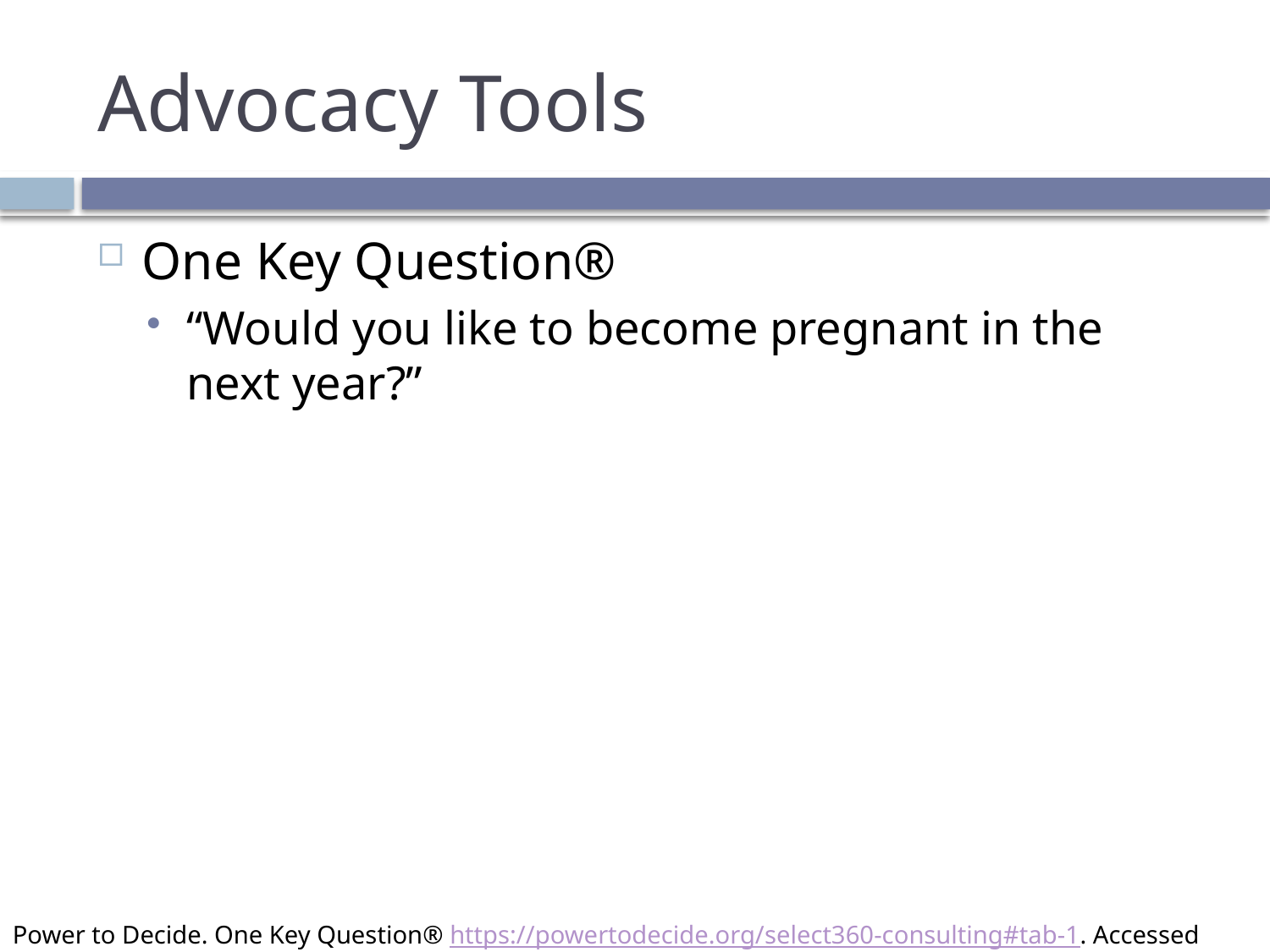

# Advocacy Tools
One Key Question®
“Would you like to become pregnant in the next year?”
Power to Decide. One Key Question® https://powertodecide.org/select360-consulting#tab-1. Accessed February 13, 2019

## Slide 32
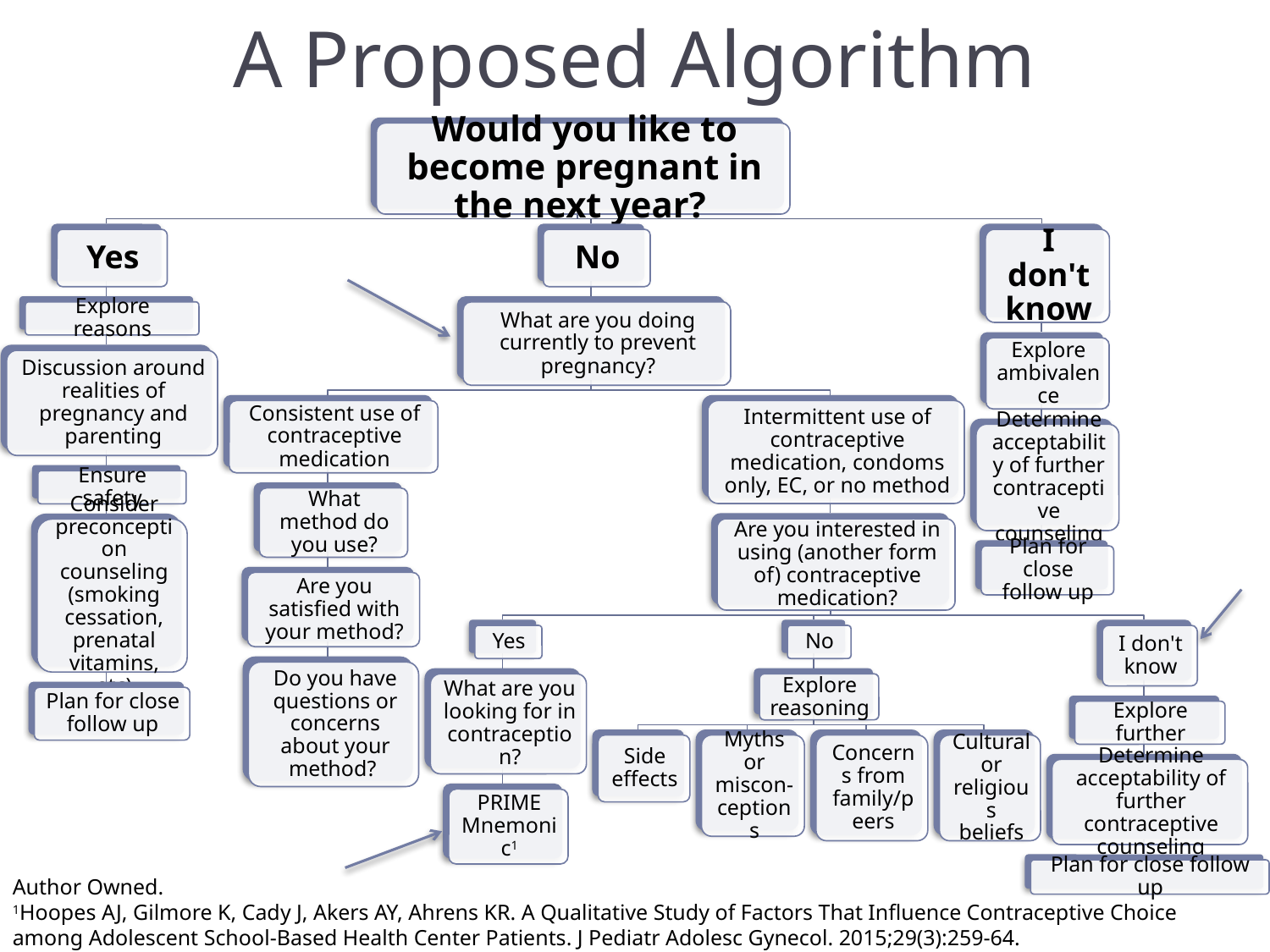

A Proposed Algorithm
Author Owned.
1Hoopes AJ, Gilmore K, Cady J, Akers AY, Ahrens KR. A Qualitative Study of Factors That Influence Contraceptive Choice among Adolescent School-Based Health Center Patients. J Pediatr Adolesc Gynecol. 2015;29(3):259-64.

## Slide 33
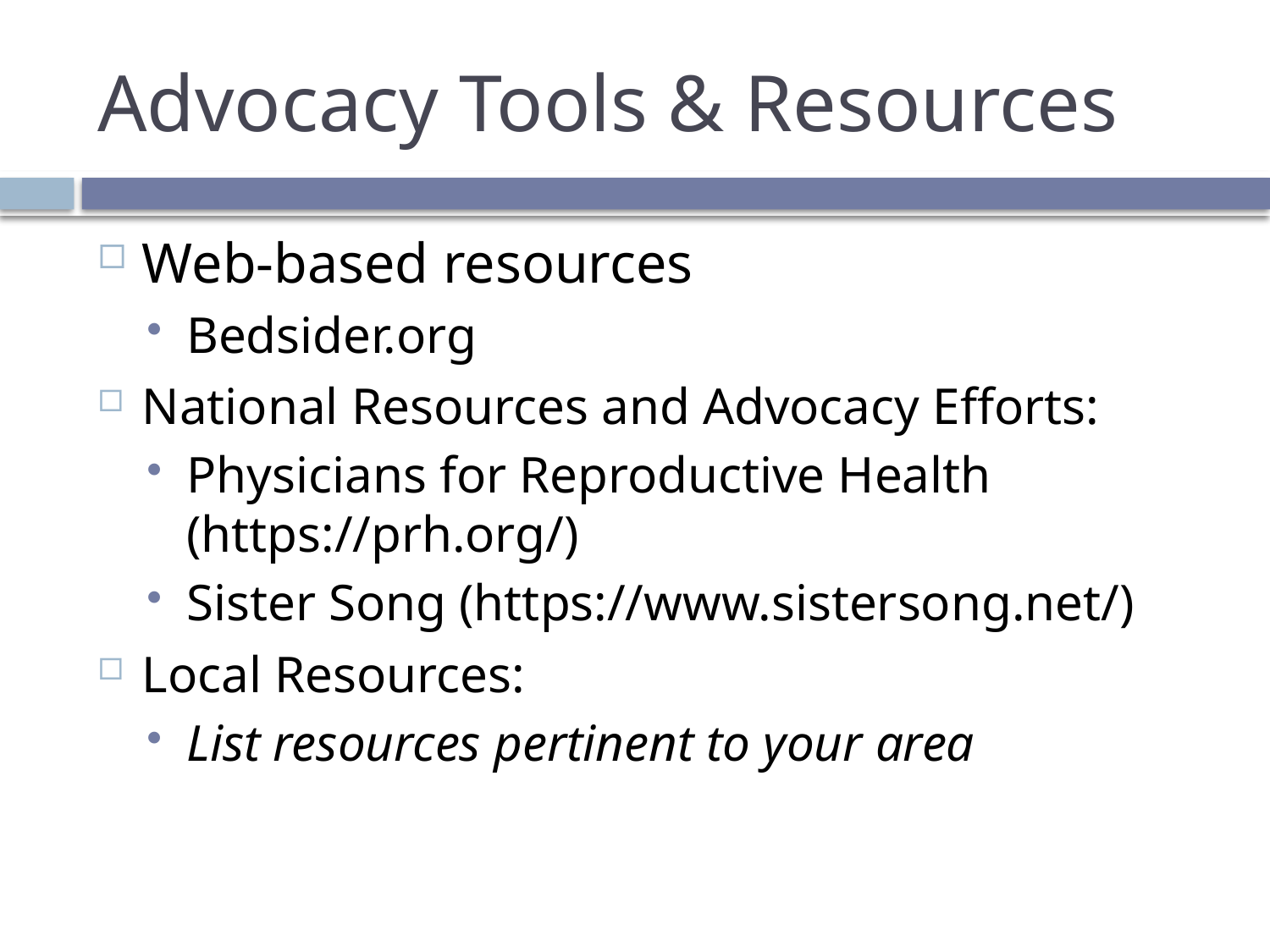

# Advocacy Tools & Resources
Web-based resources
Bedsider.org
National Resources and Advocacy Efforts:
Physicians for Reproductive Health (https://prh.org/)
Sister Song (https://www.sistersong.net/)
Local Resources:
List resources pertinent to your area

## Slide 34
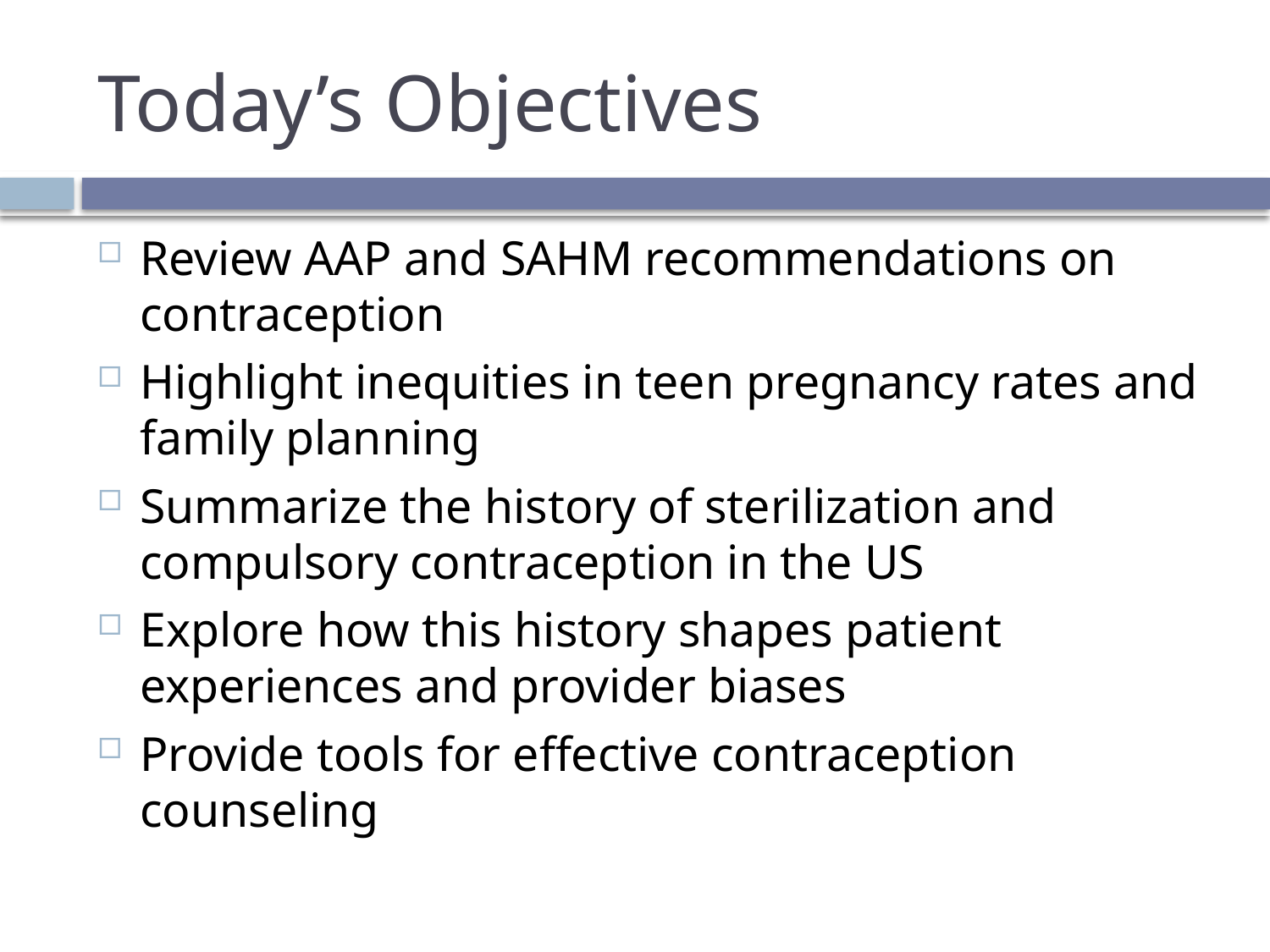

# Today’s Objectives
Review AAP and SAHM recommendations on contraception
Highlight inequities in teen pregnancy rates and family planning
Summarize the history of sterilization and compulsory contraception in the US
Explore how this history shapes patient experiences and provider biases
Provide tools for effective contraception counseling

## Slide 35
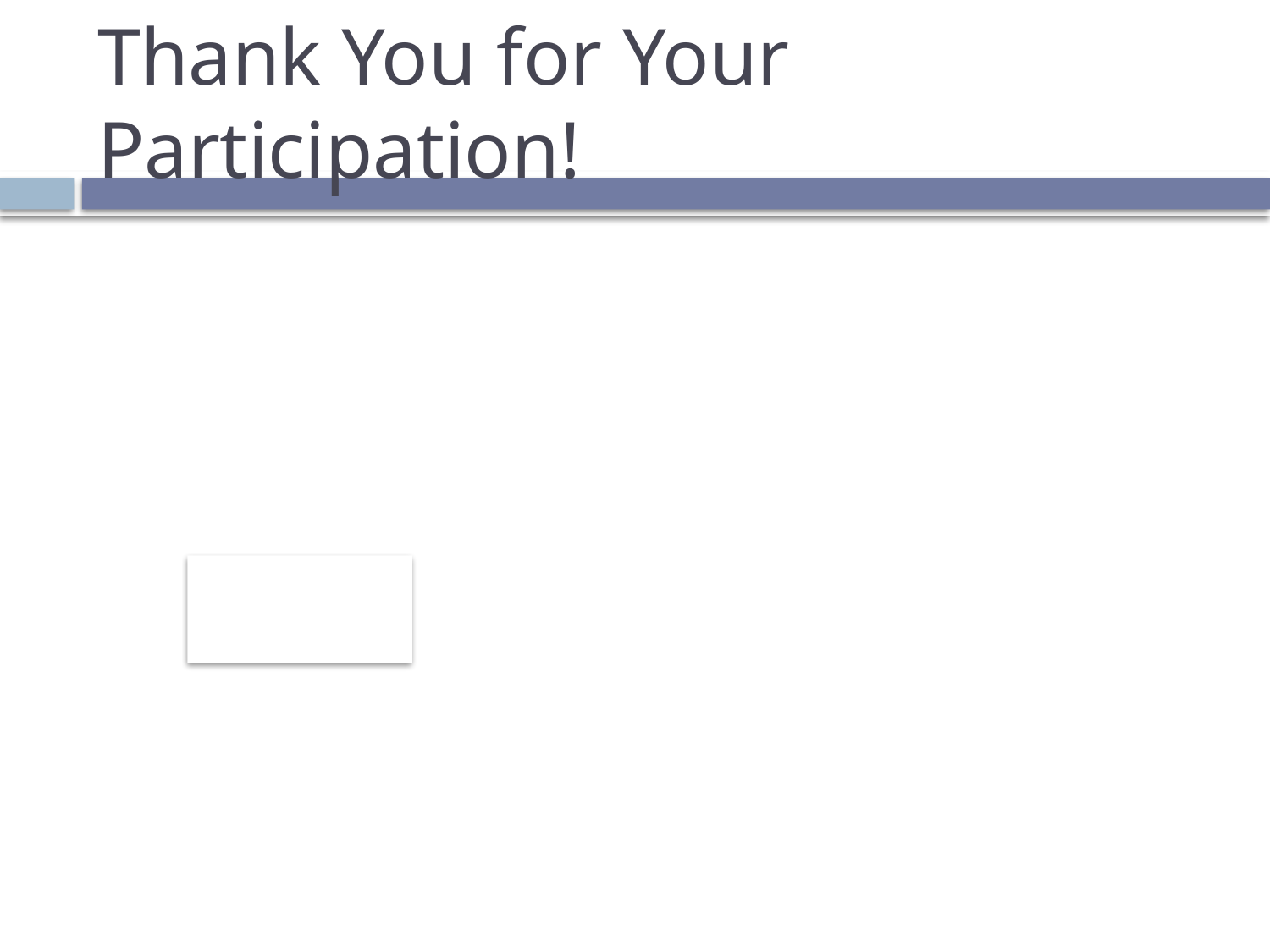

# Thank You for Your Participation!

## Slide 36
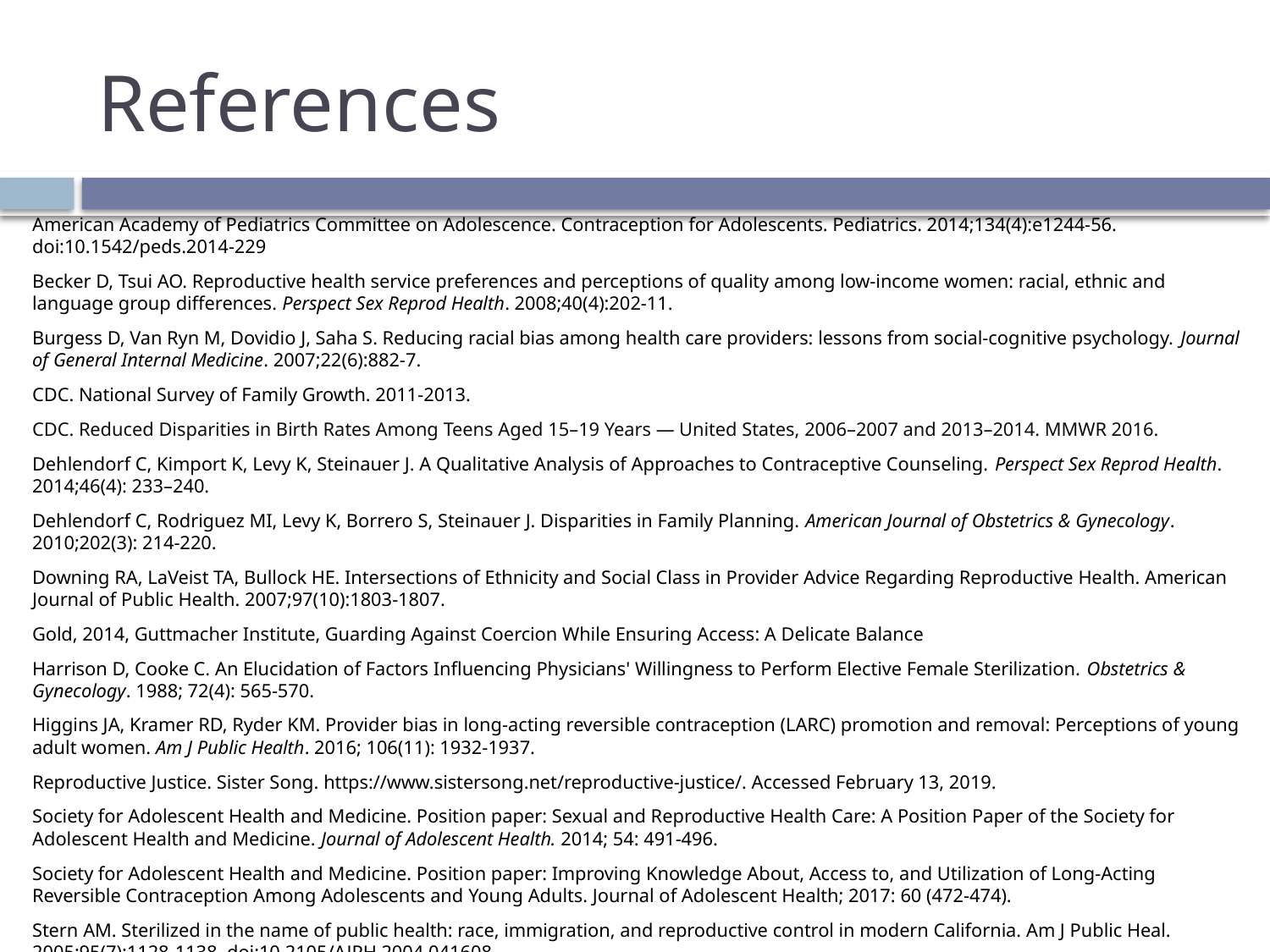

# References
American Academy of Pediatrics Committee on Adolescence. Contraception for Adolescents. Pediatrics. 2014;134(4):e1244-56. doi:10.1542/peds.2014-229
Becker D, Tsui AO. Reproductive health service preferences and perceptions of quality among low-income women: racial, ethnic and language group differences. Perspect Sex Reprod Health. 2008;40(4):202-11.​
Burgess D, Van Ryn M, Dovidio J, Saha S. Reducing racial bias among health care providers: lessons from social-cognitive psychology. Journal of General Internal Medicine. 2007;22(6):882-7.​
CDC. National Survey of Family Growth. 2011-2013.
CDC. Reduced Disparities in Birth Rates Among Teens Aged 15–19 Years — United States, 2006–2007 and 2013–2014. MMWR 2016.
Dehlendorf C, Kimport K, Levy K, Steinauer J. A Qualitative Analysis of Approaches to Contraceptive Counseling. Perspect Sex Reprod Health. 2014;46(4): 233–240. ​
Dehlendorf C, Rodriguez MI, Levy K, Borrero S, Steinauer J. Disparities in Family Planning. American Journal of Obstetrics & Gynecology. 2010;202(3): 214-220. ​
Downing RA, LaVeist TA, Bullock HE. Intersections of Ethnicity and Social Class in Provider Advice Regarding Reproductive Health. American Journal of Public Health. 2007;97(10):1803-1807.
Gold, 2014, Guttmacher Institute, Guarding Against Coercion While Ensuring Access: A Delicate Balance
Harrison D, Cooke C. An Elucidation of Factors Influencing Physicians' Willingness to Perform Elective Female Sterilization. Obstetrics & Gynecology. 1988; 72(4): 565-570.​
Higgins JA, Kramer RD, Ryder KM. Provider bias in long-acting reversible contraception (LARC) promotion and removal: Perceptions of young adult women. Am J Public Health. 2016; 106(11): 1932-1937. ​
Reproductive Justice. Sister Song. https://www.sistersong.net/reproductive-justice/. Accessed February 13, 2019.
Society for Adolescent Health and Medicine. Position paper: Sexual and Reproductive Health Care: A Position Paper of the Society for Adolescent Health and Medicine. Journal of Adolescent Health. 2014; 54: 491-496.
Society for Adolescent Health and Medicine. Position paper: Improving Knowledge About, Access to, and Utilization of Long-Acting Reversible Contraception Among Adolescents and Young Adults. Journal of Adolescent Health; 2017: 60 (472-474).
Stern AM. Sterilized in the name of public health: race, immigration, and reproductive control in modern California. Am J Public Heal. 2005;95(7):1128-1138. doi:10.2105/AJPH.2004.041608
