# Supplementary material for: Health Equity Rounds: An Interdisciplinary Case Conference to Address Implicit Bias and Structural Racism for Faculty and Trainees
Source: MedEdPORTAL. 2019 Nov 22;15:10858. doi: 10.15766/mep_2374-8265.10858 (PMC7050660; doi:10.15766/mep_2374-8265.10858)
Supplement: Supplementary file 1 — A. HER 1.pptx B. HER 2.pptx C. HER 3.pptx D. HER 4.pptx E. HER 5.pptx F. HER 6.pptx G. HER 7.pptx H. Selected HER Handouts.docx I. Case Conference Creation Guide.docx J. Glossary.docx K. Evaluation.docx [file mep-15-10858-s001.zip › E. HER 5.pptx]

## Slide 1
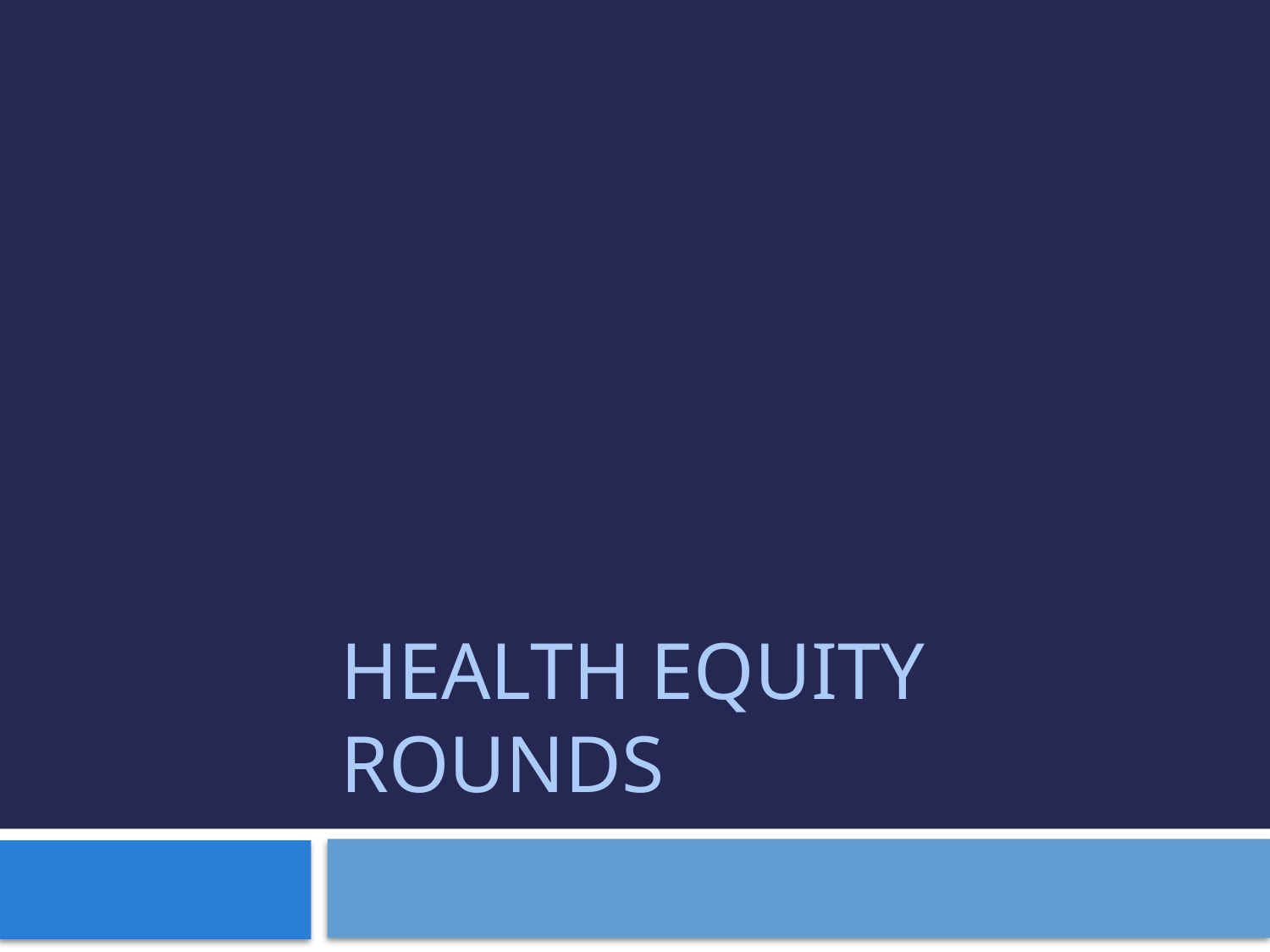

# Health Equity Rounds

## Slide 2
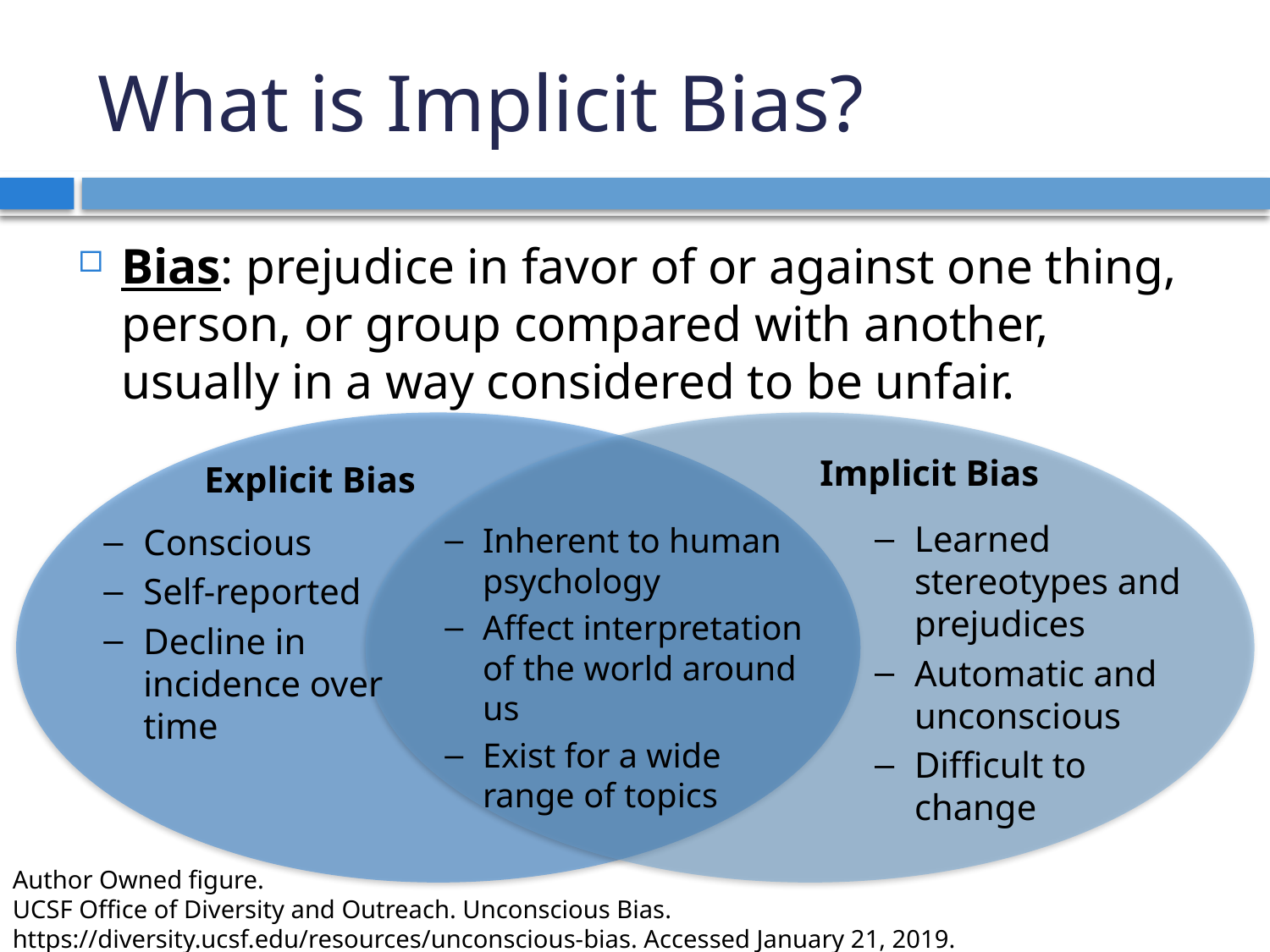

# What is Implicit Bias?
Bias: prejudice in favor of or against one thing, person, or group compared with another, usually in a way considered to be unfair.
Implicit Bias
Explicit Bias
Learned stereotypes and prejudices
Automatic and unconscious
Difficult to change
Inherent to human psychology
Affect interpretation of the world around us
Exist for a wide range of topics
Conscious
Self-reported
Decline in incidence over time
Author Owned figure.
UCSF Office of Diversity and Outreach. Unconscious Bias. https://diversity.ucsf.edu/resources/unconscious-bias. Accessed January 21, 2019.

## Slide 3
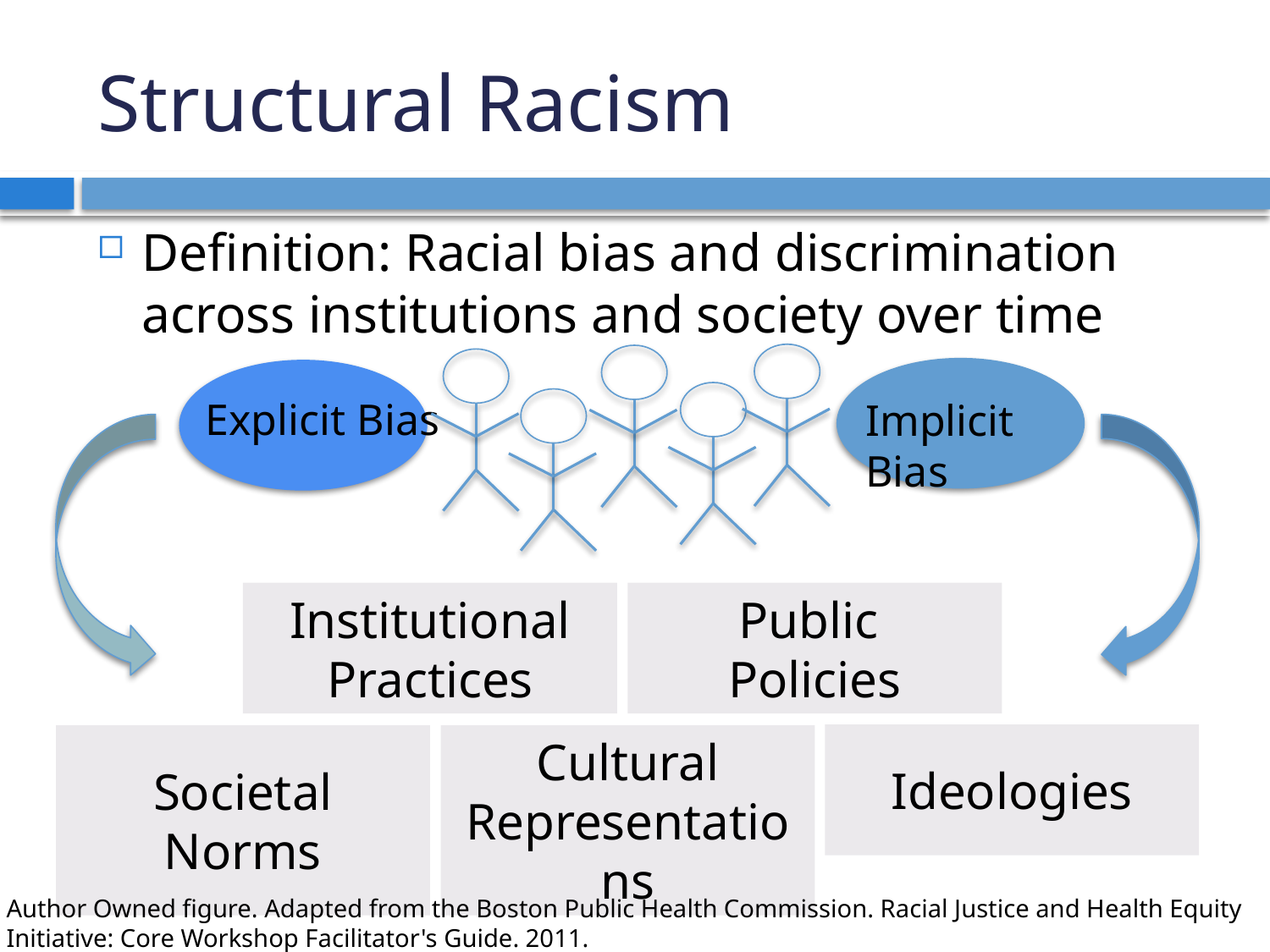

# Structural Racism
Definition: Racial bias and discrimination across institutions and society over time
Explicit Bias
Implicit Bias
Institutional Practices
Public
Policies
Ideologies
Societal Norms
Cultural
Representations
Author Owned figure. Adapted from the Boston Public Health Commission. Racial Justice and Health Equity Initiative: Core Workshop Facilitator's Guide. 2011.

## Slide 4
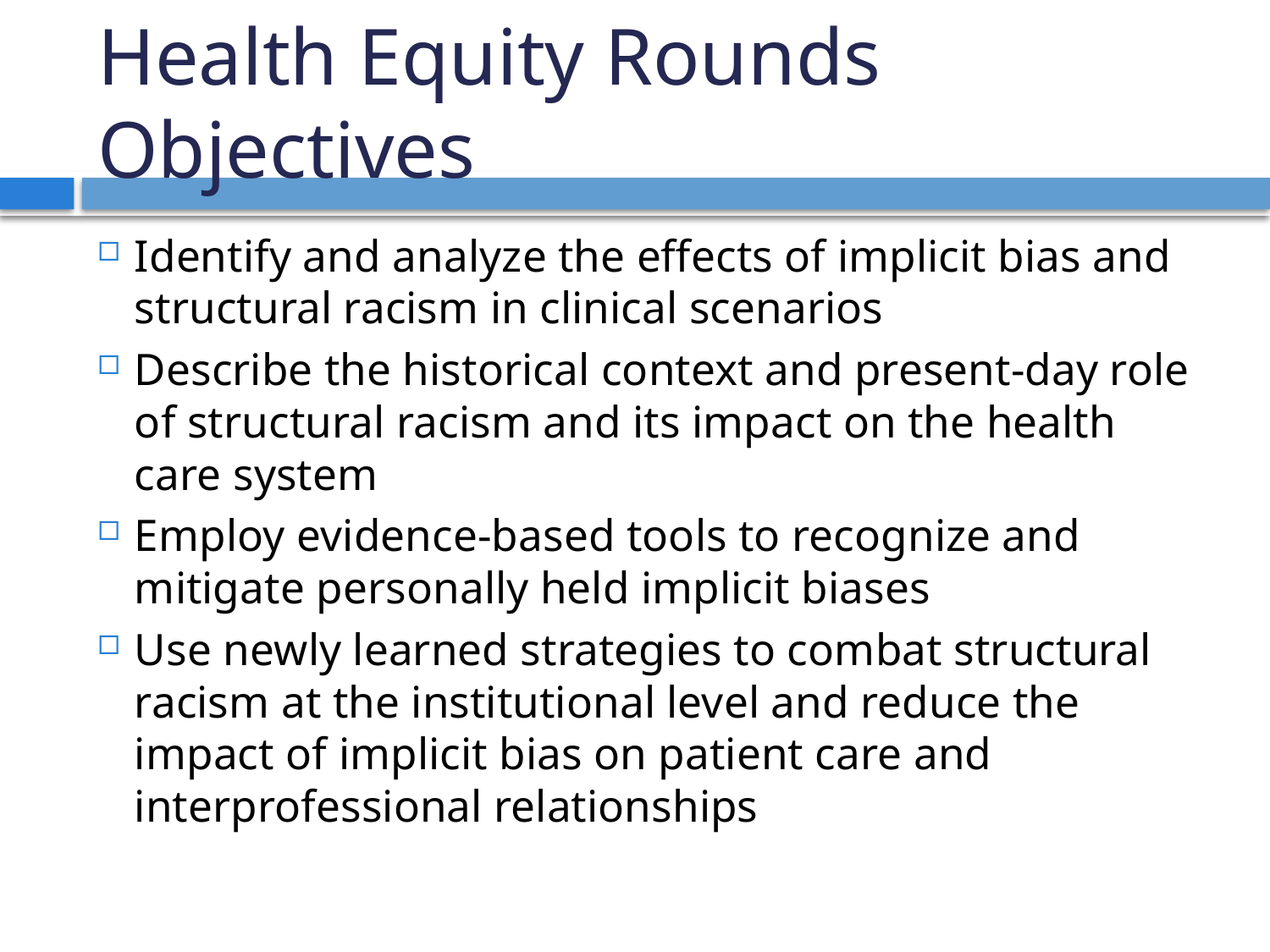

# Health Equity Rounds Objectives
Identify and analyze the effects of implicit bias and structural racism in clinical scenarios
Describe the historical context and present-day role of structural racism and its impact on the health care system
Employ evidence-based tools to recognize and mitigate personally held implicit biases
Use newly learned strategies to combat structural racism at the institutional level and reduce the impact of implicit bias on patient care and interprofessional relationships

## Slide 5
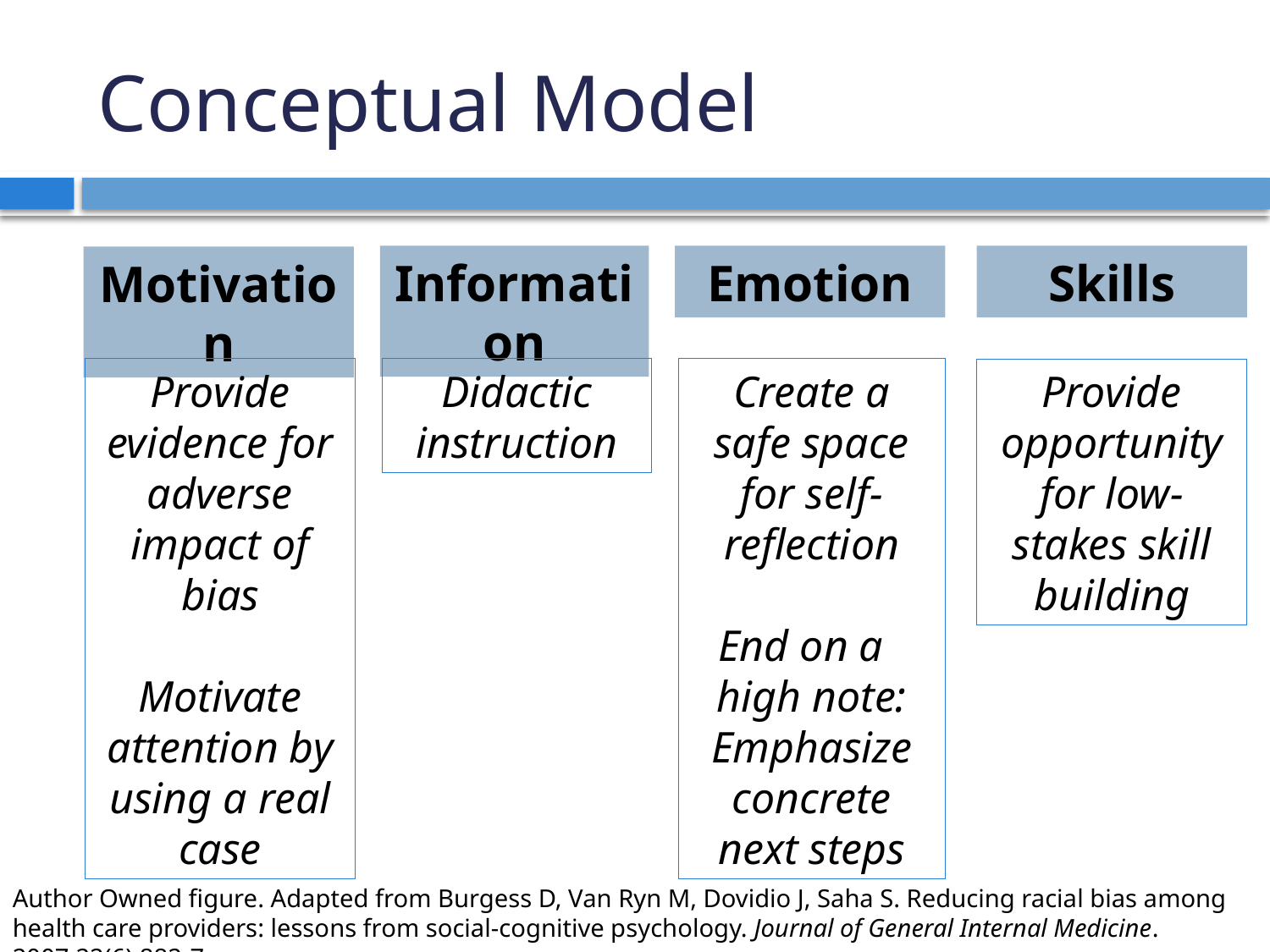

# Conceptual Model
Skills
Information
Emotion
Motivation
Create a safe space for self-reflection
d
End on a high note: Emphasize concrete next steps
Provide evidence for adverse impact of bias
Motivate attention by using a real case
Didactic instruction
Provide opportunity for low-stakes skill building
Author Owned figure. Adapted from Burgess D, Van Ryn M, Dovidio J, Saha S. Reducing racial bias among health care providers: lessons from social-cognitive psychology. Journal of General Internal Medicine. 2007;22(6):882-7.

## Slide 6
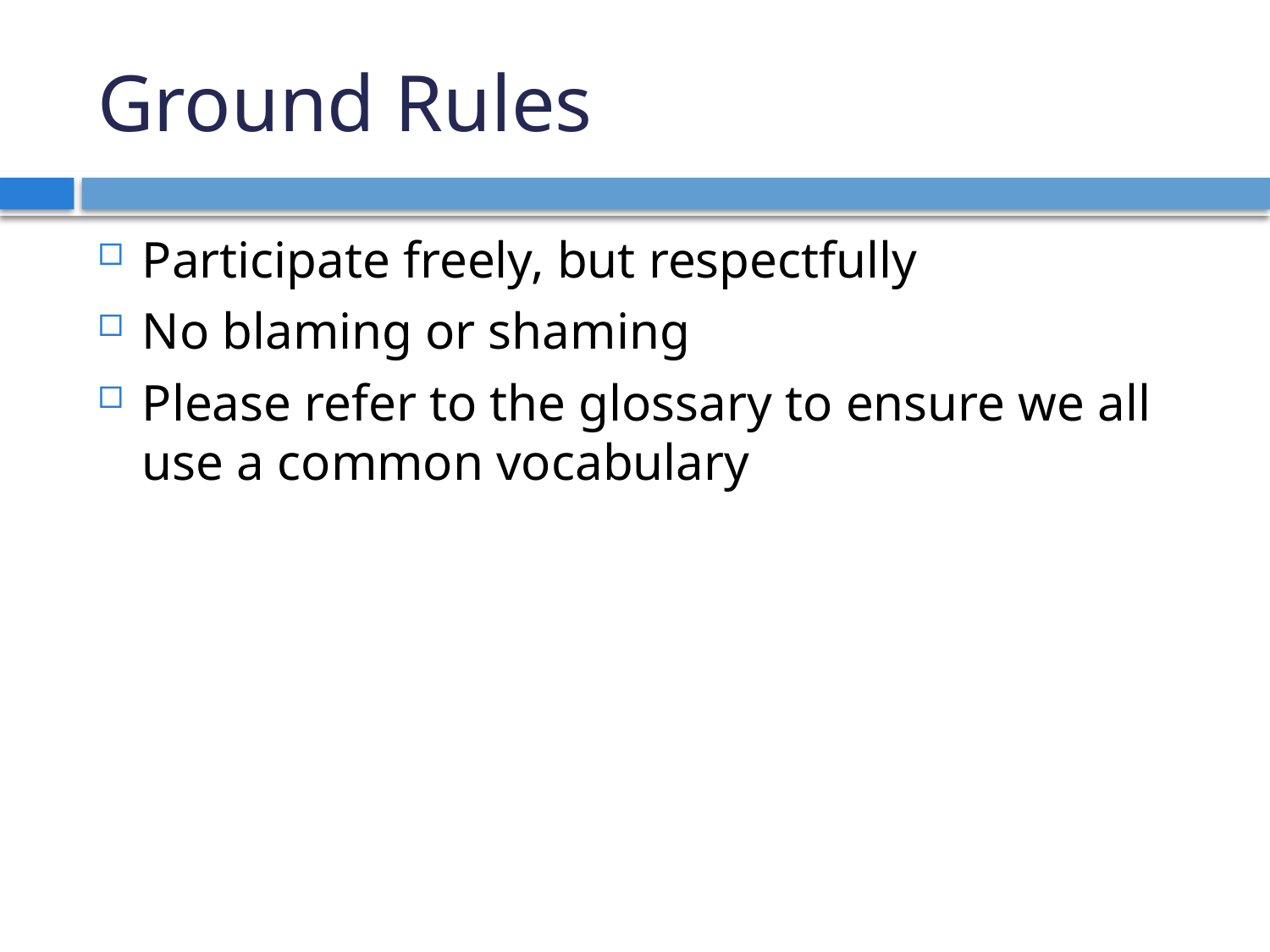

# Ground Rules
Participate freely, but respectfully
No blaming or shaming
Please refer to the glossary to ensure we all use a common vocabulary

## Slide 7
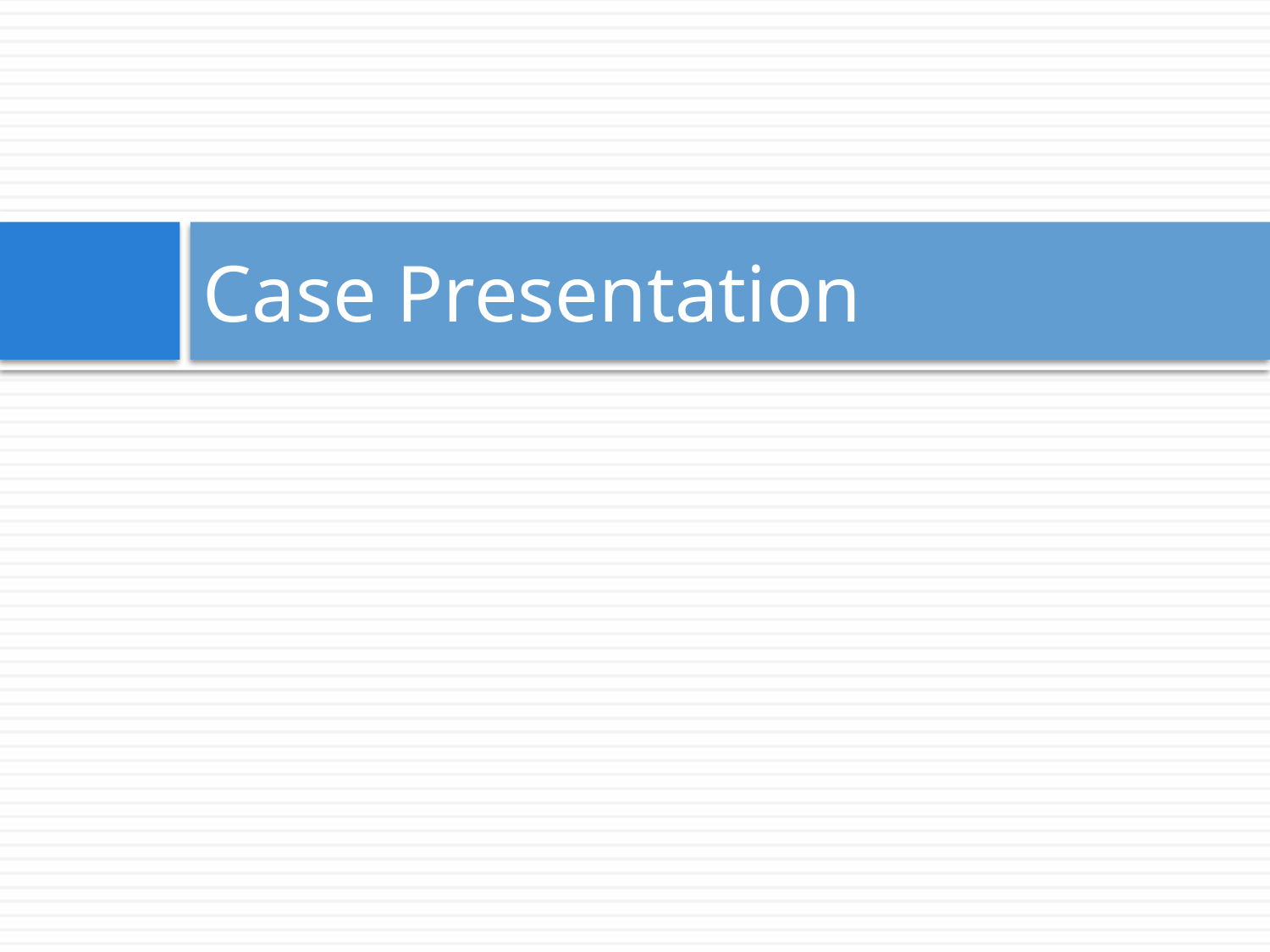

# Case Presentation

## Slide 8
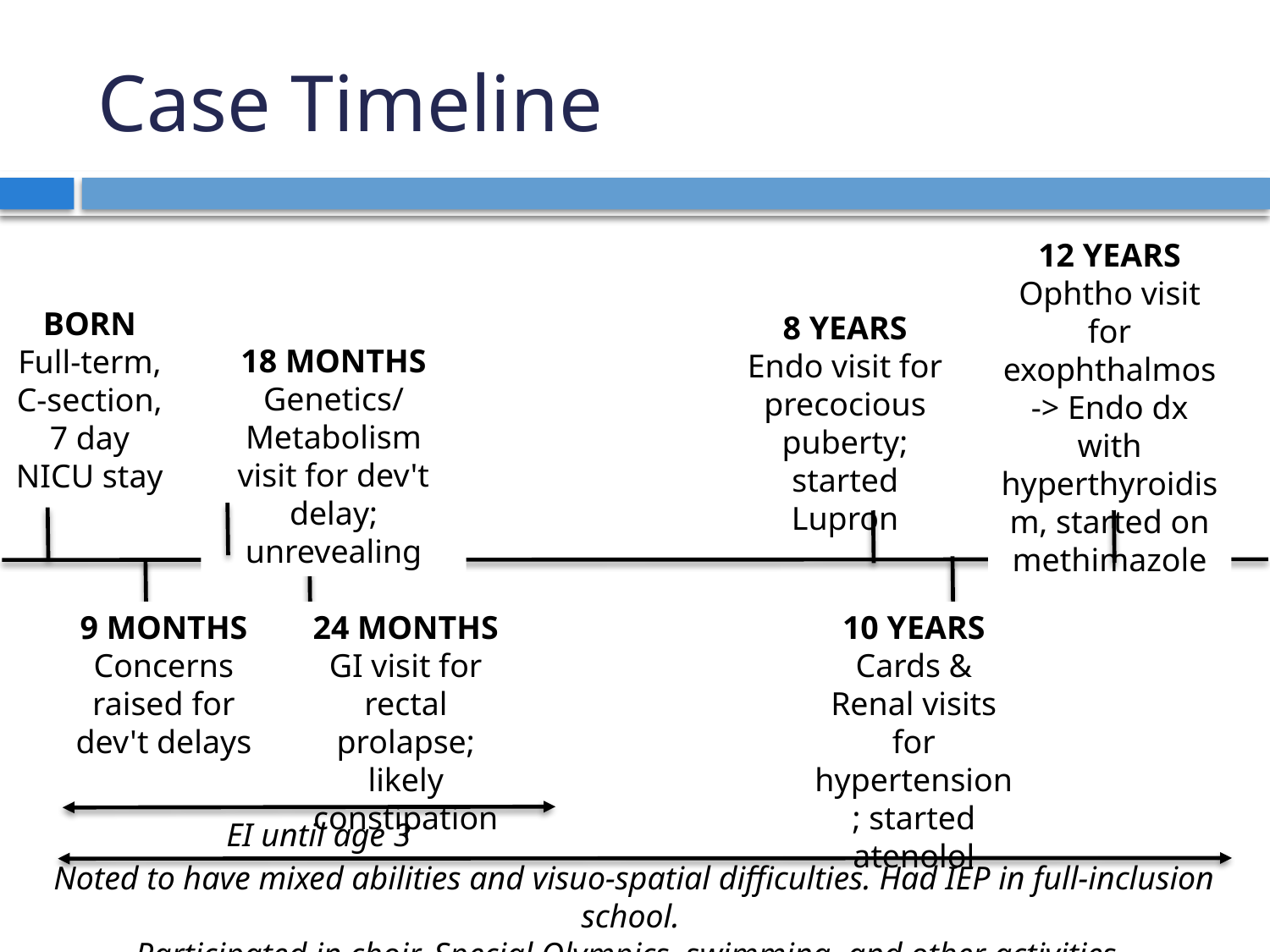

# Case Timeline
12 YEARS
Ophtho visit for exophthalmos -> Endo dx with hyperthyroidism, started on methimazole
BORN
Full-term, C-section, 7 day NICU stay
8 YEARS
Endo visit for precocious puberty; started Lupron
18 MONTHS
Genetics/Metabolism visit for dev't delay; unrevealing
10 YEARS
Cards & Renal visits for hypertension; started atenolol
24 MONTHS
GI visit for rectal prolapse; likely constipation
9 MONTHS
Concerns raised for dev't delays
EI until age 3
Noted to have mixed abilities and visuo-spatial difficulties. Had IEP in full-inclusion school.
Participated in choir, Special Olympics, swimming, and other activities.

## Slide 9
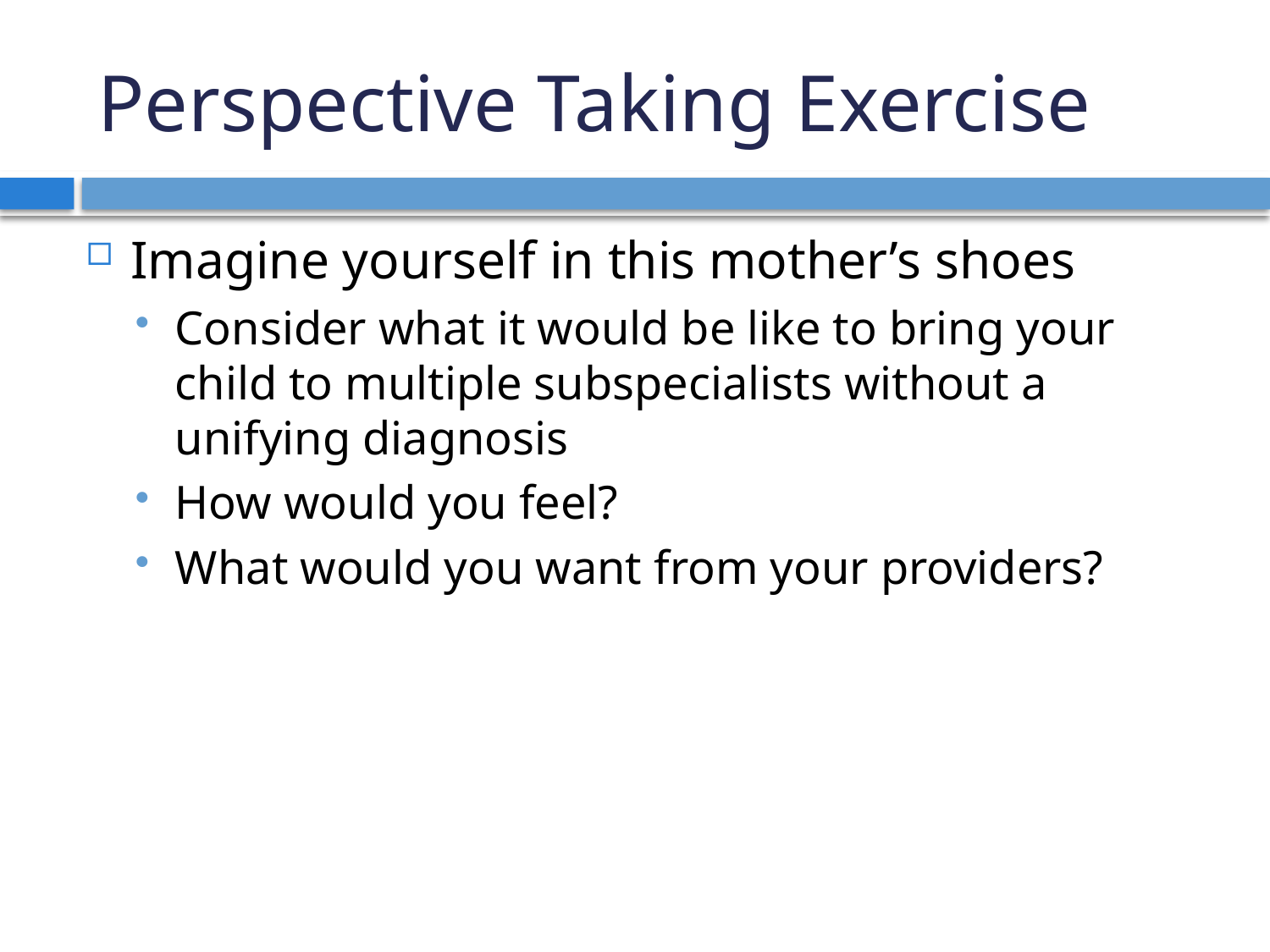

# Perspective Taking Exercise
Imagine yourself in this mother’s shoes
Consider what it would be like to bring your child to multiple subspecialists without a unifying diagnosis
How would you feel?
What would you want from your providers?

## Slide 10
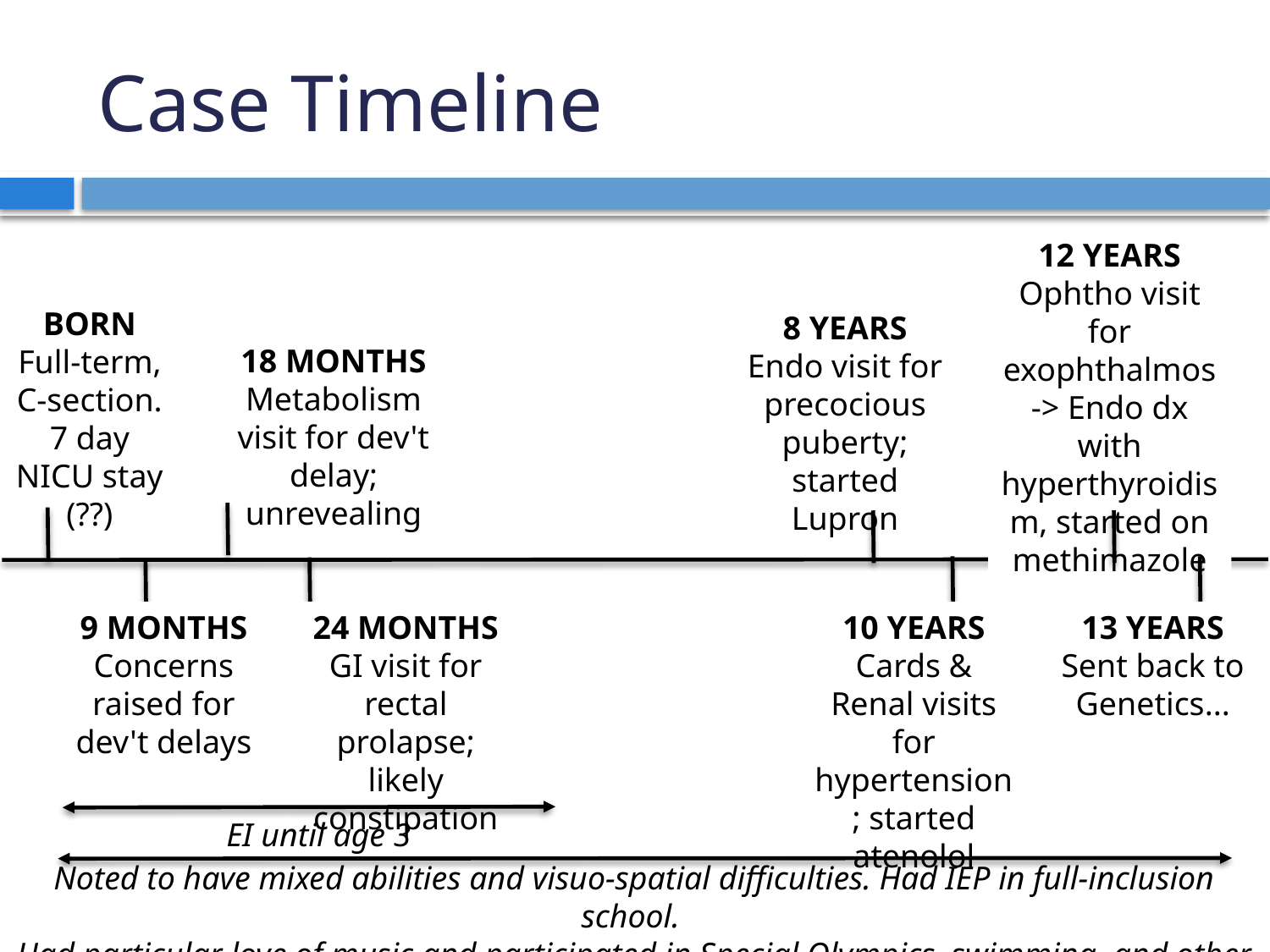

# Case Timeline
12 YEARS
Ophtho visit for exophthalmos -> Endo dx with hyperthyroidism, started on methimazole
BORN
Full-term, C-section. 7 day NICU stay (??)
8 YEARS
Endo visit for precocious puberty; started Lupron
18 MONTHS
Metabolism visit for dev't delay; unrevealing
13 YEARS
Sent back to Genetics...
9 MONTHS
Concerns raised for dev't delays
24 MONTHS
GI visit for rectal prolapse; likely constipation
10 YEARS
Cards & Renal visits for hypertension; started atenolol
EI until age 3
Noted to have mixed abilities and visuo-spatial difficulties. Had IEP in full-inclusion school.
Had particular love of music and participated in Special Olympics, swimming, and other activities.

## Slide 11
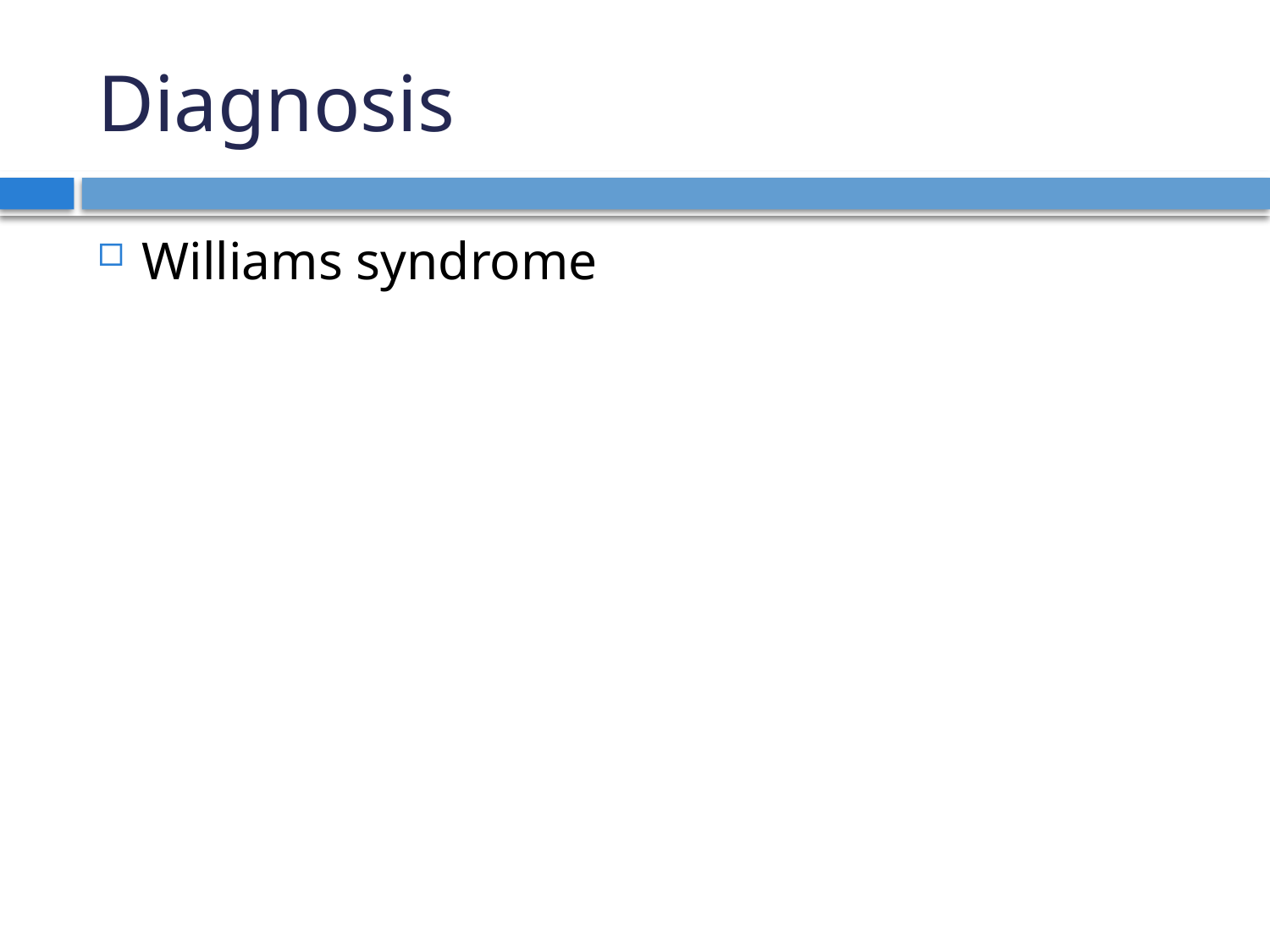

# Diagnosis
Williams syndrome

## Slide 12
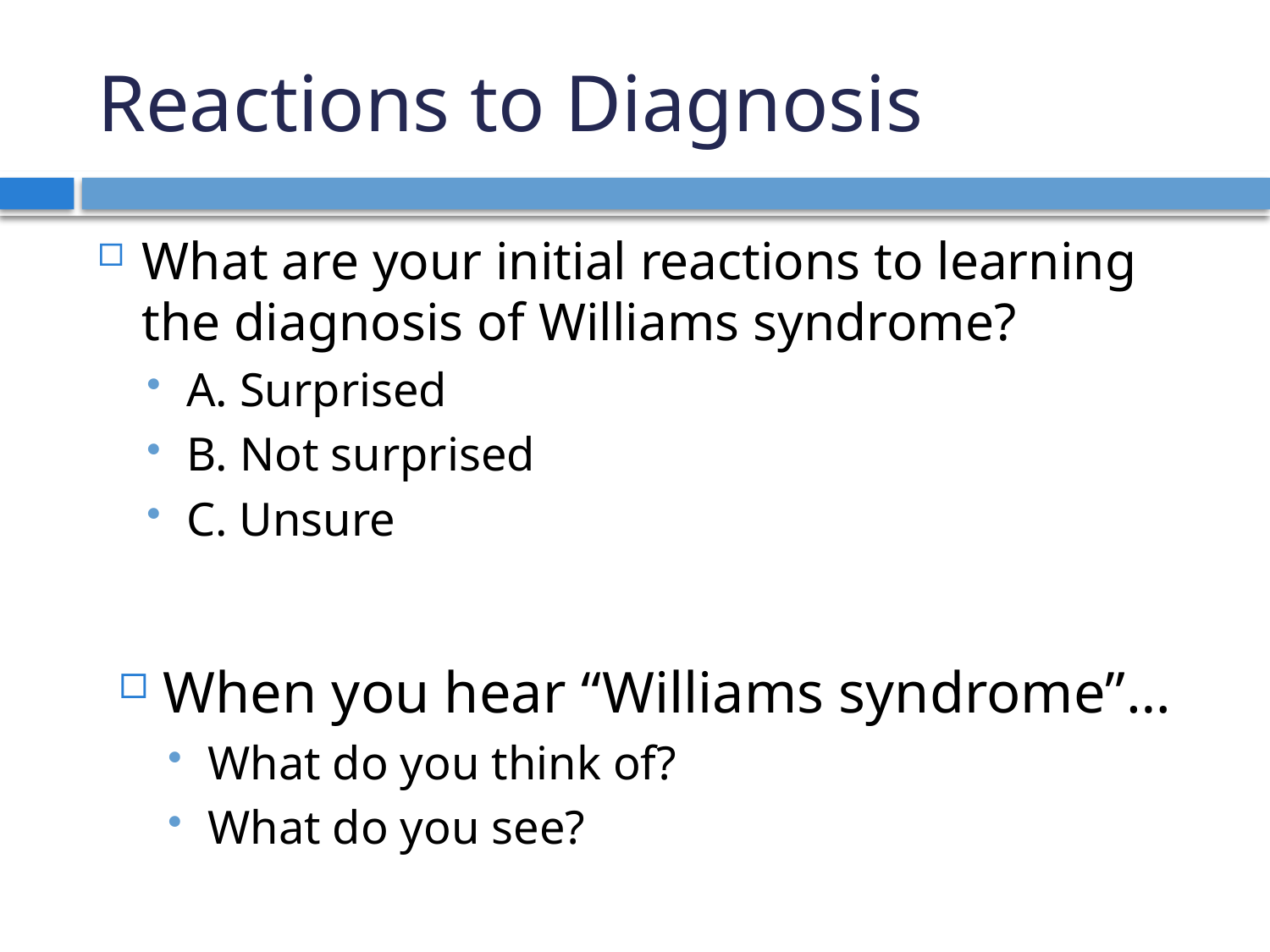

# Reactions to Diagnosis
What are your initial reactions to learning the diagnosis of Williams syndrome?
A. Surprised
B. Not surprised
C. Unsure
When you hear “Williams syndrome”…
What do you think of?
What do you see?

## Slide 13
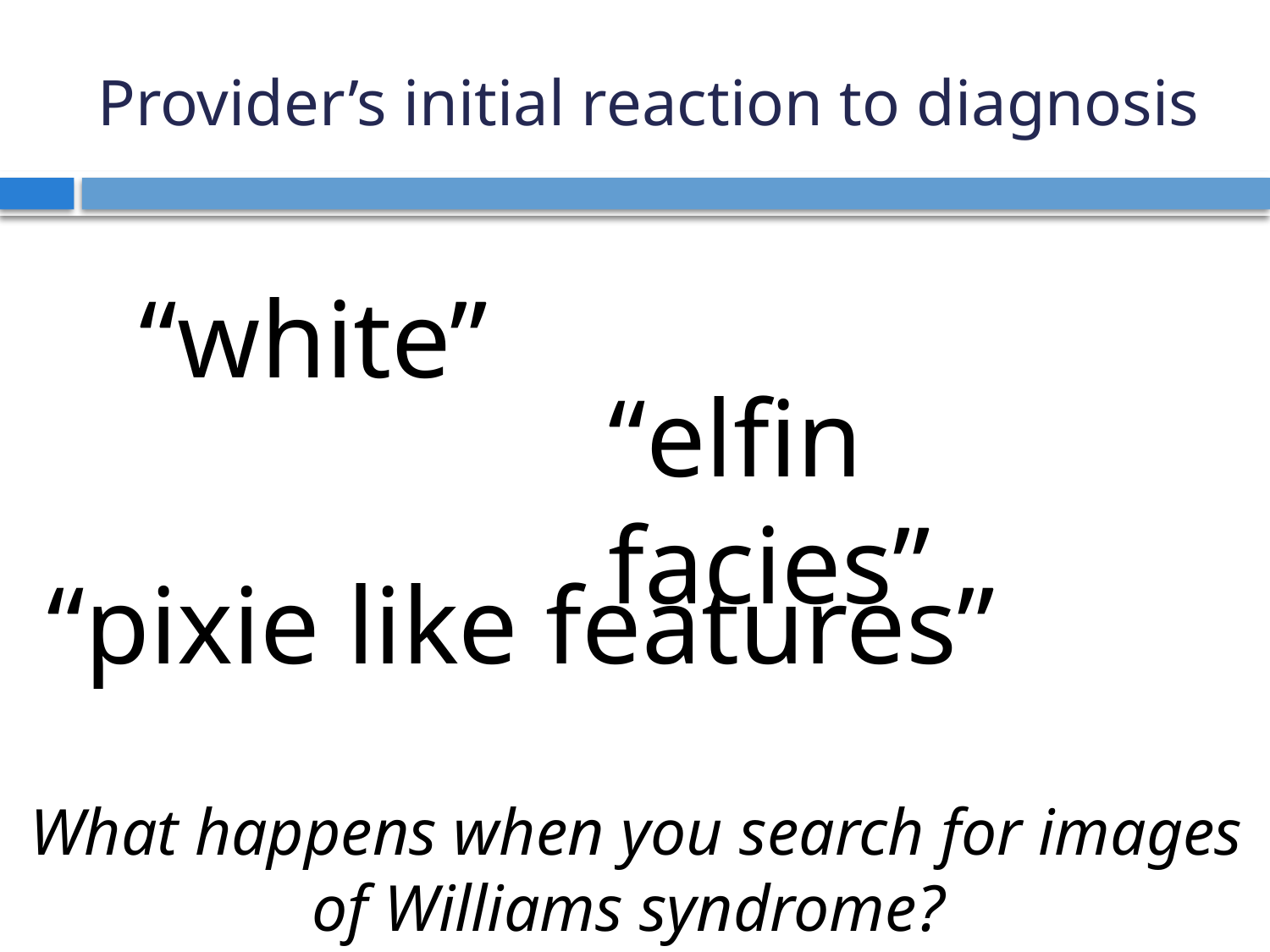

# Provider’s initial reaction to diagnosis
“white”
“elfin facies”
“pixie like features”
What happens when you search for images of Williams syndrome?

## Slide 14
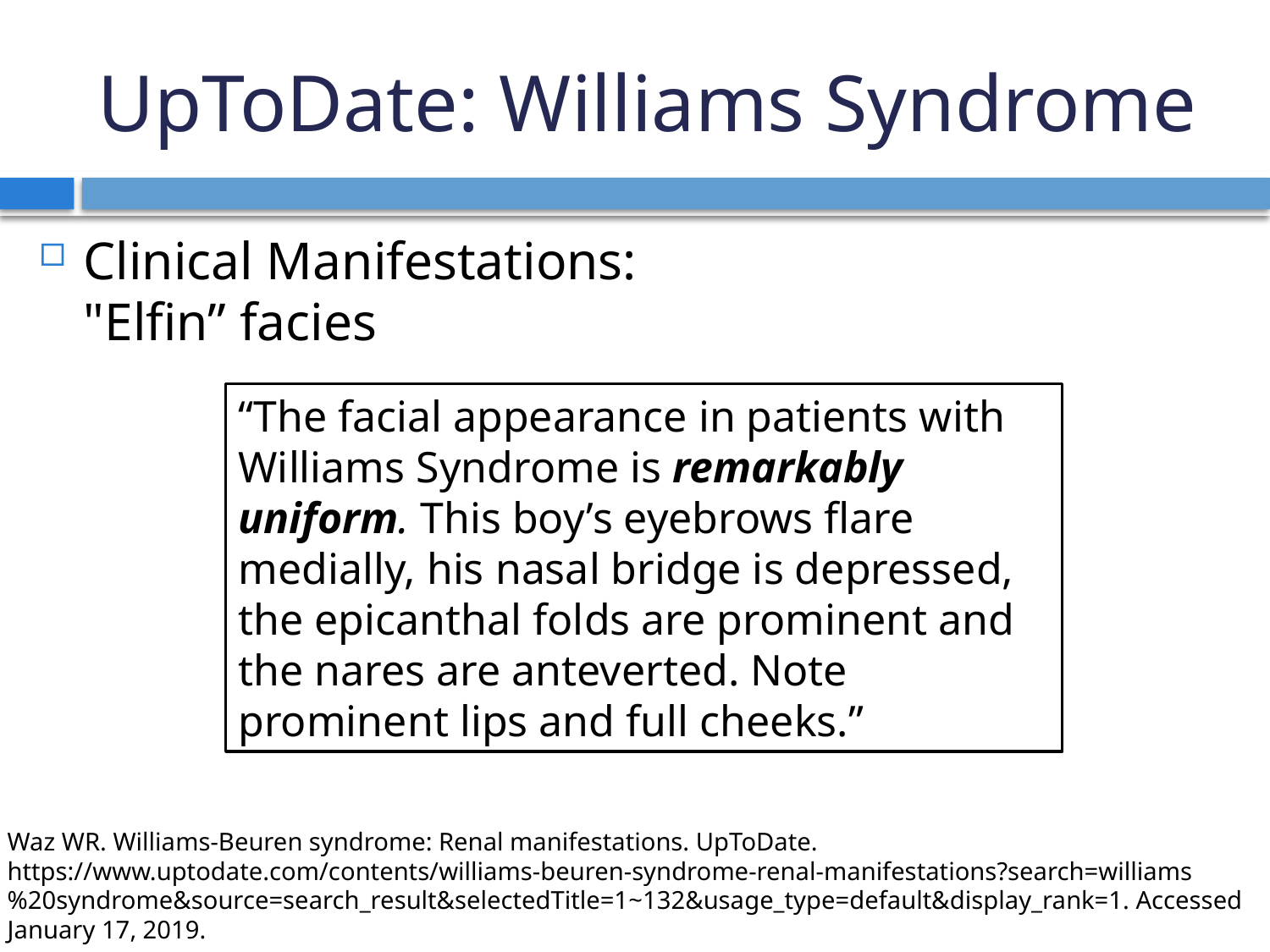

# UpToDate: Williams Syndrome
Clinical Manifestations: "Elfin” facies
“The facial appearance in patients with Williams Syndrome is remarkably uniform. This boy’s eyebrows flare medially, his nasal bridge is depressed, the epicanthal folds are prominent and the nares are anteverted. Note prominent lips and full cheeks.”
Waz WR. Williams-Beuren syndrome: Renal manifestations. UpToDate. https://www.uptodate.com/contents/williams-beuren-syndrome-renal-manifestations?search=williams%20syndrome&source=search_result&selectedTitle=1~132&usage_type=default&display_rank=1. Accessed January 17, 2019.

## Slide 15
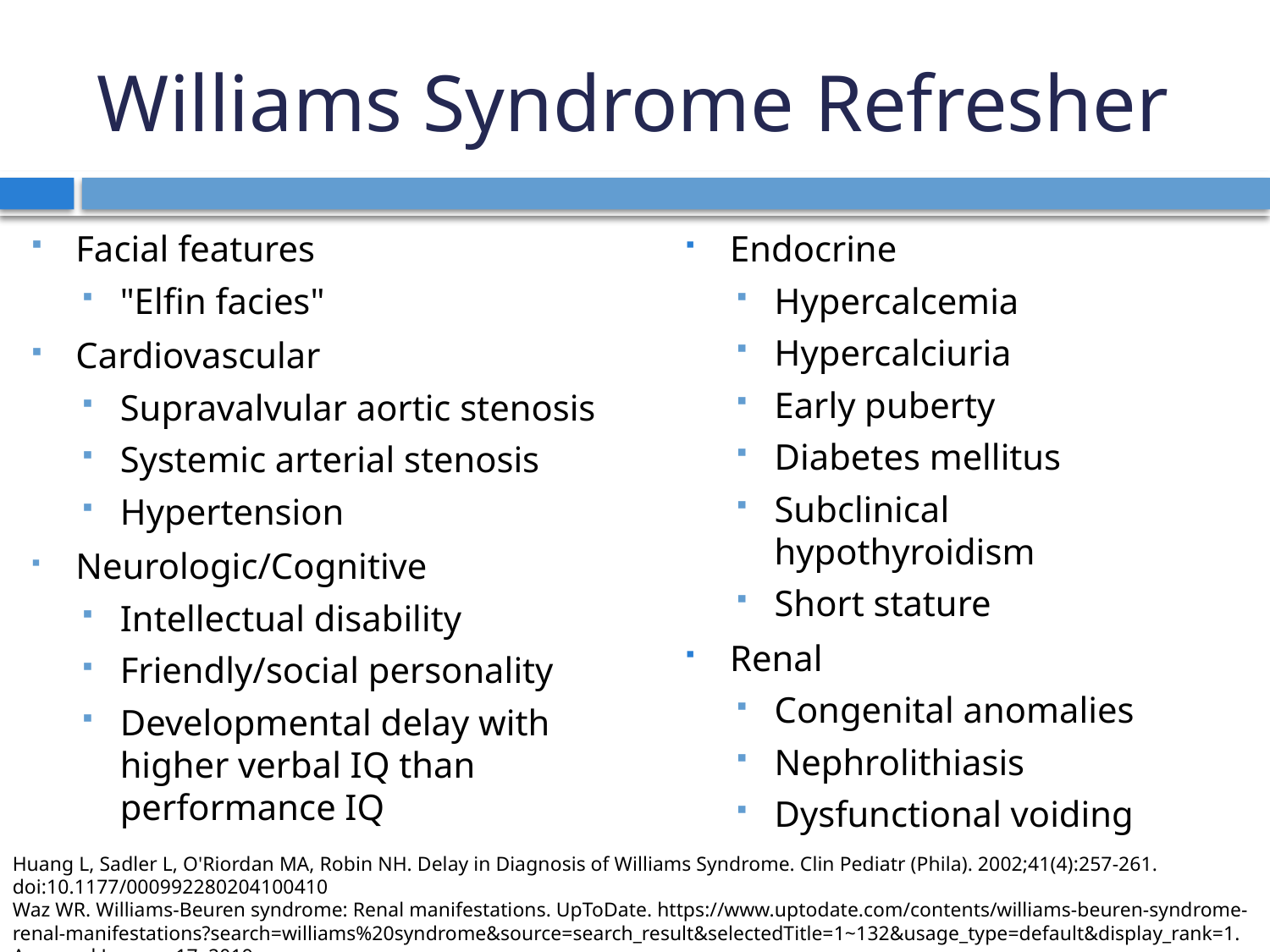

# Williams Syndrome Refresher
Facial features
"Elfin facies"
Cardiovascular
Supravalvular aortic stenosis
Systemic arterial stenosis
Hypertension
Neurologic/Cognitive
Intellectual disability
Friendly/social personality
Developmental delay with higher verbal IQ than performance IQ
Endocrine
Hypercalcemia
Hypercalciuria
Early puberty
Diabetes mellitus
Subclinical hypothyroidism
Short stature
Renal
Congenital anomalies
Nephrolithiasis
Dysfunctional voiding
Huang L, Sadler L, O'Riordan MA, Robin NH. Delay in Diagnosis of Williams Syndrome. Clin Pediatr (Phila). 2002;41(4):257-261. doi:10.1177/000992280204100410
Waz WR. Williams-Beuren syndrome: Renal manifestations. UpToDate. https://www.uptodate.com/contents/williams-beuren-syndrome-renal-manifestations?search=williams%20syndrome&source=search_result&selectedTitle=1~132&usage_type=default&display_rank=1. Accessed January 17, 2019.

## Slide 16
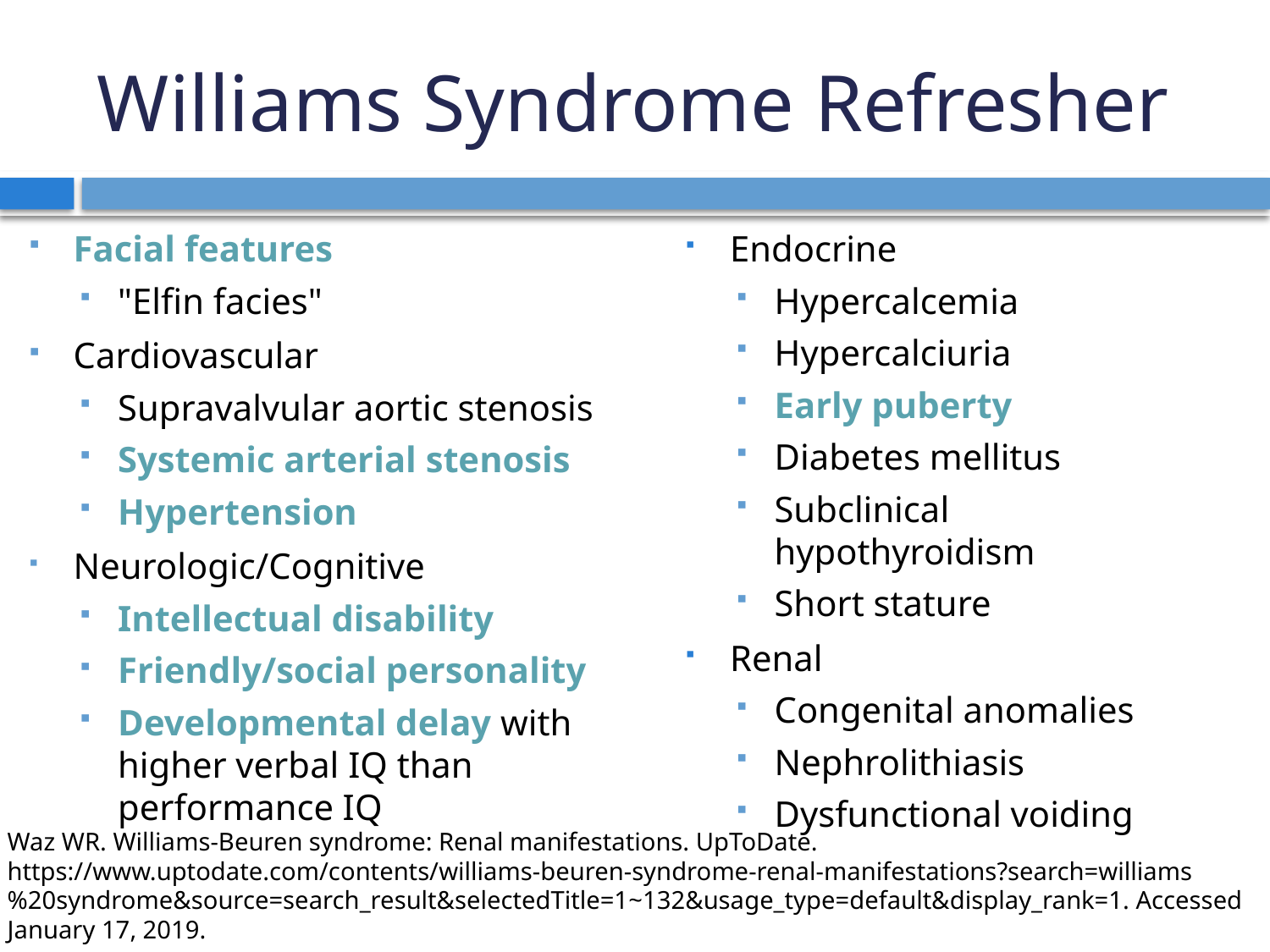

# Williams Syndrome Refresher
Facial features
"Elfin facies"
Cardiovascular
Supravalvular aortic stenosis
Systemic arterial stenosis
Hypertension
Neurologic/Cognitive
Intellectual disability
Friendly/social personality
Developmental delay with higher verbal IQ than performance IQ
Endocrine
Hypercalcemia
Hypercalciuria
Early puberty
Diabetes mellitus
Subclinical hypothyroidism
Short stature
Renal
Congenital anomalies
Nephrolithiasis
Dysfunctional voiding
Waz WR. Williams-Beuren syndrome: Renal manifestations. UpToDate. https://www.uptodate.com/contents/williams-beuren-syndrome-renal-manifestations?search=williams%20syndrome&source=search_result&selectedTitle=1~132&usage_type=default&display_rank=1. Accessed January 17, 2019.

## Slide 17
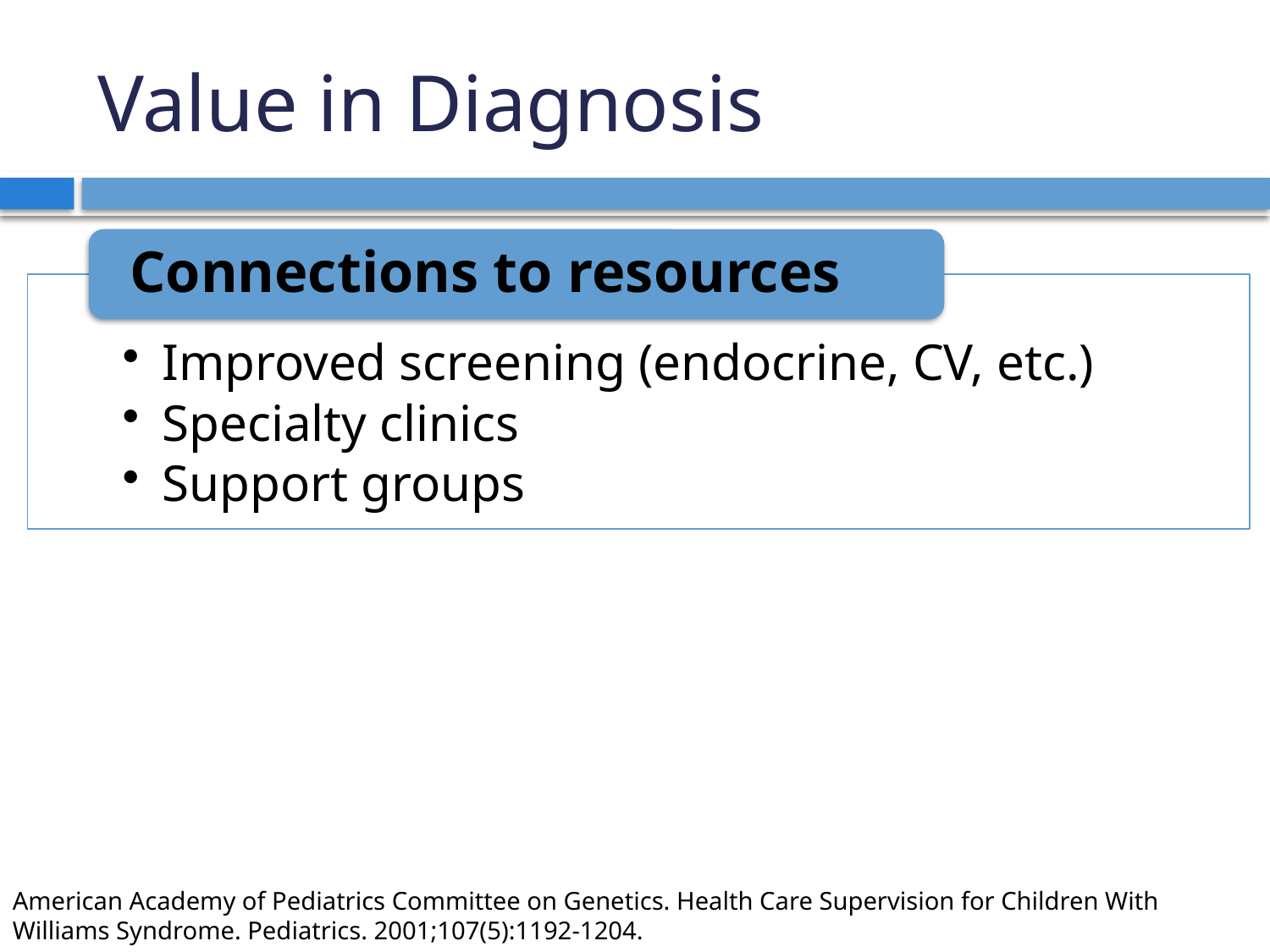

# Value in Diagnosis
American Academy of Pediatrics Committee on Genetics. Health Care Supervision for Children With Williams Syndrome. Pediatrics. 2001;107(5):1192-1204.

## Slide 18
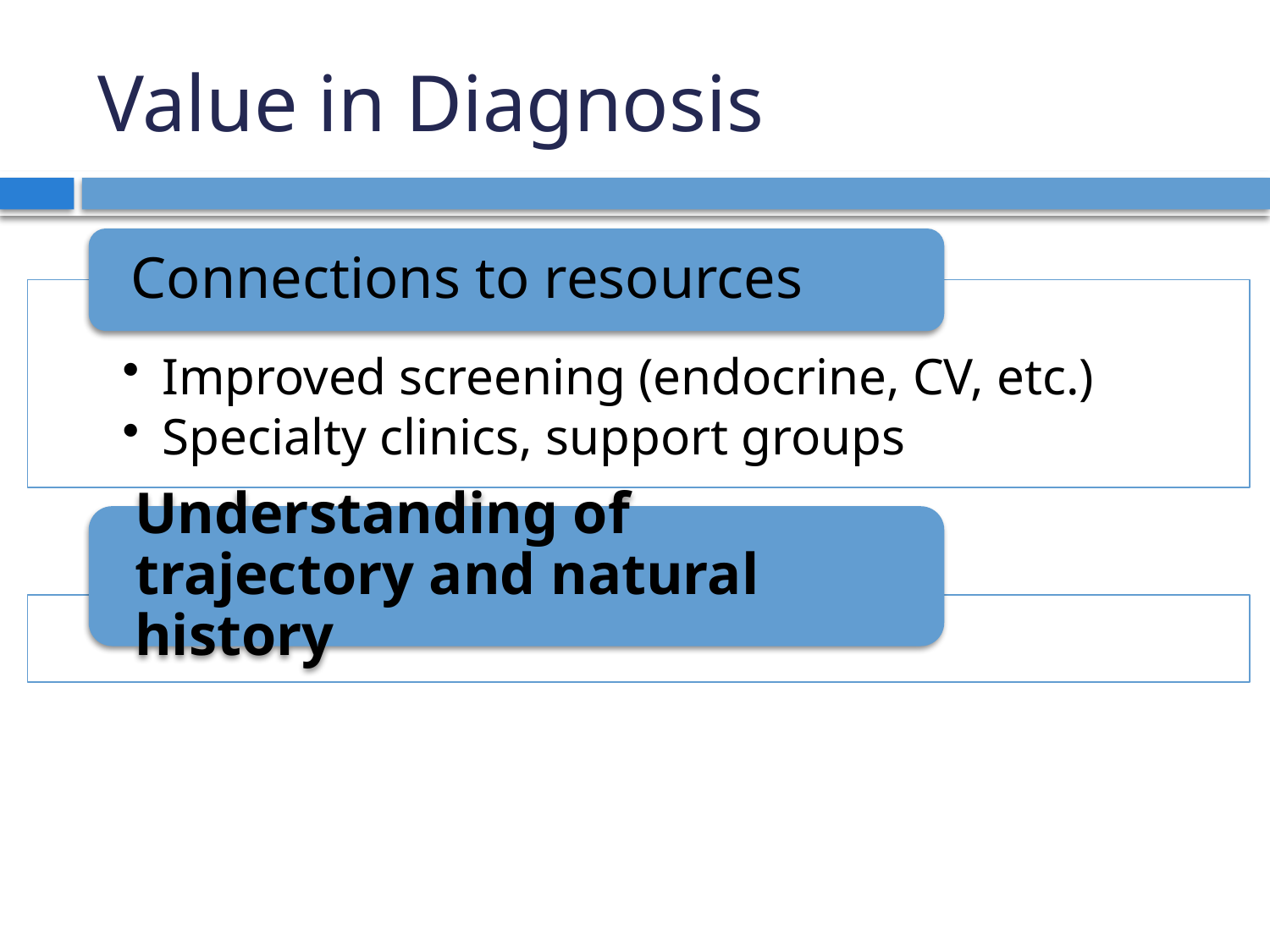

# Value in Diagnosis

## Slide 19
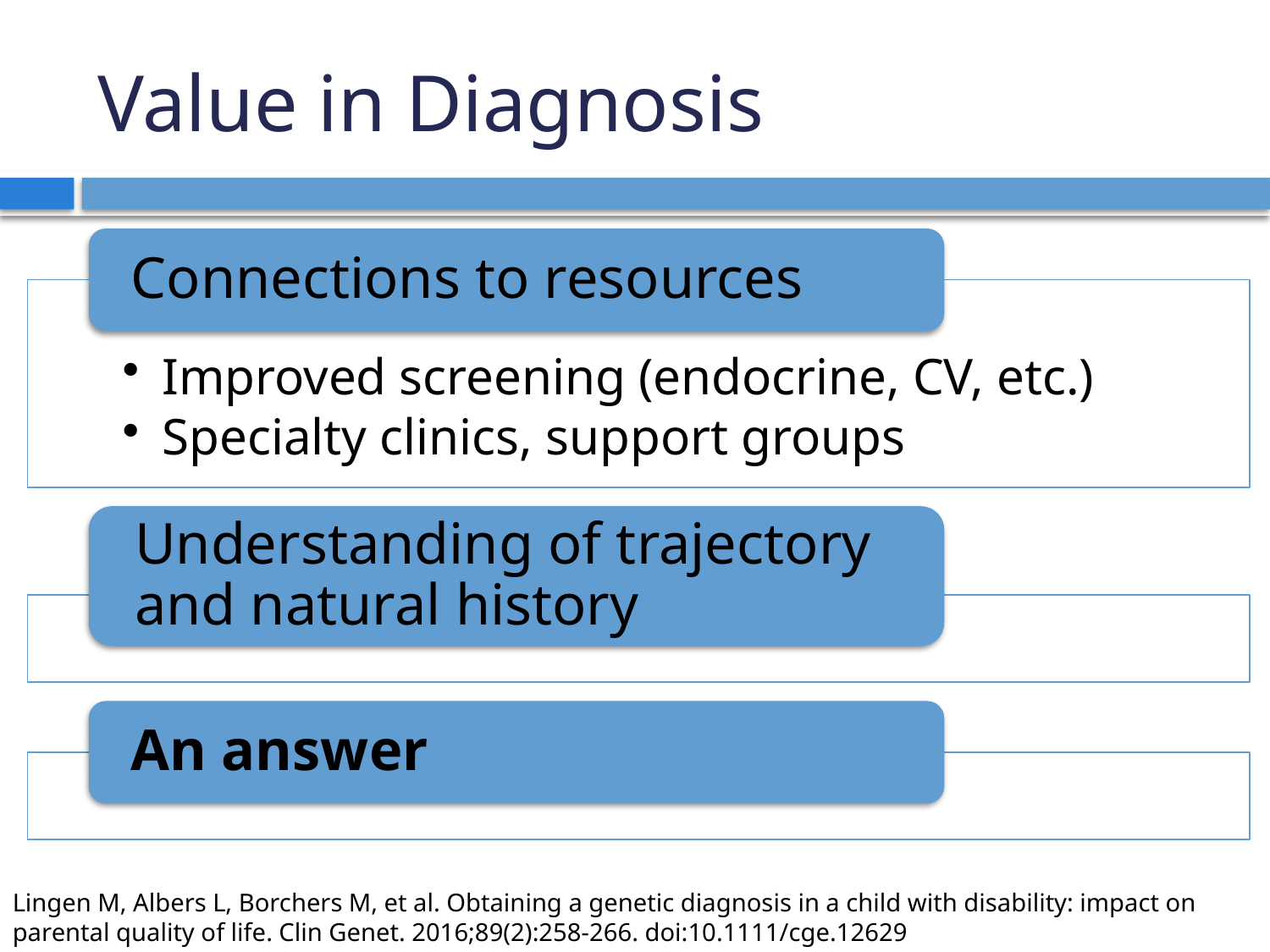

# Value in Diagnosis
Lingen M, Albers L, Borchers M, et al. Obtaining a genetic diagnosis in a child with disability: impact on parental quality of life. Clin Genet. 2016;89(2):258-266. doi:10.1111/cge.12629

## Slide 20
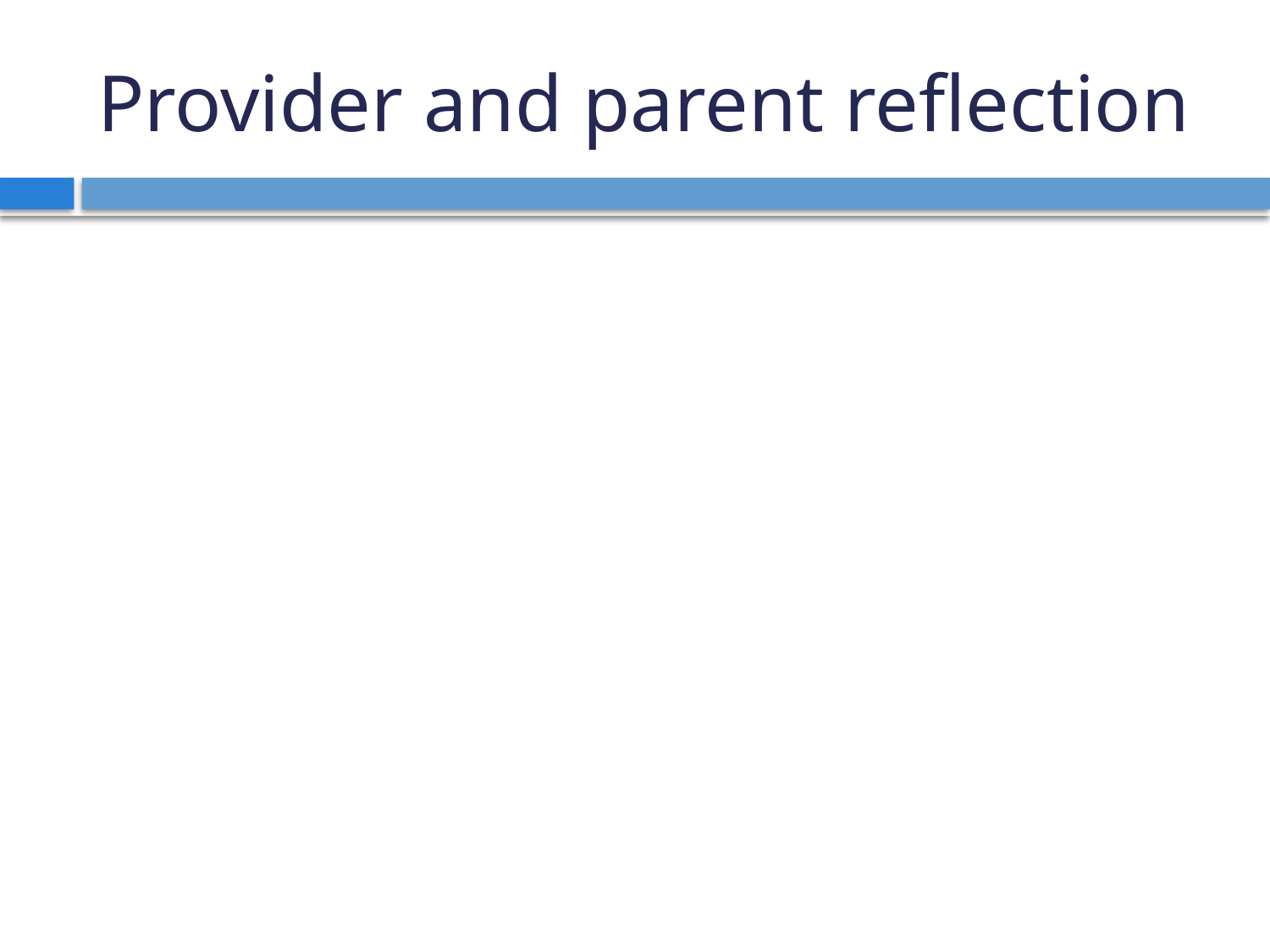

# Provider and parent reflection

## Slide 21
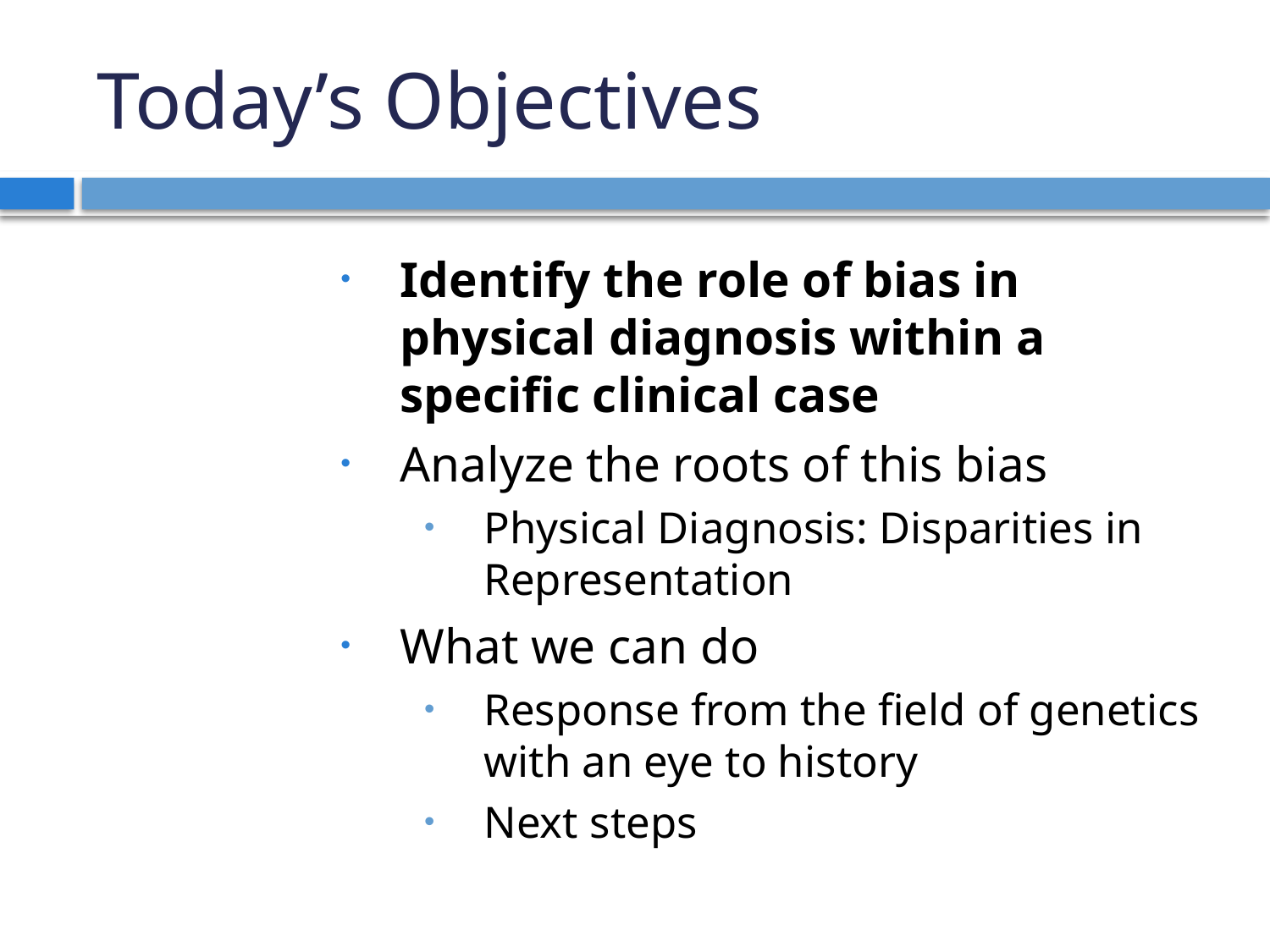

# Today’s Objectives
Identify the role of bias in physical diagnosis within a specific clinical case
Analyze the roots of this bias
Physical Diagnosis: Disparities in Representation
What we can do
Response from the field of genetics with an eye to history
Next steps

## Slide 22
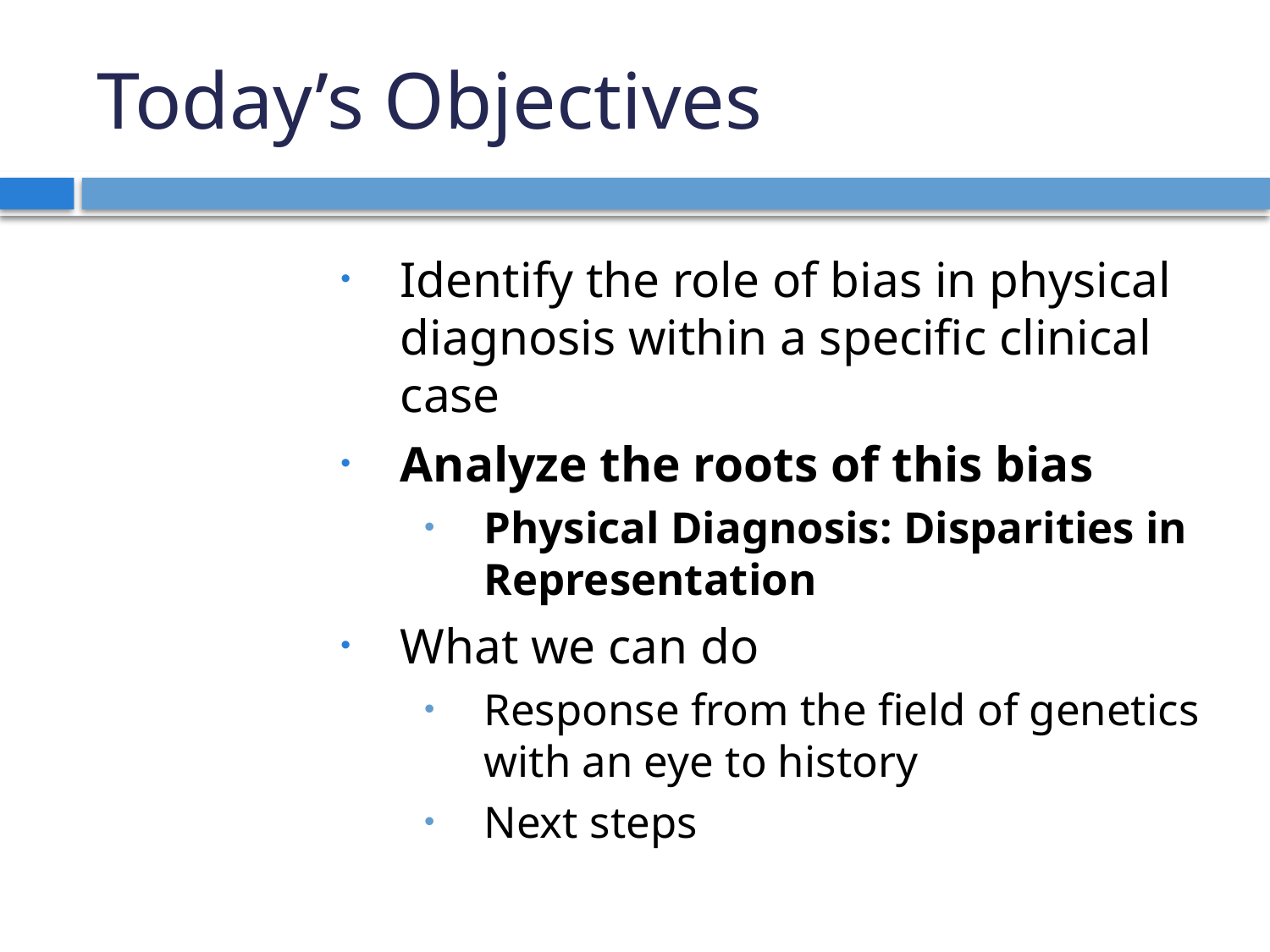

# Today’s Objectives
Identify the role of bias in physical diagnosis within a specific clinical case
Analyze the roots of this bias
Physical Diagnosis: Disparities in Representation
What we can do
Response from the field of genetics with an eye to history
Next steps

## Slide 23
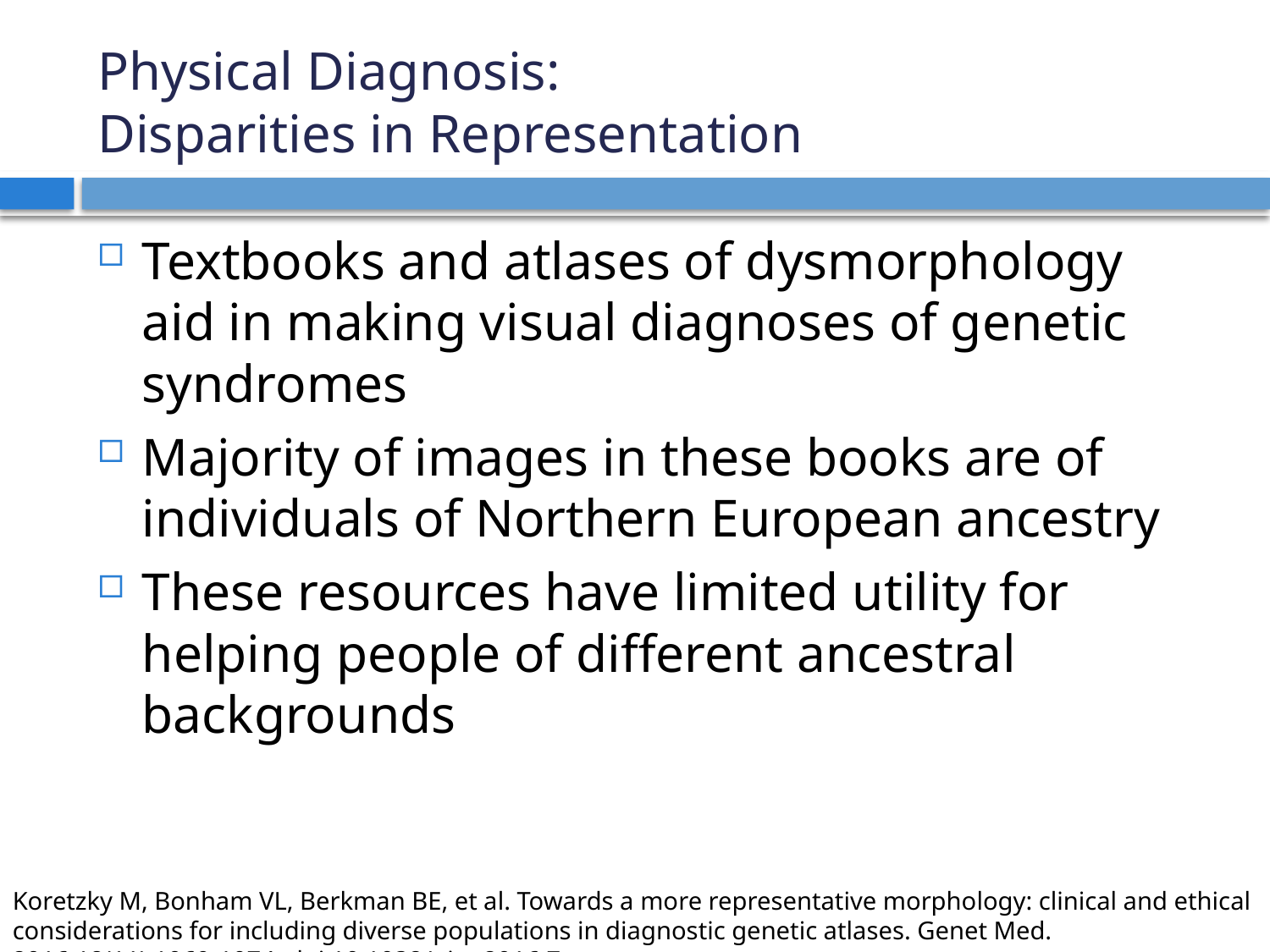

# Physical Diagnosis: Disparities in Representation
Textbooks and atlases of dysmorphology aid in making visual diagnoses of genetic syndromes
Majority of images in these books are of individuals of Northern European ancestry
These resources have limited utility for helping people of different ancestral backgrounds
Koretzky M, Bonham VL, Berkman BE, et al. Towards a more representative morphology: clinical and ethical considerations for including diverse populations in diagnostic genetic atlases. Genet Med. 2016;18(11):1069-1074. doi:10.1038/gim.2016.7

## Slide 24
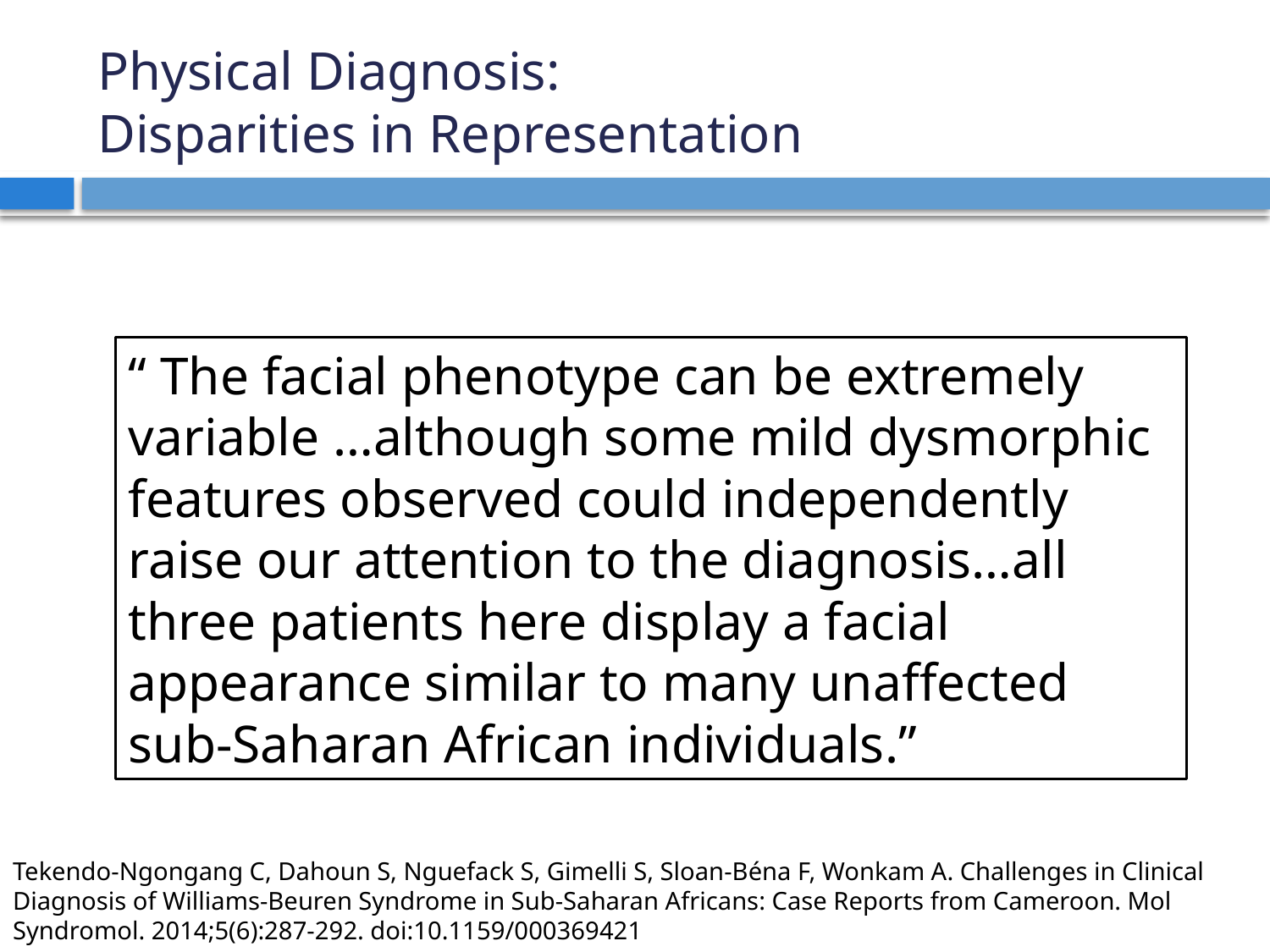

# Physical Diagnosis: Disparities in Representation
“ The facial phenotype can be extremely variable …although some mild dysmorphic features observed could independently raise our attention to the diagnosis…all three patients here display a facial appearance similar to many unaffected sub-Saharan African individuals.”
Tekendo-Ngongang C, Dahoun S, Nguefack S, Gimelli S, Sloan-Béna F, Wonkam A. Challenges in Clinical Diagnosis of Williams-Beuren Syndrome in Sub-Saharan Africans: Case Reports from Cameroon. Mol Syndromol. 2014;5(6):287-292. doi:10.1159/000369421

## Slide 25
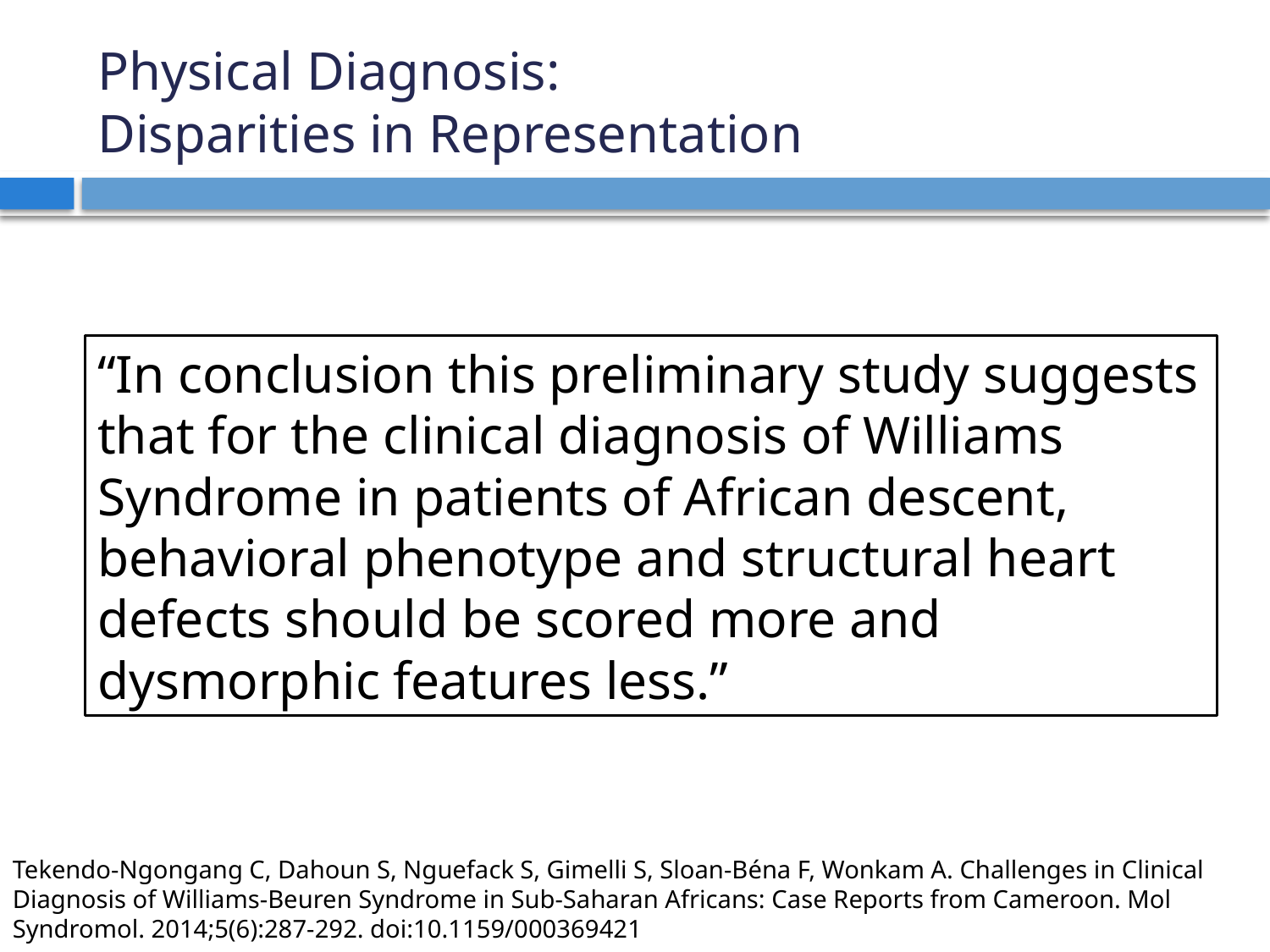

# Physical Diagnosis: Disparities in Representation
“In conclusion this preliminary study suggests that for the clinical diagnosis of Williams Syndrome in patients of African descent, behavioral phenotype and structural heart defects should be scored more and dysmorphic features less.”
Tekendo-Ngongang C, Dahoun S, Nguefack S, Gimelli S, Sloan-Béna F, Wonkam A. Challenges in Clinical Diagnosis of Williams-Beuren Syndrome in Sub-Saharan Africans: Case Reports from Cameroon. Mol Syndromol. 2014;5(6):287-292. doi:10.1159/000369421

## Slide 26
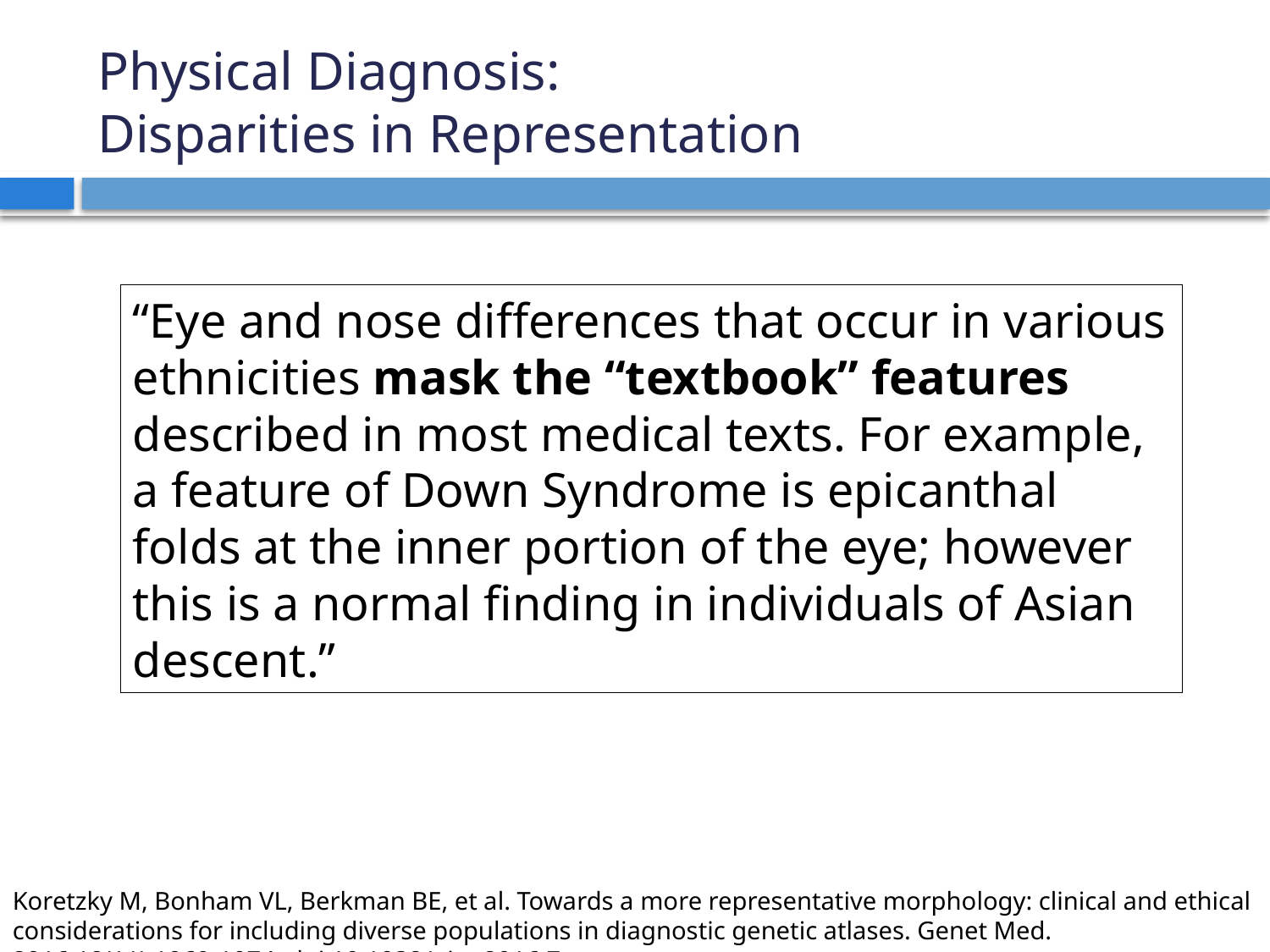

# Physical Diagnosis: Disparities in Representation
“Eye and nose differences that occur in various ethnicities mask the “textbook” features described in most medical texts. For example, a feature of Down Syndrome is epicanthal folds at the inner portion of the eye; however this is a normal finding in individuals of Asian descent.”
Koretzky M, Bonham VL, Berkman BE, et al. Towards a more representative morphology: clinical and ethical considerations for including diverse populations in diagnostic genetic atlases. Genet Med. 2016;18(11):1069-1074. doi:10.1038/gim.2016.7

## Slide 27
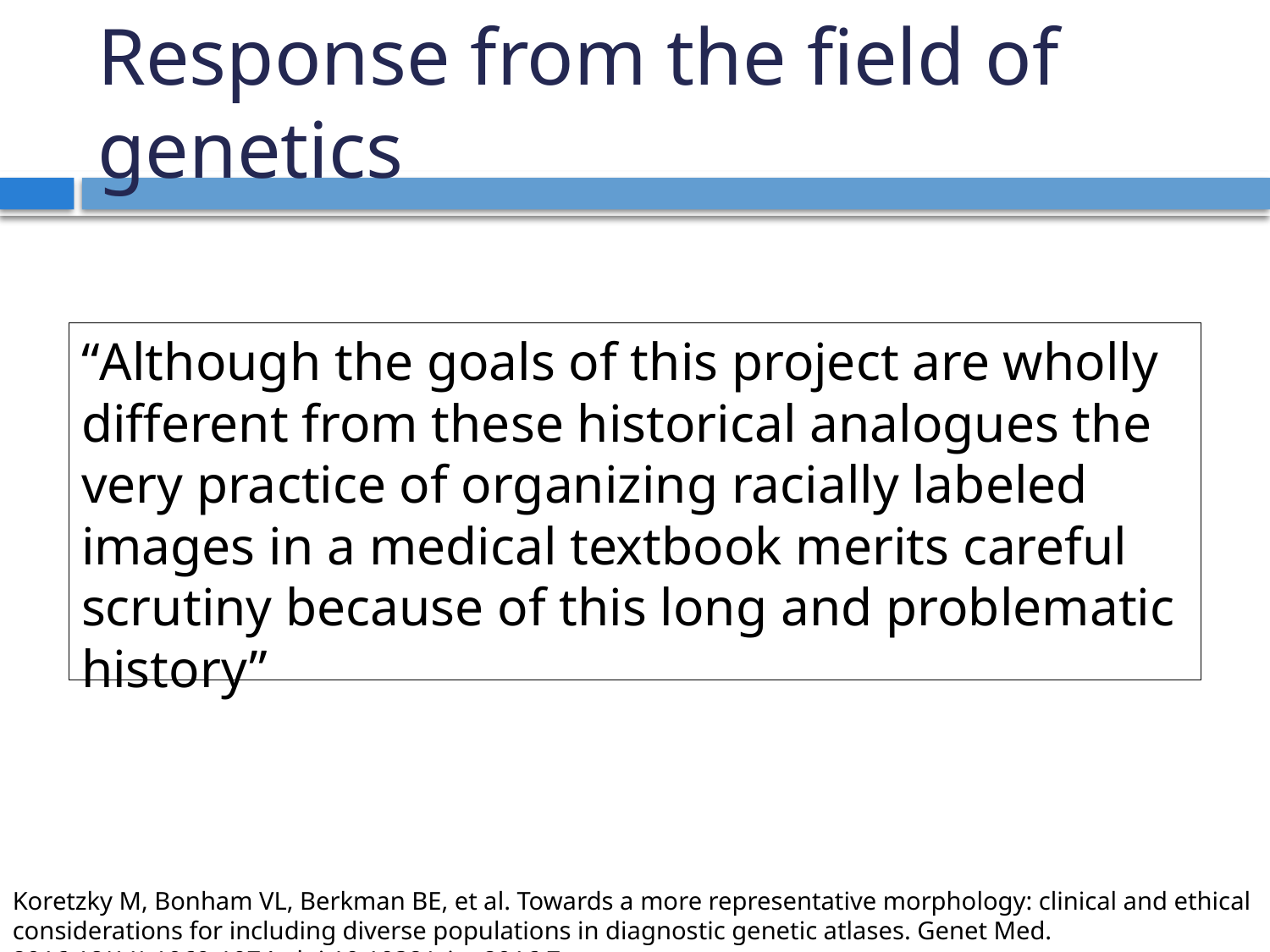

# Response from the field of genetics
“Although the goals of this project are wholly different from these historical analogues the very practice of organizing racially labeled images in a medical textbook merits careful scrutiny because of this long and problematic history”
Koretzky M, Bonham VL, Berkman BE, et al. Towards a more representative morphology: clinical and ethical considerations for including diverse populations in diagnostic genetic atlases. Genet Med. 2016;18(11):1069-1074. doi:10.1038/gim.2016.7

## Slide 28
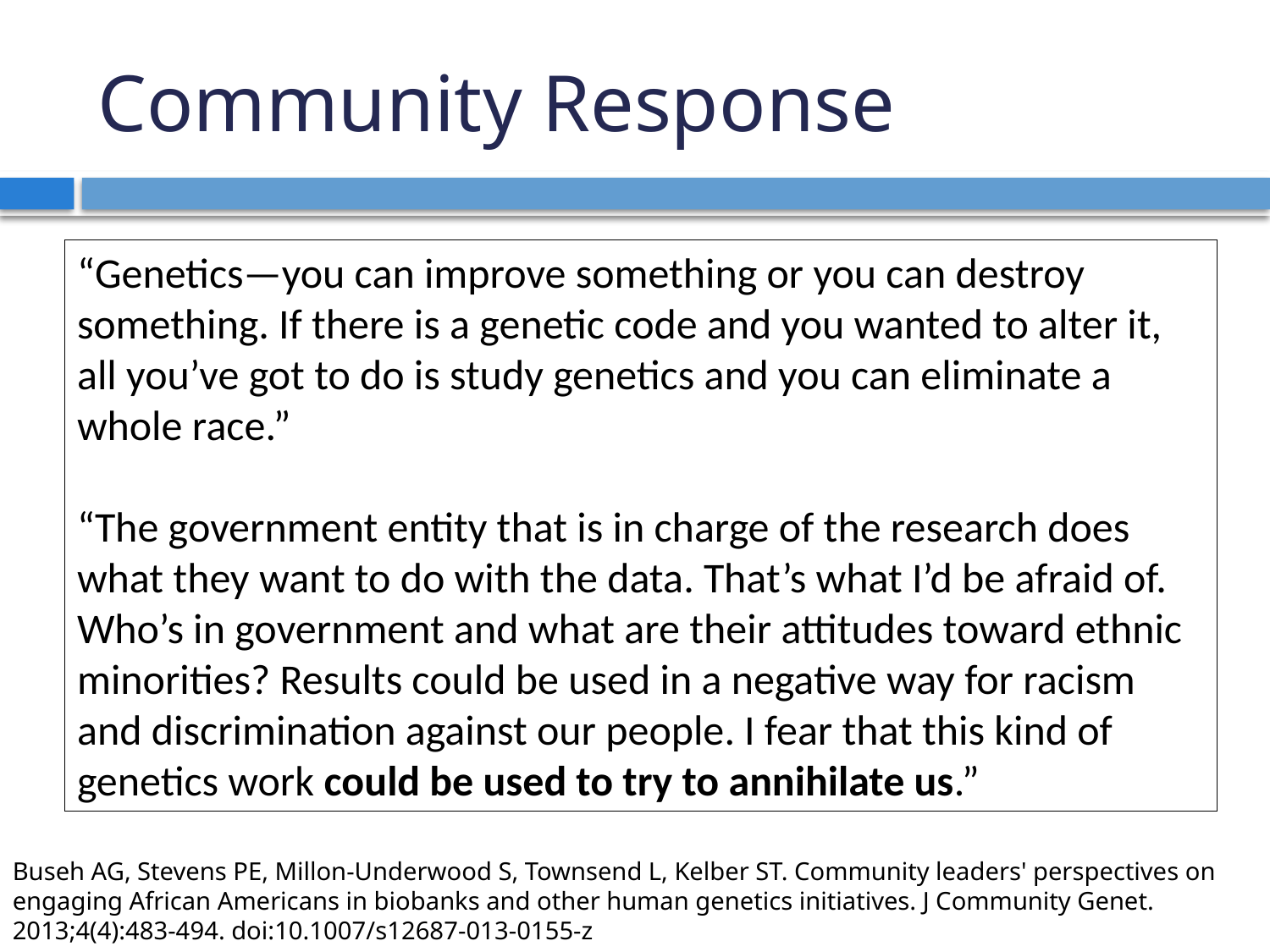

# Community Response
“Genetics—you can improve something or you can destroy something. If there is a genetic code and you wanted to alter it, all you’ve got to do is study genetics and you can eliminate a whole race.”
“The government entity that is in charge of the research does what they want to do with the data. That’s what I’d be afraid of. Who’s in government and what are their attitudes toward ethnic minorities? Results could be used in a negative way for racism and discrimination against our people. I fear that this kind of genetics work could be used to try to annihilate us.”
Buseh AG, Stevens PE, Millon-Underwood S, Townsend L, Kelber ST. Community leaders' perspectives on engaging African Americans in biobanks and other human genetics initiatives. J Community Genet. 2013;4(4):483-494. doi:10.1007/s12687-013-0155-z

## Slide 29
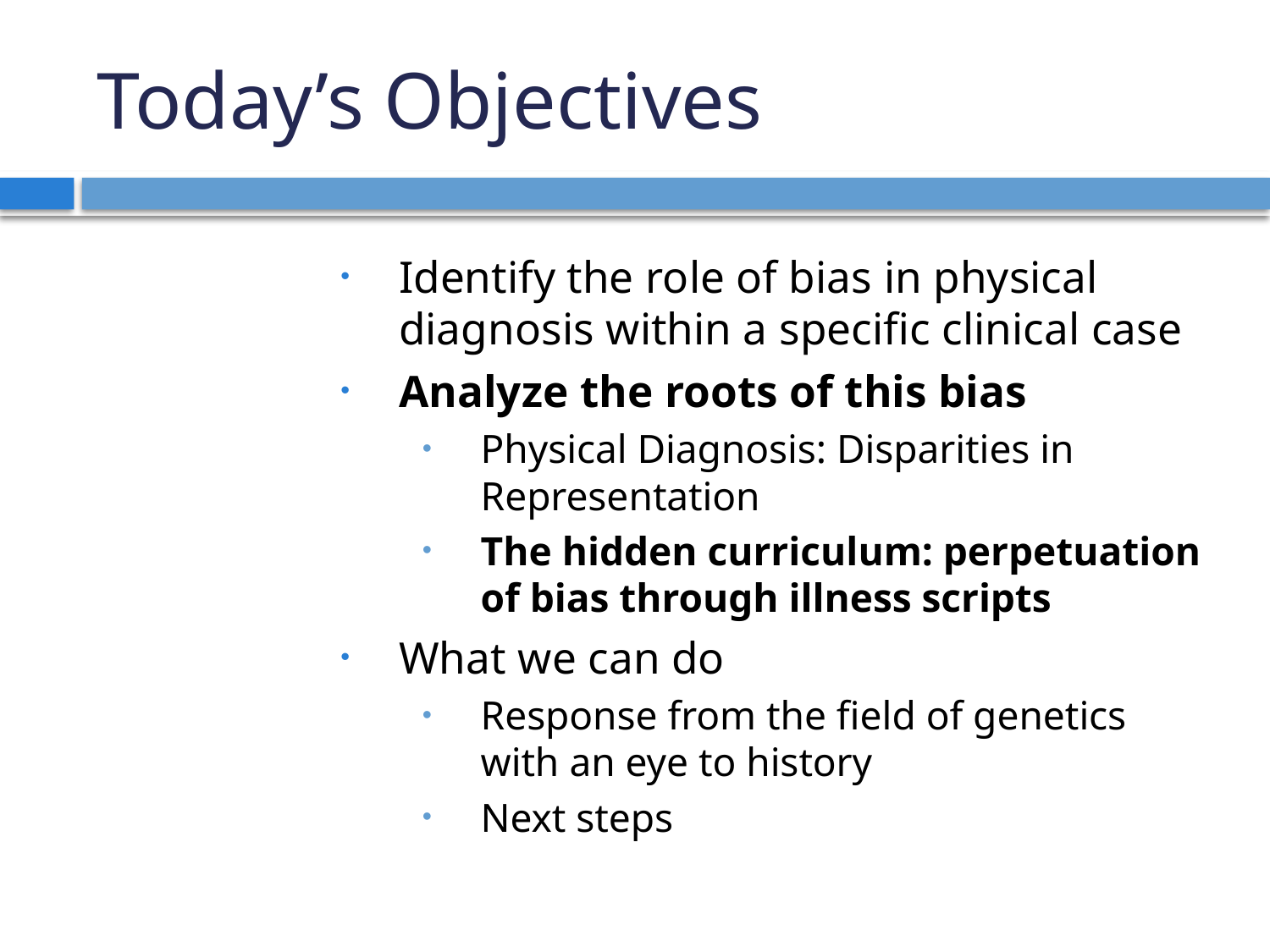

# Today’s Objectives
Identify the role of bias in physical diagnosis within a specific clinical case
Analyze the roots of this bias
Physical Diagnosis: Disparities in Representation
The hidden curriculum: perpetuation of bias through illness scripts
What we can do
Response from the field of genetics with an eye to history
Next steps

## Slide 30
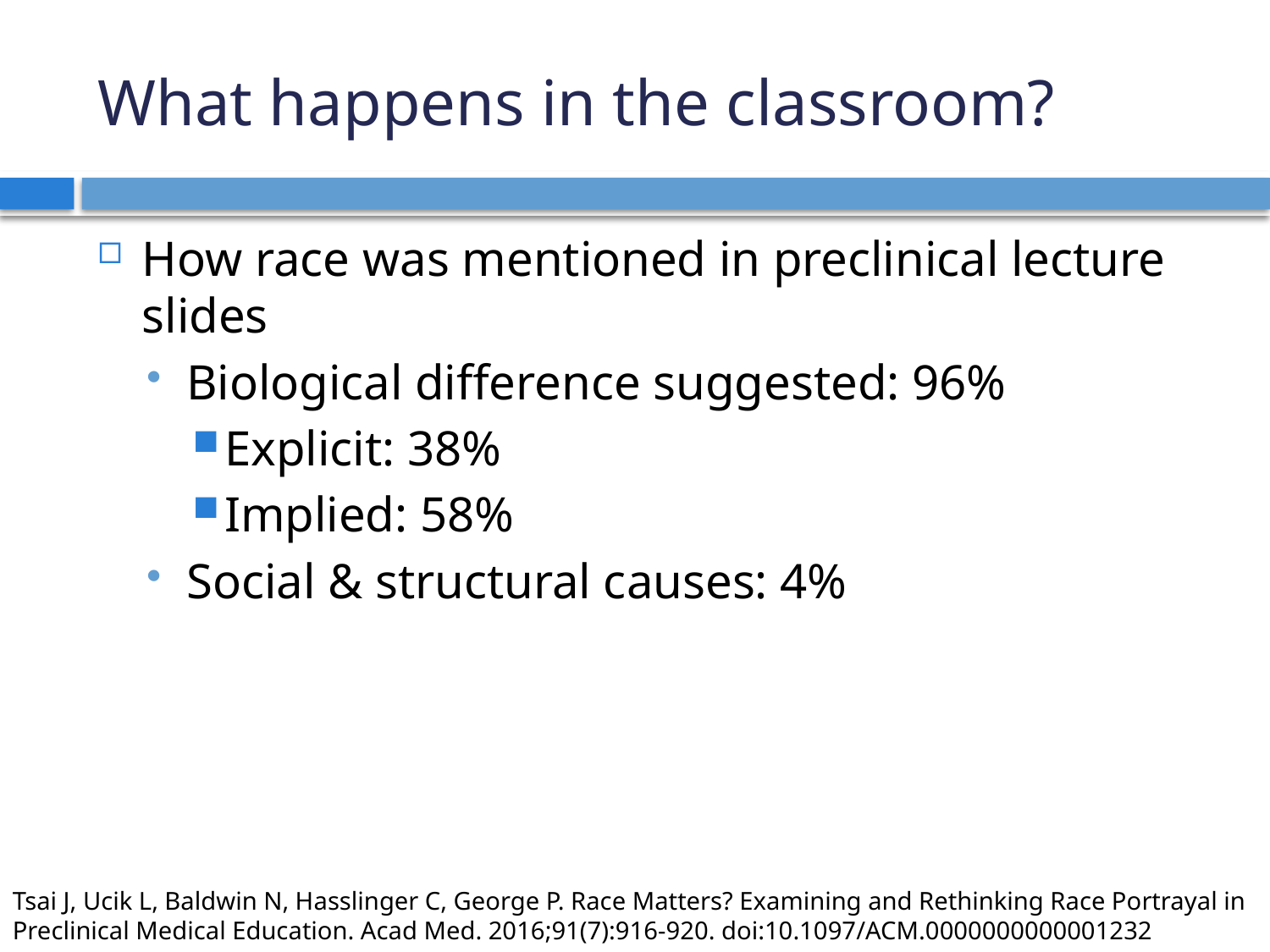

# What happens in the classroom?
How race was mentioned in preclinical lecture slides
Biological difference suggested: 96%
Explicit: 38%
Implied: 58%
Social & structural causes: 4%
Tsai J, Ucik L, Baldwin N, Hasslinger C, George P. Race Matters? Examining and Rethinking Race Portrayal in Preclinical Medical Education. Acad Med. 2016;91(7):916-920. doi:10.1097/ACM.0000000000001232

## Slide 31
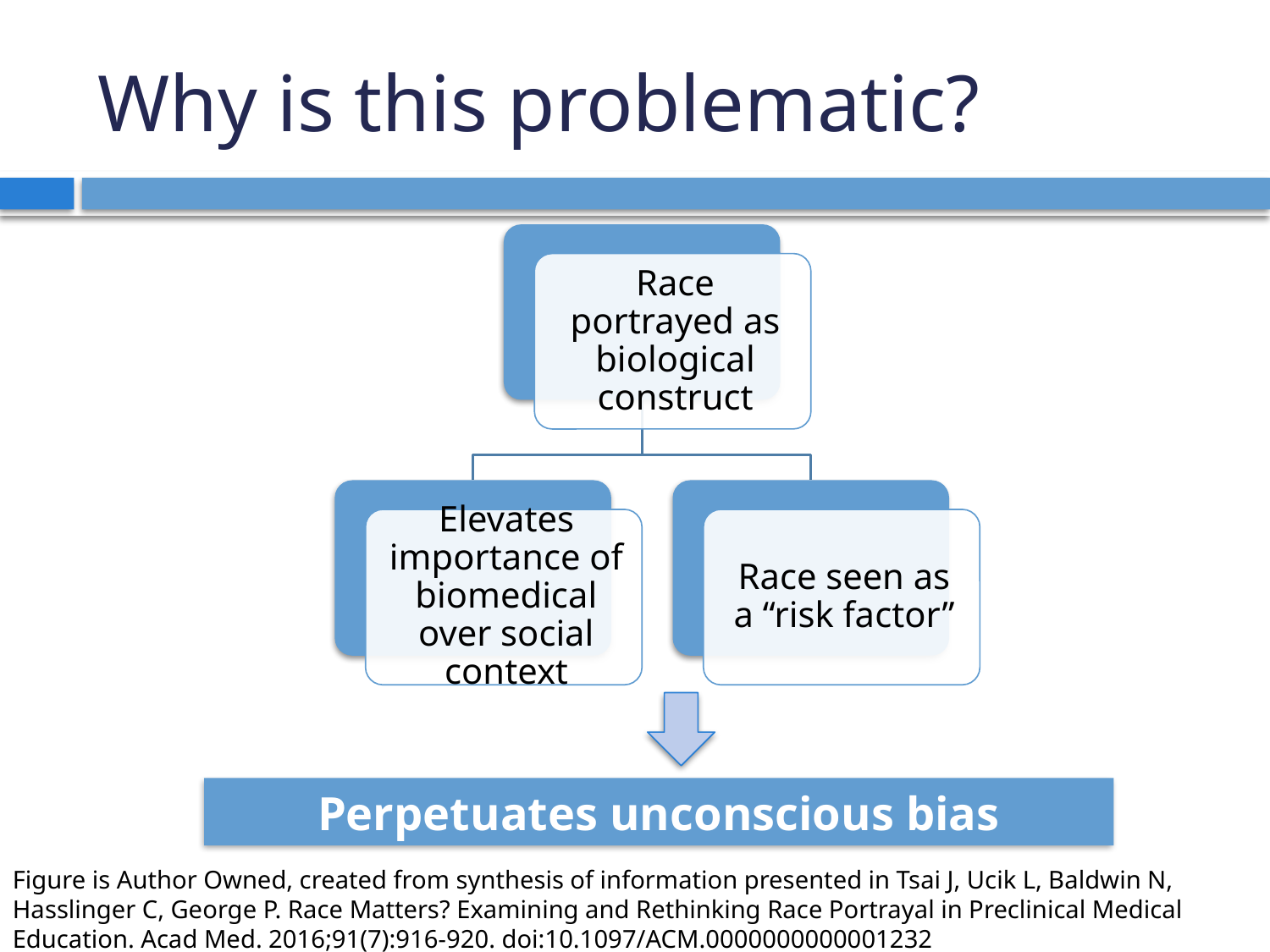

# Why is this problematic?
Perpetuates unconscious bias
Figure is Author Owned, created from synthesis of information presented in Tsai J, Ucik L, Baldwin N, Hasslinger C, George P. Race Matters? Examining and Rethinking Race Portrayal in Preclinical Medical Education. Acad Med. 2016;91(7):916-920. doi:10.1097/ACM.0000000000001232

## Slide 32
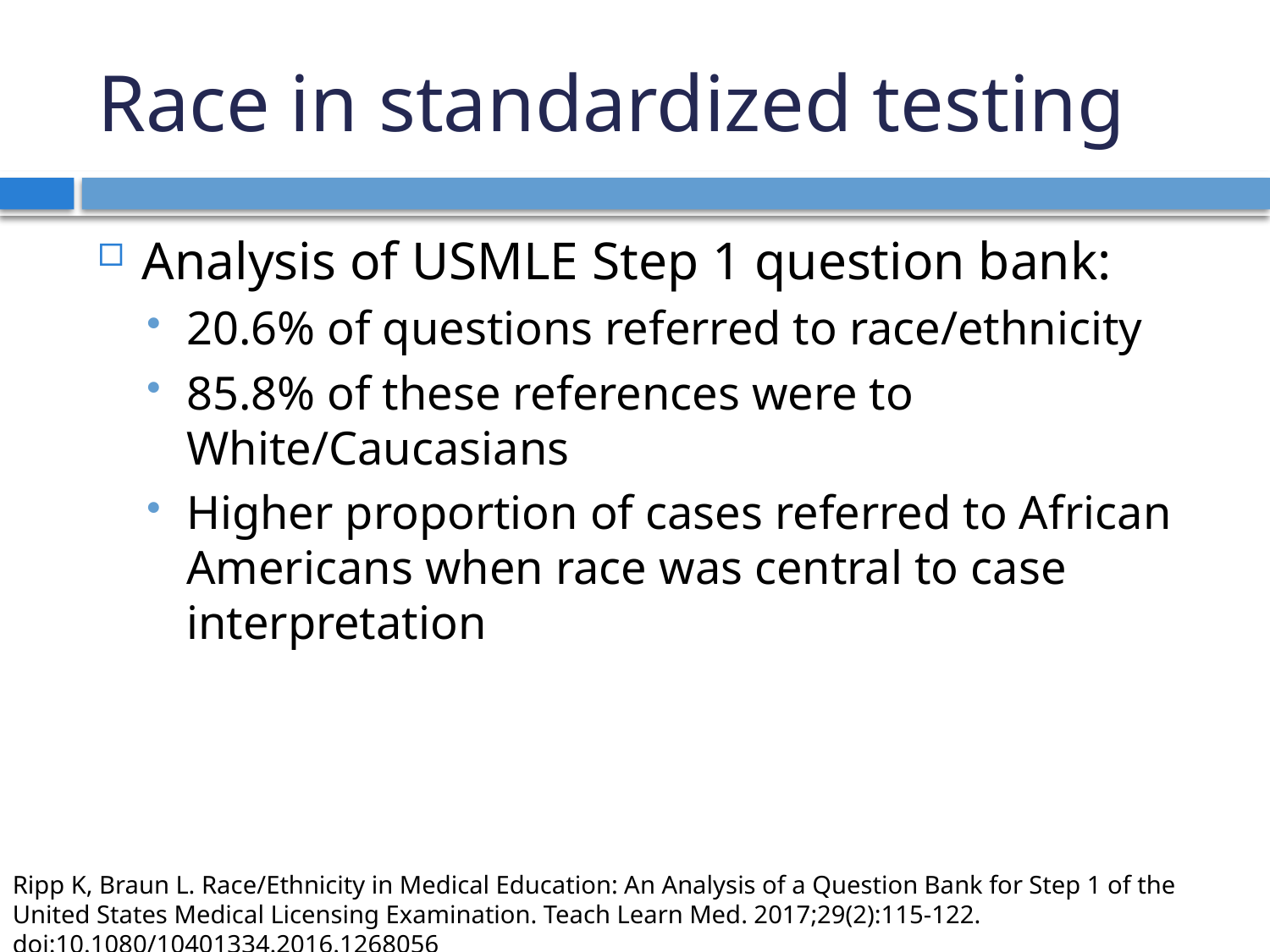

# Race in standardized testing
Analysis of USMLE Step 1 question bank:
20.6% of questions referred to race/ethnicity
85.8% of these references were to White/Caucasians
Higher proportion of cases referred to African Americans when race was central to case interpretation
Ripp K, Braun L. Race/Ethnicity in Medical Education: An Analysis of a Question Bank for Step 1 of the United States Medical Licensing Examination. Teach Learn Med. 2017;29(2):115-122. doi:10.1080/10401334.2016.1268056

## Slide 33
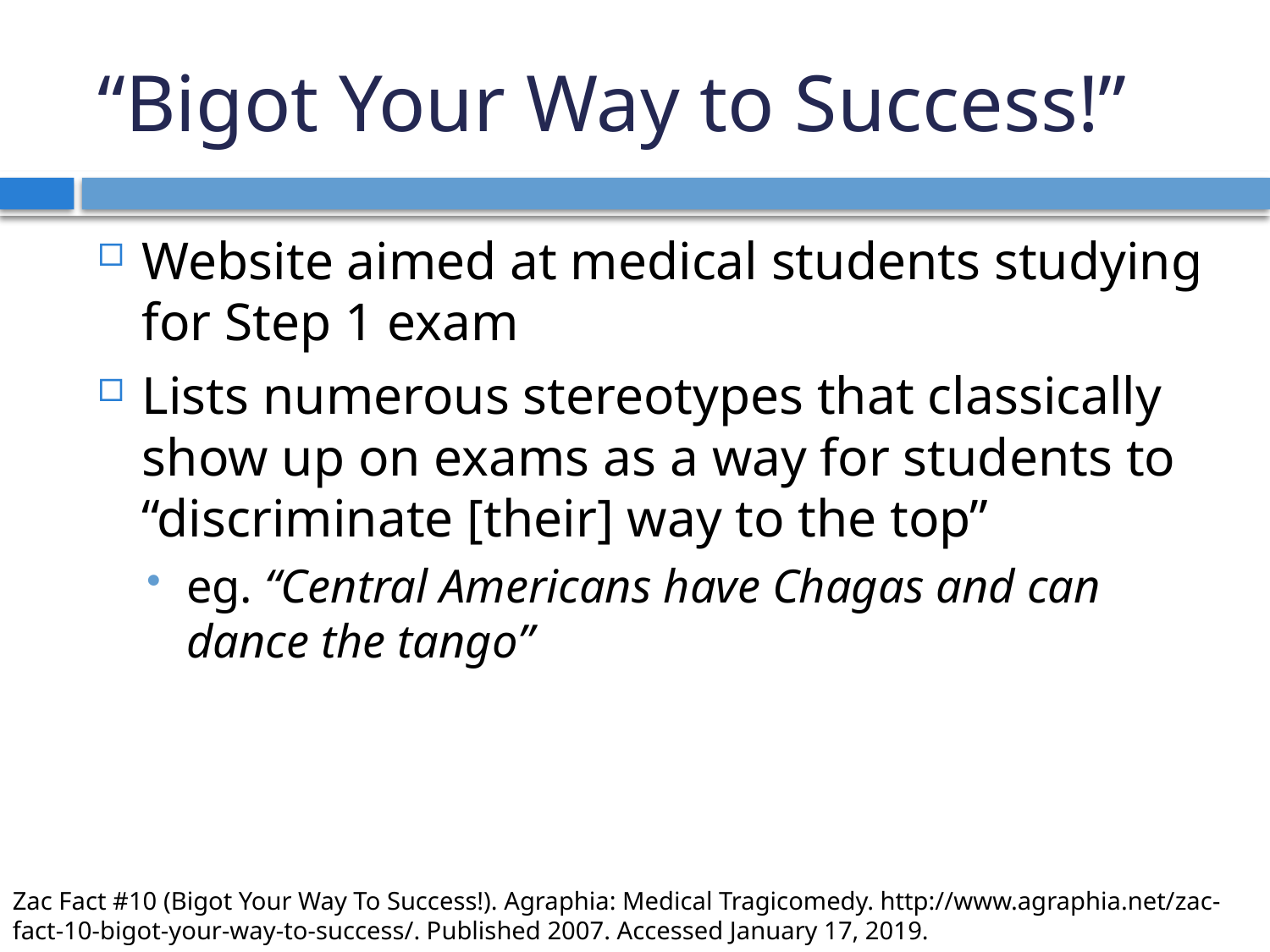

# “Bigot Your Way to Success!”
Website aimed at medical students studying for Step 1 exam
Lists numerous stereotypes that classically show up on exams as a way for students to “discriminate [their] way to the top”
eg. “Central Americans have Chagas and can dance the tango”
Zac Fact #10 (Bigot Your Way To Success!). Agraphia: Medical Tragicomedy. http://www.agraphia.net/zac-fact-10-bigot-your-way-to-success/. Published 2007. Accessed January 17, 2019.

## Slide 34
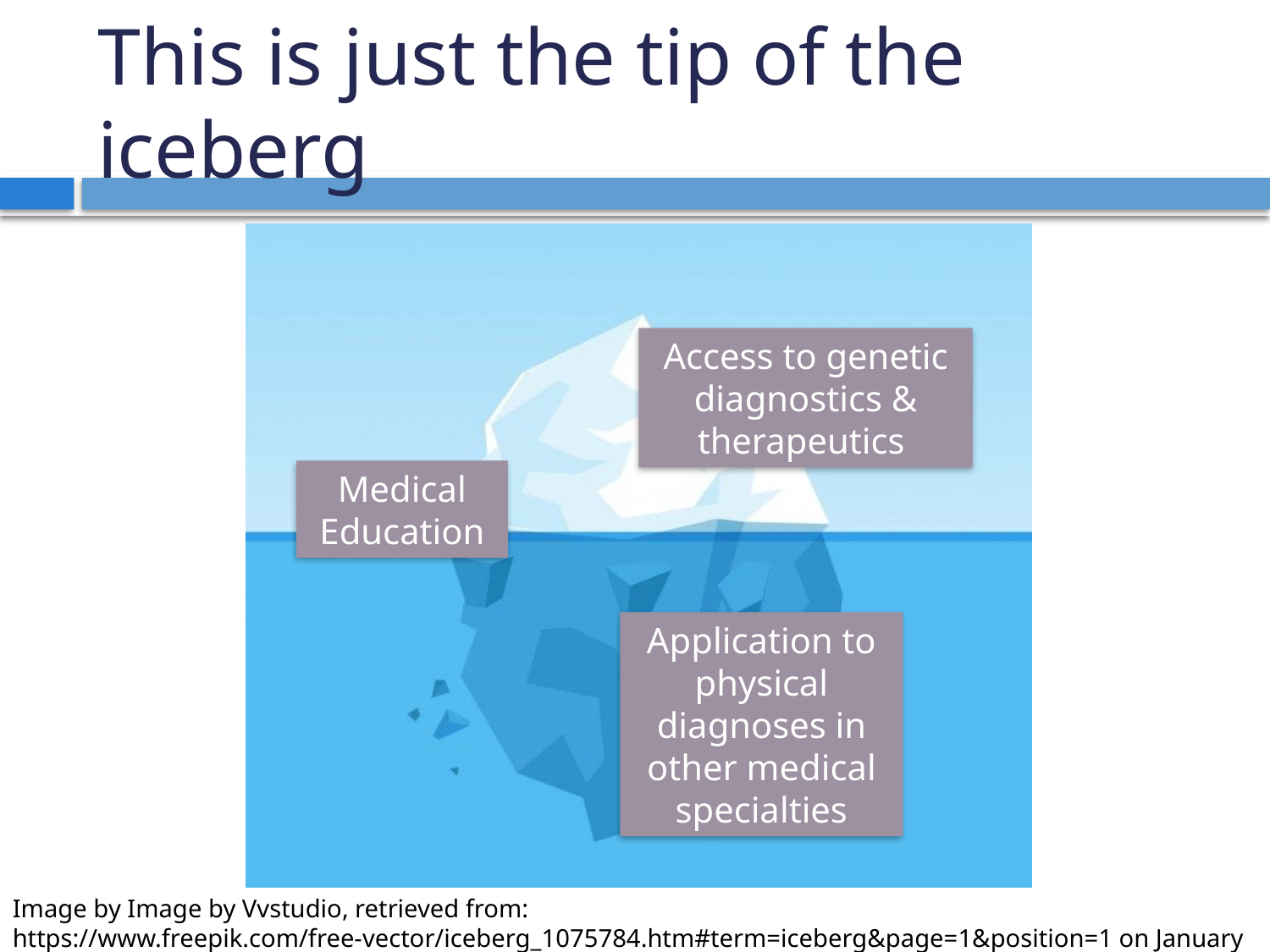

# This is just the tip of the iceberg
Access to genetic diagnostics & therapeutics
Medical Education
Application to physical diagnoses in other medical specialties
Image by Image by Vvstudio, retrieved from: https://www.freepik.com/free-vector/iceberg_1075784.htm#term=iceberg&page=1&position=1 on January 6, 2019. Image is in the public domain.”

## Slide 35
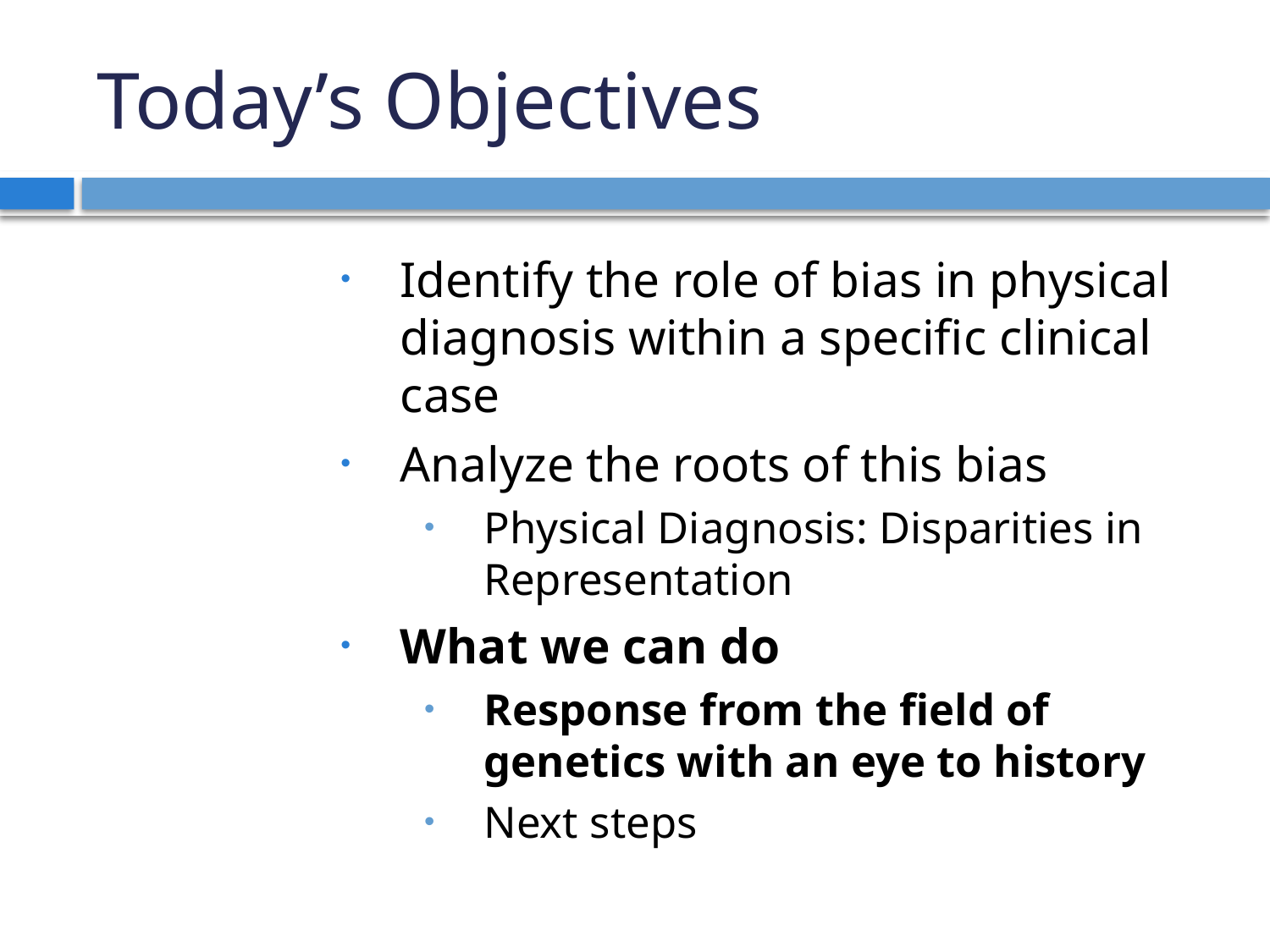

# Today’s Objectives
Identify the role of bias in physical diagnosis within a specific clinical case
Analyze the roots of this bias
Physical Diagnosis: Disparities in Representation
What we can do
Response from the field of genetics with an eye to history
Next steps

## Slide 36
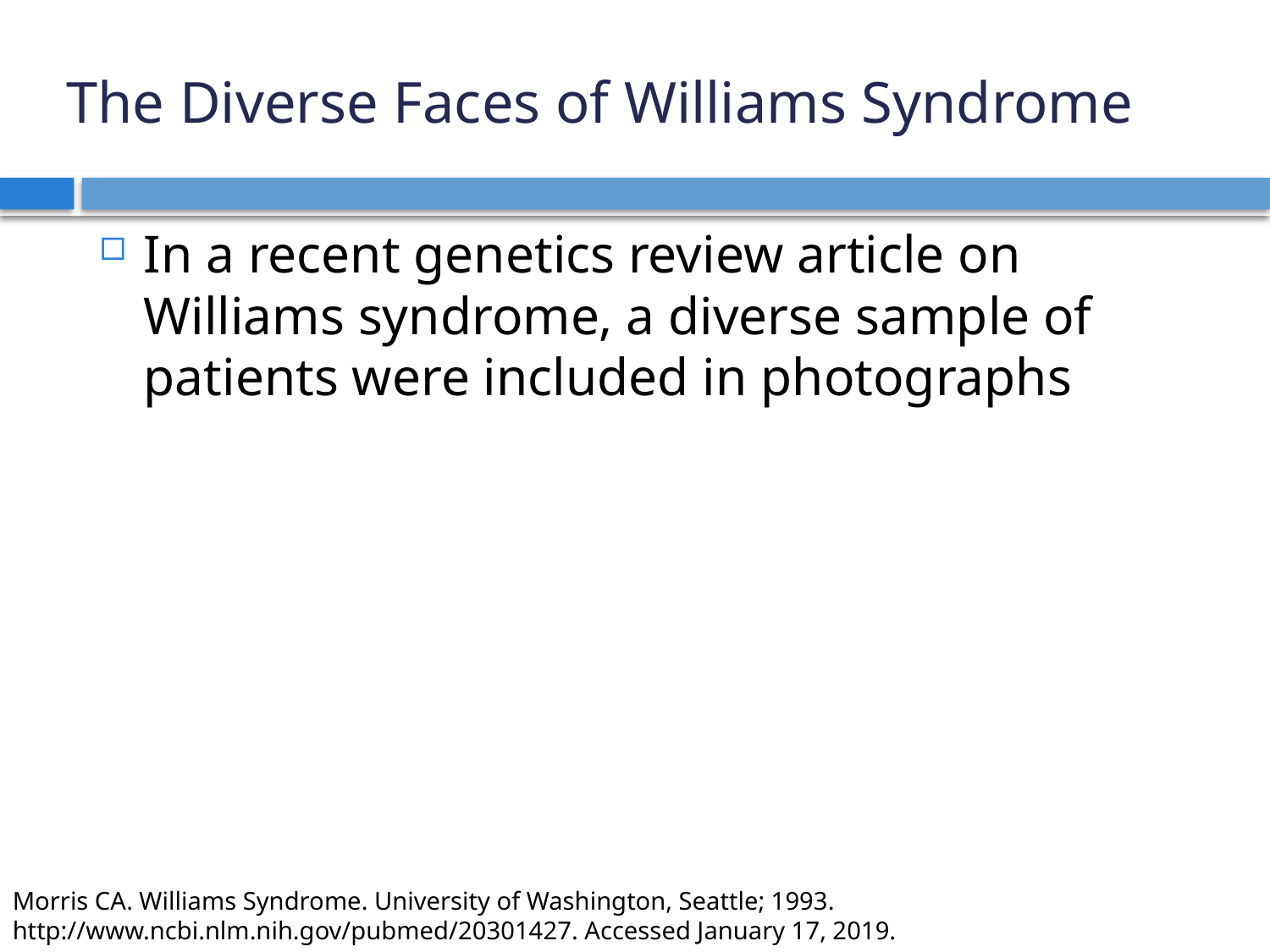

# The Diverse Faces of Williams Syndrome
In a recent genetics review article on Williams syndrome, a diverse sample of patients were included in photographs
Morris CA. Williams Syndrome. University of Washington, Seattle; 1993. http://www.ncbi.nlm.nih.gov/pubmed/20301427. Accessed January 17, 2019.

## Slide 37
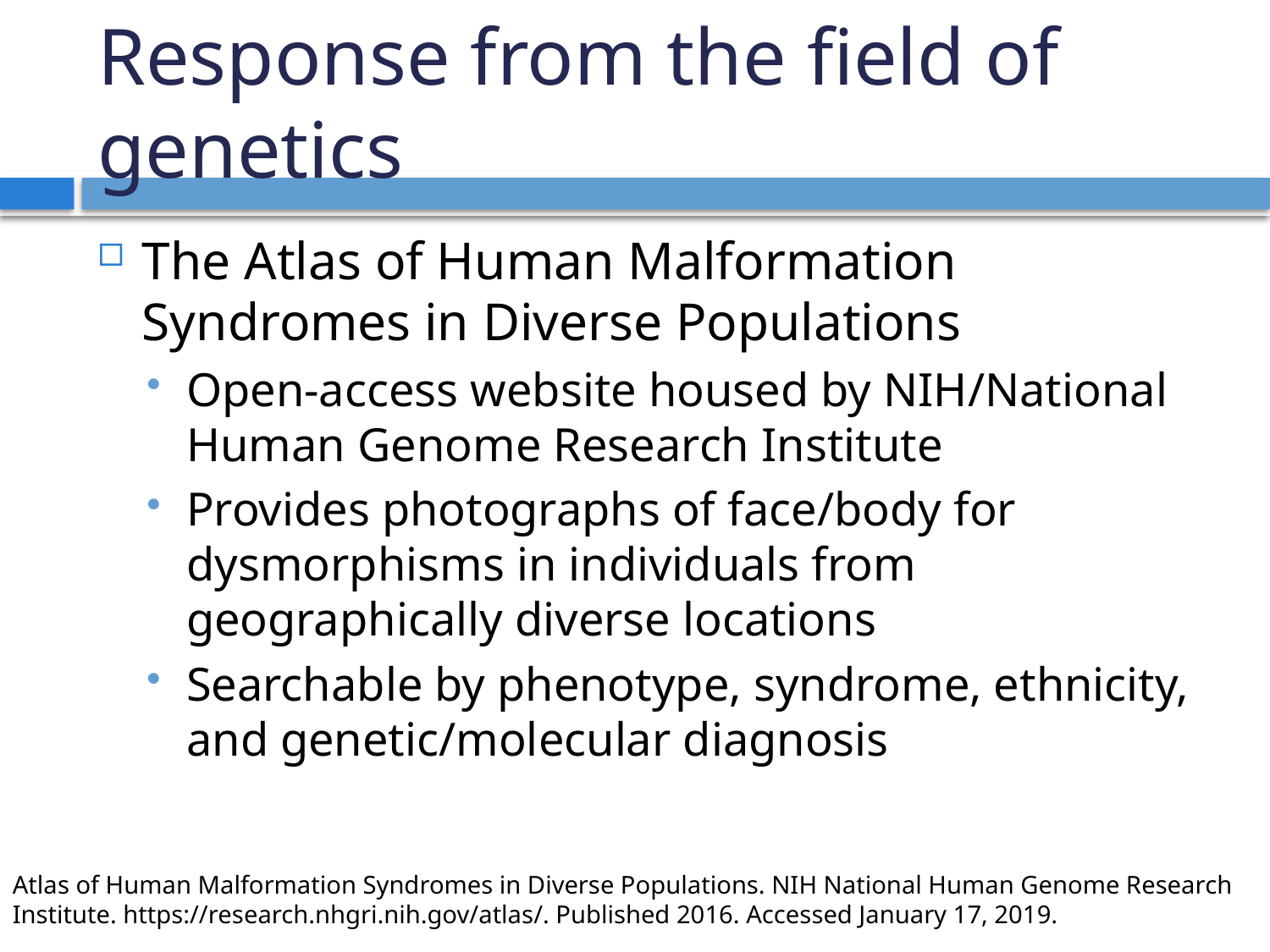

# Response from the field of genetics
The Atlas of Human Malformation Syndromes in Diverse Populations
Open-access website housed by NIH/National Human Genome Research Institute
Provides photographs of face/body for dysmorphisms in individuals from geographically diverse locations
Searchable by phenotype, syndrome, ethnicity, and genetic/molecular diagnosis
Atlas of Human Malformation Syndromes in Diverse Populations. NIH National Human Genome Research Institute. https://research.nhgri.nih.gov/atlas/. Published 2016. Accessed January 17, 2019.

## Slide 38
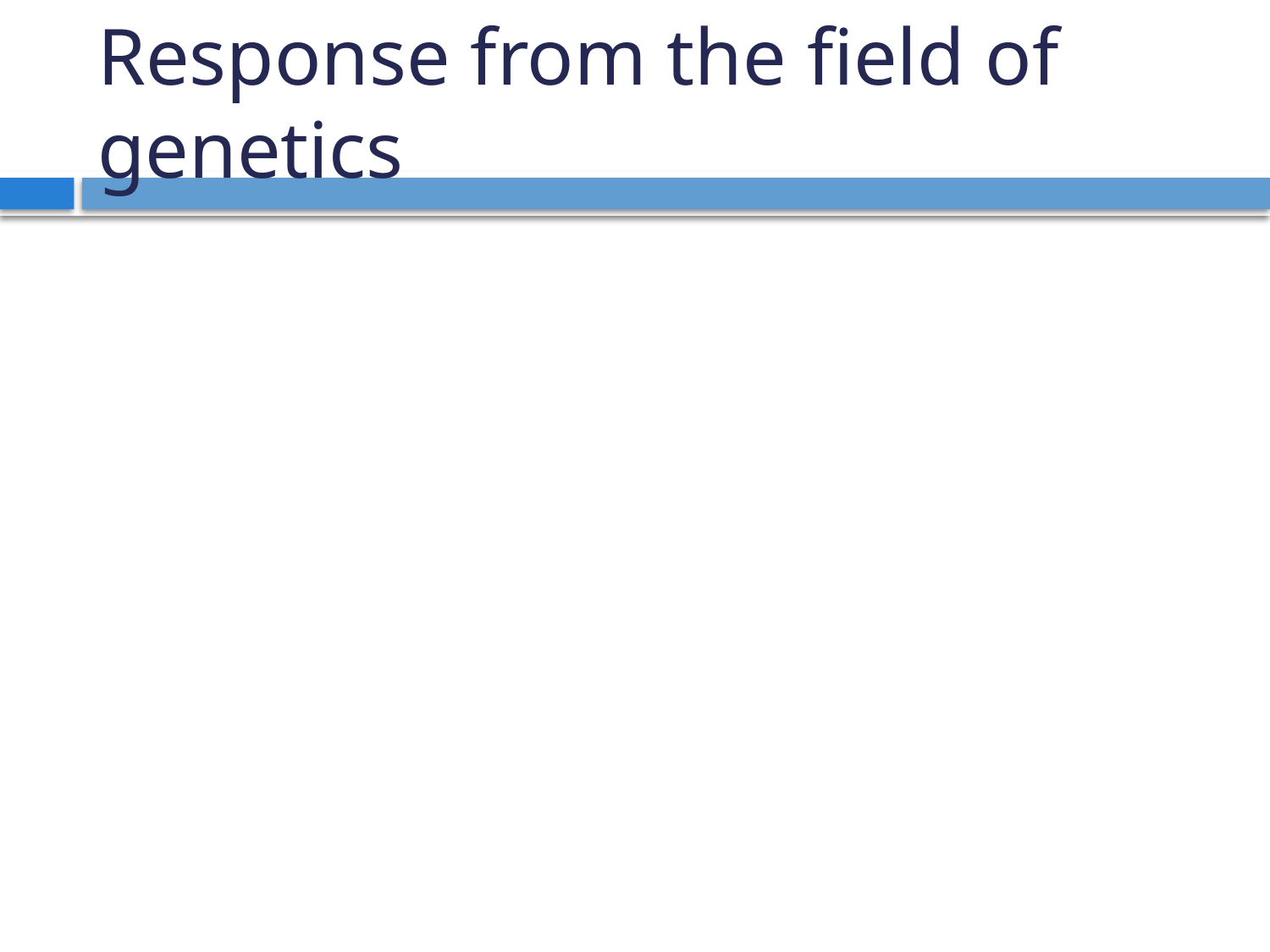

# Response from the field of genetics

## Slide 39
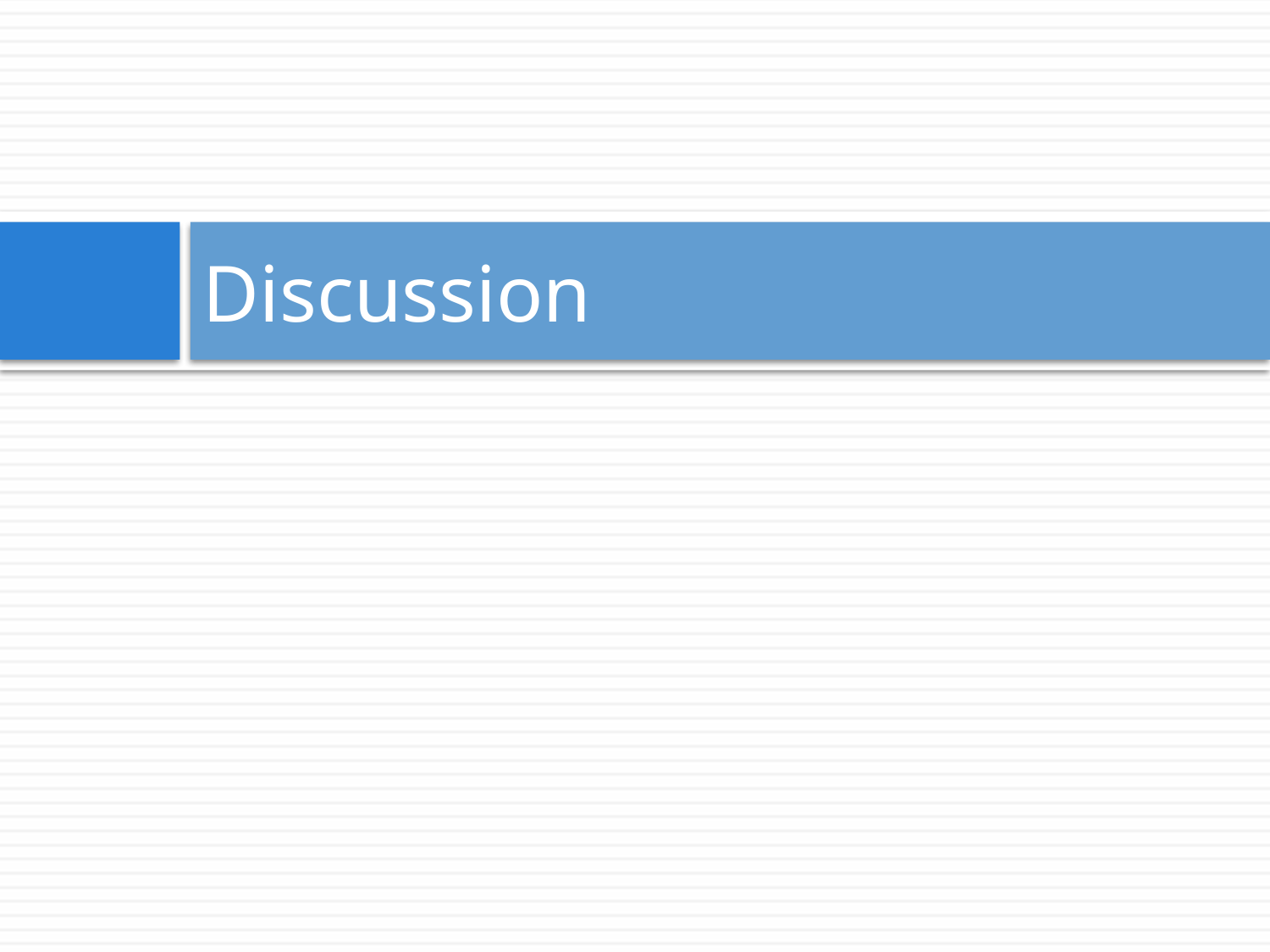

# Discussion

## Slide 40
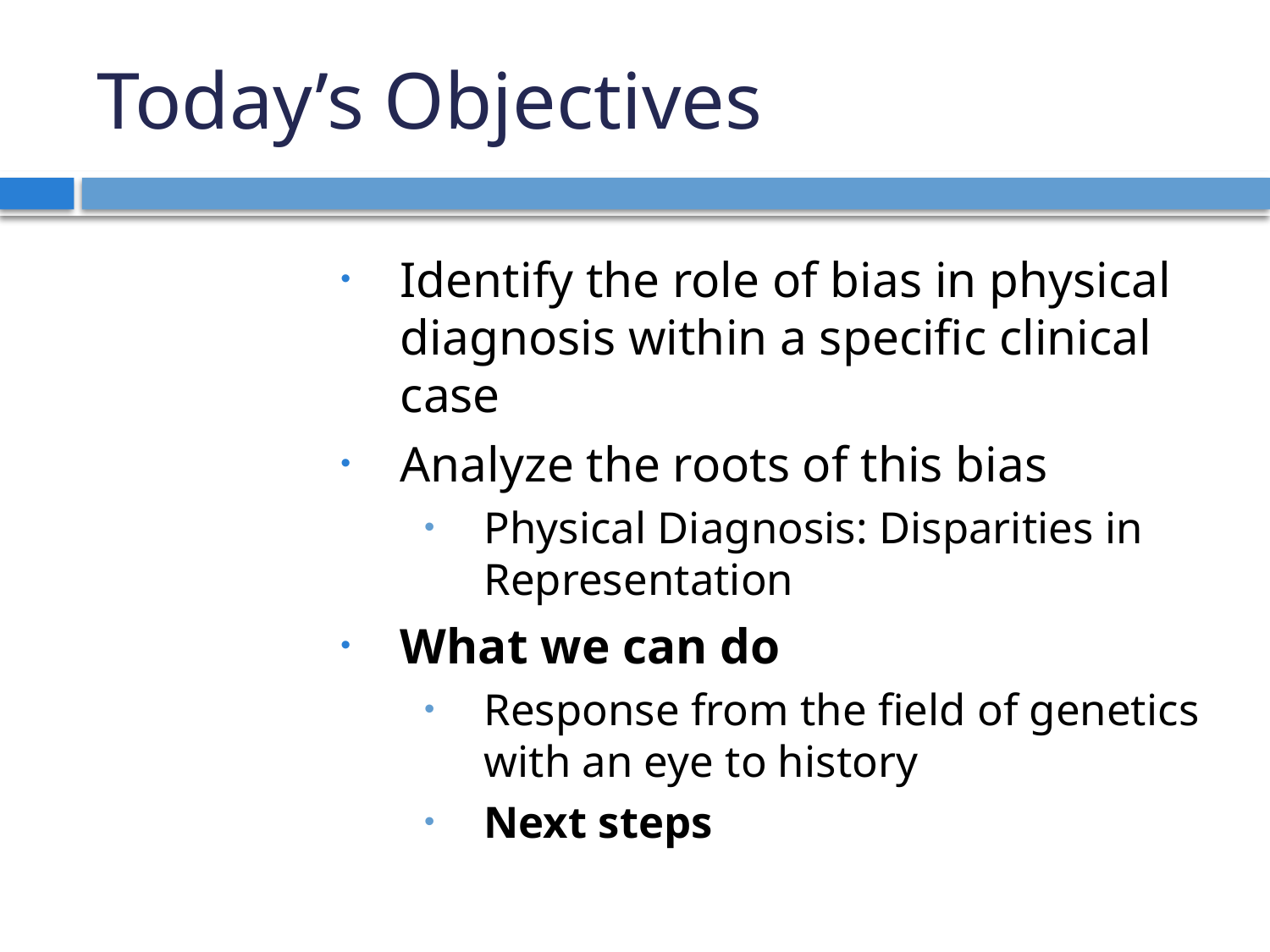

# Today’s Objectives
Identify the role of bias in physical diagnosis within a specific clinical case
Analyze the roots of this bias
Physical Diagnosis: Disparities in Representation
What we can do
Response from the field of genetics with an eye to history
Next steps

## Slide 41
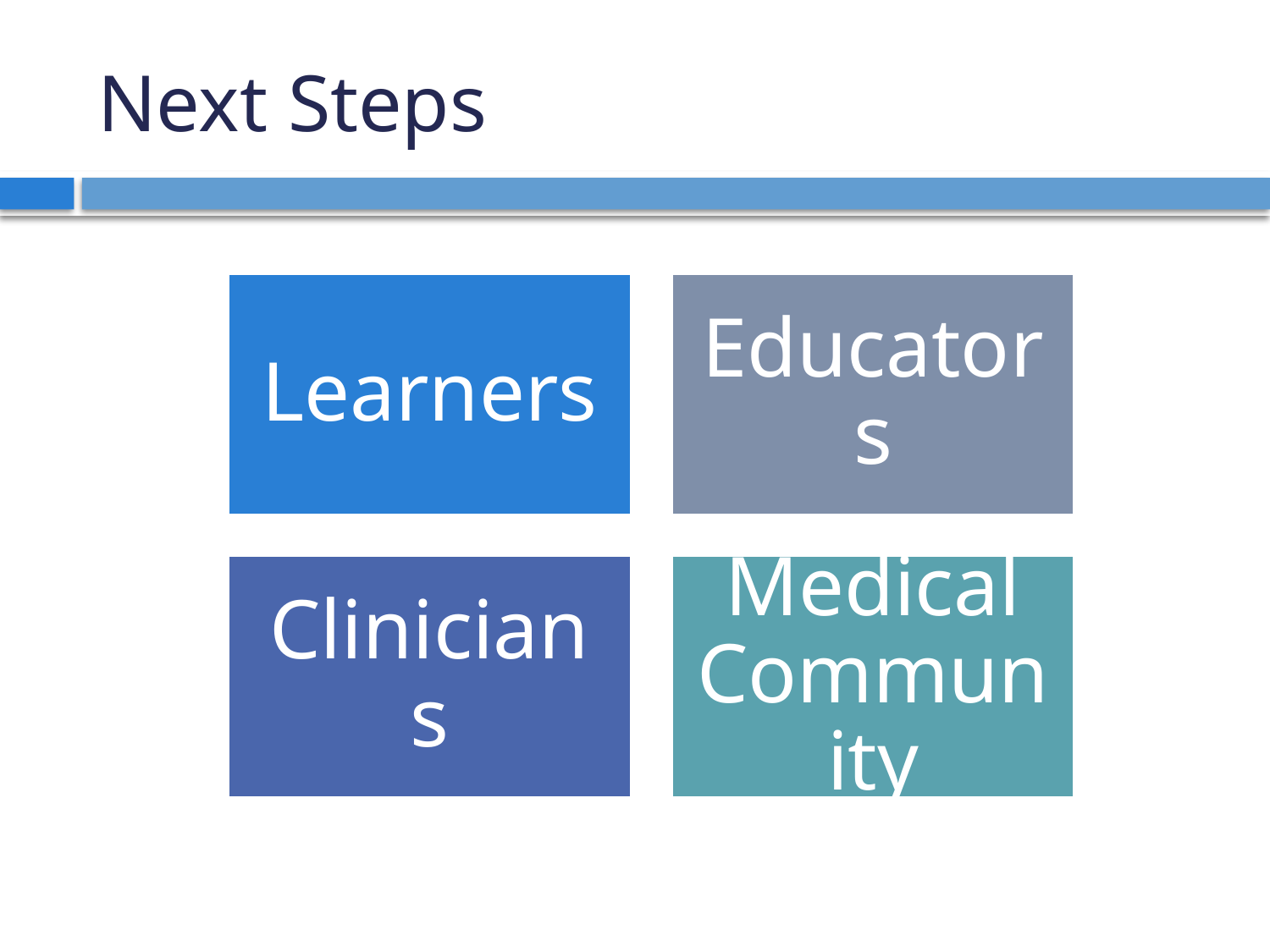

# Next Steps

## Slide 42
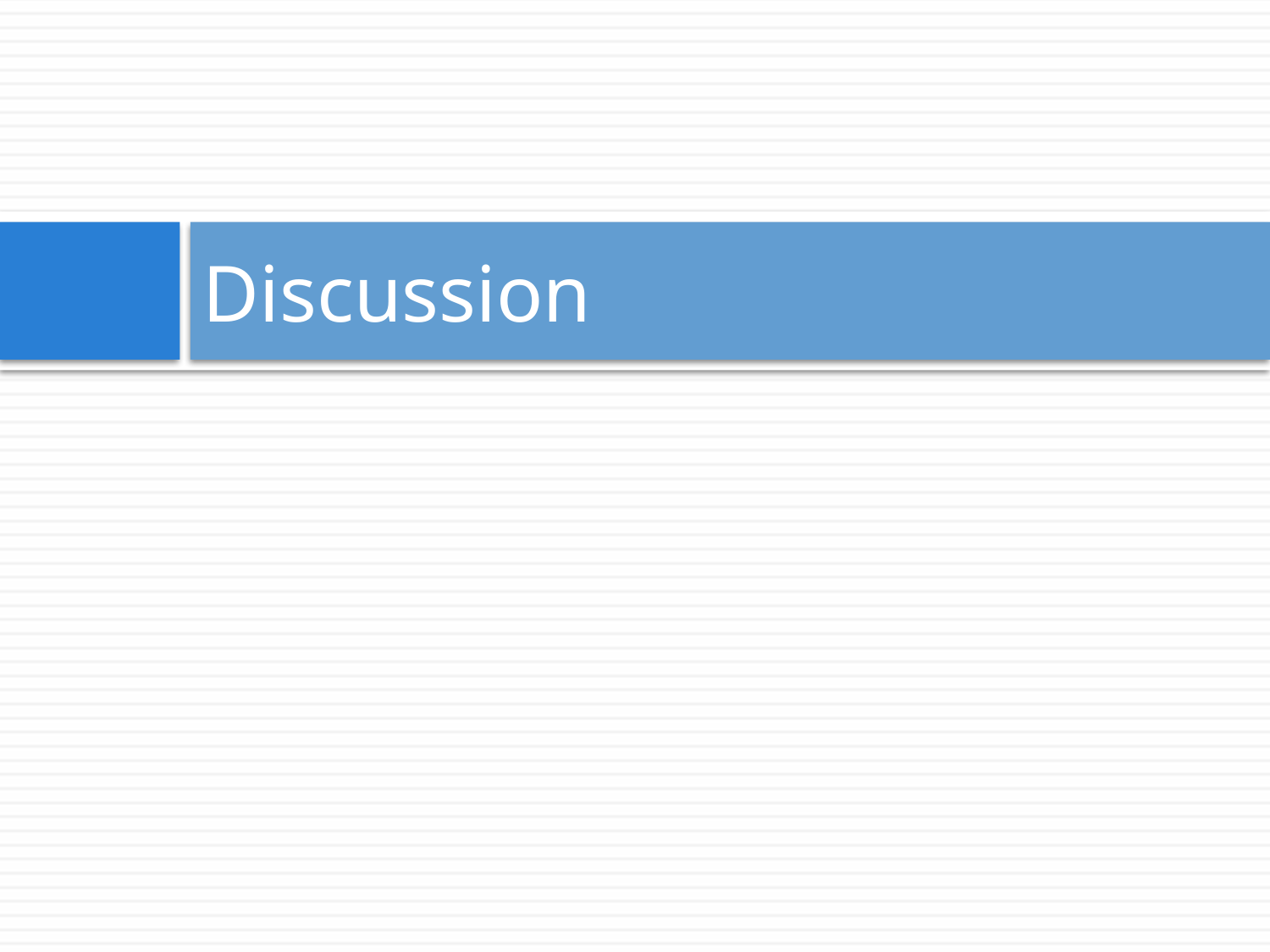

# Discussion

## Slide 43
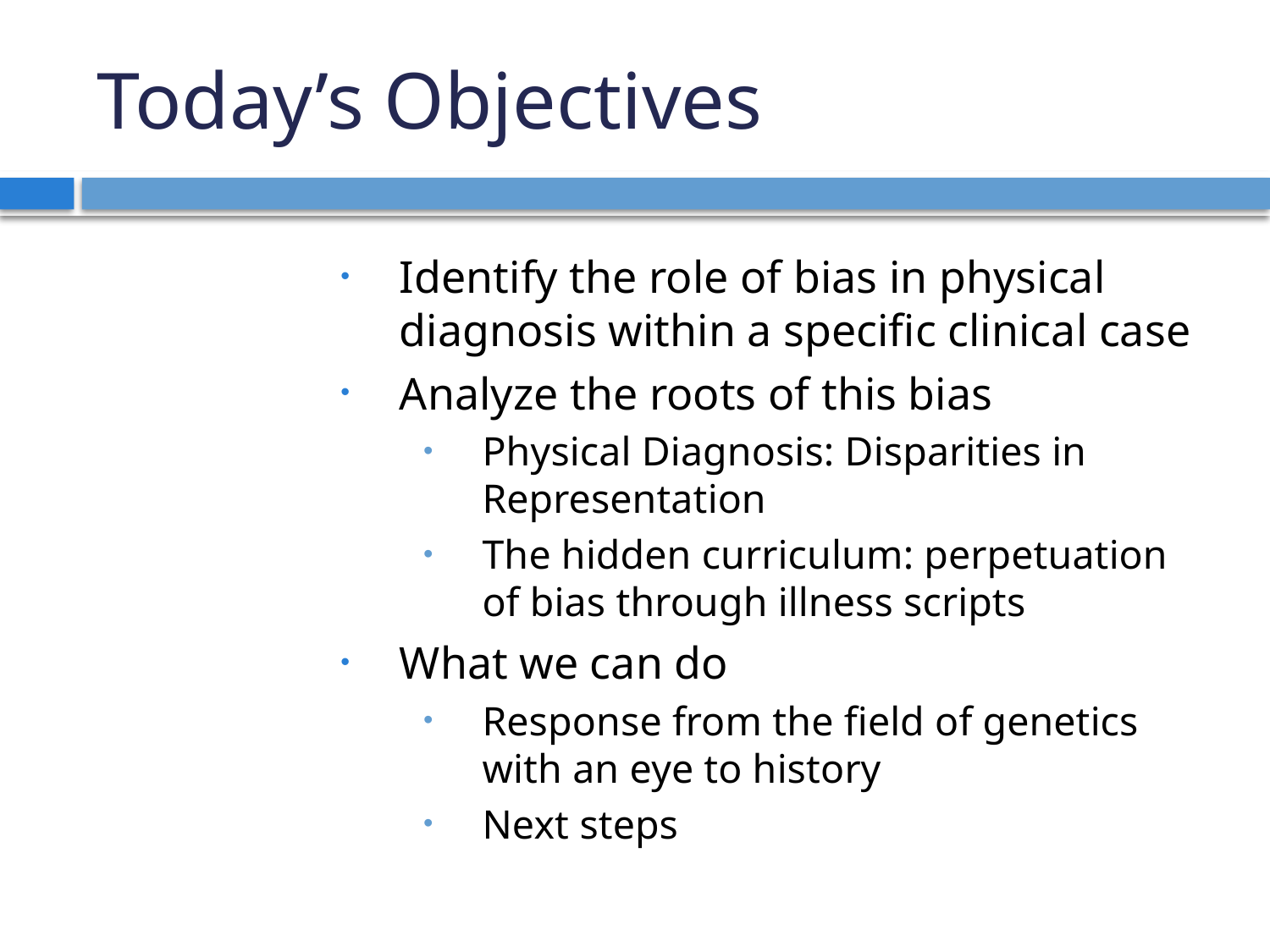

# Today’s Objectives
Identify the role of bias in physical diagnosis within a specific clinical case
Analyze the roots of this bias
Physical Diagnosis: Disparities in Representation
The hidden curriculum: perpetuation of bias through illness scripts
What we can do
Response from the field of genetics with an eye to history
Next steps

## Slide 44
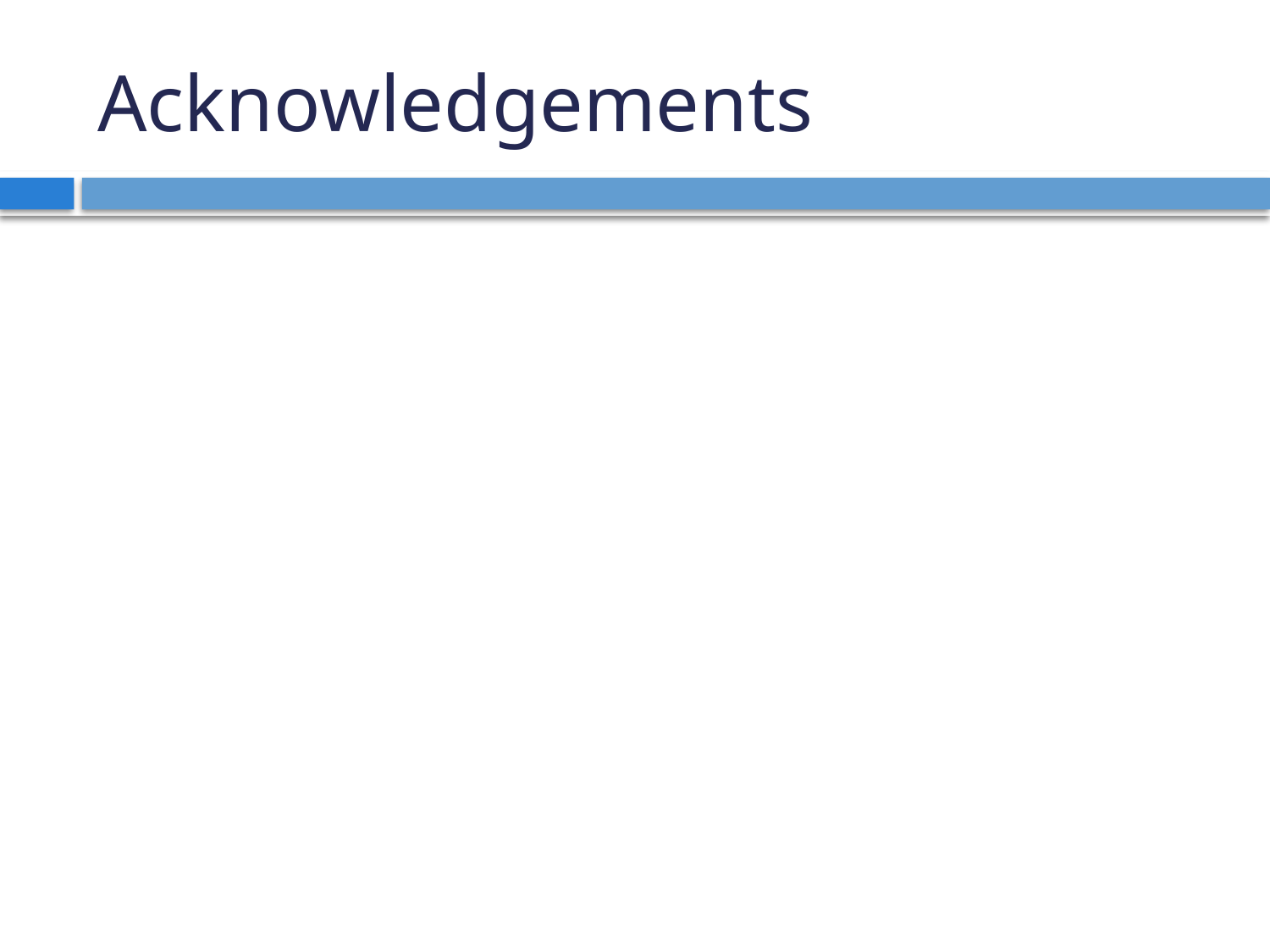

# Acknowledgements

## Slide 45
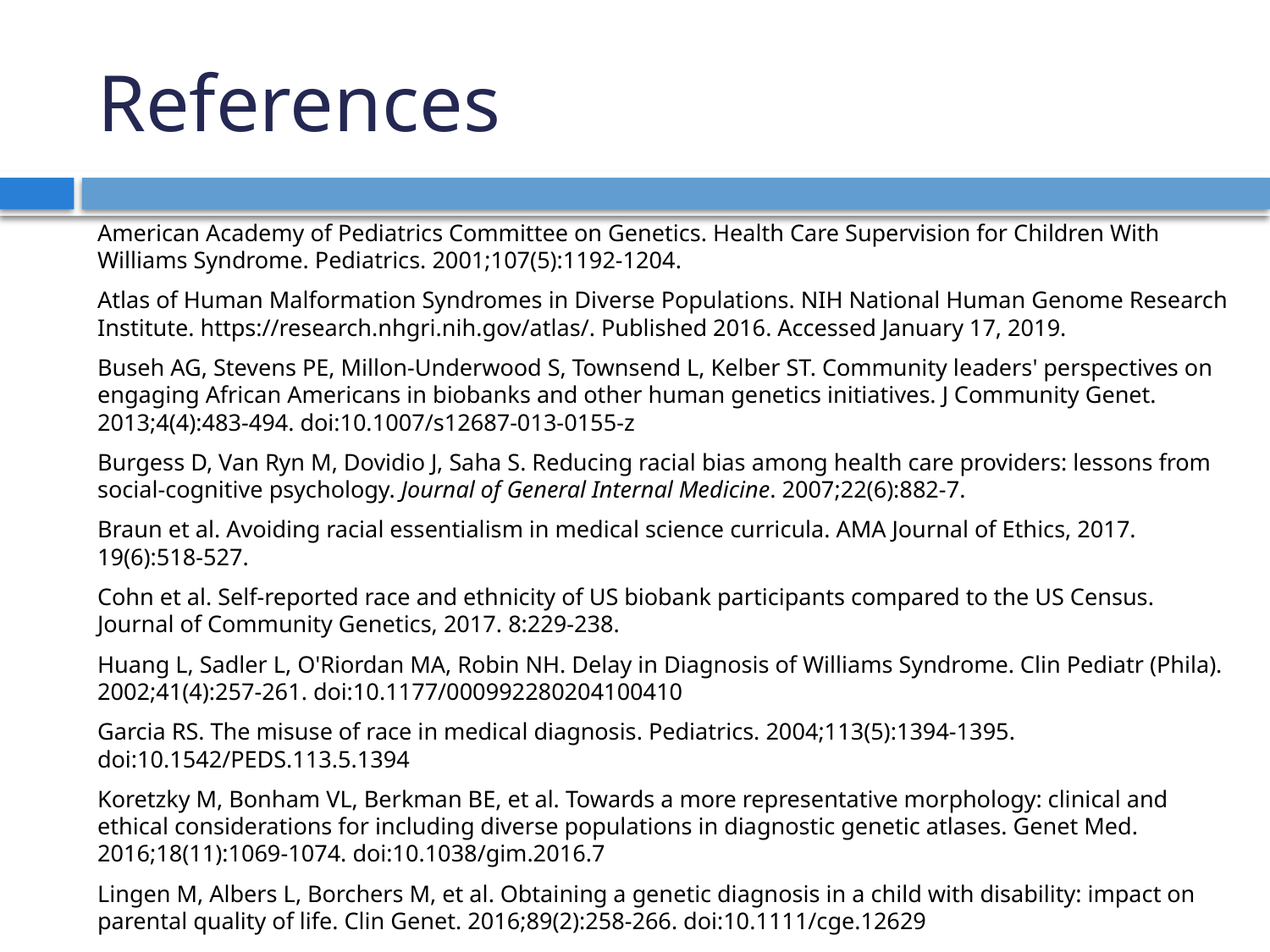

# References
American Academy of Pediatrics Committee on Genetics. Health Care Supervision for Children With Williams Syndrome. Pediatrics. 2001;107(5):1192-1204.
Atlas of Human Malformation Syndromes in Diverse Populations. NIH National Human Genome Research Institute. https://research.nhgri.nih.gov/atlas/. Published 2016. Accessed January 17, 2019.
Buseh AG, Stevens PE, Millon-Underwood S, Townsend L, Kelber ST. Community leaders' perspectives on engaging African Americans in biobanks and other human genetics initiatives. J Community Genet. 2013;4(4):483-494. doi:10.1007/s12687-013-0155-z
Burgess D, Van Ryn M, Dovidio J, Saha S. Reducing racial bias among health care providers: lessons from social-cognitive psychology. Journal of General Internal Medicine. 2007;22(6):882-7.​
Braun et al. Avoiding racial essentialism in medical science curricula. AMA Journal of Ethics, 2017. 19(6):518-527.
Cohn et al. Self-reported race and ethnicity of US biobank participants compared to the US Census. Journal of Community Genetics, 2017. 8:229-238.
Huang L, Sadler L, O'Riordan MA, Robin NH. Delay in Diagnosis of Williams Syndrome. Clin Pediatr (Phila). 2002;41(4):257-261. doi:10.1177/000992280204100410
Garcia RS. The misuse of race in medical diagnosis. Pediatrics. 2004;113(5):1394-1395. doi:10.1542/PEDS.113.5.1394
Koretzky M, Bonham VL, Berkman BE, et al. Towards a more representative morphology: clinical and ethical considerations for including diverse populations in diagnostic genetic atlases. Genet Med. 2016;18(11):1069-1074. doi:10.1038/gim.2016.7
Lingen M, Albers L, Borchers M, et al. Obtaining a genetic diagnosis in a child with disability: impact on parental quality of life. Clin Genet. 2016;89(2):258-266. doi:10.1111/cge.12629

## Slide 46
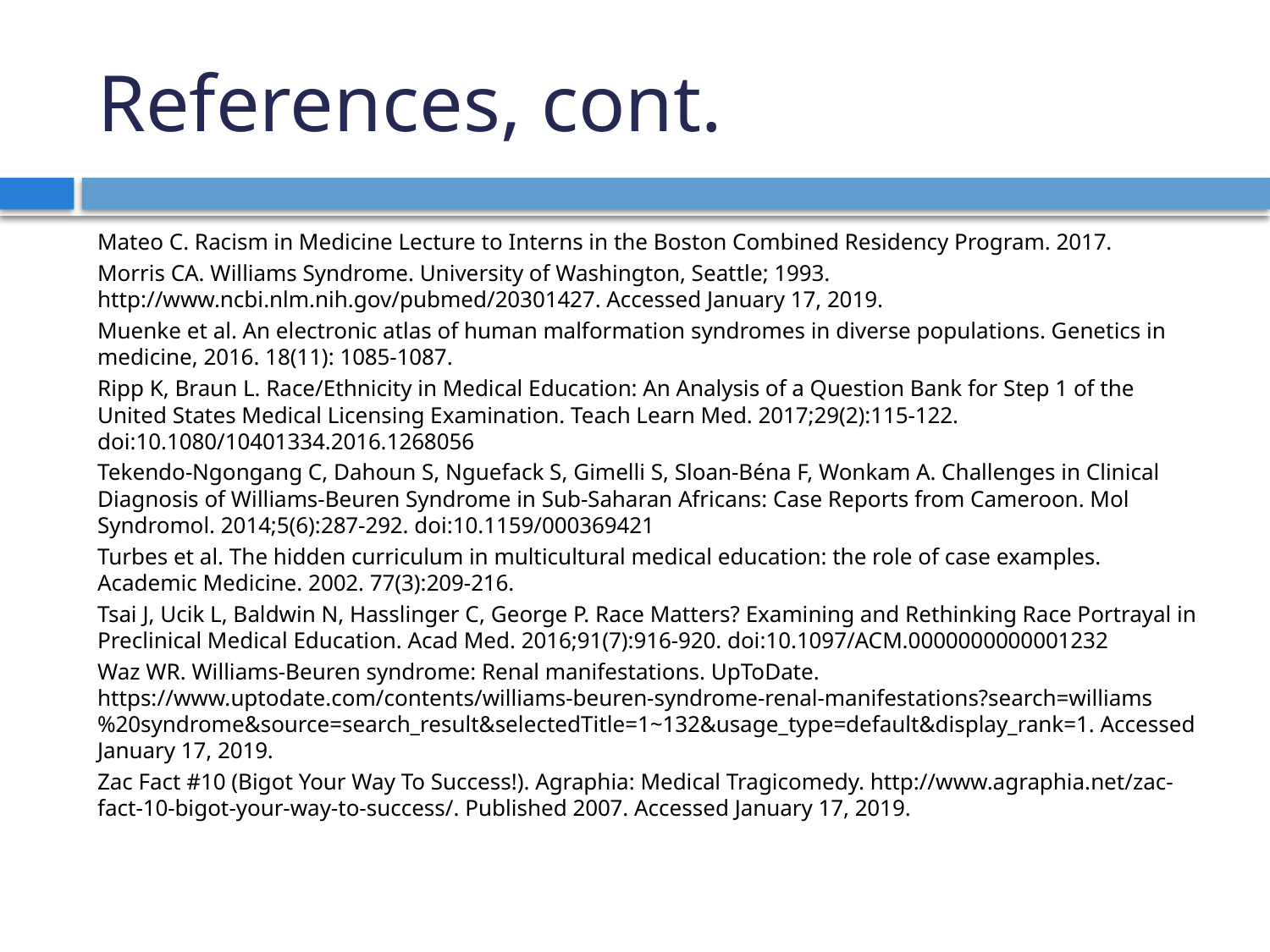

# References, cont.
Mateo C. Racism in Medicine Lecture to Interns in the Boston Combined Residency Program. 2017.
Morris CA. Williams Syndrome. University of Washington, Seattle; 1993. http://www.ncbi.nlm.nih.gov/pubmed/20301427. Accessed January 17, 2019.
Muenke et al. An electronic atlas of human malformation syndromes in diverse populations. Genetics in medicine, 2016. 18(11): 1085-1087.
Ripp K, Braun L. Race/Ethnicity in Medical Education: An Analysis of a Question Bank for Step 1 of the United States Medical Licensing Examination. Teach Learn Med. 2017;29(2):115-122. doi:10.1080/10401334.2016.1268056
Tekendo-Ngongang C, Dahoun S, Nguefack S, Gimelli S, Sloan-Béna F, Wonkam A. Challenges in Clinical Diagnosis of Williams-Beuren Syndrome in Sub-Saharan Africans: Case Reports from Cameroon. Mol Syndromol. 2014;5(6):287-292. doi:10.1159/000369421
Turbes et al. The hidden curriculum in multicultural medical education: the role of case examples. Academic Medicine. 2002. 77(3):209-216.
Tsai J, Ucik L, Baldwin N, Hasslinger C, George P. Race Matters? Examining and Rethinking Race Portrayal in Preclinical Medical Education. Acad Med. 2016;91(7):916-920. doi:10.1097/ACM.0000000000001232
Waz WR. Williams-Beuren syndrome: Renal manifestations. UpToDate. https://www.uptodate.com/contents/williams-beuren-syndrome-renal-manifestations?search=williams%20syndrome&source=search_result&selectedTitle=1~132&usage_type=default&display_rank=1. Accessed January 17, 2019.
Zac Fact #10 (Bigot Your Way To Success!). Agraphia: Medical Tragicomedy. http://www.agraphia.net/zac-fact-10-bigot-your-way-to-success/. Published 2007. Accessed January 17, 2019.
