# Supplementary material for: Health Equity Rounds: An Interdisciplinary Case Conference to Address Implicit Bias and Structural Racism for Faculty and Trainees
Source: MedEdPORTAL. 2019 Nov 22;15:10858. doi: 10.15766/mep_2374-8265.10858 (PMC7050660; doi:10.15766/mep_2374-8265.10858)
Supplement: Supplementary file 1 — A. HER 1.pptx B. HER 2.pptx C. HER 3.pptx D. HER 4.pptx E. HER 5.pptx F. HER 6.pptx G. HER 7.pptx H. Selected HER Handouts.docx I. Case Conference Creation Guide.docx J. Glossary.docx K. Evaluation.docx [file mep-15-10858-s001.zip › F. HER 6.pptx]

## Slide 1
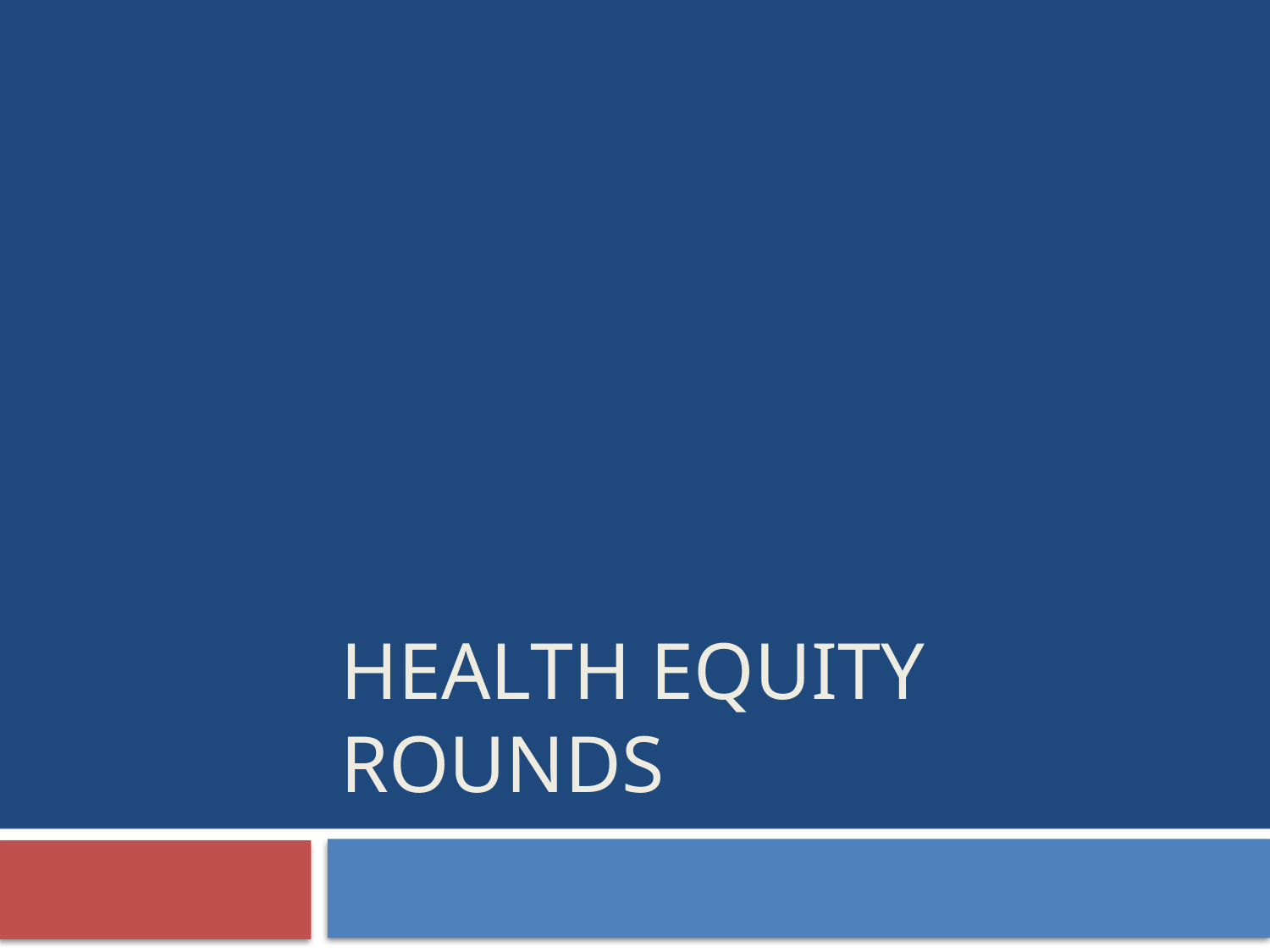

# Health equity rounds

## Slide 2
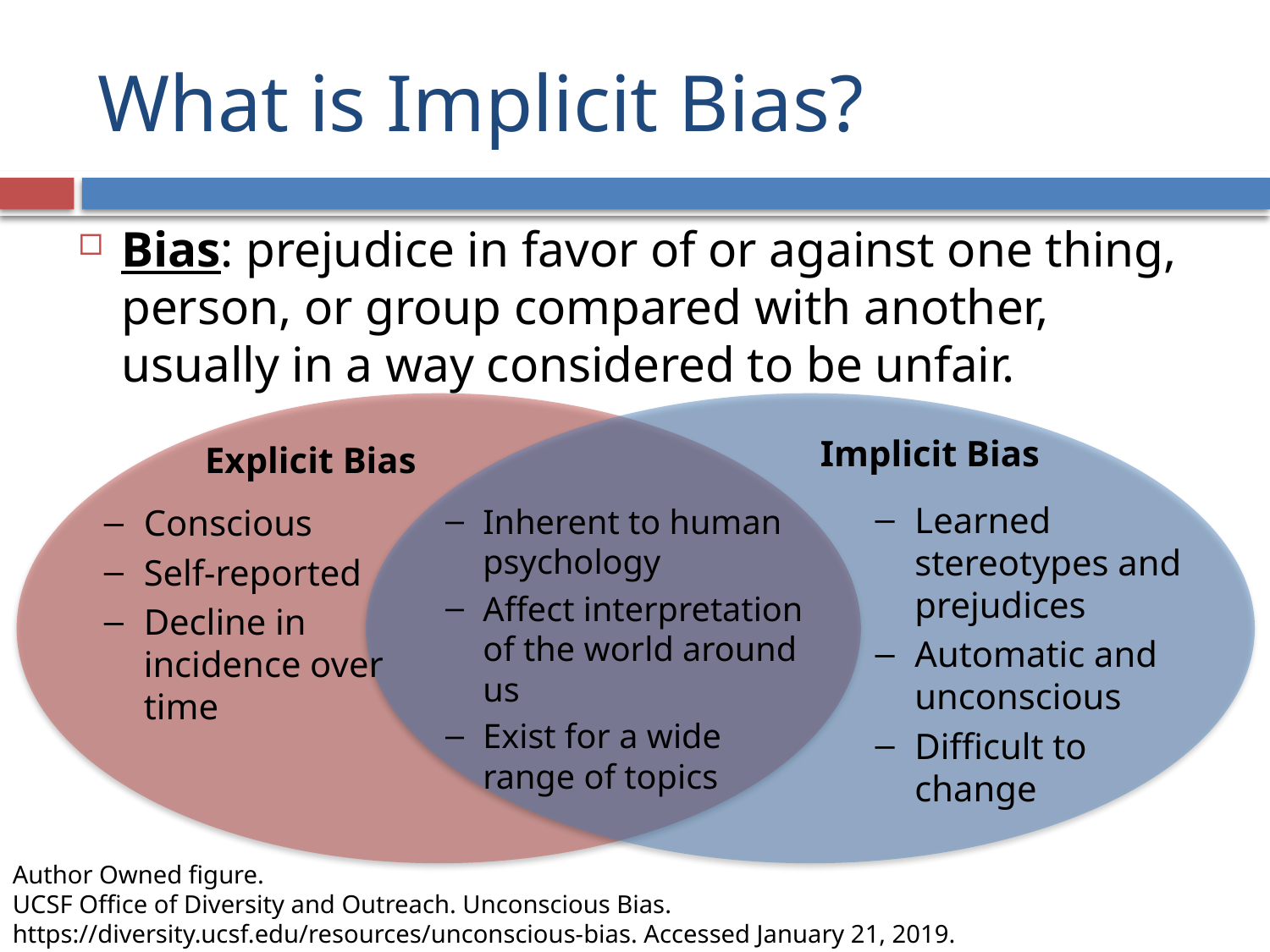

# What is Implicit Bias?
Bias: prejudice in favor of or against one thing, person, or group compared with another, usually in a way considered to be unfair.
Implicit Bias
Explicit Bias
Learned stereotypes and prejudices
Automatic and unconscious
Difficult to change
Inherent to human psychology
Affect interpretation of the world around us
Exist for a wide range of topics
Conscious
Self-reported
Decline in incidence over time
Author Owned figure.
UCSF Office of Diversity and Outreach. Unconscious Bias. https://diversity.ucsf.edu/resources/unconscious-bias. Accessed January 21, 2019.

## Slide 3
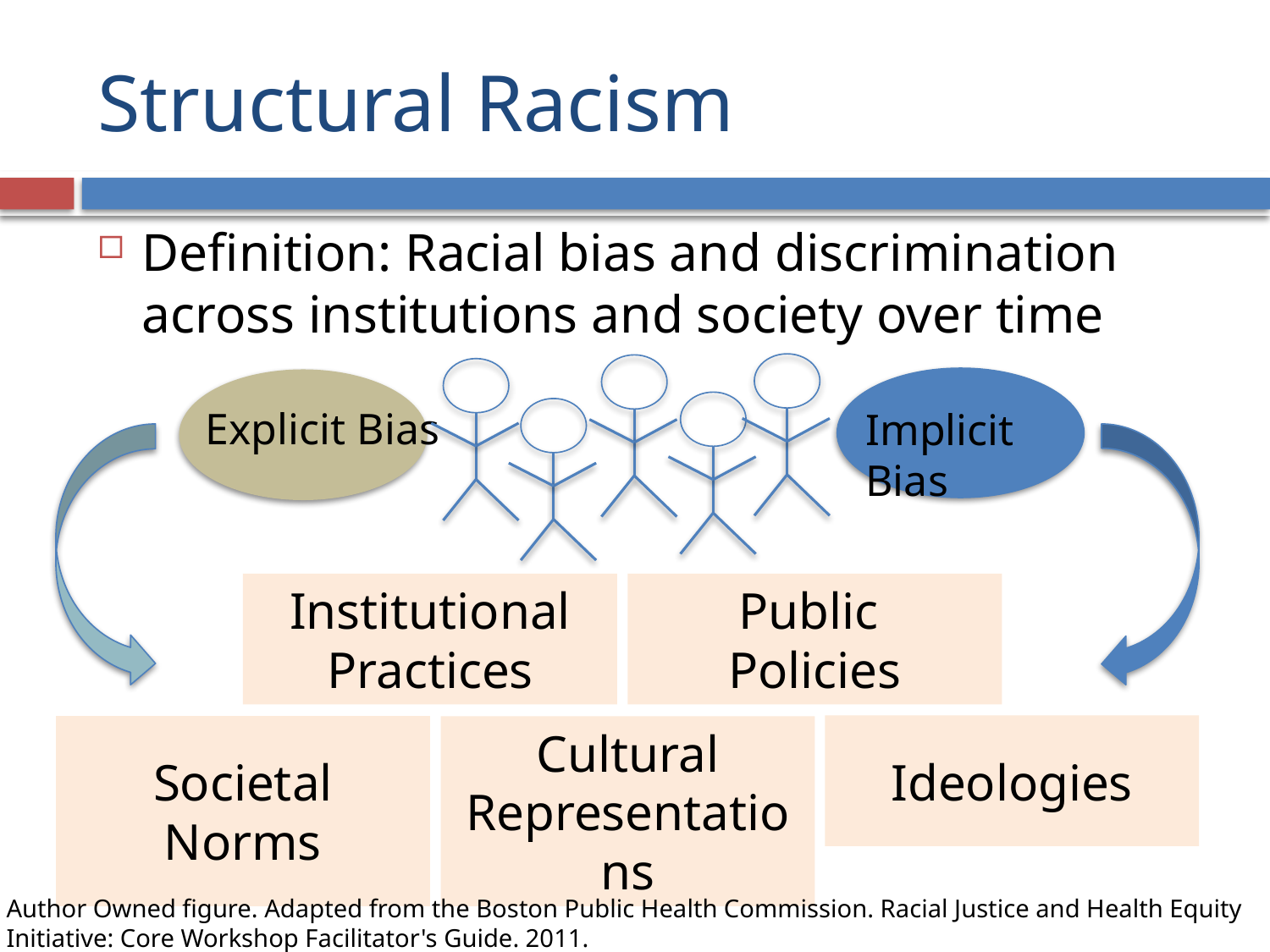

# Structural Racism
Definition: Racial bias and discrimination across institutions and society over time
Explicit Bias
Implicit Bias
Institutional Practices
Public
Policies
Ideologies
Societal Norms
Cultural
Representations
Author Owned figure. Adapted from the Boston Public Health Commission. Racial Justice and Health Equity Initiative: Core Workshop Facilitator's Guide. 2011.

## Slide 4
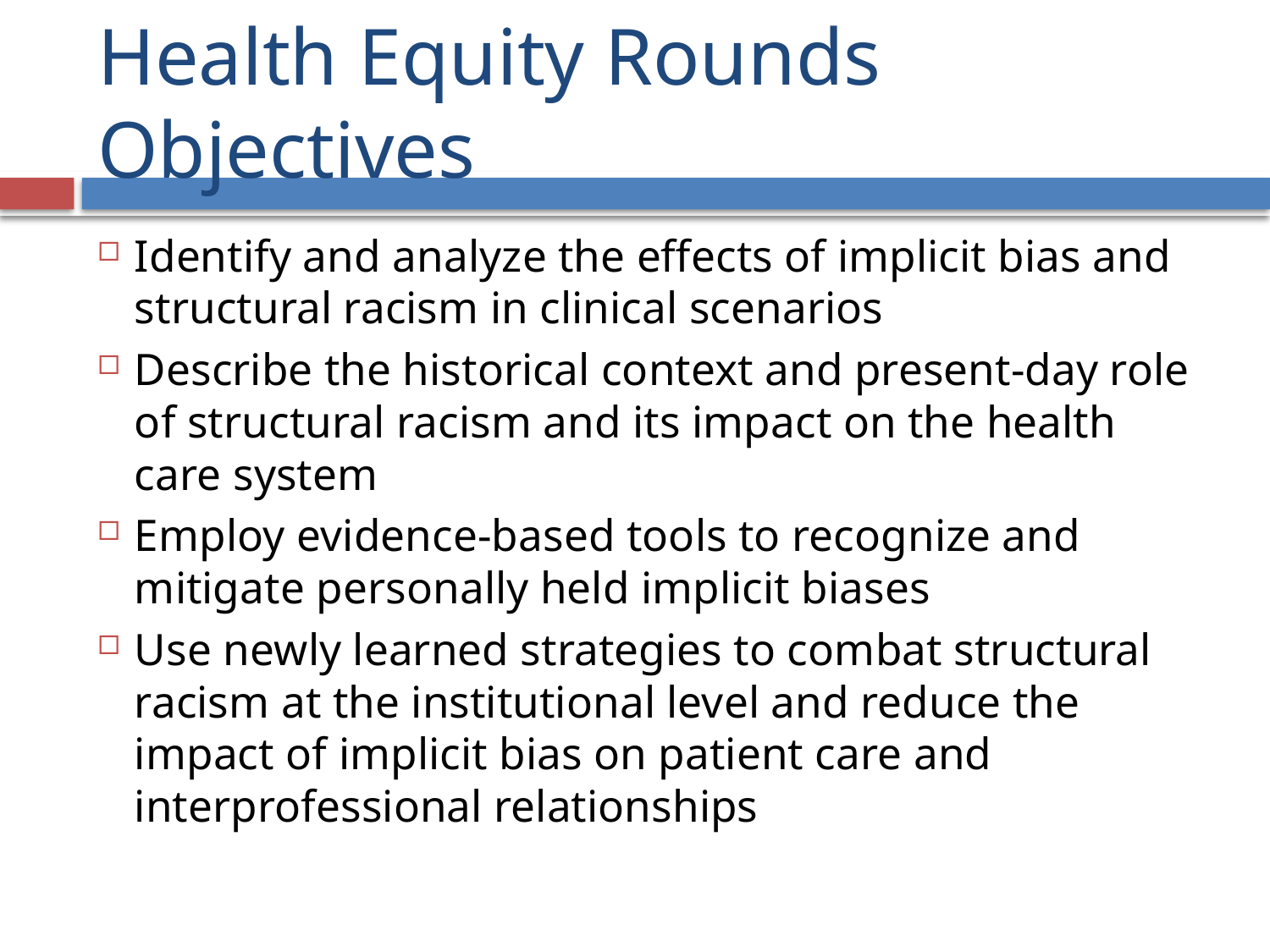

# Health Equity Rounds Objectives
Identify and analyze the effects of implicit bias and structural racism in clinical scenarios
Describe the historical context and present-day role of structural racism and its impact on the health care system
Employ evidence-based tools to recognize and mitigate personally held implicit biases
Use newly learned strategies to combat structural racism at the institutional level and reduce the impact of implicit bias on patient care and interprofessional relationships

## Slide 5
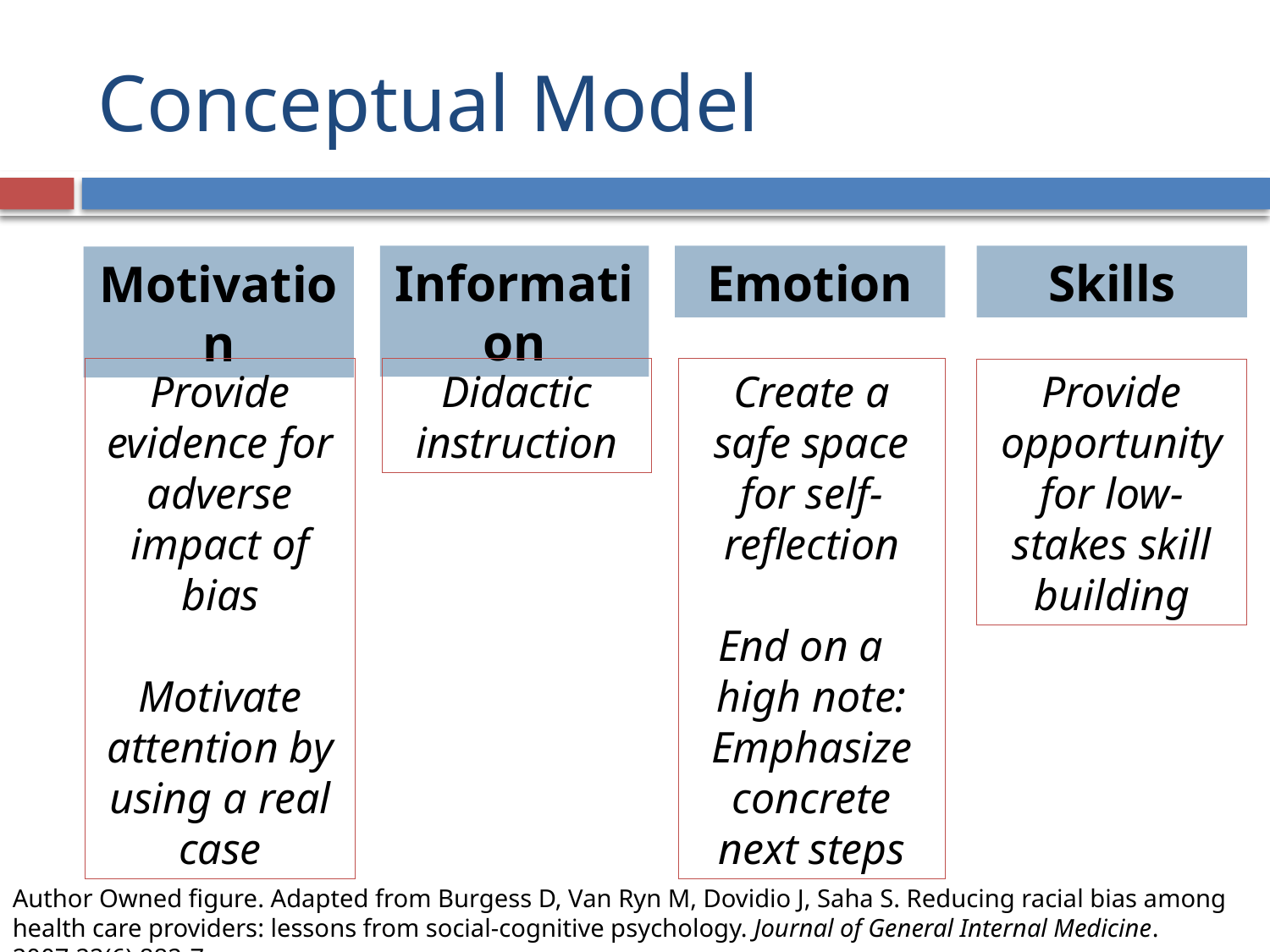

# Conceptual Model
Skills
Information
Emotion
Motivation
Create a safe space for self-reflection
d
End on a high note: Emphasize concrete next steps
Provide evidence for adverse impact of bias
Motivate attention by using a real case
Didactic instruction
Provide opportunity for low-stakes skill building
Author Owned figure. Adapted from Burgess D, Van Ryn M, Dovidio J, Saha S. Reducing racial bias among health care providers: lessons from social-cognitive psychology. Journal of General Internal Medicine. 2007;22(6):882-7.

## Slide 6
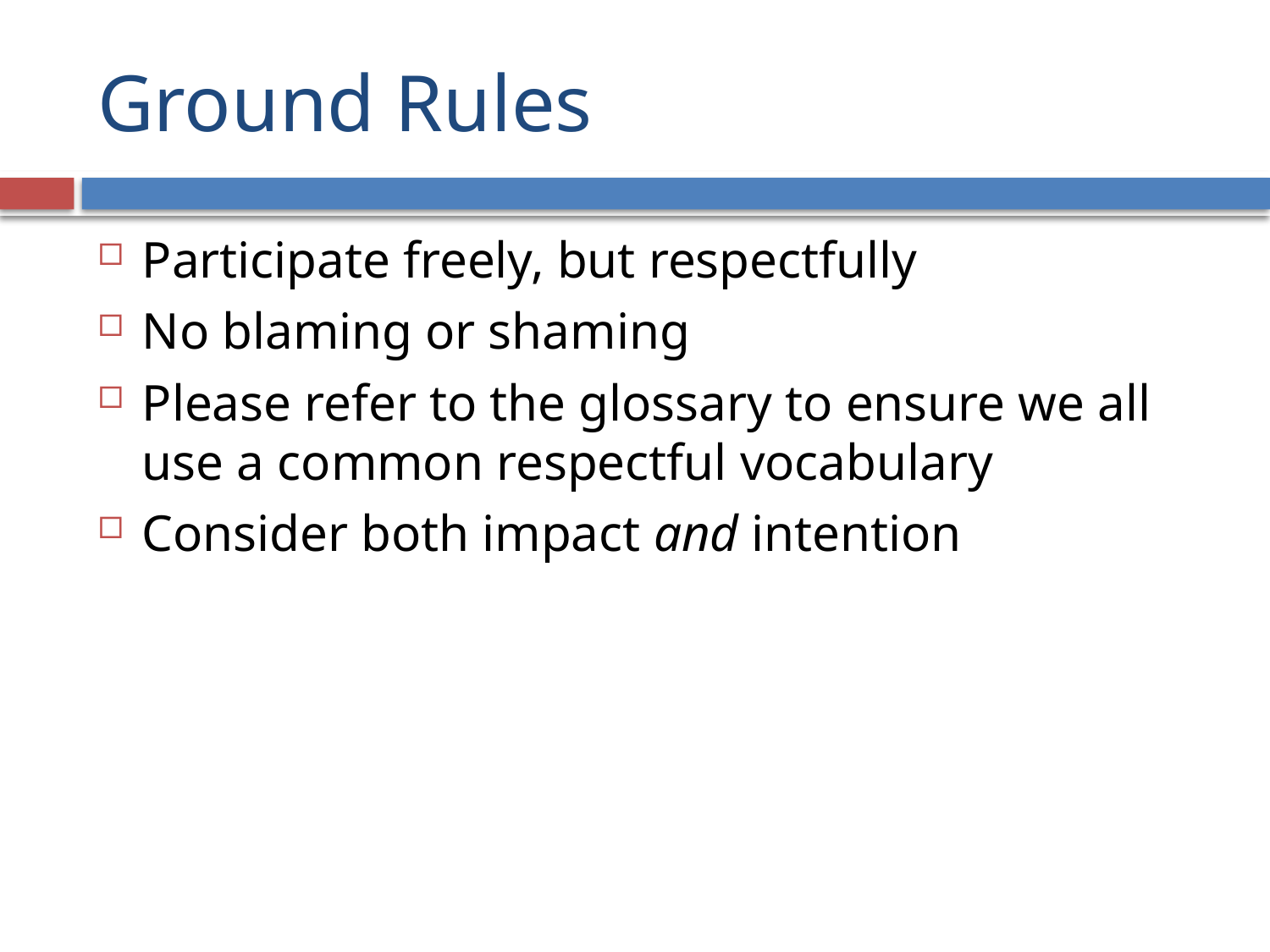

# Ground Rules
Participate freely, but respectfully
No blaming or shaming
Please refer to the glossary to ensure we all use a common respectful vocabulary
Consider both impact and intention

## Slide 7
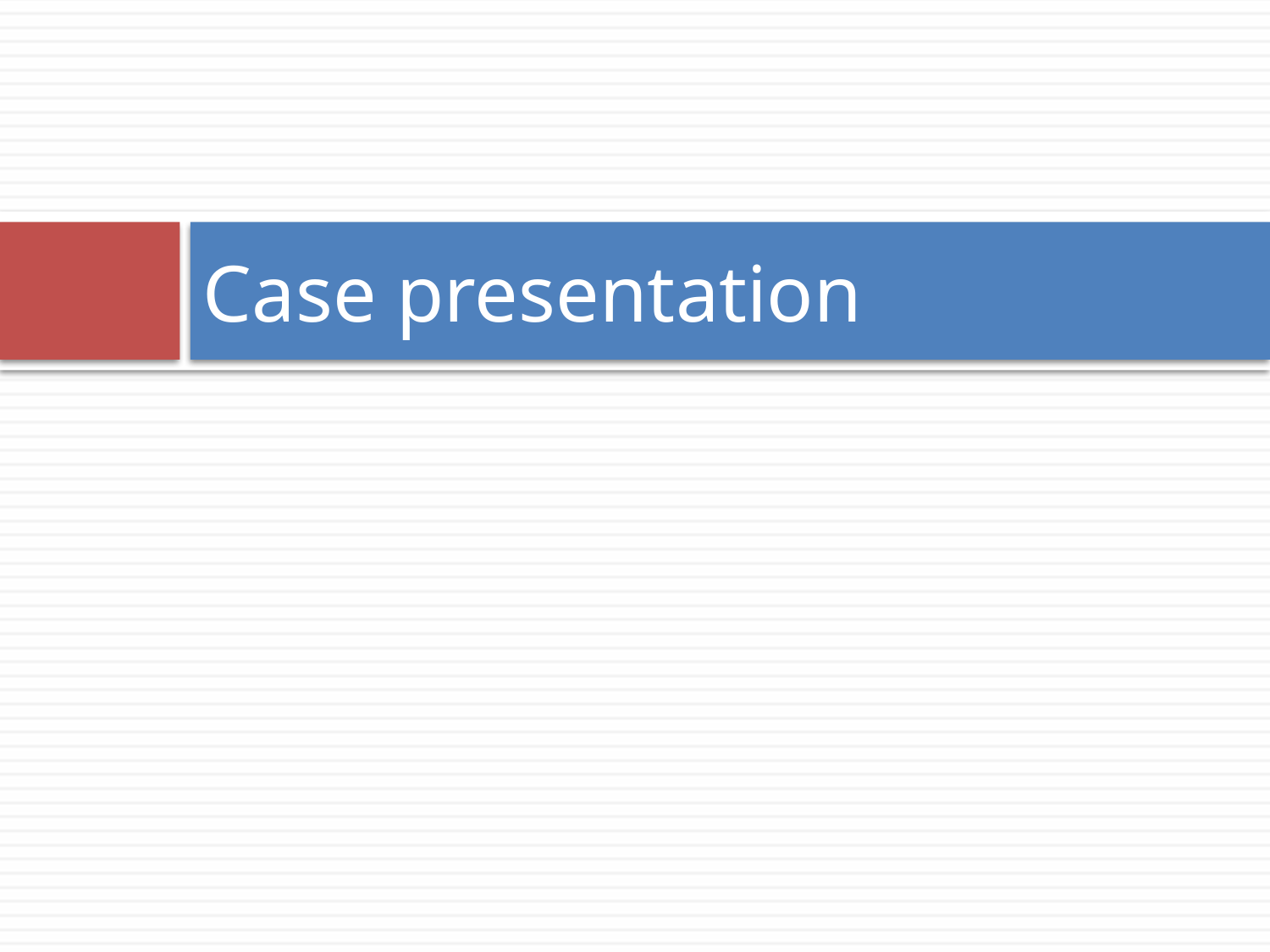

# Case presentation

## Slide 8
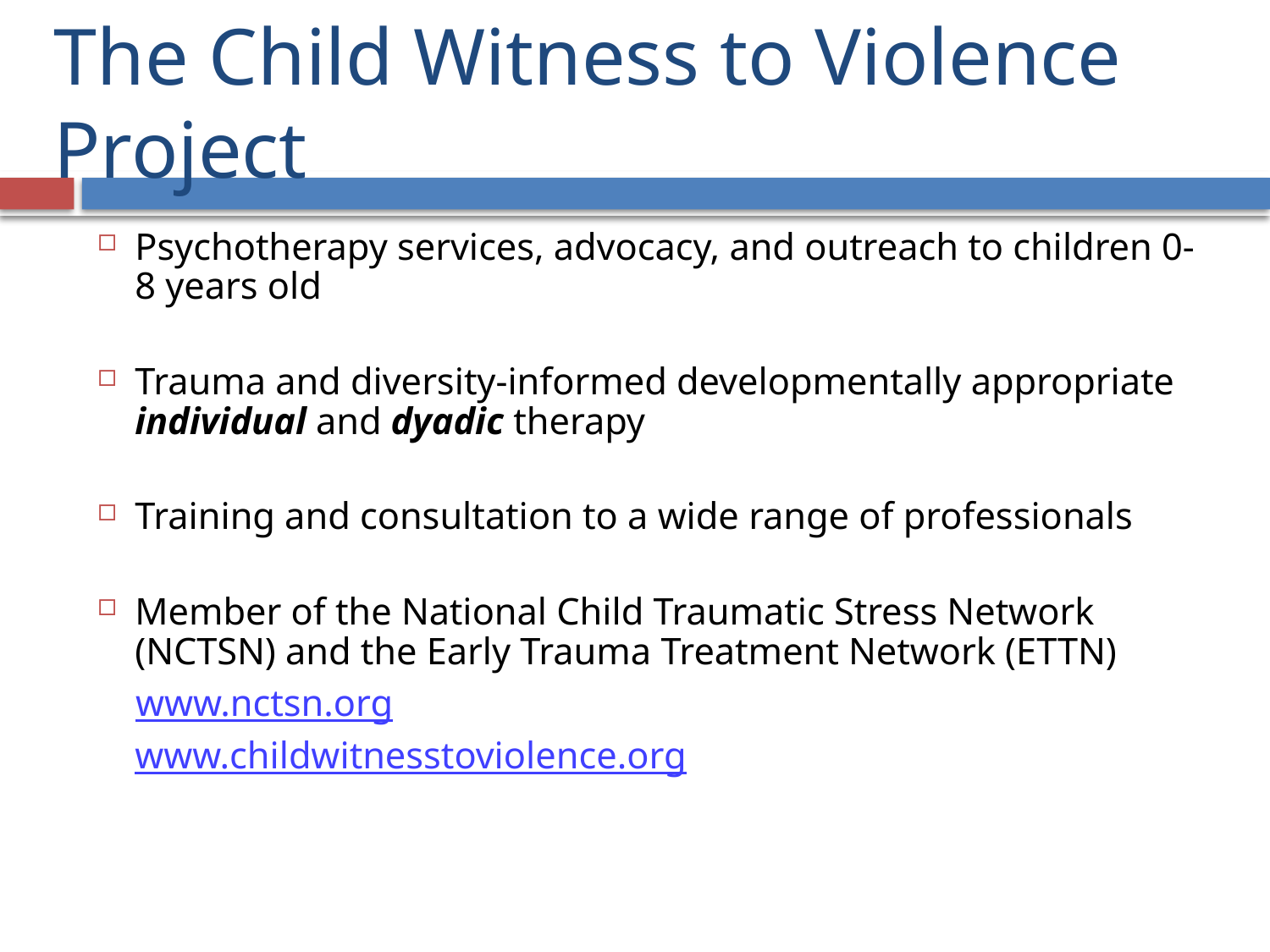

# The Child Witness to Violence Project
Psychotherapy services, advocacy, and outreach to children 0-8 years old
Trauma and diversity-informed developmentally appropriate individual and dyadic therapy
Training and consultation to a wide range of professionals
Member of the National Child Traumatic Stress Network (NCTSN) and the Early Trauma Treatment Network (ETTN)
 www.nctsn.org
	www.childwitnesstoviolence.org

## Slide 9
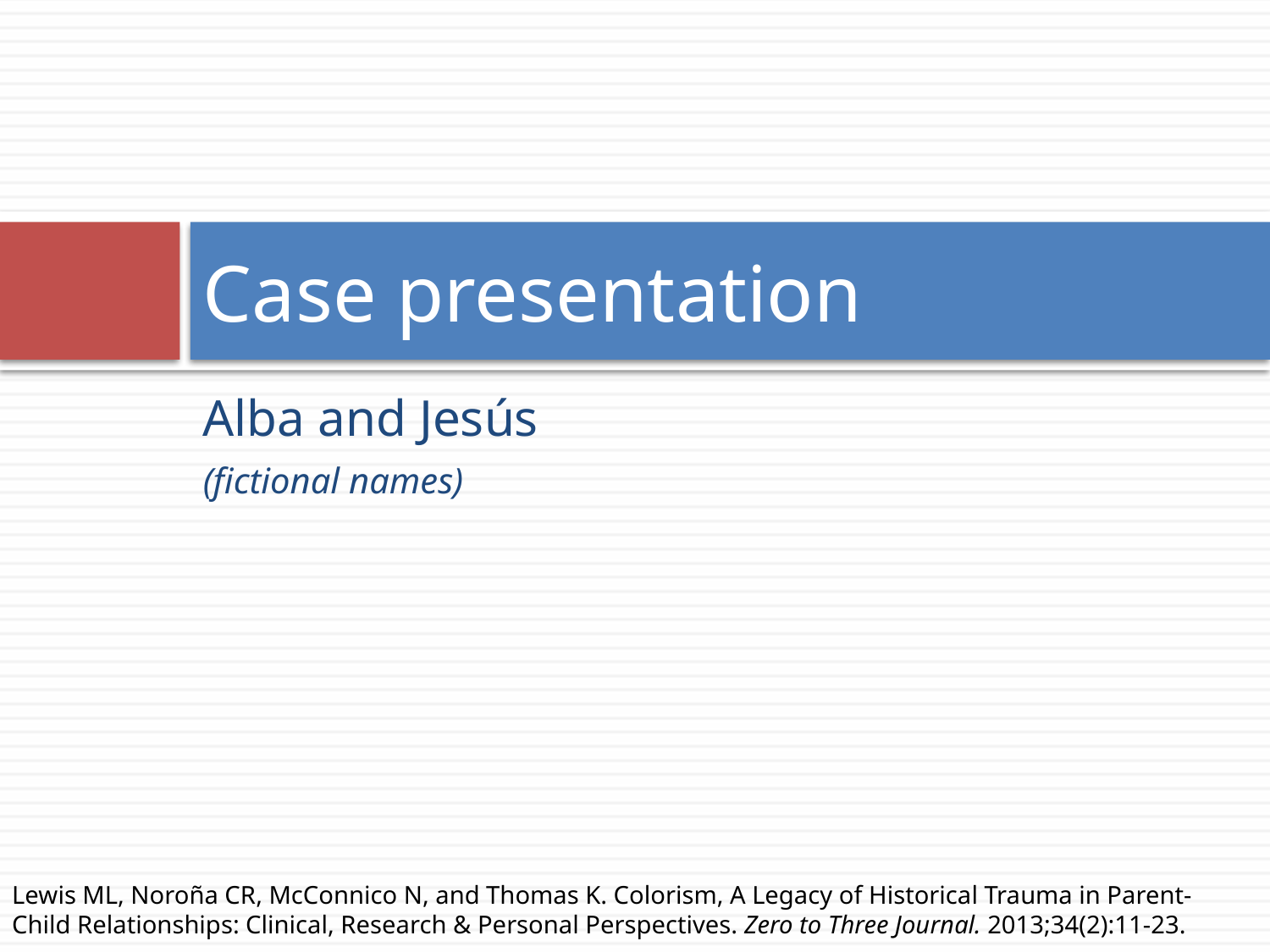

# Case presentation
Alba and Jesús
(fictional names)
Lewis ML, Noroña CR, McConnico N, and Thomas K. Colorism, A Legacy of Historical Trauma in Parent-Child Relationships: Clinical, Research & Personal Perspectives. Zero to Three Journal. 2013;34(2):11-23.

## Slide 10
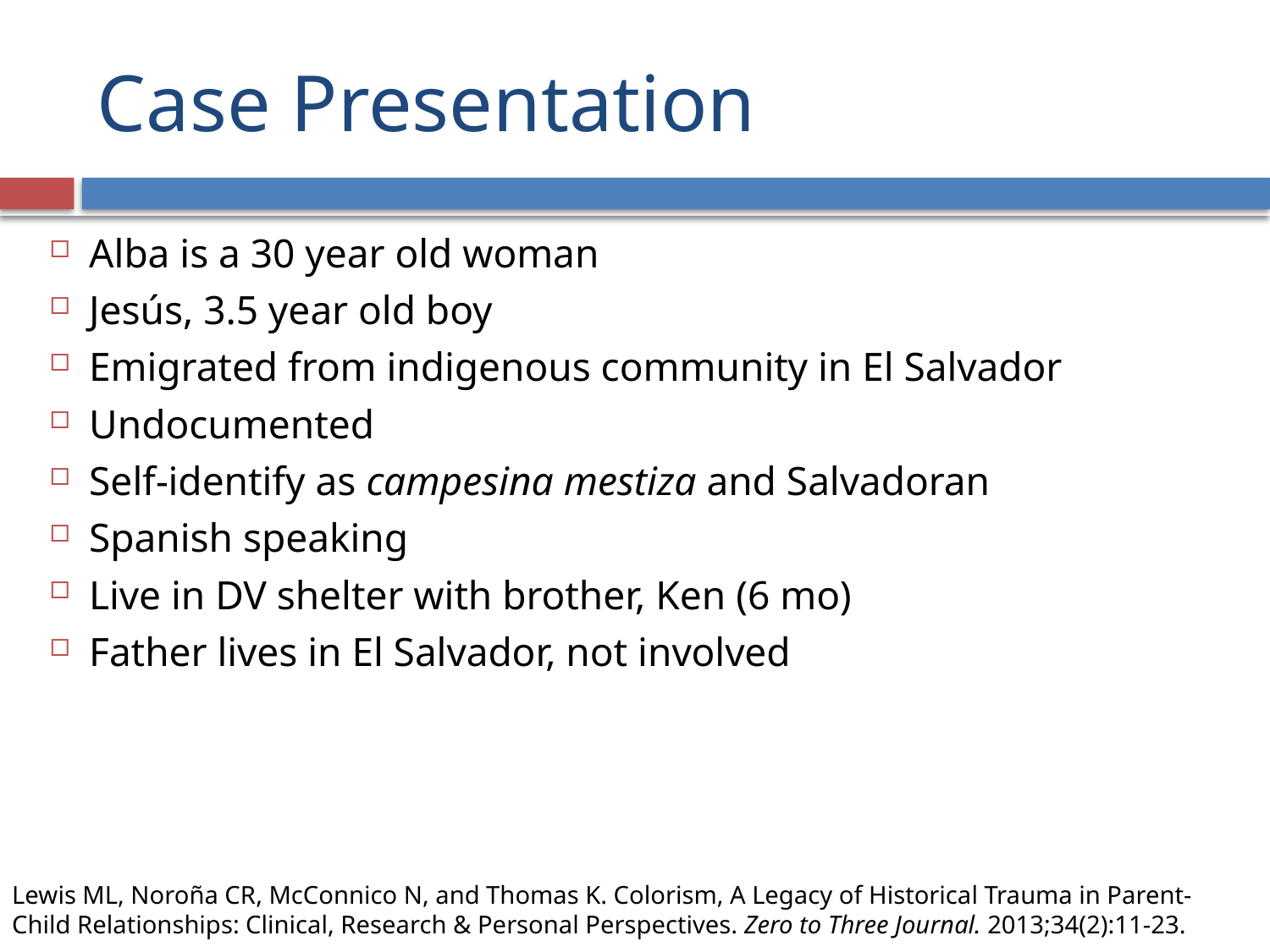

# Case Presentation
Alba is a 30 year old woman
Jesús, 3.5 year old boy
Emigrated from indigenous community in El Salvador
Undocumented
Self-identify as campesina mestiza and Salvadoran
Spanish speaking
Live in DV shelter with brother, Ken (6 mo)
Father lives in El Salvador, not involved
Lewis ML, Noroña CR, McConnico N, and Thomas K. Colorism, A Legacy of Historical Trauma in Parent-Child Relationships: Clinical, Research & Personal Perspectives. Zero to Three Journal. 2013;34(2):11-23.

## Slide 11
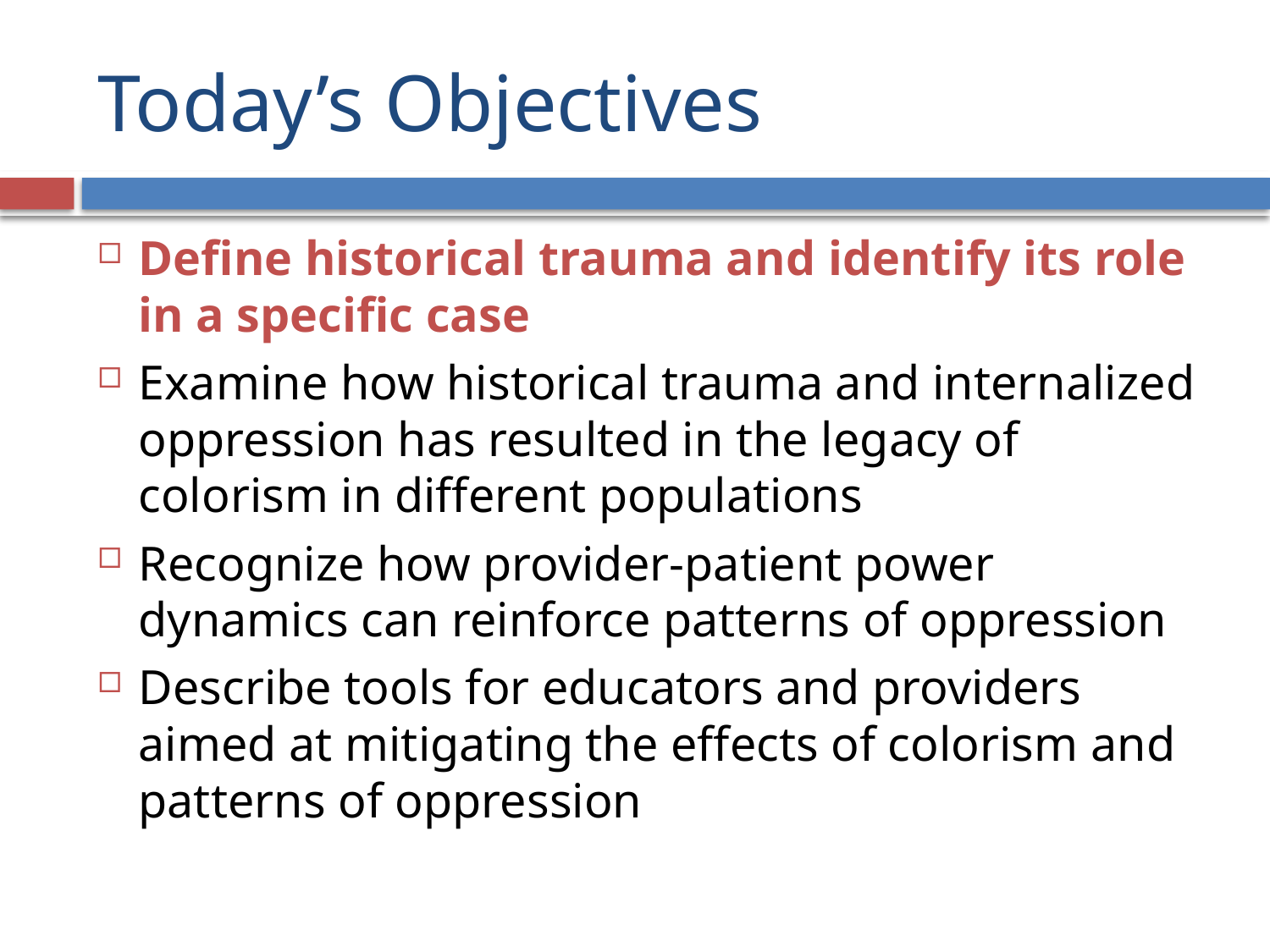

# Today’s Objectives
Define historical trauma and identify its role in a specific case
Examine how historical trauma and internalized oppression has resulted in the legacy of colorism in different populations
Recognize how provider-patient power dynamics can reinforce patterns of oppression
Describe tools for educators and providers aimed at mitigating the effects of colorism and patterns of oppression

## Slide 12
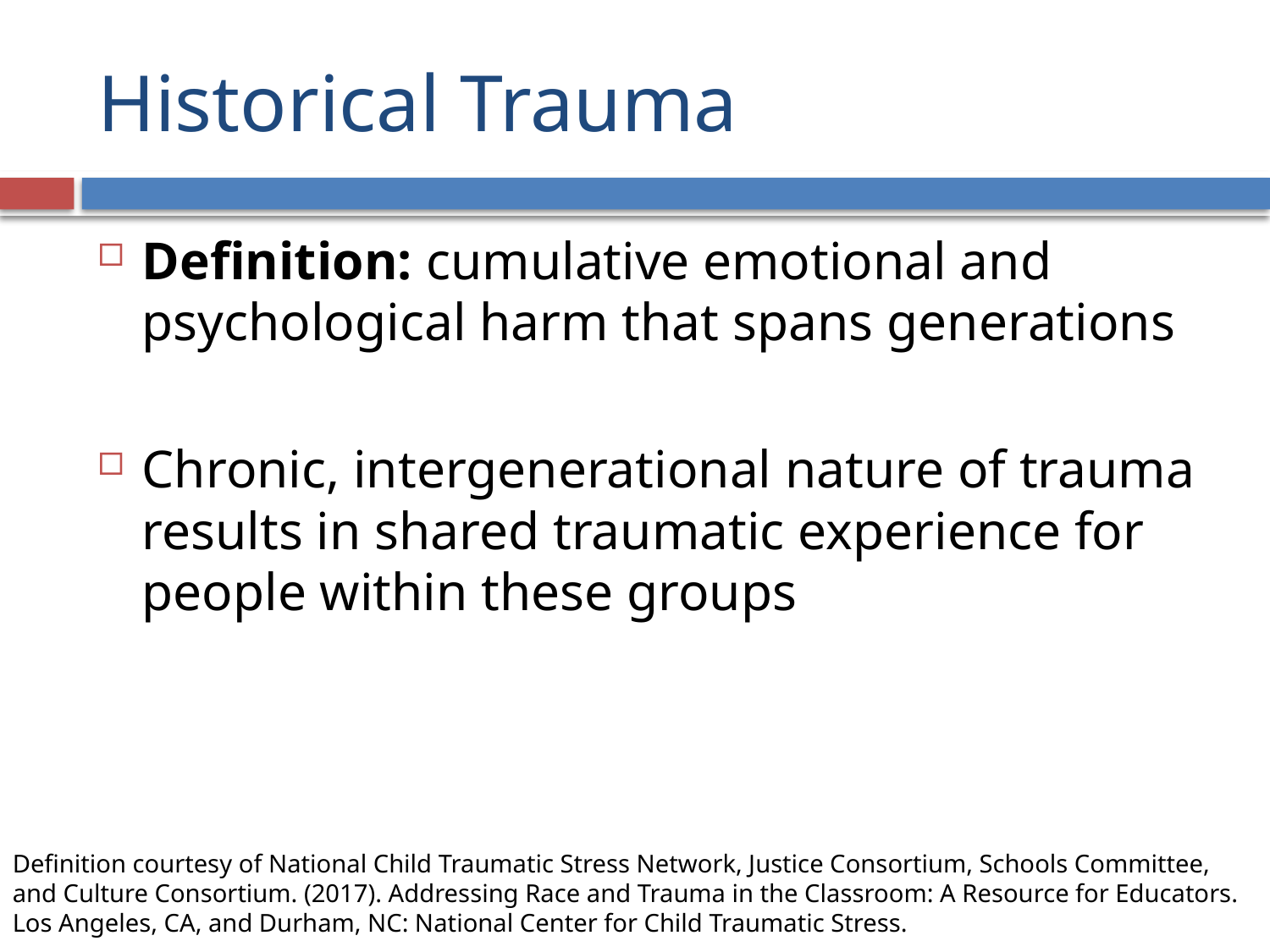

# Historical Trauma
Definition: cumulative emotional and psychological harm that spans generations
Chronic, intergenerational nature of trauma results in shared traumatic experience for people within these groups
Definition courtesy of National Child Traumatic Stress Network, Justice Consortium, Schools Committee, and Culture Consortium. (2017). Addressing Race and Trauma in the Classroom: A Resource for Educators. Los Angeles, CA, and Durham, NC: National Center for Child Traumatic Stress.

## Slide 13
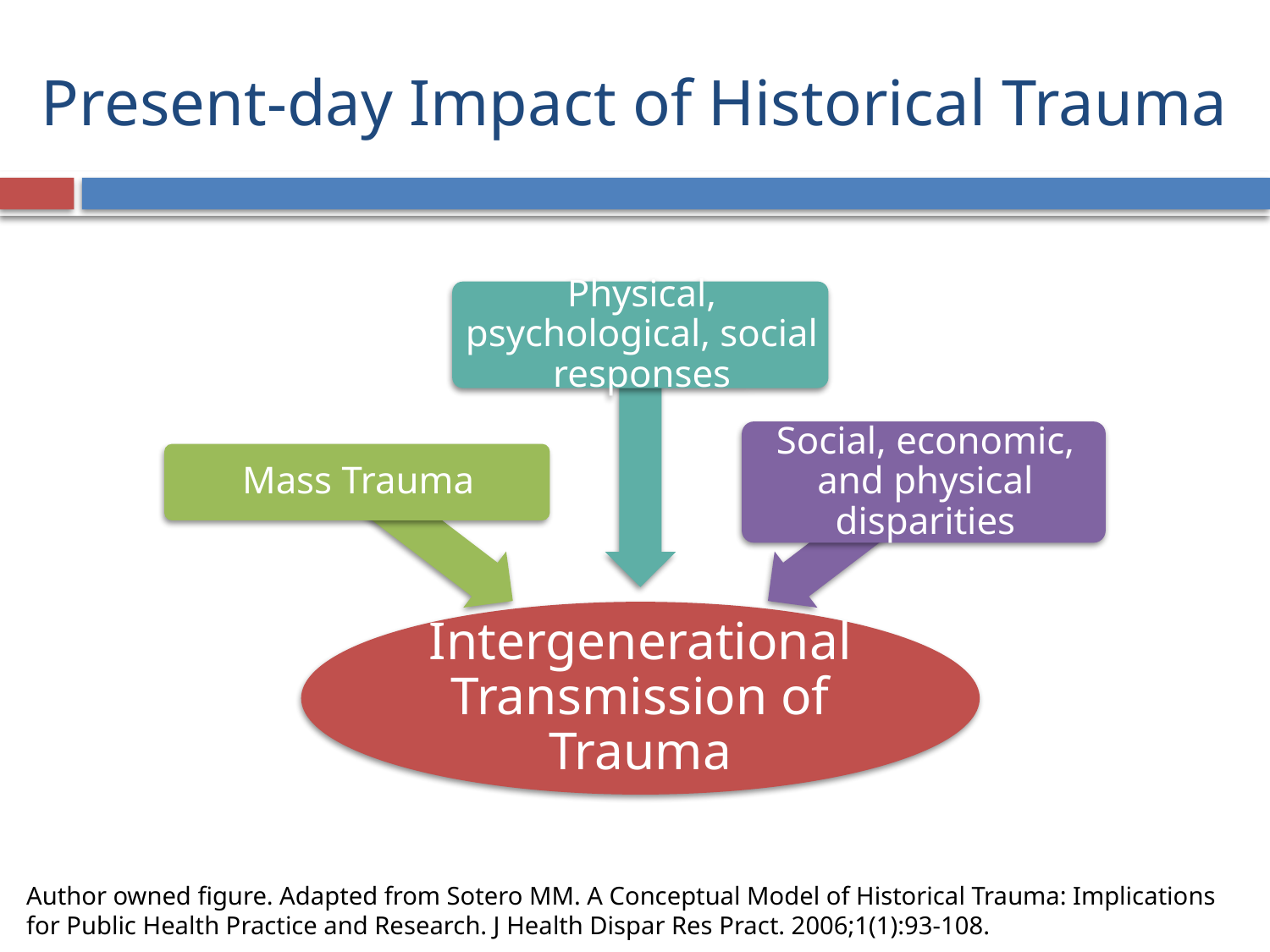

# Present-day Impact of Historical Trauma
Author owned figure. Adapted from Sotero MM. A Conceptual Model of Historical Trauma: Implications for Public Health Practice and Research. J Health Dispar Res Pract. 2006;1(1):93-108.

## Slide 14
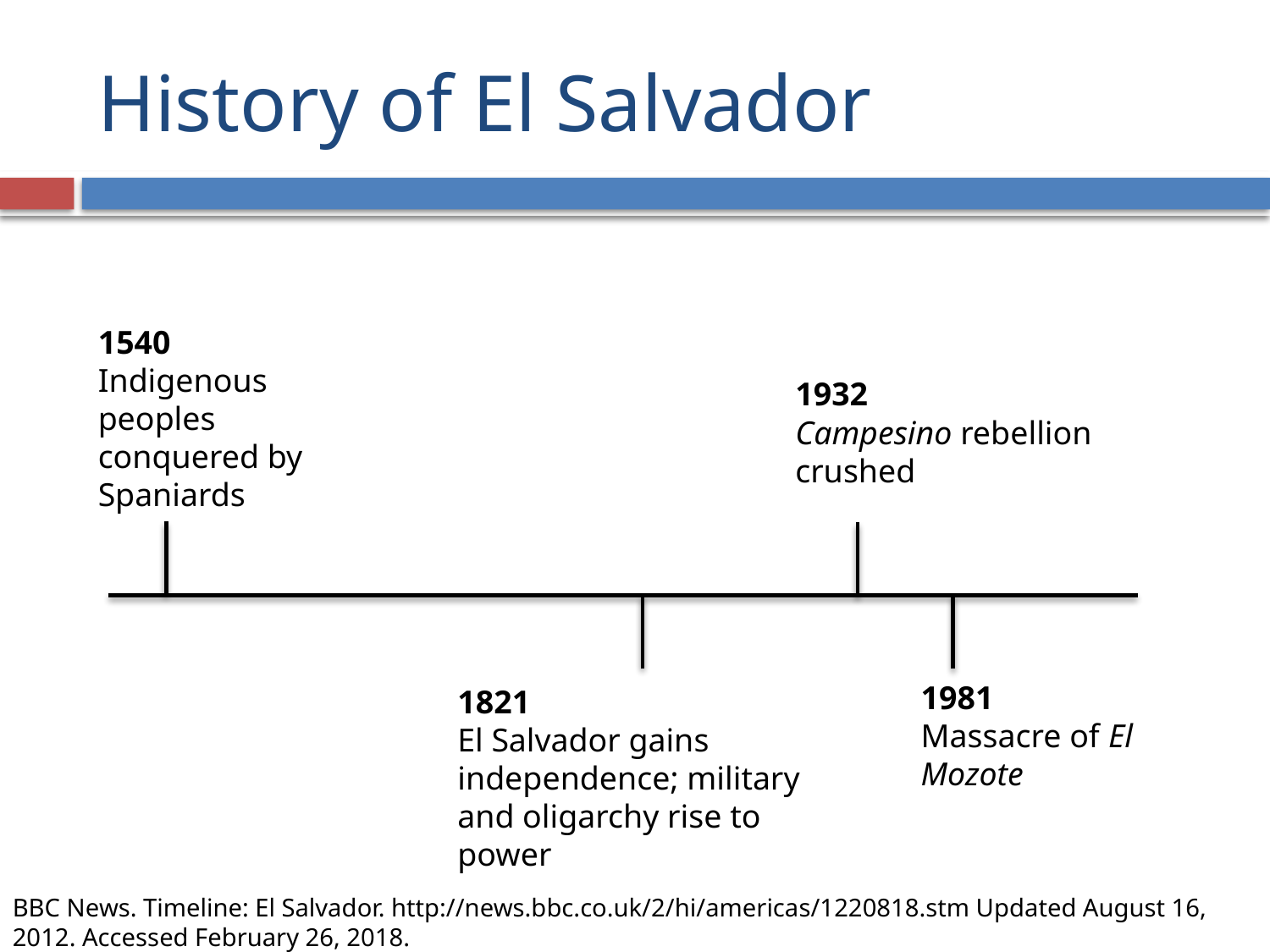

# History of El Salvador
1540
Indigenous peoples conquered by Spaniards
1932
Campesino rebellion crushed
1981
Massacre of El Mozote
1821
El Salvador gains independence; military and oligarchy rise to power
BBC News. Timeline: El Salvador. http://news.bbc.co.uk/2/hi/americas/1220818.stm Updated August 16, 2012. Accessed February 26, 2018.

## Slide 15
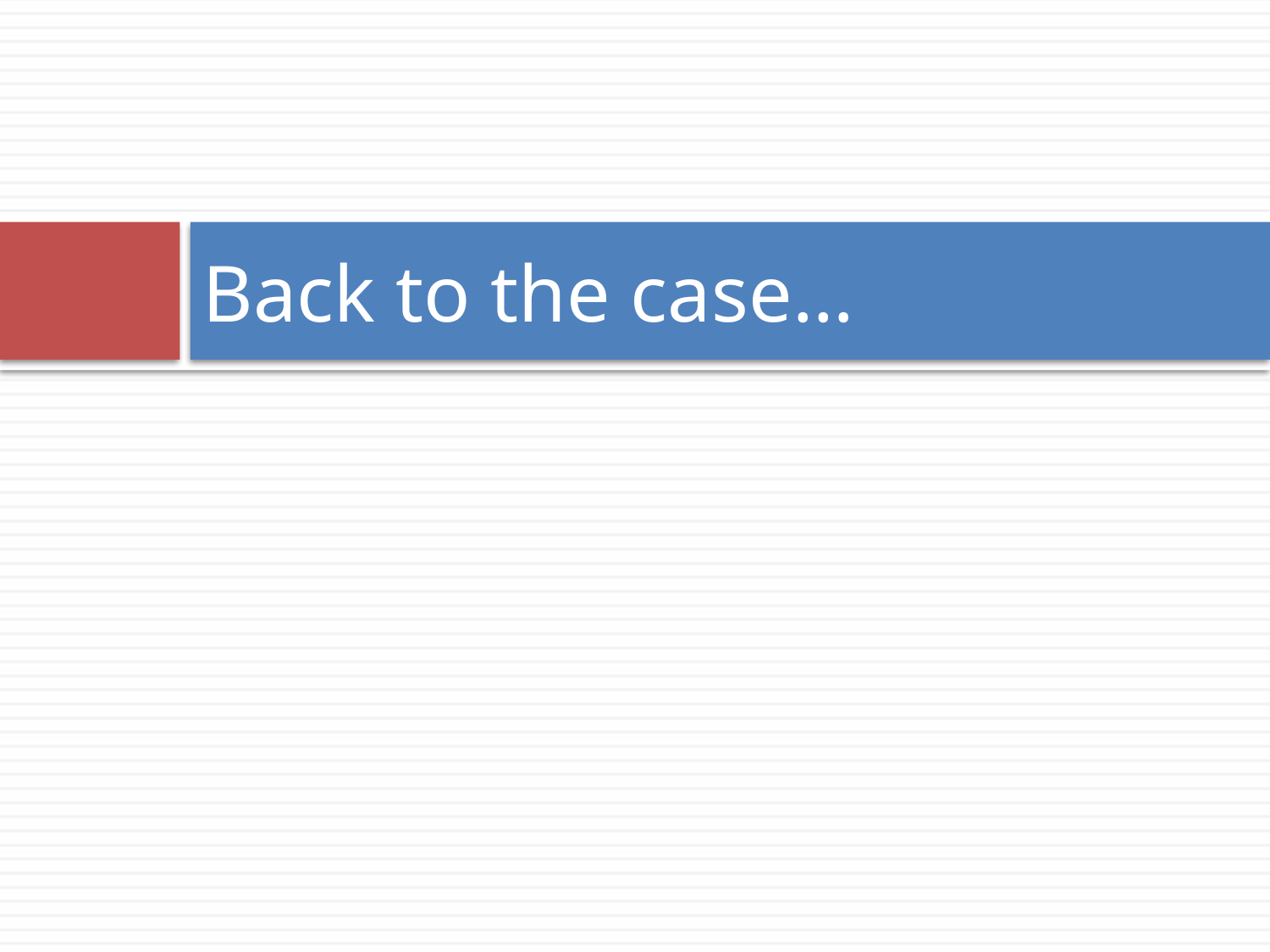

# Back to the case…

## Slide 16
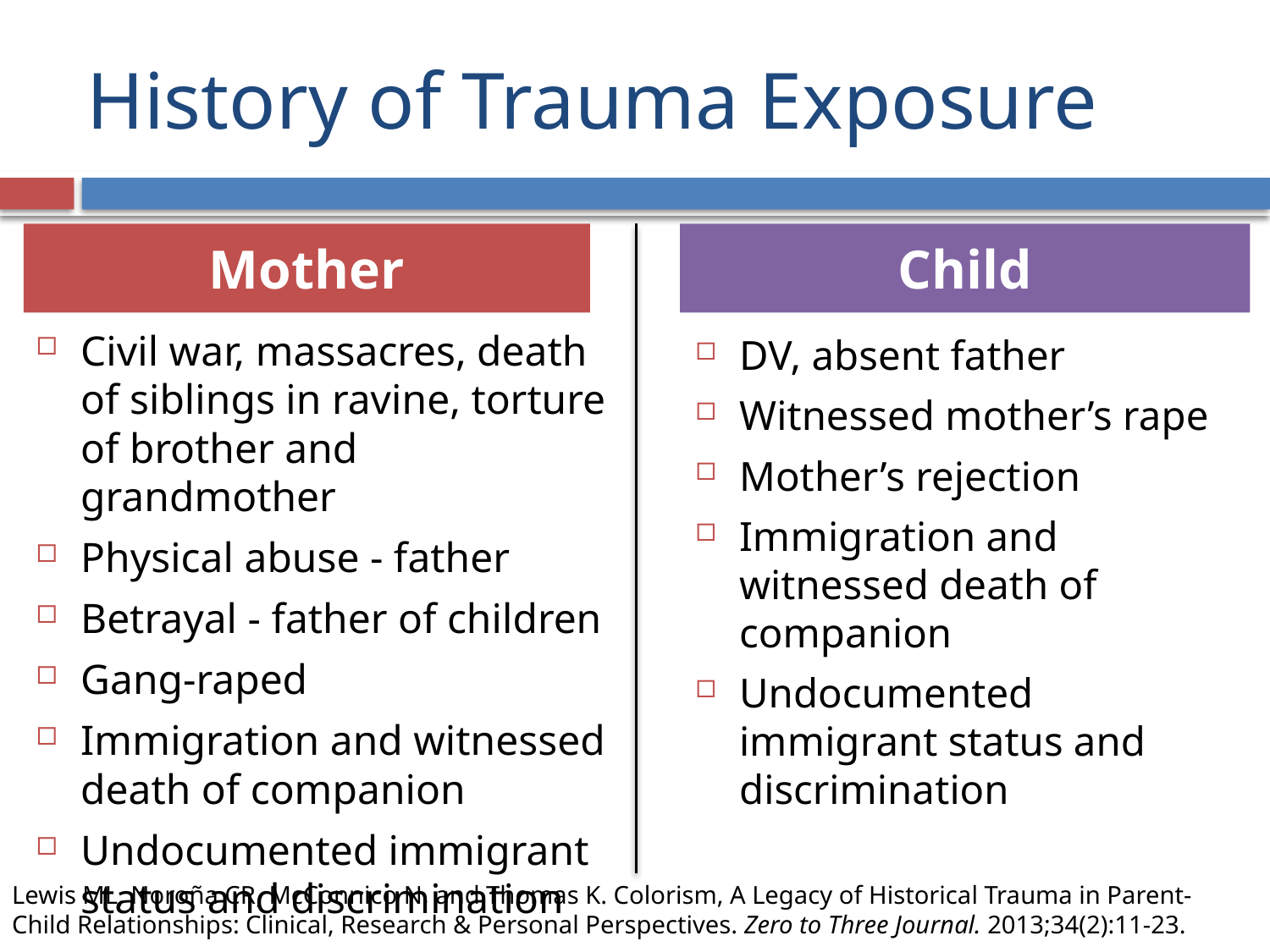

# History of Trauma Exposure
Mother
Child
Civil war, massacres, death of siblings in ravine, torture of brother and grandmother
Physical abuse - father
Betrayal - father of children
Gang-raped
Immigration and witnessed death of companion
Undocumented immigrant status and discrimination
DV, absent father
Witnessed mother’s rape
Mother’s rejection
Immigration and witnessed death of companion
Undocumented immigrant status and discrimination
Lewis ML, Noroña CR, McConnico N, and Thomas K. Colorism, A Legacy of Historical Trauma in Parent-Child Relationships: Clinical, Research & Personal Perspectives. Zero to Three Journal. 2013;34(2):11-23.

## Slide 17
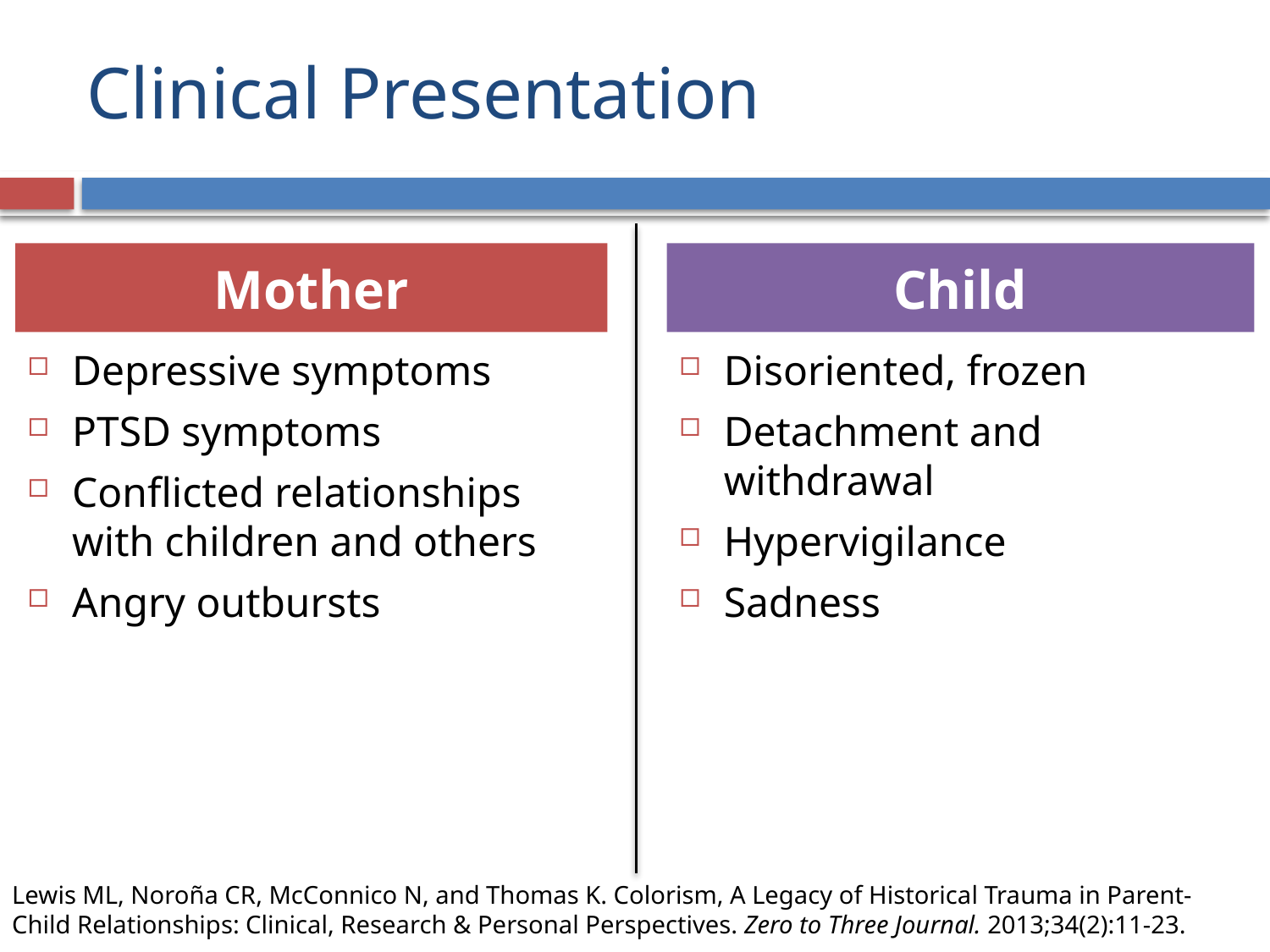

# Clinical Presentation
Mother
Child
Depressive symptoms
PTSD symptoms
Conflicted relationships with children and others
Angry outbursts
Disoriented, frozen
Detachment and withdrawal
Hypervigilance
Sadness
Lewis ML, Noroña CR, McConnico N, and Thomas K. Colorism, A Legacy of Historical Trauma in Parent-Child Relationships: Clinical, Research & Personal Perspectives. Zero to Three Journal. 2013;34(2):11-23.

## Slide 18
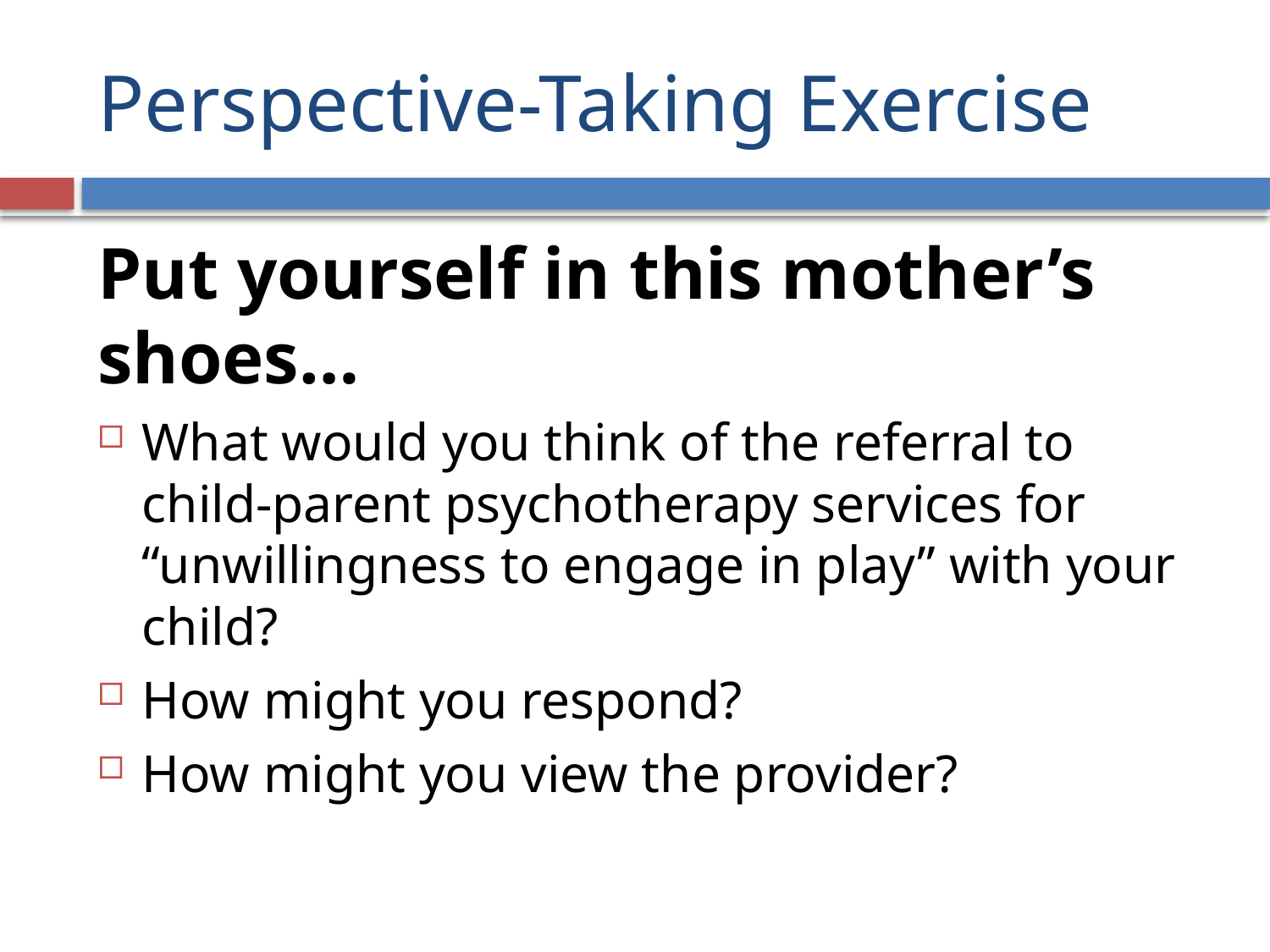

# Perspective-Taking Exercise
Put yourself in this mother’s shoes…
What would you think of the referral to child-parent psychotherapy services for “unwillingness to engage in play” with your child?
How might you respond?
How might you view the provider?

## Slide 19
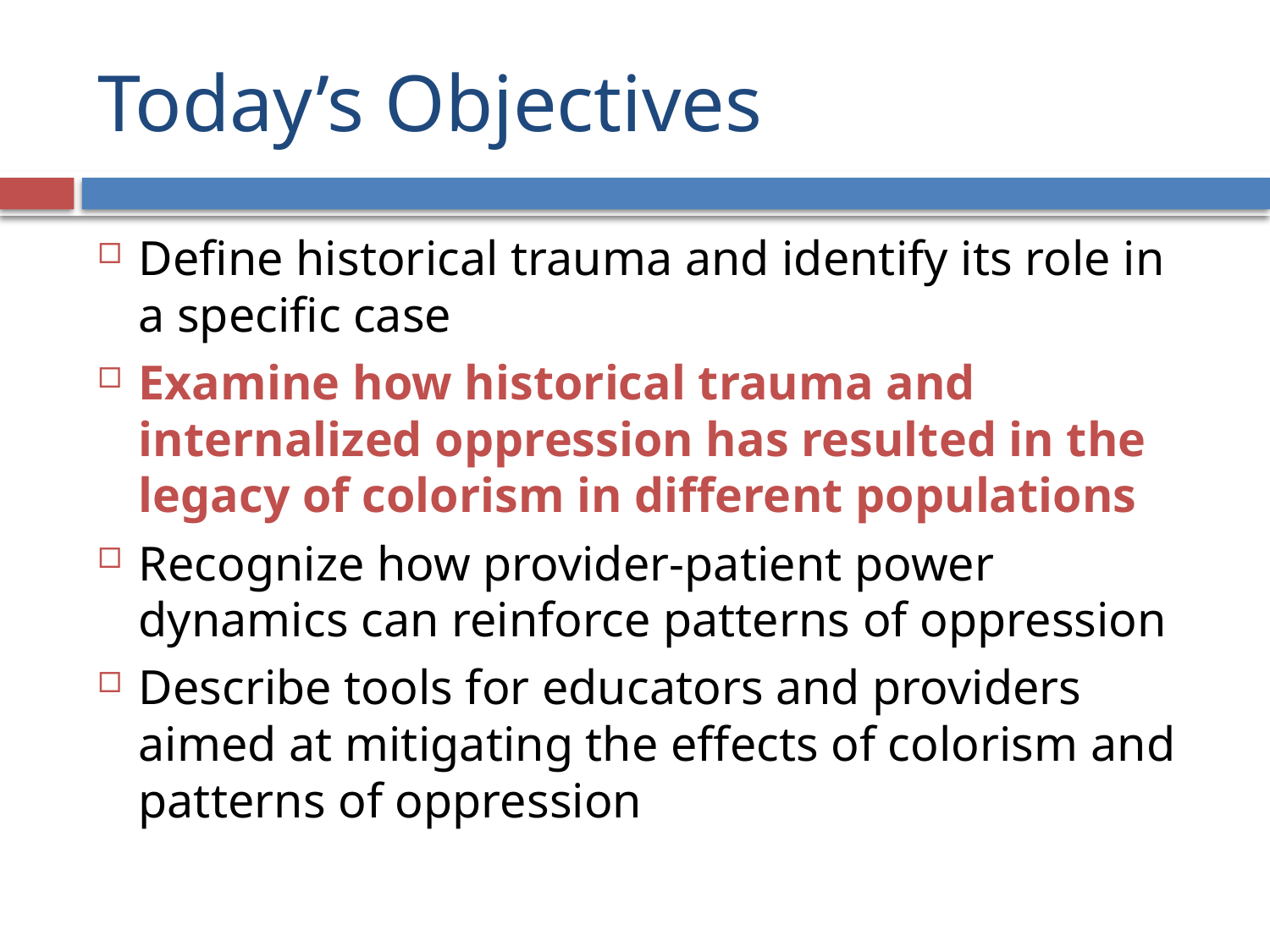

# Today’s Objectives
Define historical trauma and identify its role in a specific case
Examine how historical trauma and internalized oppression has resulted in the legacy of colorism in different populations
Recognize how provider-patient power dynamics can reinforce patterns of oppression
Describe tools for educators and providers aimed at mitigating the effects of colorism and patterns of oppression

## Slide 20
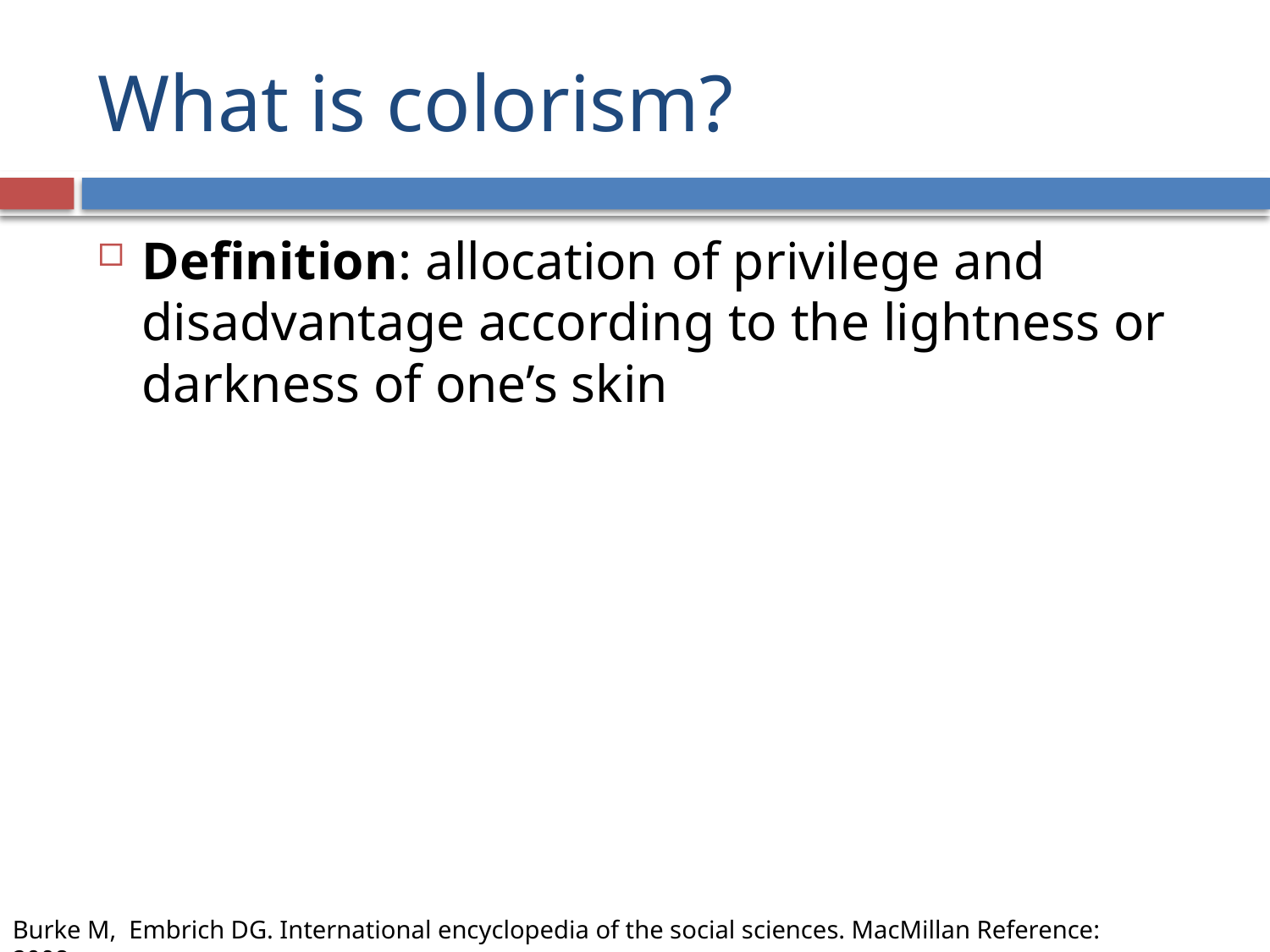

# What is colorism?
Definition: allocation of privilege and disadvantage according to the lightness or darkness of one’s skin
Burke M, Embrich DG. International encyclopedia of the social sciences. MacMillan Reference: 2008.

## Slide 21
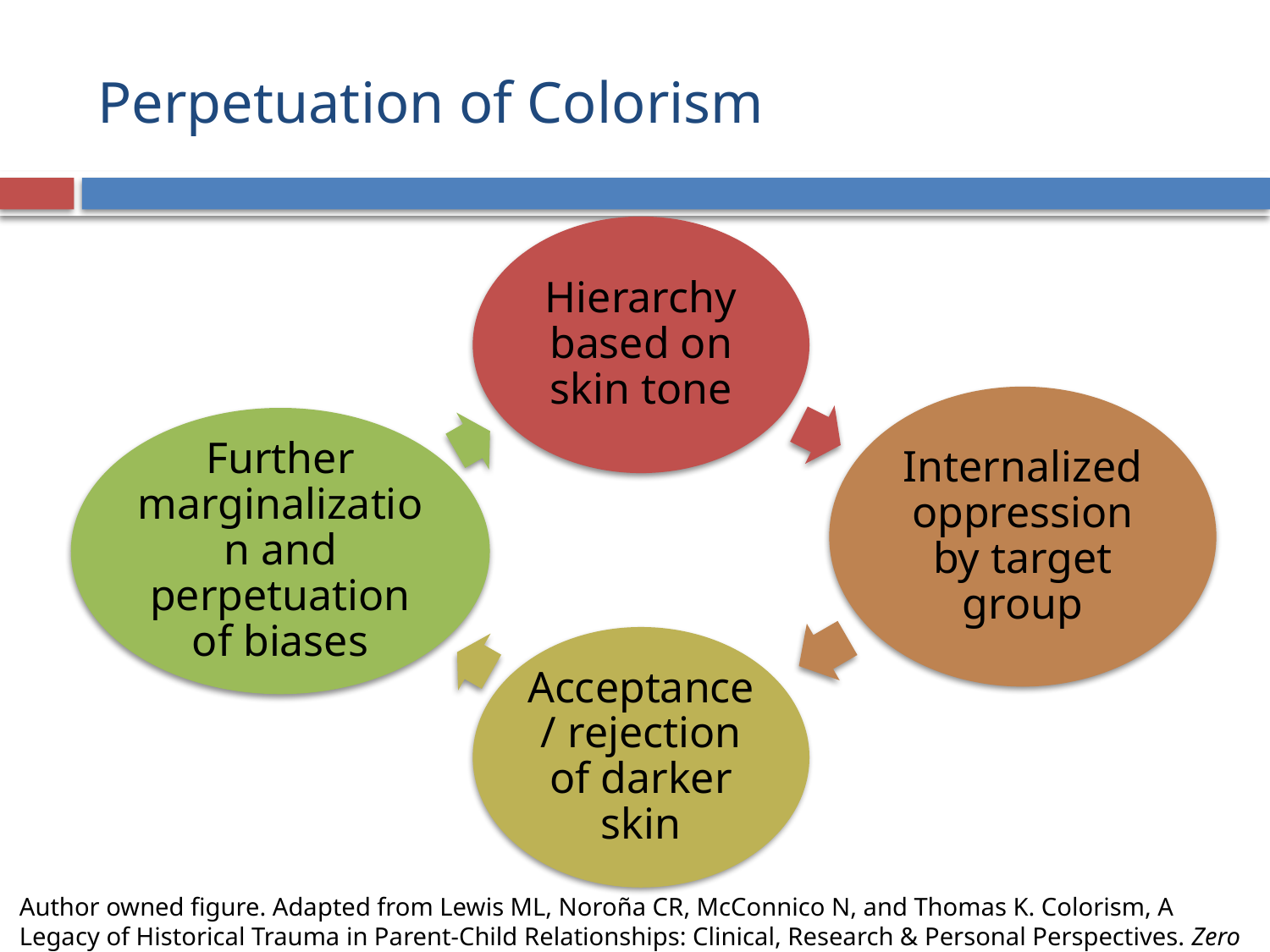

# Perpetuation of Colorism
Author owned figure. Adapted from Lewis ML, Noroña CR, McConnico N, and Thomas K. Colorism, A Legacy of Historical Trauma in Parent-Child Relationships: Clinical, Research & Personal Perspectives. Zero to Three Journal. 2013;34(2):11-23.

## Slide 22
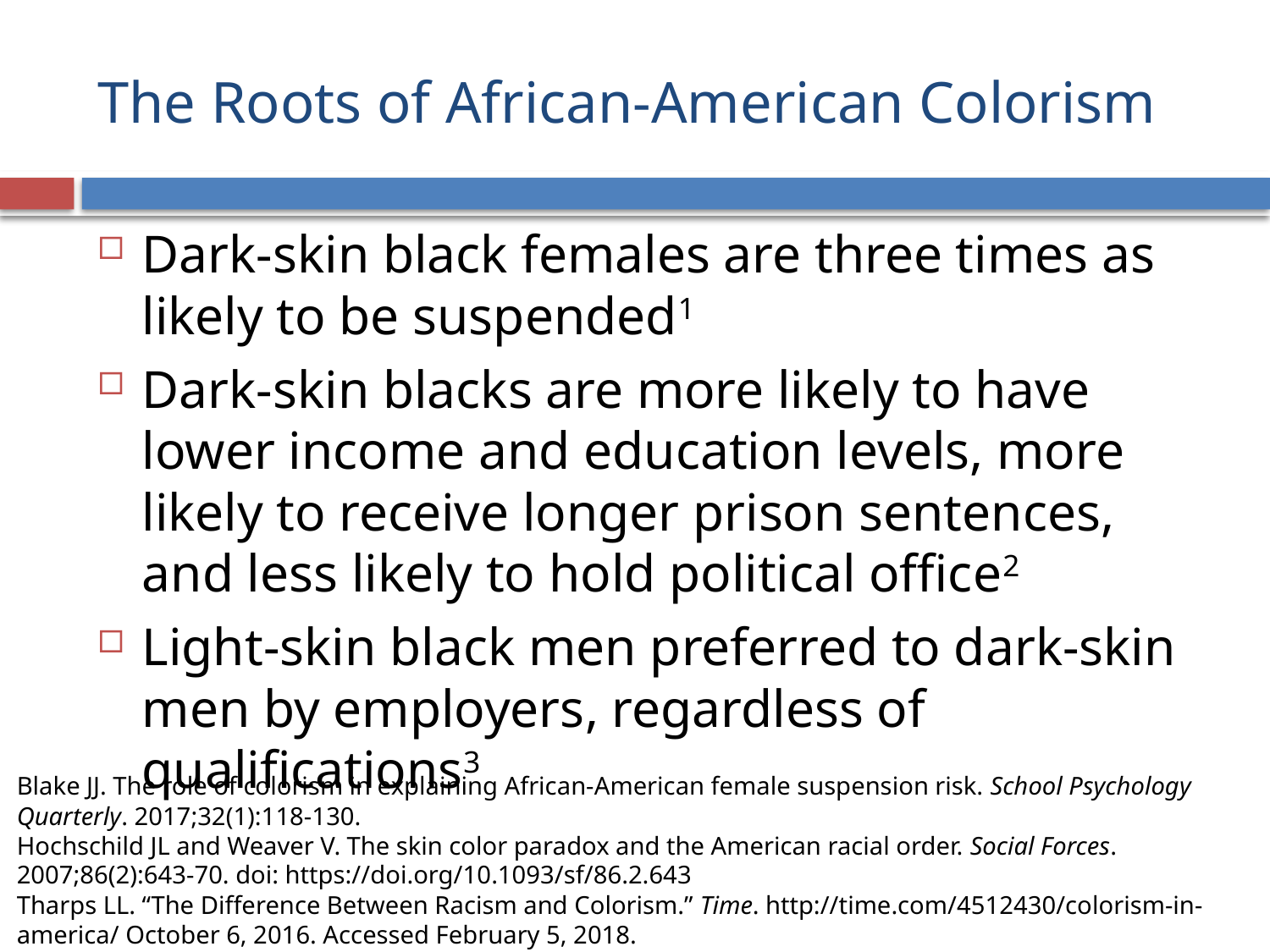

# The Roots of African-American Colorism
Dark-skin black females are three times as likely to be suspended1
Dark-skin blacks are more likely to have lower income and education levels, more likely to receive longer prison sentences, and less likely to hold political office2
Light-skin black men preferred to dark-skin men by employers, regardless of qualifications3
Blake JJ. The role of colorism in explaining African-American female suspension risk. School Psychology Quarterly. 2017;32(1):118-130.
Hochschild JL and Weaver V. The skin color paradox and the American racial order. Social Forces. 2007;86(2):643-70. doi: https://doi.org/10.1093/sf/86.2.643
Tharps LL. “The Difference Between Racism and Colorism.” Time. http://time.com/4512430/colorism-in-america/ October 6, 2016. Accessed February 5, 2018.

## Slide 23
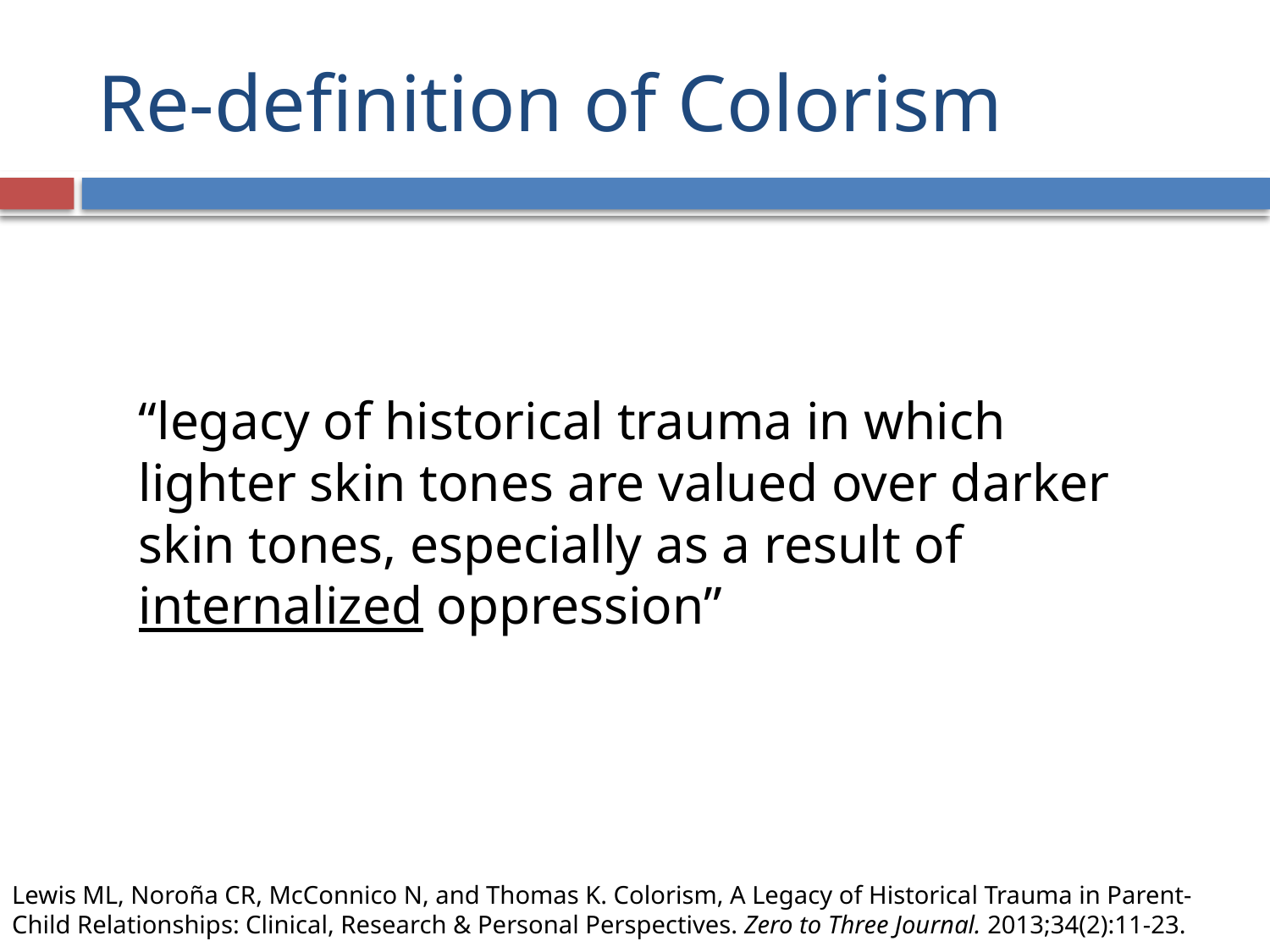

# Re-definition of Colorism
“legacy of historical trauma in which lighter skin tones are valued over darker skin tones, especially as a result of internalized oppression”
Lewis ML, Noroña CR, McConnico N, and Thomas K. Colorism, A Legacy of Historical Trauma in Parent-Child Relationships: Clinical, Research & Personal Perspectives. Zero to Three Journal. 2013;34(2):11-23.

## Slide 24
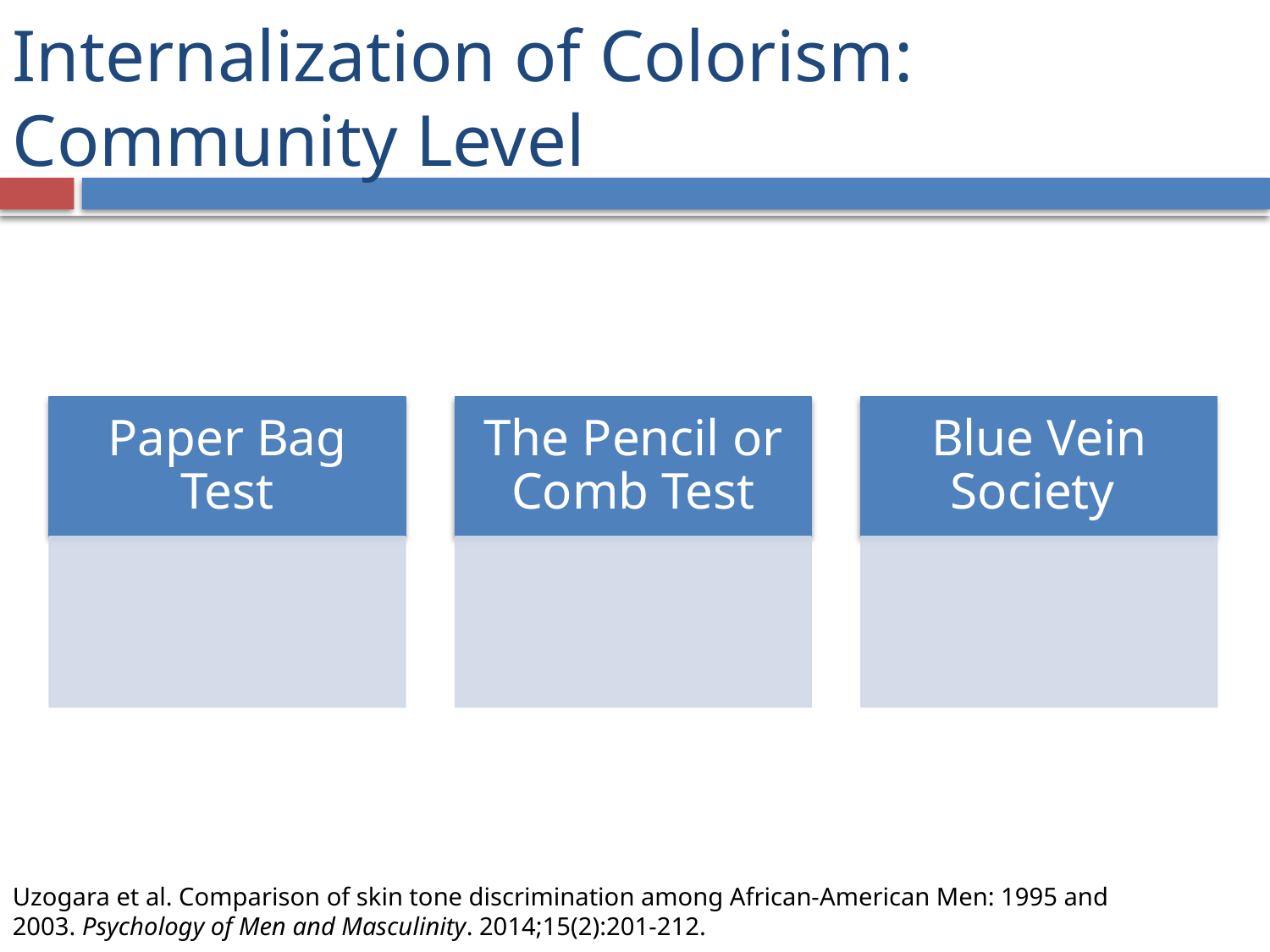

# Internalization of Colorism: Community Level
Uzogara et al. Comparison of skin tone discrimination among African-American Men: 1995 and 2003. Psychology of Men and Masculinity. 2014;15(2):201-212.

## Slide 25
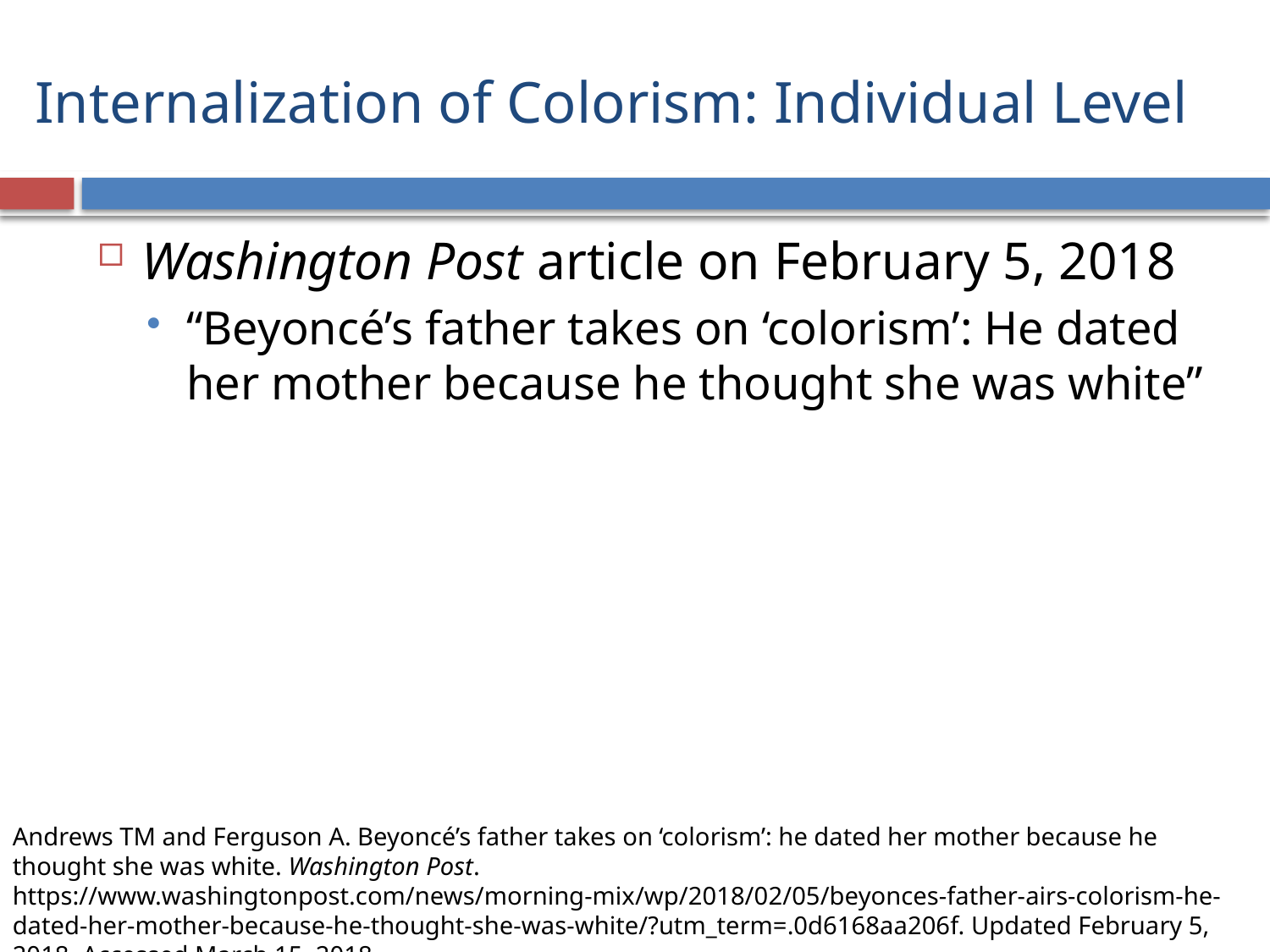

# Internalization of Colorism: Individual Level
Washington Post article on February 5, 2018
“Beyoncé’s father takes on ‘colorism’: He dated her mother because he thought she was white”
Andrews TM and Ferguson A. Beyoncé’s father takes on ‘colorism’: he dated her mother because he thought she was white. Washington Post. https://www.washingtonpost.com/news/morning-mix/wp/2018/02/05/beyonces-father-airs-colorism-he-dated-her-mother-because-he-thought-she-was-white/?utm_term=.0d6168aa206f. Updated February 5, 2018. Accessed March 15, 2018.

## Slide 26
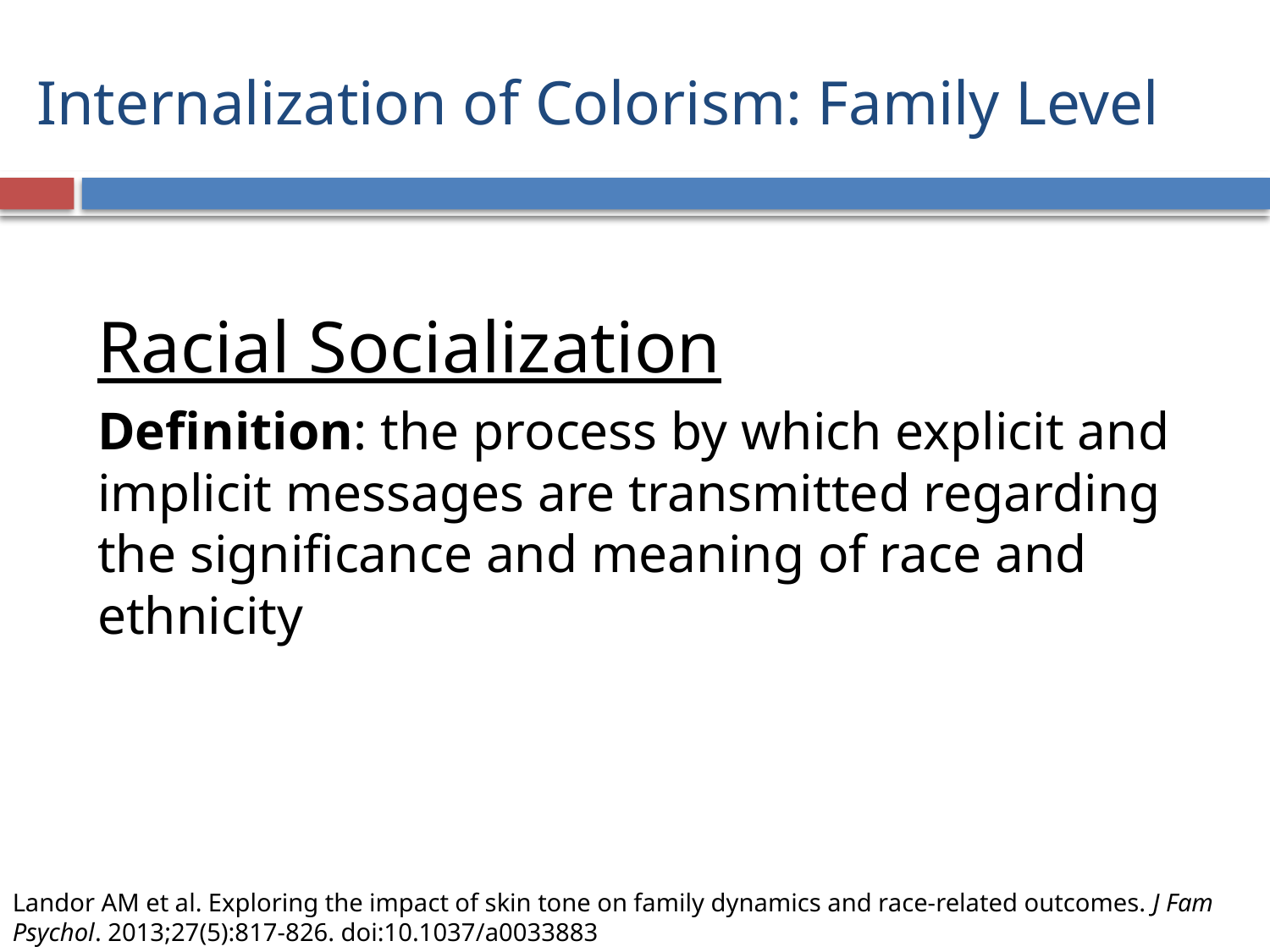

# Internalization of Colorism: Family Level
Racial Socialization
Definition: the process by which explicit and implicit messages are transmitted regarding the significance and meaning of race and ethnicity
Landor AM et al. Exploring the impact of skin tone on family dynamics and race-related outcomes. J Fam Psychol. 2013;27(5):817-826. doi:10.1037/a0033883

## Slide 27
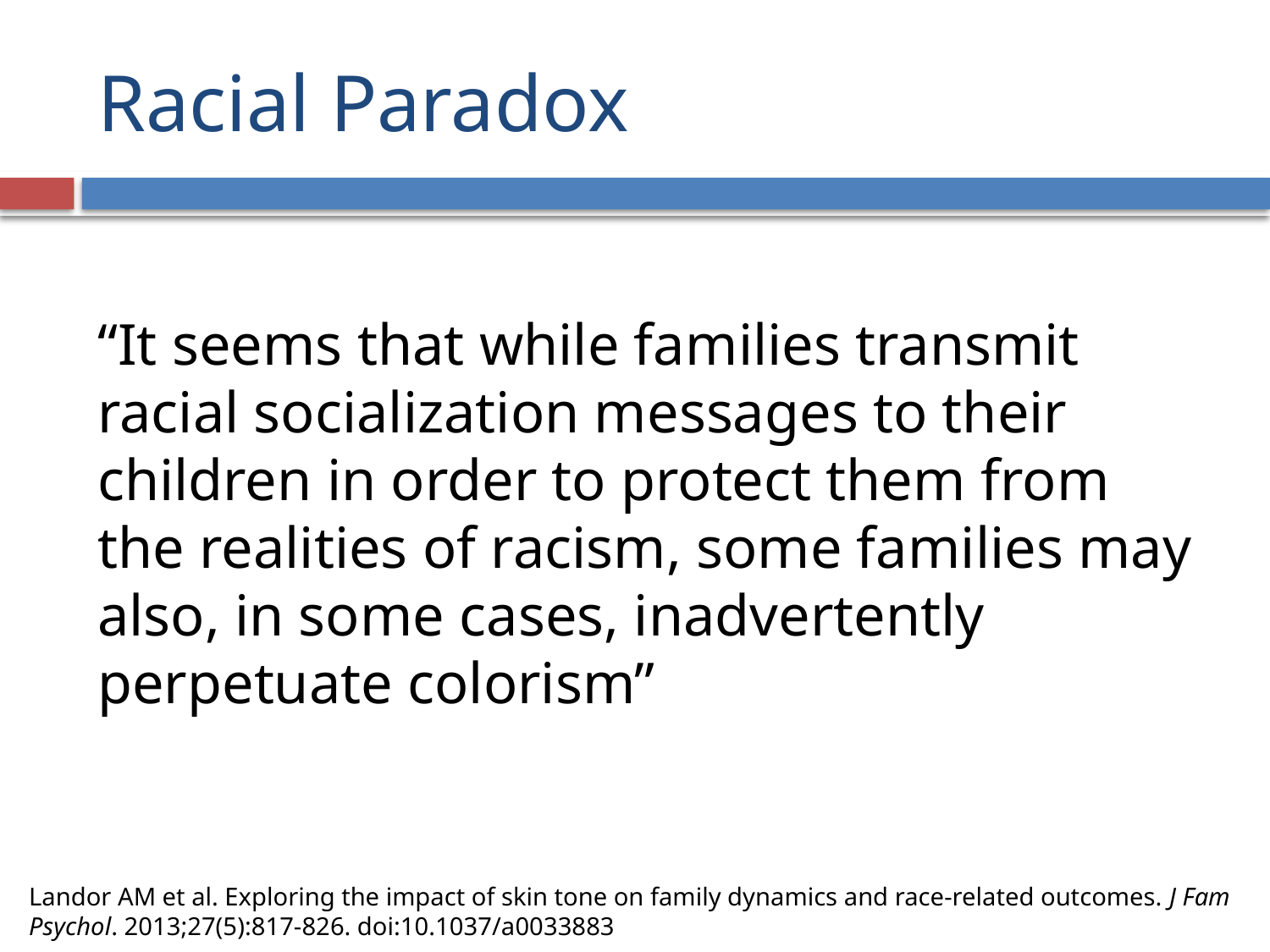

# Racial Paradox
“It seems that while families transmit racial socialization messages to their children in order to protect them from the realities of racism, some families may also, in some cases, inadvertently perpetuate colorism”
Landor AM et al. Exploring the impact of skin tone on family dynamics and race-related outcomes. J Fam Psychol. 2013;27(5):817-826. doi:10.1037/a0033883

## Slide 28
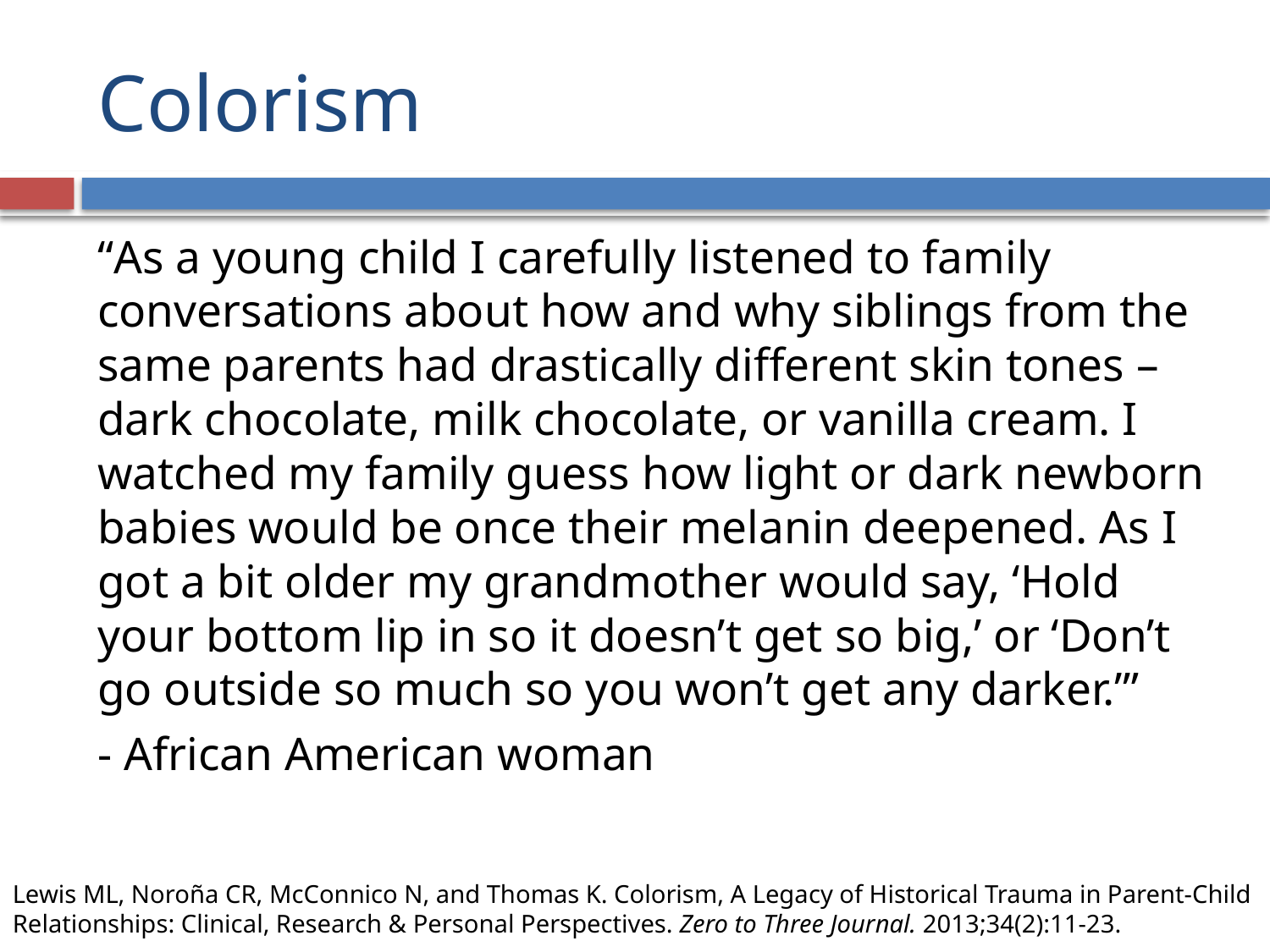

# Colorism
“As a young child I carefully listened to family conversations about how and why siblings from the same parents had drastically different skin tones – dark chocolate, milk chocolate, or vanilla cream. I watched my family guess how light or dark newborn babies would be once their melanin deepened. As I got a bit older my grandmother would say, ‘Hold your bottom lip in so it doesn’t get so big,’ or ‘Don’t go outside so much so you won’t get any darker.’”
- African American woman
Lewis ML, Noroña CR, McConnico N, and Thomas K. Colorism, A Legacy of Historical Trauma in Parent-Child Relationships: Clinical, Research & Personal Perspectives. Zero to Three Journal. 2013;34(2):11-23.

## Slide 29
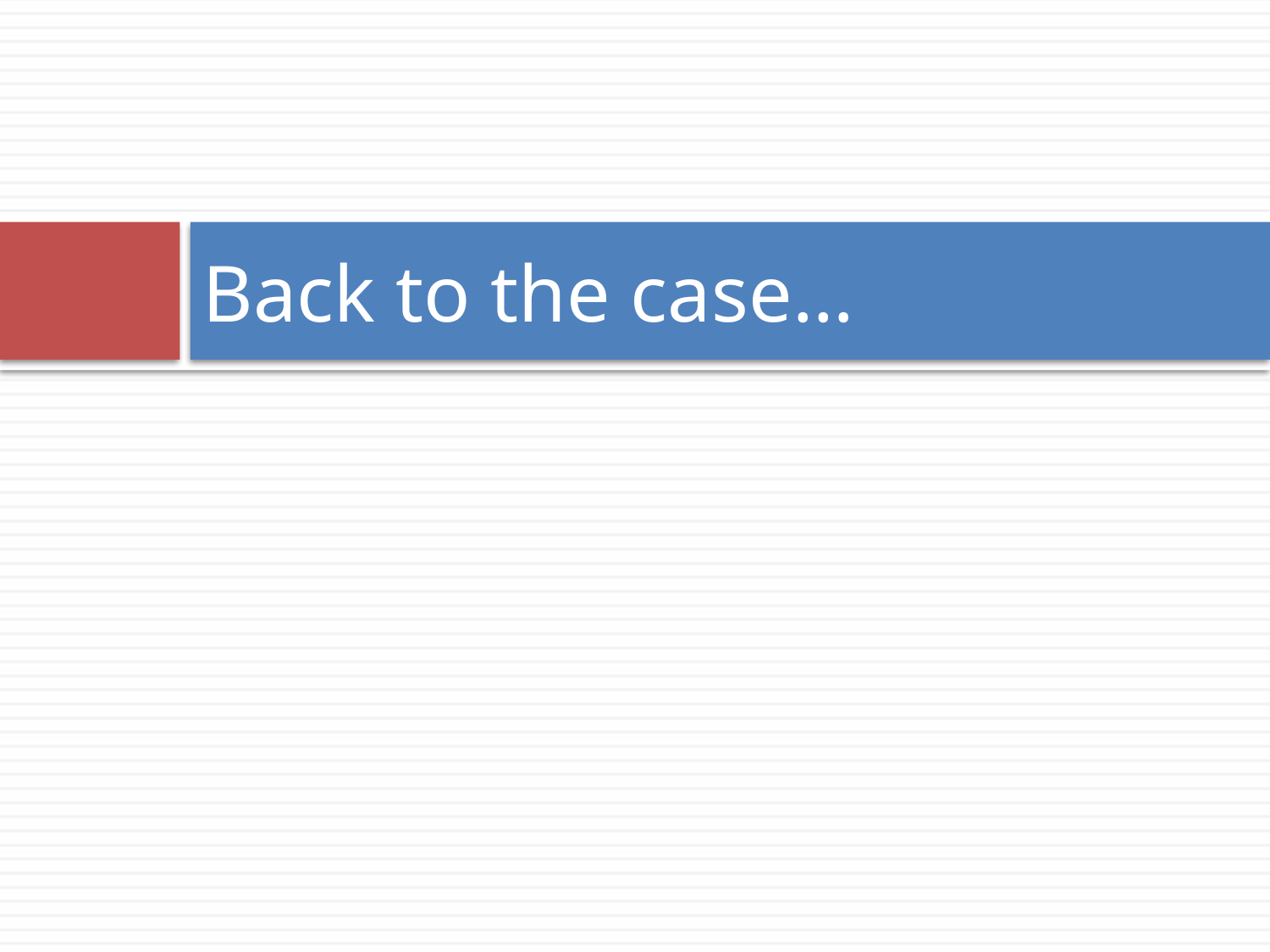

# Back to the case…

## Slide 30
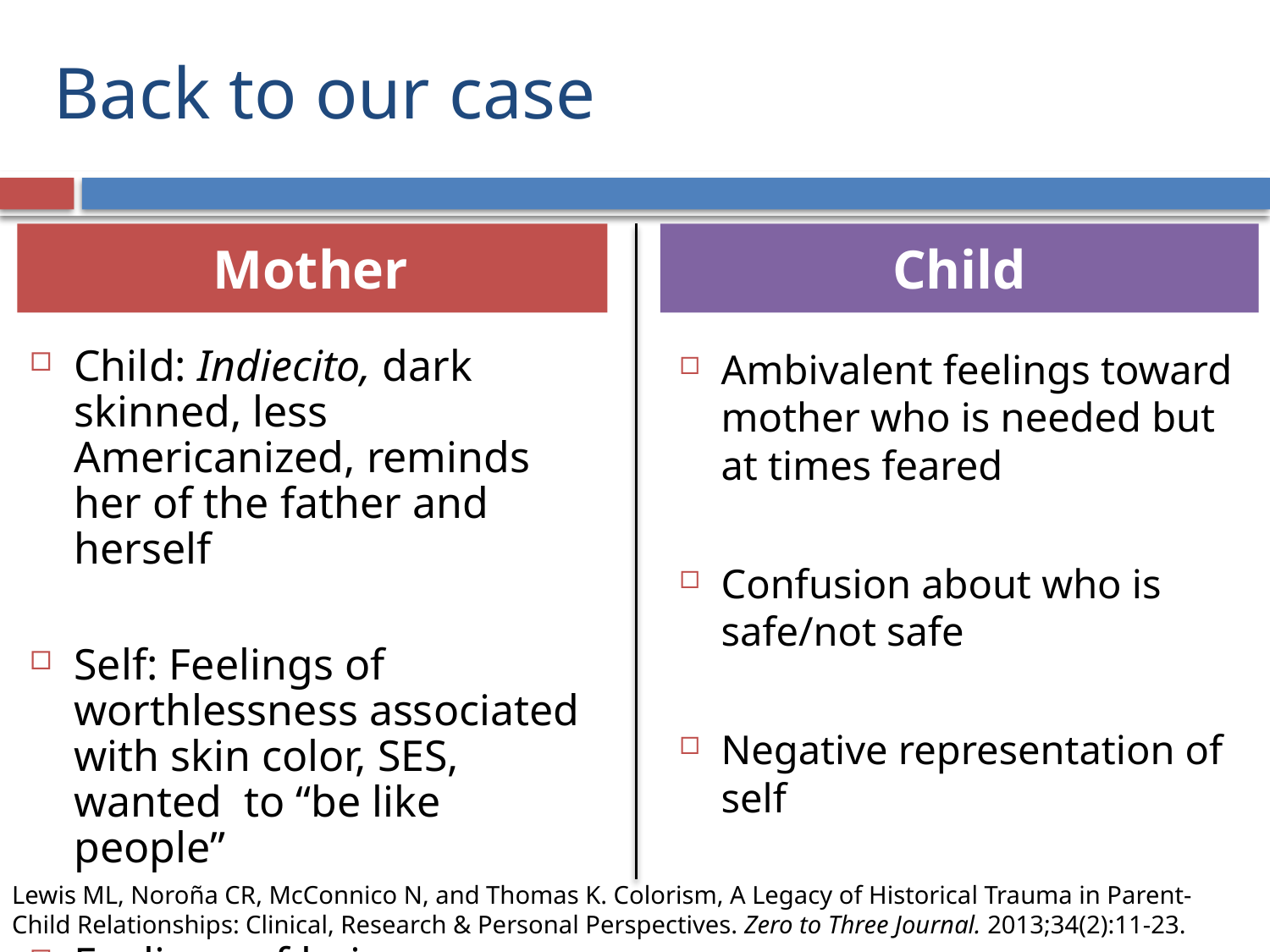

# Back to our case
 Mother
Child
Child: Indiecito, dark skinned, less Americanized, reminds her of the father and herself
Self: Feelings of worthlessness associated with skin color, SES, wanted to “be like people”
Feelings of being damaged
Ambivalent feelings toward mother who is needed but at times feared
Confusion about who is safe/not safe
Negative representation of self
Lewis ML, Noroña CR, McConnico N, and Thomas K. Colorism, A Legacy of Historical Trauma in Parent-Child Relationships: Clinical, Research & Personal Perspectives. Zero to Three Journal. 2013;34(2):11-23.

## Slide 31
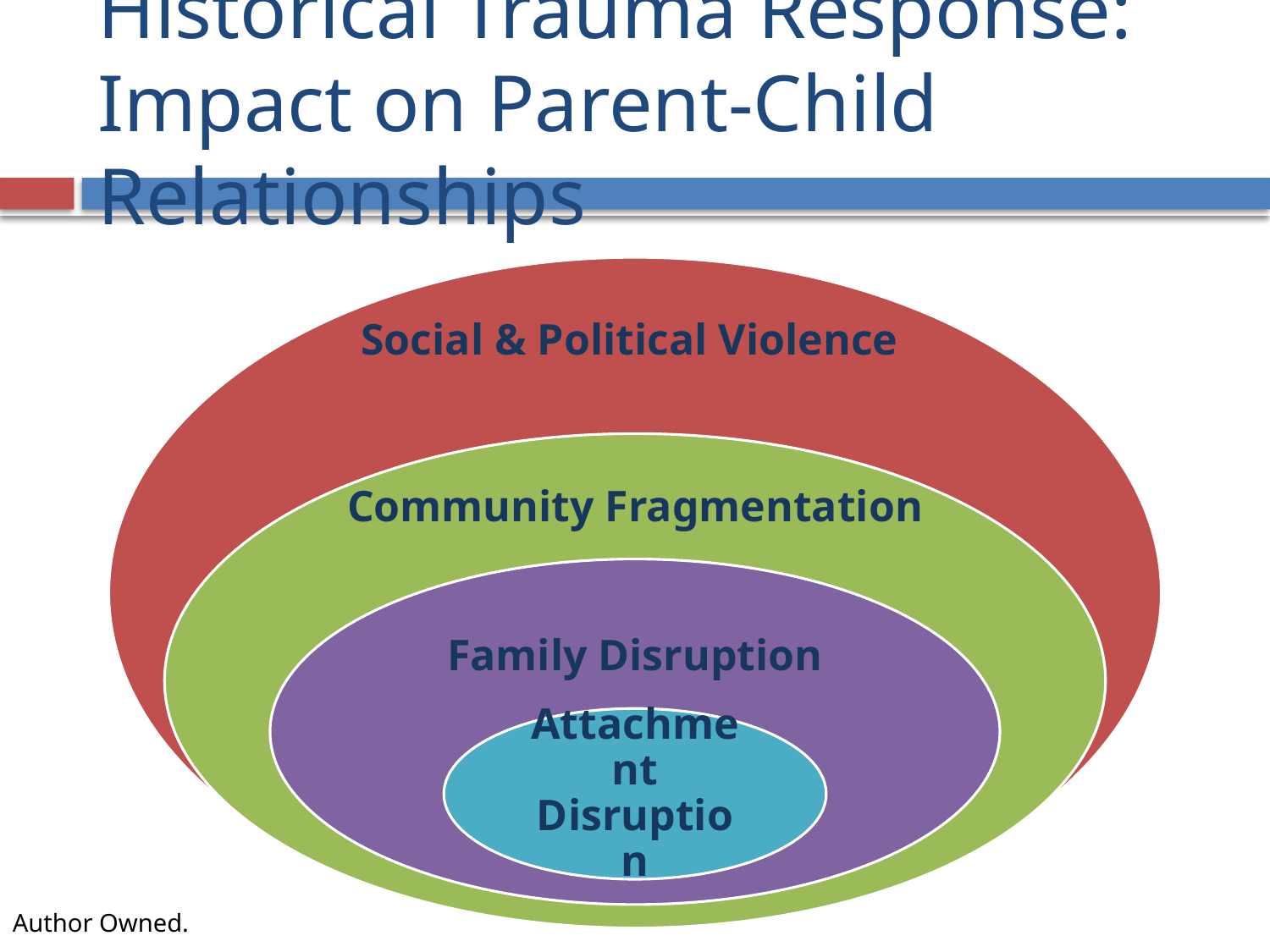

# Historical Trauma Response: Impact on Parent-Child Relationships
Author Owned.

## Slide 32
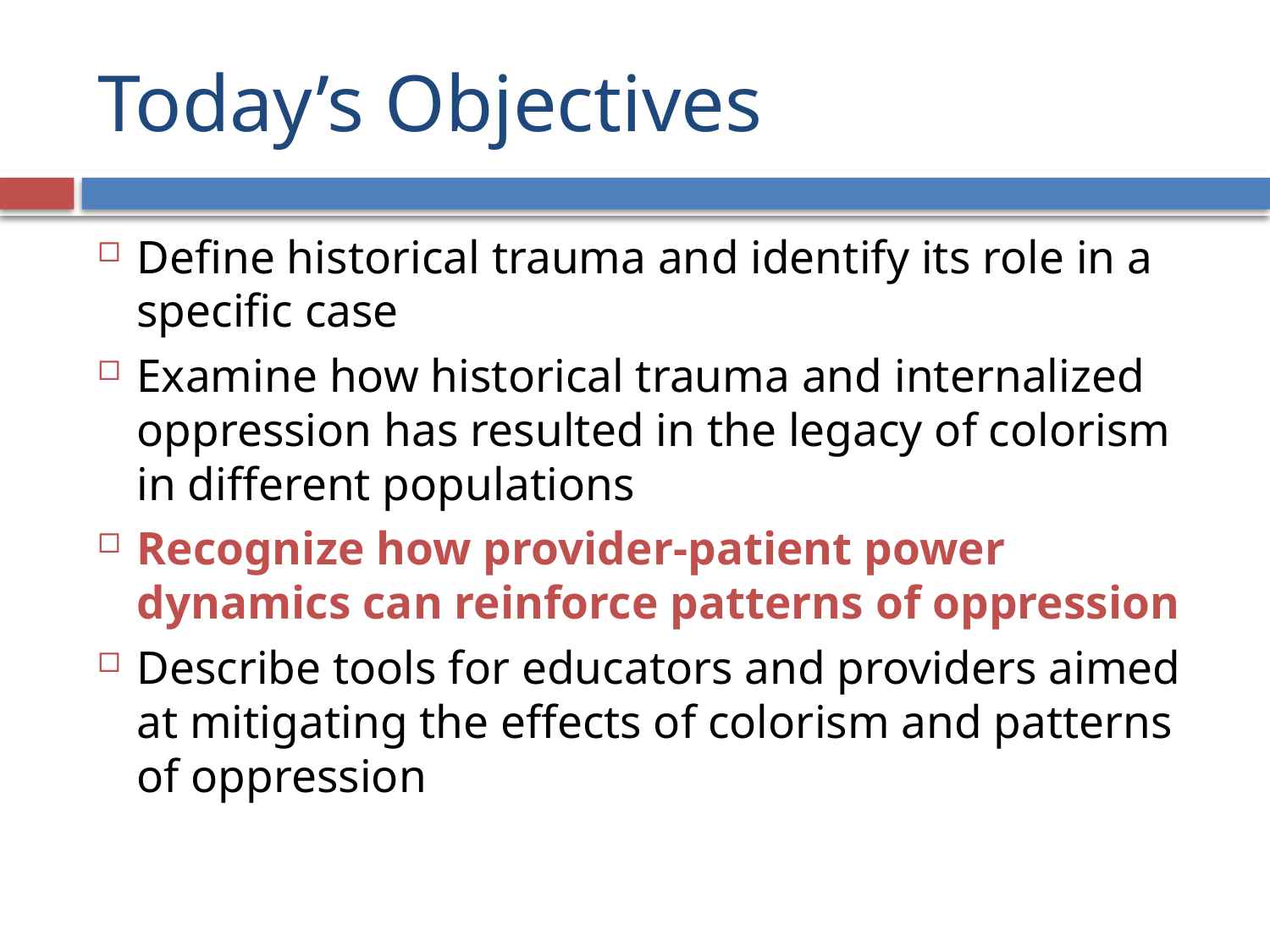

# Today’s Objectives
Define historical trauma and identify its role in a specific case
Examine how historical trauma and internalized oppression has resulted in the legacy of colorism in different populations
Recognize how provider-patient power dynamics can reinforce patterns of oppression
Describe tools for educators and providers aimed at mitigating the effects of colorism and patterns of oppression

## Slide 33
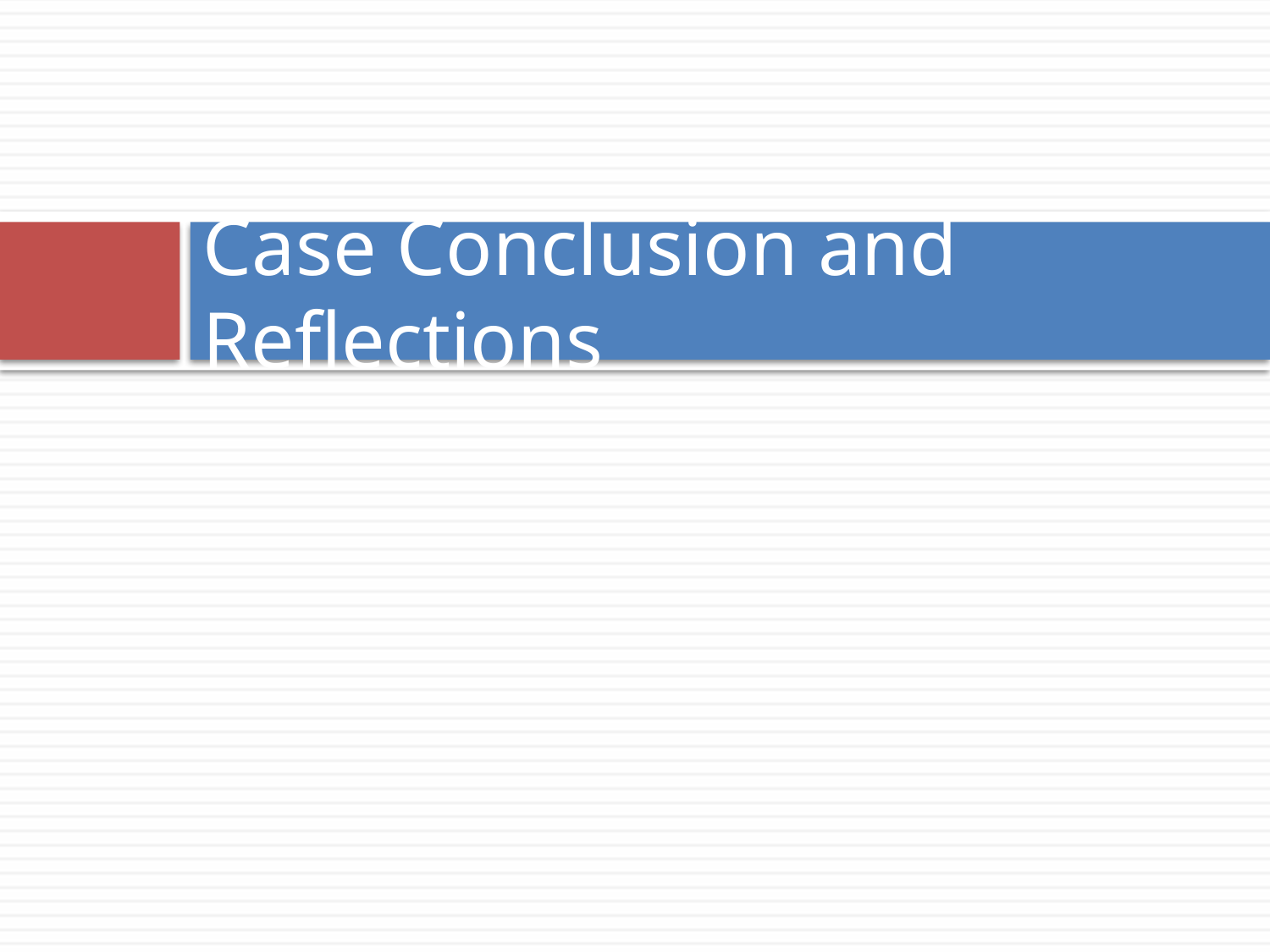

# Case Conclusion and Reflections

## Slide 34
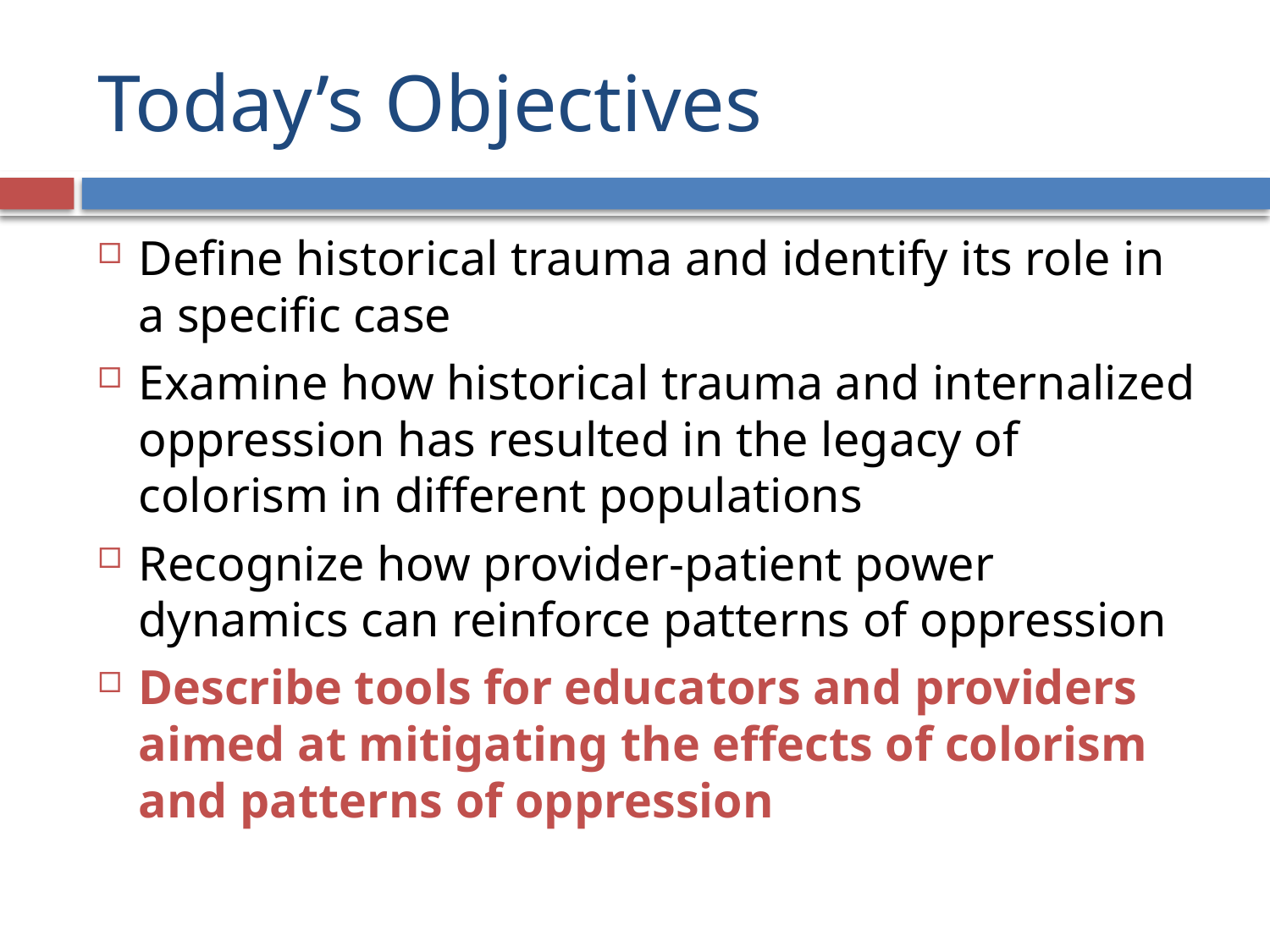

# Today’s Objectives
Define historical trauma and identify its role in a specific case
Examine how historical trauma and internalized oppression has resulted in the legacy of colorism in different populations
Recognize how provider-patient power dynamics can reinforce patterns of oppression
Describe tools for educators and providers aimed at mitigating the effects of colorism and patterns of oppression

## Slide 35
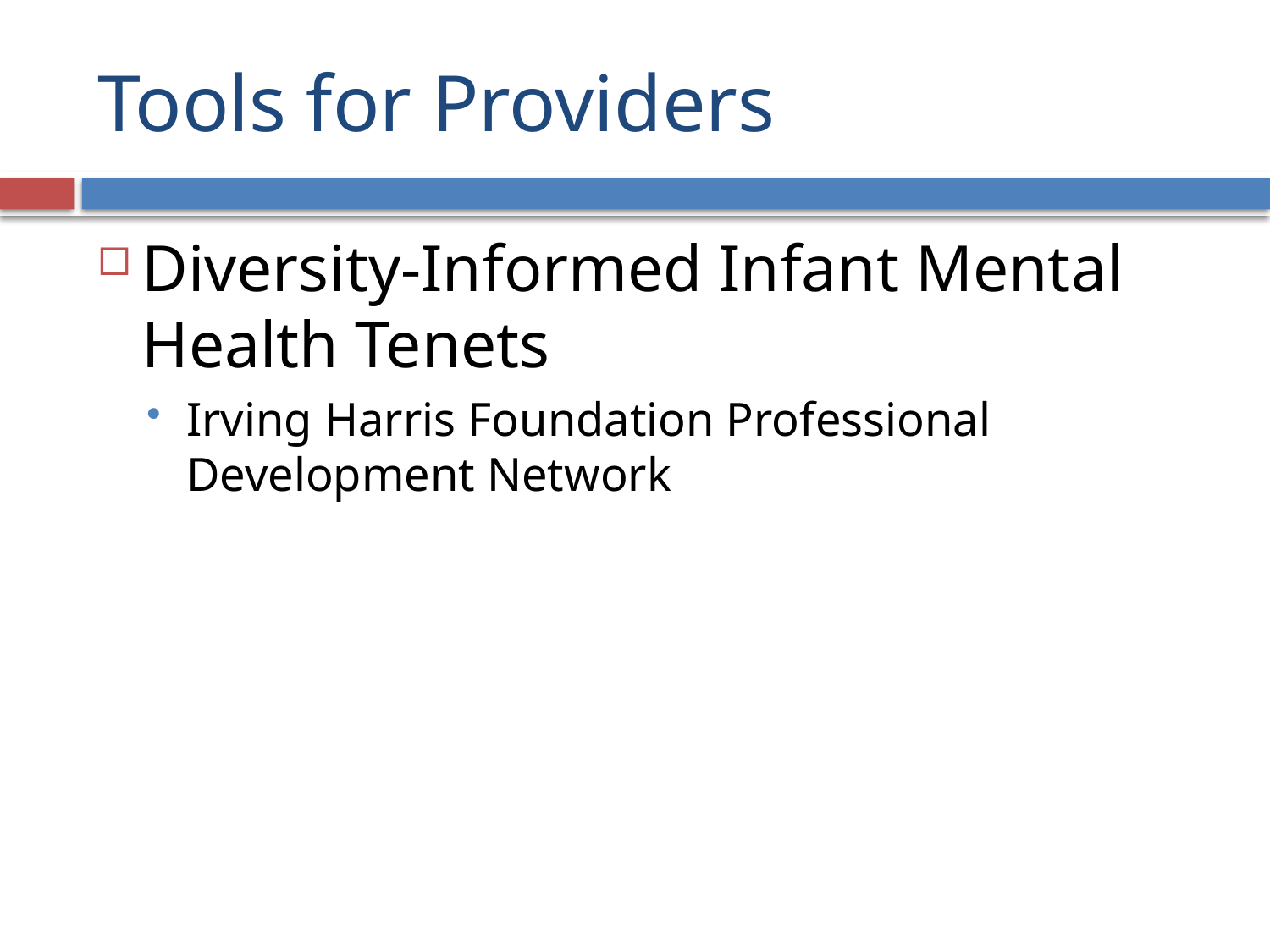

# Tools for Providers
Diversity-Informed Infant Mental Health Tenets
Irving Harris Foundation Professional Development Network

## Slide 36
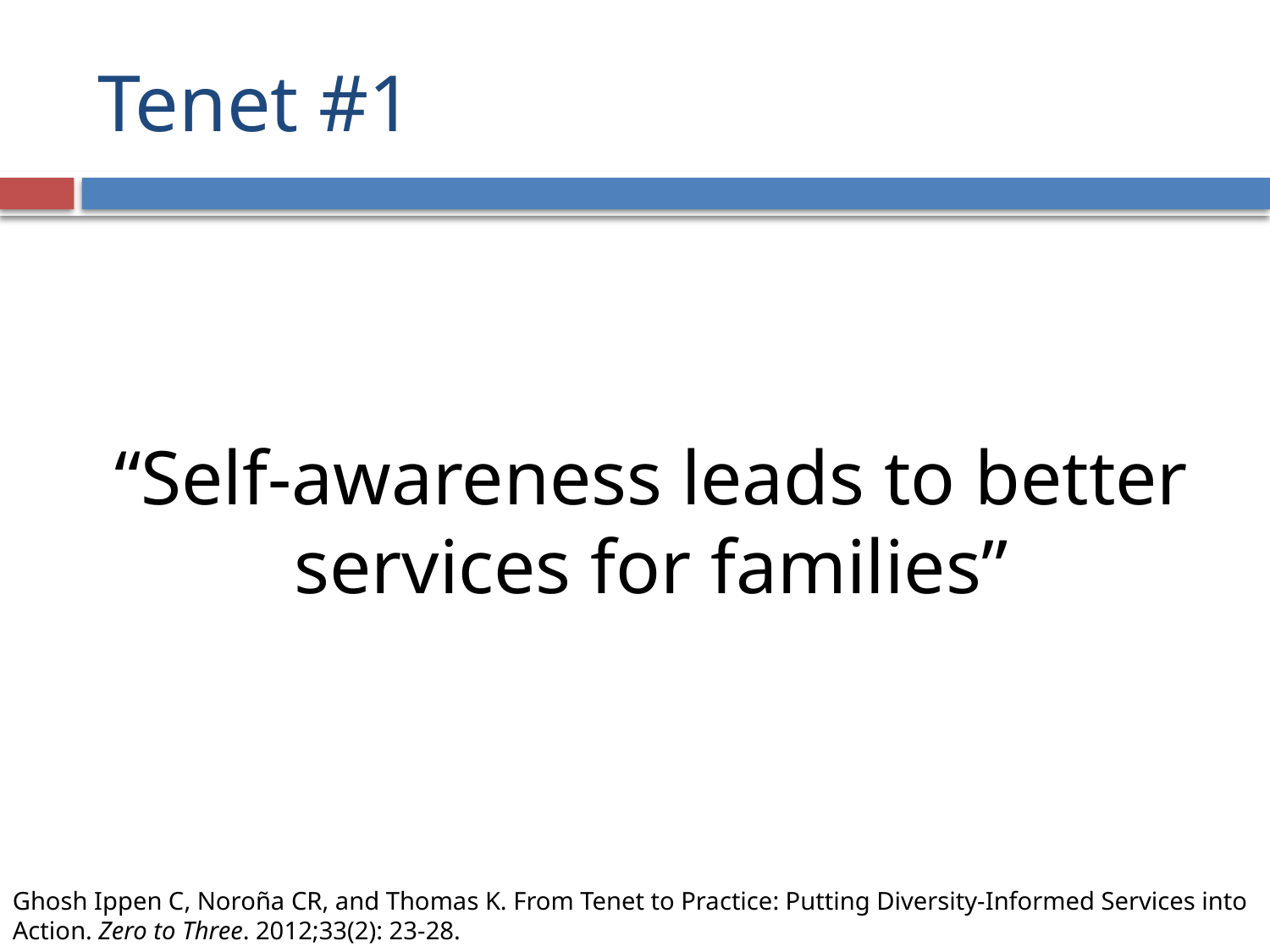

# Tenet #1
“Self-awareness leads to better services for families”
Ghosh Ippen C, Noroña CR, and Thomas K. From Tenet to Practice: Putting Diversity-Informed Services into Action. Zero to Three. 2012;33(2): 23-28.

## Slide 37
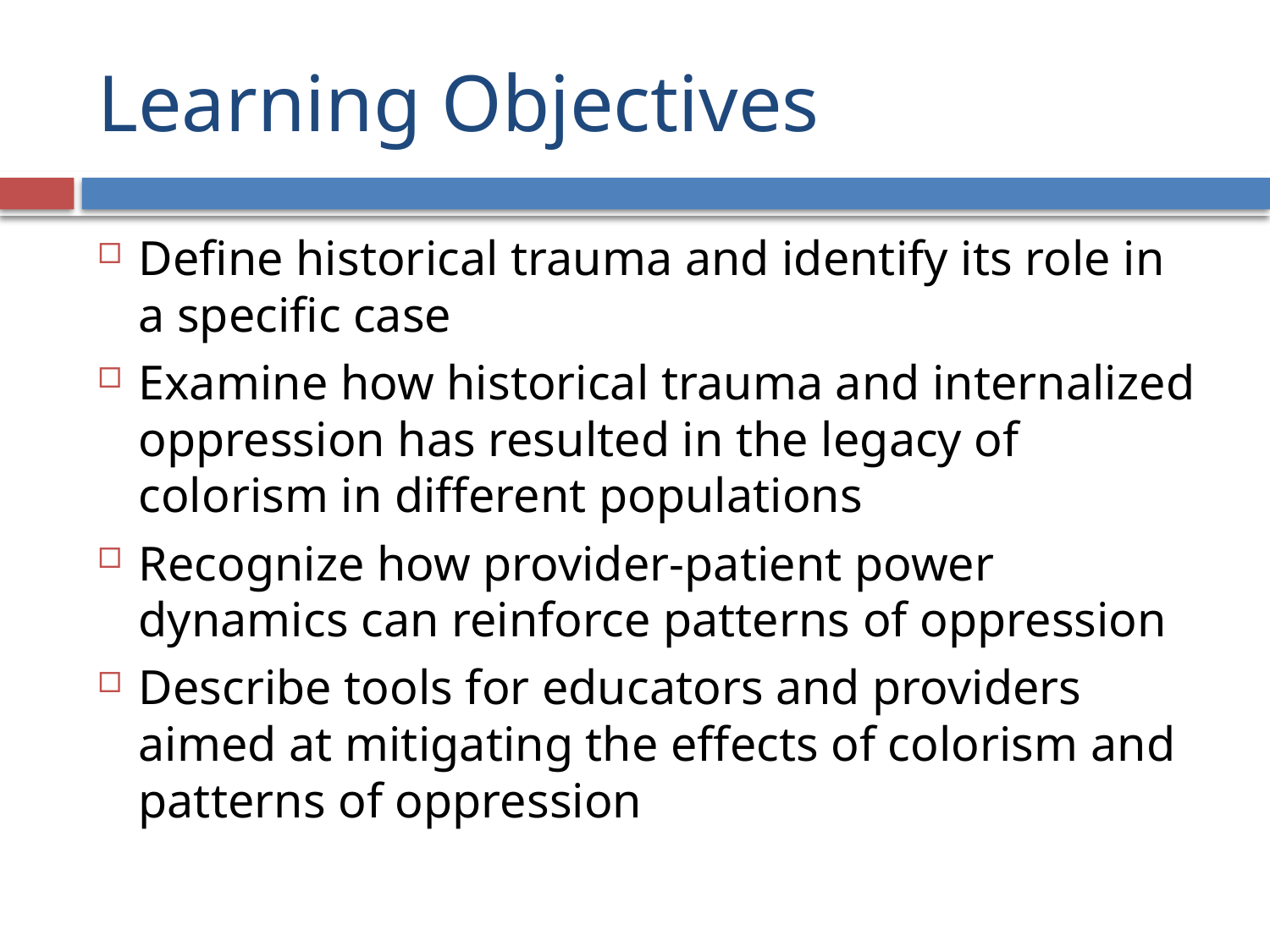

# Learning Objectives
Define historical trauma and identify its role in a specific case
Examine how historical trauma and internalized oppression has resulted in the legacy of colorism in different populations
Recognize how provider-patient power dynamics can reinforce patterns of oppression
Describe tools for educators and providers aimed at mitigating the effects of colorism and patterns of oppression

## Slide 38
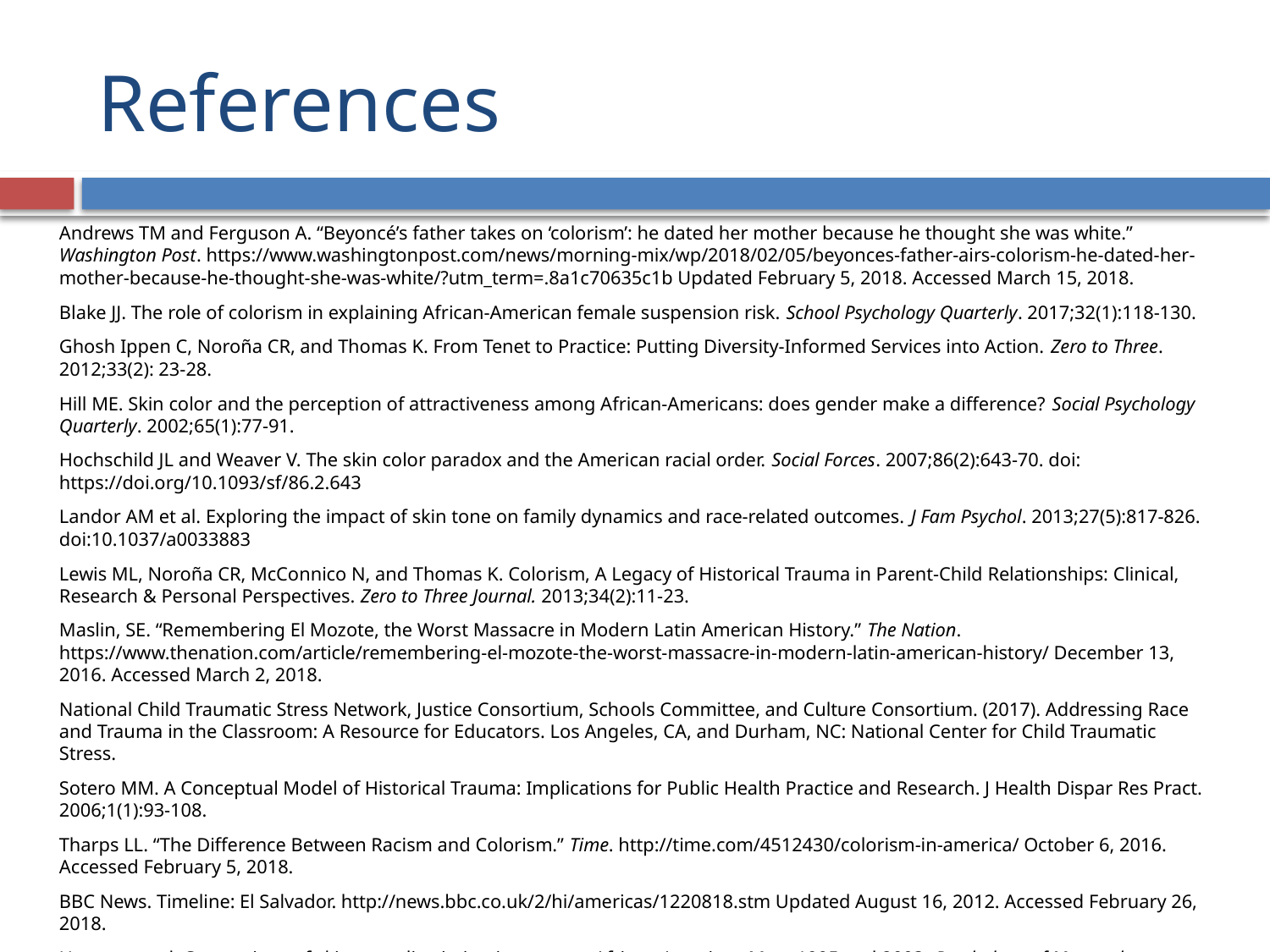

# References
Andrews TM and Ferguson A. “Beyoncé’s father takes on ‘colorism’: he dated her mother because he thought she was white.” Washington Post. https://www.washingtonpost.com/news/morning-mix/wp/2018/02/05/beyonces-father-airs-colorism-he-dated-her-mother-because-he-thought-she-was-white/?utm_term=.8a1c70635c1b Updated February 5, 2018. Accessed March 15, 2018.
Blake JJ. The role of colorism in explaining African-American female suspension risk. School Psychology Quarterly. 2017;32(1):118-130.
Ghosh Ippen C, Noroña CR, and Thomas K. From Tenet to Practice: Putting Diversity-Informed Services into Action. Zero to Three. 2012;33(2): 23-28.
Hill ME. Skin color and the perception of attractiveness among African-Americans: does gender make a difference? Social Psychology Quarterly. 2002;65(1):77-91.
Hochschild JL and Weaver V. The skin color paradox and the American racial order. Social Forces. 2007;86(2):643-70. doi: https://doi.org/10.1093/sf/86.2.643
Landor AM et al. Exploring the impact of skin tone on family dynamics and race-related outcomes. J Fam Psychol. 2013;27(5):817-826. doi:10.1037/a0033883
Lewis ML, Noroña CR, McConnico N, and Thomas K. Colorism, A Legacy of Historical Trauma in Parent-Child Relationships: Clinical, Research & Personal Perspectives. Zero to Three Journal. 2013;34(2):11-23.
Maslin, SE. “Remembering El Mozote, the Worst Massacre in Modern Latin American History.” The Nation. https://www.thenation.com/article/remembering-el-mozote-the-worst-massacre-in-modern-latin-american-history/ December 13, 2016. Accessed March 2, 2018.
National Child Traumatic Stress Network, Justice Consortium, Schools Committee, and Culture Consortium. (2017). Addressing Race and Trauma in the Classroom: A Resource for Educators. Los Angeles, CA, and Durham, NC: National Center for Child Traumatic Stress.
Sotero MM. A Conceptual Model of Historical Trauma: Implications for Public Health Practice and Research. J Health Dispar Res Pract. 2006;1(1):93-108.
Tharps LL. “The Difference Between Racism and Colorism.” Time. http://time.com/4512430/colorism-in-america/ October 6, 2016. Accessed February 5, 2018.
BBC News. Timeline: El Salvador. http://news.bbc.co.uk/2/hi/americas/1220818.stm Updated August 16, 2012. Accessed February 26, 2018.
Uzogara et al. Comparison of skin tone discrimination among African-American Men: 1995 and 2003. Psychology of Men and Masculinity. 2014;15(2):201-212.
Viglione J. Exploring the effect of objectively assessed skin tone on prison sentences among black female offenders. ProQuest LLC. 2010.
