# Supplementary material for: Health Equity Rounds: An Interdisciplinary Case Conference to Address Implicit Bias and Structural Racism for Faculty and Trainees
Source: MedEdPORTAL. 2019 Nov 22;15:10858. doi: 10.15766/mep_2374-8265.10858 (PMC7050660; doi:10.15766/mep_2374-8265.10858)
Supplement: Supplementary file 1 — A. HER 1.pptx B. HER 2.pptx C. HER 3.pptx D. HER 4.pptx E. HER 5.pptx F. HER 6.pptx G. HER 7.pptx H. Selected HER Handouts.docx I. Case Conference Creation Guide.docx J. Glossary.docx K. Evaluation.docx [file mep-15-10858-s001.zip › G. HER 7.pptx]

## Slide 1
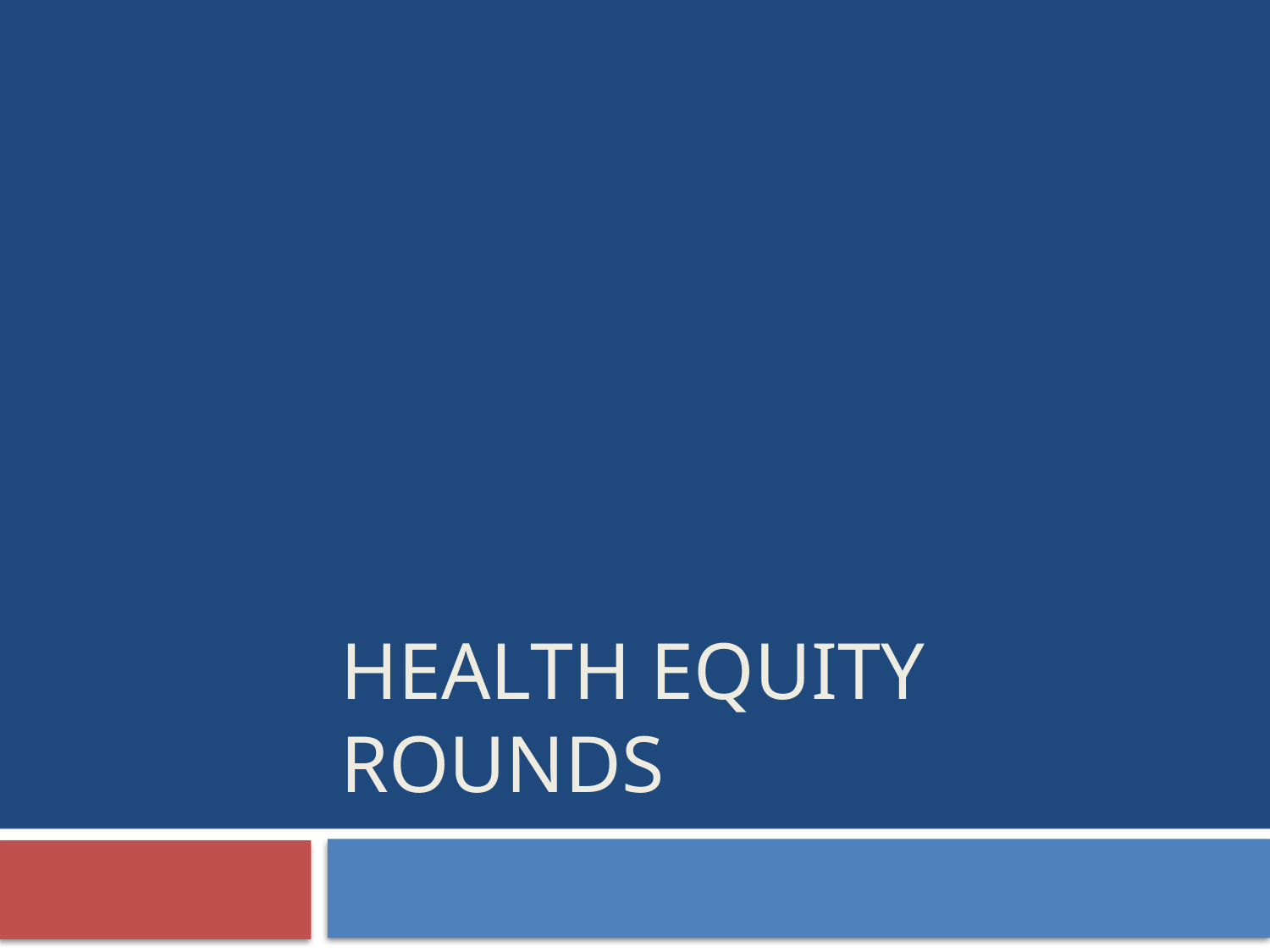

# Health equity rounds

## Slide 2
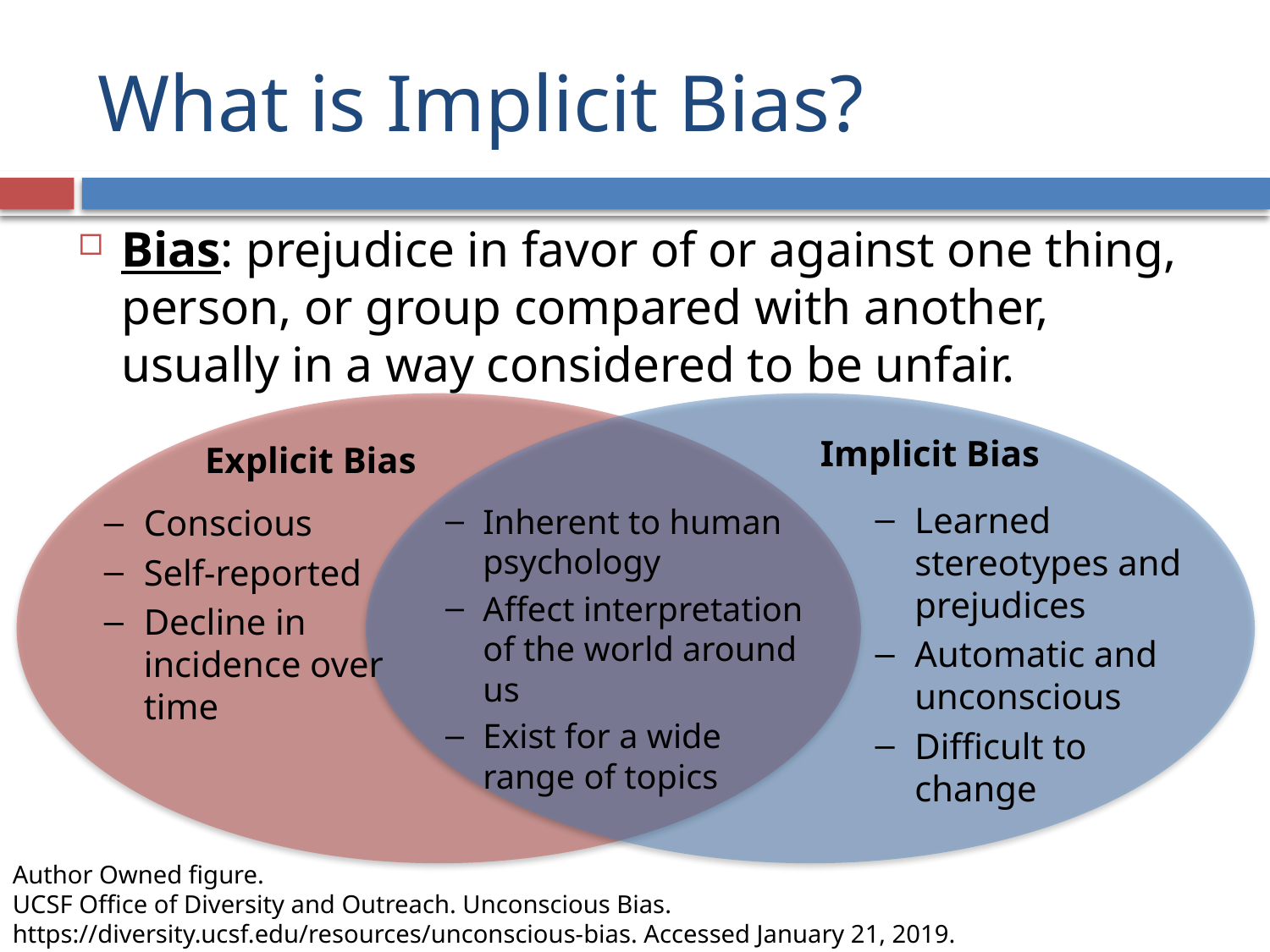

# What is Implicit Bias?
Bias: prejudice in favor of or against one thing, person, or group compared with another, usually in a way considered to be unfair.
Implicit Bias
Explicit Bias
Learned stereotypes and prejudices
Automatic and unconscious
Difficult to change
Inherent to human psychology
Affect interpretation of the world around us
Exist for a wide range of topics
Conscious
Self-reported
Decline in incidence over time
Author Owned figure.
UCSF Office of Diversity and Outreach. Unconscious Bias. https://diversity.ucsf.edu/resources/unconscious-bias. Accessed January 21, 2019.

## Slide 3
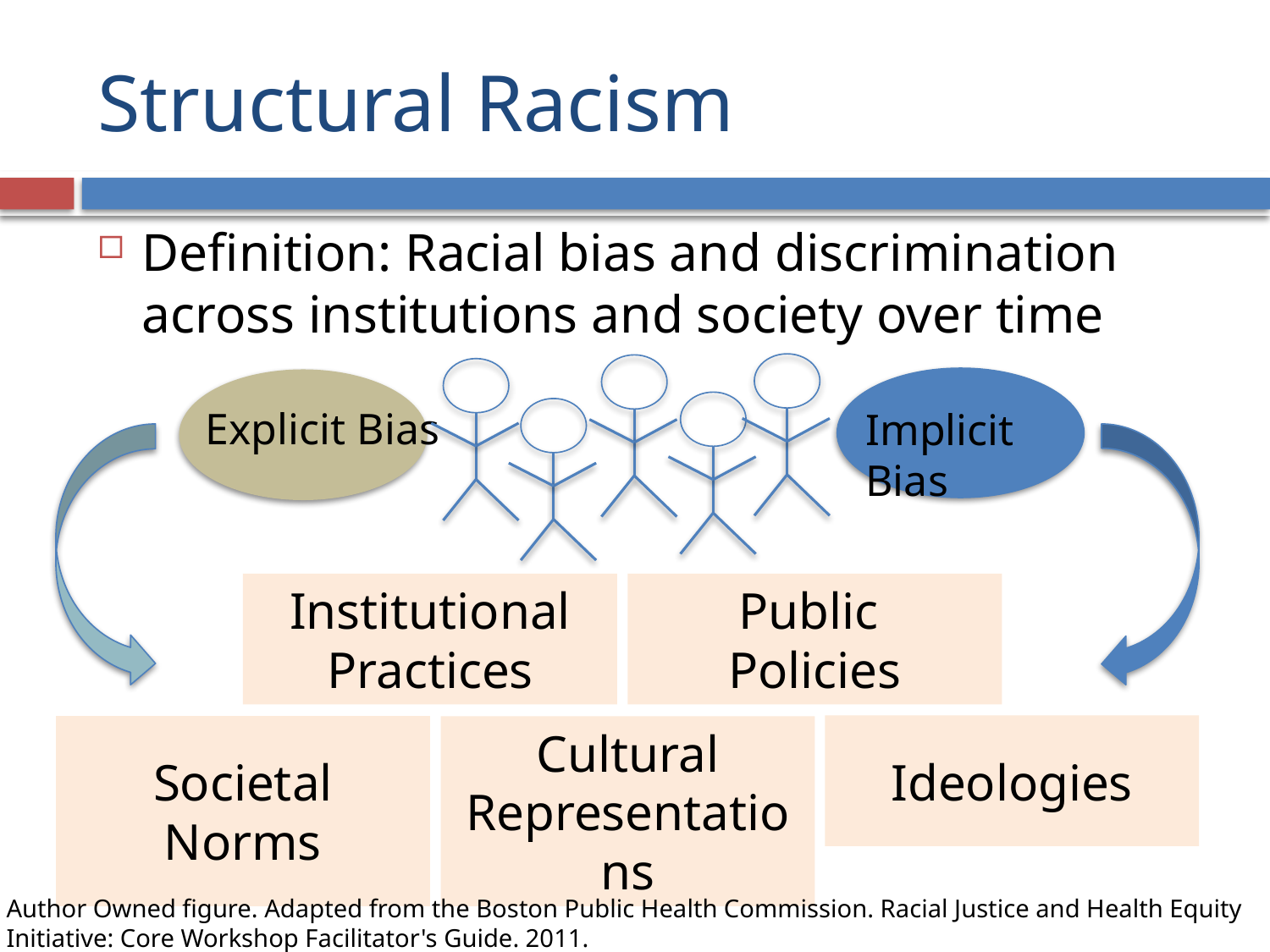

# Structural Racism
Definition: Racial bias and discrimination across institutions and society over time
Explicit Bias
Implicit Bias
Institutional Practices
Public
Policies
Ideologies
Societal Norms
Cultural
Representations
Author Owned figure. Adapted from the Boston Public Health Commission. Racial Justice and Health Equity Initiative: Core Workshop Facilitator's Guide. 2011.

## Slide 4
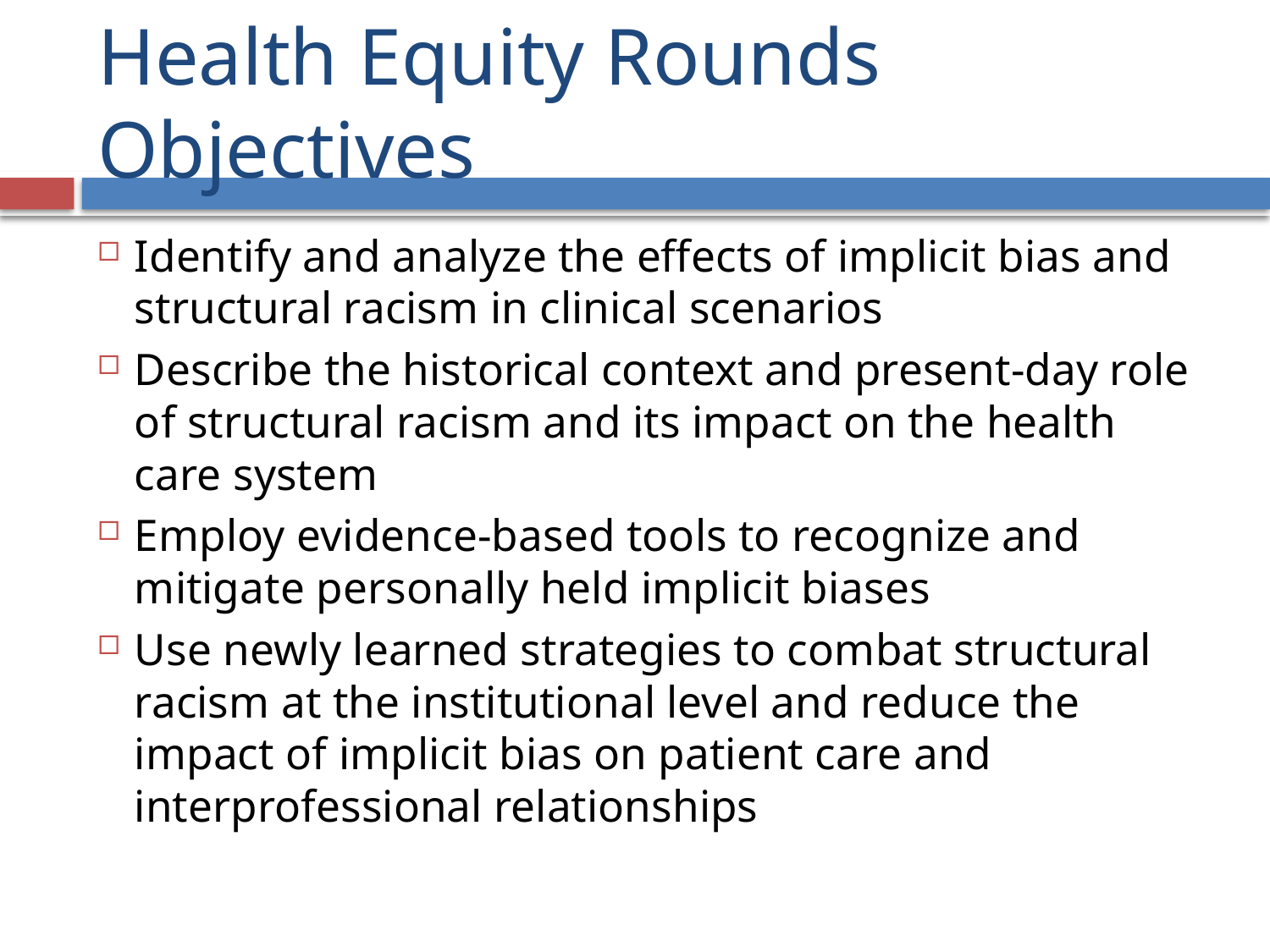

# Health Equity Rounds Objectives
Identify and analyze the effects of implicit bias and structural racism in clinical scenarios
Describe the historical context and present-day role of structural racism and its impact on the health care system
Employ evidence-based tools to recognize and mitigate personally held implicit biases
Use newly learned strategies to combat structural racism at the institutional level and reduce the impact of implicit bias on patient care and interprofessional relationships

## Slide 5
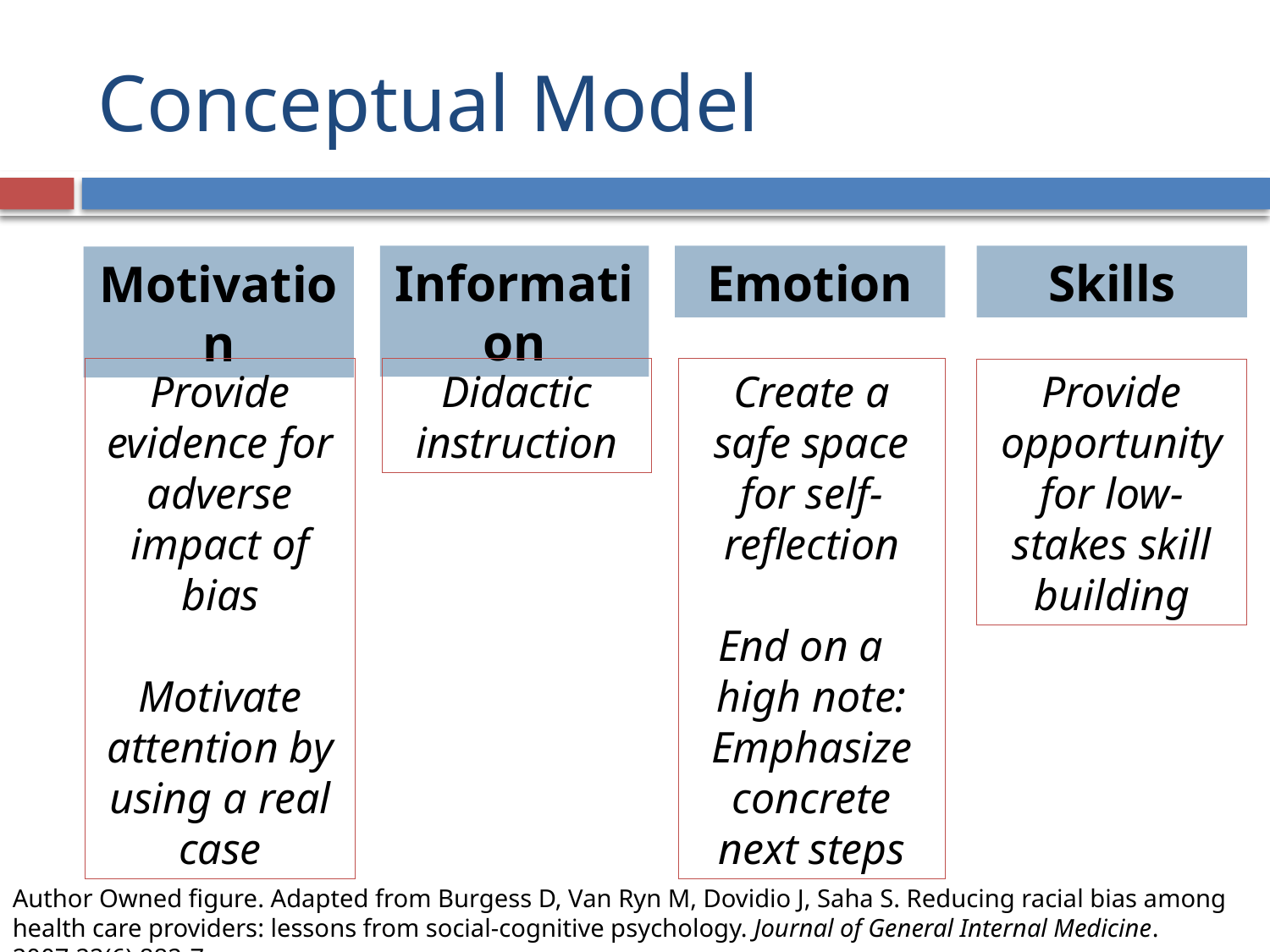

# Conceptual Model
Skills
Information
Emotion
Motivation
Create a safe space for self-reflection
d
End on a high note: Emphasize concrete next steps
Provide evidence for adverse impact of bias
Motivate attention by using a real case
Didactic instruction
Provide opportunity for low-stakes skill building
Author Owned figure. Adapted from Burgess D, Van Ryn M, Dovidio J, Saha S. Reducing racial bias among health care providers: lessons from social-cognitive psychology. Journal of General Internal Medicine. 2007;22(6):882-7.

## Slide 6
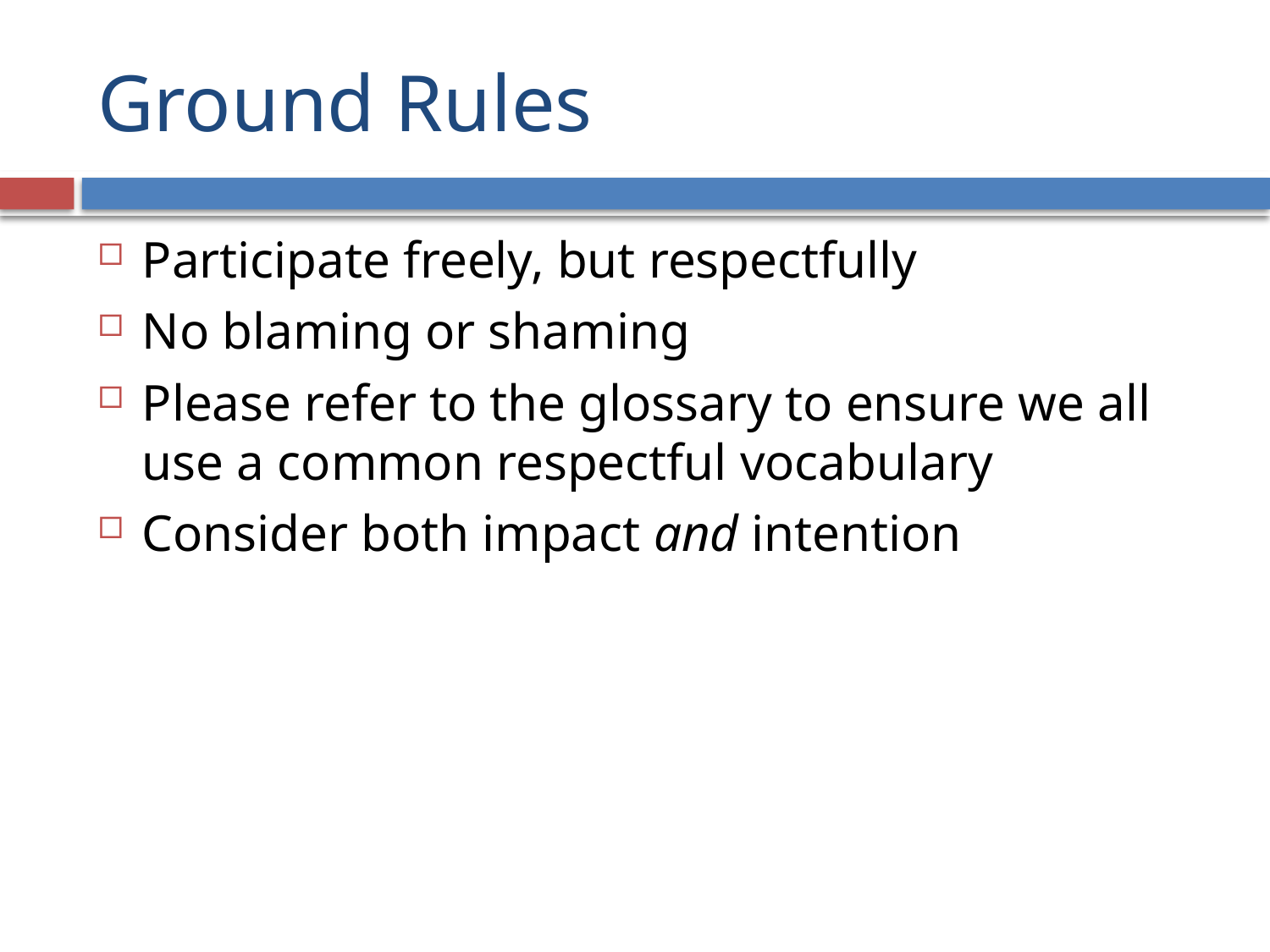

# Ground Rules
Participate freely, but respectfully
No blaming or shaming
Please refer to the glossary to ensure we all use a common respectful vocabulary
Consider both impact and intention

## Slide 7
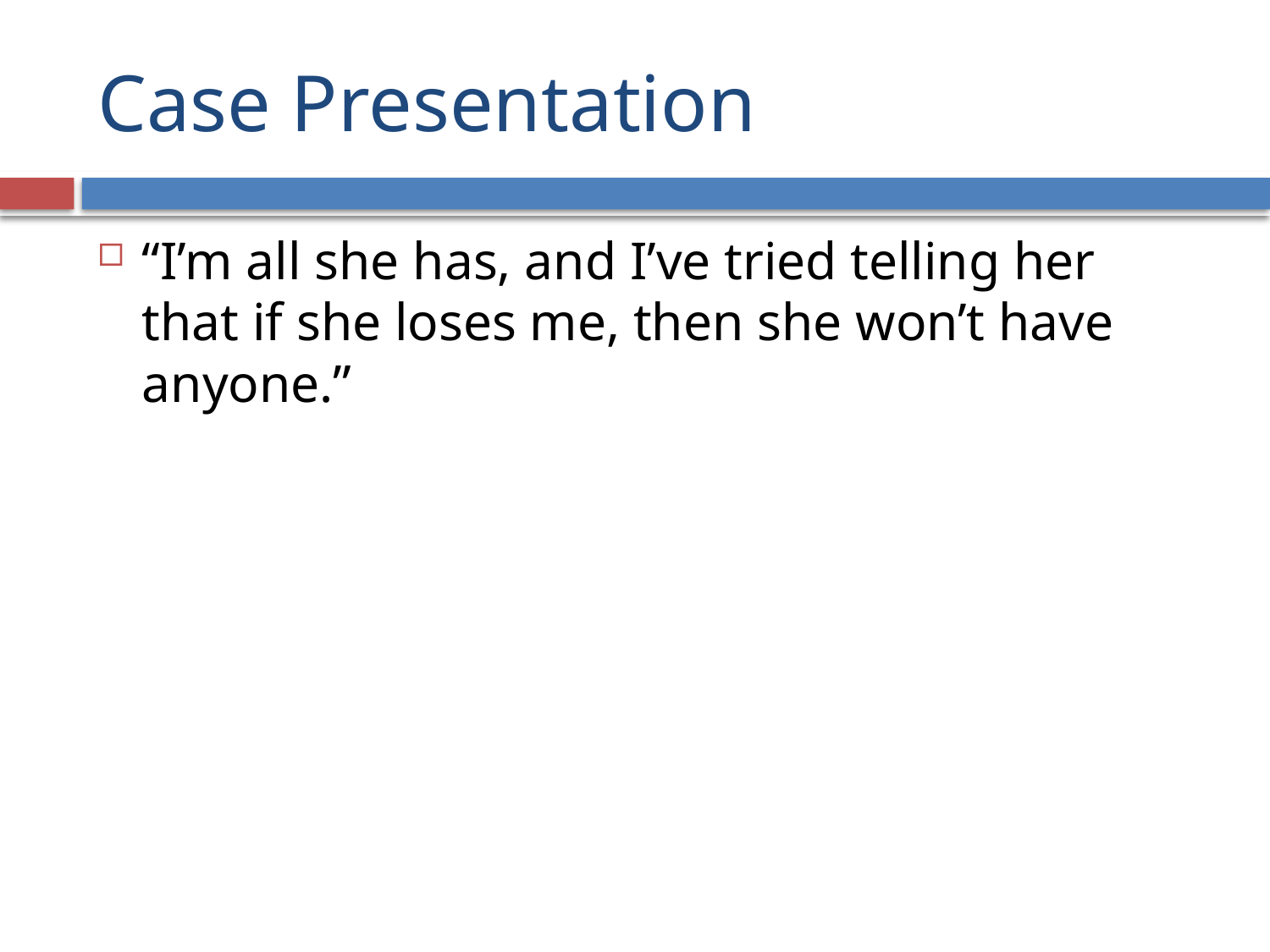

# Case Presentation
“I’m all she has, and I’ve tried telling her that if she loses me, then she won’t have anyone.”

## Slide 8
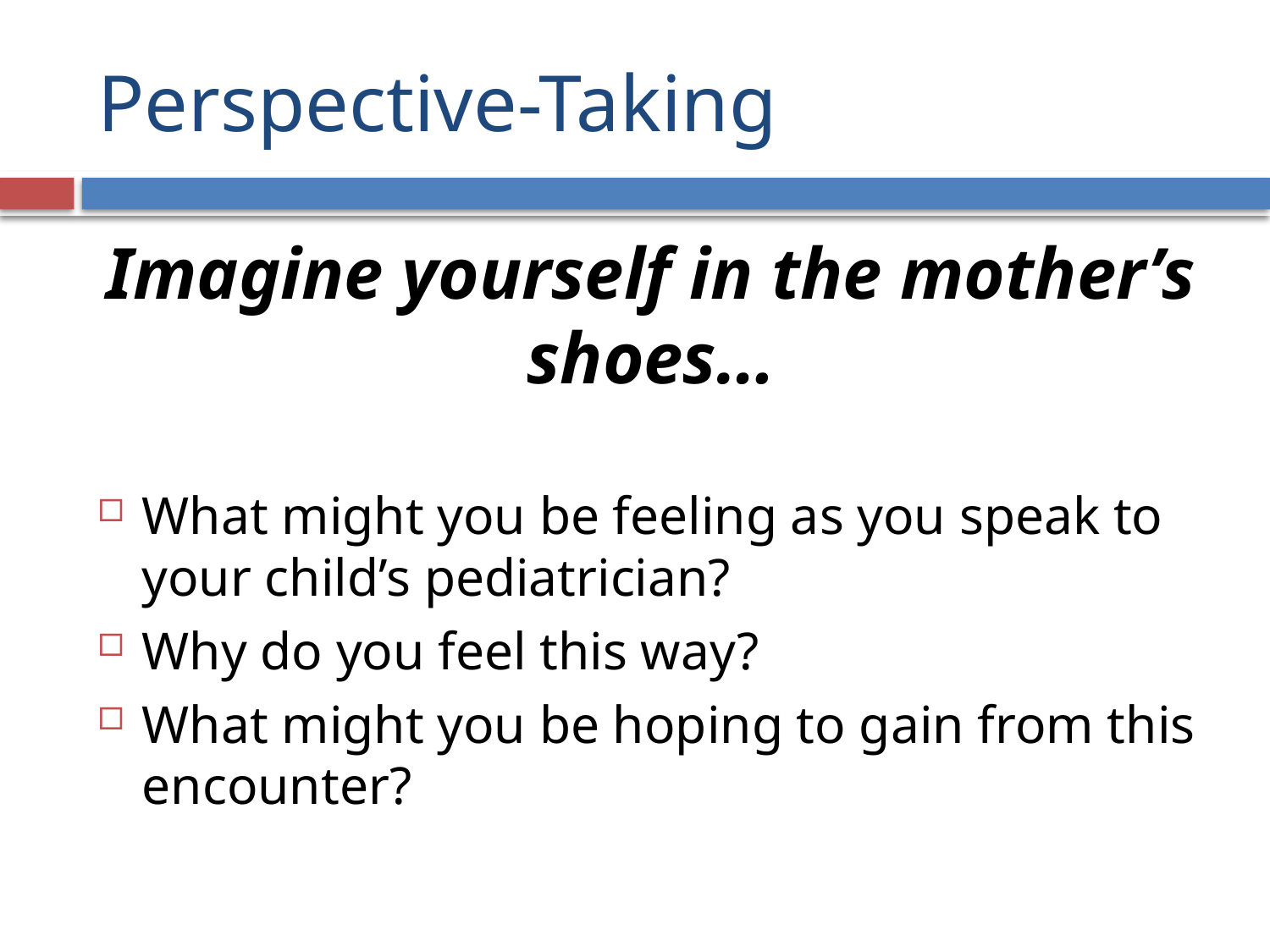

# Perspective-Taking
Imagine yourself in the mother’s shoes…
What might you be feeling as you speak to your child’s pediatrician?
Why do you feel this way?
What might you be hoping to gain from this encounter?

## Slide 9
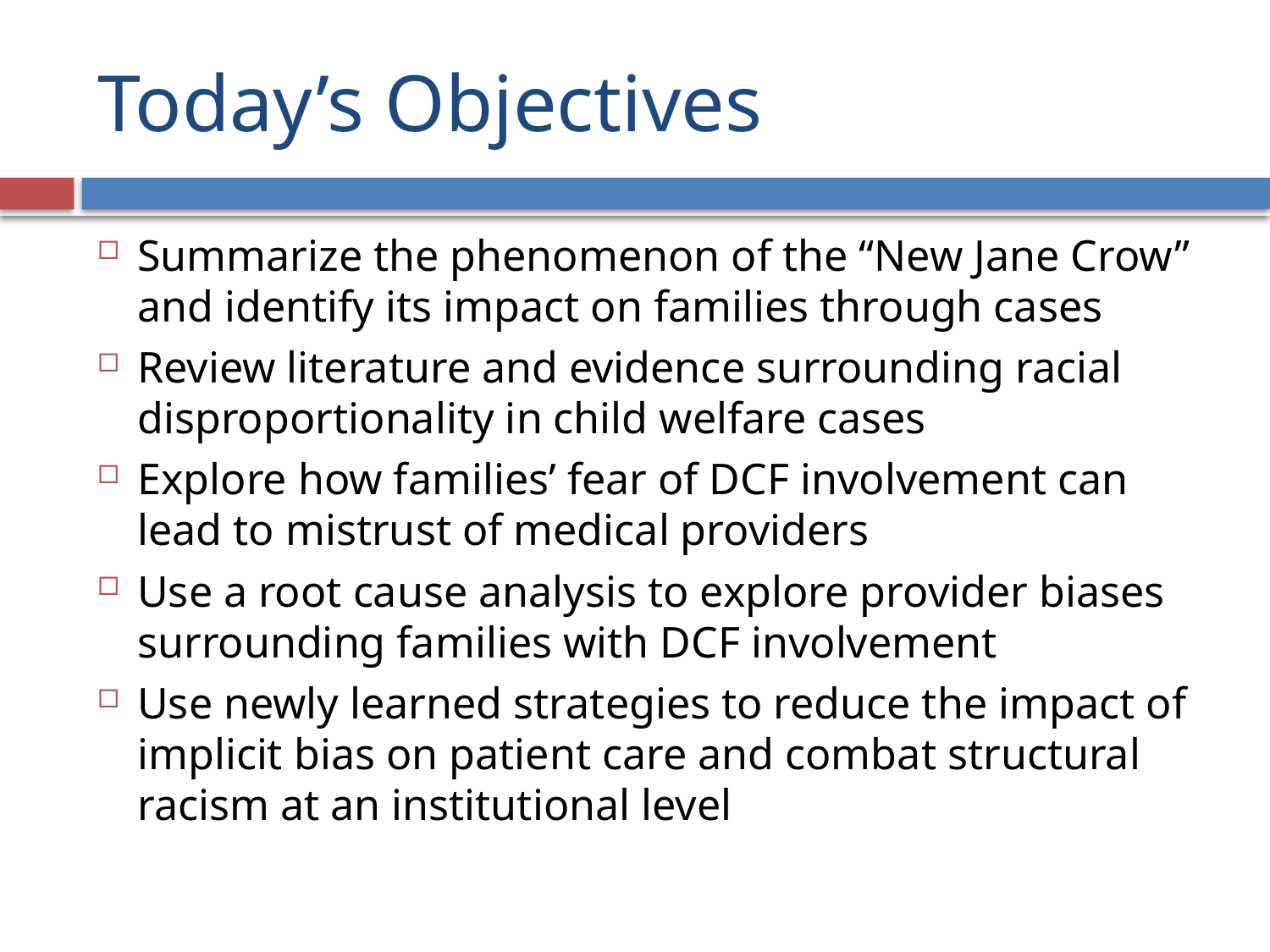

# Today’s Objectives
Summarize the phenomenon of the “New Jane Crow” and identify its impact on families through cases
Review literature and evidence surrounding racial disproportionality in child welfare cases
Explore how families’ fear of DCF involvement can lead to mistrust of medical providers
Use a root cause analysis to explore provider biases surrounding families with DCF involvement
Use newly learned strategies to reduce the impact of implicit bias on patient care and combat structural racism at an institutional level

## Slide 10
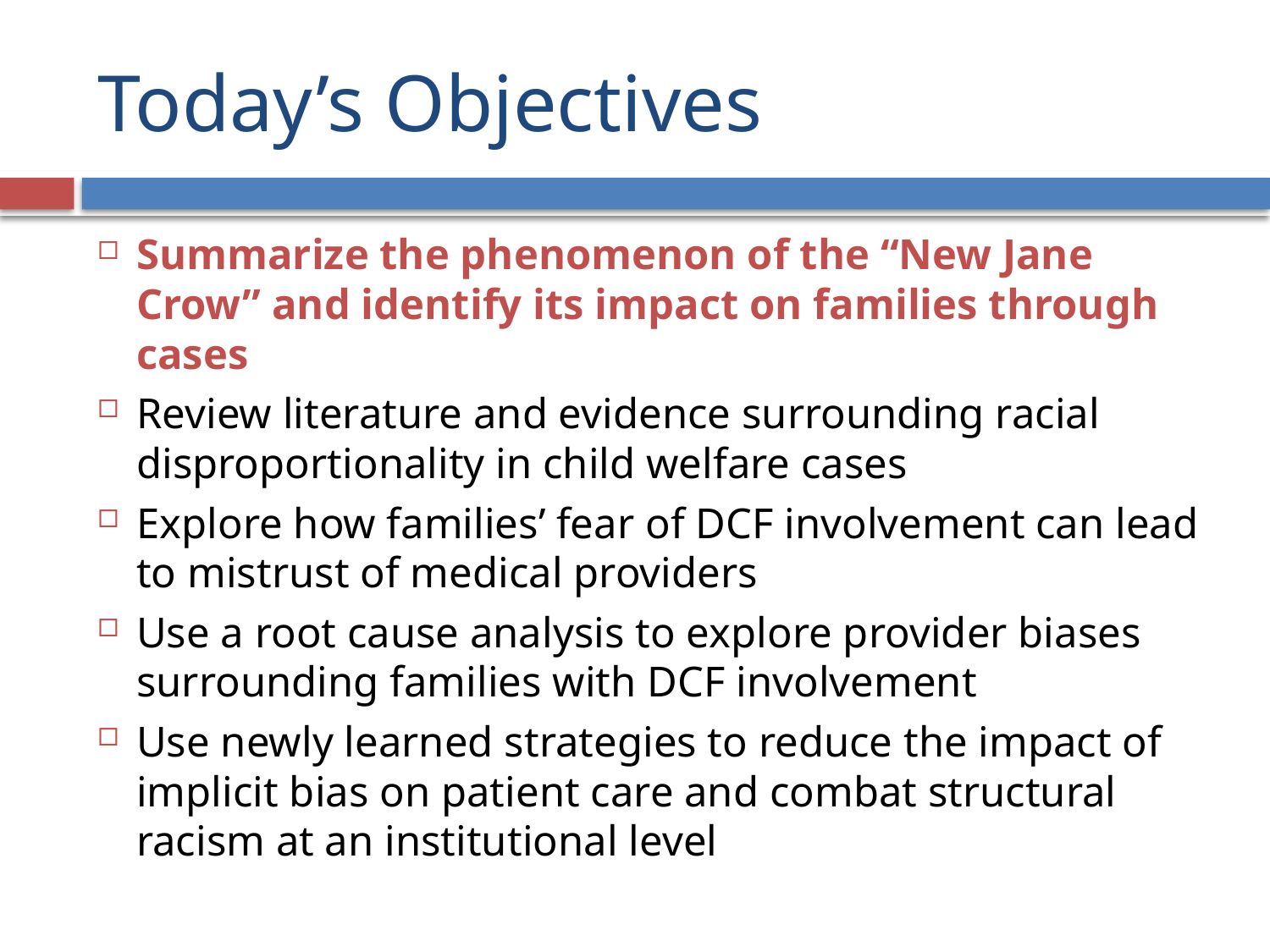

# Today’s Objectives
Summarize the phenomenon of the “New Jane Crow” and identify its impact on families through cases
Review literature and evidence surrounding racial disproportionality in child welfare cases
Explore how families’ fear of DCF involvement can lead to mistrust of medical providers
Use a root cause analysis to explore provider biases surrounding families with DCF involvement
Use newly learned strategies to reduce the impact of implicit bias on patient care and combat structural racism at an institutional level

## Slide 11
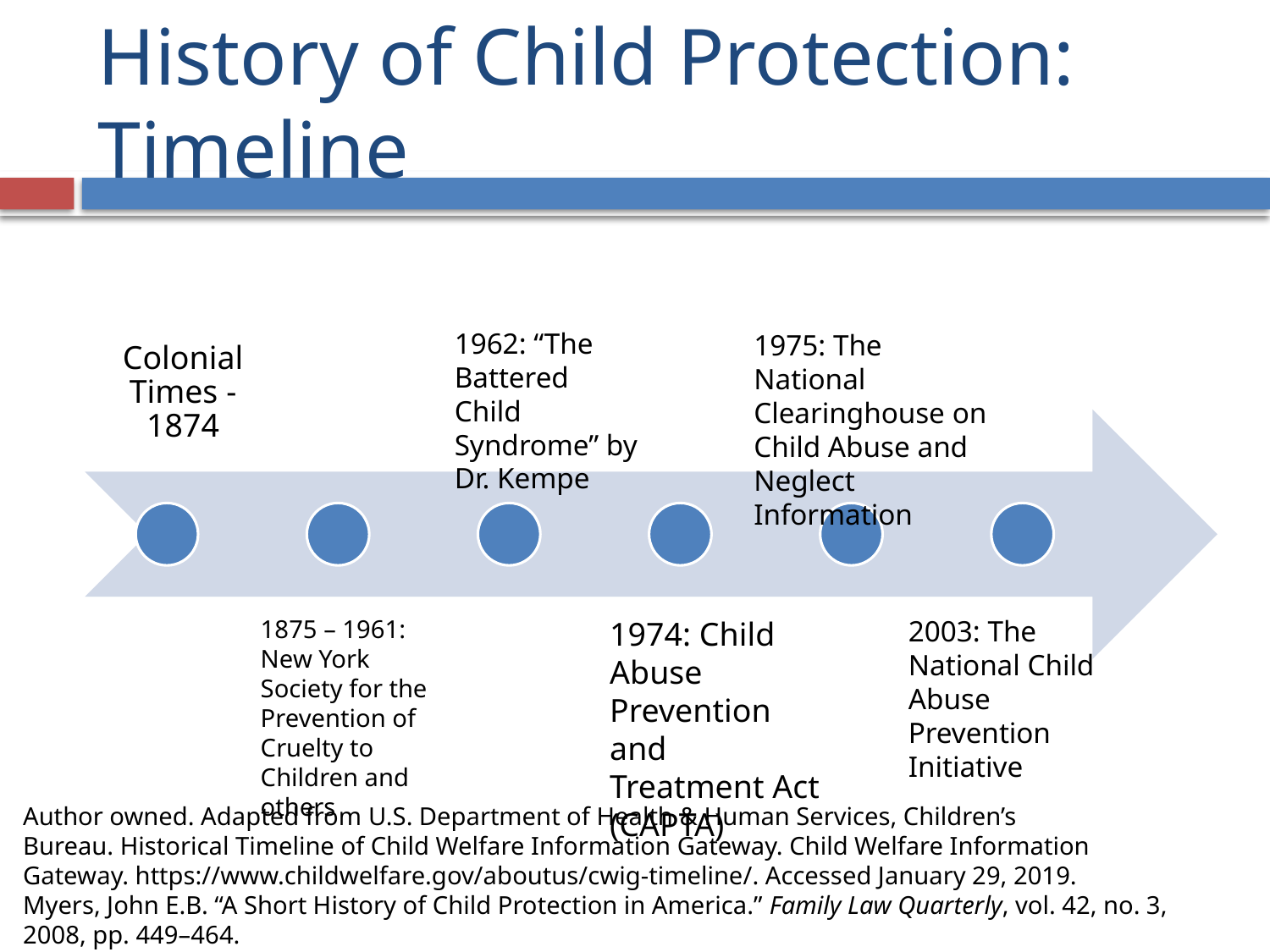

# History of Child Protection: Timeline
1962: “The Battered Child Syndrome” by Dr. Kempe
1975: The National Clearinghouse on Child Abuse and Neglect Information
Colonial Times - 1874
1875 – 1961: New York Society for the Prevention of Cruelty to Children and others
2003: The National Child Abuse Prevention Initiative
1974: Child Abuse Prevention and Treatment Act (CAPTA)
Author owned. Adapted from U.S. Department of Health & Human Services, Children’s Bureau. Historical Timeline of Child Welfare Information Gateway. Child Welfare Information Gateway. https://www.childwelfare.gov/aboutus/cwig-timeline/. Accessed January 29, 2019.
Myers, John E.B. “A Short History of Child Protection in America.” Family Law Quarterly, vol. 42, no. 3, 2008, pp. 449–464.

## Slide 12
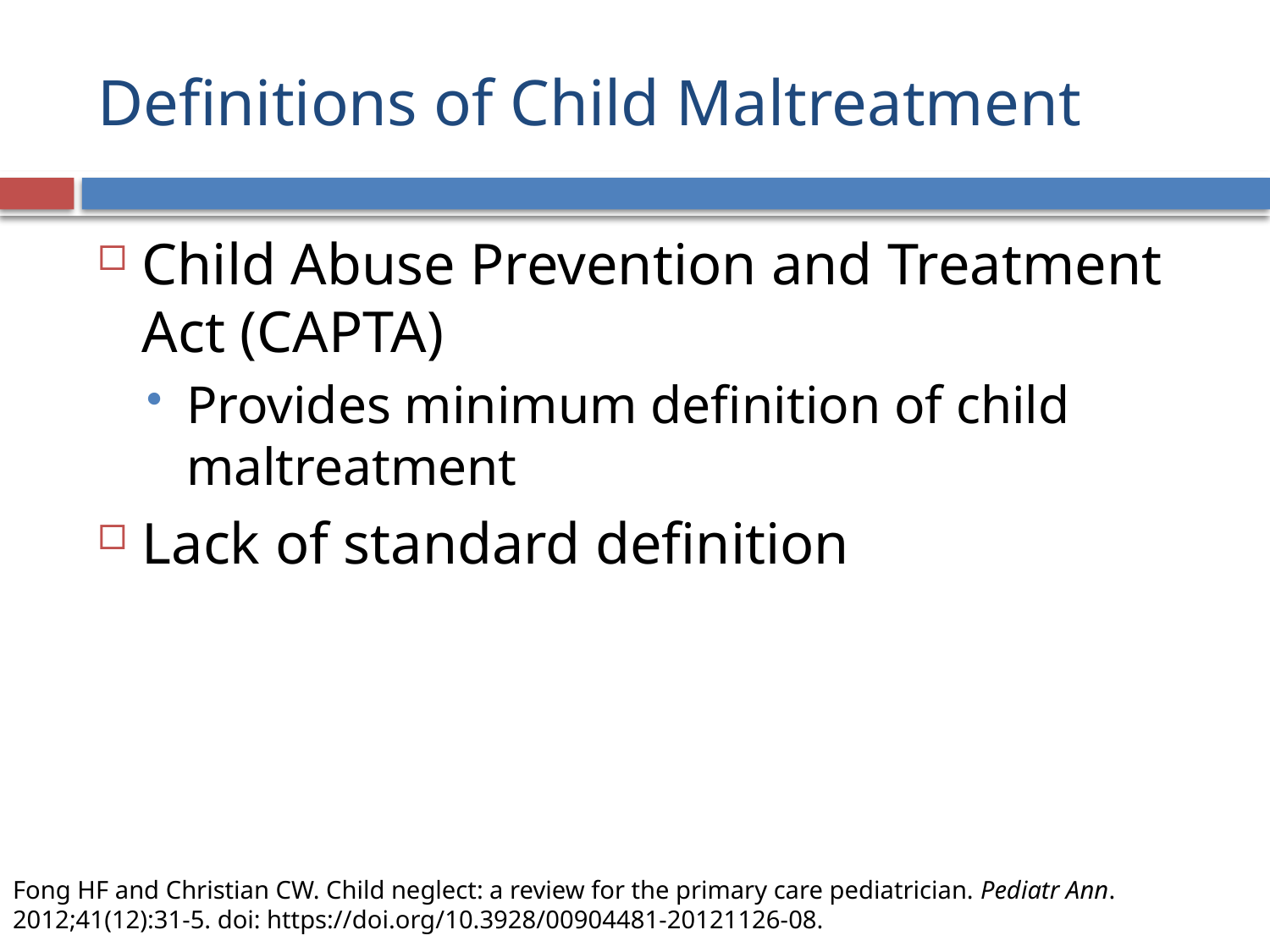

# Definitions of Child Maltreatment
Child Abuse Prevention and Treatment Act (CAPTA)
Provides minimum definition of child maltreatment
Lack of standard definition
Fong HF and Christian CW. Child neglect: a review for the primary care pediatrician. Pediatr Ann. 2012;41(12):31-5. doi: https://doi.org/10.3928/00904481-20121126-08.

## Slide 13
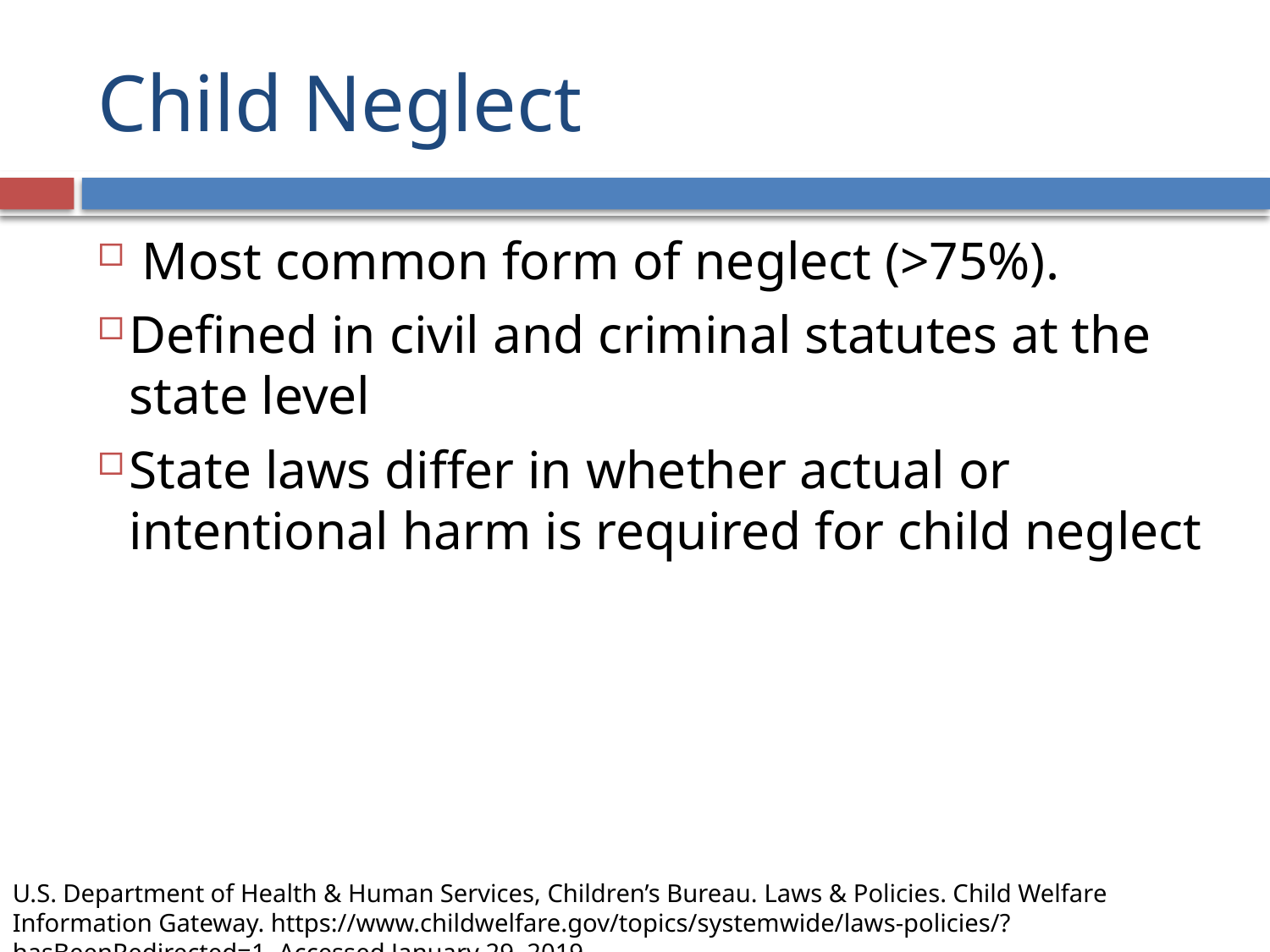

# Child Neglect
Most common form of neglect (>75%).
Defined in civil and criminal statutes at the state level
State laws differ in whether actual or intentional harm is required for child neglect
U.S. Department of Health & Human Services, Children’s Bureau. Laws & Policies. Child Welfare Information Gateway. https://www.childwelfare.gov/topics/systemwide/laws-policies/?hasBeenRedirected=1. Accessed January 29, 2019.

## Slide 14
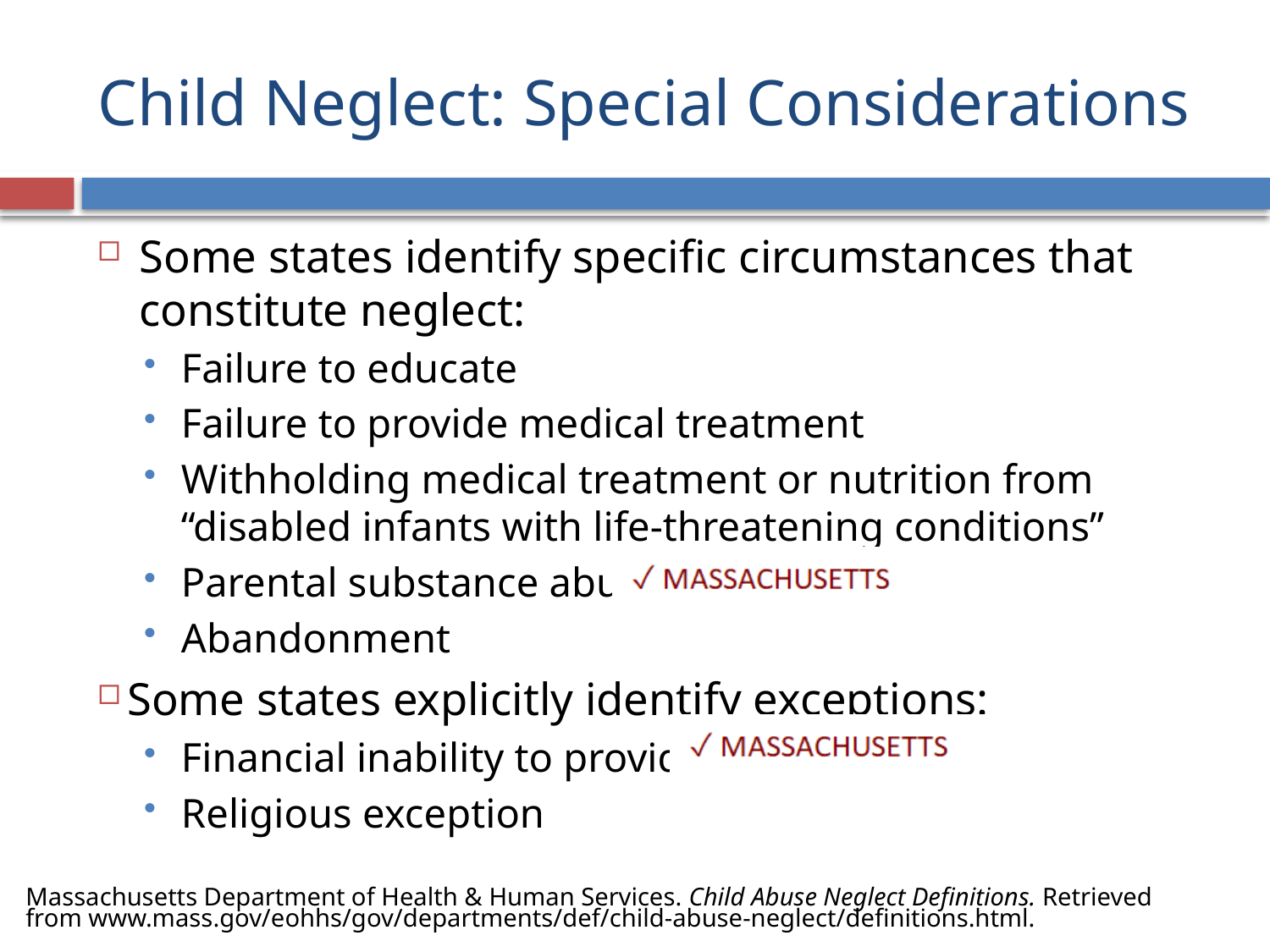

# Child Neglect: Special Considerations
Some states identify specific circumstances that constitute neglect:
Failure to educate
Failure to provide medical treatment
Withholding medical treatment or nutrition from “disabled infants with life-threatening conditions”
Parental substance abuse
Abandonment
Some states explicitly identify exceptions:
Financial inability to provide for child
Religious exception
Massachusetts Department of Health & Human Services. Child Abuse Neglect Definitions. Retrieved from www.mass.gov/eohhs/gov/departments/def/child-abuse-neglect/definitions.html.

## Slide 15
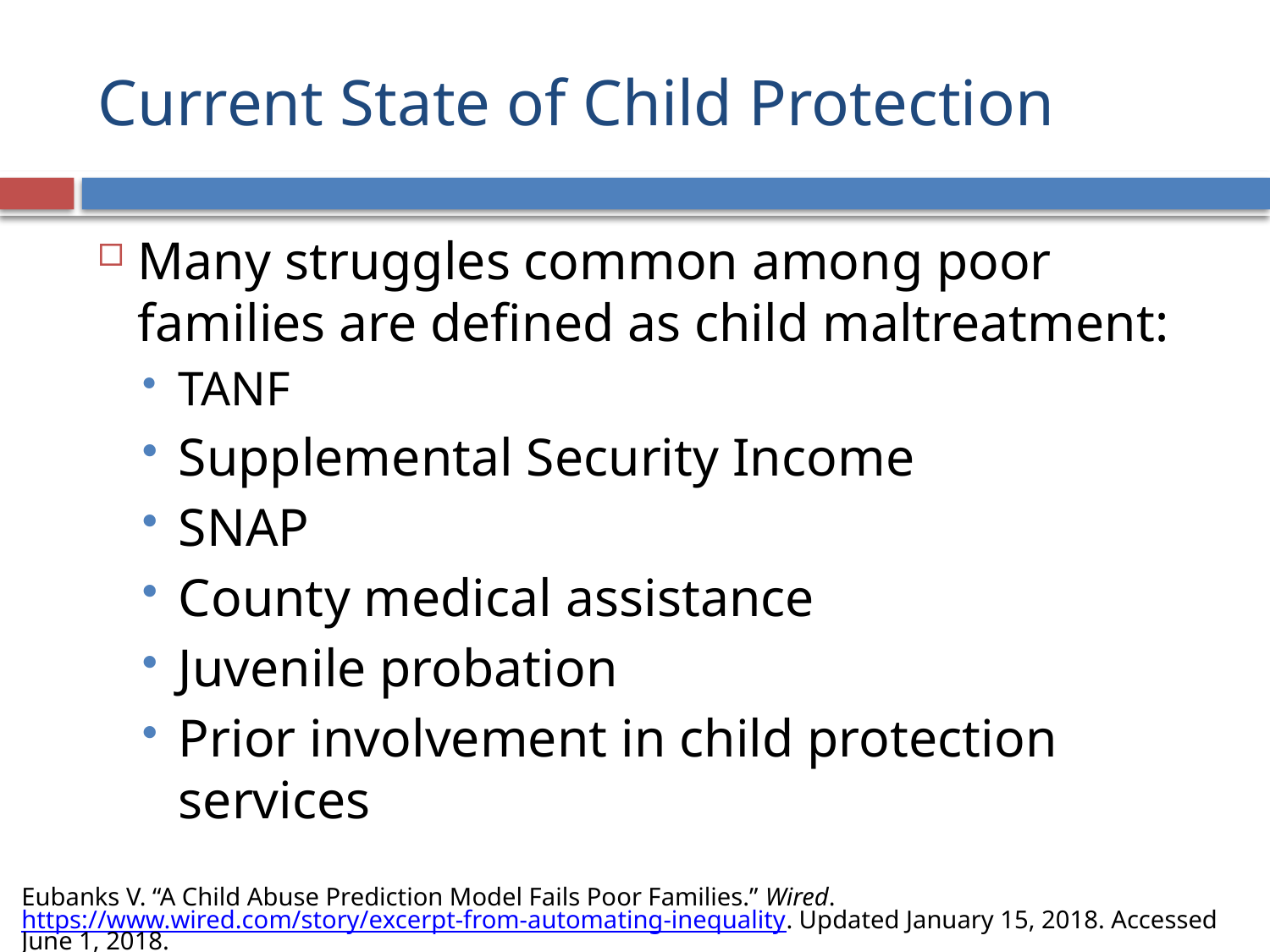

# Current State of Child Protection
Many struggles common among poor families are defined as child maltreatment:
TANF
Supplemental Security Income
SNAP
County medical assistance
Juvenile probation
Prior involvement in child protection services
Eubanks V. “A Child Abuse Prediction Model Fails Poor Families.” Wired. https://www.wired.com/story/excerpt-from-automating-inequality. Updated January 15, 2018. Accessed June 1, 2018.

## Slide 16
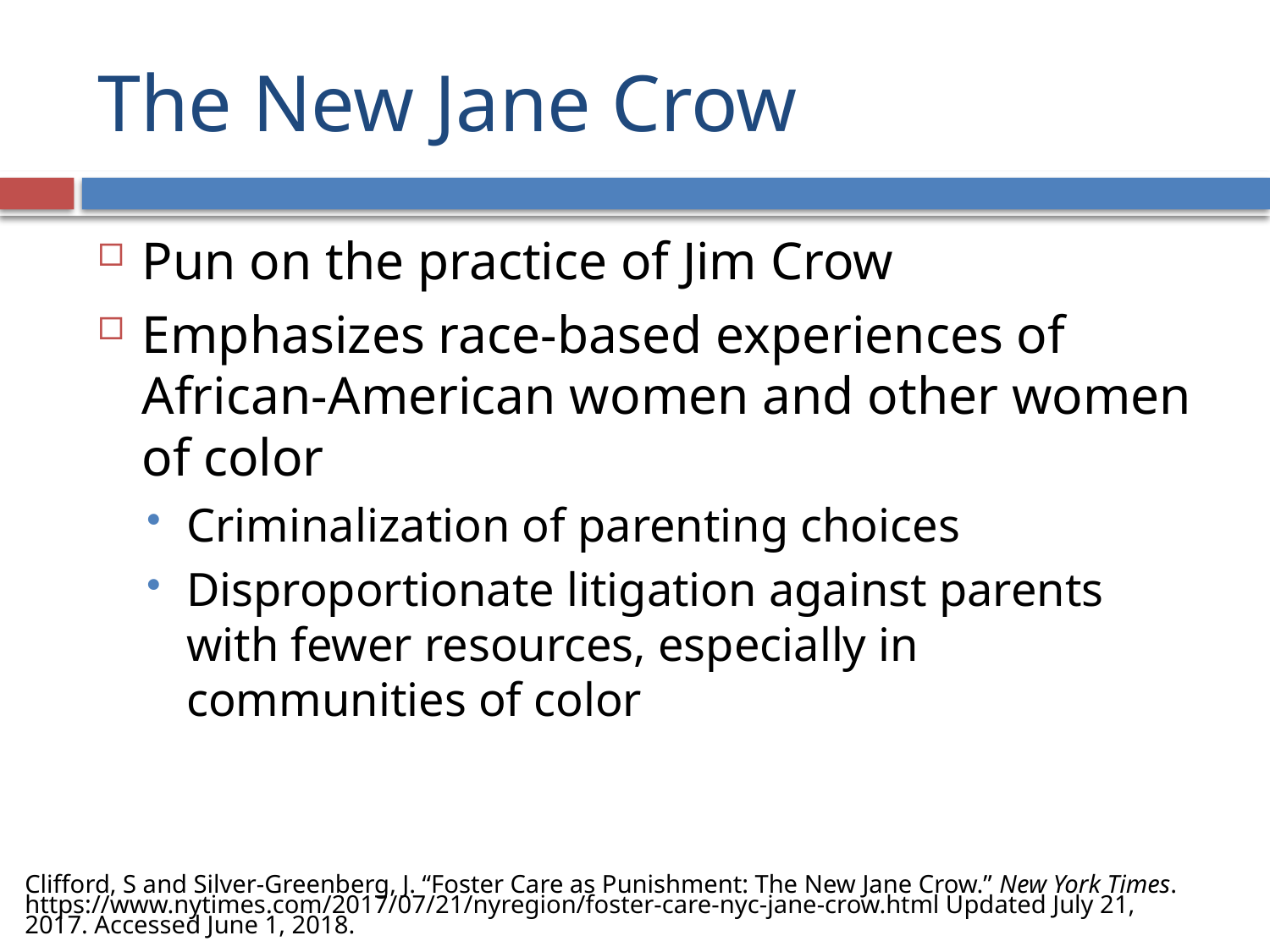

# The New Jane Crow
Pun on the practice of Jim Crow
Emphasizes race-based experiences of African-American women and other women of color
Criminalization of parenting choices
Disproportionate litigation against parents with fewer resources, especially in communities of color
Clifford, S and Silver-Greenberg, J. “Foster Care as Punishment: The New Jane Crow.” New York Times. https://www.nytimes.com/2017/07/21/nyregion/foster-care-nyc-jane-crow.html Updated July 21, 2017. Accessed June 1, 2018.

## Slide 17
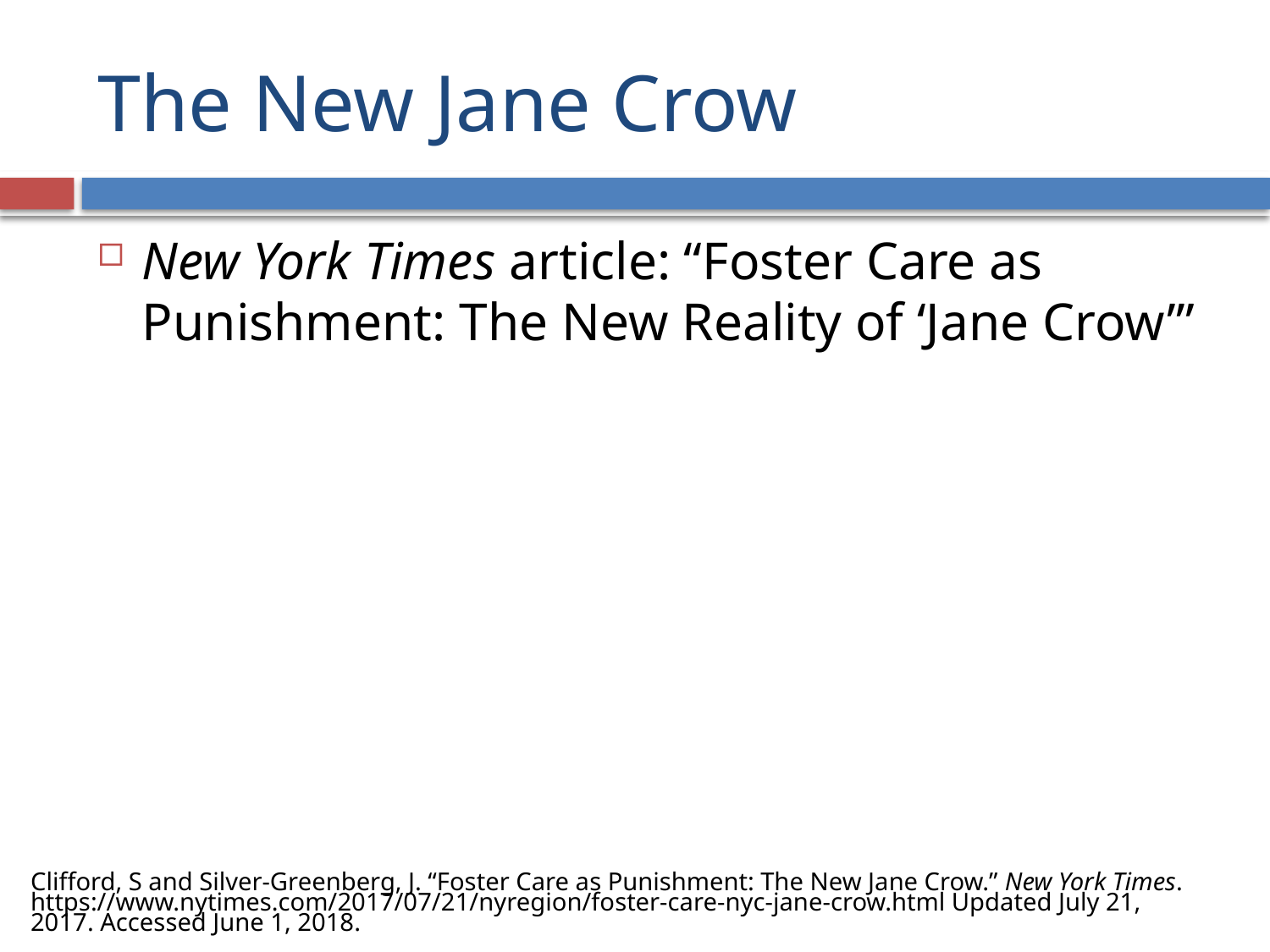

# The New Jane Crow
New York Times article: “Foster Care as Punishment: The New Reality of ‘Jane Crow’”
Clifford, S and Silver-Greenberg, J. “Foster Care as Punishment: The New Jane Crow.” New York Times. https://www.nytimes.com/2017/07/21/nyregion/foster-care-nyc-jane-crow.html Updated July 21, 2017. Accessed June 1, 2018.

## Slide 18
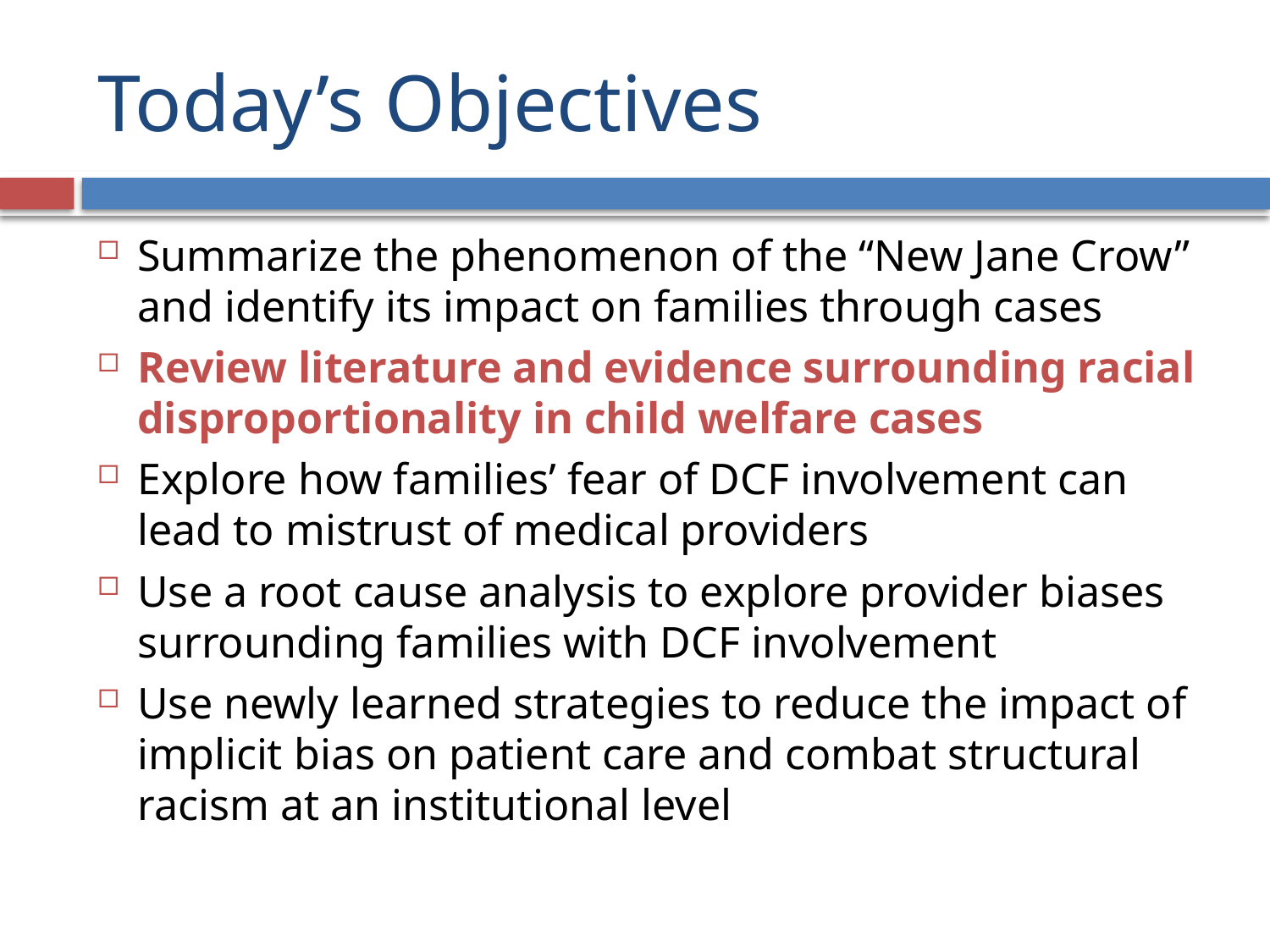

# Today’s Objectives
Summarize the phenomenon of the “New Jane Crow” and identify its impact on families through cases
Review literature and evidence surrounding racial disproportionality in child welfare cases
Explore how families’ fear of DCF involvement can lead to mistrust of medical providers
Use a root cause analysis to explore provider biases surrounding families with DCF involvement
Use newly learned strategies to reduce the impact of implicit bias on patient care and combat structural racism at an institutional level

## Slide 19
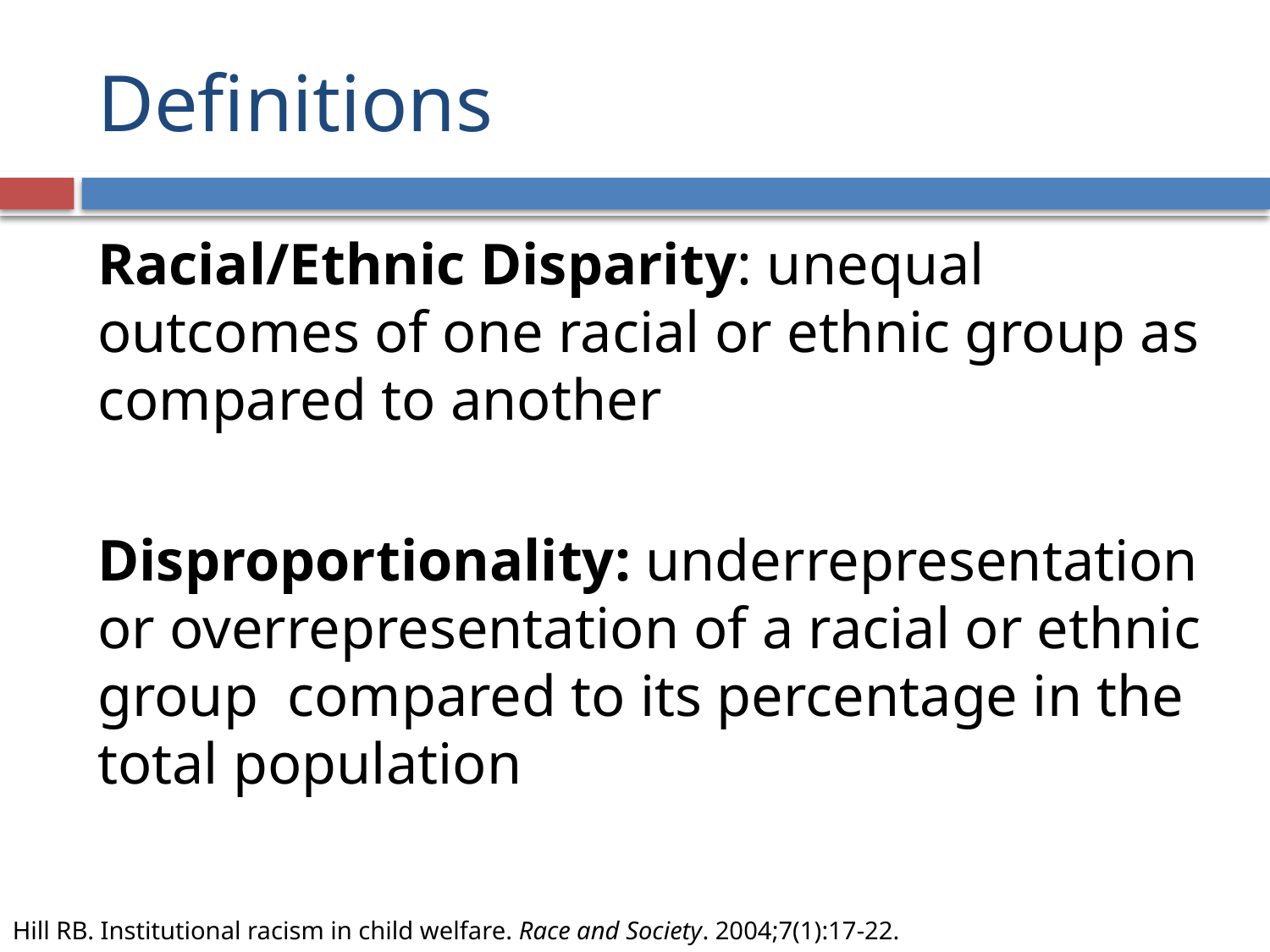

# Definitions
Racial/Ethnic Disparity: unequal outcomes of one racial or ethnic group as compared to another
Disproportionality: underrepresentation or overrepresentation of a racial or ethnic group compared to its percentage in the total population
Hill RB. Institutional racism in child welfare. Race and Society. 2004;7(1):17-22.

## Slide 20
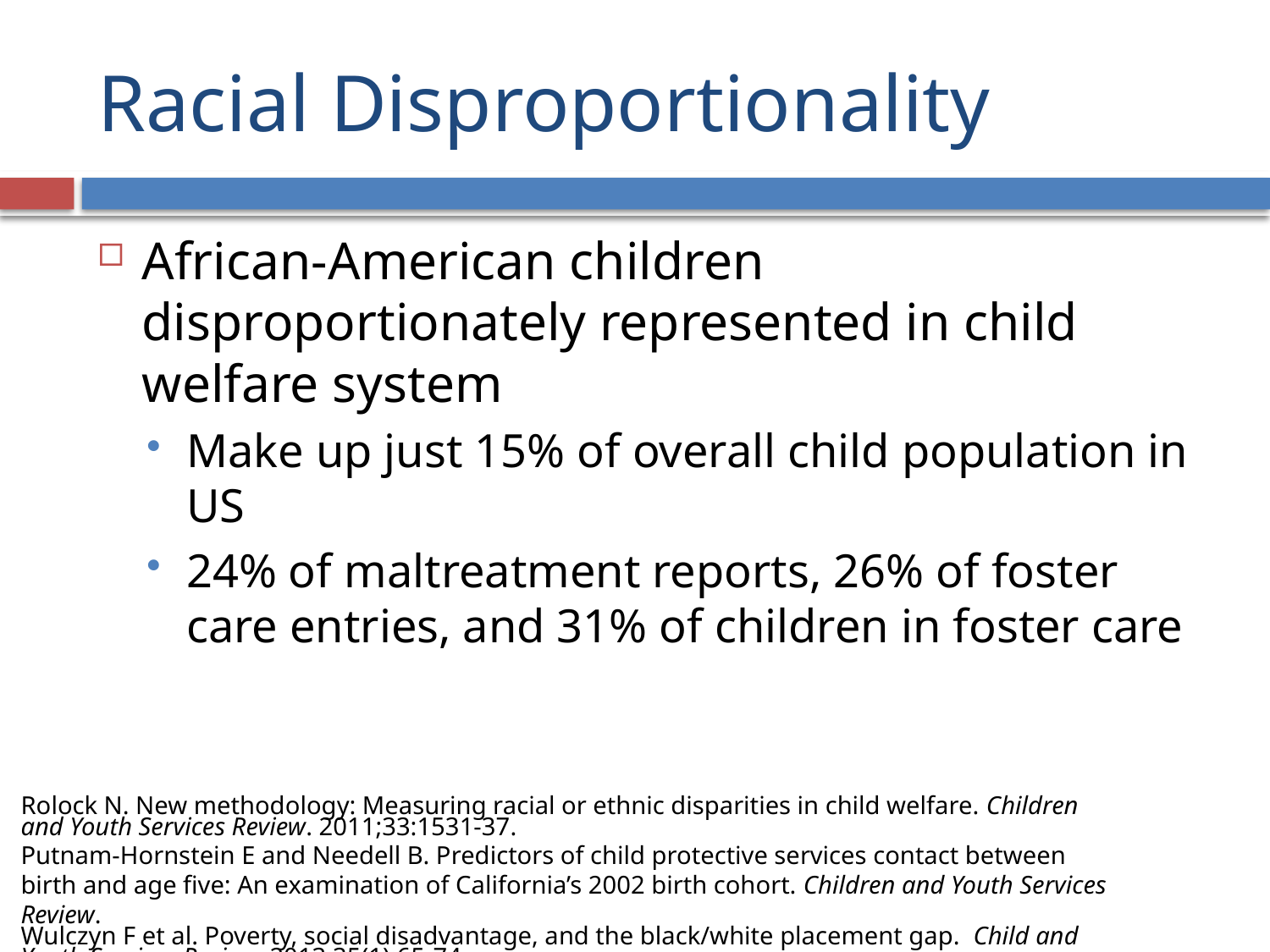

# Racial Disproportionality
African-American children disproportionately represented in child welfare system
Make up just 15% of overall child population in US
24% of maltreatment reports, 26% of foster care entries, and 31% of children in foster care
Rolock N. New methodology: Measuring racial or ethnic disparities in child welfare. Children and Youth Services Review. 2011;33:1531-37.
Putnam-Hornstein E and Needell B. Predictors of child protective services contact between birth and age five: An examination of California’s 2002 birth cohort. Children and Youth Services Review.
Wulczyn F et al. Poverty, social disadvantage, and the black/white placement gap.  Child and Youth Services Review. 2013;35(1):65-74.

## Slide 21
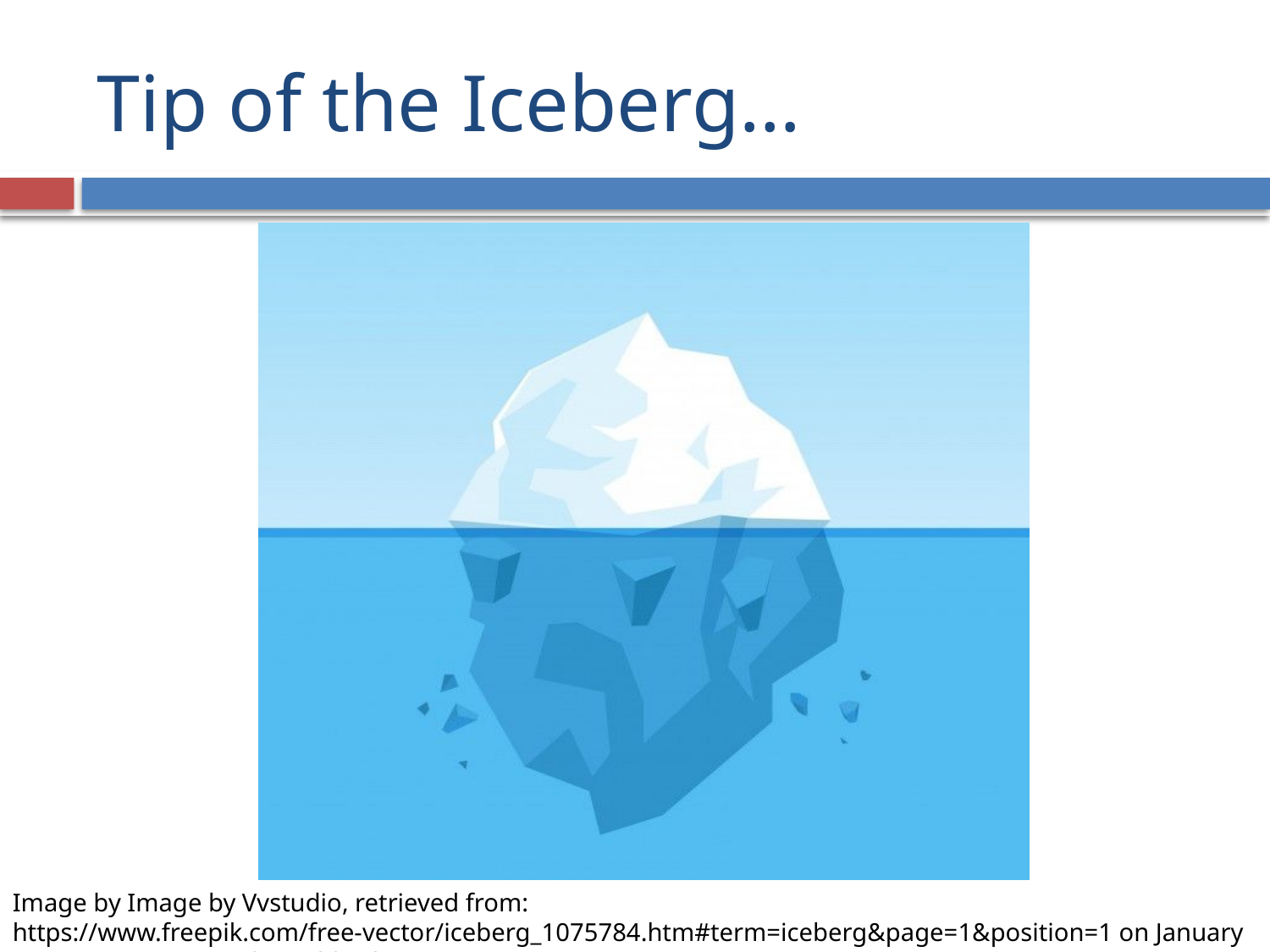

# Tip of the Iceberg…
Image by Image by Vvstudio, retrieved from: https://www.freepik.com/free-vector/iceberg_1075784.htm#term=iceberg&page=1&position=1 on January 6, 2019. Image is in the public domain.

## Slide 22
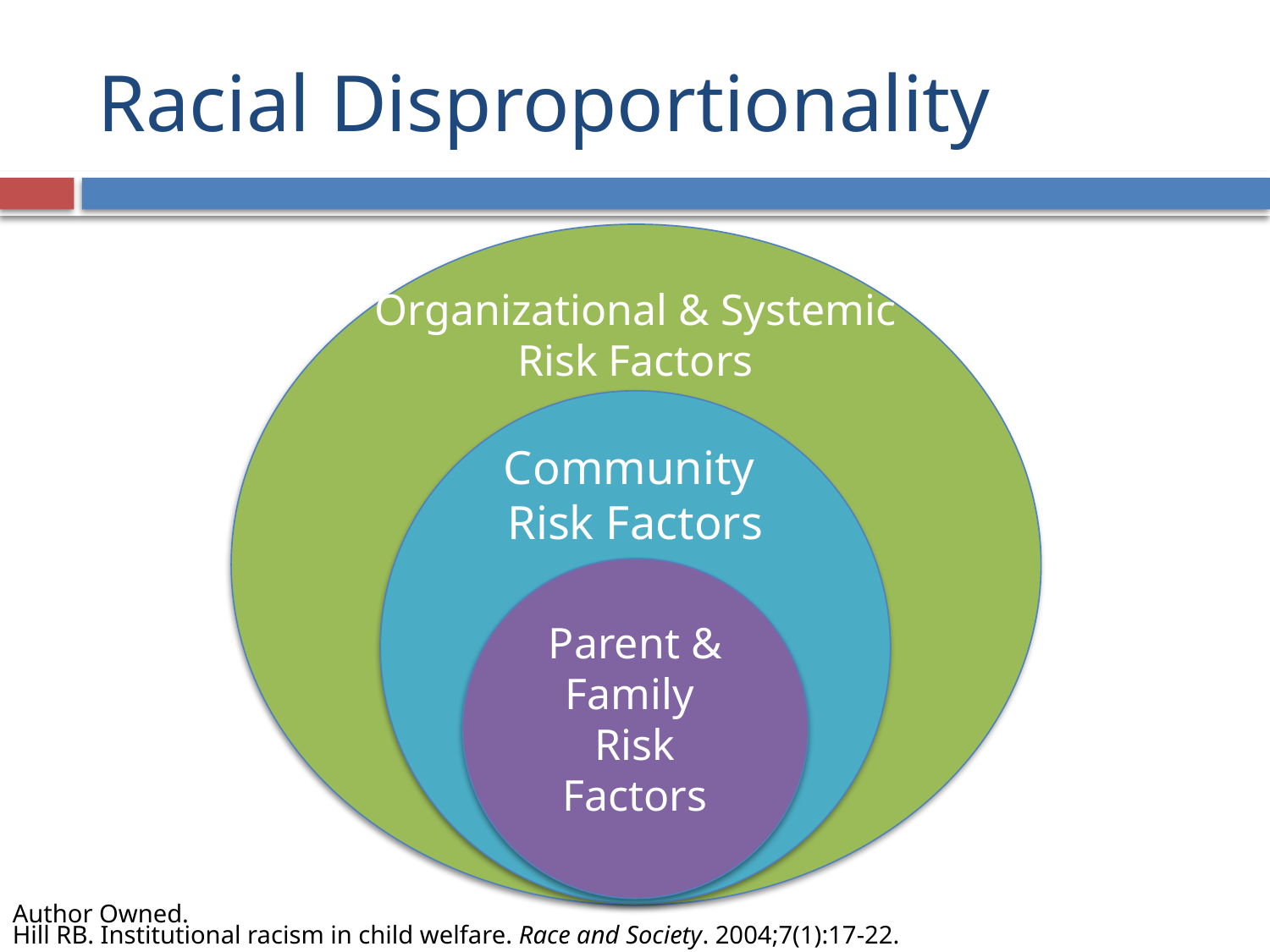

# Racial Disproportionality
Organizational & Systemic Risk Factors
Community
Risk Factors
Parent & Family
Risk Factors
Author Owned.
Hill RB. Institutional racism in child welfare. Race and Society. 2004;7(1):17-22.

## Slide 23
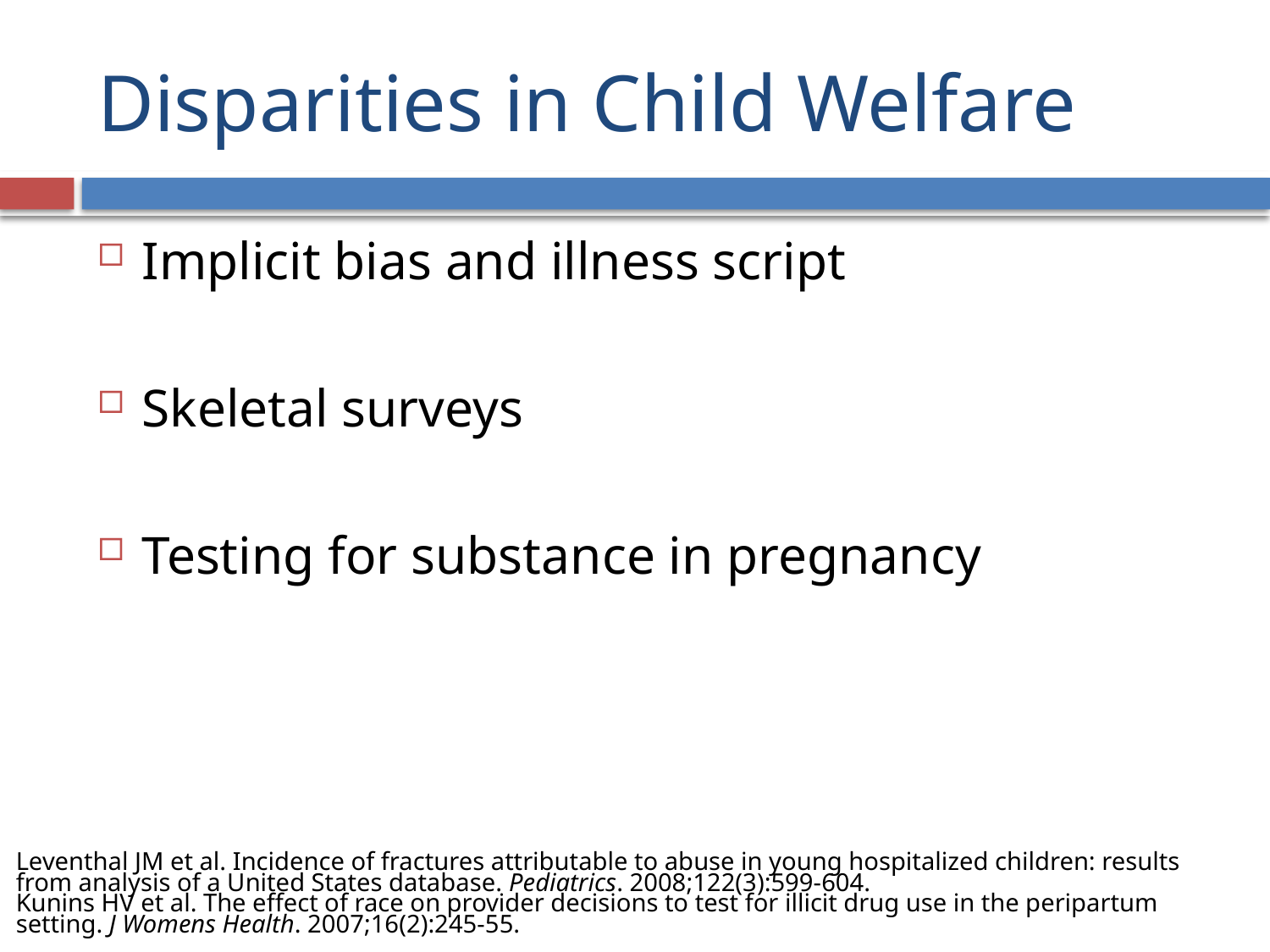

# Disparities in Child Welfare
Implicit bias and illness script
Skeletal surveys
Testing for substance in pregnancy
Leventhal JM et al. Incidence of fractures attributable to abuse in young hospitalized children: results from analysis of a United States database. Pediatrics. 2008;122(3):599-604.
Kunins HV et al. The effect of race on provider decisions to test for illicit drug use in the peripartum setting. J Womens Health. 2007;16(2):245-55.

## Slide 24
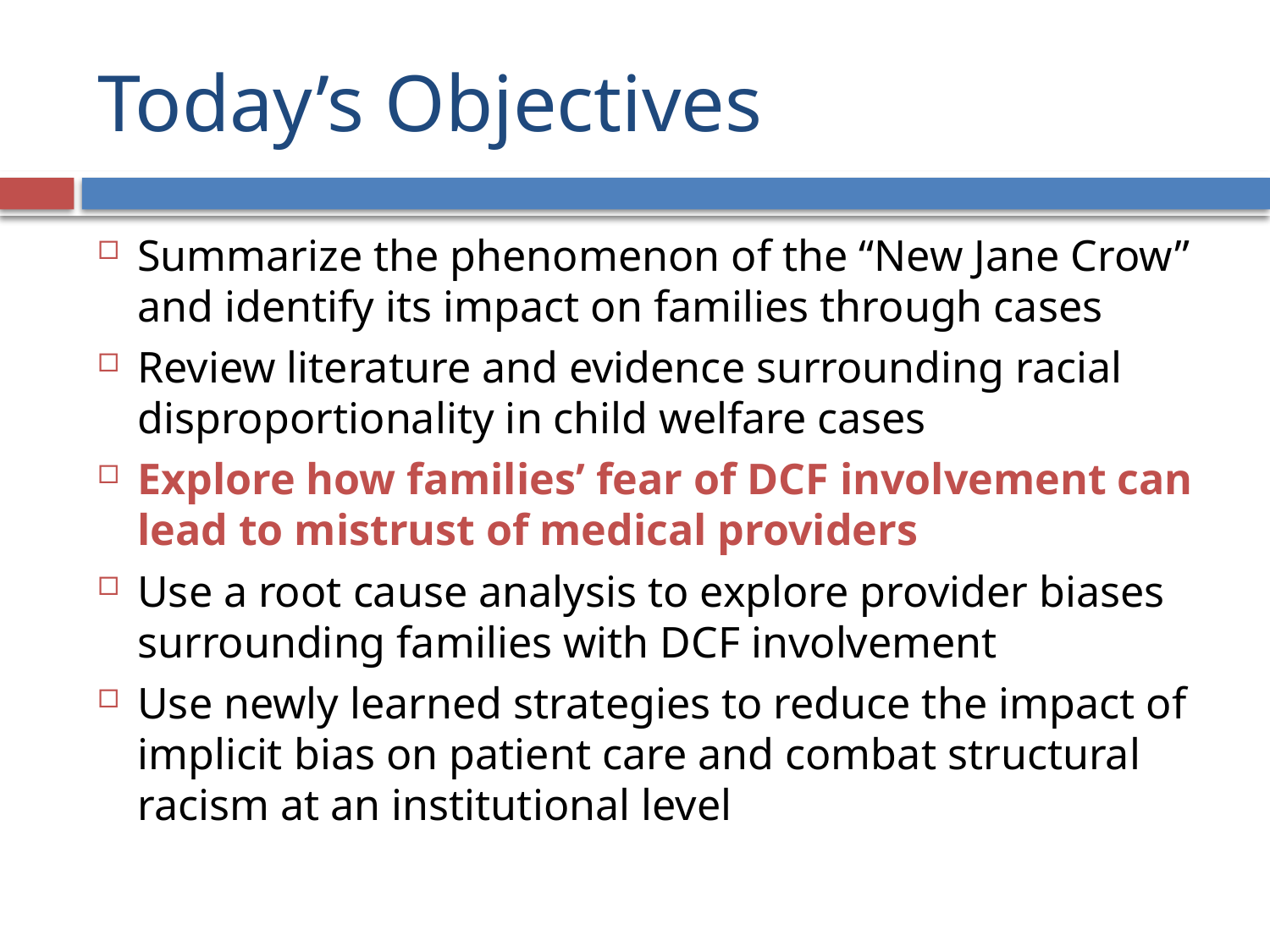

# Today’s Objectives
Summarize the phenomenon of the “New Jane Crow” and identify its impact on families through cases
Review literature and evidence surrounding racial disproportionality in child welfare cases
Explore how families’ fear of DCF involvement can lead to mistrust of medical providers
Use a root cause analysis to explore provider biases surrounding families with DCF involvement
Use newly learned strategies to reduce the impact of implicit bias on patient care and combat structural racism at an institutional level

## Slide 25
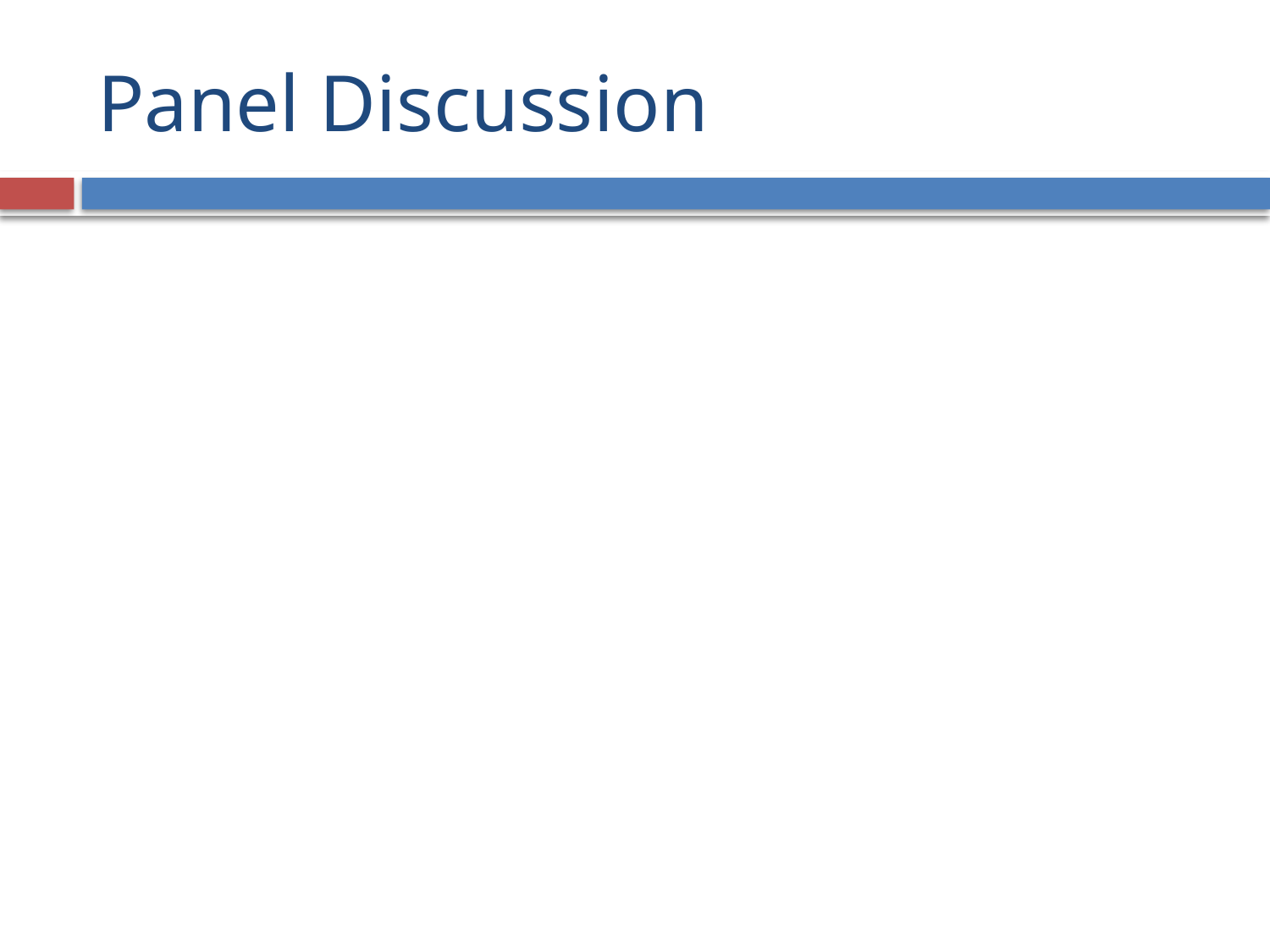

# Panel Discussion

## Slide 26
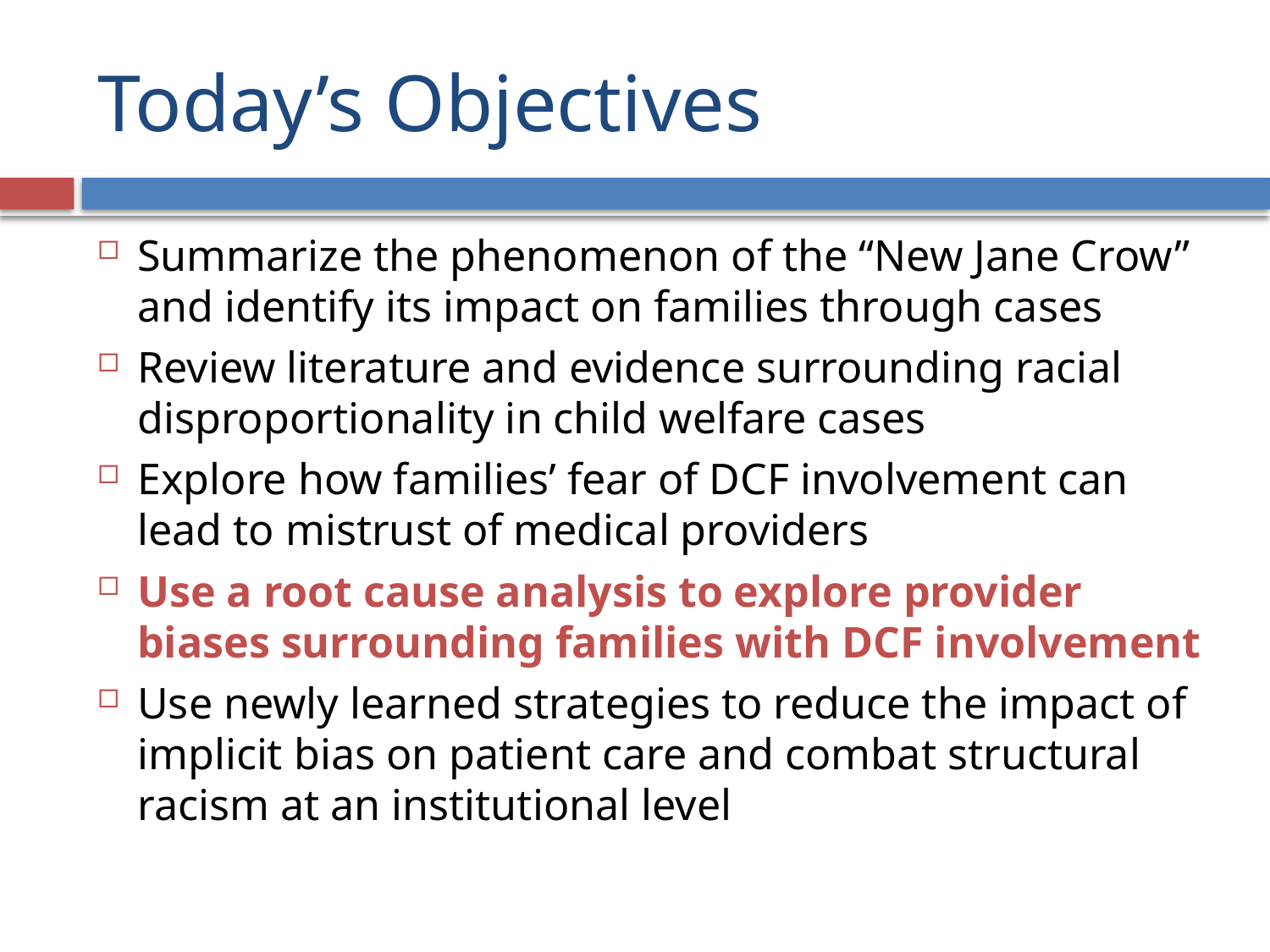

# Today’s Objectives
Summarize the phenomenon of the “New Jane Crow” and identify its impact on families through cases
Review literature and evidence surrounding racial disproportionality in child welfare cases
Explore how families’ fear of DCF involvement can lead to mistrust of medical providers
Use a root cause analysis to explore provider biases surrounding families with DCF involvement
Use newly learned strategies to reduce the impact of implicit bias on patient care and combat structural racism at an institutional level

## Slide 27
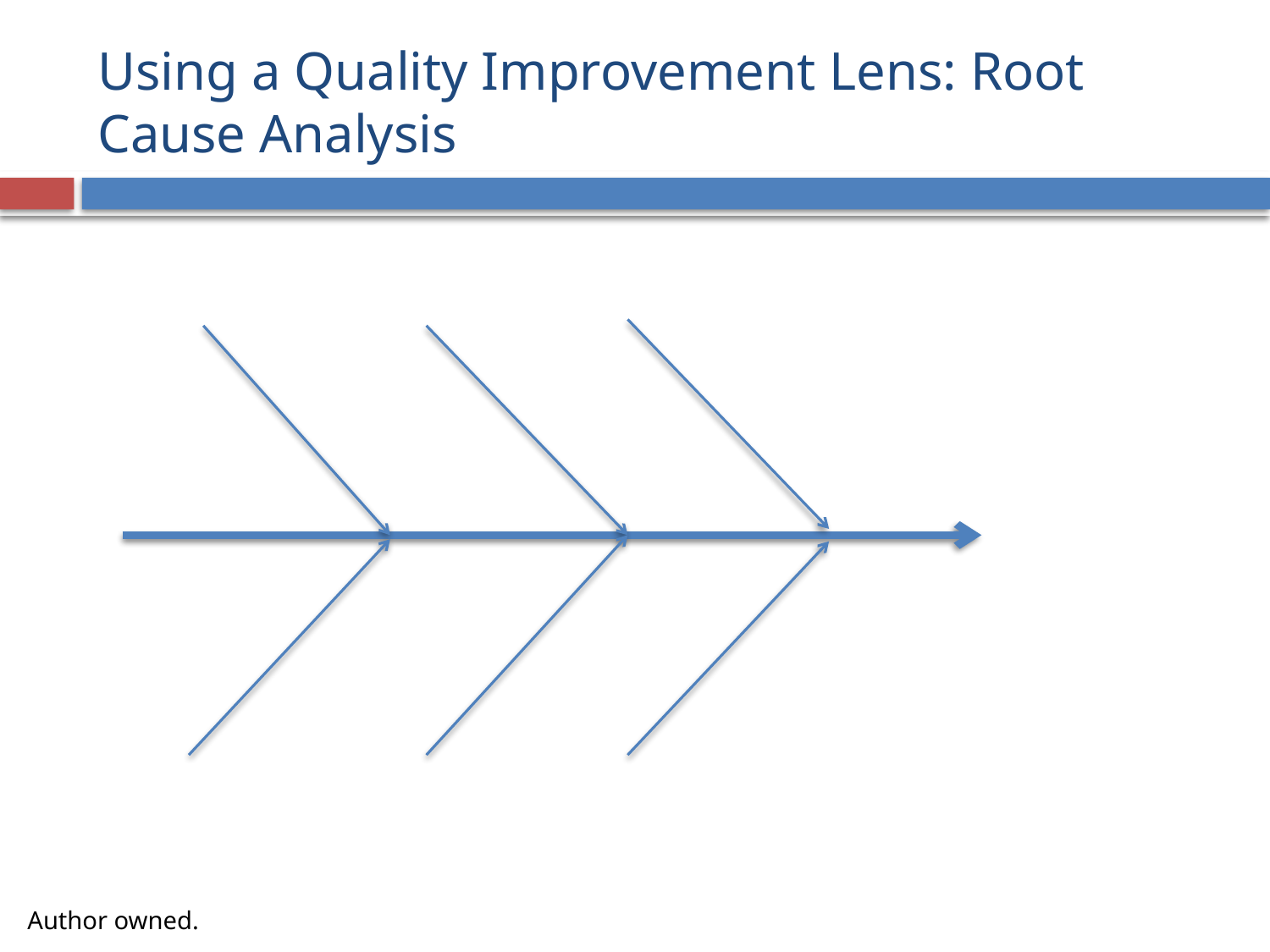

# Using a Quality Improvement Lens: Root Cause Analysis
Author owned.

## Slide 28
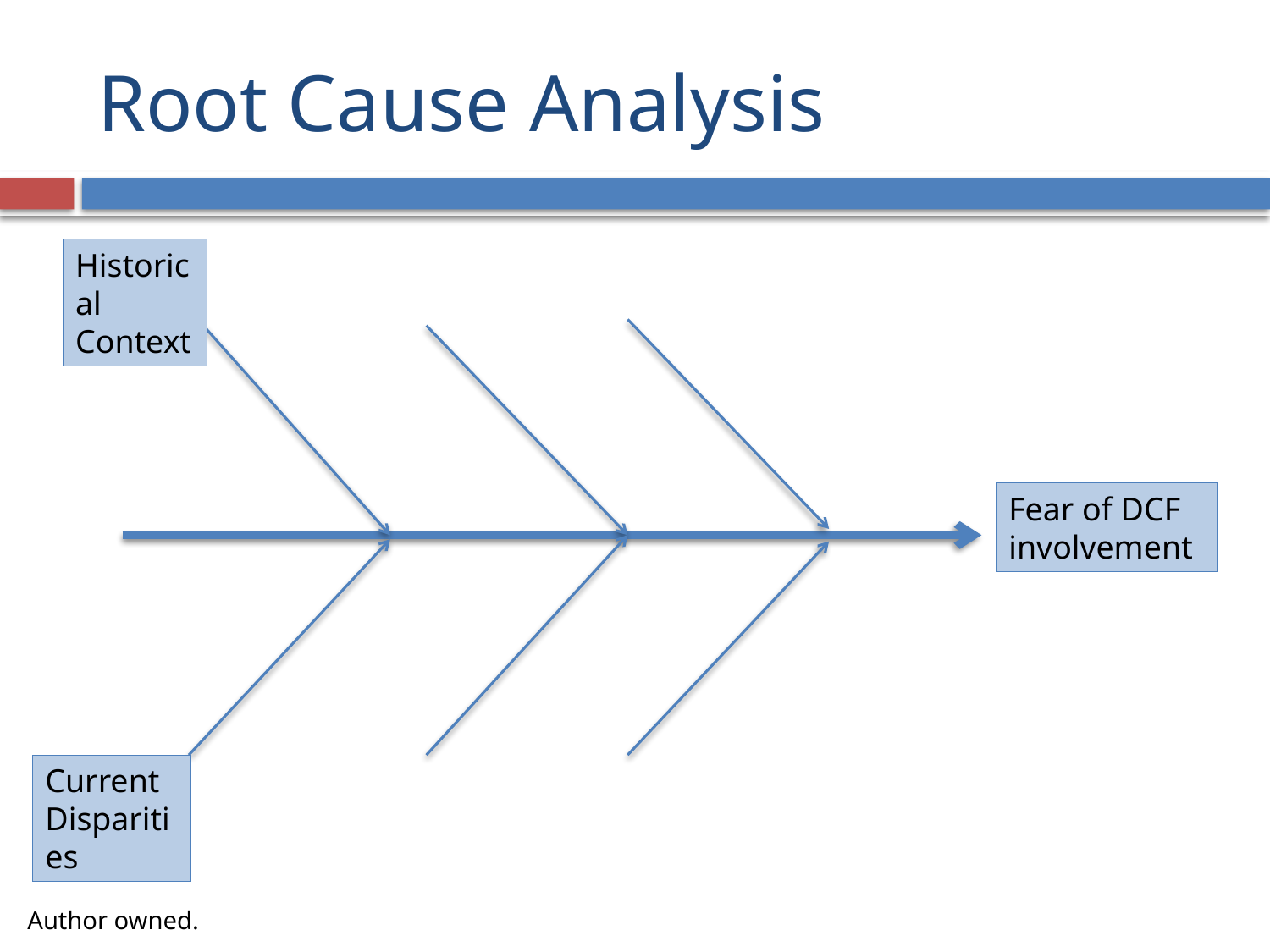

# Root Cause Analysis
Historical Context
Fear of DCF involvement
Current Disparities
Author owned.

## Slide 29
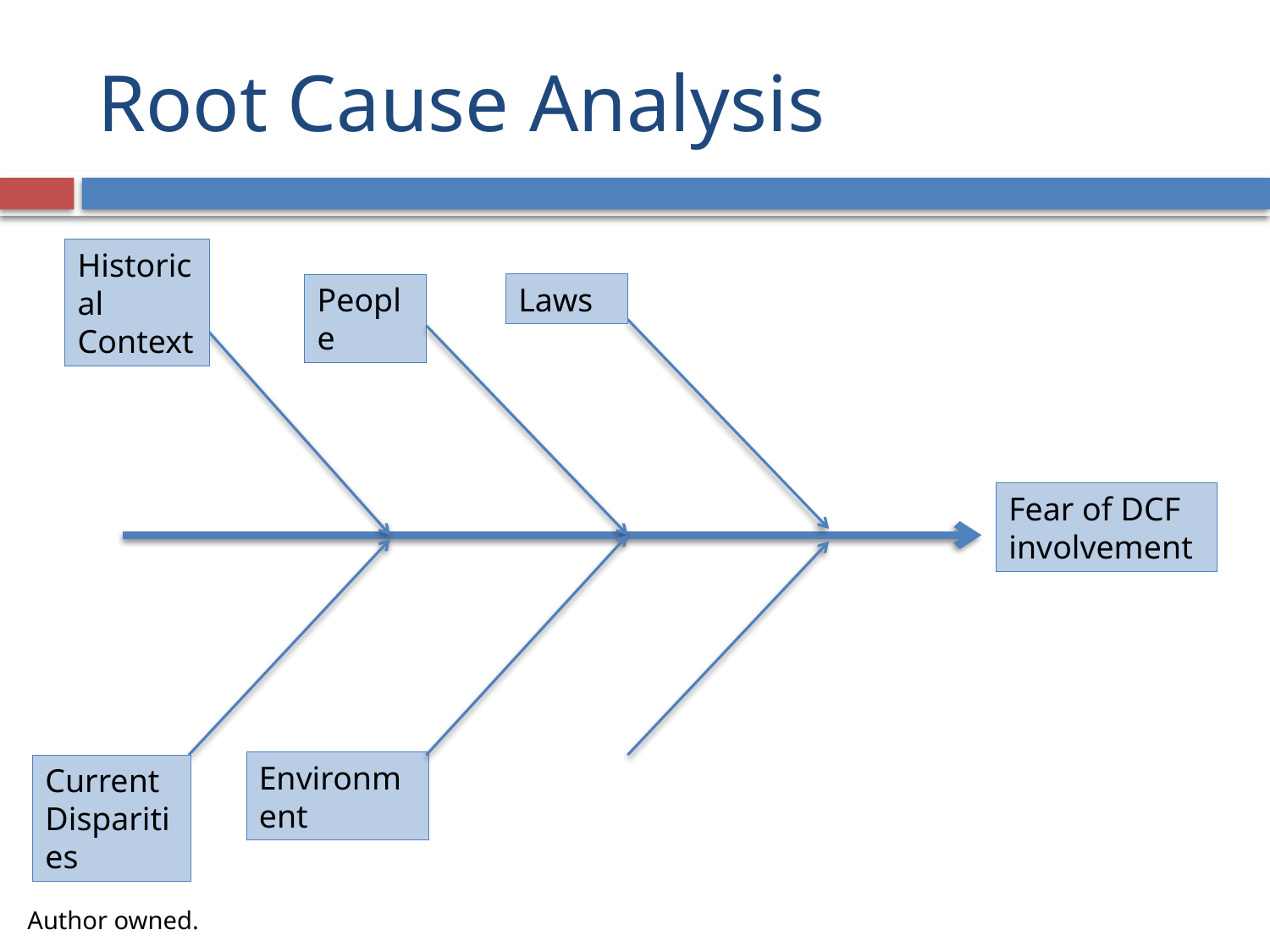

# Root Cause Analysis
Historical Context
Current Disparities
Fear of DCF involvement
Laws
People
Environment
Author owned.

## Slide 30
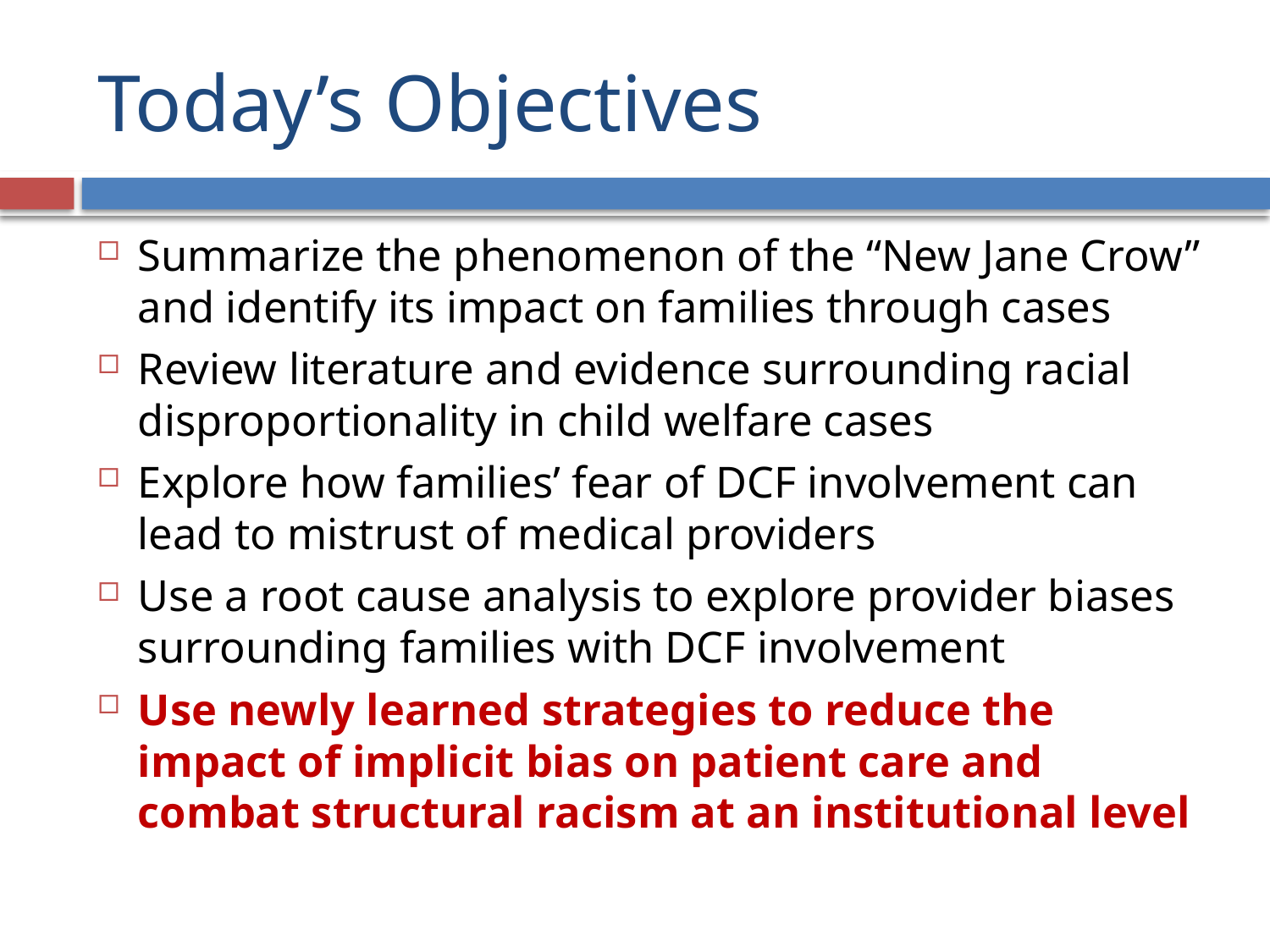

# Today’s Objectives
Summarize the phenomenon of the “New Jane Crow” and identify its impact on families through cases
Review literature and evidence surrounding racial disproportionality in child welfare cases
Explore how families’ fear of DCF involvement can lead to mistrust of medical providers
Use a root cause analysis to explore provider biases surrounding families with DCF involvement
Use newly learned strategies to reduce the impact of implicit bias on patient care and combat structural racism at an institutional level

## Slide 31
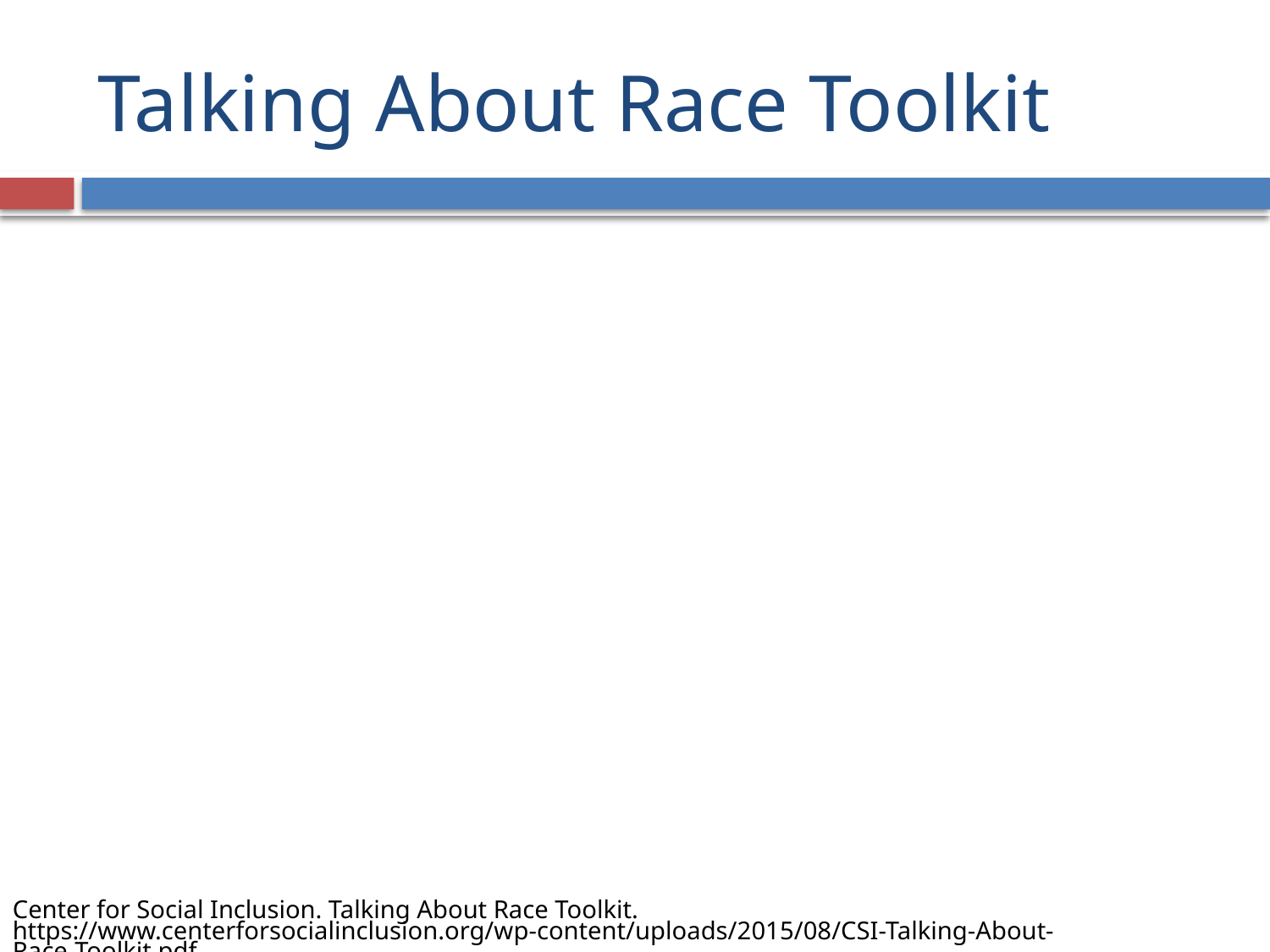

# Talking About Race Toolkit
Center for Social Inclusion. Talking About Race Toolkit. https://www.centerforsocialinclusion.org/wp-content/uploads/2015/08/CSI-Talking-About-Race-Toolkit.pdf

## Slide 32
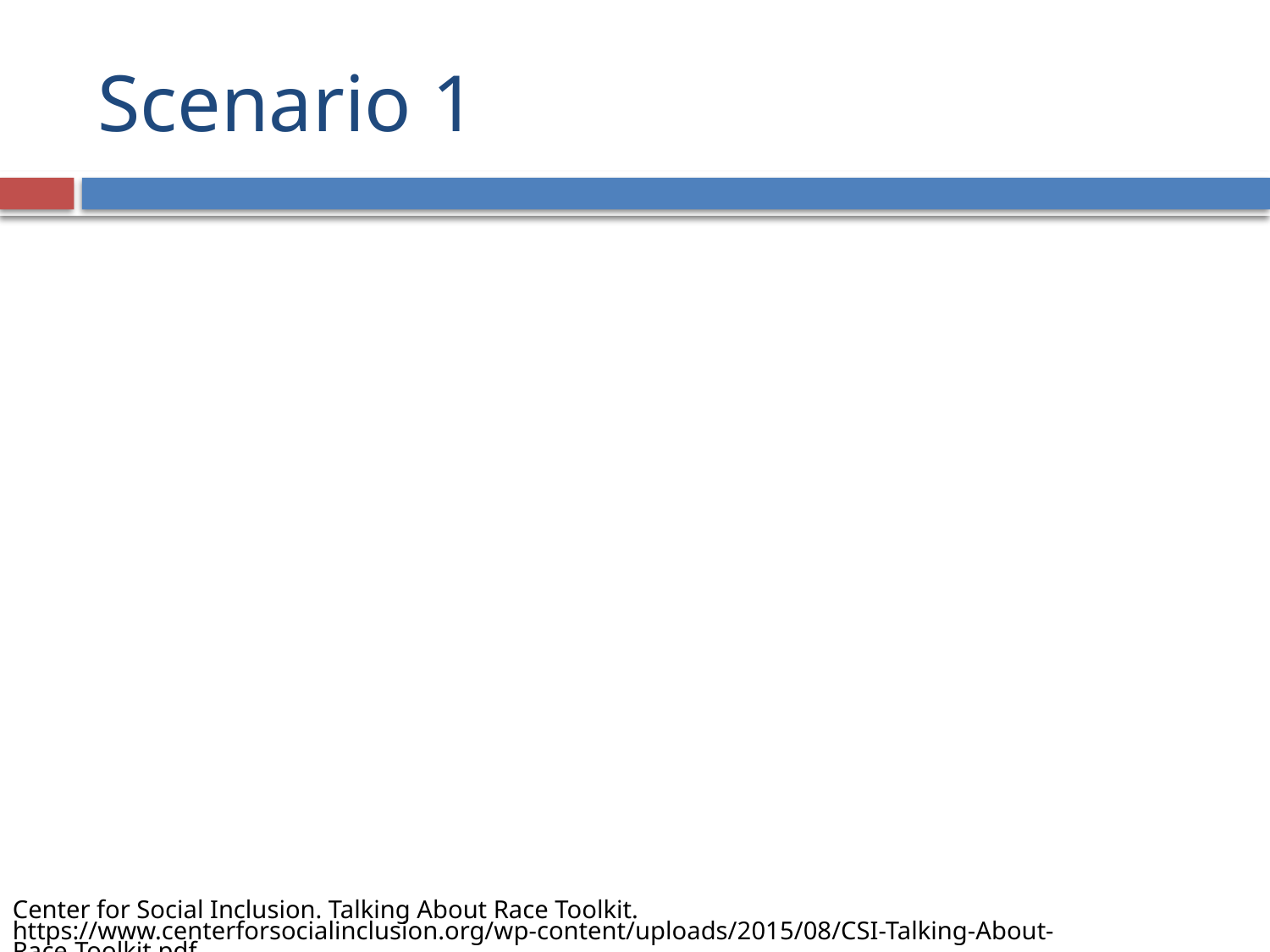

# Scenario 1
Center for Social Inclusion. Talking About Race Toolkit. https://www.centerforsocialinclusion.org/wp-content/uploads/2015/08/CSI-Talking-About-Race-Toolkit.pdf

## Slide 33
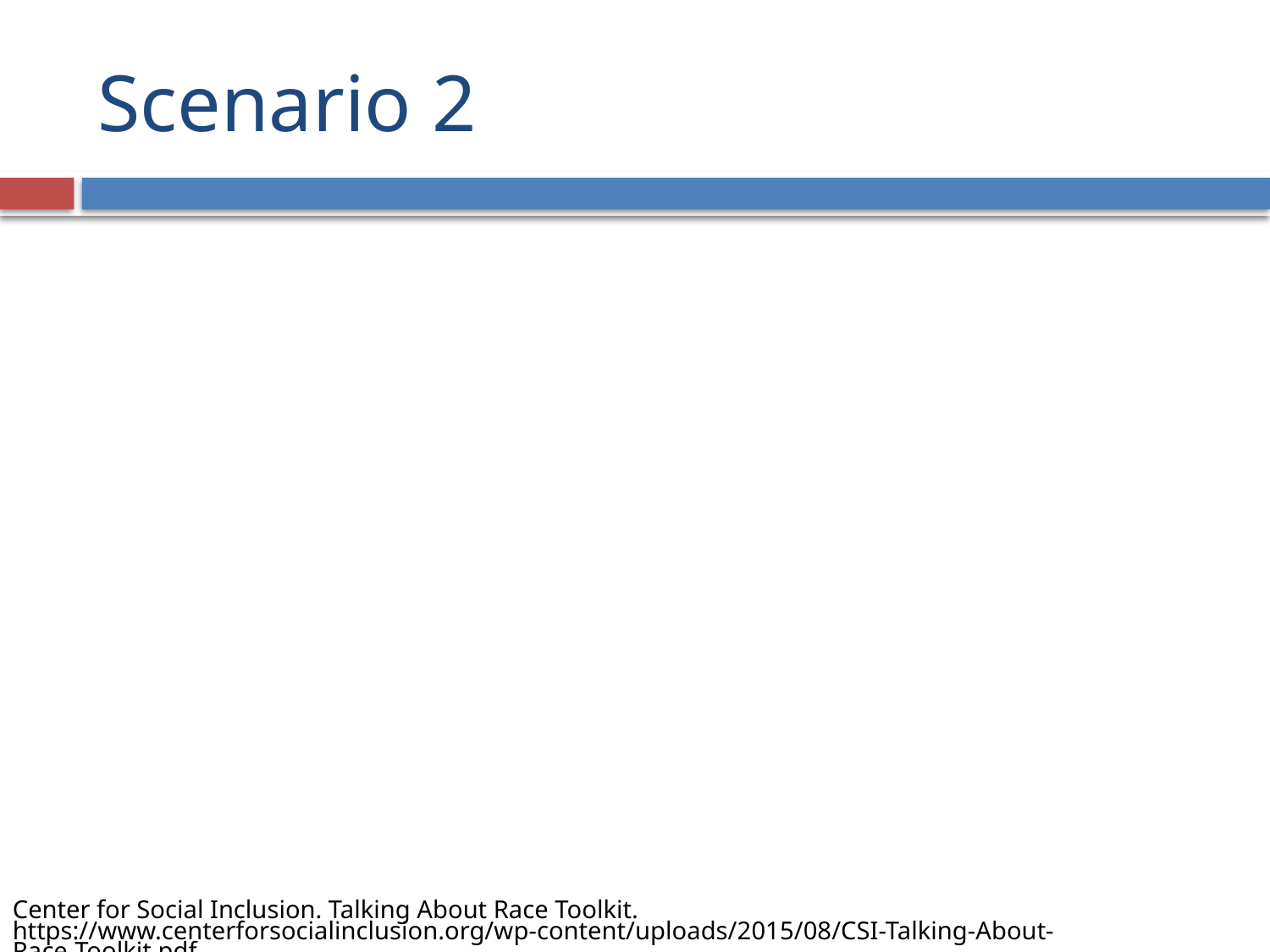

# Scenario 2
Center for Social Inclusion. Talking About Race Toolkit. https://www.centerforsocialinclusion.org/wp-content/uploads/2015/08/CSI-Talking-About-Race-Toolkit.pdf

## Slide 34
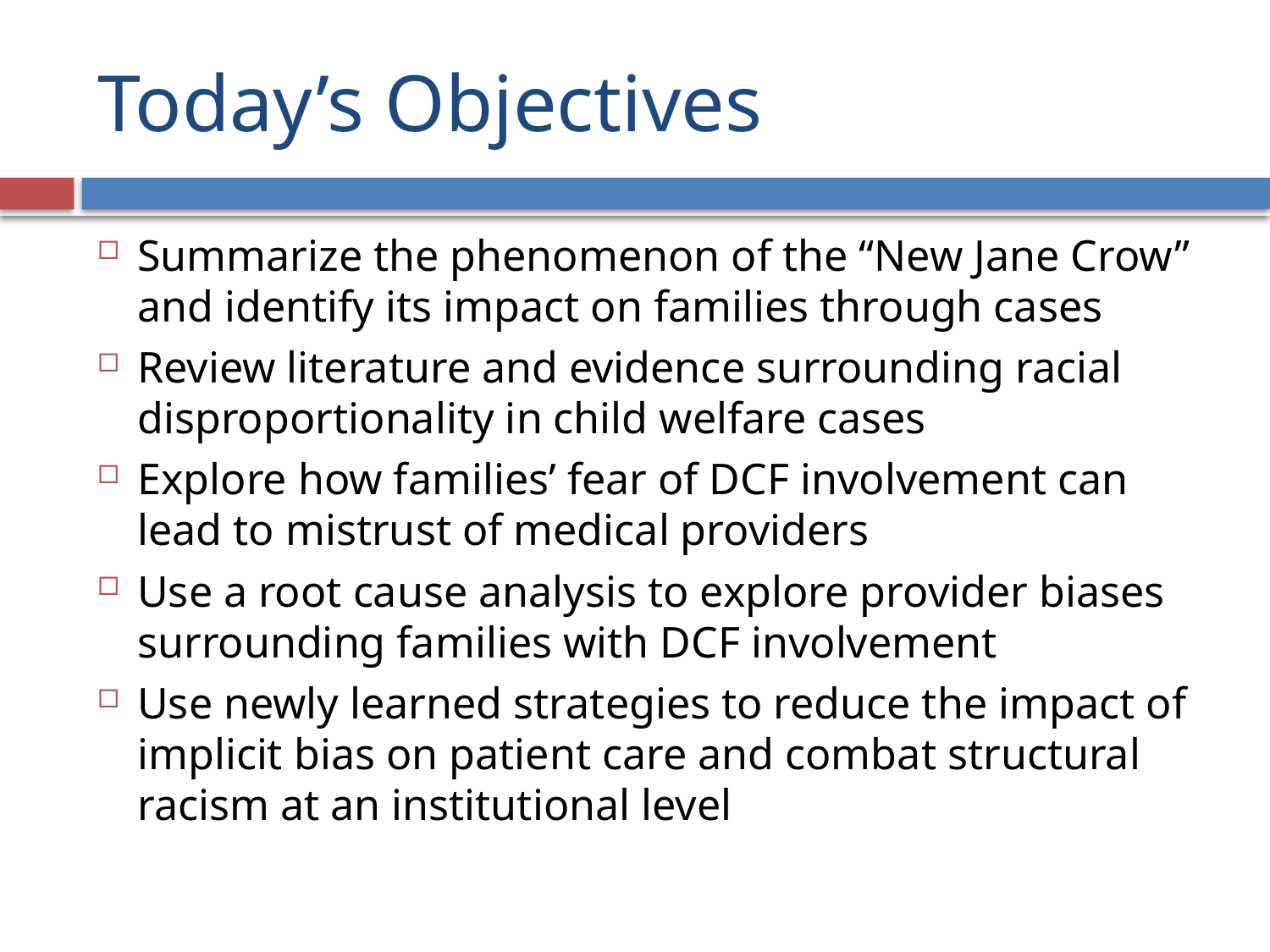

# Today’s Objectives
Summarize the phenomenon of the “New Jane Crow” and identify its impact on families through cases
Review literature and evidence surrounding racial disproportionality in child welfare cases
Explore how families’ fear of DCF involvement can lead to mistrust of medical providers
Use a root cause analysis to explore provider biases surrounding families with DCF involvement
Use newly learned strategies to reduce the impact of implicit bias on patient care and combat structural racism at an institutional level

## Slide 35
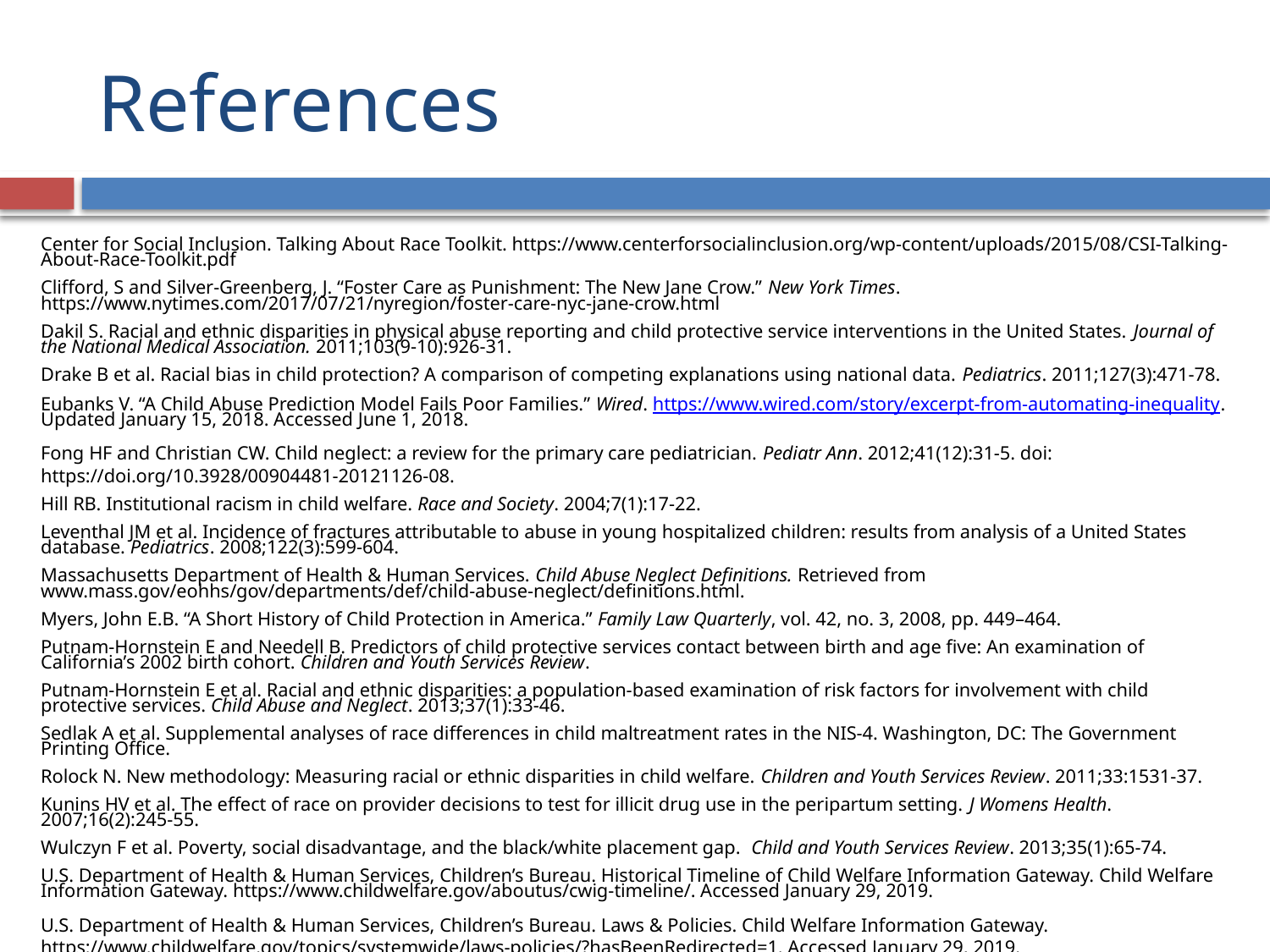

# References
Center for Social Inclusion. Talking About Race Toolkit. https://www.centerforsocialinclusion.org/wp-content/uploads/2015/08/CSI-Talking-About-Race-Toolkit.pdf
Clifford, S and Silver-Greenberg, J. “Foster Care as Punishment: The New Jane Crow.” New York Times. https://www.nytimes.com/2017/07/21/nyregion/foster-care-nyc-jane-crow.html
Dakil S. Racial and ethnic disparities in physical abuse reporting and child protective service interventions in the United States. Journal of the National Medical Association. 2011;103(9-10):926-31.
Drake B et al. Racial bias in child protection? A comparison of competing explanations using national data. Pediatrics. 2011;127(3):471-78.
Eubanks V. “A Child Abuse Prediction Model Fails Poor Families.” Wired. https://www.wired.com/story/excerpt-from-automating-inequality. Updated January 15, 2018. Accessed June 1, 2018.
Fong HF and Christian CW. Child neglect: a review for the primary care pediatrician. Pediatr Ann. 2012;41(12):31-5. doi: https://doi.org/10.3928/00904481-20121126-08.
Hill RB. Institutional racism in child welfare. Race and Society. 2004;7(1):17-22.
Leventhal JM et al. Incidence of fractures attributable to abuse in young hospitalized children: results from analysis of a United States database. Pediatrics. 2008;122(3):599-604.
Massachusetts Department of Health & Human Services. Child Abuse Neglect Definitions. Retrieved from www.mass.gov/eohhs/gov/departments/def/child-abuse-neglect/definitions.html.
Myers, John E.B. “A Short History of Child Protection in America.” Family Law Quarterly, vol. 42, no. 3, 2008, pp. 449–464.
Putnam-Hornstein E and Needell B. Predictors of child protective services contact between birth and age five: An examination of California’s 2002 birth cohort. Children and Youth Services Review.
Putnam-Hornstein E et al. Racial and ethnic disparities: a population-based examination of risk factors for involvement with child protective services. Child Abuse and Neglect. 2013;37(1):33-46.
Sedlak A et al. Supplemental analyses of race differences in child maltreatment rates in the NIS-4. Washington, DC: The Government Printing Office.
Rolock N. New methodology: Measuring racial or ethnic disparities in child welfare. Children and Youth Services Review. 2011;33:1531-37.
Kunins HV et al. The effect of race on provider decisions to test for illicit drug use in the peripartum setting. J Womens Health. 2007;16(2):245-55.
Wulczyn F et al. Poverty, social disadvantage, and the black/white placement gap.  Child and Youth Services Review. 2013;35(1):65-74.
U.S. Department of Health & Human Services, Children’s Bureau. Historical Timeline of Child Welfare Information Gateway. Child Welfare Information Gateway. https://www.childwelfare.gov/aboutus/cwig-timeline/. Accessed January 29, 2019.
U.S. Department of Health & Human Services, Children’s Bureau. Laws & Policies. Child Welfare Information Gateway. https://www.childwelfare.gov/topics/systemwide/laws-policies/?hasBeenRedirected=1. Accessed January 29, 2019.
