# Supplementary material for: Health Equity Rounds: An Interdisciplinary Case Conference to Address Implicit Bias and Structural Racism for Faculty and Trainees
Source: MedEdPORTAL. 2019 Nov 22;15:10858. doi: 10.15766/mep_2374-8265.10858 (PMC7050660; doi:10.15766/mep_2374-8265.10858)
Supplement: Supplementary file 1 — A. HER 1.pptx B. HER 2.pptx C. HER 3.pptx D. HER 4.pptx E. HER 5.pptx F. HER 6.pptx G. HER 7.pptx H. Selected HER Handouts.docx I. Case Conference Creation Guide.docx J. Glossary.docx K. Evaluation.docx [file mep-15-10858-s001.zip › H. Selected HER Handouts.docx]

**Health Equity Rounds 2 Worksheet**

Please use the following worksheet to guide you through the case and discussion.

1. Why do you do what you do? What values drive your work? **Please text in your response.**
2. What is your initial reaction to the ED board? **Please text in your response.**
3. Reflection: What is your gut reaction to this scenario as a provider? Does this remind you of any prior encounters? How would these prior encounters shape the way that you approach this clinical scenario?
4. Perspective-Taking: Imagine yourself in this mother’s shoes. How would you feel about the plan? What are you worried about? What would ED providers need to do to earn your trust?

Discussion

**­**

Next steps

**Health Equity Rounds 5 Handout**

1. Koretzky M, Bonham VL, Berkman BE, Kruszka P, Adeyemo A, Muenke M, Hull SC (2016) Towards a more representative morphology: clinical and ethical considerations for including diverse populations in diagnostic genetic atlases. Genet Med : Off J Am Coll Med Genet. doi:10.1038/gim.2016.7

2. Kahneman, D. Thinking, Fast and Slow. 1st ed. New York: Farrar, Straus and Giroux; 2011.

3. Garcia RS. The misuse of race in medical diagnosis. *Pediatrics*. 2004;113(5):1394-1395. doi:10.1542/PEDS.113.5.1394

**Health Equity Rounds 7 Worksheet**

Fishbone Diagram:

Use the following diagram to create a root cause analysis of a patient’s experience with bias.

Talking Race Tool Kit^[[1]](#endnote-1)^: A strategy for starting difficult conversations. *(An example conversation is provided in italics).*

Affirm: Affirm shared values and presumed good intentions on the part of the other person

*As pediatricians, it’s our duty to protect vulnerable children and I know that’s our first priority here.*

Counter: Explain why there is a problem in this specific situation, give historical context, state racial bias explicitly and non-judgmentally

*I'm concerned that we are prematurely filing with DCF. Statistically, this occurs more frequently in minority patients*. *(This last sentence is Important to add explicitly, but also the most difficult sentence to add).*

Transform: Propose a solution

*I think we should definitely start by reaching out to social work to complete their assessment, but we should inform them that other etiologies are still on our differential rather than telling them that this is our main concern. Let’s hold off on filing with DCF until social work has completed their evaluation.*

Your turn:

Affirm: Affirm shared values and presumed good intentions on the part of the other person

____________________________________________________________________________________

Counter: Explain why there is a problem in this specific situation, give historical context, state racial bias explicitly and non-judgmentally

____________________________________________________________________________________

Transform: Propose a solution

____________________________________________________________________________________

1. Talking About Race Toolkit. Center for Social Inclusion. https://www.centerforsocialinclusion.org/talking-race-toolkit/. Accessed January 30, 2019. [↑](#endnote-ref-1)
