# Supplementary material for: Health Equity Rounds: An Interdisciplinary Case Conference to Address Implicit Bias and Structural Racism for Faculty and Trainees
Source: MedEdPORTAL. 2019 Nov 22;15:10858. doi: 10.15766/mep_2374-8265.10858 (PMC7050660; doi:10.15766/mep_2374-8265.10858)
Supplement: Supplementary file 1 — A. HER 1.pptx B. HER 2.pptx C. HER 3.pptx D. HER 4.pptx E. HER 5.pptx F. HER 6.pptx G. HER 7.pptx H. Selected HER Handouts.docx I. Case Conference Creation Guide.docx J. Glossary.docx K. Evaluation.docx [file mep-15-10858-s001.zip › I. Case Conference Creation Guide.docx]

**Guide to Creating a Health Equity Rounds (HER) Conference**

1. **Case and topic selection**

- Select a case and topic for discussion related to implicit bias and structural racism
- Cases may arise from members of the HER team and/or may be solicited through department-wide emails or discussions. In some instances, cases can inspire the topic of the conference. However, sometimes a topic can be chosen first and then a case vignette can be selected/solicited to address/highlight that topic.

1. **Initial development of materials**

- **Develop initial learning objectives**
- **Prepare initial case slides**
  - Condense and tailor the case to fit learning objectives
  - Rid the case of unnecessary clinical data that might distract participants from objectives
- **Literature Review**
  - Take a broad approach to reviewing the literature, including academic articles, books, and articles/other media in the popular press in the search
  - Investigate the historical and institutional roots of the inequities discussed
  - Investigate the impact of the inequities, specific forms of implicit bias, and certain discriminatory practices relevant to the topic at hand

1. **Prepare slide deck and logistics of conference**

- **Introduction**
  - Adapt standard HER introductory slides including introduction to HER, introduction to implicit bias, conceptual framework, program and goals and objectives, ground rules and shared values
  - Add any additional relevant terms to the Glossary handout
- **Case slides**
  - Consider creative ways to present case to optimize audience engagement (see HER conference examples for suggested models)
  - Aim to make case rich in sensory detail but less rich in clinical data in order to dissuade discussion from the medical details of the case
  - Infuse evidence-based tools to combat implicit bias (e.g. reflection, perspective taking, empathy building)
- **Specific conference objectives**
  - These are distinct from HER program objectives
- **Didactic portion**
  - Present relevant historical context and evidence of impact of the inequities related to the topic at hand
- **Discussion**
  - Choose a skilled moderator
  - Craft targeted questions for audience members
  - May have selected discussants who are experts in the field or have specific experiences to share
- **Advocacy and next steps**
  - Provide opportunities for advocacy occurring on personal, institutional, local/national levels that address the relevant topic(s)
  - Invite people who can speak to these efforts
  - Prepare handout of advocacy resources and/or prepare to email audience members about these tools

1. **Adapt Evaluation**

- If IRB approval is required to collect evaluation data, ensure this is done in a timely fashion
- Decide on any changes that will be made to general evaluation form (e.g. inquiring about specific aspects of the conference)
- If any evaluative steps will be done during conference, integrate these into the case conference

Suggested Timeline

- 8-12 weeks before HER
  - Choose topic/case
  - Obtain IRB approval if needed to collect evaluation data
- 6-8 weeks before HER
  - Begin literature review
  - Compile case information
  - Invite any speakers or special audience members
  - Work on evaluation, including any specific questions that may change and any updates to IRB that would need to be made
- 4 weeks before HER
  - Work over next 1-2 weeks to bring case and literature review into presentable slides, add in discussion questions, add in advocacy points and have first fully completed draft done
- 2 weeks before HER
  - Obtain edits and comments from advisors and other members of the HER team
- 1 week before HER
  - Make final edits to presentation
  - Practice presentation delivery
- 2-3 days before HER
  - Print paper materials (eg. evaluations, glossary, advocacy materials)
- Immediately to a few days after HER
  - Hold debrief with HER team
  - Send thank you notes to discussants/moderator
- 1-2 weeks after HER
   Compile evaluation results
