# Supplementary material for: Health Equity Rounds: An Interdisciplinary Case Conference to Address Implicit Bias and Structural Racism for Faculty and Trainees
Source: MedEdPORTAL. 2019 Nov 22;15:10858. doi: 10.15766/mep_2374-8265.10858 (PMC7050660; doi:10.15766/mep_2374-8265.10858)
Supplement: Supplementary file 1 — A. HER 1.pptx B. HER 2.pptx C. HER 3.pptx D. HER 4.pptx E. HER 5.pptx F. HER 6.pptx G. HER 7.pptx H. Selected HER Handouts.docx I. Case Conference Creation Guide.docx J. Glossary.docx K. Evaluation.docx [file mep-15-10858-s001.zip › J. Glossary.docx]

*Glossary originally developed by the Boston Public Health Commission, with additional terms incorporated by the Health Equity Rounds team.*

**Ethnicity**: A concept referring to a shared culture and way of life.^1^

**Health Disparities:** Differences between the health of one population and another in measures of who gets disease, who has disease, who dies from disease, and other adverse health conditions that exist among specific population groups in the US.^2^

**Healthcare Disparities:** Differences among populations in access to or availability of facilities and services.^3^

**Health Equity:** The opportunity for everyone to attain his or her full health potential. No one is disadvantaged from achieving this potential because of his or her social position (e.g. class, socioeconomic status) or socially assigned circumstance (e.g. race, gender, ethnicity, religion, sexual orientation, geography, etc.).^4^

**Health Inequities:** Differences in health status and mortality rates across population groups that are systemic, avoidable, unfair, and unjust. These differences are rooted in social and economic injustice, and are attributable to social, economic and environmental conditions in which people live, work, and play.^4^

**Implicit Bias:** Learned stereotypes and prejudices that operate automatically, and unconsciously, when interacting with others. Also referred to as *unconscious bias.*^5^

**Intersectionality:** A framework first developed by Kimberlé Crenshaw^6^ that conceptualizes a person, group of people, or social problem as affected by a number of discriminations and disadvantages, taking into account overlapping identities and experiences to understand prejudices faced.^7^

**Oppression**: Unjust use of power and authority.^8^

**People of Color:** A political construct created by People of Color to describe people who would generally not be categorized as White.^8^

**Prejudice**: An unfavorable opinion or feeling formed beforehand or without knowledge, thought, or reason.^9^

**Privilege**: Advantages and immunities enjoyed by one, usually powerful group or class, especially to the disadvantage of others. **White Privilege:** Advantages and immunities enjoyed by whites in the US.^8^

**Race**: A socially constructed way of grouping people, based on skin color and other apparent physical differences, which has no genetic or scientific basis. This social construct was created and used to justify social and economic oppression of people of color by Whites.^10^

**Racial Discrimination:** Unfair treatment because of an individual's actual or perceived racial or ethnic background.^11^

**Racial Justice:** The creation and proactive reinforcement of policies, practices, attitudes, and actions that produce equitable power, access, opportunities, treatment and outcomes for all people, regardless of race.^12^

**Racism:** A system of advantage based on race.^13^

§ **Internalized Racism** - The set of private beliefs, prejudices, and ideas that individuals have about the superiority of Whites and the inferiority of people of color. Among people of color, it manifests as internalized racial oppression. Among Whites, it manifests as internalized racial superiority.^8^

§  **Interpersonal Racism** - The expression of racism between individuals. These are interactions occurring between individuals that often take place in the form of harassing, racial slurs, or telling of racial jokes.^8^

§ **Institutional Racism** - Discriminatory treatment, unfair policies and practices, and inequitable opportunities and impacts within organizations and institutions, based on race.^8^

§  **Structural Racism** - Racial bias across institutions and society over time. It is the cumulative and compounded effects of an array of factors such as public policies, institutional practices, cultural representations, and other norms that work in various, often reinforcing, ways to perpetuate racial inequity.^8^

**Social Determinants of Health:** The circumstances in which people are born, grow, live, work, play, and age that influence access to resources and opportunities that promote health. The social determinants of health include housing, education, employment, environmental exposure, health care, public safety, food access, income, and health and social services.^14^

**Stereotype**: A standardized mental picture that is held in common about members of a group that represents an oversimplified opinion, attitude, or unexamined judgment, without regard to individual difference.^8^

Definitions were adapted from the following sources:

1. Johnson AG. *The Blackwell Dictionary of Sociology: A User’s Guide to Sociological Language*. 2nd ed. Malden, MA: Blackwell; 2000.

2. Office of Disease Prevention and Health Promotion. Disparities. Healthy People 2020. https://www.healthypeople.gov/2020/about/foundation-health-measures/Disparities. Accessed January 18, 2019.

3. Orgera K, Artiga S. Disparities in Health and Health Care: Five Key Questions and Answers. Kaiser Family Foundation. https://www.kff.org/disparities-policy/issue-brief/disparities-in-health-and-health-care-five-key-questions-and-answers/#footnote-267499-2. Published 2018. Accessed January 19, 2019.

4. Whitehead M, Dahlgren G. *Concepts and Principles for Tackling Social Inequities in Health: Levelling up Part 1*. Copenhagen; 2006.

5. Staats C, Capatosto K, Wright RA, Contractor D. *State of the Science: Implicit Bias Review 2015*.; 2015. http://kirwaninstitute.osu.edu/wp-content/uploads/2015/05/2015-kirwan-implicit-bias.pdf. Accessed January 19, 2019.

6. Crenshaw K. *Demarginalizing the Intersection of Race and Sex: A Black Feminist Critique of Antidiscrimination Doctrine, Feminist Theory and Antiracist Politics Recommended Citation Crenshaw, Kimberle () &quot;Demarginalizing the Intersection of Race and Sex: A Black Feminist Critique of Antidiscrimination Doctrine, Feminist Theory and Antiracist Politics*.; 1989. http://chicagounbound.uchicago.edu/uclfhttp://chicagounbound.uchicago.edu/uclf/vol1989/iss1/8. Accessed January 22, 2019.

7. YW Boston. What is intersectionality, and what does it have to do with me? YW Boston Blog. https://www.ywboston.org/2017/03/what-is-intersectionality-and-what-does-it-have-to-do-with-me/. Published 2017. Accessed January 22, 2019.

8. Boston Public Health Commission. Racial Justice and Health Equity Initiative: Core Workshop Facilitator’s Guide. 2011.

9. Prejudice. Dictionary.com. https://www.dictionary.com/browse/prejudice. Accessed January 19, 2019.

10. Adelman L, Cheng J, Shim I. *Race: The Power of Illusion*. California Newsreel; 2003.

11. Smedley B, Stith A, Nelson A. *Unequal Treatment: Confronting Racial and Ethnic Disparities in Health Care*. Washington, D.C.: National Academies Press; 2002.

12. About Race Forward. Race Forward. https://www.raceforward.org/about. Accessed January 19, 2019.

13. Wellman DT. *Portraits of White Racism*. Cambridge; New York: Cambridge University Press; 1993.

14. Office of Disease Prevention and Health Promotion. Social Determinants of Health. Healthy People 2020. https://www.healthypeople.gov/2020/topics-objectives/topic/social-determinants-of-health. Accessed January 18, 2019.
