# Supplementary material for: Health Equity Rounds: An Interdisciplinary Case Conference to Address Implicit Bias and Structural Racism for Faculty and Trainees
Source: MedEdPORTAL. 2019 Nov 22;15:10858. doi: 10.15766/mep_2374-8265.10858 (PMC7050660; doi:10.15766/mep_2374-8265.10858)
Supplement: Supplementary file 1 — A. HER 1.pptx B. HER 2.pptx C. HER 3.pptx D. HER 4.pptx E. HER 5.pptx F. HER 6.pptx G. HER 7.pptx H. Selected HER Handouts.docx I. Case Conference Creation Guide.docx J. Glossary.docx K. Evaluation.docx [file mep-15-10858-s001.zip › K. Evaluation.docx]

Thank you in advance for participating in Health Equity Rounds. We are relying your feedback to improve the event’s structure and content. We would greatly appreciate if you filled out this anonymous survey during or after the conference today. We are offering two methods for filling out the program evaluation: online and paper. Please only complete one survey.

**Are you from the Department of Pediatrics or a different department? (Please circle one)**

Pediatrics

Other (please identify) _______________________

**Role (Please circle all that apply)**

Resident Attending Administrator

Social worker Fellow Applicant

Medical student Nurse Other ______________

**Meeting Objectives | After participating in Health Equity Rounds, please rate your abilities to do the following:**

|  | Please circle one | Comments |
| --- | --- | --- |
| Identify and analyze the effects of implicit bias and structural racism in clinical scenarios | Yes  No  Partially  I don’t know |  |
| Describe the historical context and present-day role of structural racism and its impact on the health care system | Yes  No  Partially  I don’t know |  |
| Employ evidence-based tools to recognize and mitigate personally held implicit biases | Yes  No  Partially  I don’t know |  |
| Use newly learned strategies to combat structural racism at the institutional level and reduce the impact of implicit bias on patient care and inter-professional relationships | Yes  No  Partially  I don’t know |  |

**How would you rate the educational value of this activity overall?**

| 1  Poor | 2 | 3 | 4 | 5  Excellent |
| --- | --- | --- | --- | --- |
|  |  |  |  |  |

**Would you be interested in attending another Health Equity Rounds?**

| Yes |  |
| --- | --- |
| No |  |
| No sure |  |

**Did we provide enough non-clinical background information to stimulate discussion?**

| 1  There was not enough background information | 2 | 3  There was the appropriate amount of background information | 4 | 5  There was too much background information |
| --- | --- | --- | --- | --- |
|  |  |  |  |  |

**To what extent was the conference engaging today?**

| 1  Not engaging | 2 | 3  Somewhat engaging | 4 | 5  Very engaging |
| --- | --- | --- | --- | --- |
|  |  |  |  |  |

**Did any aspect(s) of this discussion feel inappropriate?**

| Yes |  |
| --- | --- |
| No |  |
| Not sure |  |

Please elaborate:

Were you ever offended at any point during Health Equity Rounds?

| Yes |  |
| --- | --- |
| No |  |
| Not sure |  |

Please elaborate:

**Will what you learned in this session impact your practice?**

| Yes |  |
| --- | --- |
| No |  |
| Not sure |  |

If yes, what will you change or will you do differently?

If no, why not?

**Please use this space for any other comments, suggestions, or feedback.**
